# Proteome-Wide Discovery of Degradable Proteins Using Bifunctional Molecules

**DOI:** 10.1021/acscentsci.5c01594

**Published:** 2025-10-21

**Authors:** Ines Forrest, Louis P. Conway, Clara Gathmann, Appaso M. Jadhav, Tzu-Yuan Chiu, Christian M. Chaheine, Michelle Estrada, Anurupa Shrestha, Kathy Sarris, Justin M. Reitsma, Scott E. Warder, Anil Vasudevan, Shaun M. McLoughlin, Christopher G. Parker

**Affiliations:** † Department of Chemistry, 4356 The Scripps Research Institute, La Jolla, California 92130, United States; ‡ Skaggs Graduate School of Chemical and Biological Sciences, La Jolla, California 92130, United States; § Technology and Therapeutic Platforms, Discovery Research, AbbVie Inc., North Chicago, Illinois 60064, United States

## Abstract

Targeted protein degradation (TPD) is an emergent therapeutic strategy with the potential to circumvent challenges associated with targets unamenable to conventional pharmacological inhibition. Among TPD approaches, proteolysis-targeting chimeras (PROTACs) have shown marked advancement, with numerous candidates in clinical development. Despite their potential, most PROTACs utilize advanced small-molecule inhibitors, inherently limiting the scope of this approach. More generally, the fraction of the proteome tractable to small-molecule-induced degradation strategies is unknown. Here we describe a chemical proteomic strategy for the agnostic discovery of degradable proteins in cells using a new class of bifunctional degrader molecules called “AgnoTACs”. Proteome-wide screening of 72 AgnoTACs in human cells uncovered downregulation events spanning >50 functionally and structurally diverse proteins, most of which lack chemical probes. While many events progressed through canonical degradation pathways, we also observed instances of alternative mechanisms, indicating that AgnoTACs can impact protein stability via multiple modes of action. Our findings highlight the potential of function-biased chemical libraries coupled with proteomic profiling to discover degrader starting points as well as furnish a blueprint for expanding our understanding of the chemically degradable proteome.

## Graphical abstract

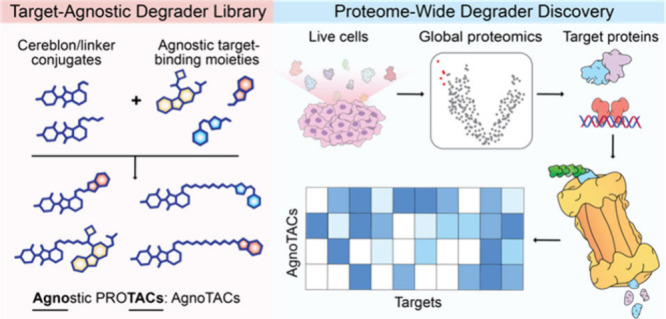

## Introduction

The human genome encodes more than 20,000 proteins, yet only a small fraction has useful chemical probes available to investigate their function.^1^ Conventional chemical probe and drug discovery efforts have focused mainly on the identification of active/functional site inhibitors. Although this approach is highly successful for target protein classes such as enzymes and receptors, it is inevitably limited to proteins possessing functionally well-defined, druggable sites.^2^ By harnessing the cell’s natural degradation machinery to eliminate proteins, targeted protein degradation (TPD) methods, such as proteolysis targeting chimeras (PROTACs), offer a compelling alternative.^3,4^ PROTACs are heterobifunctional molecules comprising a target binding moiety (TBM) linked to an E3 ligase binding moiety (EBM), which induce proximity between an E3 ligase and protein of interest (POI), facilitating directed ubiquitination of POIs and leading to their subsequent degradation.^5,6^ PROTACs possess distinct advantages over conventional small molecule inhibition, including (i) a catalytic mechanism of action (MoA), (ii) the potential for increased selectivity owing to mandatory ternary complex formation, (iii) the ability to engage a target protein potentially anywhere on its surface rather than being limited to functional sites, and (iv) the ability to disrupt multiple protein functions (e.g., scaffolding roles).^7,8^ Not surprisingly, PROTACs have now been developed for a multitude of disease areas and have shown promising preliminary results in clinical trials.^5,9,10^ However, despite their potential, the vast majority of PROTACs utilize pre-existing small molecule inhibitors as TBMs, including all disclosed structures of those currently in clinical development. For instance, ARV-471, an estrogen receptor (ER) degrader, is based on the known ER antagonist Lasofoxifene.^11^ Similarly, ARV-110 which degrades the androgen receptor (AR) is derived from enzalutamide AR inhibitor scaffolds,^12^ and KT-333 that targets STAT3-dependent pathologies, originates from SI-109, a STAT3 SH2 domain inhibitor.^13^ This reliance inherently restricts PROTAC scope to targets possessing well-characterized, advanced ligands.

In this vein, powerful “binding-first” screening approaches, including DNA-encoded libraries (DELs) and chemical proteomics, have demonstrated potential to furnish small molecules that can be co-opted for TPD. For example, DEL-based and other high-throughput synthesis-coupled screens have recently been deployed for the discovery of molecular glues.^14–20^ Chemical proteomic screening has broadened the available repertoire of co-optable E3 ligase components^21–27^ and enabled the discovery of molecular glues.^28–30^ Chemical proteomic-based degradation readouts have also facilitated target identification of bioactive compounds through conversion into PROTACs.^31,32^ In a more recent study, a PROTAC-based degrader for metallothionein 2A (MT2A) was discovered by proteomic screening of a covalent ligand derivatized with a VHL E3 ligase recruiter.^33^ Despite the potential of these approaches, to date, they have largely been limited to targeting predefined proteins, the identification of IMiD-induced CRBN neosubstrates and the discovery of new covalent ligand TBMs or EBMs. More generally, the operational path to convert “binders” into functional compounds is often not straightforward. Critical challenges include prerequisite knowledge of a given pocket to accommodate modified ligands for proximity induction with concomitant effector proteins. Additionally, in the context of TPD, ligand binding itself may influence protein stability through a variety of mechanisms, potentially complicating differentiation of binders and designed degraders.^34–36^ Finally, since many of these screening approaches are often target specific, they require target-dependent readouts and available ligands that can accommodate further synthetic derivatization.

Inspired by these concepts, we report a chemical proteomic strategy that integrates target-agnostic libraries of small molecules that are functionally biased toward protein degradation with quantitative proteomics to discover degradable proteins proteome-wide. With this goal in mind, we designed a series of 72 target-agnostic PROTACs, termed AgnoTACs, composed of cereblon (CRBN) ligase ligands,^10^ chemically linked to structurally diverse, drug-like small molecules. Proteome-wide profiling of this library in human cancer cells revealed that AgnoTACs can mediate the degradation of 50+ proteins, including enzymes, transcription factors, adaptor proteins, transporters, regulatory proteins and protein complexes. Mechanistic investigations demonstrated that AgnoTAC degradation events often proceed through expected CRBN- and proteasome-mediated pathways, though alternative degradation pathways were also encountered. We observed that chemical features of AgnoTACs, including linker length and composition, not only impact apparent potency of degradation for given targets but also proteome-wide selectivity. Further, preliminary studies highlight the potential of AgnoTACs to serve as “pathfinder” chemical tools for functional investigations of proteins. These results underline how proteome-wide profiling of target-agnostic, yet function-focused chemical libraries can expedite the discovery of small molecule degraders for a broad spectrum of human proteins.

## Results

### Proteome-Wide Profiling of AgnoTACs in Human Cells

Recent investigations of PROTACs based upon promiscuous kinase and HDAC inhibitors have provided valuable insights into principles governing TPD.^37–39^ Specifically, these studies revealed that key factors traditionally associated with successful small-molecule inhibition, such as high target affinity and cellular target engagement (TE), do not reliably predict degradation efficiency or selectivity. Motivated by these findings, we rationalized that PROTAC libraries composed of relatively low molecular weight, drug-like ligands lacking predefined targets could serve as starting points to achieve degradation of proteins.

Toward this end, we designed and synthesized a library of 72 AgnoTAC molecules composed of thalidomide-based CRBN ligands conjugated to 18 structurally diverse small molecules possessing drug-like properties through four different linkers (Figures 1a and S1).^40^ Though, in theory, any established EBM could be utilized for E3 ligase recruitment, we chose well-established CRBN ligands due to their favorable drug-like properties and established track record in clinical trials.^41^ Likewise, we selected simple linear polyethylene glycol, or PEG (PEG2 and PEG3), and alkyl linkers (C2 and C5) due to their conformational flexibility and prevalence among reported PROTACs.^42,43^ The 18 TBMs were designed by benchmarking their molecular descriptors against the distribution of DrugBank ligands (Figure S2), while ensuring they remained amenable to straightforward coupling methods and generally commercially available. Specifically, candidate scaffolds were evaluated and optimized across key parameters including molecular weight, calculated LogP (cLogP), hydrogen bond donor and acceptor counts, rotatable bond count, aromatic ring content, polar surface area, and quantitative estimate of drug-likeness (QED). We further incorporated measures of 3D shape and stereochemical complexity, including normalized principal moments of inertia (NPR) and plane of best fit (PBF), to capture diverse molecular geometries. Final TBMs were selected to maximize chemical diversity (assessed by Tanimoto coefficient similarity) while residing within the physicochemical space characteristic of clinically approved compounds. AgnoTACs were first evaluated for their impact on cell viability in human MDA-MB-231 triple-negative breast cancer cells, as highly cytotoxic compounds could impact protein abundances through indirect mechanisms.^44^ Overall, we observed minimal viability effects for most library members (Figure S3). More recently, a wide variety of electrophiles have been shown to promote stress granule formation and alter protein abundance at elevated concentration.^45^ By contrast, high concentrations of AgnoTACs did not elicit stress granule formation in cells (Figure S4).

**Figure 1:**
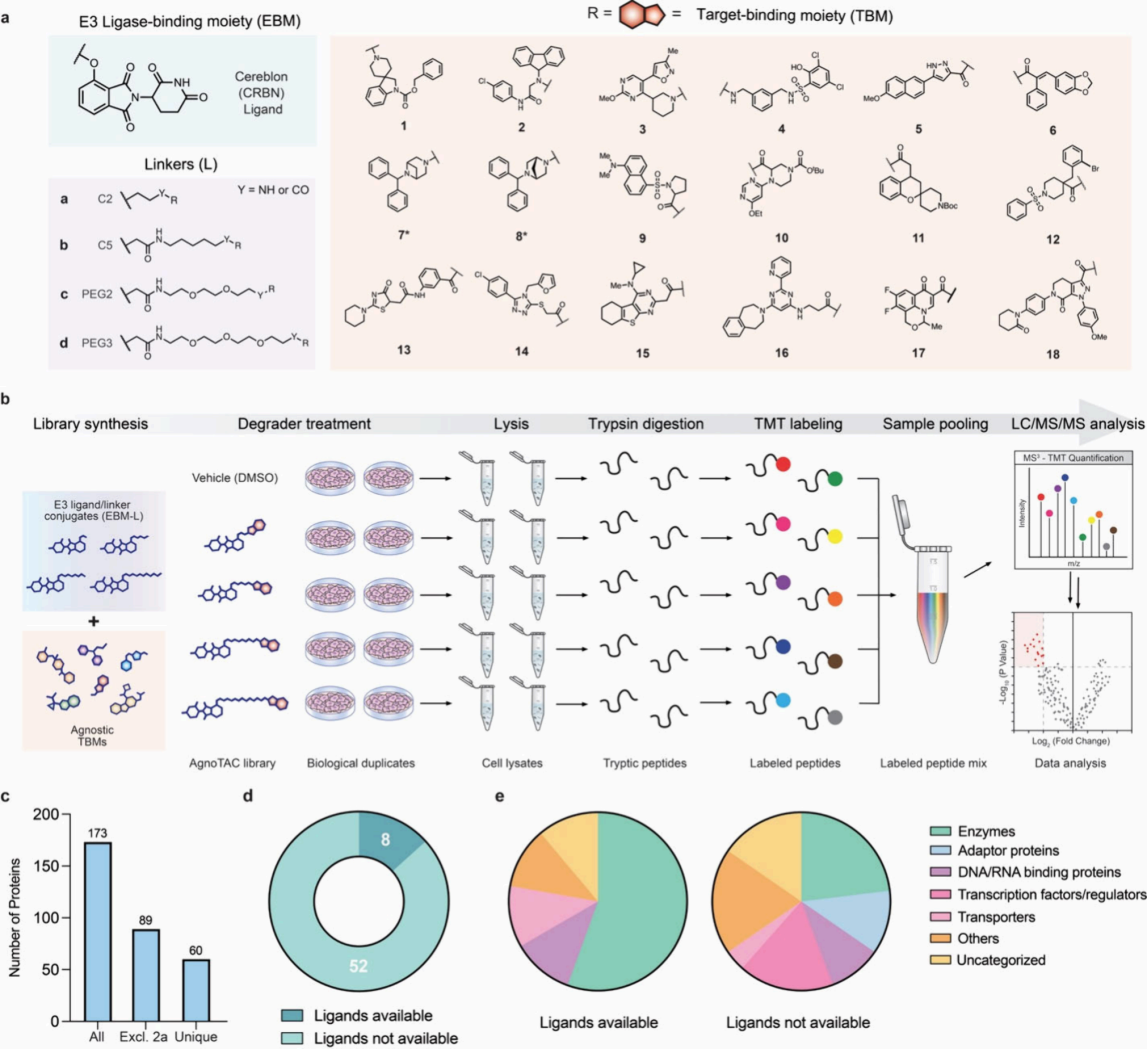
**Chemical proteomic strategy to interrogate the degradable proteome.** (a) Chemical structures of 72 AgnoTACs designed with CRBN E3 ligand, PEG or aliphatic linkers, and 18 diverse target-binding headgroups. Compounds marked with an asterisk (*) contain an aryl ether linkage rather than an oxyacetamide group. (b) Chemical proteomic workflow to identify degraded proteins. Cells are incubated with AgnoTAC library members (18 h treatment), followed by lysis, tryptic digestion, labeling with tandem mass tags (TMT), and protein identification and quantitation by LC-MS analysis (Supplemental Dataset 1). (c) Number of downregulated targets, defined as proteins displaying >2-fold decrease in MS^3^ abundance relative to DMSO and *p* < 0.05 across duplicate experiments (excl. = excluding). (d, e) Categorization of AgnoTAC-downregulated targets based on existence of ligands in DrugBank and protein class distribution (associated data can be found in Supplemental Dataset 2).

We next employed quantitative proteomics to identify potential targets by measuring changes in protein abundance in response to treatment with each AgnoTAC. Given that library members exhibited minimal cytotoxicity, we sought to maximize the discovery of potential degradable proteins. F01–F09 AgnoTACs, which predominantly feature fragment-like head groups were screened at 100 μM, while F10–F18 degraders, containing higher molecular weight head groups, were tested at 50 μM. Adopting a TMT-based proteomics workflow^46^ (Figure 1b), we quantified 8037 unique proteins, among which 173 showed substantial decreases in abundance (>2-fold relative to DMSO and *p* < 0.05 across duplicate experiments; Figures 1c and S5 and Supplemental Dataset 1). We noted that **2a** downregulated a relatively large number of targets (i.e., ∼85), which we attributed to potential secondary effects emanating from its apparent cytotoxicity (Figure S3b). To mitigate the inclusion of false positives (e.g., indirect abundance changes related to cytotoxicity) we excluded this AgnoTAC from our analysis, resulting in 60 uniquely downregulated targets (Figures 1c and S6a and Supplemental Dataset 1). Despite the structurally simple TBMs, we observed each AgnoTAC to be remarkably selective, with the vast majority inducing downregulation of a single protein (Figure S6a and Supplemental Dataset 1). Additionally, we identified cores (e.g., F02, F07, F08, F12, F15) which induce broader proteomic effects, while some were found to minimally affect protein abundances (e.g., F04, F09, F13, F14, F16–F18). We also evaluated a subset of our library at lower concentrations (10 μM) and shorter time points (6 h) to assess whether potential targets might be missed under our conditions but generally observed relatively muted profiles (Figures S7 and S8). We observed that a significant portion of targets fall out of traditional “druggable” classes, including adaptor proteins and transcription factors (Figure 1d,e) with only a small fraction (∼13%) possessing known functional ligands (largely enzymes) as estimated by their presence or absence in the DrugBank database (Figures 1d,e and S6b,c). In contrast, the larger subset of targets (∼87%) lacking reported bioactive ligands showed a broader functional distribution, with a reduced fractional representation of enzymes counterbalanced by expanded coverage of DNA/RNA binding proteins, transcription factors/regulators, and functionally uncategorized proteins (Figures 1d,e and S6b,c). In addition, recent studies have shown that certain CRBN-based degraders can reduce the abundance of short half-life proteins via indirect mechanisms, which can potentially complicate mechanistic interpretation.^44^ We examined reported half-lives of targets across all AgnoTACs and found that the vast majority are not short-lived (Figure S9), suggesting that the observed events unlikely arise from indirect effects associated with rapid protein turnover.

### Validation of Targeted Degradation of Identified Proteins

We recognize that changes in protein abundance upon AgnoTAC treatment may not necessarily be reflective of expected TPD mechanisms. We first determined half-maximal degradation concentration (DC_50_) ranges of 9.9–28.5 μM and maximal degradation (*D*_max_) ranges of 51–78% for a select set of AgnoTAC-target pairs, including: **1c**-UFD1, **2d**-PLOD2, 7b*-CHCHD2, and **10d**-BRD2 (Figure 2). These targets were prioritized as they represent members from multiple functional classes (e.g., enzymes, transcription factors and adaptor proteins) and they possess high quality commercially available antibodies. We next sought to assess whether targets were being downregulated through a Cullin Ring ligase (CRL)-dependent mechanism.^47^ MDA-MB-231 cells were treated with increasing concentrations of AgnoTACs, MG132 proteasome inhibitor (10 μM), or MLN4924 neddylation inhibitor (1 μM) to inhibit CRL activity,^48,49^ and protein abundance was monitored for a subset of identified downregulated targets. Co-incubation of **1c** and MG132 or **1c** and MLN4924 resulted in marked rescue of the ubiquitin recognition factor UFD1, an adaptor protein involved the ubiquitin-dependent proteolytic pathway,^50^ suggesting that **1c** promotes proteasomal degradation of UFD1 via the action of a CRL in a dose-dependent fashion (Figure 2a–c). We observed similar results upon cell treatment with AgnoTACs **2d**, 7b* and **10d** degrading the PLOD2 lysyl hydroxylase^51^ (Figure 2d–f), Parkinson’s disease associated transcription factor CHCHD2 ^52^ (Figure 2g–i), and the chromatin reader protein BRD2 ^53^ (Figure 2j–l) respectively. We observed that for a subset of targets, such as CHCHD2, protein levels were influenced by MG132 treatment alone, potentially convoluting interpretation of rescue experiments. In such instances, we relied on protein rescue through neddylation inhibition, which did not affect protein levels, to confirm degradation mechanism. To further confirm that observed degradation effects for these targets were specific to the bifunctional molecule and not induced by the AgnoTAC TBM or thalidomide alone, we synthesized capped, linkerless analogs and dosed cells with each ligand alone at matched concentrations. Immunoblot analysis revealed no change in protein levels with either ligand treatment, indicating that neither the TBM nor thalidomide individually drives observed degradation (Figure S10). These results support that degradation is AgnoTAC-dependent and requires the combined bifunctional scaffold.

**Figure 2:**
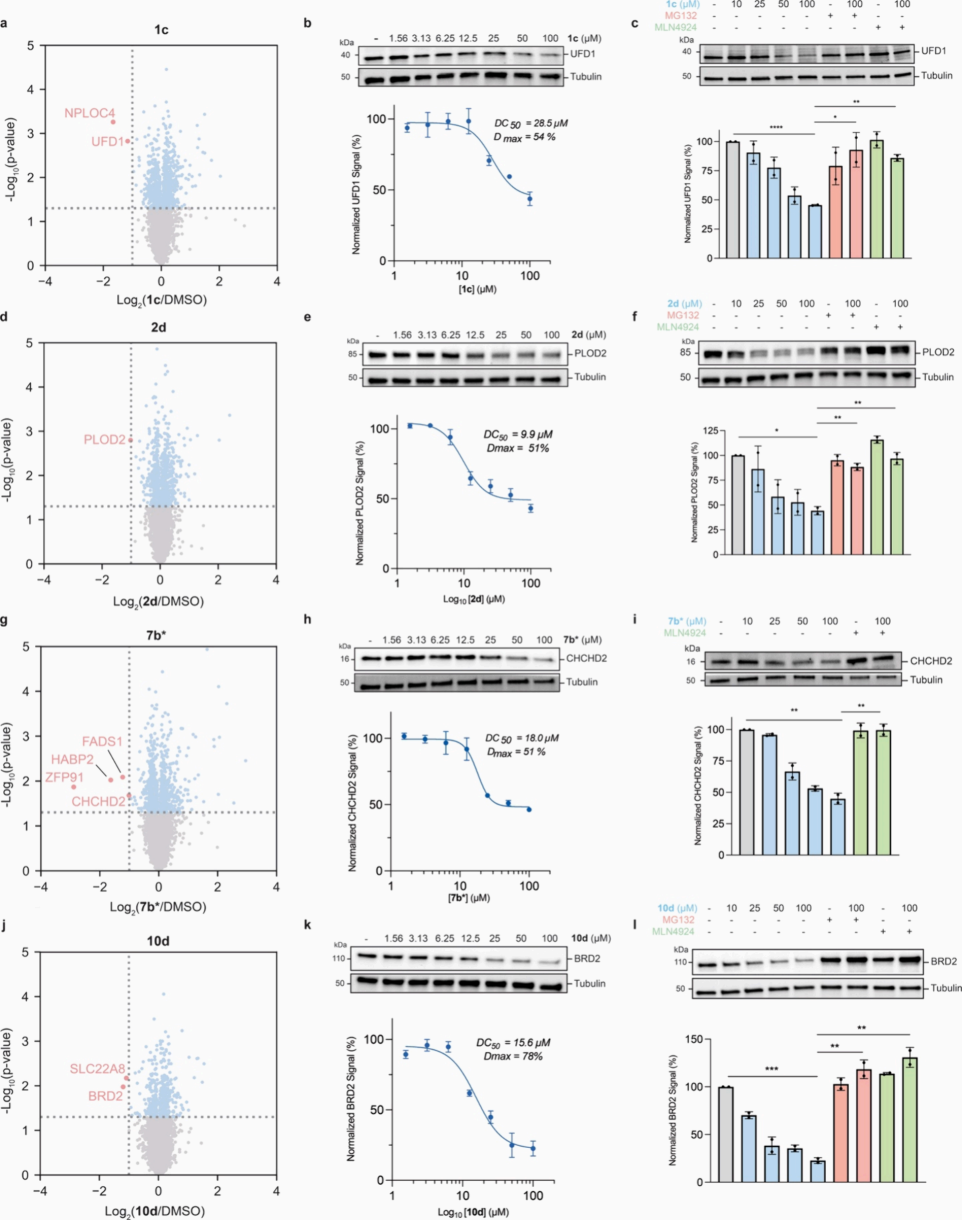
**Targets degraded by AgnoTACs.** Representative validation of select targets. MDA-MB-231 cells were treated with various AgnoTACs for 18 h, including (a–c) **1c**, (d–f) **2d**, (g–i) 7c*, and (j–l) **10d**. Scatter plots depict relative fold change in protein abundance following treatment of MDA-MB-231 cells with probes. The *x* axis represents log_2_(fold change) in protein expression. The *y* axis represents statistical significance (*p* value). Downregulated proteins are highlighted in red. Dashed lines represent thresholds (fold change > 2, *p* < 0.05). Data represents *n* = 2 biologically independent experiments. Associated proteomics data are provided in Supplemental Dataset 1. Validation by immunoblot analysis was performed by preincubating cells with MG132 (10 μM) or MLN4924 (1 μM) for 2 h, and subsequently coincubating with probes for 18 h.

In addition to high-confidence targets, we also noted proteins that fell outside our strict threshold cut-offs but nonetheless showed consistent trends of decreased abundance. For example, sirtuin 1 deacetylase (SIRT1), a regulator of diverse cellular processes including transcription, stress response, metabolism, and aging,^54^ was downregulated upon treatment with **5a** (1.96-fold, *p* < 0.05) but did not meet our strict threshold (Figure S11a,b). Follow-up experiments confirmed that SIRT1 degradation occurs in a dose-dependent and proteasome-dependent manner (Figure S11c,d). This example demonstrates that while our analysis focuses on statistically robust targets, additional biologically relevant degraders may emerge through targeted follow-up of trends observed in the proteomic data. Collectively, this data supports that AgnoTACs promote dose-dependent CRL-mediated proteasomal degradation for a wide variety of proteins.

In addition to the expected canonical mechanism described above, we also observed intriguing instances where AgnoTACs induced proteasomal degradation of targets independently of CRL activity. While CRBN is typically associated with Cullin-mediated ubiquitination, these findings suggest the existence of alternative degradation pathways mediated by AgnoTACs. For example, degradation of the oncogenic transcription factor ETS-1 ^55^ by **5a** (Figure 3a–c) and the functionally uncharacterized transmembrane protein TMEM205 ^56^ by 8d* (Figure 3d–f) were dependent upon proteasomal activity but were surprisingly unaffected by cotreatment with MLN4924. To confirm that AgnoTACs are engaging such targets in cells, we synthesized a “fully functionalized” (FF) probe of the F08 headgroup (**FFF-8**) containing a photoreactive diazirine group and an alkyne tag. We observed dose-dependent labeling of endogenous TMEM205 by **FFF-8** (Figure 3g), which is blocked when coincubated (over a short duration, ∼30 min) by excess 8d*, confirming target engagement (Figure 3h). Finally, we show that TMEM205 degradation is unaffected in cells where CRBN is depleted via VHL-based CRBN degrader CRBN-6,5,5,VHL^57^ (Figure 3i). We therefore speculate that such degradation events could result from protein destabilization upon AgnoTAC binding or alternative yet-to-be-characterized mechanisms, independent of CRBN. Nonetheless, these findings emphasize the need for thorough validation experiments to fully account for distinct mechanisms driving small molecule-mediated protein degradation.

**Figure 3:**
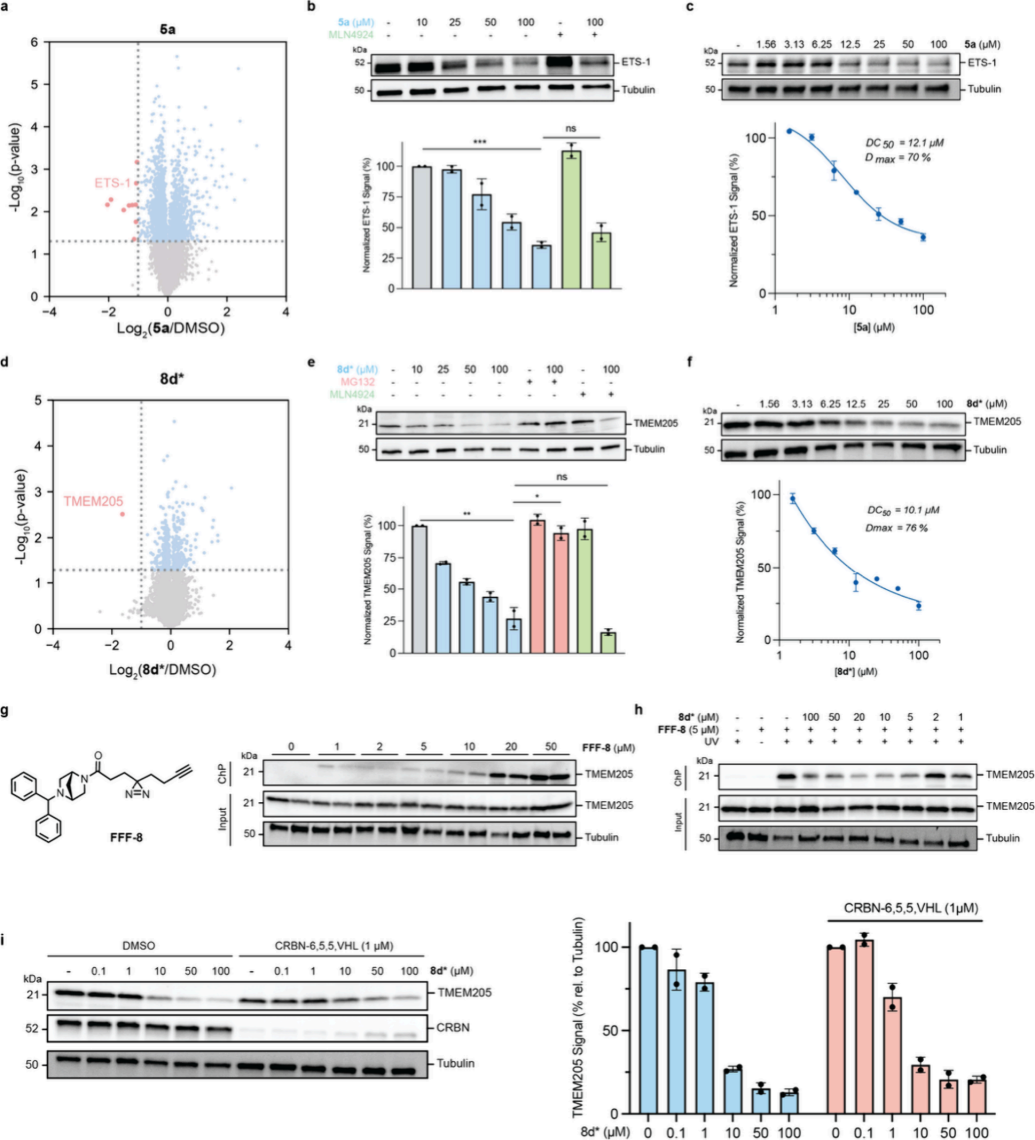
**Noncanonical AgnoTAC-induced degradation events.** (a) Volcano plot depicting global protein abundance changes upon **5a** treatment of MDA-MB-231 cells for 18 h. Vertical dashed line represents a fold change (FC) threshold of 2, while horizontal dashed lines denote a *p*-value threshold of 0.05. Statistical significance was assessed using a two-tailed Student’s *t* test. Red dots indicate targets that meet both significance thresholds. (b–f) Dose-dependent proteasome-mediated and neddylation independent degradation of (b, c) ETS-1 transcription factor by **5a**, and (d–f) TMEM205 transmembrane protein by 8d*. For rescue experiments, MDA-MB-231 cells are pretreated with MG132 (10 μM) or MLN4924 (1 μM) and subsequently coincubated with probes for 18 h. Data are represented as mean ± SD (*n* = 2 biological replicates). Statistical significance was determined using a two-tailed Student’s *t* test (*, *p* < 0.05; **, *p* < 0.01; ***, *p* < 0.001; ns = not significant). (g) Dose-dependent TMEM205 engagement by **FFF-8**. (h) Competition of endogenous TMEM205 binding to **FFF-8** by AgnoTAC 8d*. Results are representative of *n* = 2 biological replicates. (i) TMEM205 levels following CRBN-induced degradation (mean ± SD, *n* = 2 independent experiments).

As noted above, **2a** significantly impacted cell viability and our first pass proteomics profiling revealed a substantial number of proteins with altered abundance (Figure 4a). Interestingly, most downregulated targets were mitochondrial proteins and components of the oxidative phosphorylation (OXPHOS)^58^ pathway (Figures 4b and S12). Notably, coincubation with MLN4924 substantially rescued **2a**-induced cytotoxicity (Figure 4c), suggesting toxicity may be partly driven by an AgnoTAC-mediated degradation event. To investigate further, we monitored proteome-wide changes over 2, 4, 6, and 12 h, revealing mitochondrial carrier homologue 2 (MTCH2) protein, an outer mitochondrial membrane insertase^59^ as the protein first affected by compound **2a**. MTCH2 was the only downregulated protein detected within the first 2 h of treatment and continued to be significantly reduced at all subsequent time points (4–6 fold relative to DMSO, Figure 4d). Rescue experiments confirmed that MTCH2 degradation occurred dose-dependently and via proteasome and neddylation-mediated pathways (Figure 4e). Finally, to assess whether MTCH2 is a direct target of **2a**, we generated a photoaffinity analog of the **2a** TBM (**FFF-2**) and confirmed dose-dependent labeling of MTCH2 in cells (Figure 4f). Notably, this labeling is robustly blocked in cells coincubated with **FFF-2** and **2a**, consistent with **2a** directly binding MTCH2. Recent reports have implicated MTCH2 in maintaining mitochondrial homeostasis and regulating apoptosis,^60,61^ suggesting that its degradation by **2a** may drive the observed cytotoxic effects. However, further investigation is needed to clarify whether MTCH2 degradation is the primary mechanism of action or if other mitochondrial disturbances also contribute to **2a**’s cytotoxicity.

**Figure 4:**
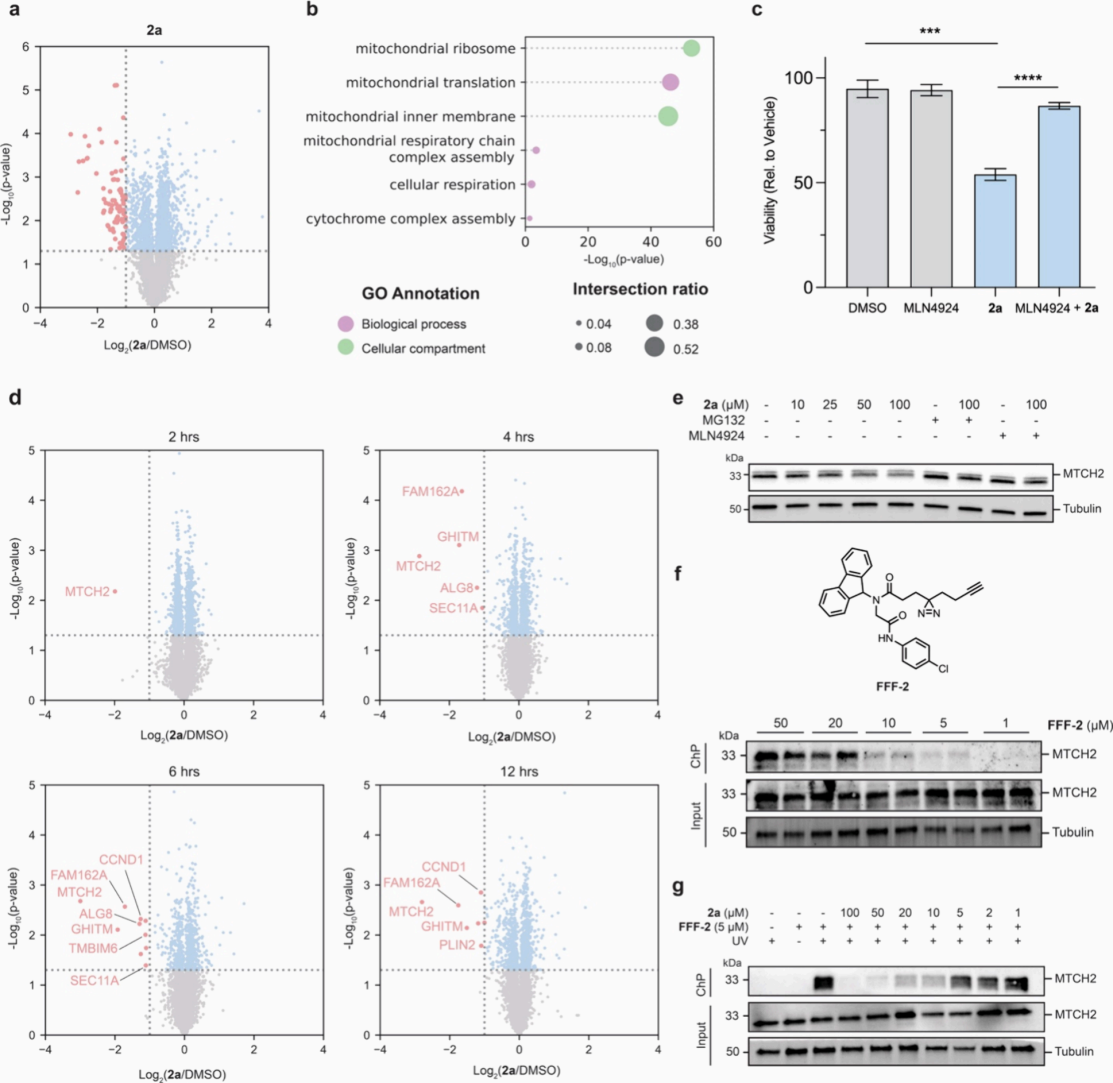
**Characterization of 2a proteomic effects.** (a) Volcano plot of protein abundances following 18 h treatment of MDA-MB-231 cells with **2a**. (b) Top Gene Ontology (GO) driver terms categorized by biological process and cellular compartments. The adjusted *p* value is calculated with Fisher’s exact test. Intersection ratio represents proportion of targets within a given category relative to the total number of targets in the query. (c) Cell viability assay of MDA-MB-231 cells treated for 18 h with DMSO (vehicle), MLN4924 (1 μM), **2a** (50 μM) or a combination of MLN4924 and **2a**. Results depict the relative luminescence percentage, normalized to vehicle. Error bars represent mean ± SD from *n* = 3 independent experiments. Statistical significance was determined using a two-tailed Student’s *t* test (*, *p* < 0.05; ***, *p* < 0.001; ****, *p* < 0.0001). (d) Volcano plots of protein abundances at 2, 4, 6, and 12 h following **2a** treatment. Downregulated targets are highlighted in red. (e) Immunoblot confirming proteasom**e**- and CRBN-mediated degradation of MTCH2 following 2 h preincubation with MG132 (10 μM) or MLN4924 (1 μM), and subsequent 2 h cotreatment with **2a**. (f) Immunoblot of dose-dependent MTCH2 engagement by fully functionalized fragment headgroup F02 (**FFF-2**). (g) Immunoblot of endogenous MTCH2 engagement by **FFF-2** (5 μM) competed by **2a**. Results are representative of *n* = 2 independent biological replicates. ChP indicates chemoprecipitation.

### Impact of AgnoTAC Modifications on Target Degradation

It is well-established that changes to linker length and composition can impact the potency and selectivity of bifunctional molecules.^62–64^ We therefore wondered how variations in linker design affect the proteome-wide degradation profiles across all scaffolds. Analysis of our chemical proteomic data revealed that each linker series within a scaffold generally downregulated the same number of targets (∼25), with the exception of PEG2, which had 12 targets (Figures 5a and S13). Moreover, unique targets across all scaffolds were overwhelmingly downregulated by a single linker type (Figure 5b), indicating that linker structure plays a critical role in AgnoTAC selectivity. For example, BRD2 degradation is primarily observed with PEG linker-containing AgnoTACs based on scaffold F10, whereas aliphatic linkers had minimal effect on BRD2 abundance (Figures 5c,d and S14a,b). We further evaluated BRD2 depletion using a HiBiT assay in the HEK293T BRD2-HiBiT cell line (Figure S14c), which confirmed that **10d** most potently degraded BRD2, as observed in our proteomic studies. This observation was notably carried over to other BET family proteins (e.g., BRD3 and BRD4, Figure S14d,e). Additionally, in some cases, degradation is preferentially mediated by a single linker type. For instance, UFD1 and NPLOC4 (nuclear protein localization 4, NPL4) are both degraded equipotently by **1c** (Figures 5e–g and S15a). Additional examples of linker-dependent degradation profiles include PLOD2 (Figure S15b–d), TMEM205 (Figure S15e-g,k), and ETS-1 (Figure S15h–j,l).

**Figure 5:**
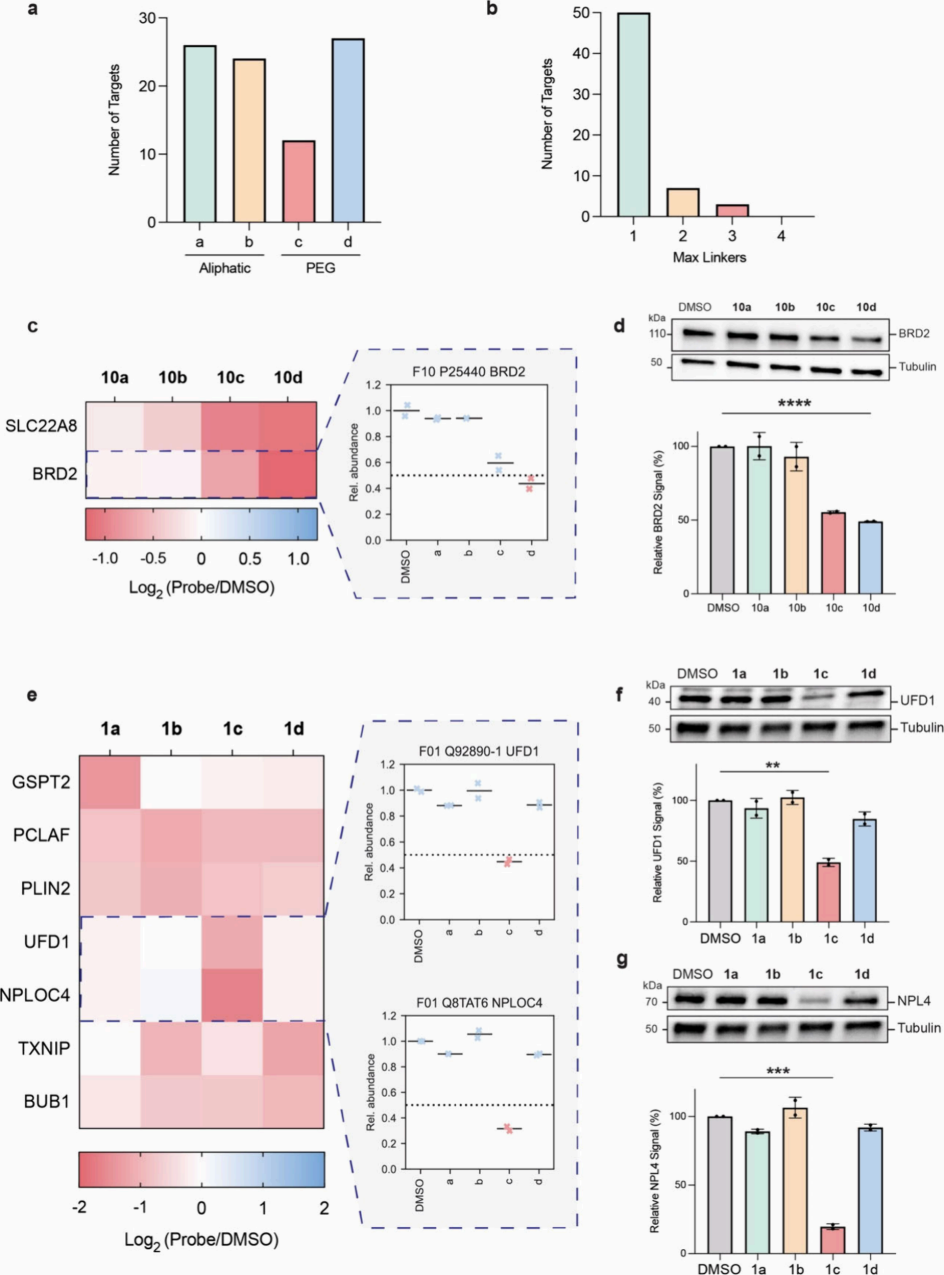
**Linker composition influences degradation profiles.** (a) Total number of targets downregulated classified by linker type (aliphatic or PEG). (b) Number of unique downregulated proteins targeted by 1, 2, 3, or all 4 linker types across scaffolds. (c, d) Protein abundance heatmaps and quantitative analysis of the mean abundance of BRD2 across different linker types, in response to 50 μM treatments. (e, g) Protein abundance heatmaps and quantitative analysis of the mean abundance of UFD1/NPL4 in response to F01 AgnoTAC treatments. Dashed line represents downregulation threshold. Bar plots represent mean ± SD (*n* = 2 biological replicates). Statistical significance was determined using a two-tailed Student’s *t* test (**, *p* < 0.01; ***, *p* < 0.001; ****, *p* < 0.0001).

Previous studies have shown that thalidomide-based PROTACs can induce degradation of immunomodulatory (IMiD) targets through molecular glue degrader (MGD) mechanisms.^65–67^ Structural studies of CRBN ternary complexes with IMiDs have revealed a recurring motif among various neosubstrates, including CK1α, GSPT1 and IKFZ proteins.^68,69^ This motif is characterized by a β-hairpin loop containing a key glycine (G) residue, facilitating interactions with the CRBN/MGD binding interface.^29,70^ Given the prevalence of β-hairpin G-loops in the human proteome,^71^ we wondered whether AgnoTAC targets possess this motif. Using a known G-loop structure as a query, we identified nine proteins among AgnoTAC targets containing at least one β-hairpin loop (Figure S16a), raising the possibility of being potential IMiD off-targets. Among 72 AgnoTAC profiles, we identified 17 that degrade established IMiD glue-based off-targets, including GSPT1, GSPT2 and ZFP91 (Figures S6a and S16b). Notably, these degradation events occurred with both aryl ether and oxyacetamide linkages used in our studies, though IMiD off-targets were often not shared across all headgroups with the same linkages (Figure S16c,d). We noted even subtle structural modifications, such as altering the connectivity of the bridging C–C bond in the benzhydrylpiperazine group between 7b* and 8b* (C5 linker) resulted in distinct effects on GSPT1 levels, with 8b* causing more pronounced downregulation (Figure S16e–h).

Together, these findings underscore the significant influence of linker design on target degradation patterns as well as highlights the challenges in extrapolating generalizable linker design principles when examining a broad range of targets.

### Mechanistic Investigation of Degraded Targets

Though we have demonstrated, in several examples, that AgnoTAC-dependent protein degradation is mediated through Cullin and proteasome pathways, we sought to further characterize these degradation events. To this end, we conducted a series of experiments to further delineate the mechanism of **10d**-induced BRD2 proteasomal degradation (Figure 2j–l). We first assessed degradation kinetics, where we observed BRD2 degradation occurs as early as 2 h following **10d** treatment (Figure S17a). We next assessed E3 ligase-binding dependence by synthesizing a structurally similar negative control **10d-neg** in which the glutarimide moiety of the thalidomide derivative is capped with a methyl group (Figure 6a), preventing CRBN recruitment.^72^ As expected, **10d-neg** failed to induce BRD2 degradation, confirming that E3 ligase engagement is needed for **10d**’s activity (Figure 6b). We further confirmed that cotreatment of **10d** and JQ1, a bromodomain-targeting inhibitor,^73^ rescued BRD2 levels, suggesting that **10d** binds BRD2 competitively with JQ1 and coincubation disrupts ternary complex formation to prevent degradation (Figure 6c,d). We generated analogous negative control AgnoTACs for several additional targets displaying Cullin-dependent degradation, including CHCHD2 (Figure S17b,c), SIRT1 (Figure S17d,e) and PLOD2 (Figure 6e,f), confirming degradation dependence on CRBN. Perhaps not surprising, for targets undergoing Cullin-independent degradation, both the parent as well as the methyl-capped thalidomide control displayed similar profiles, confirming, in those instances, that degradation occurs via alternative mechanisms (Figure S17f–k).

**Figure 6:**
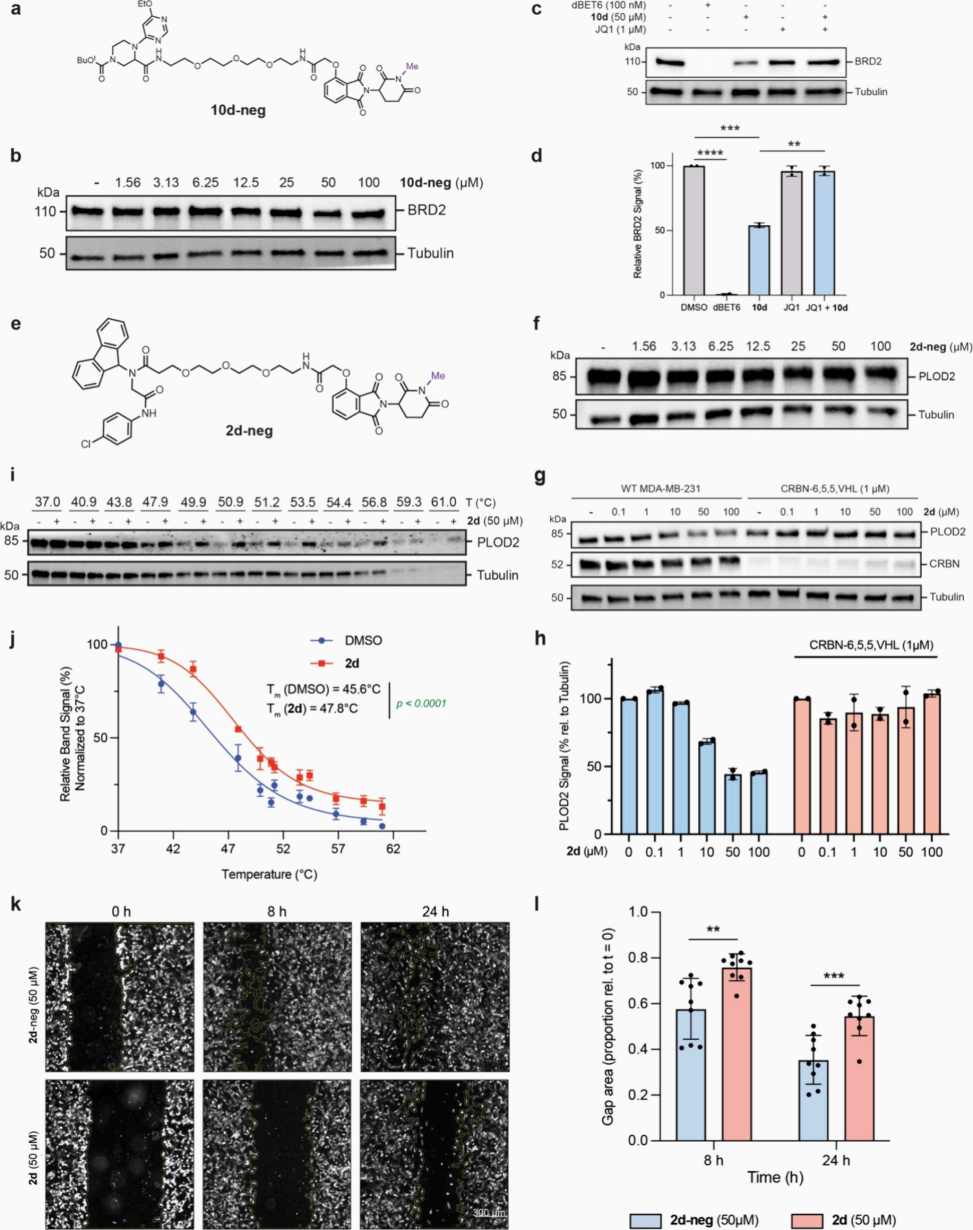
**Mechanistic characterization of AgnoTAC-induced degradation events.** (a) Chemical structure of negative control **10d-neg** and (b) immunoblot analysis of dose–response at indicated concentrations following 18 h treatment of MDA-MB-231 cells. (c, d) Immunoblot and quantitative analysis of BRD2 degradation after 18 h treatment with dBET6 (100 nM), JQ1 (1 μM) and **10d** (50 μM). JQ1-treated cells were pretreated with inhibitor for 1 h. Bar plot data represents mean ± SD (*n* = 2 biological replicates), statistical significance was determined using a two-tailed Student’s *t* test (**, *p* < 0.01; ***, *p* < 0.001; ****, *p* < 0.0001). (e) Chemical structure of negativ**e** control **2d-neg** and (f) immunoblot analysis of PLOD2 with **2d** dose–response at indicated concentrations following 18 h treatment. (g, h) Immunoblot analysis and quantification of PLOD2 levels upon CRBN-6,5,5,VHL-induced CRBN depletion. Data points represent mean ± SD, *n* = 2 biological replicates. (i, j) CETSA for PLOD2 upon MDA-MB-231 treatment with **2d** for 30 min. Protein levels were assessed by immunoblot and quantified to generate thermal stabilization curves. Data points represent mean ± SD, *n* = 2 independent experiments. Statistical significance is indicated with *p* values. (k) Representative images of **2d** and **2d-neg** effects on MDA-MB-231 migration measured by a wound healing assay and (l) corresponding statistical significance analysis for *n* = 9 biological replicates.

Among the validated targets, we observed the degradation for PLOD2, mediated by **2d** (DC_50_ = 9.9 μM and *D*_max_ = 51%, Figure 2d–f) to be among the most potent. Depletion of PLOD2 was observable as early as 6 h and continued until 24 h after treatment (Figure S17l). In addition, we observe no PLOD2 degradation in cells treated with the glutarimide control (**2d-neg**, Figure 6e,f) or in cells where CRBN is depleted via CRBN degrader (Figure 6g,h), confirming CRBN dependence. To further verify that PLOD2 degradation was mediated by binding, we performed a cellular thermal shift assay (CETSA), where we observed marked stabilization of PLOD2 upon treatment with **2d** (Figure 6i,j) over an applied temperature gradient. PLOD2 catalyzes lysyl hydroxylation on the Gly-X-Y motif of collagen peptides and is required for the formation of stabilized collagen cross-links.^74^ Given these functions, it is perhaps not surprising that PLOD2 is critical for epithelial mesenchymal transition (EMT), migration, and metastasis of various cancers,^75,76^ as well as tissue repair and fibrosis,^77^ though no selective inhibitors have been reported. To assess whether targeted degradation of PLOD2 would disrupt cellular migration, we treated MDA-MB-231 cells with **2d** and observed a substantial decrease in migration rates relative to **2d-neg**-treated cells (Figure 6k,l). Taken together, these data suggest that AgnoTACs directly engage and degrade targets through a CRBN-mediated mechanism and can serve as chemical probes to investigate protein functions.

As noted above, AgnoTAC **1c** induces linker-dependent, selective and equipotent downregulation of UFD1 and NPL4 (Figures 2a and 5h–j). Ubiquitin fusion degradation 1 (UFD1) and nuclear protein localization 4 (NPL4) are adaptor proteins that complex with AAA ATPase p97 (also known as VCP), mediating various p97 functions in protein ubiquitination and degradation,^50,78,79^ endoplasmic reticulum-associated degradation (ERAD),^80–82^ and transport processes.^83,84^ Previous studies demonstrated that genetic ablation of either UFD1 or NPL4 destabilizes the VCP-UFD1-NPL4 complex, leading to proteasomal degradation of the other component, releasing free VCP.^85^ Interestingly, we observe no changes in VCP abundance (Supplemental Dataset 1 and Figure S18), prompting us to speculate that AgnoTAC **1c** either directly targets UFD1 and NPL4 for degradation, or contributes to the destabilization of this complex resulting in subsequent degradation. As noted previously, we found UFD1 to be degraded by **1c** via a CRBN-mediated pathway (Figure 2c). Though degradation of NPL4 by **1c** appeared to progress equipotently (Figure 7a,b) and with the same kinetics as UFD1 (Figure, S17m), its levels were restored upon proteasome inhibition, but not by neddylation inhibition (Figure 7c,d). Notably, UFD1 and NPL4 degradation was recapitulated across multiple human cell lines derived from diverse tissues (Figure S19), suggesting that these effects are not restricted to a specific model system. In addition, UFD1 and NPL4 levels were unaffected in cells incubated with negative control analog **1c-neg (** Figure 7e,f) or depleted of CRBN (Figure 7g–i) implying that the degradation of both proteins is dependent on CRBN and is likely not the result of **1c** binding alone. To further assess whether UFD1 or NPL4 is the direct binding target of **1c**, we conducted CETSA at shorter incubation periods where protein loss was not observed (Figure 7j). Treatment of MDA-MB-231 cells with **1c** resulted in increased thermal stability for UFD1 (Figure 7k), whereas no stabilization was observed for NPL4 (Figure 7l), suggesting direct engagement of UFD1 by **1c**. Collectively these results indicate that **1c** likely binds UFD1, resulting in its CRBN-mediated proteasomal degradation, and that this binding and recruitment of CRBN leads to subsequent proteasomal degradation of NPL4 via endogenous pathways.

**Figure 7:**
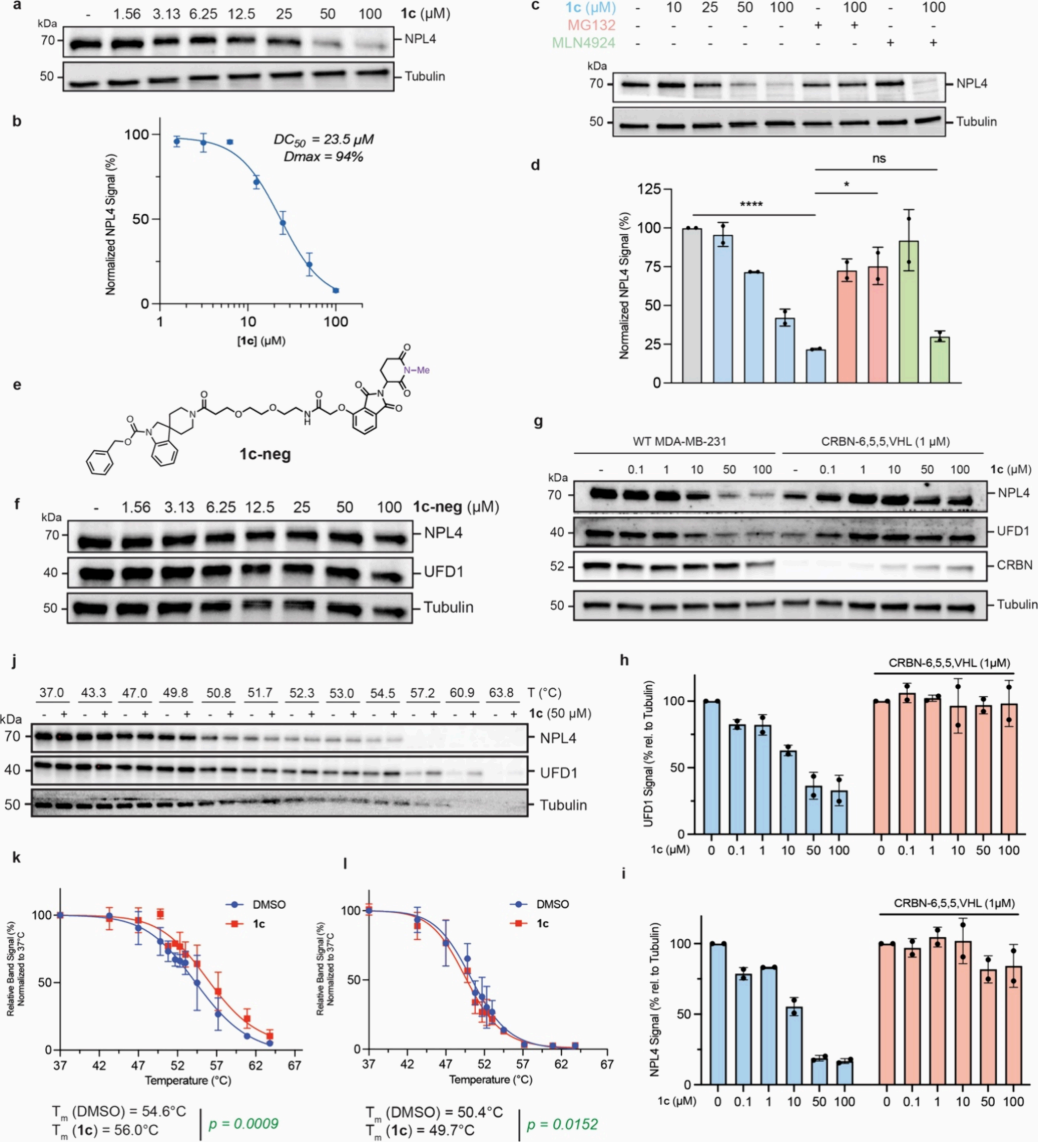
**UFD1/NPL4 adaptor protein complex degradation mechanism.** (a–d) Immunoblot analyses of NPL4 in MDA-MB-231 cells treated with various concentrations of **1c**, MG132 (10 μM), or MLN4924 (1 μM). Bar graph displays normalized NPL4 signal intensities, highlighting significant reductions and protein level rescues upon **1c** treatment (mean ± SD, *n* = 2 independent experiments; *, *p* < 0.05; ****, *p* < 0.0001; ns, not significant). (e) Chemical structure of **1c-neg** control, followed by (f) immunoblot analysis of NPL4 and UFD1 levels after **1c-neg** treatment at various concentrations. (g) Immunoblot analysis and (h, i) quantification of UFD1 and NPL4 levels upon CRBN-6,5,5,VHL-induced CRBN depletion (mean ± SD, *n* = 2 independent experiments). (j) CETSA for NPL4 and UFD1 upon **1c** treatment for 30 min. Protein levels were assessed by immunoblot. (k, l) Thermal stabilization curves for (k) UFD1 and (l) NPL4 in the presence of **1c** compared to DMSO control. Data points represent mean ± SD, *n* = 2 independent experiments. Statistical significance is indicated with *p* values.

## Conclusion

Chemical proteomics is firmly established as a powerful strategy for global ligand and target discovery in native systems. Founded on the use of active site-directed probes as reporters for enzyme function and pocket occupancy (i.e., ABPP),^86–90^ these methods are now routinely deployed to map covalent and noncovalent small molecule interactions proteome-wide, independent of protein function or class.^91^ Despite numerous instances of chemical proteomic methods being utilized to develop functional small molecules against diverse targets,^92–101^ conventionally such methods report binding, rather than functional outcomes. More generally, understanding how small molecule binding might relate to downstream consequences, and how binders can be converted into functional compounds, is often target-specific. To begin addressing this gap, recent efforts have aimed to read out proteome-wide functional changes, such as perturbations to protein abundance^36^ and complex formation,^102^ in response to small molecule treatment. However, in these examples, functionally unbiased chemical libraries are utilized and necessitate substantial follow-up investigation to pinpoint the mechanism of observed functional outcomes. Considering these challenges, we sought a chemical proteomic strategy for the systematic and global discovery of small molecules that impose specific functional consequences. This effort was driven primarily by the lack of such methods to discover small molecule protein degraders beyond targets with established ligands. We hypothesized that integrating function-specific library design principles with quantitative proteomics would enable prospective discovery of small molecules that are tuned to degrade proteins through a predefined mechanism.

To establish a proof-of-concept, we employed straightforward coupling chemistry to generate a library of bifunctional molecules aimed at agnostically identifying degradable proteins via CRBN-mediated proteasomal degradation. Given the library’s small size relative to conventional high-throughput screening (HTS), we prioritized structurally diverse, drug-like TBMs (Figures S1 and S2) to maximize exploration of chemical space, which we suspect contributed to the successful identification of novel degraders. While the importance of linkers in achieving productive ternary complex formation for specific targets is well-established, our findings emphasize their crucial roles in *de novo* target discovery using agnostic screens. From these efforts, we identified 50+ unique downregulation events, many of which were validated to proceed via the intended degradation mechanism. Notably, we identified, to our knowledge, first-in-class chemical probes for several proteins, including UFD1/NPL4, CHCHD2, MTCH2, and PLOD2, resulting in their targeted degradation. Finally, despite their modest potencies, the tested AgnoTACs possessed surprisingly selective degradation profiles, indicating that they could serve as suitable probes to investigate protein function, (e.g., PLOD2, Figure 6k,l).

Bifunctional molecules tend to exhibit a phenomenon wherein excessive ligand saturates individual binding partners, outcompeting ternary complex formation (i.e., the hook effect). In our studies, we observed AgnoTACs generally produced monotonic dose–response curves, with no appreciable loss of degradation at high compound concentrations across a diverse set of targets (Figures 2, 3, 7, and S11). We typically observed DC_50_ values in the 10–30 μM range, with maximal degradation levels of 40–80%, indicating that AgnoTACs likely possess lower affinities for their targets, unoptimized linkers, and/or limited permeability. Coupled to this observation, the reported affinities for the CRBN ligands used in our study are in the low micromolar range,^103,104^ and therefore the binary complexes are likely poorly populated, even at high ligand concentrations, potentially leading to the observed monotonic activity profiles.^105^ Alternatively, despite low binary affinities, ternary complexes for these targets could possess high cooperativity, which may also manifest a delayed hook effect.^106^ Of course, we cannot exclude the possibility of mechanisms that may limit intracellular accumulation of AgnoTACs. We emphasize that these factors must be evaluated on a case-by-case basis, as they are inherently dependent on the specific combination of target, recruiter, linker architecture, and cellular context.

When considering global degrader-discovery strategies, several technical aspects merit attention. First, we noted that many AgnoTACs, despite screening at relatively high concentrations, led to relatively minimal proteomic fluctuations. Most TBM ligands tested generally have low molecular weight and complexity and therefore may have limited functional proteomic interactions at these concentrations. As alluded to above, some AgnoTACs may possess limited cell permeability or be prone to efflux mechanisms resulting in insufficient intracellular accumulation. Drawing parallels to phenotypic screening of conventional small molecule libraries,^107^ we view the ability to distinguish cell-active from inactive AgnoTACs, as a strength, as it allows early stage triaging of chemotypes unsuitable for cellular studies. In addition, when employing new libraries, cell types, etc., factors such as dose and incubation time should be carefully considered. Though we generally observed more proteomic effects at higher AgnoTAC concentrations and longer incubation times (Figures S5, S7, and S8), lower screening concentrations may capture higher affinity degradation events (due to the hook effect), and shorter incubation times may prevent confounding abundance changes emanating from indirect mechanisms.

A key takeaway from our study is the importance of rigorous validation to confirm AgnoTAC-mediated degradation proceeds through the intended mechanism. Employing negative control probes, direct target engagement assays, and effector controls (e.g., CRBN depletion) is essential to differentiate genuine degradation events from indirect effects. While many events were consistent with canonical CRBN- and proteasome-dependent pathways, and direct target engagement was confirmed using a wide array of orthogonal assays, including CETSA (e.g., PLOD2, UFD1), known small-molecule binder competition (e.g., BRD2) and photoaffinity labeling experiments (e.g., MTCH2), supporting a bifunctional mode of action, we cannot rule out the possibility of alternative mechanisms in some instances. For example, in the case of UFD1/NPL4, our data suggests that UFD1 is the direct target of **1c**, while NPL4 degradation likely occurs through a bystander mechanism resulting from destabilization of the UFD1/NPL4 complex and subsequent proteasomal clearance, a pathway that, to our knowledge, has yet to be annotated. Additionally, we identified proteasomal degradation events that appeared to be CRBN-independent (e.g., ETS-1, TMEM205). In such cases, we speculate that direct binding of the AgnoTAC leads to protein destabilization, or perhaps downstream consequences of degradation events not captured in our proteomic studies. In some instances, we expect AgnoTACs to target essential proteins or disrupt key cellular processes (e.g., transcription, cell cycle), leading to broader proteomic changes and presenting additional challenges in elucidating the primary target and mechanism. This was evidenced with **2a**, which reduced cell viability and mediated downregulation of ∼85 proteins. We demonstrated that **2a** viability effects were, in part, CRBN-dependent and converged on MTCH2 as a primary degradation target. In this context, we see potential in applying AgnoTAC libraries in phenotypic screens where, akin to other chemical proteomic libraries,^93,95,108,109^ hit compounds could directly serve as tools for target identification.^110^ Finally, as noted above, the observed lack of a hook effect may reflect weak binding affinities, but we cannot rule out the possibility of CRBN-like molecular glue-like mechanisms. Accordingly, when advancing hits from such screens, additional assays characterizing ternary complex formation (e.g., pull-downs, AlphaScreen/AlphaLISA, time-resolved fluorescence energy transfer (TR-FRET)) or complementary biophysical methods^111,112^ should be considered. In this vein, we view AgnoTACs primarily as a discovery strategy for identifying proteins susceptible to small-molecule–induced degradation, with mechanistic details to be resolved on a case-by-case basis.

An additional limitation of our study is the relatively low potencies of our discovered AgnoTAC hits. We however anticipate that hits identified in this study could serve as pioneer probes that can be advanced through medicinal chemistry optimization. Such follow-up work will be essential for establishing the translational potential of these approaches. Building on the evolving concepts of chemical proteomic library design,^113–117^ future AgnoTAC libraries incorporating sp^3^-rich, stereochemically defined and densely functionalized cores should enable deeper exploration of the proteome while expediting the discovery of authentic, molecular recognition-driven degradation events. We envision systematic expansion of this approach, including broader TBM repertoires, alternative linker chemistries, and additional E3 ligase systems, as well as profiling in diverse cellular contexts will further expand the scope of degradable proteins and help define applicability across disease-relevant settings. To aide deconvolution efforts, integration of profiling methods that provide mechanistic information, for example, MS-based ubiquitinomics^30^ or pulse labeling experiments,^118^ should streamline the identification of AgnoTAC direct targets. Projecting forward, we believe that global profiling of target-agnostic, functionally defined libraries will fill gaps in small molecule-probe development by enabling the concatenated discovery of functional compounds and corresponding targets. In this context, this strategy extends TPD beyond targets with well-established ligands and we expect will furnish valuable starting points for the development of advanced small molecule degraders for biologically and therapeutically compelling proteins.

## Supplementary Material









## Notes

Figures S1–S20, Table S1, safety statement, detailed procedures for all experiments, and specifics for reagents and instruments used (PDF)
Proteomics data (XLSX)
Figure data (XLSX)
Synthetic methods and compound characterization (PDF)
