# Supplementary material for: Proteome-Wide Discovery of Degradable Proteins Using Bifunctional Molecules
Source: ACS Cent Sci. 2025 Oct 21;11(11):2240–56. doi: 10.1021/acscentsci.5c01594 (PMC12670307; doi:10.1021/acscentsci.5c01594)
Supplement: Supplementary file 1 [file oc5c01594_si_001.pdf]

Supporting Information for

Proteome-Wide Discovery of Degradable Proteins Using Bifunctional Molecules

Ines Forrest, Louis P. Conway, Clara Gathmann, Appaso M. Jadhav, Tzu-Yuan Chiu, Christian M. Chaheine, Michelle Estrada, Anurupa Shrestha, Kathy Sarris, Justin M. Reitsma, Scott E. Warder, Anil Vasudevan, Shaun M. McLoughlin, Christopher G. Parker*

*Corresponding author: Christopher G. Parker, cparker@scripps.edu

Table of content

Supplementary Figures
Figure S1 – Chemical structures of AgnoTAC degrader library
Figure S2 – Chemoinformatic analysis of the AgnoTAC library
Figure S3 – Evaluation of AgnoTAC effects on cell viability
Figure S4 – AgnoTACs do not induce stress granule formation in cells
Figure S5 – Proteomic profiles of AgnoTAC library
Figure S6 – Analysis of detected and downregulated targets
Figure S7 – Representative examples of proteomic profiles at lower AgnoTAC concentrations
Figure S8 – Representative examples of proteomic profiles at shorter time points.
Figure S9 – Analysis of short-lived proteins across four cell lines
Figure S10 – Individual ligand treatments do not affect protein levels.
Figure S11 – Additional target degraded by AgnoTAC 5a
Figure S12 – Protein cluster analysis of 2a-treated samples
Figure S13 – Linker composition influences target degradation, related to Fig. 5
Figure S14 – Effect of F10 AgnoTACs on bromodomain protein levels.
Figure S15 – Additional linker effects, related to Fig. 5.
Figure S16 – Analysis of molecular glue off-targets
Figure S17 – Additional mechanistic characterization of AgnoTAC targets
Figure S18 – AgnoTAC 1c does not induce changes in VCP protein abundance
Figure S19 – AgnoTAC 1c degrades UFD1/NPL4 in several cell lines
Figure S20 – Compiled replicates and uncropped Western blots

Supplementary Tables
Table S1 – Reagents, kits, assays, antibodies and softwares used in this study.
Supplemental Dataset 1 – Proteomics data
Supplemental Dataset 2 – Figure data

Safety Statement

Biological Methods

References

SUPPLEMENTARY FIGURES

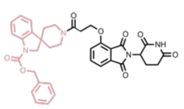

1a

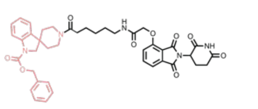

1b

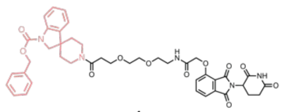

1c

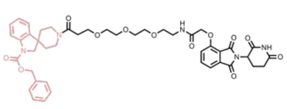

1d

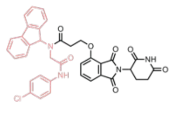

2a

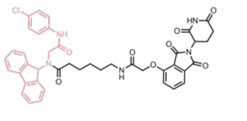

2b

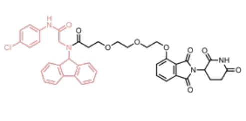

2c

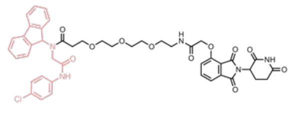

2d

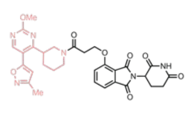

3a

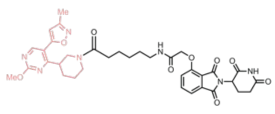

3b

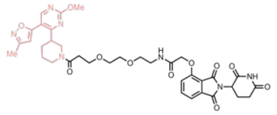

3c

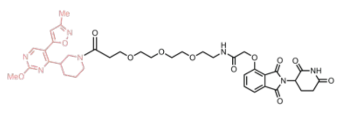

3d

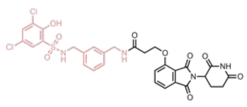

4a

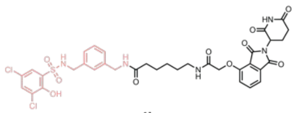

4b

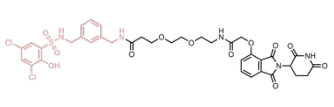

4c

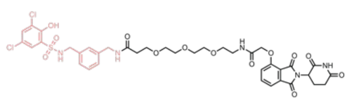

4d

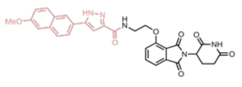

5a

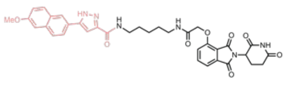

5b

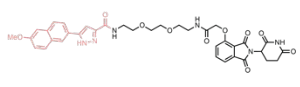

5c

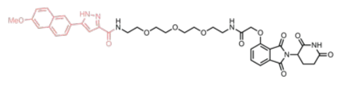

5d

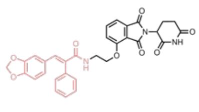

6a

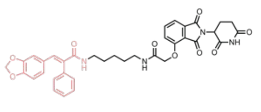

6b

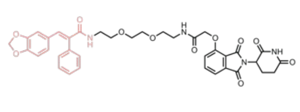

6c

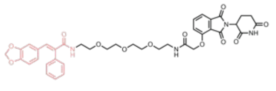

6d

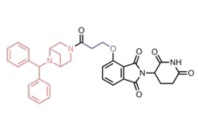

7a*

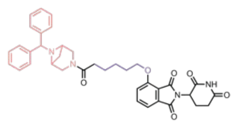

7b*

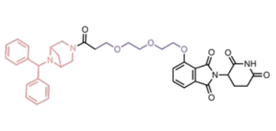

7c*

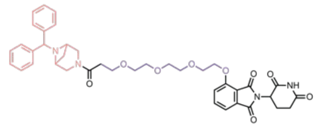

7d*

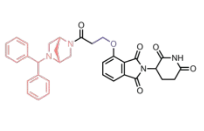

8a*

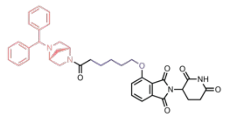

8b*

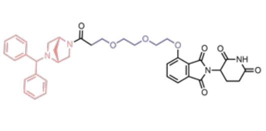

8c*

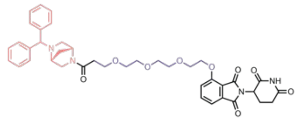

8d*

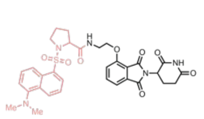

9a

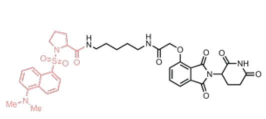

9b

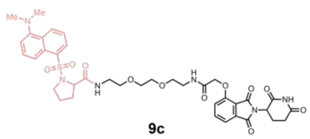

9c

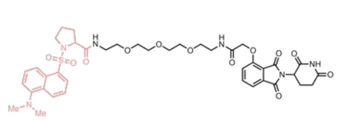

9d

Fig. S1| Chemical structures of AgnoTAC degrader library (continued on next page)

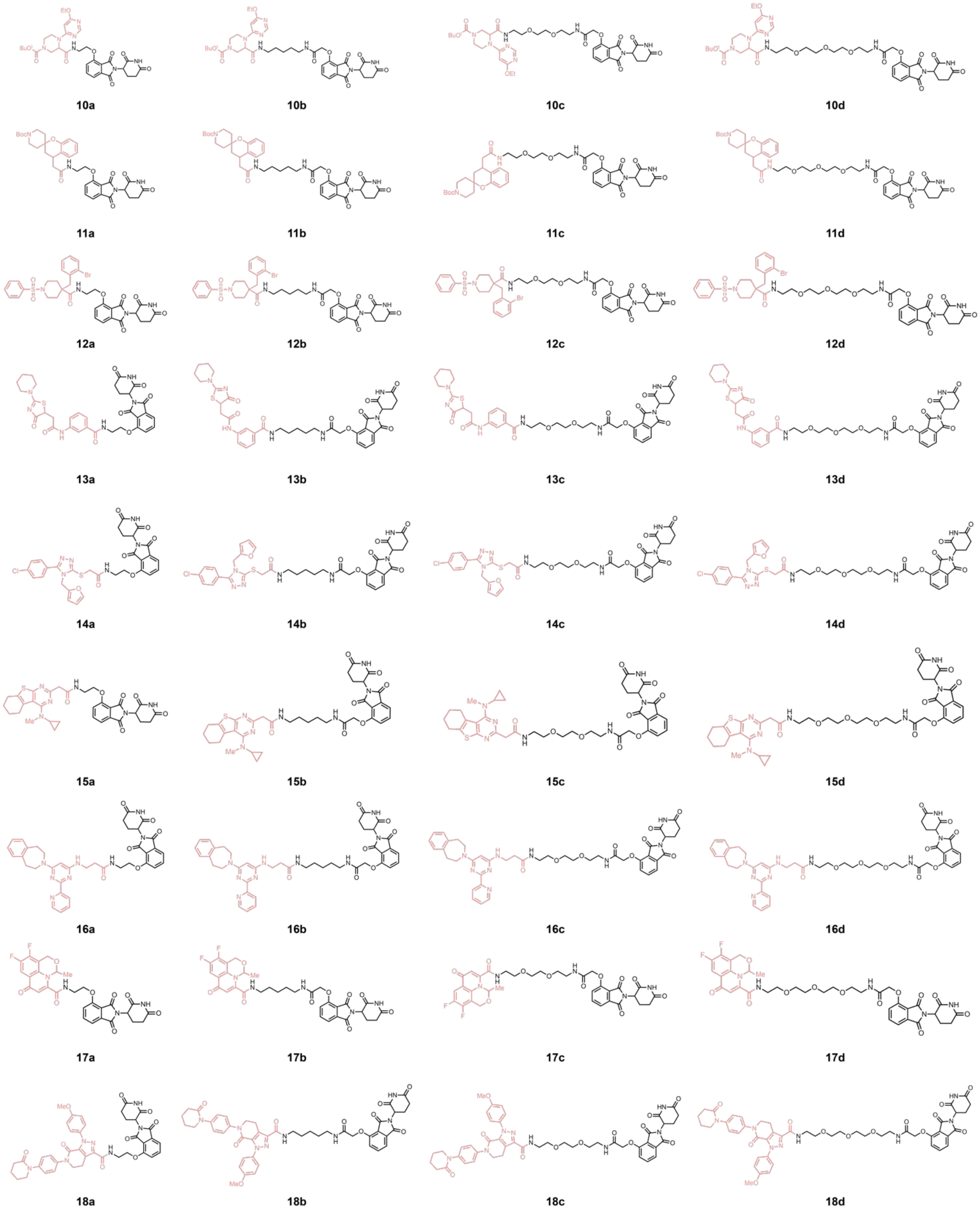

Fig. S1| Chemical structures of AgnoTAC degrader library.

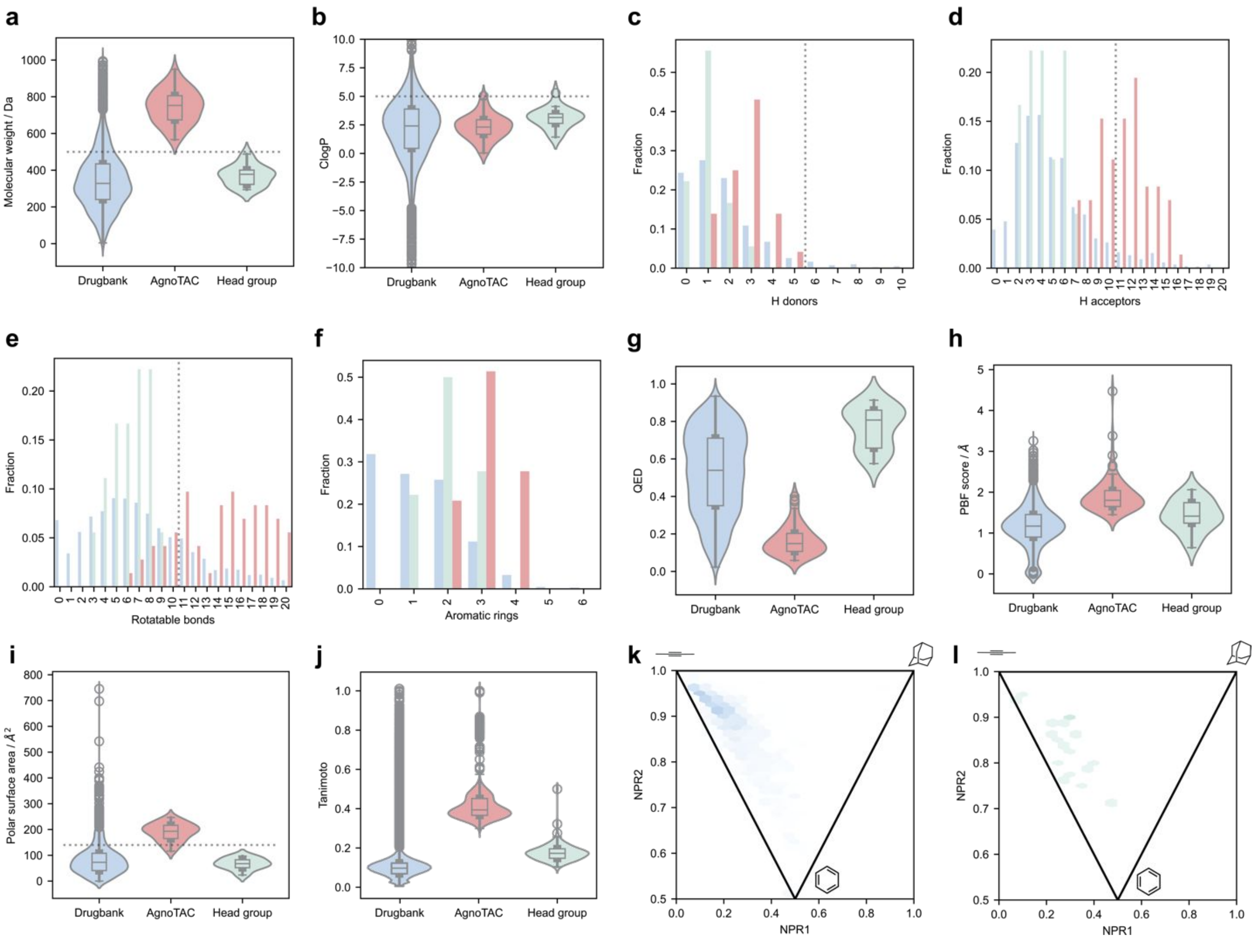

Fig. S2| Chemoinformatic analysis of AgnoTAC library. Overview of the molecular properties of AgnoTAC headgroups (green) and degraders (red) compared to DrugBank ligands (blue): (**a**) molecular weight; (**b**) calculated LogP (cLogP); (**c**) number of H bond donors and (**d**) H bond acceptors; (**e**) number of rotatable bonds; (**f**) number of aromatic rings; (**g**) quantitative estimate of drug likeness (QED); (**h**) characterization of compound sp^3 character using plane of best fit (PBF); (**i**) polar surface area distribution; (**j**) Tanimoto coefficient: similarity analysis measuring chemical diversity. (**k, l**) distributions of normalized principal moments of inertia ratios (NPR) depicting molecular shape of (**k**) DrugBank ligands compared to (**l**) AgnoTAC headgroups.

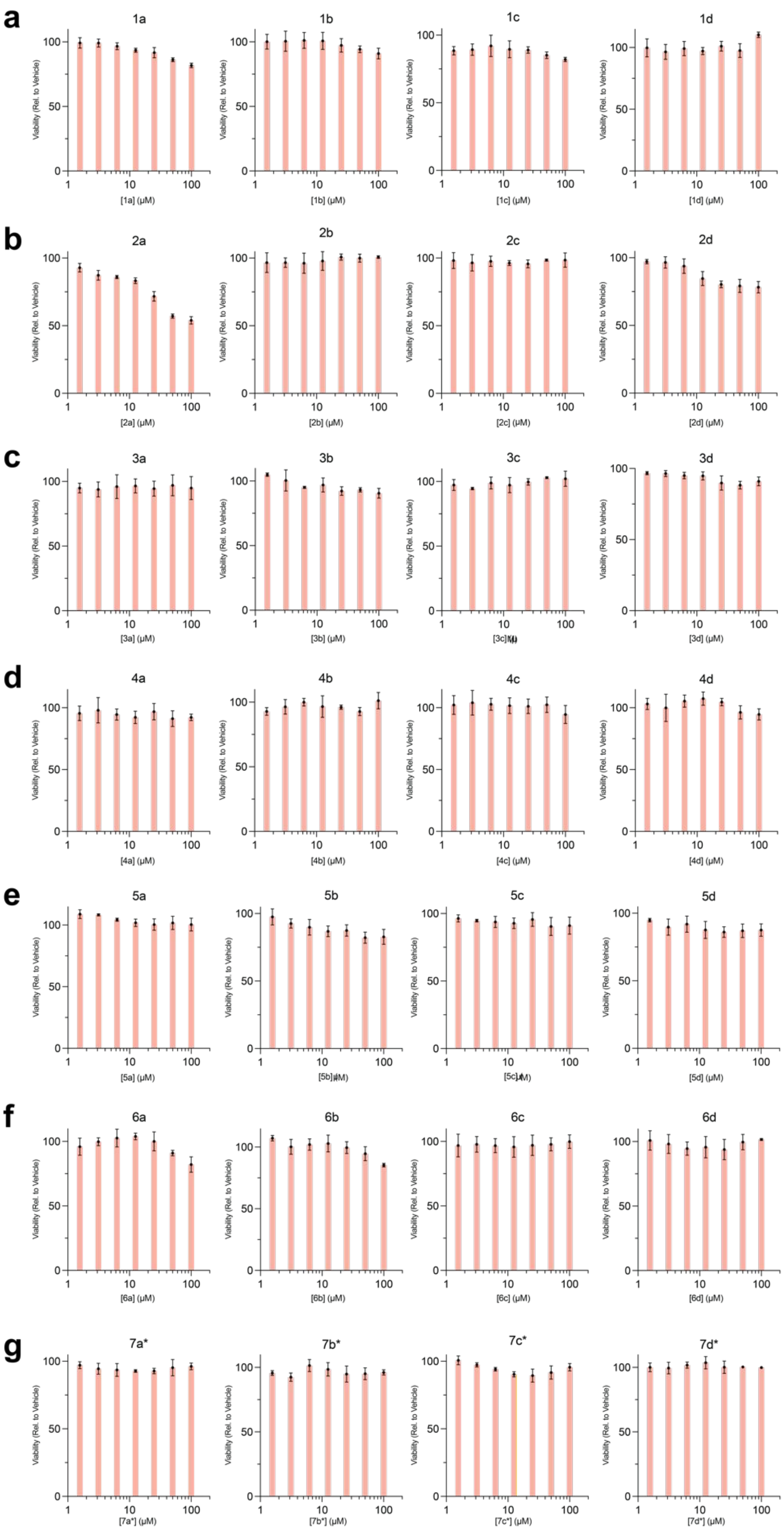

Fig. S3| Evaluation of AgnoTAC effects on cell viability (continued on next page)

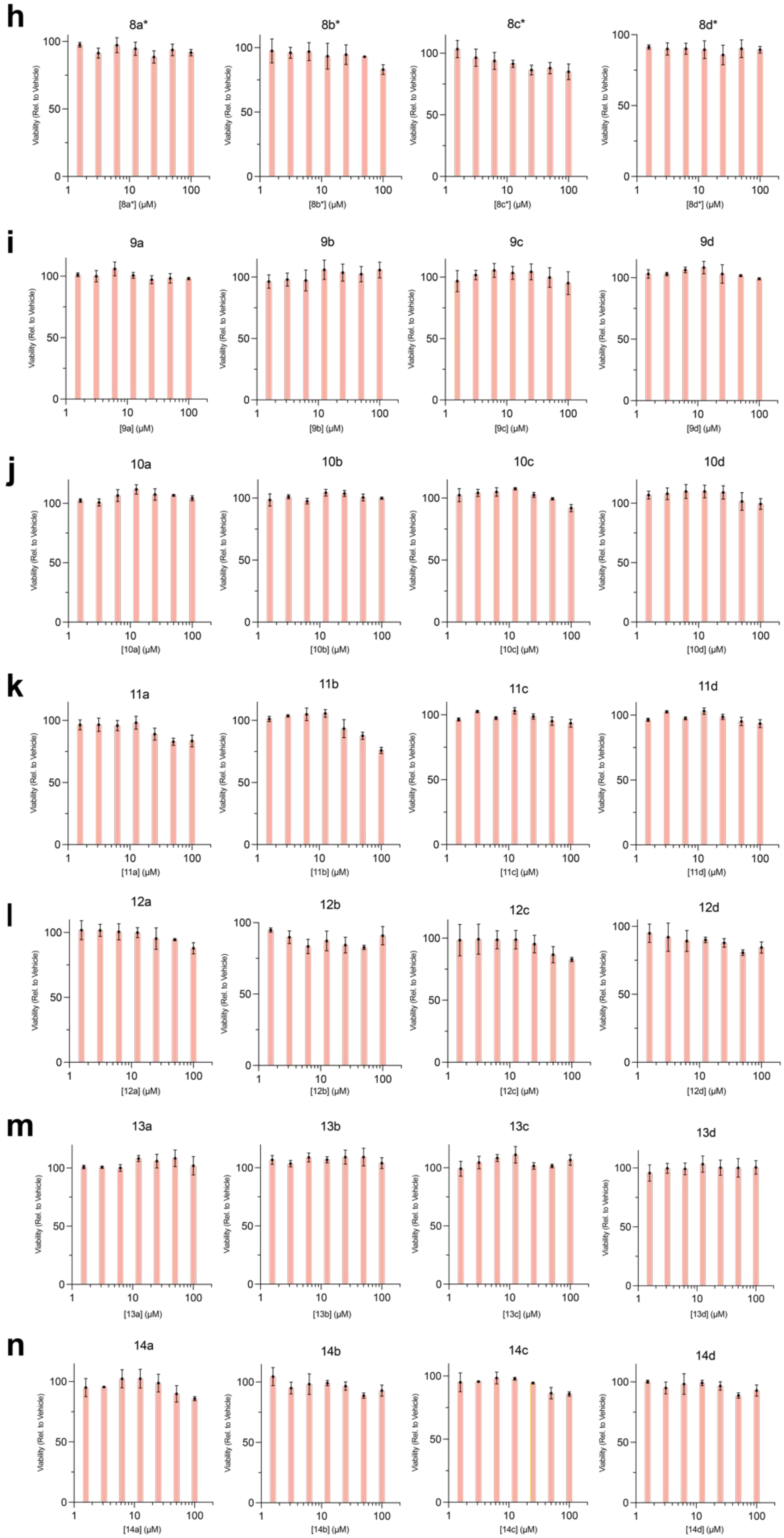

Fig. S3| Evaluation of AgnoTAC effects on cell viability **(continued on next page)**

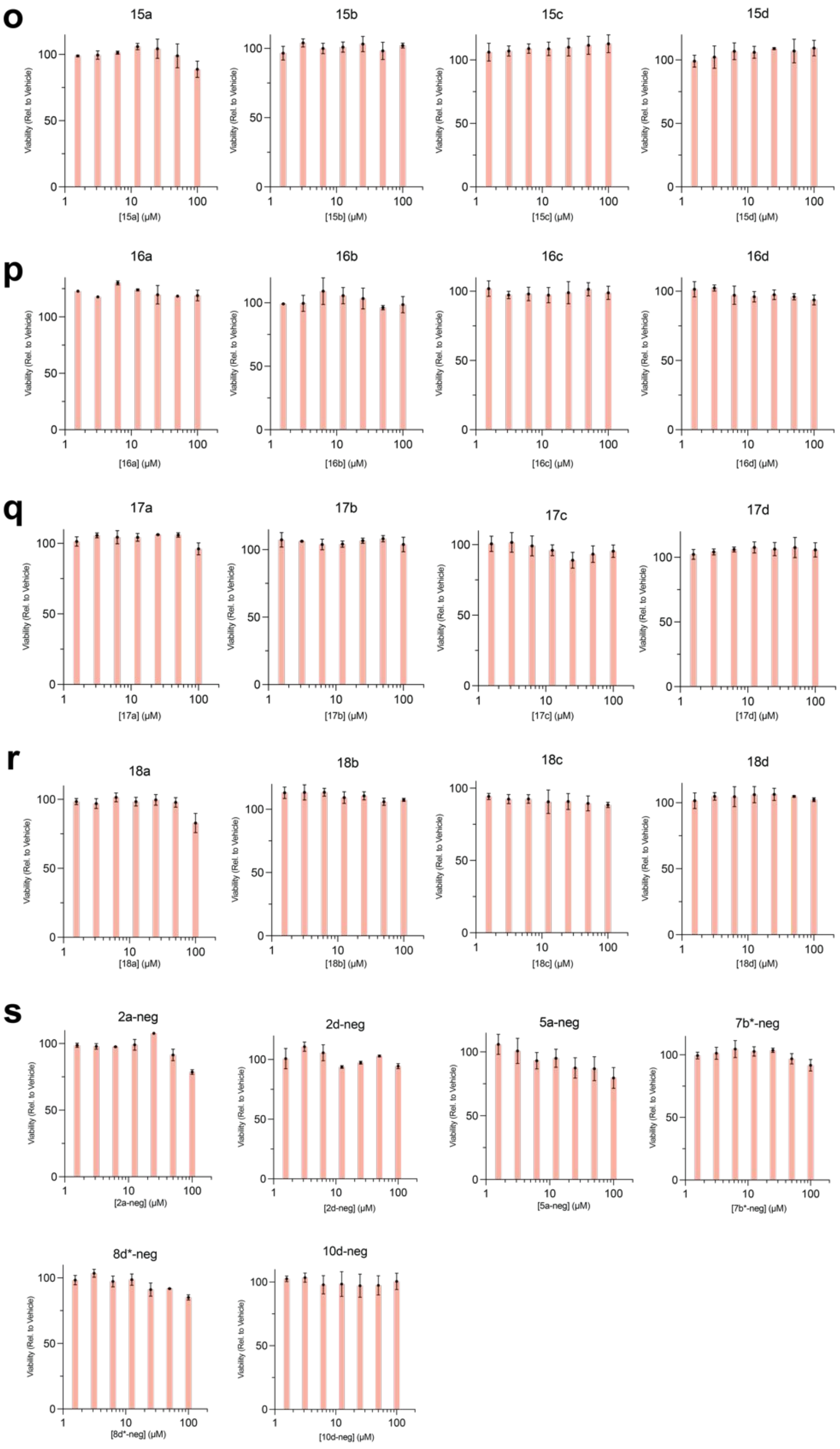

Fig. S3| Evaluation of AgnoTAC effects on cell viability. Cell viability was assessed using Cell Titer-Glo luminescence assay following MDA-MB-231 cell treatment with AgnoTACs at varying concentrations for 18 h. Results depict the relative luminescence percentage, normalized to DMSO vehicle. Error bars represent mean ± standard deviation (SD) from n = 3 independent experiments.

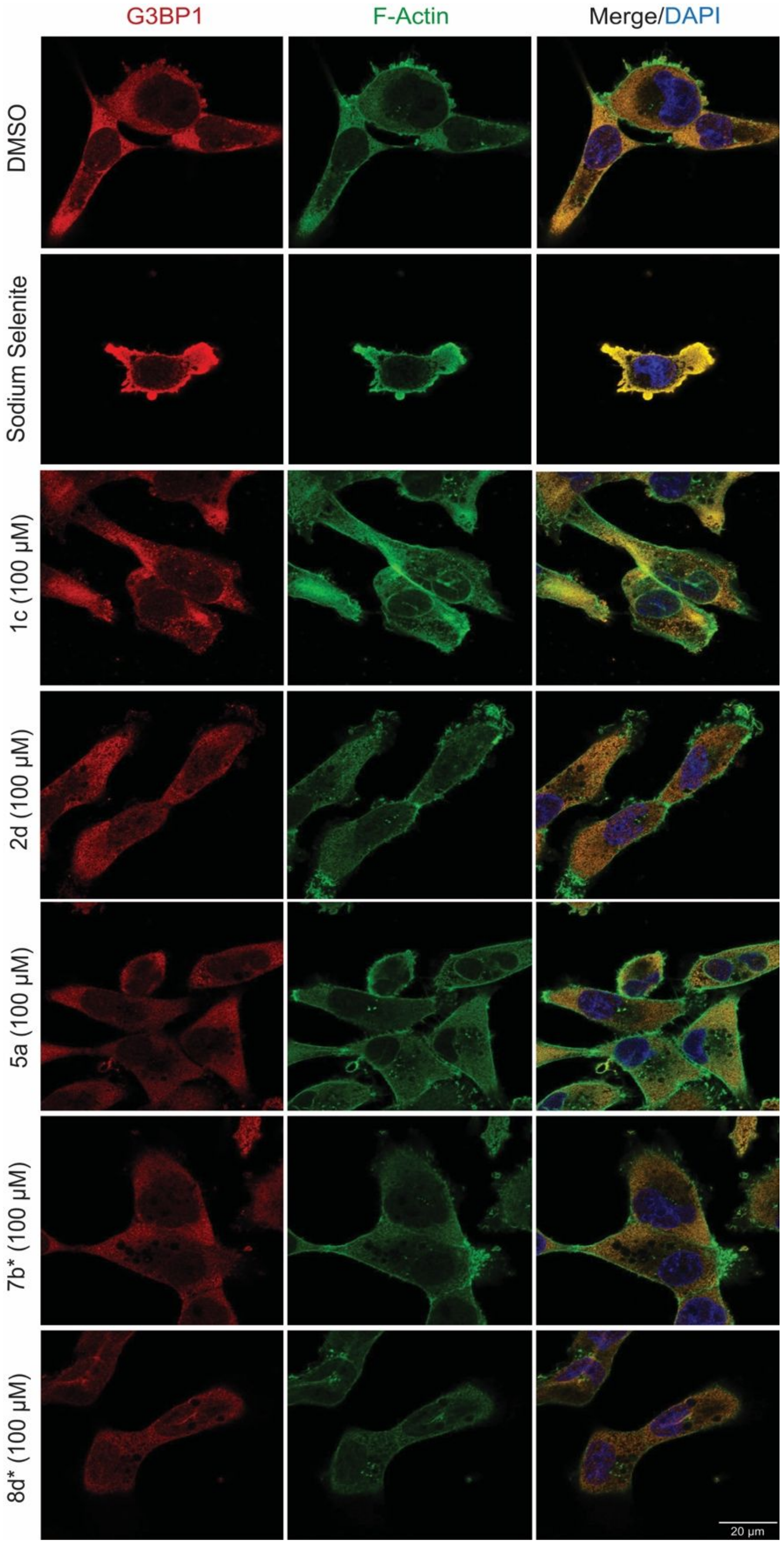

Fig. S4. AgnoTACs do not induce stress granule formation in cells. Representative images showing effects of AgnoTACs on protein aggregation. Cells were treated with DMSO, sodium selenite (400 µM) and various AgnoTACs (100 µM) for 6 h and stained for G3BP1 (stress granule marker) and F-actin (cytoskeletal structure marker).

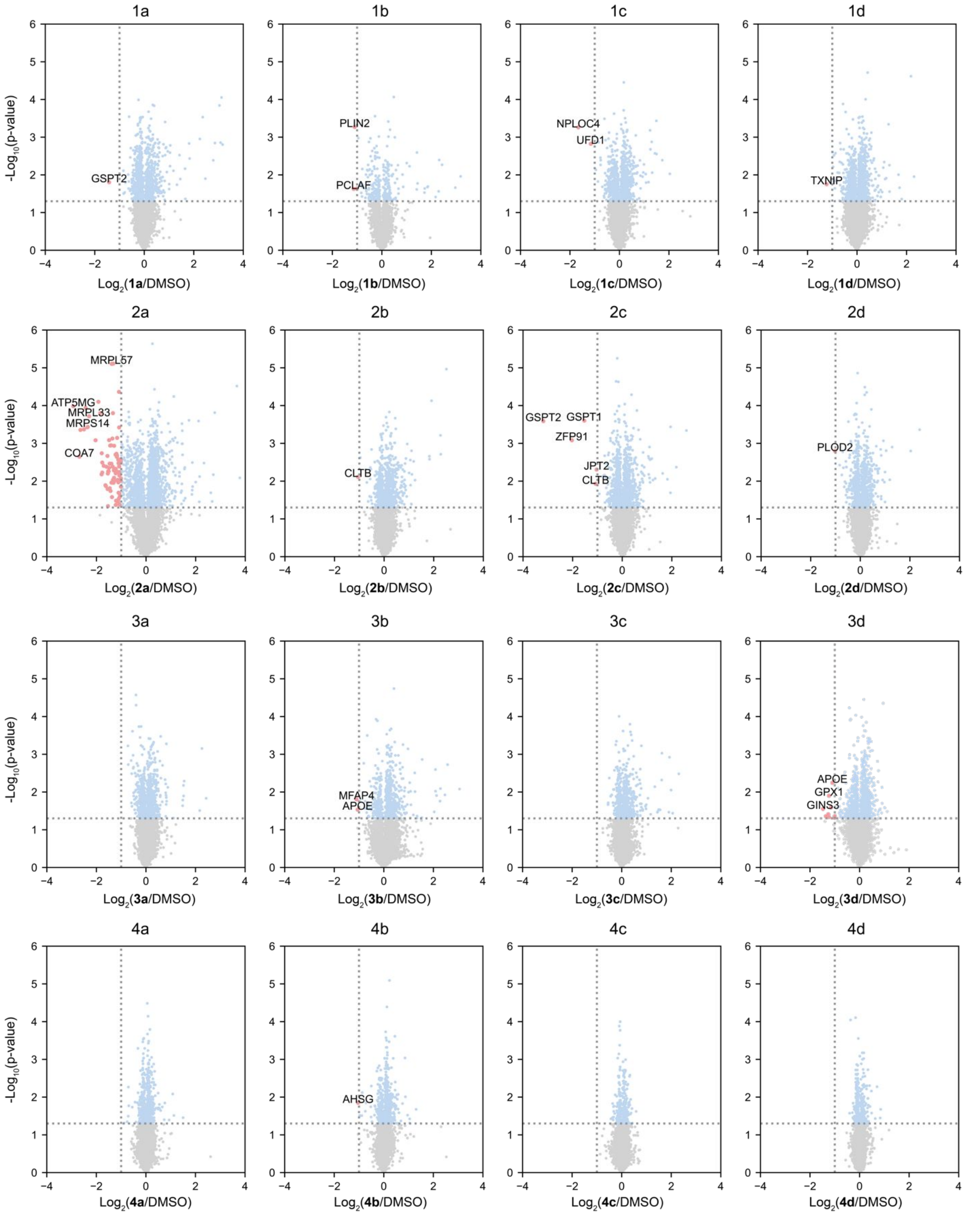

Fig. S5| Proteomic profiles of AgnoTAC library (continued on next page)

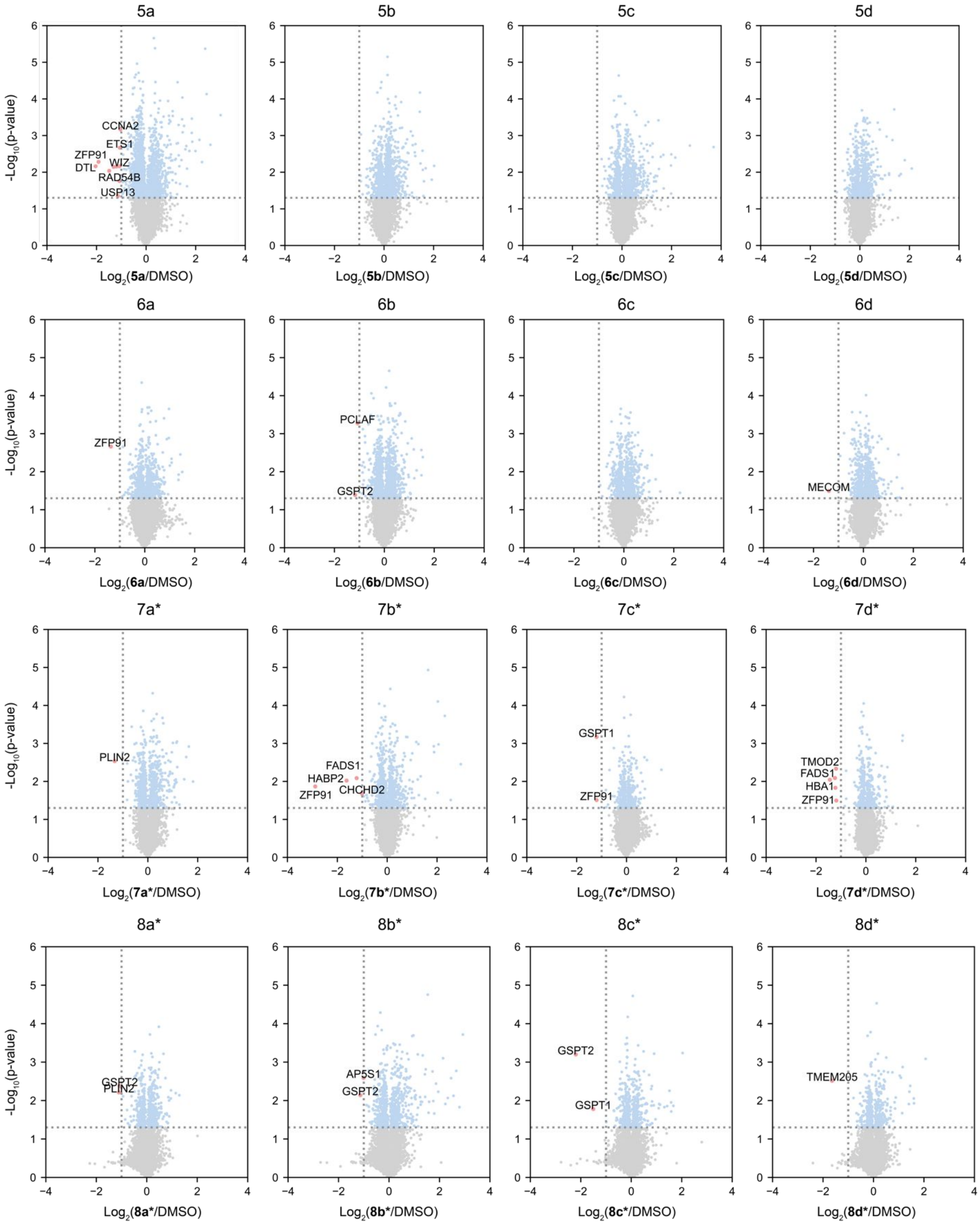

Fig. S5| Proteomic profiles of AgnoTAC library (continued on next page)

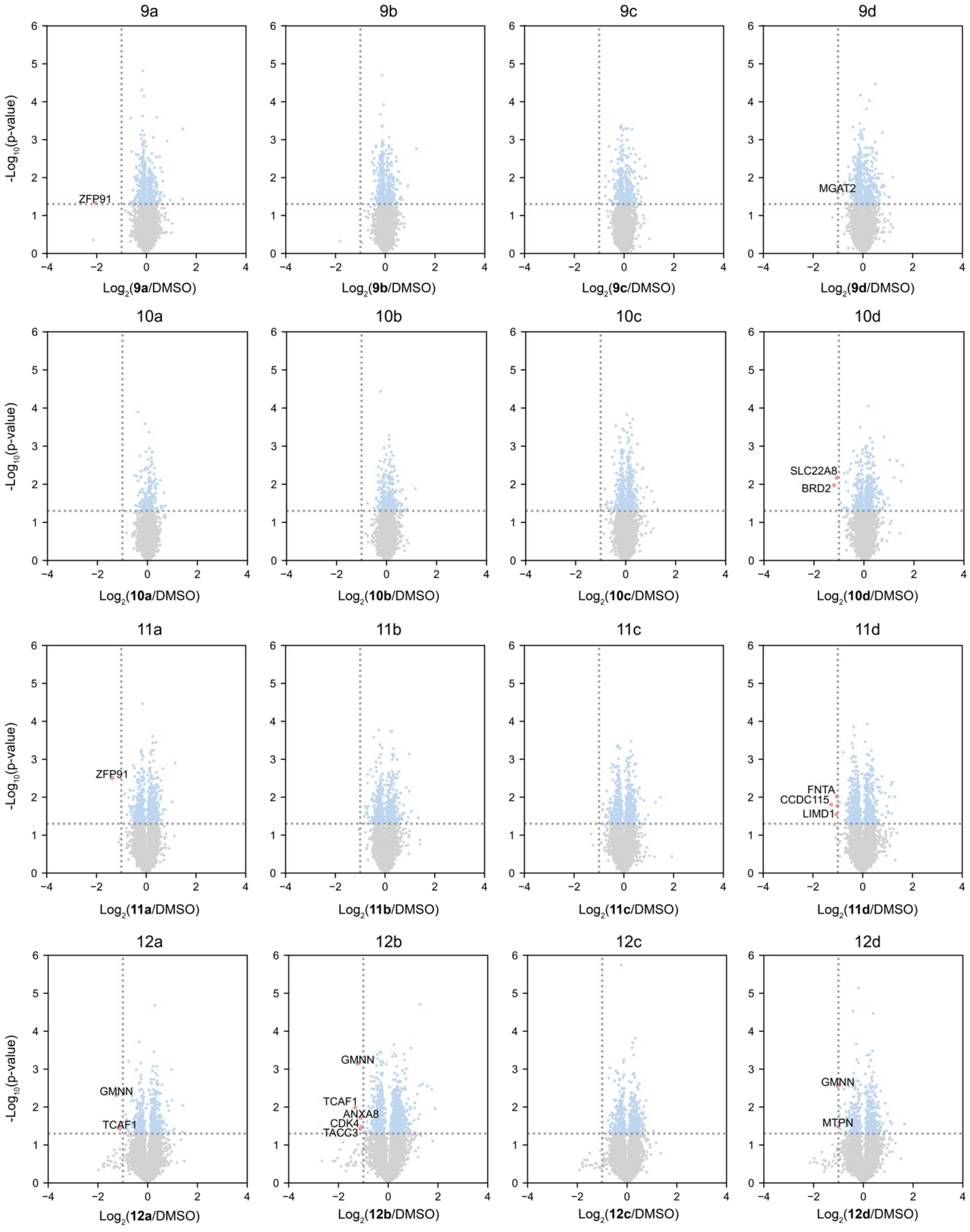

Fig. S5| Proteomic profiles of AgnoTAC library (continued on next page)

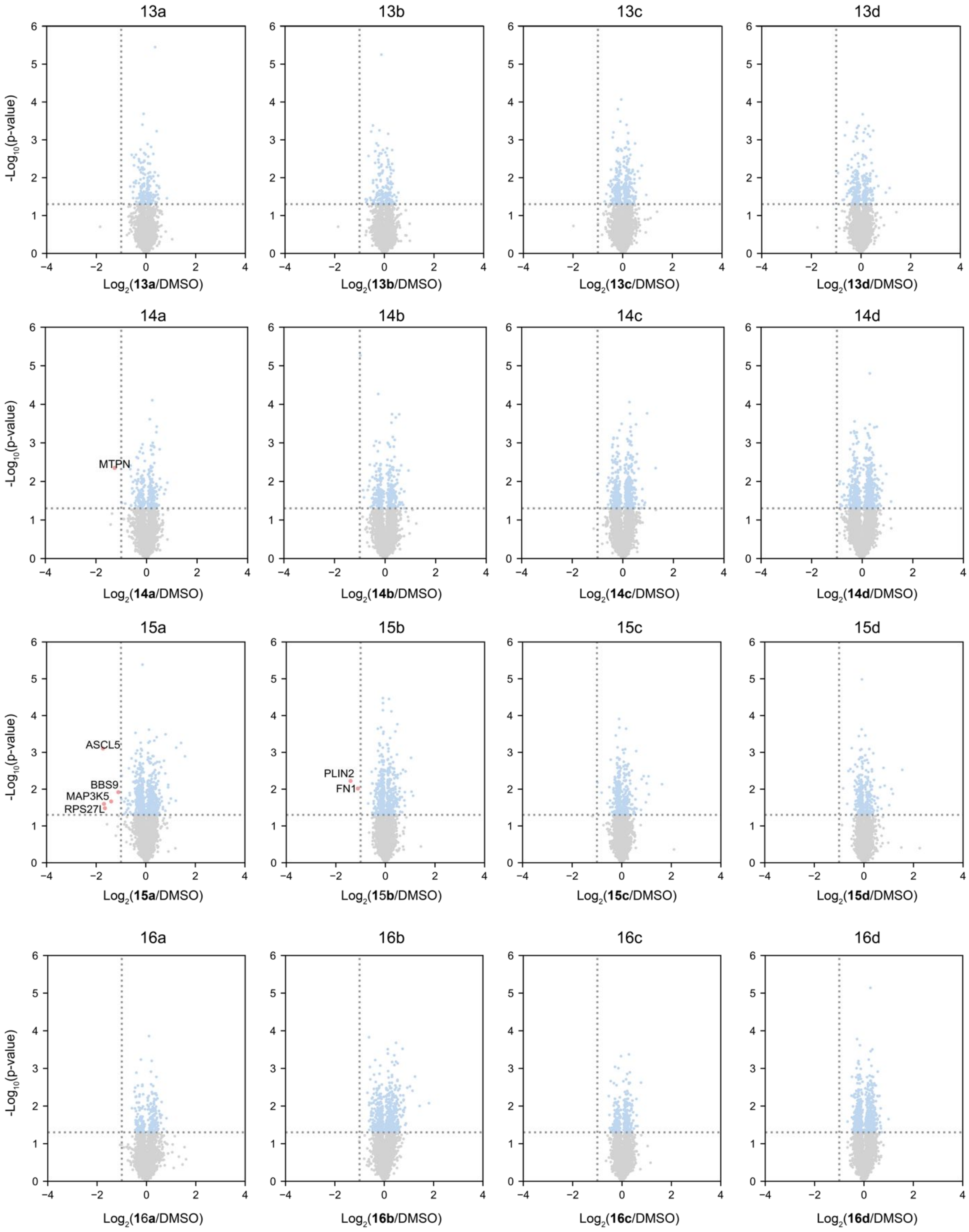

Fig. S5| Proteomic profiles of AgnoTAC library (continued on next page)

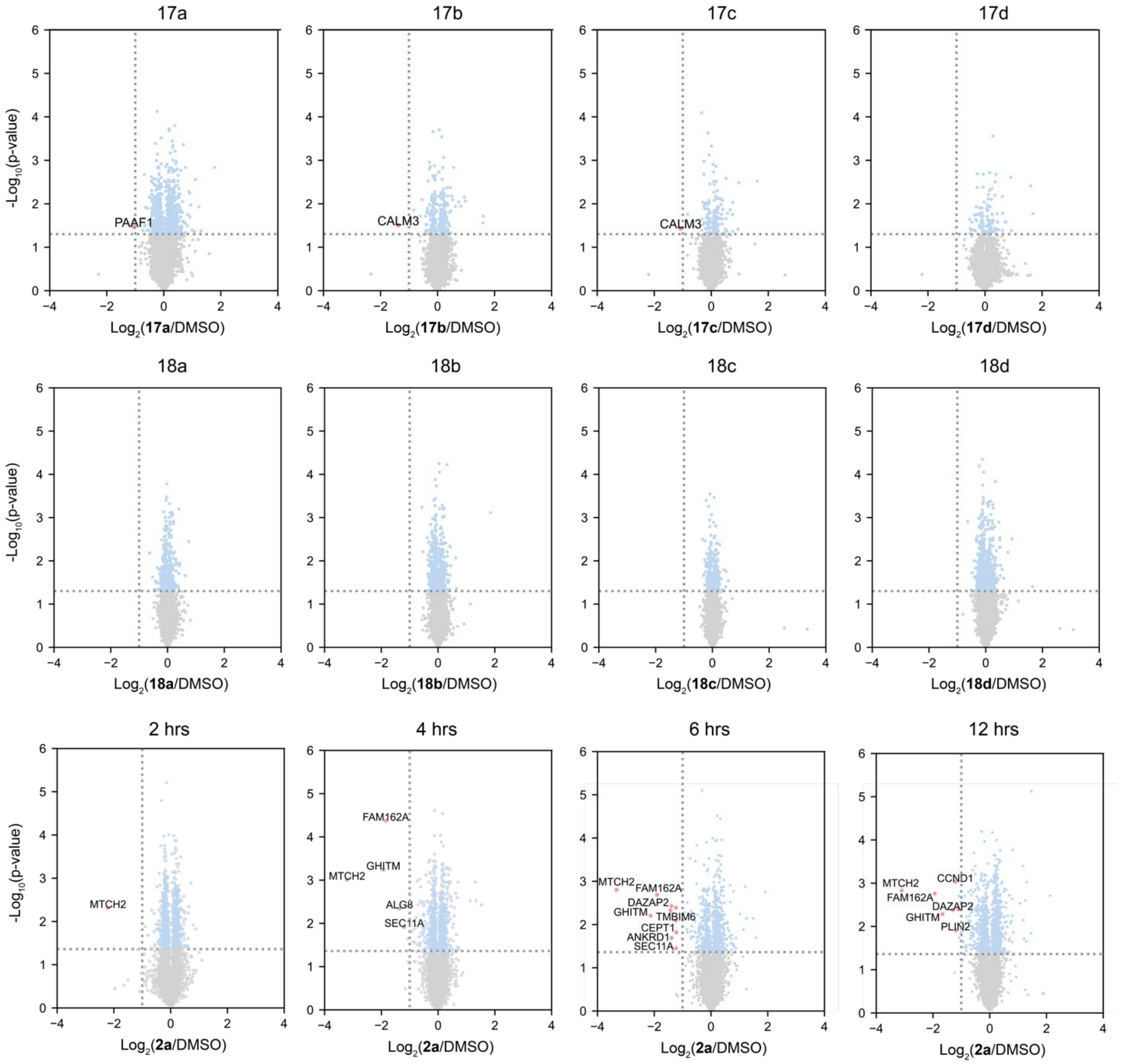

Fig. S5| Proteomic profiles of AgnoTAC library. Volcano plots of proteomic changes upon AgnoTAC treatment (18h). Detailed data sets and full list of downregulated targets can be found in **supplemental dataset 1**.

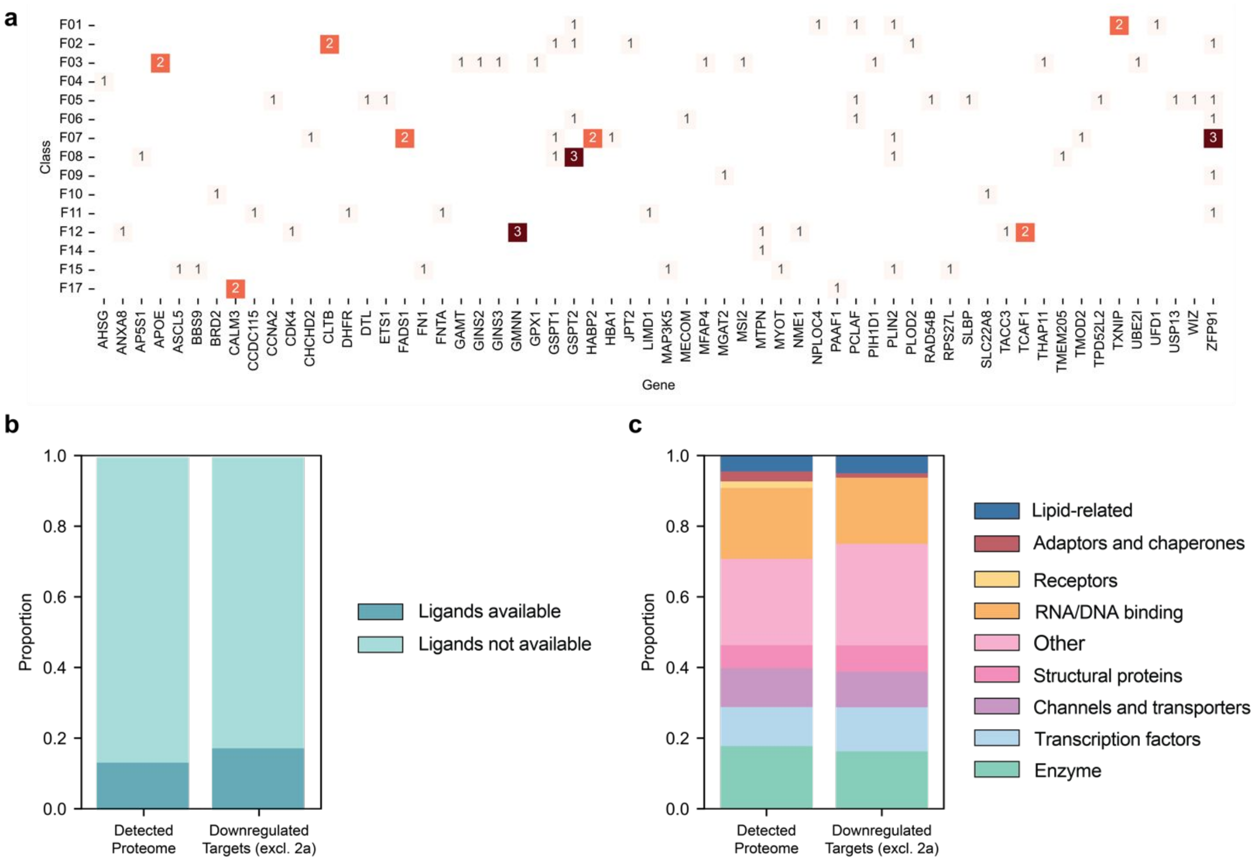

Fig. S6| Analysis of detected and downregulated targets. (**a**) Heatmap of unique targets significantly downregulated across headgroups. (**b, c**) Bar graphs comparing the proportion of downregulated targets (excluding cytotoxic AgnoTAC **2a**) to all detected proteins in MDA-MB-231 cells classified by (**b**) the availability of chemical probes as assigned by the DrugBank database and (**c**) protein functional classes as assigned by UniProt. The “Other” category refers to motor, metal-ion binding, carbohydrate-binding, protein-binding, heme-related, fatty acid metabolism-related proteins and uncharacterized proteins.

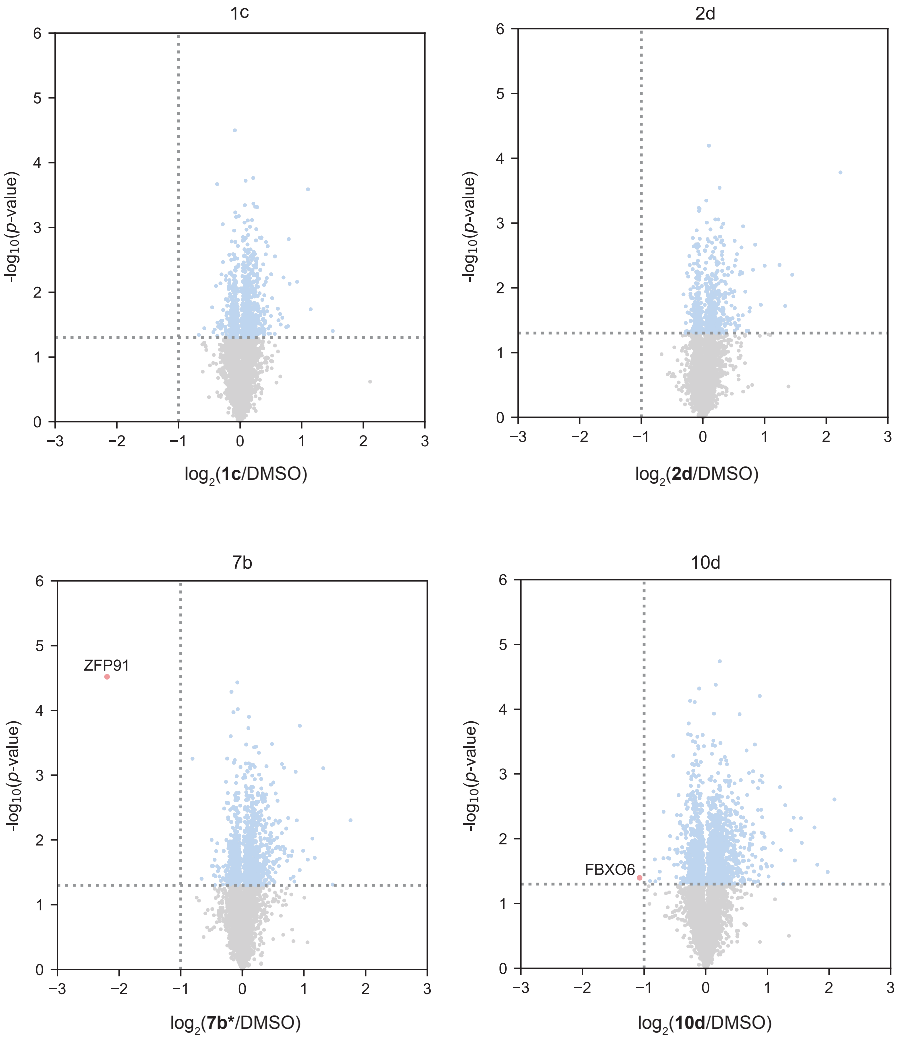

Fig. S7| Representative examples of proteomic profiles at lower AgnoTAC concentrations. Volcano plots depicting proteomic changes upon 10 µM treatments with select AgnoTACs. Detailed data sets and full list of downregulated targets can be found in **supplemental dataset 1.**

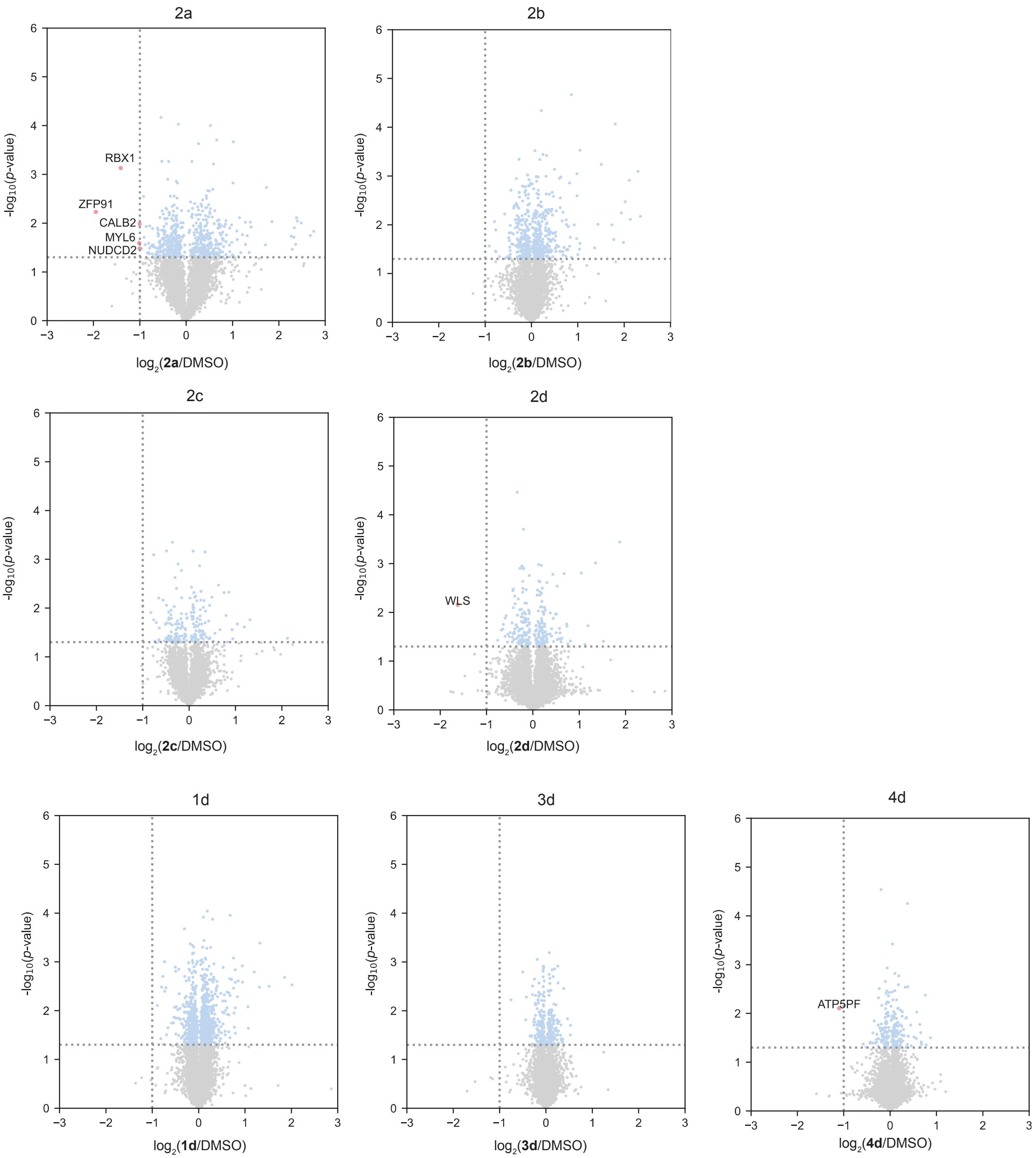

Fig. S8| Representative examples of proteomic profiles at shorter time points. Volcano plot depicting proteomic changes after 6 h treatments with select AgnoTACs. Detailed data sets and full list of downregulated targets can be found in **supplemental dataset 1.**

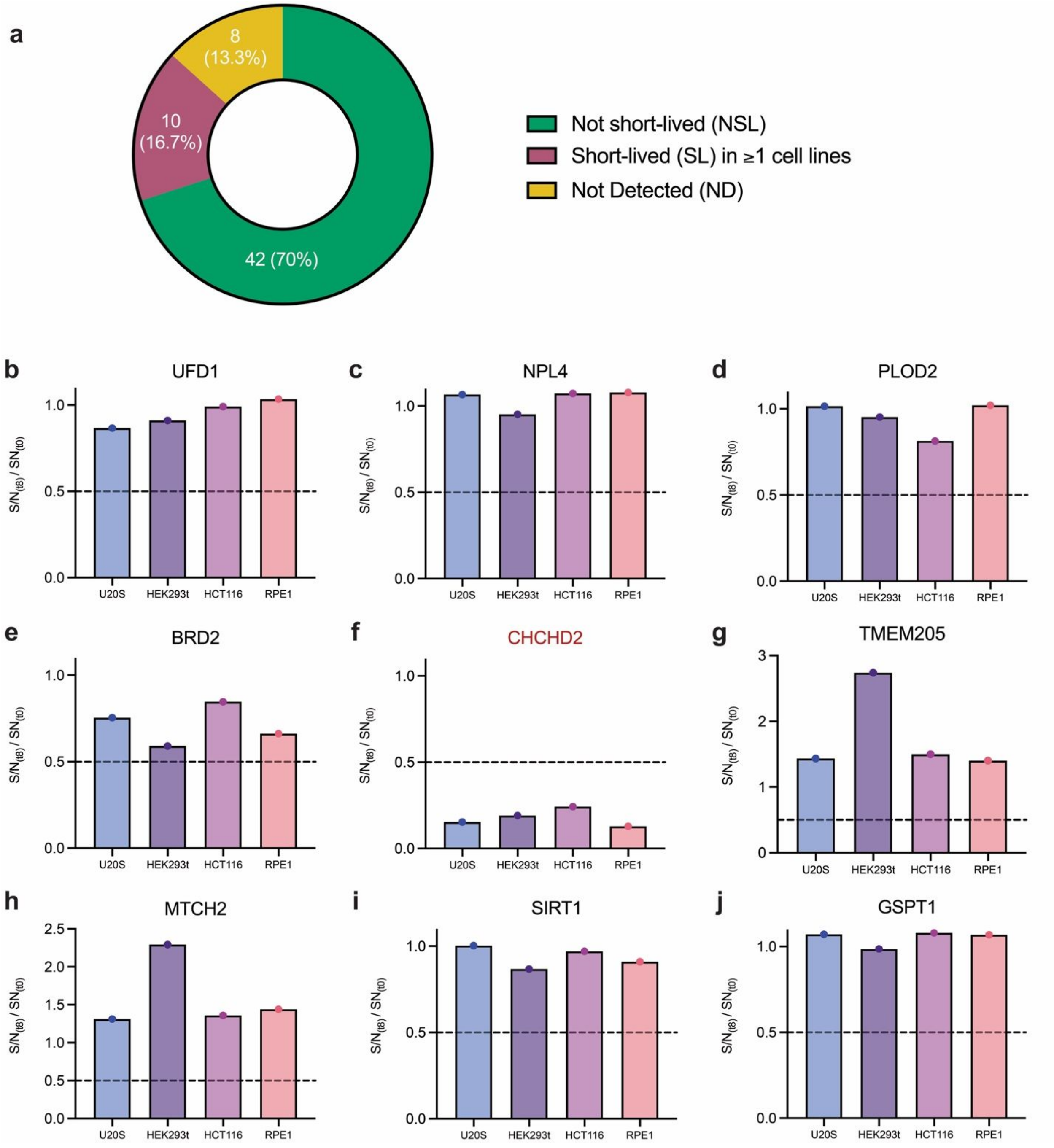

Fig. S9| Analysis of short-lived proteins across four cell lines. (a) Classification of not short-lived (NSL) and short-lived (SL) proteins amongst the 60 uniquely downregulated targets, based on the short-lived protein dataset from Li *et al*[1]. (b) Representative examples of NSL and SL proteins, with the signal-to-noise (S/N) ratio at t = 8 h relative to t = 0 h calculated in U2OS, HEK293T, HCT116 and RPE1 cells. The dashed line indicates the threshold below which a protein is considered short-lived according to Li *et al*[1] criteria. Associated calculations can be found in **supplemental dataset 2.**

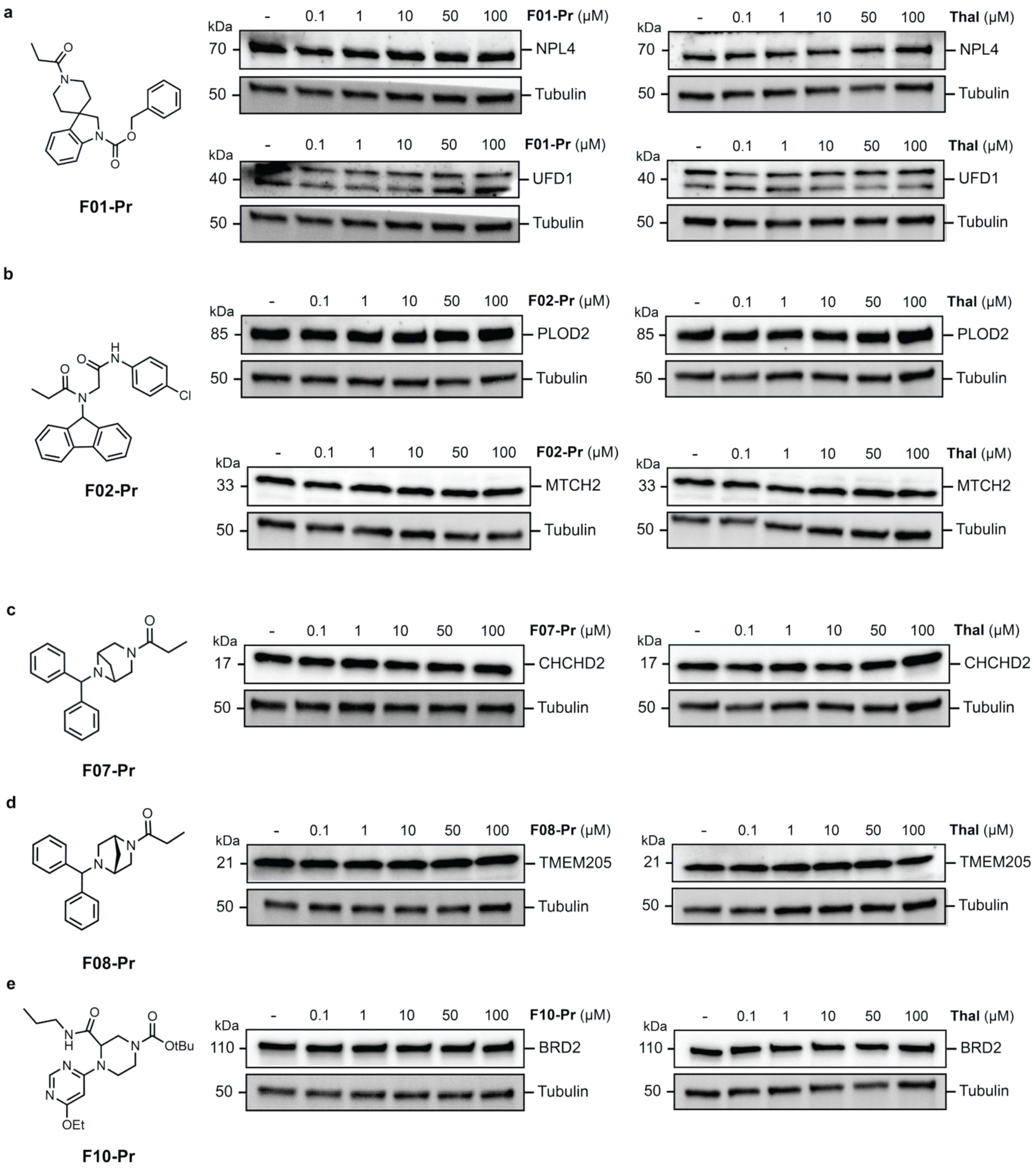

Fig. S10| Individual ligand treatments do not affect protein levels. Immunoblot analysis of validated target expression levels upon treatment with thalidomide (**Thal**) and propylated (Pr) headgroup scaffolds (**a**) **F01-Pr**, (**b**) **F02-Pr**, (**c**) **F07-Pr**, (**d**) **F08-Pr** and (**e**) **F10-Pr**. MDA-MB-231 cells were treated for 18 h with varying doses of individual ligands. Results are representative of n = 2 biological replicates.

a

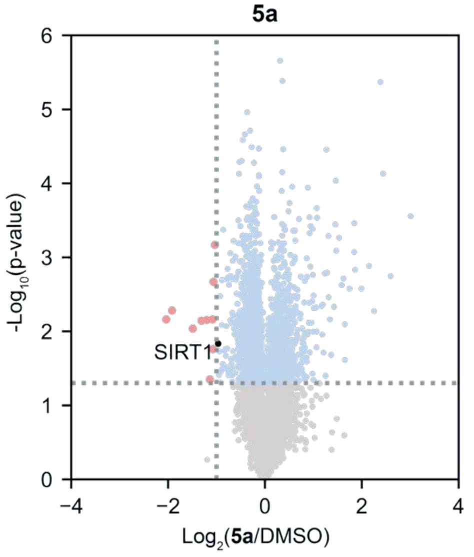

b

Protein	SIRT1
Fold Change (Ratio)	0.51
p-Value	0.015
Unique Peptides	3
PSMs	4

c

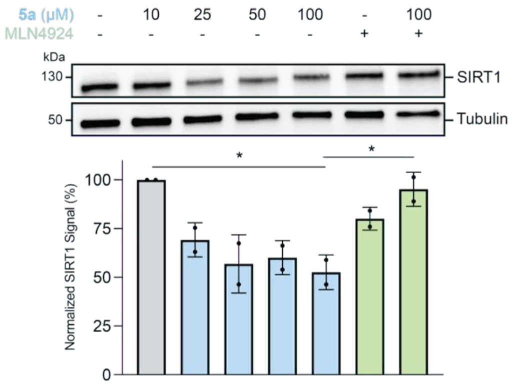

d

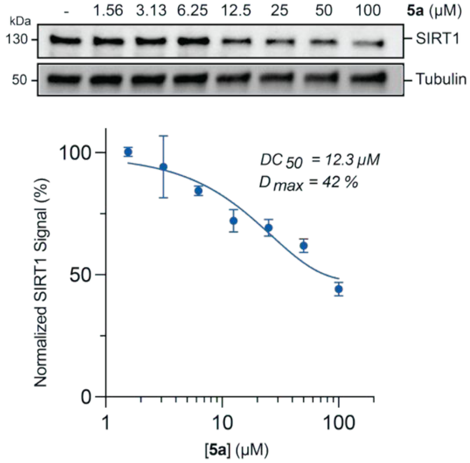

Fig. S11| Additional target degraded by AgnoTAC 5a. (**a**) Volcano plot depicting global protein abundance changes upon **5a** treatment of MDA-MB-231 cells for 18 h. SIRT1 downregulation is highlighted by a black dot. (**b**) Fold change, p-value, unique peptide and peptide-spectrum matches (PSMs) for SIRT1 deacetylase. (**c, d**) Dose-dependent, proteasome, and neddylation-mediated degradation of SIRT1.

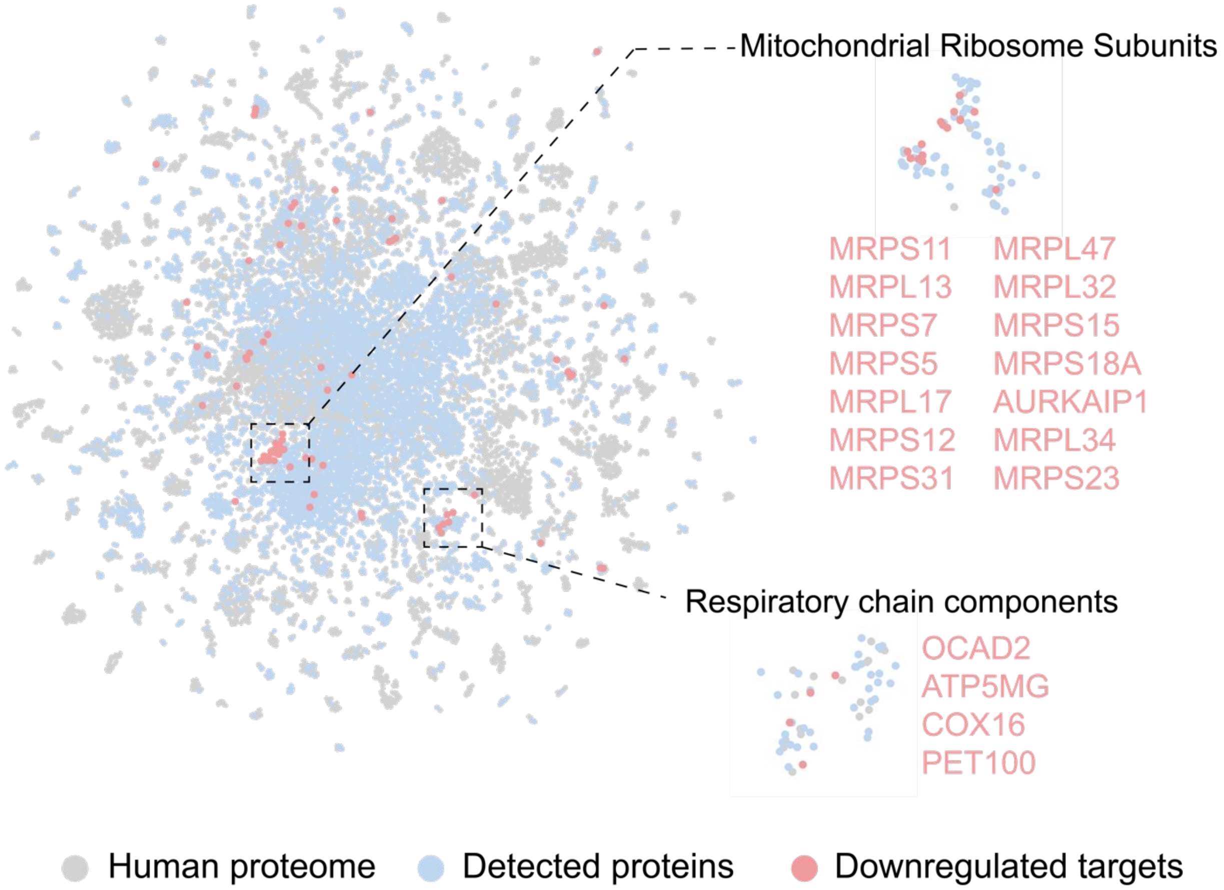

Fig. S12| Protein cluster analysis of 2a-treated samples. 2D UMAP projection illustrating clustered proteins following MDA-MB-231 cell treatment with 2a for 18 h. Gray dots represent all human proteins, blue dots represent detected proteins, and red dots highlight downregulated targets. Dashed insets emphasize protein clusters.

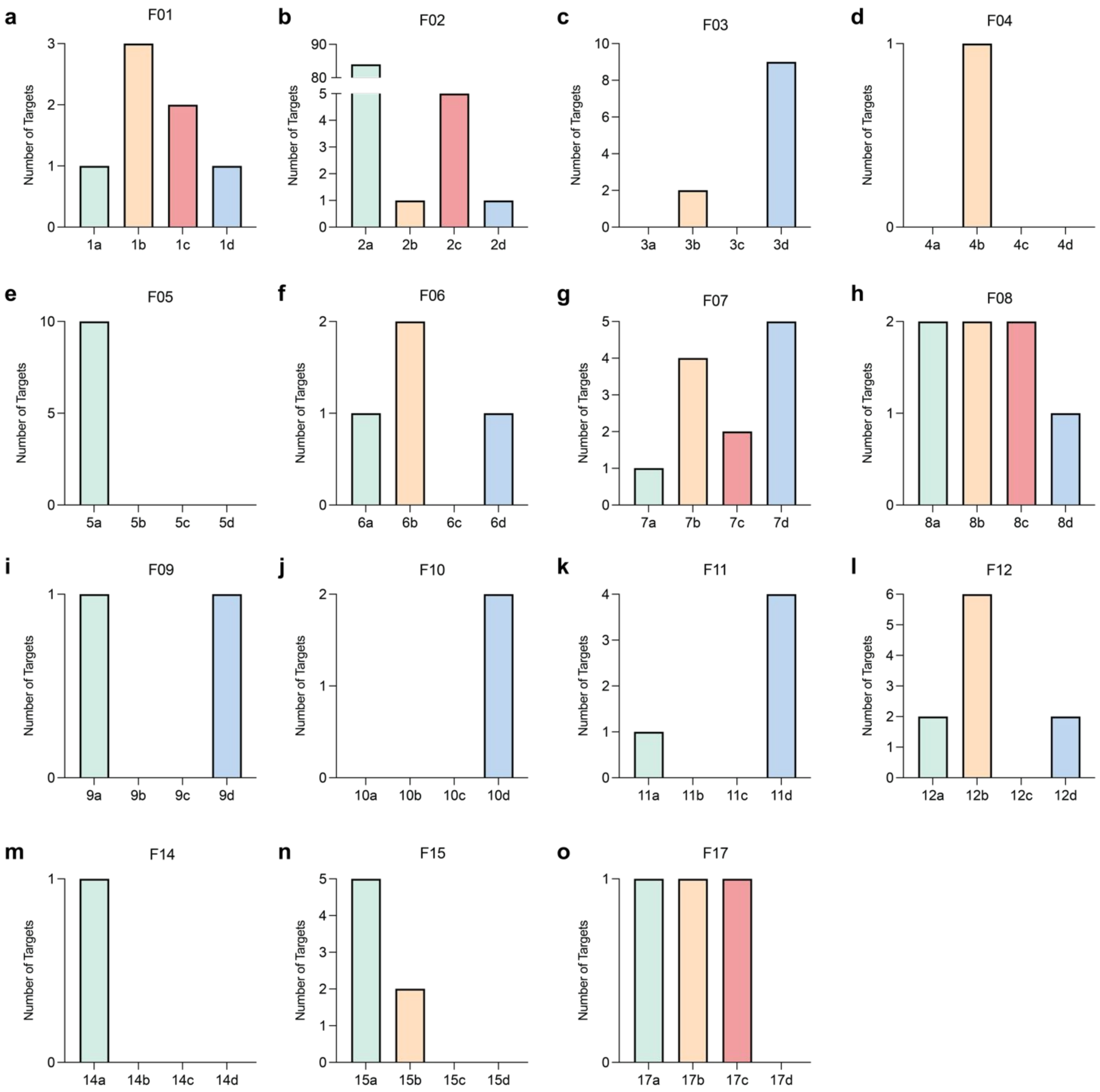

Fig. S13| Linker composition influences degradation profiles, related to Fig. 5. (**a-o**) Bar plots depicting total number of targets downregulated by each probe across all 18 headgroups. AgnoTACs corresponding to headgroups not displayed (i.e., F13, F16 and F18) did not significantly downregulate any target. Summary of results (excluding AgnoTAC 2a) is provided in **Fig. 3a.** Associated data can be found in **supplemental dataset 2**.

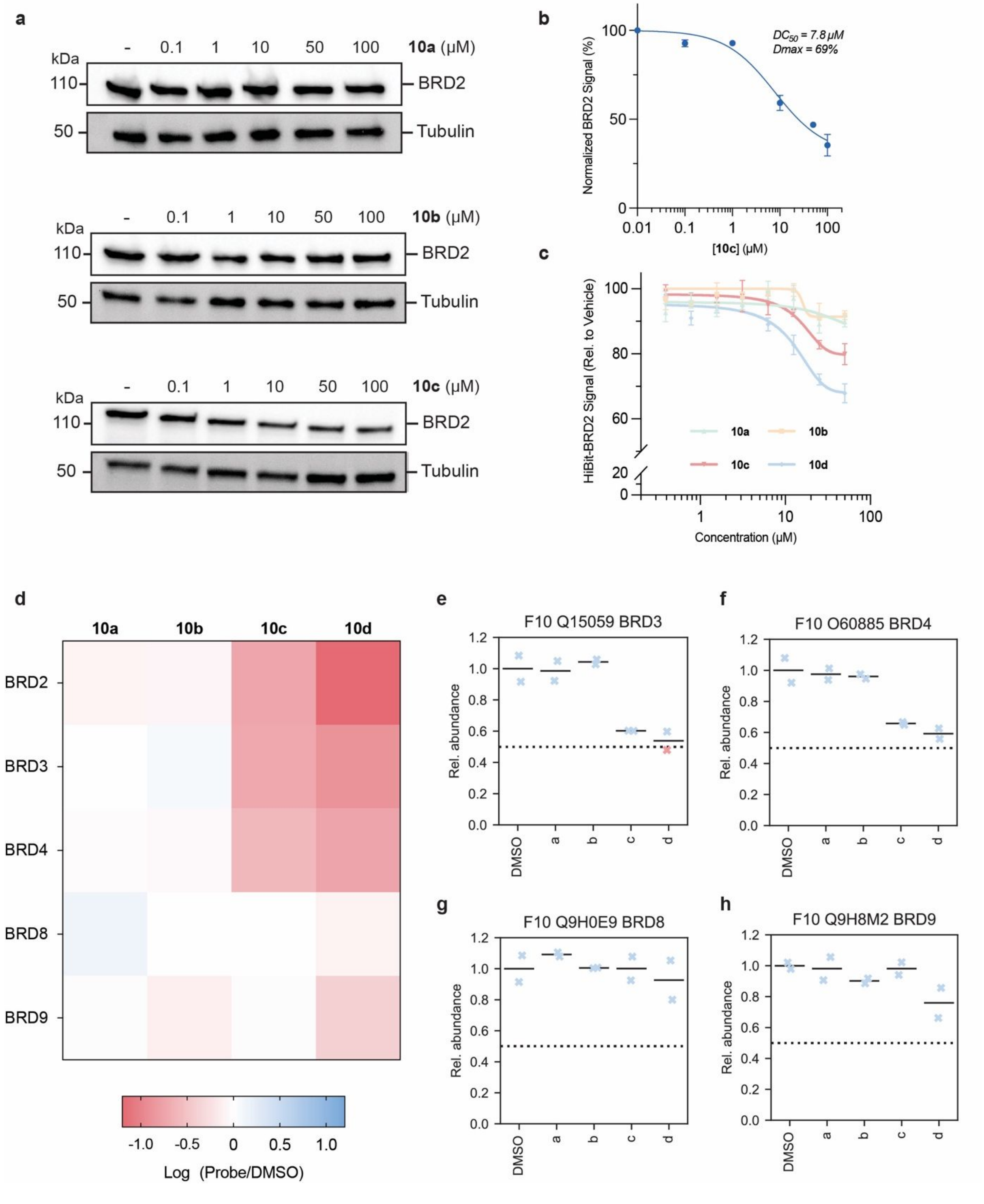

Fig. S14| Effect of F10 AgnoTACs on bromodomain protein levels. (**a**) Immunoblot analyses of BRD2 degradation by F10 AgnoTACs and (**b**) corresponding dose-response curve following treatment by additional hit **10c.** (**c**) BRD2 degradation assessed by HiBiT assay. HEK293t cells containing endogenously tagged HiBiT-BRD2 were treated with increasing doses of **10a**, **10b**, **10c** or **10d**. Data was normalized to DMSO control. Errors bars are expressed as mean

± SD (n = 3 biological replicates). (**d**) Heatmap of additional bromodomain proteins identified in F10 AgnoTAC-treated samples. (**e-h**) Mean proteomic abundance ratios for (**e**) BRD3, (**f**) BRD4, (**g**) BRD8 and (**h**) BRD9 proteins.

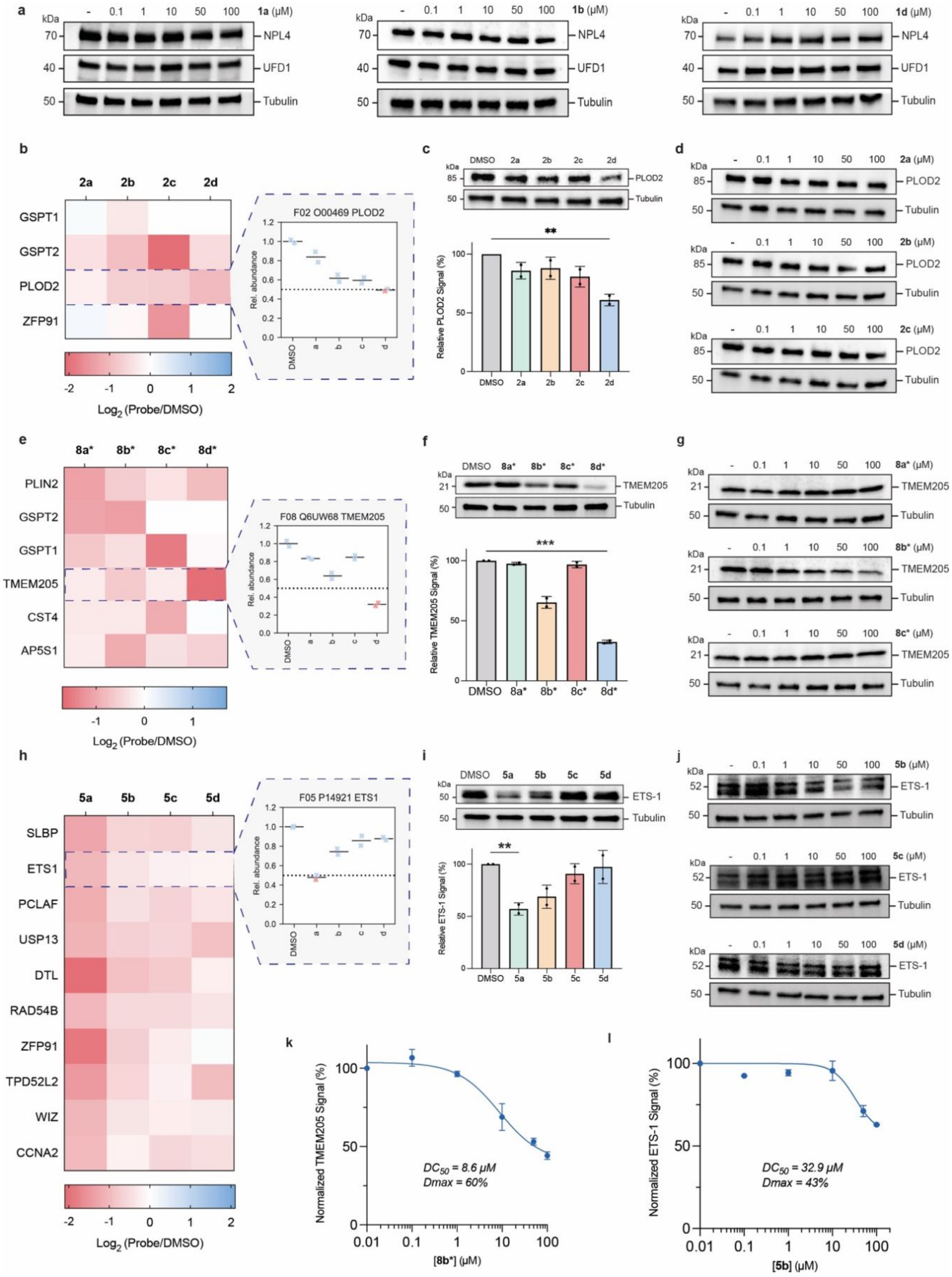

Fig. S15| Additional linker effects, related to Fig. 5. (**a-i**) Additional degradability heatmaps and abundance ratios highlighting linker-dependent changes for (**a-c**) PLOD2, (**d-f**) TMEM205 and (**g-i**) ETS-1. Immunoblots were obtained after 18 h AgnoTAC treatments. Error bars in bar plots represent mean ± SD (n = 2 biological replicates). (**j, k**) Dose-response curves for additional hits degraded by (**j**) **8b*** (TMEM205) and (k) **5b** (ETS-1) uncovered from embedded linkerology. Results are representative of n = 2 biological replicates.

a

Gene	Accession	AA_Start	AA_End	Turn Query	Peptide	Turn Overlap	Control Align	Query Align	Overlap Align	RMSD
**ZFP91**	Q96JP5	400	409	LQCEICGFT	LQCEICGFTC	*..**.	FQCNQCGASF	LQCEICGFTC	.**..**...	0.192818418
**ZFP91**	Q96JP5	430	439	FSCNICGKK	FSCNICGKKF	**.**.	FQCNQCGASF	FSCNICGKKF	*.**.**..*	0.55828917
**GSPT2**	Q8IYD1	548	593	LVDKKSGEK	CIEEVEITALISLVDKKSGEKSKTRPRFVKQDQVCIARLRTAGTI	....*.	------------FQCNQCGASF------------------------	CIEEVEITALISLVDKKSGEKSKTRPRFVKQDQVCIARLRTAGTI	......*...	0.443048328
**MECOM**	Q03112	348	357	HACPECGKT	HACPECGKTF	*..**.	FQCNQCGASF	HACPECGKTF	..*..**..*	0.372687995
**MECOM**	Q03112	405	414	IKCKDCGQM	IKCKDCGQMF	*..**.	FQCNQCGASF	IKCKDCGQMF	..*..**..*	0.60982269
**MECOM**	Q03112	912	921	YTCRYCGKI	YTCRYCGKIF	*..**.	FQCNQCGASF	YTCRYCGKIF	..*..**..*	0.435923904
**GSPT1**	P15170	419	442	LVDKKSGEK	CIEEVEITALICLVDKKSGEKSKT	....*.	------------FQCNQCGASF--	CIEEVEITALICLVDKKSGEKSKT	......*..	0.422952771
**CDK4**	P11802	18	40	ARDPHSGHF	GTVYKARDPHSGHFVALKSVRVP	....*.	-----FQCNQCGASF--------	GTVYKARDPHSGHFVALKSVRVP	......*...	0.737835705
**FN1**	P02751	96	107	CFDKYTGNT	TCFDKYTGNTYR	....*.	-FQCNQCGASF-	TCFDKYTGNTYR	......*...	0.959801316
**FN1**	P02751	560	571	CQDSETGTF	QCQDSETGTFYQ	....*.	-FQCNQCGASF-	QCQDSETGTFYQ	.*....*...	0.718473136
**PAAF1**	Q9BRP4	116	130	IWQASNGEL	MKIWQASNGELRRVL	....*.	--FQCNQCGASF---	MKIWQASNGELRRVL	......*..	0.382800102
**MAP3K5**	Q99683	809	820	LINTYSGVL	VLINTYSGVLKI	....*.	-FQCNQCGASF-	VLINTYSGVLKI	......*...	0.534017503
**WIZ**	O95785	304	313	LACGECGWA	LACGECGWAF	*..**.	FQCNQCGASF	LACGECGWAF	..*..**..*	0.494413674
**WIZ**	O95785	416	425	SACVFCGFP	SACVFCGFPA	*..**.	FQCNQCGASF	SACVFCGFPA	..*..**..	0.304864138
**WIZ**	O95785	870	879	TTCEVCGAC	TTCEVCGACF	*..***	FQCNQCGASF	TTCEVCGACF	..*..***.*	0.403276116
**WIZ**	O95785	1043	1052	IRCEFCGEF	IRCEFCGEFF	*..**.	FQCNQCGASF----------	IRCEFCGEFFIRCEFCGEFF	..*..**..*	0.516328692
**WIZ**	O95785	1227	1236	IRCEFCGEF	IRCEFCGEFF	*..**.	FQCNQCGASF----------	IRCEFCGEFFIRCEFCGEFF	..*..**..*	0.516328692

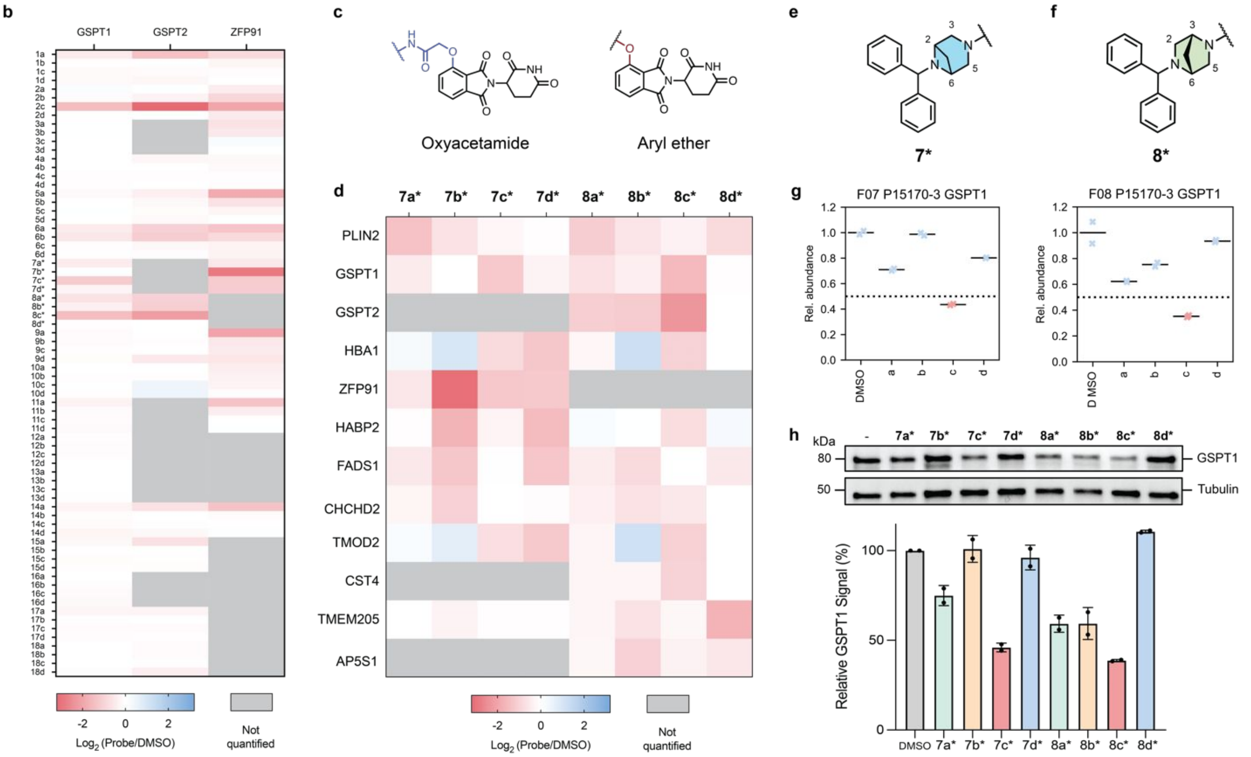

Fig. S16| Analysis of molecular glue off-targets. (**a**) Heatmap depicting changes in the abundances of known molecular glue targets, including GSPT1, GSPT2 and ZFP91, across all 72 AgnoTACs. (**b**) Structural comparison of 4-oxyacetamide and 4-hydroxythalidomide (aryl ether) moieties. (**c**) Heatmap displaying $\log_2$(fold-change) values of proteins targeted by structurally similar **F07***- and **F08***-based AgnoTAC analogs. (**d, e**) Chemical structures of **F07*** and **F08*** headgroups. (**f**) Comparison of GSPT1 mean proteomic ratios following treatment with **F07*** and **F08***. (**g**) Immunoblot validation of GSPT1 downregulation by **F07***- and **F08***. Error bars in bar plot represent mean ± SD (n = 2 biological replicates). All experiments were performed in MDA-MB-231 cells treated for 18 h. (**h**) Table of AgnoTAC targets possessing motifs that resemble b-hairpin G-loop structures.

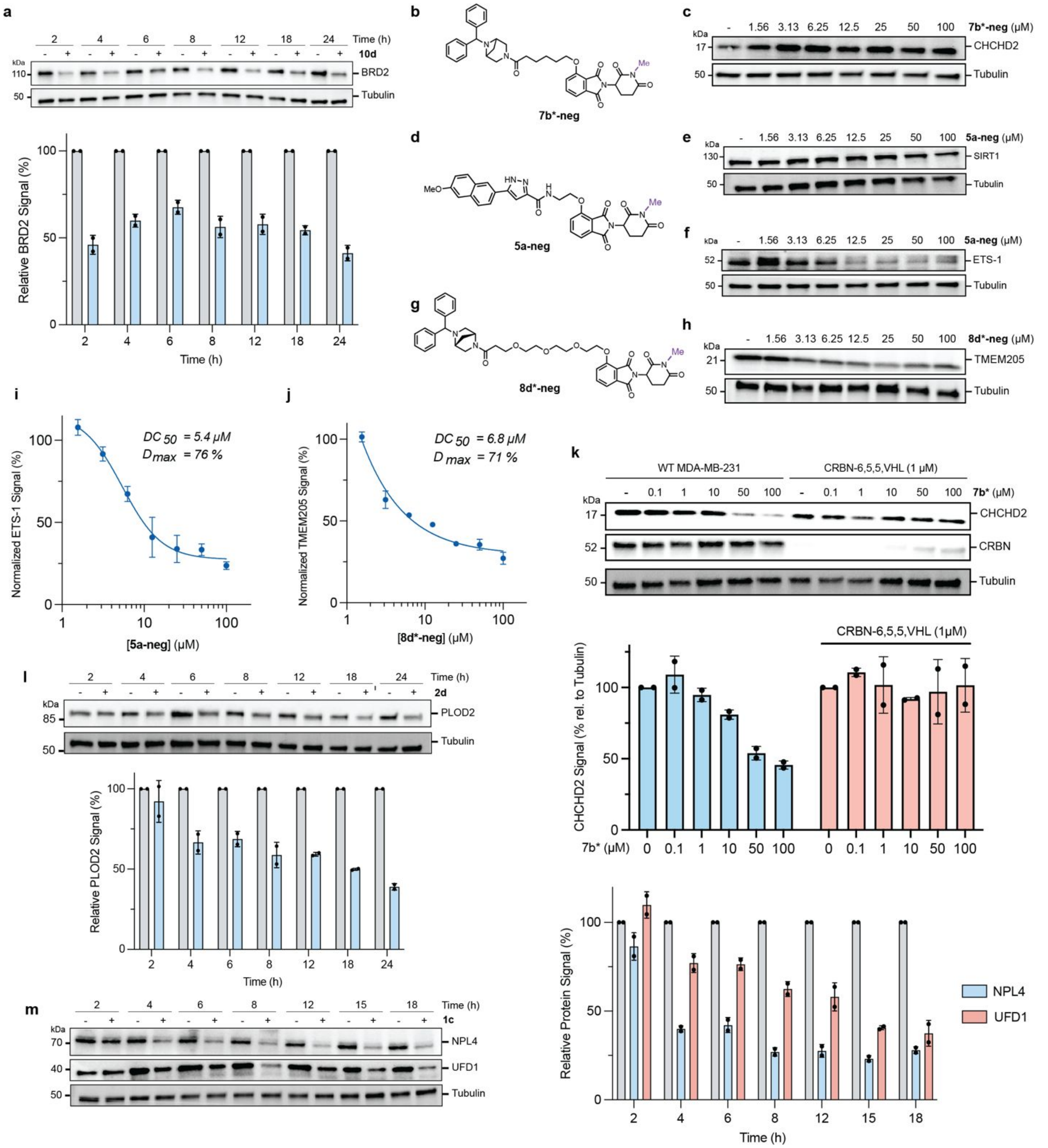

Fig. S17| Additional mechanistic characterization of AgnoTAC targets. (**a**) Time-course analysis of BRD2 degradation following treatment of MDA-MB-231 cells with 50 µM **10d** at indicated times and quantitative analysis (normalized to loading control). (**b-h**) Chemical structures of additional negative controls and corresponding immunoblot dose-response analyses for (**b, c**) CHCHD2 upon **7b*-neg** treatment, (**d-f**) SIRT1 and ETS-1 following **5a-neg** treatment, and (**g, h**) TMEM205 levels after **8d*-neg** treatment. (**i, j**) Dose-dependent depletion of (**j**) ETS-1 by **5a-neg** and (**k**) TMEM205 by **8d*-neg**, highlighting Cullin-independent degradation mechanism. All shown results were obtained after 18 h treatments of MDA-MB-231 cells at indicated concentrations. Dose response curves for ETS-1 and TMEM205 are representative of n = 2 biological replicates. (**k**) Immunoblot analysis and quantification of (**k**) CHCHD2 levels following CRBN-induced degradation (mean ± SD, n = 2 independent experiments). (**l, m**) Time-course analyses of (**l**) PLOD2 degradation by **2d** (50 µM) and (**m**) NPL4/UFD1 degradation by **1c** (40 µM) at indicated times in MDA-MB-231 cells.

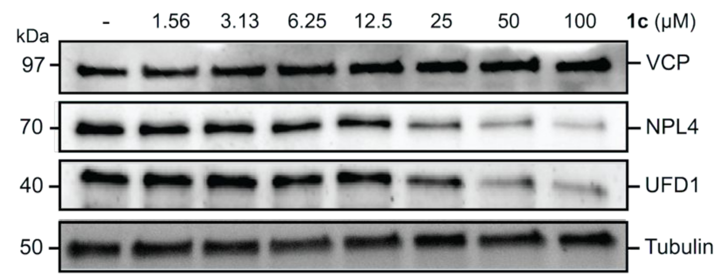

b

Accession	Q8TAT6	Q92890-1	P55072
Gene Symbol	NPLOC4	UFD1	VCP
PSMs	69	49	558
Unique Peptides	24	14	59
Abundances DMSO_1	7619.228	5033.294	47173.725
Abundances DMSO_2	7596.641	5154.900	48949.801
Abundances 1c_1	2525.839	2369.065	50663.734
Abundances 1c_2	2282.569	2187.830	53839.850
Average Fold Change (FC)	0.316	0.447	1.087
Log_2(FC)	-1.66	-1.16	0.121
p-Value	0.0006	0.0015	0.148
-Log_{10}(p-Value)	3.26	2.82	0.830

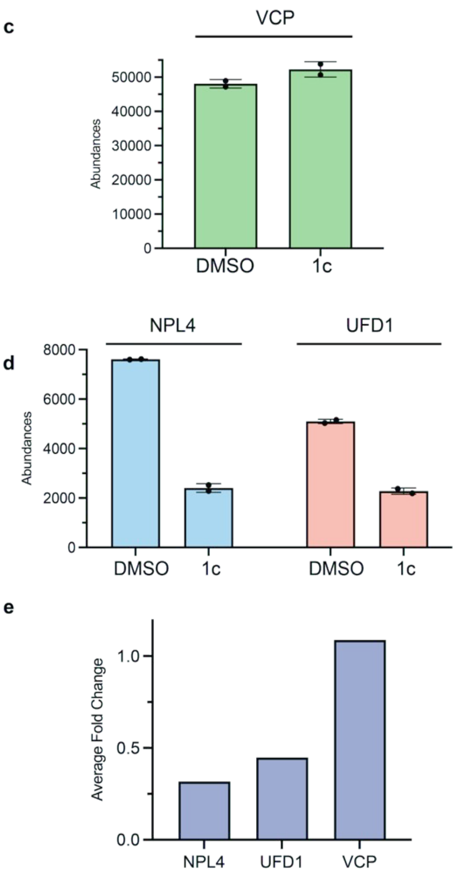

Fig. S18| AgnoTAC 1c does not induce changes in VCP protein abundance. (**a**) Immunoblot analysis of VCP, NPL4 and UFD1 protein levels following treatment of MDA-MB-231 cells with increasing concentrations of **1c** for 18 h. (**b**) Summary of quantitative proteomic results. (**c, d**) Comparative analysis of protein abundance between DMSO- and **1c**-treated samples for (**c**) VCP and (**d**) NPL4 and UFD1. (**e**) Fold change comparisons from proteomic anlaysis. All bar plots represent n = 2 biological replicates. Associated data can be found in **supplemental dataset 1**.

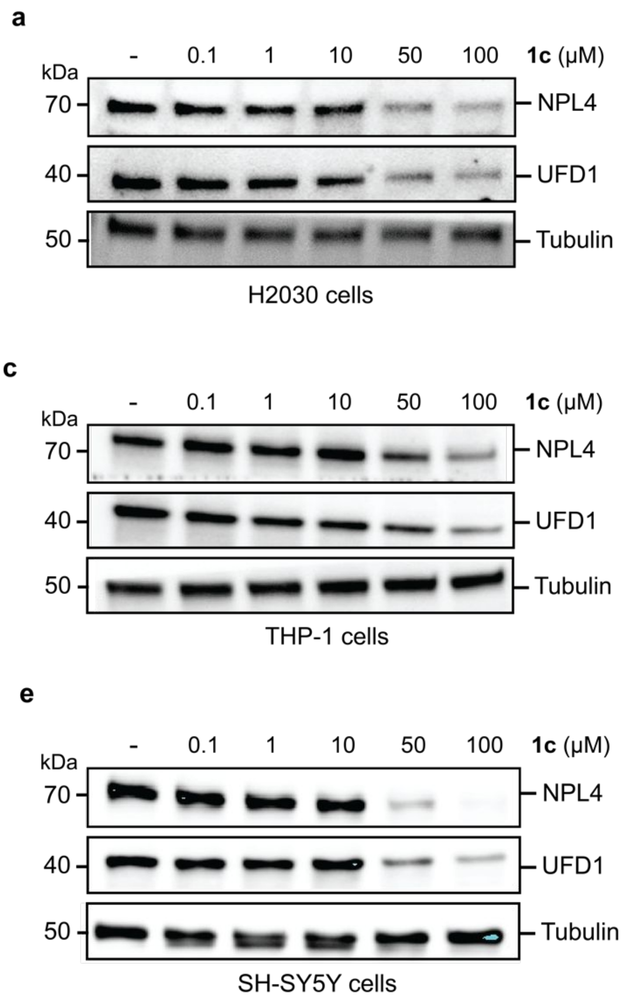

Fig. S19| AgnoTAC 1c degrades UFD1/NPL4 in several cell lines. Immunoblot analysis of NPL4 and UFD1 downregulation in (**a**) H2030 and (**b**) H23 non-small lung cancer cells, (**c**) THP-1 leukemia monocytes, (**d**) HEK293t kidney epithelial cells, and (**e**) SH-SY5Y neuroblastoma cells upon **1c** treatment at indicated concentrations for 6 h.

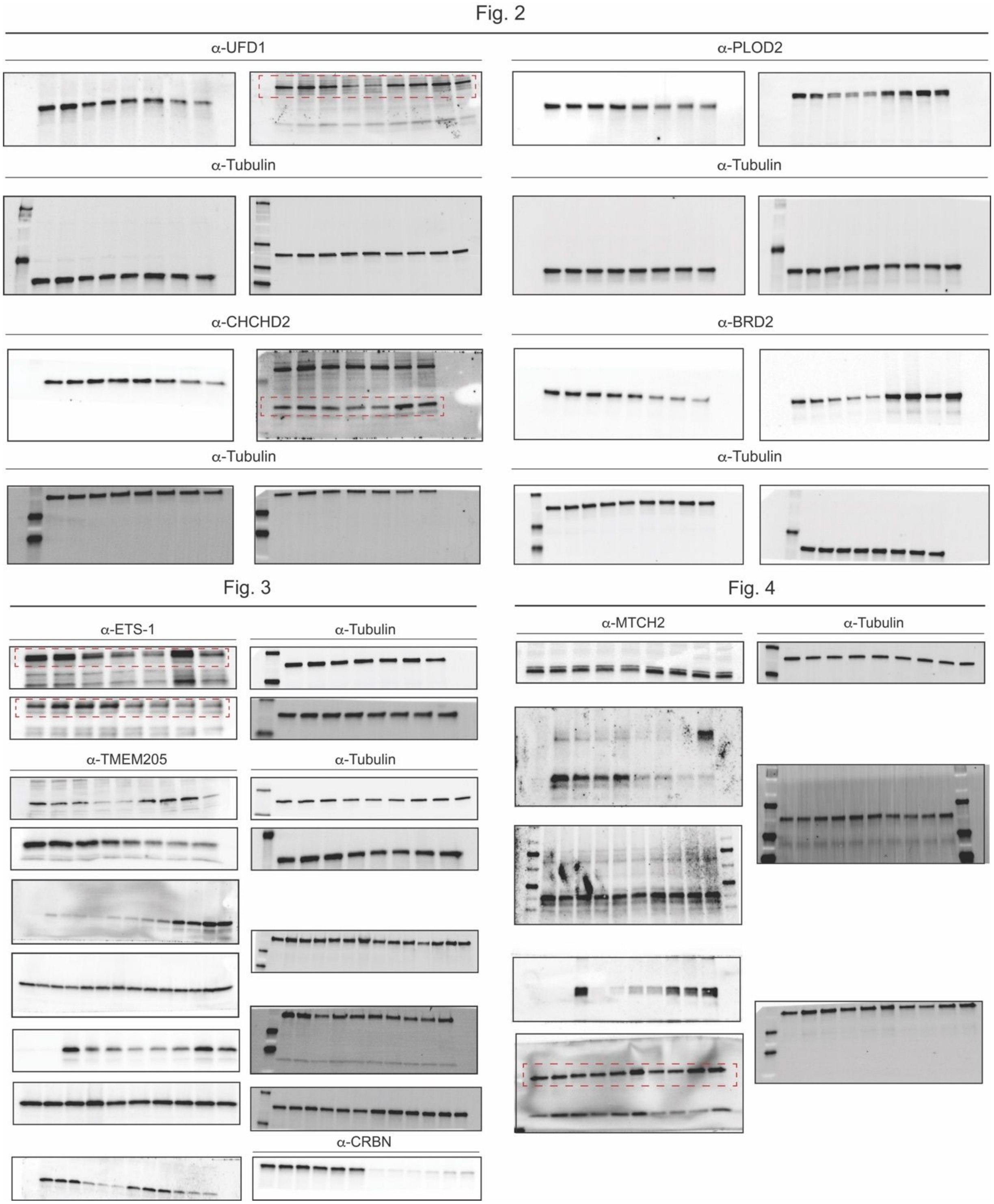

Fig. S20| Compiled replicates and uncropped Western blots (continued on next page)

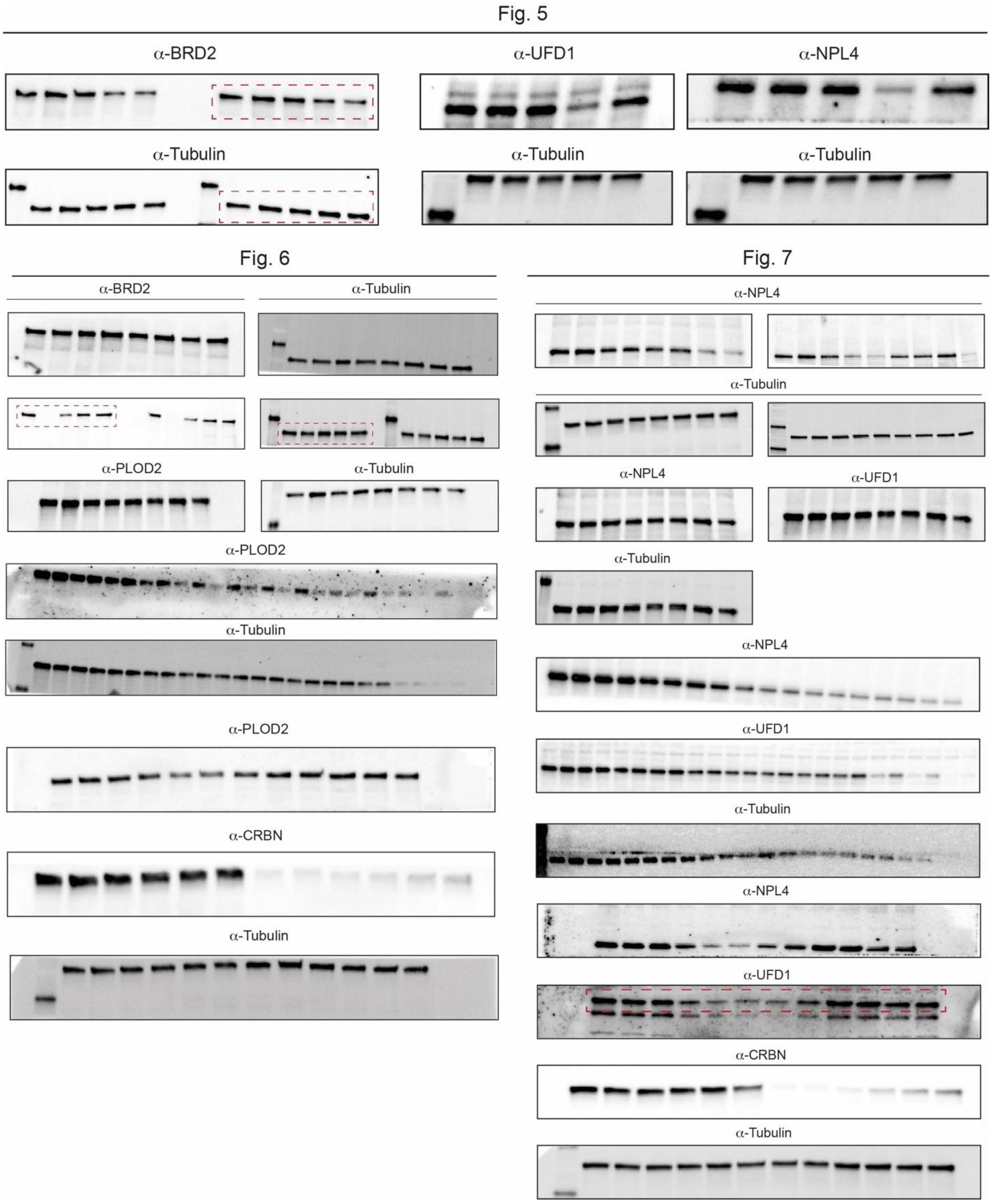

Fig. S20| Compiled replicates and uncropped Western blots **(continued on next page)**

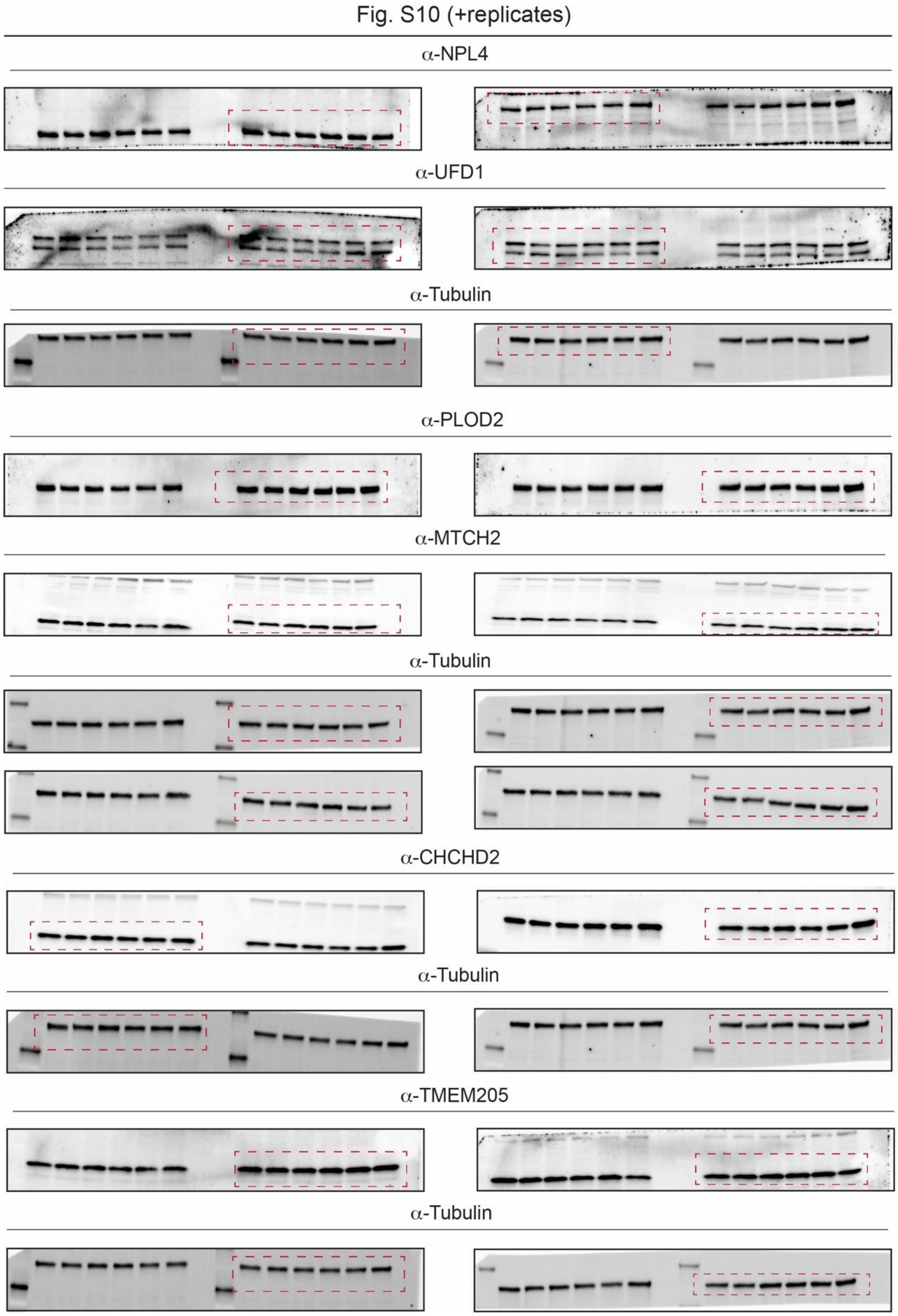

Fig. S20| Compiled replicates and uncropped Western blots (continued on next page)

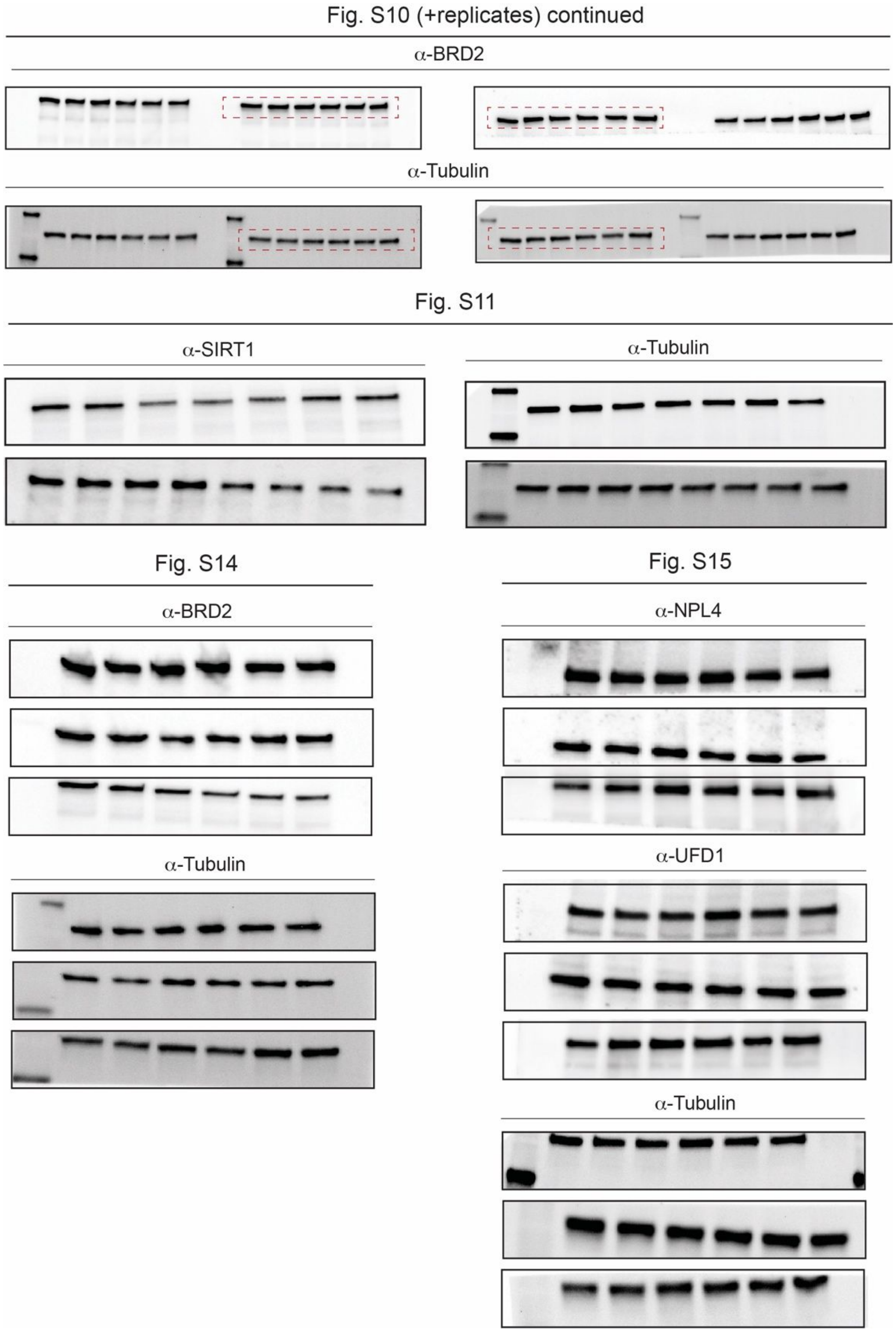

Fig. S20| Compiled replicates and uncropped Western blots (continued on next page)

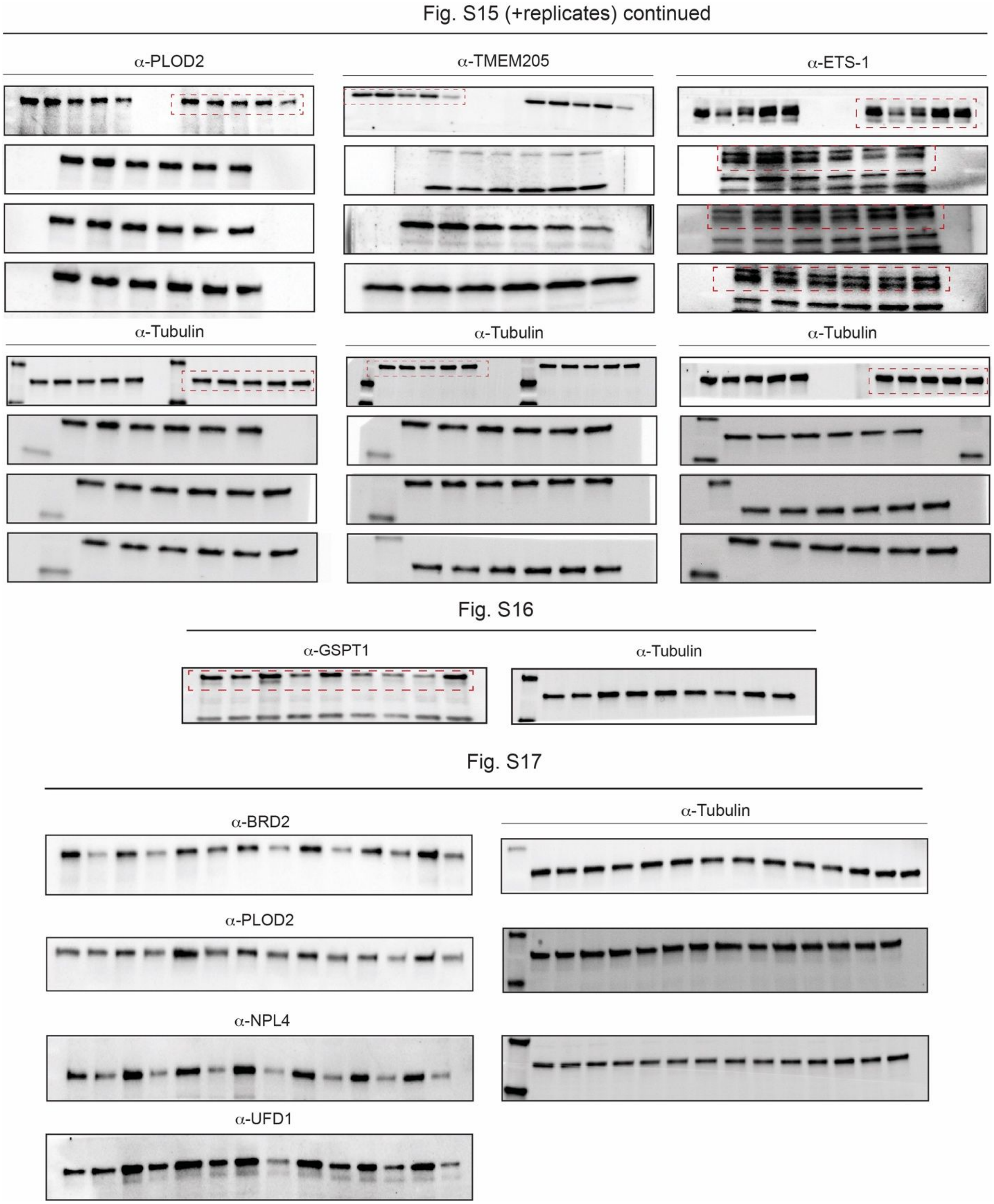

Fig. S20| Compiled replicates and uncropped Western blots (continued on next page)

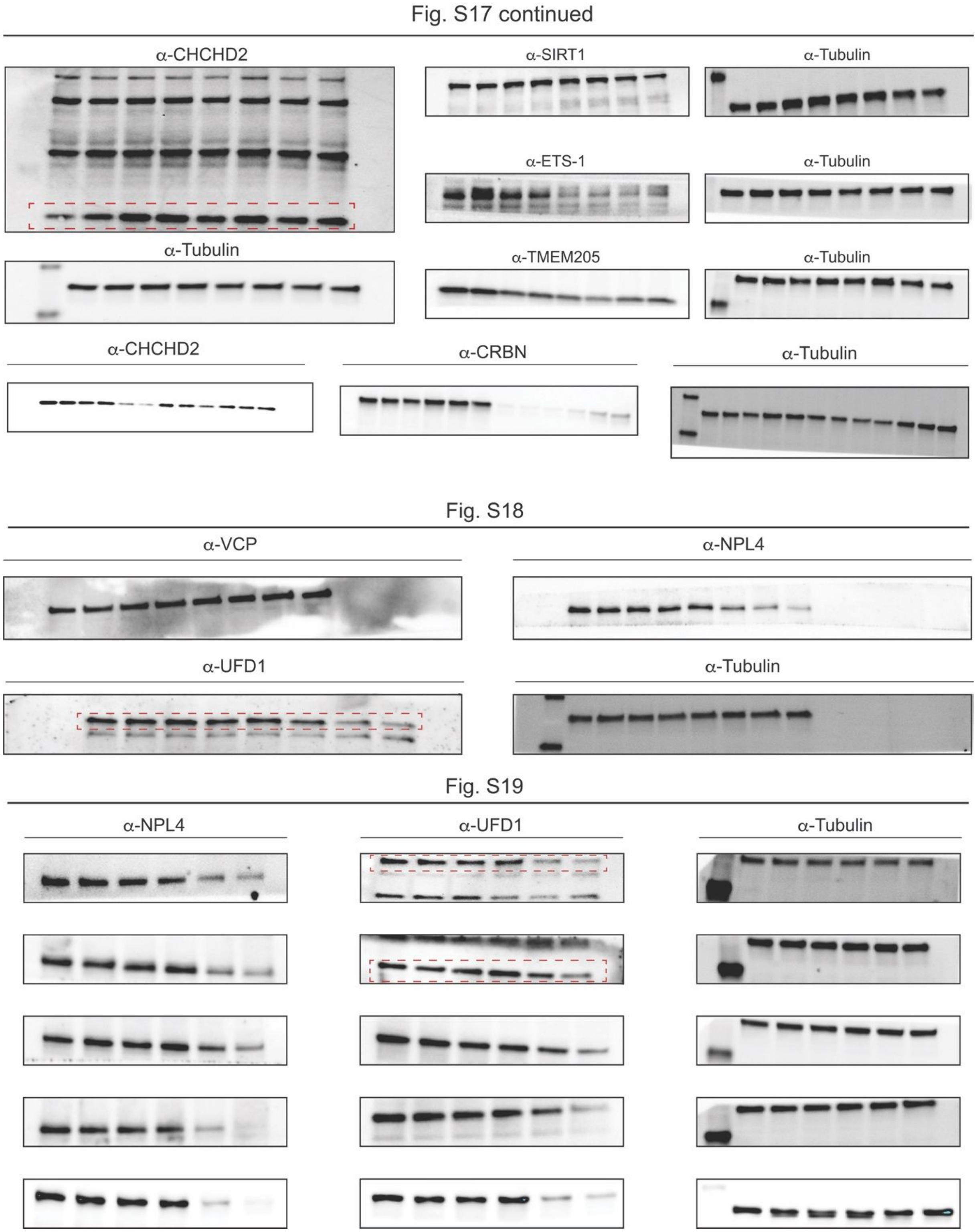

Fig. S20| Compiled replicates and uncropped Western blots (continued on next page)

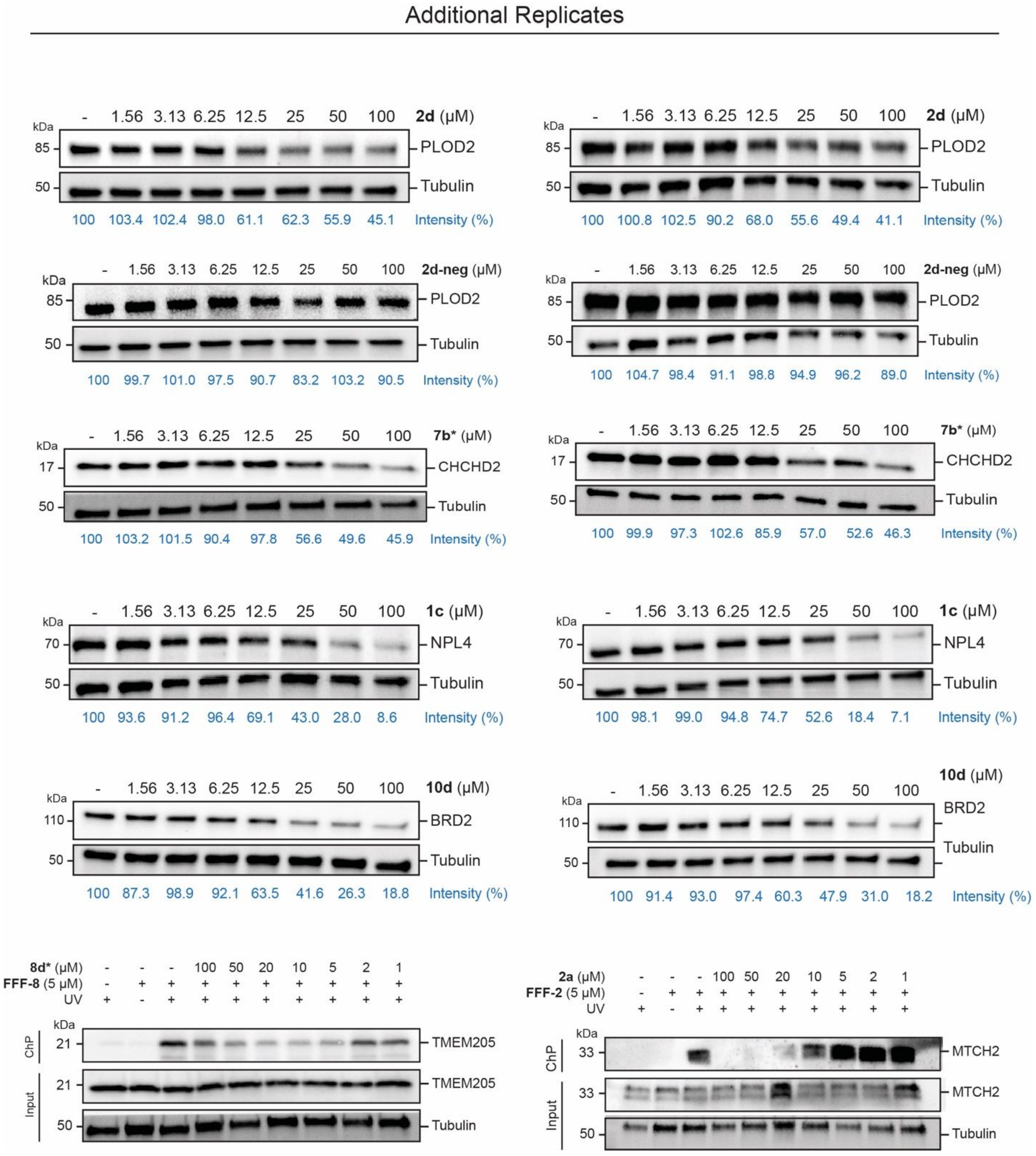

Fig. S20| Compiled replicates and uncropped Western blots. Red insets indicate correct bands displayed in figures.

SUPPLEMENTARY TABLES

	SOURCE	IDENTIFIER
**ANTIBODIES**		
Rabbit anti-UFD1 (1:1,000 dilution),	Cell Signaling Technology	13789S
Rabbit anti-PLOD2 (1:1,000 dilution)	Cell Signaling Technology	44709S
Rabbit anti-CHCHD2 (1:2,000 dilution)	Proteintech	19424-1-AP
Rabbit anti-BRD2 D89B4 clone (1:1,000 dilution)	Cell Signaling Technology	5848S
Rabbit anti-SIRT1 D1D7 clone (1:1,000 dilution)	Cell Signaling Technology	9475S
Rabbit anti-ETS-1 D8O8A clone (1:1,000 dilution)	Cell Signaling Technology	14069S
Rabbit anti-TMEM205 (1:1,000 dilution)	Invitrogen	PA5-59599
Rabbit anti-CCNA2 E6D1J clone (1:1,000 dilution)	Cell Signaling Technology	67955S
Rabbit anti-MTCH2 (1:10,000 dilution)	Proteintech	16888-1-AP
Rabbit anti-NPL4 (1:1,000 dilution)	Cell Signaling Technology	13489S
Rabbit anti-GSPT1 (1:2,000 dilution)	Proteintech	10763-1-AP
Rabbit anti-HA Tag C29F4 clone (1:2,000 dilution)	Cell Signaling Technology	3724S
Anti-tubulin hFAB rhodamine IgG (1:10,000 dilution)	Bio-Rad	12004165
Goat anti-Rabbit IgG, HRP (1:10,000 dilution)	Thermo Fisher Scientific	31460
**EXPERIMENTAL MODELS: CELL LINES**		
MDA-MB-231	ATCC	HTB-26
HEK293t	ATCC	CRL-3216
SH-SY5Y	ATCC	CRL--2266
NCI-H2030	ATCC	CRL-5914
NCI-H23	ATCC	CRL-5800
THP-1	ATCC	TIB-202
**COMMERICAL ASSAYS, KITS AND REAGENTS**		
CellTiter-Glo Luminescent Cell Viability Assay	Promega	G7570
Nano-Glo HiBiT Lytic Detection System	Promega	N3030
DC Protein Assay	Bio-Rad	5000112
Pierce High pH Reversed-Phase Fractionation Kit	Thermo Fisher Scientific	84868
Pierce Quantitative Fluorometric Peptide Assay Kit	Thermo Fisher Scientific	23290
TMT10plexTM Isobaric label reagent set	Thermo Fisher Scientific	90110
Sequencing Grade Modified Trypsin	Promega	V5111
Endoproteinase LysC	New England Biolabs	P8109
Halt Protease Inhibitor	Thermo Fisher Scientific	78438
ProteaseMAX™ Surfactant, Trypsin Enhancer	Promega	V2072
Pierce C18 Spin Columns	Thermo Fisher Scientific	89873
Iodoacetamide (IAA)	Sigma-Aldrich	16125-10G
Tetraethylammonium bromide (TEAB)	Thermo Scientific	9C114
Trifluoroacetic acid (TFA)	Thermo Scientific	85183
Formic acid (FA)	Fisher	A117-50
50% wt. H_2O Hydroxylamine	Sigma-Aldrich	467804
Urea, Molecular Biology Grade	Sigma-Aldrich	U5128
Tris(2-carboxyethyl)phosphine (TCEP)	Research Products International	T26500-10
Potassium carbonate (K_2CO_3)	Sigma-Aldrich	209619
Methanol, Optima LC-MS Grade	Fisher Scientific	A456-4
Chloroform, HPLC-Grade	Fisher Scientific	C607-4
Acetonitrile, Optima LC-MS Grade	Fisher Scientific	A955-4
Water, Optima LC-MS Grade	Fisher Scientific	W64
Calcium chloride ($CaCl_2$), ACS Grade	Sigma-Aldrich	2082904X
4X Laemmli SDS Reducing Sample Buffer	BioWorld	10570020
4-20% Mini-PROTEAN TGX Precast Gels, 10 wells	Bio-Rad	4561093

4-20% Criterion TGX Precast Midi gels, 26 wells	Bio-Rad	5671095
Dulbecco’s phosphate-buffered saline (DBPS)	Corning	21-031-CV
Tween-20	Millipore Sigma	655205
Trans-Blot Turbo RTA transfer Kit, LF, PVDF	Bio-Rad	1704275
(S)-MG132	MedChem Express	HY-13259
Pevonedistat (MLN4924)	MedChem Express	HY-70062
Dulbecco's Modified Eagle Medium (DMEM)	Gibco	11995-065
Roswell Park Memorial Institute (RPMI) Medium	Gibco	21870-076
Opti-MEM™ I Reduced Serum Medium	Gibco	31985070
Fetal Bovine Serum (FBS)	Omega Scientific	FB-01
L-Glutamine 200 mM solution	Corning	25-005-CI
Penicillin/Streptomycin 100X solution	Corning	30-002-CI
**SOFTWARES AND ALGORITHMS**		
Proteome Discoverer (Version 3.0)	Thermo Fisher Scientific	-
Prism (Version 10.1.1)	GraphPad	-
Xcalibur (Version 4.1.50)	Thermo Fisher Scientific	-
ImageLab (Version 6.1.0)	Bio-Rad Laboratories	-
ChemDraw (Version 22.2.0)	Revvity Signals	-
MestReNova (Version 14.0.0)	Mestrelab Research	-
Excel (Version 16.93.1)	Microsoft	-
Python (Version 3.11.3)	Python Software Foundation	-
Illustrator 2025 (Version 29.1)	Adobe	-

Table S1| Reagents, kits, assays, antibodies and softwares used in this study.

SAFETY STATEMENT

No unexpected or unusually high safety hazards were encountered

BIOLOGICAL METHODS

Cell Culture

MDA-MB-231 cells (ATCC) and HEK293t cells (ATCC) were maintained in DMEM supplemented with 10% (v/v) fetal bovine serum (FBS), 1% (v/v) penicillin/streptomycin, and 2mM glutamine. SH-SY5Y cells (gift from the Hyeryun Choe lab) were maintained in DMEM supplemented with 20% (v/v) fetal bovine serum (FBS), 1% (v/v) penicillin/streptomycin, and 2mM glutamine. NCI-H2030 cells (ATCC), NCI-H23 cells (ATCC) and THP-1 cells (ATCC) were maintained in RPMI 1640 supplemented with 10% (v/v) fetal bovine serum (FBS), 1% (v/v) penicillin/streptomycin, and 2mM glutamine. All cells were grown at 37°C in a humidified 5% CO_2 atmosphere.

Live Cell Treatment

Cells were grown to 80-95% confluency in appropriate medium. The growth medium was aspirated, and the cells were incubated with media containing compounds at indicated concentrations and time points. The cultures were scraped, washed with cold DPBS, and collected into 15 mL centrifuge tubes, then transferred to 1.5 mL Eppendorf tubes. The cell suspensions were centrifuged (1,400 g, 3 min) and the pellets were stored at -80 °C until the next stage of processing.

Quantitative Proteomics Sample Preparation

Cell pellets were resuspended in 350 µL DPBS containing 1 x Halt protease inhibitor cocktail, and lysed by sonication (15 ms on, 40 ms off, 15% amplitude, 1 s total). Protein concentrations were normalized (2 mg/mL in 100 µL with cold DPBS) using the Lowry Protein Assay (Pierce). Urea (48 mg) was weighed for each sample in 2 mL LoBind Eppendorf tubes and cell lysates (100 µL = 200 µg) were added (final urea concentration = 8 M). Pellets were resuspended in 50 µL of a freshly prepared 1:1 solution of TCEP (200 mM in DPBS) and K_2CO_3 (600 mM in DPBS) and incubated for 30 min at 37 °C) while shaking. After reaction, 70 µL of a solution of freshly prepared iodoacetamide (70 µL, 400 mM in DPBS) was added and incubated for 30 min at room temperature while protected from light. After reaction, 1.8 mL of cold 3:1:2 MeOH/$CHCl_3$/H_2O solution was added to each tube, and the samples were centrifuged (10,000 x *g,* 10 min, 4 °C), forming a disc. The supernatant was carefully removed, MeOH (600 µL) was added, samples were centrifuged as previously described and the supernatant was removed. Cell pellets were resuspended in TEAB (160 µL, 100 mM, pH 8.5) and sonicated as described previously. Endoproteinase LysC (20 µL, 1/2 vial, 10 µg dissolved in 220 µL of 100 mM TEAB pH 8.5) was added to each and incubated at 37°C for 2 h with shaking. Sequencing-grade modified porcine trypsin (20 µL, 1 vial, 20 µg dissolved in 220 µL of 100 mM TEAB pH 8.5), a solution of Protease Max (2 µL, 1% (w/v) in 100 mM TEAB pH 8.5) and a solution of $CaCl_2$ (2 µL, 100 mM) were added to the samples and incubated at 37 °C overnight (14 h) with shaking. The digest was separated by centrifugation (12,000 *x g*, 10 min, 4 °C). Peptide concentration was determined using Pierce Quantitative Fluorometric Peptide Assay Kit (Thermo Fisher Scientific, 23290) according to manufacturer's instructions. MS-grade acetonitrile (12.5 µL) was added to each tube, and samples were labeled with respective TMT 10 plex or 16-plex isotope (8 µL, 20 µg/ µL) for 1 h with occasional vortexing at RT. To quench, hydroxylamine (3 µL, 5% *v/v*) was added to each sample, vortexed, and incubated for 15 min at RT. Formic acid (5 µL) was added to each tube for acidification, and samples were dried under vacuum centrifugation. The samples were combined by redissolving the contents of one tube in a solution of trifluoroacetic acid (TFA, 400 µL, 0.1% in water) and transferred into each sample until all samples were redissolved. The stepwise process was repeated with formic acid (Buffer A, 200 µL, 0.1% in water) for a final volume of 600 µL. The samples were fractionated using a fractionation kit (Pierce high pH Reversed-Phase Fractionation Kit, Thermo Fisher Scientific 84868) according to manufacturer's instructions. The peptide fractions were eluted from the spin column with

consecutive solutions of 0.1% triethylamine combined with MeCN (5-50% MeCN). The fractions were combined pairwise (fraction 1 and fraction 10, fraction 2 and fraction 11, etc.), dried via vacuum centrifugation, and stored at -80 °C until ready for mass spectrometer injection.

LC-MS Analysis of TMT-Labeled Samples

TMT labeled samples were redissolved in MS buffer A (65 µL, 0.1% formic acid in water). 10 µL of each sample was loaded onto an Acclaim PepMap 100 precolumn (75 µm x 2 mm) and eluted on an Acclaim PepMap RSLC analytical column (75 µm x 15 cm) using the UltiMate 3000 RSLCnano system (Thermo Fisher Scientific). Buffer A was prepared as described above and buffer B (0.1% formic acid in MeCN) were used in a 220 min gradient (flow rate 0.3 mL min, 35 °C) of 2 % buffer B for 10 min, followed by an incremental increase to 30 % buffer B over 192 min, 60 % buffer B for 5 min, 60-95 % buffer B for 1 min, hold at 95 % buffer B for 5 min, followed by descent to 2% buffer B for 1 min followed by re-equilibration at 2 % for 6 min. The elutions were analyzed with a Thermo Fisher Scientific Orbitrap Fusion Lumos mass spectrometer with a cycle time of 3 s and nano-LC electrospray ionization source applied voltage of 2.0 kV. MS^1 spectra were recorded at a resolution of 120,000 with an automatic gain control (AGC) value of $1x10^6$ ions, maximum injection time of 50 ms (dynamic exclusion enabled, repeat count 1, duration 20 s). The scan range was specified from 375 to 1,500 m/z. Peptide fragmentation MS^2 spectra was recorded via collision-induced diffusion (CID) and quadrupole ion trap analysis (AGC $1.8x10^4$, 30 % collision energy, maximum inject time 120 ms, isolation window 1.6). MS3 spectra were generated by high-energy collision-induced dissociation (HCD) with collision energy of 65 %. Precursor selection included up to 10 MS^2 ions for the MS^3 spectrum.

Proteomics Data Processing

Raw MS-data analysis was performed using processing software Proteome Discoverer 3.0 (Thermo Fisher Scientific). Peptide sequences were identified by matching proteome databases with experimental fragmentation patterns via the SEQUEST HT algorithm. Fragment tolerances were set to 0.6 Da, and MS1 precursor mass tolerances set to 10 ppm with one missed cleavage site allowed. Trypsin specificity was designated with a maximum of one missed cleavage. Variable amino acid modifications included methionine oxidation (M, +15.994915) and cysteine carbamidomethylation (C, +57.02146), while TMT-tags (K and N-termini, +229.1629 for 10-plex) were specified as fixed modifications. Spectra were searched against the *Homo Sapiens* proteome database (74,782 sequences) using a false discovery rate (FDR) of 1 % (Percolator). MS^3 peptide quantitation of TMT reporter ions was performed with a mass tolerance of 20 ppm. Relative TMT ratios obtained by Proteome Discoverer were transformed with $log_2(x)$, and statistical significance (p-values) were calculated via Student's two-tailed t-tests across two biological replicates (significance threshold was set at $p < 0.05$).

Cell Viability Assays

Cells were seeded in white-opaque 96-well plates in full growth media at a density of 5,000 cells/well (50 µL) and were allowed to grow for 24 h at 37°C in a humidified 5% CO_2 atmosphere. The cells were then treated with compounds in triplicate and incubated at 37°C in a humidified 5% CO_2 atmosphere for 18 hours. Cell viability was determined using the luciferase-based Cell Titer-Glo Luminescent Cell Viability Assay (Promega) following manufacturer's guidelines. Data represents the average and standard deviation of triplicates in measured luminescence.

Stress Granule Formation Assay

To detect stress granule formation, MDA-MB-231 cells were seeded on 0.01% poly-L-lysine (Sigma, P1274) coated µ-Slide overnight. Cells were then treated with indicated compounds for 6 h, then fixed in 4% PFA (Electron Microscopy Sciences) for 10 min. Fixed cells were washed twice with PBS, permeabilized with 1% TX-100 in PBS (PBST) for 10 min and then blocked with 3% bovine serum albumin (BSA) /PBST for 30 min. Cells were stained overnight at 4 °C with the anti-G3BP1 (1:200;

Proteintech, 66486-1-Ig) in blocking solution. Cells were washed 3 times with PBST and stained for 1 h with CF488A-conjugated Phalloidin (1:1000; Biotium, #00042) and AF555-conjugated anti-mouse secondary antibodies (1:500; Biolegend, 405324) at room temperature. Cells were then washed three times with PBST and counterstained with NucBlue Probe (Thermo Fisher Scientific, R37606). Images were acquired on a confocal microscope (Zeiss LSM 710, Carl Zeiss) and analyzed by ImageJ.

Proteasome and Neddylation Inhibition Experiments

To evaluate the dependence of AgnoTAC-induced protein degradation on the proteasome and neddylation pathways, cells were pre-treated with 10 µM (S)-MG132 (proteasome inhibitor) or 1 µM MLN4924 (NEDD8-activating enzyme inhibitor) for 2 hrs. Following pre-incubation, media was aspirated, and cells were co-treated with inhibitors and the AgnoTAC at indicated time points and concentrations. Cell lysates were then collected and further analyzed by Western blotting.

Immunoblots (Western blots)

After cells were harvested, cell pellets were resuspended in 100 µL DPBS containing 1 x Halt protease inhibitor cocktail, and lysed by sonication (15 ms on, 40 ms off, 15% amplitude, 1 s total). Protein concentrations were normalized (2 mg/mL in 100 µL with cold DPBS) as previously described. 4X SDS gel loading buffer (33 µL) was added to the solution of protein lysate and the resulting mixture was heated at 95 °C for 10 min. Proteins (15 µg total protein loaded per gel lane) were resolved by SDS-PAGE (10 % acrylamide) made in-house, and transferred to PVDF membrane (0.2 µM, 1620177, Bio-Rad). The membrane was blocked with 5% BSA (w/v) in Tris-buffered saline with Tween (TBST) buffer (0.1% Tween 20, 20 mM Tris-HCl 7.6, 150 mM NaCl) at room temperature for 1 h. The antibody was diluted with fresh 5% BSA in TBST buffer (dilutions were performed following manufacturer's guidelines) and incubated with membrane overnight at 4 °C. Membrane was washed three times with TBST buffer, left 5 minutes between each wash on a rocker and then incubated with secondary antibody in 5% dry milk in TBST at room temperature for 2 h on a rocker. Membrane was washed three times with TBST buffer and visualized by in-gel fluorescence on a Bio-Rad ChemiDoc MP Imaging System. The images were processed using Image Lab (version 6.1.0) software.

HiBiT assay

HEK293t cells expressing HiBiT-BRD2 were generated as previously reported[2]. Degradation of HiBiT-BRD2 was quantified using the Nano-Glo HiBiT Lytic Detection System (Promega, #N3030) according to manufacturer protocol. Briefly, $2*10^3$ cells were plated in 30 µL complete medium (DMEM, 4 mM glutamine, supplemented with 10% FBS, penicillin 100 u/mL, and streptomycin 100 µg/mL) in a 384-well plate. Cells were incubated for 24 hrs prior to the experiment (37 °C/5% CO_2). Compounds (20 mM stock in DMSO) were diluted to 3x stocks into complete medium and serially diluted in medium maintaining a constant DMSO concentration (1.5%). 15 µL of these solutions were then added to cells in duplicate and incubated for 18 hrs (37 °C/5% CO_2). After equilibration at room temperature, 40 µL of Nano-Glo HiBiT lysis buffer freshly supplemented with LgBiT (1:100) and Nano-Glo Lytic substrate (1:50) were added to each well. Plate was shaken at 200 rpm for 10 min before imaging using a microplate reader (unfiltered luminescence, ClarioSTAR, BMG Labtech). Signal was normalized to control wells containing no compound as 100% HiBiT-BRD2.

Photoaffinity Probe Cell Treatments

MDA-MB-231 cells were grown to 95% confluency in 15-cm dishes and incubated with fully-functionalized probes or DMSO for 30 min (37 °C, 5% CO_2) in serum-free DMEM. For chemoprecipitation experiments, cells were pre-incubated for AgnoTAC competitor for 30 min, media was aspirated, and cells were co-incubated with AgnoTAC competitor and corresponding photoaffinity probe. Treated cells were irradiated at 4°C under UV light for 20 min using UVP Crosslinker from Analytik Jena equipped with 5x Hitachi F8T5 UV bulbs (365 nm emission, plate lids off) and harvested following previously described methods.

Cellular Thermal Shift Assay (CETSA)

MDA-MB-231 cells ($1*10^7$ cells per milliliter for each condition) were centrifuged (400g, 4 min, 4°C), resuspended in growth medium supplemented with DMSO or indicated concentration of compounds, and incubated for 30 min at 37°C. Sample were then carefully washed with cold DPBS supplemented with 1x Halt protease inhibitor cocktail and compound two times (400g, 4 min, 4°C). Next, 50 μL aliquots were transferred to polymerase chain reaction (PCR) tubes and heated at different temperatures on a Bio-Rad C1000 Thermal Cycler for 3 min and further incubated for 3 min at 25 °C. Each PCR tube underwent three snap freeze–thaw cycles in liquid nitrogen. Lysates were then transferred to 1.5-ml Eppendorf tubes and centrifuged at 20,000g for 20 min at 4 °C. Lastly, 30 μl of soluble protein was transferred to 10 μl of 4× SDS sample buffer-containing PCR tubes, boiled at 95°C for 5 min, and resolved by SDS-PAGE before immunoblot analysis.

Wound Healing Assay

MDA-MB-231 cells were grown to ~60 % confluence in 3 × 6-well plates that were scored on the base with a razor to facilitate image alignment. The cells were treated with **2d** or **2d-neg** (50 μM) in growth medium for 18 h, then scratched with a 200 μl pipette tip. The medium was aspirated to remove debris and replaced with the appropriate solution of compound in growth medium. The cells were imaged (bright field) at 8 hrs and 24 hrs post-scratch. Images were aligned and cropped to a constant size using a custom Python script, and the wound area was quantified using ImageJ with the Wound Healing Tool macro from Montpellier Ressources Imagerie (RRID:SCR_025260; Method: variance, Variance filter radius: 10, Threshold: 200, Radius open: 4, Min. size: 10000). p-Values were calculated using the two-tailed Student's t-test. Protocol was based on ref [3].

Meta-Analysis of Identified Targets

Protein targets were classified as 'liganded' if they were annotated as having approved, non-nutraceutical, small molecule ligands in the DrugBank database[4] (retrieved 20230401). Proteins were classified as enzymes if they were assigned Enzyme Commission numbers in the UniProt database[5] (retrieved 20220516), otherwise their functional classification was based on UniProt keywords adapted from Parker *et al.*[6]. Chemical space plots were generated by applying the UMAP dimension reduction algorithm (0.5.5, arXiv:1802.03426) to 2048-bit molecular fingerprints generated using RDKit .

Chemoinformatic Analysis

Chemoinformatic analysis was performed using Python (3.11.3) and the RDKit library (2022.09.5) to calculate chemoinformatic descriptors. Where the descriptors necessitated the calculation of 3D structures (PBF, NPR1, NPR2), a previous procedure was adapted Ogasawara *et al.*[7]. For each structure, 200 conformers were calculated using the RDKit ETKDG algorithm. These conformers were refined using the RDKit MMFF algorithm, then pruned by iteratively selecting the lowest energy conformer, calculating its RMSD from other conformers and excluding those with RMSD < 1.0 until three conformers remained. The mean of the descriptor values for these three conformers was then calculated.

Putative IMiD-recruiting motif search

Structures of hit proteins were extracted from the AF2 v4 database and inspected for the presence of G-loop motifs, defined as a beta-hairpin turn containing a central Gly residue. The identified G-loops were aligned structurally to the second zinc finger domain of IKZF2 as a template and root-mean-square deviation (RMSD) was computed. Those motifs with an RMSD < 1.00 Å were collected as potential targets of IMiD-class molecular glues.

Quantification and Statistical Analysis

All data fitting and statistical analysis were performed using GraphPad Prism version 10.1.1 for Windows and Mac, www.graphpad.com. Statistical significance was defined as $p < 0.05$ and determined by two-tailed Student's *t* tests.

Data Availability
The mass spectrometry proteomics data have been deposited to the ProteomeXChange Consortium via the PRIDE[8] partner repository with the dataset identifier PXD061964.

REFERENCES

1. Li J, *et al.* Proteome-wide mapping of short-lived proteins in human cells. Mol Cell. **81**, 4722-4735 e4725 (2021).

2. Riching KM, *et al.* Quantitative Live-Cell Kinetic Degradation and Mechanistic Profiling of PROTAC Mode of Action. ACS Chem Biol. **13**, 2758-2770 (2018).

3. Kabala-Dzik A, *et al.* Migration Rate Inhibition of Breast Cancer Cells Treated by Caffeic Acid and Caffeic Acid Phenethyl Ester: An In Vitro Comparison Study. Nutrients. **9**, (2017).

4. Knox C, *et al.* DrugBank 6.0: the DrugBank Knowledgebase for 2024. Nucleic Acids Res. **52**, D1265-D1275 (2024).

5. UniProt C. UniProt: the Universal Protein Knowledgebase in 2025. Nucleic Acids Res. **53**, D609-D617 (2025).

6. Parker CG, *et al.* Ligand and Target Discovery by Fragment-Based Screening in Human Cells. Cell. **168**, 527-541 e529 (2017).

7. Ogasawara D, *et al.* Chemical tools to expand the ligandable proteome: Diversity-oriented synthesis-based photoreactive stereoprobes. Cell Chem Biol. **31**, 2138-2155 e2132 (2024).

8. Perez-Riverol Y, *et al.* The PRIDE database at 20 years: 2025 update. Nucleic Acids Res. **53**, D543-D553 (2025).
