# Supplementary material for: Proteome-Wide Discovery of Degradable Proteins Using Bifunctional Molecules
Source: ACS Cent Sci. 2025 Oct 21;11(11):2240–56. doi: 10.1021/acscentsci.5c01594 (PMC12670307; doi:10.1021/acscentsci.5c01594)

Supplemental Document for

Proteome-Wide Discovery of Degradable Proteins Using Bifunctional Molecules

Ines Forrest, Louis P. Conway, Clara Gathmann, Appaso M. Jadhav, Tzu-Yuan Chiu, Christian M. Chaheine, Michelle Estrada, Anurupa Shrestha, Kathy Sarris, Justin M. Reitsma, Scott E. Warder, Anil Vasudevan, Shaun M. McLoughlin, Christopher G. Parker*

*Corresponding author: Christopher G. Parker, cparker@scripps.edu

SYNTHETIC METHODS AND COMPOUND CHARACTERIZATION

General Synthetic Information

All commercial reagents acquired from commercial vendors, including Sigma-Aldrich, Fisher Scientific, Combi-Blocks, MedChemExpress, eNovation Chemicals, AstaTech, Matrix Scientific, BroadPharm and AmBeed were used without further purification. Distilled water was used for all water necessities in synthetic procedures (e.g., reagent, solvent, work-up). Anhydrous solvents were purchased from Sigma-Aldrich in Sure/Seal™ formulations. All reactions were monitored by thin-layer chromatography (TLC, Merck silica gel 60 F-254 plates) and directly visualized with UV light (254 nm) or stained either with para-anisaldehyde (2.5% p-anisaldehyde, 1% AcOH, 3.5% H_2SO_4 (conc.) in 95% EtOH), iodine, ninhydrin (0.3% ninhydrin (w/v), 97:3 EtOH-AcOH) or KMnO4 (1.5g of KMnO4, 10g K_2CO_3, and 1.25mL 10% NaOH in 200mL water), followed by gentle heating. Reaction purification was carried out using Flash chromatography (230 – 400 mesh silica gel), and Biotage® (manually packed columns of 5G, 10G or 25G of silica) or preparative thin layer chromatography (EMD Millipore silica gel coated (250 µm) F254 glass plates or Analtech glass backed plates (1000-2000 µm thickness). ^{1}H- and ^{13}C-NMR spectra were acquired in the indicated solvents and recorded on a Bruker AVANCE NEO 400 MHz instrument with SampleXpress, Bruker AVANCE NEO 500 MHz NMR instrument equipped with 5mm BBFO SP probe, and SampleCase-24 sample changer, and Bruker AVANCE III HD 600 instrument equipped with a 5mm CPDCH CryoProbe. Data was collected at ambient temperature unless otherwise stated using standard pulse methods as supplied by Bruker software. Coupling constants are quoted to the nearest 0.1 Hz and multiplicities are given by the following abbreviations and combinations: m (multiplet), s (singlet), d (doublet), dd (doublet of doublets), ddd (doublet of doublet of doublets), t (triplet), td (triplet of doublets), tt (triplet of triplets), q (quartet), br (broad). All NMR data was processed in MestReNova v14.0.0. Chemical shifts for proton and carbon resonances are reported in parts per million (ppm) on the δ scale relative to the residual protons of the deuterated solvent of relevance. Mass spectrometry data were collected on a Thermo Scientific ISQ single-quadrupole instrument (ESI; low resolution), Agilent 6125 and 6135 single-quadrupole instruments (ESI; low resolution), and Agilent 6230 single-quadrupole TOF (ESI-TOF; high resolution HRMS).

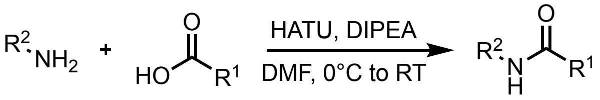

General Procedure 1A: Synthesis of amides using HATU as coupling reagent (in vial/flask)

To a solution of amine (1.1 eq.), carboxylic acid (1.0 eq.) and HATU (1.5 eq.) in dry DMF at 0°C was added DIPEA (3.0 eq.). The reaction mixture was stirred for 2 - 18 h and then quenched with DI H_2O and diluted with Ethyl acetate (EtOAc). The aqueous layer was extracted 3x with EtOAc, and the combined organic layers were washed 2x with H_2O, 2x with a saturated aqueous solution of NH_4Cl and 2x with a saturated aqueous solution of NaCl before being dried over anhydrous Na_2SO_4 and filtered. Volatiles were removed by rotary evaporation and the crude product was purified by automated column chromatography, pTLC, or organic solvent washes (i.e., the organic solvent was added into the vial and mixed with the solid decanting).

General Procedure 1B: Synthesis of amides using HATU as coupling reagent (high-throughput)

In a 96 well plate, stock solutions of amine (HCl or TFA salt, 0.42 M, 1.05 eq.), carboxylic acid (0.40 M, 1.0 eq.) and HATU (0.42 M, 1.05 eq.) were combined. A stock solution of DIPEA (1.4 M, 3.5 eq.) in dry DMF was then added and the reaction mixture was shaken at RT for 6 h. The reaction was diluted with 2x MeCN before purification by preparative HPLC (MeCN/H_2O gradient, 0.1% FA), followed by pTLC if necessary.

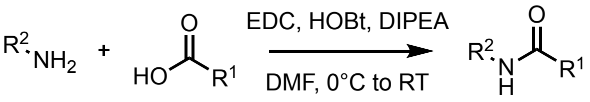

General Procedure 2: Synthesis of amides using EDC as coupling reagent

To a solution of amine (1.1 eq.), carboxylic acid (1.0 eq.), EDC (1.5 eq.) and HOBt (1.5 eq.) in dry CH_2Cl_2 (DCM) at 0°C was added DIPEA (3.0 eq.). The reaction mixture was stirred overnight, then quenched with deionized H_2O. The aqueous layer was extracted 3x with CH_2Cl_2, and the combined organic layers were washed 2x with H_2O, 2x with a saturated aqueous solution of NH_4Cl and 2x with a saturated aqueous solution of NaCl before being dried over anhydrous Na_2SO_4 and filtered. Volatiles were removed by rotary evaporation and the crude product was purified by automated column chromatography, pTLC, or organic solvent washes (i.e., the organic solvent was added into the vial and mixed with the solid decanting).

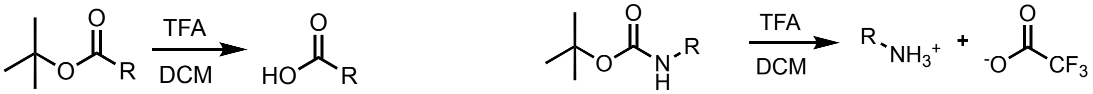

General Procedure 3: *Tert*-butyl ester deprotection of carboxylic acids and Boc deprotection of amines using TFA

To a solution of t-Butyl protected acid (left, 1.0 eq.) or Boc protected amine (right, 1.0 eq.) in dry CH_2Cl_2 (DCM) was added a solution of TFA (20-50%). The reaction mixture was stirred at RT for 0.5 - 12 h. The crude product was concentrated through a nitrogen flow, diluted in CH_2Cl_2 and evaporated again. No purification was needed.

General Procedure 4: Methylated negative control

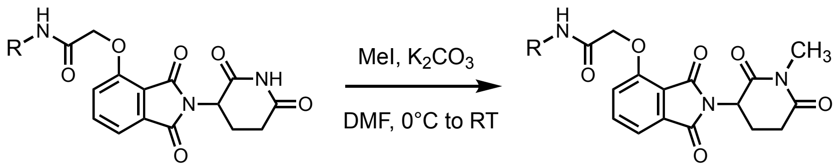

To a solution of selected bifunctional molecule (1.0 eq) in DMF (1 mL) at 0°C, was added potassium carbonate (K_2CO_3, 1.2 eq) and methyl iodide (MeI, 1.2 eq). The reaction mixture was stirred at RT for 0.5 – 1 h, then quenched with cold with H_2O and extracted with EtoAC (2 × 50 mL). The combined organic extracts were dried over anhydrous Na_2SO_4, filtered, and concentrated under reduced pressure. The crude product was purified by pTLC or Biotage® using a 0–6% methanol gradient in CH_2Cl_2 (DCM) to afford the corresponding methylated negative control.

Compound Characterization

Thalidomide-linker intermediates

Thalidomide-O-C2-acid, thalidomide-O-C2-amine, thalidomide-O-acetamido-C5-acid, thalidomide-O-acetamido-C5-amine, thalidomide-O-C5-acid, thalidomide-O-acetamido-PEG2-C2-acid, thalidomide-O-acetamido-PEG2-C2-amine, thalidomide-O-PEG2-acid, thalidomide-O-acetamido-PEG3-C2-acid, thalidomide-O-acetamido-PEG3-C2-amine and thalidomide-O-PEG3-C2-acid starting materials were purchased from BroadPharm and Tenova Pharmaceuticals Inc. and used as received without purification.

Headgroup intermediates

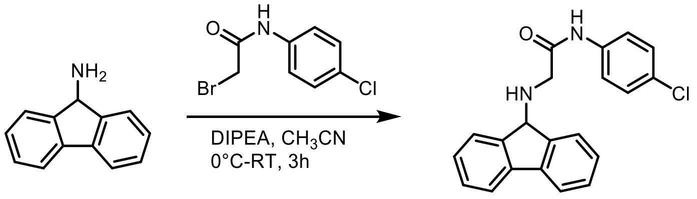

F2

Fragment F2: 2-((9H-fluoren-9-yl)amino)-N-(4-chlorophenyl)acetamide (F2)

To a solution of 9H-Fluoren-9-amine hydrochloride 88 mg (40.4 µmol, 1 eq) and 2-bromo-N-(4-chlorophenyl)acetamide 100 mg (40.4 µmol, 1 eq) in acetonitrile (5 mL), DIPEA (120.4 µmol, 3 eq) were added at 0°C. The reaction mixture was stirred at RT for 3 h. The reaction mixture was then diluted with cold water and extracted with ethyl acetate. The organic layer was dried over anhydrous Na_2SO_4, filtered, and concentrated under reduced pressure. The crude product was purified by Biotage® using a 0–60% ethyl acetate in hexane gradient to afford the desired product (**F2**) as a white powder (125 mg, 35.3 µmol, 65%). ^{1}H NMR (600 MHz, $CDCl_3$) δ 9.42 (s, 1H), 7.74 (d, J = 7.6 Hz, 2H), 7.65 (d, J = 7.4 Hz, 2H), 7.56 – 7.50 (m, 2H), 7.46 – 7.42 (m, 2H), 7.36 – 7.34 (m, 2H), 7.32 – 7.29 (m, 2H), 5.08 (s, 1H), 3.24 (s, 2H). ^{13}C NMR (151 MHz, CDCl3) δ 169.60, 143.83, 140.96, 136.07, 129.17, 129.11, 129.07, 128.88, 127.71, 124.65, 120.67, 120.29, 63.17, 48.50. LC-MS (ESI+) calc'd for $C_{21}H_{17}ClN_2O10$: 348.10, found $[M+H]^+$ 349.10.

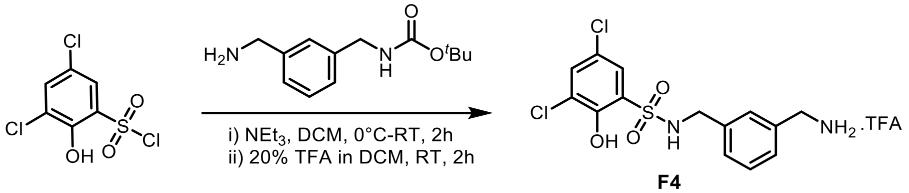

F4

Fragment F4: **N-(3-(aminomethyl)benzyl)-3,5-dichloro-2-hydroxybenzenesulfonamide (F4)**

To a solution of tert-Butyl 3-(aminomethyl)benzylcarbamate 300 mg (1.27 µmol, 1 eq) and 3,5-Dichloro-2-hydroxybenzenesulfonyl chloride 332 mg (1.27 µmol, 1 eq) in dichloromethane (5 mL), triethylamine 257 mg (2.54 µmol, 2 eq) were added at 0°C. The reaction mixture was stirred at RT for 2 h. The reaction mixture was then diluted with cold water and extracted with dichloromethane. The organic layer was dried over anhydrous Na_2SO_4, filtered, and concentrated under reduced pressure. The crude product was purified by Biotage® using a 0–80% ethyl acetate in hexane gradient to afford the Boc intermediate, which was subjected to deprotection as described in general procedure 3 to obtain N-(3-(aminomethyl)benzyl)-3,5-dichloro-2-hydroxybenzenesulfonamide (**F4**) as TFA salt which was used without further purification. (470 mg, 72 %) ^{1}H NMR (600 MHz, DMSO-d6) δ 10.97 (s, 1H), 8.24 – 8.19 (m, 2H), 7.82 – 7.72 (m, 1H), 7.53 (d, *J* = 2.6 Hz, 1H), 7.33 – 7.28 (m, 3H), 4.13 (s, 2H), 3.97 (s, 2H). LC-MS (ESI+) calc'd for $C_{14}H_{14}Cl_2N_2O_3S$: 360.01, found $[M+H]^+$ 361.01.

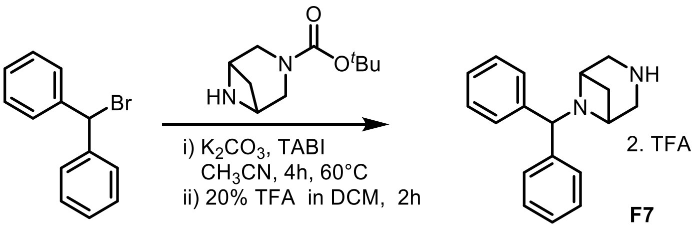

F7

Fragment F7: **2-benzhydryl-2,5-diazabicyclo[2.2.1]heptane (F7)**

To a stirred solution of bromodiphenylmethane 100 mg (0.40 µmol, 1 eq) in acetonitrile (5 mL) was added tert-Butyl 3,6-diazabicyclo[3.1.1]heptane-3-carboxylate 88 mg (0.44 µmol, 1.2 eq), K_2CO_3 167 mg (1.21 µmol, 3 eq) and TABI 15 mg (0.04 µmol, 0.1 eq). The mixture was stirred for 4h at 60 °C, then cooled to room temperature, diluted with water, and extracted in ethyl acetate (50 mL X2). The combined extract was dried over Na_2SO_4, filtered, and concentrated. The crude product was purified by Biotage® using a 0–50% Ethyl acetate in Hexane gradient to afford tert-butyl-5-benzhydryl-2,5-diazabicyclo[2.2.1]heptane-2-carboxylate intermediate was subjected to deprotection as described in general procedure 3 to obtain 2-benzhydryl-2,5-diazabicyclo[2.2.1]heptane (**F7**) as TFA salt which was used without further purification. (150 mg, 67 %) ^{1}H NMR (600 MHz, CD_3OD) δ 7.65 (d, J = 7.7 Hz, 4H), 7.44 (d, J = 7.6 Hz, 4H), 7.38 (d, J = 7.1 Hz, 2H), 5.88 (s, 1H), 4.18 – 4.12 (m, 2H), 4.10 – 4.02 (m, 2H), 3.74 (s, 2H), 3.61 – 3.38 (m, 1H), 2.18 (s, 1H). LC-MS (ESI+) calc'd for $C_{18}H_{20}N_2$: 264.1, found $[M+H]^+$ 265.1.

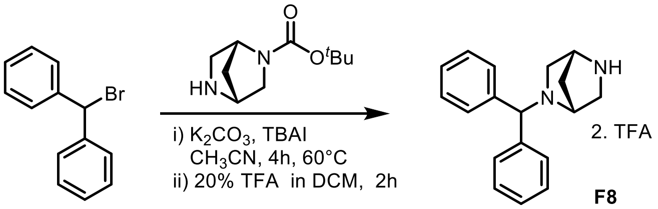

F8

Fragment F8: **(1R,4R)-2-benzhydryl-2,5-diazabicyclo[2.2.1]heptane (F8)**

To a stirred solution of bromodiphenylmethane 100 mg (0.40 µmol, 1 eq) in acetonitrile (5 mL) was added (*1R*, *4R*)-diazabicyclo[2,2,1]-heptane 88 mg (0.44 µmol, 1.2 eq), K_2CO_3 167 mg (1.21 µmol, 3 eq) and TABI 15 mg (0.04 µmol, 0.1 eq). The mixture was stirred for 4h at 60 °C, then cooled to room temperature, diluted with water, and extracted in ethyl acetate (50 mL x 2). The combined extract was dried over Na_2SO_4, filtered, and concentrated. The crude product was purified by Biotage® using a 0–50% Ethyl acetate in Hexane gradient to afford the tert-butyl (*1R,4R*)-5-benzhydryl-2,5-diazabicyclo[2.2.1]heptane-2-carboxylate intermediate which was subjected for deprotection as a general procedure 3 to obtain (1R,4R)-2-benzhydryl-2,5-diazabicyclo[2.2.1]heptane **(F8)** as TFA salt which was used without further purification. (112 mg, 56 %) ^{1}H NMR (600 MHz, CD_3OD) δ 7.61 – 7.51 (m, 4H), 7.38 – 7.32 (m, 4H), 7.32 – 7.23 (m, 2H), 4.95 (s, 1H), 4.32 (s, 1H), 3.89 – 3.59 (m, 2H), 3.22 – 3.18 (m, 1H), 3.02 – 2.98 (m, 2H), 2.45 – 2.43 (m, 1H), 1.91 – 1.89 (m, 1H). LC-MS (ESI+) calc'd for $C_{18}H_{20}N_2$: 264.1, found $[M+H]^+$ 265.1.

Propylated Headgroups

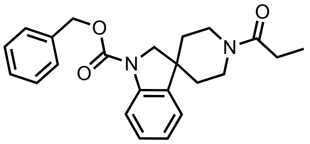

F01-Pr

Benzyl 1'-propionylspiro[indoline-3,4'-piperidine]-1-carboxylate (F01-Pr)

To a solution of benzyl spiro[indoline-3,4'-piperidine]-1-carboxylate hydrogen chloride **F1** (19 mg, 1.0 eq, 53 μmol) and triethylamine (21 mg, 30 μL, 4.0 eq, 0.21 mmol) in DCM (0.2 mL) was added a solution of propionyl chloride (12 mg, 12 μL, 2.5 eq, 0.13 mmol). The solution was stirred for 2h at room temperature, showing completion by TLC. The solvents were removed under reduced pressure and the residue was filtered over silica (100% EtOAc) to yield amide **F01-Pr** as a transparent wax (19.5 mg, 51.5 μmol, 97 % yield). ^{1}H NMR (400 MHz, MeOD) δ 7.82 (br s, 1H), 7.51 – 7.43 (m, 2H), 7.42 – 7.30 (m, 3H), 7.23 – 7.15 (m, 2H), 7.00 (t, *J* = 7.5 Hz, 1H), 5.27 (br s, 2H), 4.55 (d, *J* = 13.7 Hz, 1H), 4.02 (d, *J* = 3.3 Hz, 2H), 3.96 (d, *J* = 14.6 Hz, 1H), 3.23 (t, *J* = 13.6 Hz, 1H), 2.79 (t, *J* = 13.5 Hz, 1H), 2.46 (q, *J* = 7.6 Hz, 2H), 1.95 – 1.61 (m, 4H), 1.14 (t, *J* = 7.5 Hz, 3H). ^{13}C NMR (151 MHz, MeOD) δ 173.39, 153.04, 141.47, 138.15, 136.38, 128.27, 127.97, 127.92, 127.88, 122.92, 122.48, 114.43, 66.91, 56.76, 42.52, 38.66, 36.88, 36.10, 25.93, 8.60. *Note: Signals near carbamate have reduced intensity due to conformational exchange.* HR-MS (ESI+) calc'd for $[M+H]^+$ $C_{23}H_{27}N_2O_3$: 379.2016, found 379.2024 (2.2 ppm).

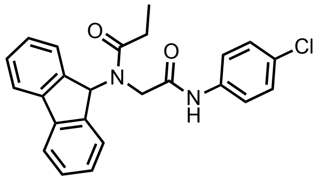

F02-Pr

***N*-(2-((4-chlorophenyl)amino)-2-oxoethyl)-*N*-(9*H*-fluoren-9-yl)propionamide (F02-Pr)**

To a solution of 2-((9*H*-fluoren-9-yl)amino)-*N*-(4-chlorophenyl)acetamide **F2** (27.7 mg, 1.0 eq, 80 μmol) and triethylamine (34 μL, 0.24 mmol) in DCM (3 mL) was added a solution of propionyl chloride (8.2 mg, 7.7 μL, 1.1 eq, 87 μmol). The solution was stirred for 2h at room temperature, showing completion by TLC. Solvents were removed under reduced pressure and the residue was filtered over silica (100% EtOAc) to yield amide **F02-Pr** as a white solid (30.6 mg, 76 μmol, 95 % yield). ^{1}H NMR (600 MHz, $CDCl_3$) δ 8.65 (s, 1H), 7.74 (s, 1H), 7.69 (d, *J* = 7.6 Hz, 1H), 7.50 (d, *J* = 7.5 Hz, 1H), 7.44 (s, 1H), 7.38 (d, *J* = 7.5 Hz, 2H), 7.28 (d, *J* = 4.1 Hz, 1H), 7.25 – 7.22 (m, 2H), 7.20 (d, *J* = 2.5 Hz, 2H), 7.09 (d, *J* = 8.4 Hz, 1H), 5.94 (s, 1H), 3.41 (d, *J* = 3.4 Hz, 2H), 2.93 (q, *J* = 7.4 Hz, 2H), 1.42 (t, *J* = 7.4 Hz, 3H). ^{13}C NMR (151 MHz, $CDCl_3$) δ 176.83, 167.62, 142.23, 141.67, 141.21, 140.89, 136.81, 130.00, 129.16, 128.20, 125.93, 124.96, 124.22, 121.47, 121.31, 121.11, 120.78, 77.59, 77.37, 77.16, 63.48, 59.78, 48.76, 27.62, 27.44, 10.42, 9.85. HR-MS (ESI+) calc'd for $[M+H]^+$ $C_{24}H_{21}ClN_2O_2$: 405.1325, found 405.1328.

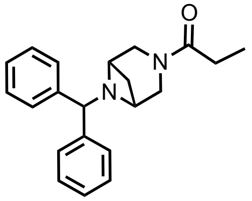

F07-Pr

1-(6-benzhydryl-3,6-diazabicyclo[3.1.1]heptan-3-yl)propan-1-one (F07-Pr)

To a solution of 6-benzhydryl-3,6-diazabicyclo[3.1.1]heptane trifluoroacetate **F7** (13.7 mg, 1.0 eq, 28 µmol) and triethylamine (11.3 mg, 15.5 µL, 4.0 eq, 111 µmol) in DCM (0.2 mL) was added a solution of propionyl chloride (6.4 mg, 6.0 µL, 2.5 eq, 70 µmol). The solution was stirred for 2h at room temperature, showing completion by TLC. The solvents were removed under reduced pressure and the residue was purified by preparative TLC (1% MeOH, 70 mM NH_3 in DCM) to yield amide **F07-Pr** as a white solid (7.5 mg, 23 µmol, 84 % yield).^{1}H NMR (400 MHz, $CDCl_3$) δ 7.56 – 7.43 (m, 4H), 7.31 – 7.21 (m, 4H), 7.21 – 7.09 (m, 2H), 4.66 (s, 1H), 3.70 – 3.51 (m, 4H), 3.45 (d, *J* = 13.8 Hz, 1H), 3.38 – 3.26 (m, 1H), 2.66 (q, *J* = 6.2 Hz, 1H), 2.35 (q, *J* = 7.4 Hz, 2H), 1.44 (d, *J* = 8.7 Hz, 1H), 1.22 (t, *J* = 7.4 Hz, 3H).^{13}C NMR (151 MHz, $CDCl_3$) δ 175.02, 142.06, 141.82, 128.83, 128.71, 127.50, 127.36, 64.91, 56.40, 56.13, 44.50, 42.81, 28.90, 26.71, 9.12. HR-MS (ESI+) calc'd for $[M+H]^+$ $C_{21}H_{25}N_2O$: 321.1962, found 321.1966 (1.3 ppm).

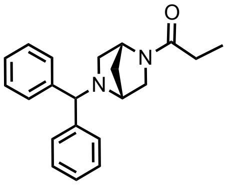

F08-Pr

1-((1*R*,4*R*)-5-benzhydryl-2,5-diazabicyclo[2.2.1]heptan-2-yl)propan-1-one (F08-Pr)

To a solution of (1R,4R)-2-benzhydryl-2,5-diazabicyclo[2.2.1]heptane trifluoroacetate **F8** (15.2 mg, 1.0 eq, 30.9 µmol) and triethylamine (12.5 mg, 17.2 µL, 4.0 eq, 123 µmol) in DCM (0.2 mL) was added a solution of propionyl chloride (7.14 mg, 6.71 µL, 2.5 eq, 77.2 µmol). The solution was stirred for 2h at room temperature, showing completion by TLC. The solvents were removed under reduced pressure and the residue was purified by preparative TLC (1% MeOH, 70 mM NH_3 in DCM) to yield amide **F08-Pr** as a transparent wax (8.1 mg, 25 µmol, 82 % yield). ^{1}H NMR (400 MHz, DMSO, 100°C) δ 7.48 (d, *J* = 7.6 Hz, 4H), 7.27 (t, *J* = 7.5 Hz, 4H), 7.16 (t, *J* = 7.3 Hz, 2H), 4.69 (s, 1H), 4.46 (s, 1H), 3.57 (d, *J* = 9.6 Hz, 1H), 3.39 (s, 1H), 3.17 (br s, 1H), 2.78 – 2.73 (m, 1H), 2.44 (d, *J* = 9.7 Hz, 1H), 2.34 – 2.12 (m, 2H), 1.93 (d, *J* = 9.7 Hz, 1H), 1.61 (d, *J* = 9.6 Hz, 1H), 1.05 (t, *J* = 7.5 Hz, 3H). *Note*: *At room temperature, a ~ 1:1 distribution of two conformers is seen. Signal pairs (mostly close to piperazine ring) are reported together when obvious.* ^{13}C NMR (151 MHz, DMSO) δ 170.26 & 170.16, 144.50 & 144.47, 143.60, 128.52, 127.53, 127.09 & 127.06, 126.92 & 126.84, 71.14 & 70.97, 59.83, 58.91, 58.78, 57.95, 57.79, 55.48, 49.76 & 49.18, 35.52 & 34.27, 26.76 & 26.14, 9.24 & 8.87. HR-MS (ESI+) calc'd for $[M+H]^+$ $C_{21}H_{25}N_2O$: 321.1962, found 321.1969 (2.2 ppm).

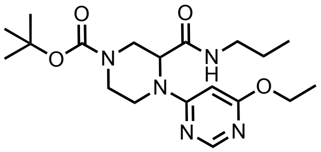

F10-Pr

***tert*-butyl 4-(6-ethoxypyrimidin-4-yl)-3-(propylcarbamoyl)piperazine-1-carboxylate (F10-Pr)**

To a solution of 4-(tert-butoxycarbonyl)-1-(6-ethoxypyrimidin-4-yl)piperazine-2-carboxylic acid (7.2 mg, 1.0 eq, 20 µmol) and HATU (16 mg, 2.0 eq, 41 µmol) in DMF at 0°C was added DIPEA (7.9 mg, 11 µL, 3.0 eq, 61 µmol) and then propan-1-amine (2.4 mg, 3.4 µL, 2.0 eq, 41 µmol). The solution was warmed up to room temperature and stirred for 5h, showing completion by LC-MS. The solvents were removed, and the residue was purified by flash chromatography (C18, 50-60% MeCN in H_2O, 0.1% TFA) and preparative TLC (7% MeOH in DCM) to afford amide **F10-Pr** as a white solid (7.2 mg, 18 µmol, 90 % yield). ^{1}H NMR (400 MHz, $CDCl_3$) δ 8.32 (s, 1H), 6.29 (d, *J* = 79.4 Hz, 1H), 5.83 (s, 1H), 5.18 – 4.86 (br m, 1H), 4.58 – 4.43 (br m, 1H), 4.36 (q, *J* = 7.1 Hz, 2H), 3.89 – 3.70 (m, 2H), 3.43 (br s, 1H), 3.43 – 3.28 (m, 2H), 3.28 – 3.08 (m, 2H), 1.54 – 1.42 (m, 2H), 1.46 (s, 9H), 1.36 (t, *J* = 7.1 Hz, 3H), 0.85 (t, *J* = 7.5 Hz, 3H). *Note*: *Conformational exchange visible on piperazine signals (broad peaks with reduced intensity).* ^{13}C NMR (151 MHz, $CDCl_3$) δ 170.58, 169.69, 164.10 & 163.76 (broad, rot.), 157.70, 154.90, 86.58, 80.58, 62.44, 56.09, 43.32 (br), 42.85 (br), 41.80 (br), 41.45, 28.48, 22.92, 14.73, 11.43. *Note*: *Conformational exchange visible on piperazine signals (broad peaks with reduced intensity).* HR-MS (ESI+) calc'd for $[M+H]^+$ $C_{19}H_{32}N_5O_4$: 394.2449, found 394.2458 (2.3 ppm).

Fully-Functionalized Photoaffinity Probes

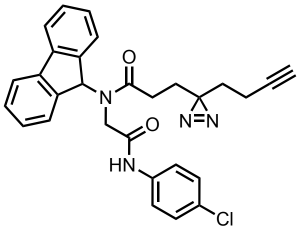

FFF-2

3-(3-(but-3-yn-1-yl)-3*H*-diazirin-3-yl)-*N*-(2-((4-chlorophenyl)amino)-2-oxoethyl)-*N*-(9*H*-fluoren-9-yl)propenamide (FFF-2)

To a solution of 2-((9*H*-fluoren-9-yl)amino)-*N*-(4-chlorophenyl)acetamide **F2** (20 mg, 1.0 eq, 57.3 µmol) and HATU (24 mg, 1.1 eq, 63 µmol) in DMF (1 mL) at 0˚C was added DIPEA (22 mg, 30 µL, 3.0 eq, 0.17 mmol) and then 3-(3-(but-3-yn-1-yl)-3*H*-diazirin-3-yl)propanoic acid (11 mg, 1.1 eq, 63.1 µmol). The solution was warmed up to room temperature and stirred for 2h. The solvents were removed, and the residue was purified by preparative TLC (7% MeOH in DCM) to afford amide **FFF-2** as a sticky yellow solid (17.8 mg, 35.8 µmol, 63% yield). ^{1}H NMR (600 MHz, CDCl3) δ 8.45 (d, *J* = 4.5 Hz, 1H), 7.72 (dd, *J* = 7.8, 3.8 Hz, 2H), 7.66 (t, *J* = 7.9 Hz, 1H), 7.49 (d, *J* = 4.8 Hz, 1H), 7.44 – 7.41 (m, 2H), 7.38 (d, *J* = 7.5 Hz, 2H), 7.27 (d, *J* = 3.9 Hz, 2H), 7.17 (dt, *J* = 4.3, 2.7 Hz, 2H), 5.81 (d, *J* = 3.8 Hz, 1H), 3.38 (d, *J* = 3.5 Hz, 1H), 3.34 – 3.30 (m, 1H), 2.56 (td, *J* = 6.9, 2.8 Hz, 2H), 2.21 (dd, *J* = 5.6, 3.4 Hz, 2H), 2.08 – 2.06 (m, 2H), 1.97 – 1.96 (m, 1H), 1.76 (dt, *J* = 7.4, 4.0 Hz, 2H). ^{13}C NMR (151 MHz, CDCl3) δ 173.68, 167.23, 141.95, 141.59, 141.17, 140.57, 136.68, 130.06, 129.52, 129.22, 129.12, 128.26, 126.04, 125.06, 121.56, 121.37, 121.09, 120.64, 82.98, 69.75, 63.32, 48.53, 33.13, 28.65, 28.58, 28.13, 27.90, 27.59, 13.70. HR-MS (ESI+) calc'd for $[M+H]^+$ $C_{29}H_{25}ClN_4O_2$: 497.1698, found 497.1704.

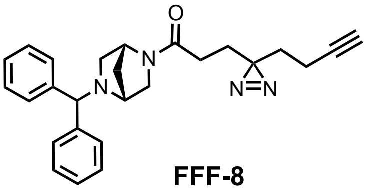

FFF-8

1-((1*R*,4*R*)-5-benzhydryl-2,5-diazabicyclo[2.2.1]heptan-2-yl)-3-(3-(but-3-yn-1-yl)-3*H*-diazirin-3-yl)propan-1-one (FFF-8)

To a solution of (1*R*,4*R*)-2-benzhydryl-2,5-diazabicyclo[2.2.1]heptane **F8** (15 mg, 1.0 eq, 33 µmol), EDC (10 mg, 1.5 eq, 49 µmol) and HOBt (8 mg, 1.5 eq, 49 µmol) in DMF (1 mL) at 0˚C was added DIPEA (34 µL, 6.0 eq, 0.2 mmol) and then 3-(3-(but-3-yn-1-yl)-3*H*-diazirin-3-yl)propanoic acid (6 mg, 1.0 eq, 33 µmol). The solution was stirred at room temperature for 5h. The solvents were removed, and the residue was purified by preparative TLC (5% MeOH in DCM) to afford amide **FFF-8** as a transparent wax (14.9 mg, 36.1 µmol, 64% yield). ^{1}H NMR (600 MHz, CDCl3) δ 7.45 (td, *J* = 4.1, 1.7 Hz, 5H), 7.27 (d, *J* = 2.0 Hz, 1H), 7.25 (d, *J* = 1.9 Hz, 1H), 7.17 (dd, *J* = 7.5, 1.6 Hz, 3H), 4.72 (s, 1H), 4.57 (s, 1H), 4.17 (s, 1H), 3.77 (dd, *J* = 11.5, 1.8 Hz, 1H), 3.59 – 3.55 (m, 1H), 3.48 (d, *J* = 16.2 Hz, 2H), 3.15 (td, *J* = 10.0, 2.3 Hz, 2H), 2.93 (dd, *J* = 10.1, 2.2 Hz, 1H), 2.75 (dd, *J* = 10.2, 2.3 Hz, 1H), 2.59 (d, *J* = 10.2 Hz, 1H), 2.40 (dd, *J* = 10.0, 1.6 Hz, 1H), 2.07 – 2.00 (m, 5H), 1.96 – 1.92 (m, 4H), 1.90 – 1.85 (m, 3H), 1.70 – 1.66 (m, 3H), 1.59 (d, *J* = 9.8 Hz, 1H). ^{13}C NMR (151 MHz, CDCl3) δ 169.05, 144.13, 143.38, 128.96, 127.98, 127.61, 127.54, 127.45, 83.15, 73.15, 72.27, 69.50, 59.94, 59.48, 59.12, 58.28, 56.76, 51.48, 49.58, 36.73, 34.80, 32.87, 28.62, 28.11, 27.97, 13.66. *Note: rotameric mixture.* HR-MS (ESI+) calc'd for $[M+H]^+$ $C_{26}H_{28}N_4O$: 413.2306, found 413.2311.

Final Degrader Compounds

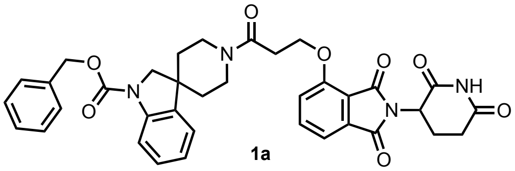

1a

Benzyl 1'-(3-((2-(2,6-dioxopiperidin-3-yl)-1,3-dioxoisoindolin-4-yl)oxy)propanoyl)spiro[indoline-3,4'-piperidine]-1-carboxylate (1a)

General Procedure 1A. Reaction scale: Thalidomide-O-C2-acid 19 mg (1 eq, 55.7 μmol) and Benzyl spiro[indoline-3,4'-piperidine]-1-carboxylate 20 mg (1 eq, 55.7 μmol). Purified by pTLC (6% MeOH/DCM) to afford **1a** as an off-white solid (8.0 mg, 43 %). ^{1}H NMR (600 MHz, $CDCl_3$) δ 8.65 (s, 1H), 7.93 (s, 1H), 7.74 – 7.64 (m, 2H), 7.51 – 7.44 (m, 6H), 7.46 – 7.40 (m, 3H), 7.36 – 7.30 (m, 2H), 7.24 – 7.16 (m, 2H), 7.16 – 7.12 (m, 2H), 7.06 – 6.98 (m, 2H), 5.42 – 5.21 (m, 4H), 4.96 (s, 2H), 4.70 – 4.64 (m, 2H), 4.62 – 4.54 (m, 4H), 4.06 – 3.96 (m, 5H), 3.48 (s, 1H), 3.25 (s, 1H), 3.08 – 2.98 (s, 2H), 2.83 – 2.68 (m, 6H), 2.10 (s, 2H), 1.97 (s, 1H), 1.93 – 1.77 (m, 6H), 1.73 – 1.71 (m, 4H). ^{13}C NMR (151 MHz, $CDCl_3$) δ 167.01, 165.79, 156.23, 136.72, 133.73, 128.68, 128.39, 128.31, 123.05, 119.16, 117.11, 116.15, 49.16, 39.00, 37.20, 36.33, 31.40. (*Note: rotameric mixture observed*) HR-MS (ESI+) calc'd for $C_{36}H_{34}N_4O_8$: 650.2377, found $[M+H]^+$ 651.2448.

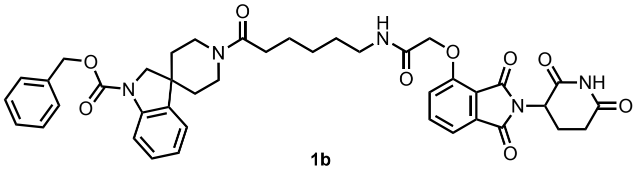

1b

Benzyl 1'-(6-(2-((2-(2,6-dioxopiperidin-3-yl)-1,3-dioxoisoindolin-4 yl)oxy) acetamido) hexanoyl) spiro[indoline-3,4'-piperidine]-1-carboxylate (1b)

General Procedure 1A. Reaction scale: Thalidomide-O-acetamido-C5-acid 19 mg (1eq, 41.7 μmol) and Benzyl spiro[indoline-3,4'-piperidine]-1-carboxylate 15 mg (1 eq, 41.7 μmol). Purified by pTLC (6% MeOH/DCM) to afford **1b** as an off-white solid (7.0 mg, 41 %). *Note: Repurified by C18 MeCN/H2O 0.1% TFA, Isolated as a complex with TFA.* ^{1}H NMR (400 MHz, $CDCl_3$) δ 9.52 (br m, 1H), 7.91 (br s, 1H), 7.83 – 7.71 (m, 2H), 7.57 (d, *J* = 7.3 Hz, 1H), 7.52 – 7.31 (m, 5H), 7.25 – 7.15 (m, 2H), 7.14 – 6.94 (m, 2H), 5.45 – 5.17 (m, 2H), 5.05 – 4.88 (m, 1H), 4.82 – 4.54 (m, 3H), 3.94 (s, 2H), 3.92 – 3.80 (m, 1H), 3.50 – 3.37 (m, 1H), 3.37 – 3.28 (m, 1H), 3.26 – 3.09 (m, 1H), 2.96 – 2.63 (m, 4H), 2.54 – 2.32 (m, 2H), 2.17 (s, 1H), 1.95 – 1.59 (m, 8H), 1.55 – 1.35 (m, 2H). ^{13}C NMR (151 MHz, $CDCl_3$) δ 172.73, 171.50, 168.51, 167.59, 166.69, 166.46, 154.82, 153.75 & 152.88 (rot), 141.71 & 140.50 (rot), 138.35 & 137.51 (rot), 137.28, 136.27 & 136.03 (rot), 133.64, 128.81, 128.72 (br), 128.53, 128.45, 123.29, 123.01 & 122.65 (rot), 120.48, 118.61, 117.88, 115.16 (br), 68.68, 68.23 & 67.39 (rot.), 57.50 & 57.33 (rot), 49.46, 43.24 & 42.75 (rot.) 42.08 (br), 39.43, 39.31, 37.35 & 36.57 (rot.), 33.27, 31.53, 28.89, 26.82, 25.50, 22.95. *Note: Rotameric peaks are located near the carbamate bond and exchanging ring conformers are visible.* HR-MS (ESI+) calc'd for $C_{41}H_{43}N_5O_9$: 749.3177, found $[M+H]^+$ 750.3142.

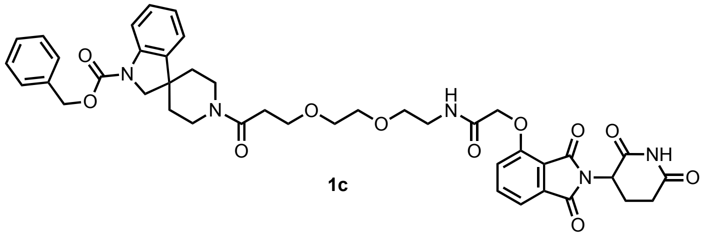

1c

Benzyl 1-(3-(2-(2-(2-((2-(2,6-dioxopiperidin-3-yl)-1,3-dioxoisoindolin-4-yl)oxy)acetamido)ethoxy) ethoxy)propanoyl)spiro[indoline-3,4'-piperidine]-1'-carboxylate (1c)

General Procedure 1A. Reaction scale: Thalidomide-O-acetamido-PEG2-C2-acid 20 mg (1 eq, 31.7 µmol) and 1'Benzyl spiro[indoline-3,4'-piperidine]-1-carboxylate 14 mg (1.1 eq, 34.9 µmol). Purified by pTLC (7% MeOH/DCM) to afford **1c** as an off-white solid (12.0 mg, 42%). ^{1}H NMR (600 MHz, $CDCl_3$) δ 9.64 (s, 2H), 7.98 – 7.96 (m, 1H), 7.84 – 7.72 (m, 3H), 7.68 – 7.55 (m, 2H), 7.50 – 7.44 (m, 6H), 7.37 – 7.21 (m, 3H), 7.19 – 7.13 (m, 2H), 7.11 – 6.99 (m, 2H), 6.70 – 6.68 (m, 1H), 5.42 – 5.34 (m, 4H), 5.17 – 4.92 (m, 2H), 4.76 – 4.72 (m, 5H), 4.09 – 3.83 (m, 8H), 3.83 – 3.67 (m, 8H), 3.66 – 3.64 (m, 2H), 3.26 – 3.24 (m, 3H), 3.03 – 2.59 (m, 10H), 2.23 – 2.19 (m, 2H), 2.10 – 2.06 (m, 3H), 1.90 – 1.86 (m, 3H), 1.78 – 1.74 (m, 3H), 1.58 – 1.38 (m, 8H). ^{13}C NMR (151 MHz, $CDCl_3$) δ 171.60, 169.68, 168.50, 166.87, 166.62, 165.72, 154.18, 153.45, 152.59, 141.42, 140.21, 138.27, 137.47, 136.88, 136.03, 133.45, 128.51, 128.32, 128.21, 128.11, 122.95, 122.42, 119.15, 117.70, 117.03, 114.79, 70.10, 70.07, 69.32, 67.84, 67.54, 67.14, 67.03, 56.86, 55.17, 49.15, 43.19, 42.78, 42.48, 41.82, 38.83, 37.00, 36.11, 33.30, 31.31, 29.53, 22.54, 18.38, 16.97, 12.45. (*Note: rotameric mixture observed*) HR-MS (ESI+) calc'd for $C_{42}H_{45}N_5O_{11}$: 795.3116, found $[M+H]^+$ 796.3183.

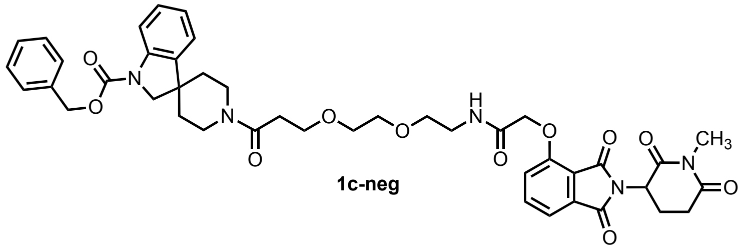

1c-neg

Benzyl 1-(3-(2-(2-(2-((2-(1-methyl-2,6-dioxopiperidin-3-yl)-1,3-dioxoisoindolin-4-yl)oxy)acetamido) ethoxy)ethoxy)propanoyl)spiro[indoline-3,4'-piperidine]-1'-carboxylate (1c-neg)

General Procedure 4. Reaction scale: 10 mg (1 eq, 13.3 µmol) **1c** and methyl iodide 6 mg (2.0 eq, 26.6 µmol). Purified by pTLC (6% MeOH/DCM) to afford **1c-neg** as a sticky solid (3 mg, 35%). ^{1}H NMR (600 MHz, $CDCl_3$) δ 7.90 (s, 1H), 7.72 (dd, *J* = 8.3, 7.4 Hz, 1H), 7.63 – 7.58 (m, 1H), 7.53 (d, *J* = 7.3 Hz, 1H), 7.43 (d, *J* = 7.4 Hz, 2H), 7.43 – 7.38 (m, 2H), 7.37 – 7.31 (m, 1H), 7.22 (s, 1H), 7.18 (d, *J* = 8.4 Hz, 1H), 7.08 (d, *J* = 7.4 Hz, 1H), 7.00 (d, *J* = 7.7 Hz, 1H), 5.46 – 5.14 (m, 3H), 5.05 – 4.95 (m, 1H), 4.64 (s, 3H), 3.93 – 3.87 (m, 3H), 3.85 – 3.75 (m, 2H), 3.67 – 3.61 (m, 5H), 3.60 – 3.56 (s, 2H), 3.20 (s, 3H), 3.17 – 3.14 (m, 1H), 3.03 – 2.94 (m, 1H), 2.86 – 2.76 (m, 2H), 2.69 – 2.63 (m, 3H), 2.14 – 2.08 (m, 1H), 1.82 – 1.78 (m, 2H), 1.75 – 1.64 (m, 4H). ^{13}C NMR (151 MHz, $CDCl_3$) δ 171.22, 171.20, 169.37, 168.82, 168.79, 167.02, 166.84, 166.82, 165.97, 165.95, 154.47, 154.45, 136.97, 136.95, 133.69, 133.67, 128.68, 128.66, 128.51, 128.37, 128.29, 128.27, 123.09, 122.51, 119.44, 118.04, 117.24, 115.02, 77.35, 77.14, 76.93, 70.39, 70.38, 70.32, 70.30, 69.57, 69.55, 67.97, 67.42, 67.20, 57.02, 55.11, 50.02, 50.01, 43.13, 42.82, 38.99, 38.98, 38.78, 37.21, 36.31, 33.56, 31.86, 31.84, 29.69, 27.26, 27.25, 21.94, 21.93, 18.59, 17.25, 12.41 (*Note: rotameric mixture observed*) HR-MS (ESI+) calc'd for $C_{43}H_{47}N_5O_{11}$: 809.3272, found $[M+H]^+$ 810.3352.

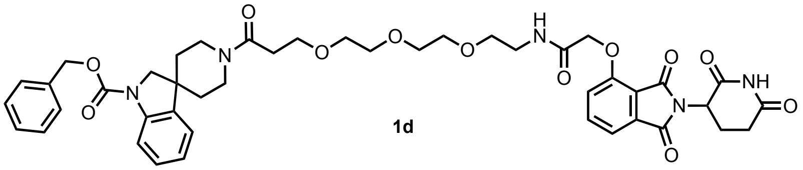

1d

Benzyl 1'-(1-((2-(2,6-dioxopiperidin-3-yl)-1,3-dioxoisoindolin-4-yl)oxy)-2-oxo-6,9,12-trioxa-3-azapentadecan-15-oyl)spiro[indoline-3,4'-piperidine]-1-carboxylate (1d)

General Procedure 1A. Reaction scale: Thalidomide-O-acetamido-PEG3-C2-acid 20 mg (1 eq, 31.7 µmol) and 1'Benzyl spiro[indoline-3,4'-piperidine]-1-carboxylate 13 mg (1.1 eq, 34.9 µmol). Purified by pTLC (7% MeOH/DCM) to afford **1d** as an off-white solid (5.0 mg, 38%). *Note: Repurified by C18 MeCN/H2O 0.1% TFA, Isolated as a complex with TFA.* ^{1}H NMR (600 MHz, $CDCl_3$) δ 9.05 (s, 1H), 7.93 – 7.86 (m, 1H), 7.80 – 7.68 (m, 2H), 7.54 (d, *J* = 7.2 Hz, 1H), 7.48 – 7.31 (m, 5H), 7.24 – 7.17 (m, 2H), 7.11 – 6.96 (m, 2H), 5.29 (br m, 2H), 4.95 (dd, *J* = 12.3, 5.2 Hz, 1H), 4.66 (br m, 3H), 3.93 (br m, 3H), 3.83 (br s, 2H), 3.75 – 3.50 (m, 12H), 3.28 – 3.09 (m, 1H), 2.88 (dd, *J* = 15.9, 4.1 Hz, 1H), 2.85 – 2.64 (m, 5H), 2.14 (dt, *J* = 12.0, 4.5 Hz, 1H), 1.82 (t, *J* = 12.9 Hz, 2H), 1.70 (t, *J* = 10.7 Hz, 2H). ^{13}C NMR (151 MHz, $CDCl_3$) δ 171.19, 170.12, 168.21, 167.24, 166.67, 165.88, 154.41, 152.78 (br), 141.61 & 140.42 (rot.), 138.33 & 137.49 (rot), 137.05, 136.16 & 135.88 (rot.), 133.66, 128.69, 128.57 (br), 128.40, 128.32, 123.13, 122.54 (br), 119.43, 118.04, 117.40, 115.26 & 115.05 (rot.), 70.45, 70.34, 70.27, 70.26, 69.41, 68.07 (br), 67.90, 67.27, 57.40 & 57.07 (rot.), 49.34, 43.19 (br), 42.63 (br), 41.94 (br), 39.19, 37.22 (br), 36.32 (br), 33.43, 31.48, 22.70. *Note: Rotameric peaks are located near the carbamate bond and exchanging ring conformers are visible.* HR-MS (ESI+) calc'd for $C_{44}H_{49}N_5O_{12}$: 839.3378, found $[M+H]^+$ 840.3449.

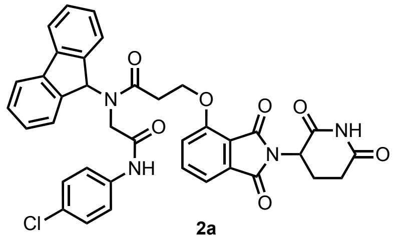

2a

N-(2-((4-chlorophenyl)amino)-2-oxoethyl)-3-((2-(2,6-dioxopiperidin-3-yl)-1,3 dioxoisoindolin-4-yl)oxy)-N-(9H-fluoren-9-yl)propenamide (2a)

General Procedure 1A. Reaction scale: Thalidomide-O-C2-acid 20 mg (1 eq, 57.3 µmol) and **F2** 20 mg (57.3 µmol, 1 eq). Purified by pTLC (6% MeOH/DCM) to afford **2a** as a sticky solid (8.0 mg, 42 %). ^{1}H NMR (600 MHz, $CDCl_3$) δ 9.62 (s, 1H), 8.14 – 8.12 (m, 1H), 7.84 – 7.68 (m, 4H), 7.63 (s, 2H), 7.56 – 7.47 (m, 2H), 7.42 – 7.39 (m, 2H), 7.21 – 7.18 (m, 3H), 6.73 – 6.70 (m, 1H), 6.40 – 6.36 (m, 1H), 6.13 – 6.11 (m, 1H), 5.30 (s, 2H), 5.17 (s, 1H), 4.85 – 4.76 (m, 1H), 3.42 (t, J = 5.7 Hz, 2H), 3.16 – 2.98 (m, 4H), 2.93 – 2.83 (m, 1H), 2.86 – 2.76 (m, 1H), 2.75 – 2.67 (m, 1H), 2.66 – 2.68 (m, 2H). ^{13}C NMR (151 MHz, $CDCl_3$) δ 170.76, 170.64, 170.05, 167.70, 167.64, 166.36, 166.19, 164.92, 164.75, 163.33, 146.84, 146.40, 141.12, 136.47, 136.29, 136.20, 134.33, 133.26, 133.16, 128.93, 128.86, 128.69, 127.57, 126.70, 125.41, 122.46, 121.71, 121.50, 120.82, 120.40, 66.66, 56.28, 53.46, 49.40, 49.35, 31.34, 31.29, 22.52, 22.37. (*Note: rotameric mixture observed*) HR-MS (ESI+) calc'd for $C_{37}H_{29}ClN_4O_7$: 676.1725, found $[M+H]^+$ 677.1798.

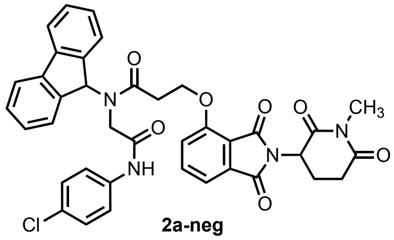

2a-neg

N-(2-((4-chlorophenyl)amino)-2-oxoethyl)-N-(9H-fluoren-9-yl)-3-((2-(1-methyl-2,6-dioxopiperidin-3-yl)-1,3-dioxoisoindolin-4-yl)oxy)propenamide (2a-neg)

General Procedure 4. Reaction scale: bifunctional molecule **2a** 9.6 mg (14.1 µmol, 1 eq) and methyl iodide 2 mg (28.2 µmol, 1 eq). Purified by pTLC (6% MeOH/DCM) to afford **2a-neg** as a sticky solid (3.0 mg, 32%). ^{1}H NMR (600 MHz, $CDCl_3$) δ 9.61 (s, 1H), 7.84 – 7.68 (m, 6H), 7.62 (s, 3H), 7.54 – 7.52 (m, 3H), 7.45 – 7.38 (m, 4H), 7.21 – 7.17 (m, 4H), 5.17 (s, 1H), 4.82 – 4.78 (m, 1H), 3.43 (s, 2H), 3.16 – 3.13 (m, 4H), 3.10 – 3.02 (m, 4H), 2.96 – 2.88 (m, 2H), 2.65 (s, 3H), 1.97 (s, 1H). ^{13}C NMR (151 MHz, CDCl3) δ 170.76, 170.64, 170.05, 167.70, 167.64, 166.36, 166.19, 164.92, 164.75, 163.33, 146.84, 146.40, 141.12, 136.47, 136.29, 136.20, 134.33, 133.26, 133.16, 128.93, 128.86, 128.69, 127.57, 126.70, 125.41, 122.46, 121.71, 121.50, 120.82, 120.40, 66.66, 56.28, 53.46, 49.40, 49.35, 31.34, 31.29, 22.52, 22.37. (*Note: rotameric mixture observed*) HR-MS (ESI+) calc'd for $C_{38}H_{31}ClN_4O_7$: 690.1881, found $[M+H]^+$ 691.1957.

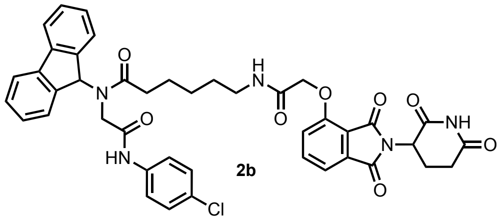

2b

N-(2-((4-chlorophenyl)amino)-2-oxoethyl)-6-(2-((2-(2,6-dioxopiperidin-3-yl)-1,3-dioxoisoindolin-4-yl)oxy)acetamido)-N-(9H-fluoren-9-yl)hexanamide (2b)

General Procedure 1A. Reaction scale: Thalidomide-O-C5-acid 20 mg (1 eq, 44.9 µmol) and **F2** 16 mg (1.1 eq, 44.9 µmol). Purified by pTLC (6% MeOH/DCM) to afford **2b** as a sticky solid (7.0 mg, 45 %). ^{1}H NMR (600 MHz, $CDCl_3$) δ 8.44 (s, 1H), 7.74 – 7.68 (m, 6H), 7.55 (s, 3H), 7.42 – 7.38 (m, 6H), 7.19 (s, 6H), 7.06 (s, 2H), 6.98 (s, 1H), 5.95 (s, 1H), 4.96 – 4.92 (m, 2H), 4.64 – 4.58 (m, 4H), 3.56 – 3.24 (m, 7H), 3.00 – 2.82 (m, 4H), 2.74 (s, 3H), 2.65 (s, 1H), 2.50 (s, 1H), 2.14 (s, 2H), 1.95 (s, 2H), 1.84 (s, 2H), 1.72 (s, 3H), 1.56 (s, 4H), 1.46 – 1.43 (m, 3H), 1.26 – 1.23 (m, 7H). ^{13}C NMR (151 MHz, DMSO) δ 172.85, 170.14, 169.93, 166.75, 165.54, 155.07, 145.02, 140.37, 137.54, 136.96, 133.06, 128.57, 128.15, 127.33, 126.78, 125.13, 120.73, 120.39, 120.04, 116.81, 116.09, 67.63, 62.33, 48.82, 47.14, 38.74, 38.04, 30.97, 28.49, 26.65, 23.08, 22.01. (*Note: rotameric mixture observed*) HR-MS (ESI+) calc'd for $C_{42}H_{38}ClN_5O_8$: 775.2409, found $[M+H]^+$ 776.2471

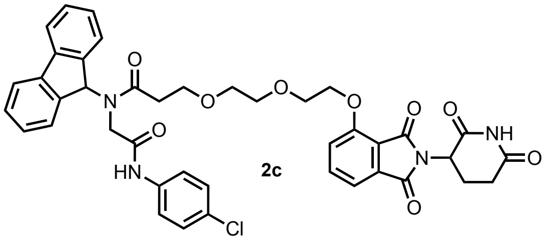

2c

N-(2-((4-chlorophenyl)amino)-2-oxoethyl)-3-(2-(2-((2-(2,6-dioxopiperidin-3-yl)-1,3-dioxoisoindolin-4-yl)oxy)ethoxy)ethoxy)-N-(9H-fluoren-9-yl)propenamide (2c)

General Procedure 1A. Reaction scale: Thalidomide-O-PEG2-C2-acid 15 mg (34.5 μmol, 1 eq) and **F2** 12 mg (34.5 μmol, 1 eq). Purified by pTLC (7% MeOH/DCM) to afford **2c** as a sticky solid (3.0 mg, 28%). ^{1}H NMR (600 MHz, $CDCl_3$) δ 8.54 (s, 1H), 8.09 (br m, 1H), 7.73 (d, *J* = 7.6 Hz, 1H), 7.70 – 7.58 (m, 2H), 7.51 (t, *J* = 6.4 Hz, 1H), 7.49 – 7.45 (m, 1H), 7.45 – 7.41 (m, 2H), 7.33 (q, *J* = 6.8 Hz, 1H), 7.30 – 7.18 (m, 4H), 7.16 – 7.10 (m, 2H), 6.95 (s, 1H), 6.10 (s, 1H), 4.93 – 4.70 (m, 1H), 4.20 (q, *J* = 5.1 Hz, 2H), 4.09 – 4.04 (m, 1H), 4.00 (t, *J* = 6.2 Hz, 1H), 3.95 – 3.83 (m, 2H), 3.82 – 3.70 (m, 4H), 3.56 – 3.32 (m, 2H), 3.21 (t, *J* = 6.0 Hz, 1H), 2.98 – 2.49 (m, 4H), 2.15 – 1.93 (m, 1H). ^{13}C NMR (151 MHz, $CDCl_3$) δ 174.79 & 174.46 (rot.), 171.05 & 170.82 (rot), 168.56 & 168.26 (rot.), 167.35 & 167.05 (rot.), 167.03 & 166.96 (rot.), 165.98 & 165.79 (rot.), 156.40 & 156.36 (rot.), 141.74, 141.53 & 141.39 (rot.), 140.98 & 140.96 (rot.), 140.79 & 140.76, 136.79 & 136.65 (rot.), 136.50 & 135.83 (rot.), 133.85 & 133.72 (rot.), 129.77, 129.65, 129.18 & 129.15 (rot.), 129.06, 128.96 & 128.91 (rot.), 127.99, 127.89 & 127.84, 126.01, 125.14, 121.54 & 121.38 (rot.), 120.79, 120.31 & 120.26, 119.28 & 119.15, 117.35 & 117.05, 116.33, 71.14 & 71.05 (rot.), 70.85 & 70.79 (rot.), 69.36, 69.32 & 69.07 (rot.), 68.08 & 67.81 (rot.), 63.45, 59.61, 49.23 & 48.36 (rot.), 34.64 & 34.46 (rot.), 31.47 & 31.34 (rot), 22.72. *Note*: *Mixture of two rotamers. Rotameric pairs are indicated where obvious.* HR-MS (ESI+) calc'd for $C_{41}H_{37}ClN_4O_9$: 764.2249, found $[M+H]^+$ 765.2313.

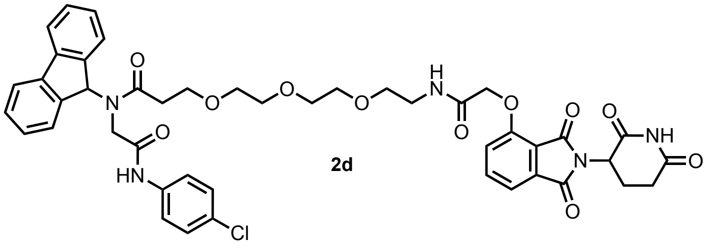

N-(2-((4-chlorophenyl)amino)-2-oxoethyl)-3-(2-(2-(2-(2-((2-(2,6-dioxopiperidin-3-yl)-1,3-dioxoisoindolin-4-yl)oxy)acetamido)ethoxy)ethoxy)ethoxy)-N-(9H-fluoren-9-yl)propenamide (2d)

General Procedure 1A. Reaction scale: Thalidomide-O-acetamido-PEG3-C2-acid 20 mg (29.7 μmol, 1 eq) and **F2** 10 mg (29.7 μmol, 1 eq). Purified by pTLC (7% MeOH/DCM) to afford **2d** as a sticky solid (8mg, 40 %). ^{1}H NMR (600 MHz, $CDCl_3$) δ 9.30 (s, 1H), 9.16 (s, 1H), 8.52 (s, 1H), 8.19 (s, 1H), 7.73 – 7.70 (m, 3H), 7.69 – 7.67 (m, 1H), 7.72 – 7.64 (m, 1H), 7.67 – 7.62 (m, 3H), 7.61 (s, 1H), 7.52 – 7.48 (m, 6H), 7.44 – 7.46 (m, 2H), 7.36 – 7.30 (m, 2H), 7.28 – 7.23 (m, 6H), 7.22 – 7.19 (m, 1H), 7.18 – 7.09 (m, 6H), 6.88 (s, 1H), 6.03 (s, 1H), 4.96 – 4.94 (m, 1H), 4.93 – 4.89 (m, 1H), 4.65 – 4.50 (m, 4H), 4.07 – 3.90 (m, 4H), 3.73 – 3.68 (m, 2H), 3.64 – 3.58 (m, 7H), 3.56 – 3.52 (m, 6H), 3.51 – 3.46 (m, 2H), 3.44 – 3.40 (m, 2H), 3.38 (s, 2H), 3.22 – 3.18 (m, 2H), 3.15 – 3.12 (m, 1H), 2.80 – 2.74 (m, 4H), 2.12 – 2.08 (m, 4H), 1.46 – 1.39 (m, 4H).^{13}C NMR (151 MHz, $CDCl_3$) δ 174.42, 173.86, 171.57, 168.91, 167.11, 166.99, 166.89, 166.68, 166.60, 165.89, 165.86, 154.37, 154.27, 141.72, 141.60, 141.31, 141.26, 140.74, 137.03, 137.01, 136.55, 135.98, 133.52, 133.48, 129.54, 129.19, 128.98, 128.70, 128.68, 128.65, 127.85, 127.83, 127.70, 127.67, 125.85, 125.17, 121.51, 121.19, 120.52, 120.09, 120.07, 119.45, 119.34, 117.86, 117.79, 117.25, 117.20, 77.32, 77.11, 76.90, 70.64, 70.33, 70.27, 70.24, 70.22, 70.18, 70.17, 69.50, 67.81, 67.68, 67.49, 67.39, 63.20, 59.50, 55.24, 49.27, 47.95, 43.24, 39.08, 39.00, 34.35, 33.99, 31.37, 22.62, 22.59, 18.55, 17.18, 12.46. (*Note: rotameric mixture observed*) HR-MS (ESI+) calc'd for $C_{45}H_{44}ClN_5O_{11}$: 865.2726, found $[M+H]^+$ 866.2799.

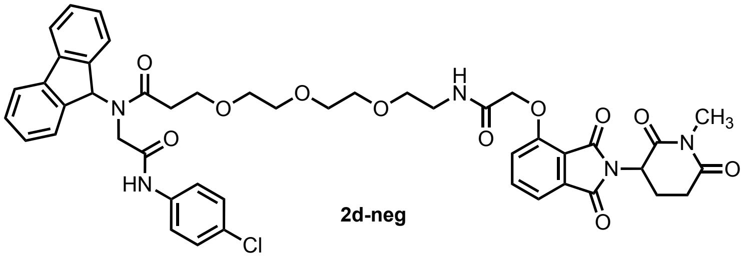

N-(2-((4-chlorophenyl)amino)-2-oxoethyl)-N-(9H-fluoren-9-yl)-3-(2-(2-(2-(2-((2-(1-methyl-2,6-dioxopiperidin-3-yl)-1,3-dioxoisoindolin-4-yl)oxy)acetamido)ethoxy)ethoxy)ethoxy)propenamide (2d-neg)

General Procedure 4. Reaction scale: bifunctional molecule **2d** 12 mg (13.8 µmol, 1 eq) and methyl iodide 2 mg (27.7 µmol, 2 eq). Purified by pTLC (6% MeOH/DCM) to afford **2d-neg** as a sticky solid (6.0 mg, 50 %). ^{1}H NMR (600 MHz, $CDCl_3$) δ 8.62 (s, 1H), 8.02 (s, 1H), 7.79 – 7.66 (m, 6H), 7.57 – 7.50 (m, 7H), 7.47 – 7.42 (m, 2H), 7.41 – 7.36 (m 2H), 7.32 – 7.24 (m, 6H), 7.24 – 7.13 (m, 4H), 6.97 (s, 1H), 6.10 (s, 1H), 4.98 – 4.94 (m, 2H), 4.62 – 4.59 (m, 2H), 4.56 – 4.54 (m, 2H), 4.06 – 4.04 (m, 2H), 4.00 – 4.94 (m, 2H), 3.75 – 3.66 (m, 4H), 3.65 – 3.56 (m, 13H), 3.55 – 3.49 (m, 5H), 3.47 – 3.37 (m, 4H), 3.24 – 3.18 (m, 4H), 3.18 (s, 3H), 2.98 – 2.94 (m, 2H), 2.86 – 2.82 (m, 2H), 2.81 – 2.73 (m, 3H), 2.15 – 2.06 (m, 2H). ^{13}C NMR (151 MHz, $CDCl_3$) δ 174.24, 173.80, 171.15, 171.12, 168.77, 168.70, 167.06, 166.93, 166.79, 166.75, 165.98, 154.46, 154.41, 141.92, 141.86, 141.27, 140.82, 140.75, 136.95, 136.55, 136.03, 133.63, 133.60, 129.60, 129.27, 128.95, 128.78, 127.85, 127.70, 125.94, 125.91, 125.03, 121.33, 121.18, 121.11, 120.61, 120.06, 119.45, 118.00, 117.98, 117.34, 117.30, 70.71, 70.55, 70.46, 70.38, 70.27, 70.23, 69.48, 69.38, 68.15, 67.99, 67.91, 67.65, 63.26, 59.42, 50.00, 48.16, 47.97, 39.03, 38.94, 34.45, 34.16, 31.95, 31.87, 29.72, 27.29, 22.72, 21.92. (*Note: rotameric mixture observed*) HR-MS (ESI+) calc'd for $C_{46}H_{46}ClN_5O_{11}$: 879.2882, found $[M+H]^+$ 880.2954.

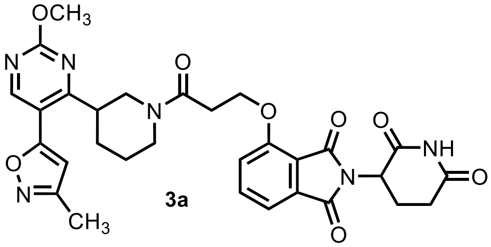

2-(2,6-dioxopiperidin-3-yl)-4-(3-(3-(2-methoxy-5-(3-methylisoxazol-5-yl)pyrimidin-4-yl)piperidin-1-yl)-3-oxopropoxy)isoindoline-1,3-dione (3a)

General Procedure 1A. Reaction scale: Thalidomide-O-C2-acid 14 mg (38.6 µmol, 1 eq) and 5-(2-methoxy-4-(piperidin-3-yl)pyrimidin-5-yl)-3-methylisoxazole 15 mg (38.6 µmol, 1 eq). Purified by pTLC (6% MeOH/DCM) to afford **3a** as a sticky solid (5.0 mg, 32 %). ^{1}H NMR (600 MHz, $CDCl_3$) δ 8.67 (d, *J* = 1.1 Hz, 1H), 8.64 – 8.61 (m, 1H), 8.17 – 8.14 (m, 1H), 8.12 – 8.09 (m, 1H), 7.72 – 7.65 (m, 2H), 7.49 – 7.43 (m, 2H), 7.33 – 7.27 (m, 2H), 6.42 – 6.38 (m, 1H), 6.32 – 6.28 (m, 1H), 4.96 – 4.92 (m, 1H), 4.91 – 4.84 (m, 1H), 4.76 – 4.72 (m, 1H), 4.70 – 4.65 (m, 1H), 4.58 – 4.45 (m, 2H), 4.12 – 4.05 (m, 4H), 4.04 – 4.00 (m, 1H), 3.58 – 3.48 (m, 1H), 3.31 – 3.11 (m, 3H), 3.07 – 2.92 (m, 2H), 2.91 – 2.85 (m, 2H), 2.83 – 2.63 (m, 4H), 2.35 – 2.28 (m, 4H), 2.16 – 2.04 (m, 3H), 2.03 – 1.96 (m, 1H), 1.92 – 1.88 (m, 1H), 1.86 – 1.82 (m, 1H), ^{13}C NMR (151 MHz, $CDCl_3$) δ 171.15, 170.81, 170.70, 168.31, 168.17, 166.93, 165.67, 165.53, 165.40, 165.28, 164.76, 160.43, 159.46, 159.38, 156.19, 136.64, 133.65, 133.56, 119.08, 117.01, 116.07, 115.95, 115.37, 104.53, 104.08, 65.90, 55.33, 55.25, 49.04, 48.95, 46.20, 45.67, 41.92, 40.87, 32.57, 31.33, 30.00, 25.59, 22.53, 11.42, 11.34. (*Note: rotameric mixture observed*) HR-MS (ESI+) calc'd for $C_{30}H_{30}N_6O_8$: 602.2125, found $[M+H]^+$ 603.2198.

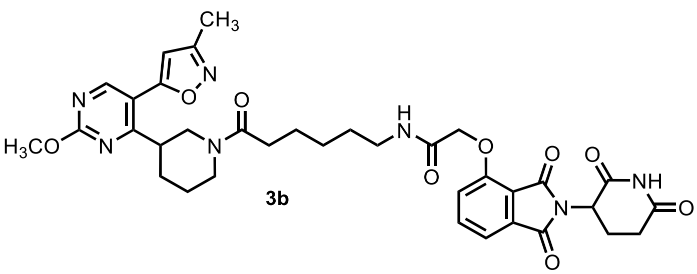

2-((2-(2,6-dioxopiperidin-3-yl)-1,3-dioxoisoindolin-4-yl)oxy)-N-(6-(3-(2-methoxy-5-(3-methylisoxazol-5-yl)pyrimidin-4-yl)piperidin-1-yl)-6-oxohexyl)acetamide (3b)

General Procedure 1A. Reaction scale: Thalidomide-O-acetamido-C5-acid 16 mg (38.6 µmol, 1 eq) and 5-(2-methoxy-4-(piperidin-3-yl)pyrimidin-5-yl)-3-methylisoxazole 15 mg (38.6 µmol, 1 eq) Purified by pTLC (6% MeOH/DCM) to afford **3b** as a sticky solid (9.0 mg, 38 %). ^{1}H NMR (600 MHz, $CDCl_3$) δ 8.70 – 8.54 (m, 2H), 7.73 – 7.68 (m, 2H), 7.67 – 7.57 (m, 2H), 7.56 – 7.54 (m, 2H), 7.21 – 7.18 (m, 2H), 6.46 – 6.42 (m, 1H), 6.28 – 6.24 (m, 1H), 5.03 – 4.98 (m, 1H), 4.98 – 4.94 (m, 1H), 4.80 – 4.73 (m, 1H), 4.73 – 4.63 (m, 4H), 4.61 – 4.58 (m, 2H), 4.12 – 4.08 (m, 3H), 4.07 – 4.04 (m, 6H), 3.97 – 3.94 (m, 1H), 3.80 – 3.76 (m, 1H), 3.70 – 3.57 (m, 1H), 3.41 – 3.38 (m, 3H), 3.32 – 3.26 (m, 2H), 3.20 – 3.04 (m, 3H), 3.00 – 2.96 (m, 1H), 2.92 – 2.81 (m, 2H), 2.81 – 2.70 (m, 4H), 2.63 – 2.51 (m, 1H), 2.38 – 2.34 (m, 6H), 2.16 – 2.14 (m, 2H), 2.02 – 1.83 (m, 8H), 1.65 – 1.60 (m, 6H), 1.59 – 1.52 (m, 4H), 1.48 – 1.34 (m, 6H). ^{13}C NMR (151 MHz, $CDCl_3$) δ 172.11, 172.03, 171.94, 171.43, 171.37, 171.30, 171.12, 170.95, 170.85, 170.77, 168.44, 168.36, 166.84, 166.78, 166.56, 166.19, 165.59, 165.42, 165.37, 165.23, 164.83, 164.74, 160.57, 160.43, 160.37, 159.48, 159.38, 154.70, 136.97, 133.49, 120.12, 118.38, 117.51, 117.48, 115.47, 115.39, 115.24, 104.69, 104.56, 104.01, 68.53, 60.34, 55.31, 55.19, 49.69, 49.61, 49.29, 49.26, 46.16, 46.13, 45.73, 45.63, 42.05, 41.96, 40.85, 40.80, 39.17, 39.12, 33.25, 33.18, 33.05, 33.01, 31.86, 31.34, 31.31, 30.10, 30.06, 29.87, 29.63, 28.84, 28.80, 28.77, 28.73, 26.72, 26.63, 25.69, 25.66, 25.31, 25.25, 25.17, 24.50, 22.83, 22.76, 22.62, 21.00, 14.14, 14.06, 11.44, 11.42, 11.38. (*Note: rotameric mixture observed*) HR-MS (ESI+) calc'd for $C_{35}H_{39}N_7O_9$: 701.2809, found $[M+H]^+$ 702.2878.

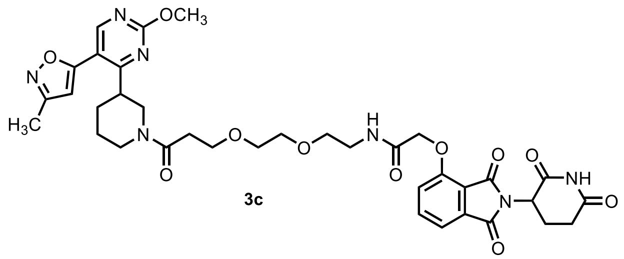

3c

2-((2-(2,6-dioxopiperidin-3-yl)-1,3-dioxoisoindolin-4-yl)oxy)-N-(2-(2-(3-(3-(2-methoxy-5-(3-methylisoxazol-5-yl)pyrimidin-4-yl)piperidin-1-yl)-3-oxopropoxy)ethoxy)ethyl)acetamide (3c)

General Procedure 1A. Reaction scale: Thalidomide-O-acetamido-PEG2-C2-acid 20 mg (31.7 µmol, 1 eq) and 5-(2-methoxy-4-(piperidin-3-yl)pyrimidin-5-yl)-3-methylisoxazole 14 mg (31.7 µmol, 1.1 eq) Purified by pTLC (6% MeOH/DCM) to afford **3c** as a sticky solid (5.0 mg). ^{1}H NMR (600 MHz, $CDCl_3$) δ 9.54 (s, 1H), 8.69 – 8.59 (m, 1H), 7.75 – 7.70 (m, 1H), 7.68 – 7.61 (m, 1H), 7.57 – 7.50 (m, 1H), 7.22 – 7.14 (m, 1H), 6.47 – 6.21 (m, 1H), 5.00 – 4.92 (m, 1H), 4.79 – 4.56 (m, 3H), 4.13 – 4.03 (m, 3H), 4.01 – 3.88 (m, 1H), 3.87 – 3.74 (m, 2H), 3.74 – 3.42 (m, 9H), 3.29 – 2.94 (m, 2H), 2.92 – 2.53 (m, 5H), 2.45 – 2.26 (m, 3H), 2.13 (ddt, J = 8.6, 5.8, 3.6 Hz, 1H), 2.07 – 1.87 (m, 2H), 1.80 – 1.75 (m, 1H), 1.67 – 1.48 (m, 1H). *Note: Presence of rotamers splitting signals in two or more peaks (integrated together).* ^{13}C NMR (151 MHz, $CDCl_3$) δ 171.54, 171.51, 171.45, 171.07, 170.94, 170.87, 169.82, 169.68, 168.48, 168.44, 166.87, 166.84, 166.83, 165.88, 165.87, 165.85, 165.74, 165.59, 165.58, 165.31, 164.94, 164.90, 160.68, 160.67, 160.58, 159.62, 159.57, 159.55, 154.46, 154.45, 154.44, 137.02, 136.99, 133.87, 119.14, 118.12, 118.10, 118.09, 117.31, 117.28, 115.57, 115.37, 115.35, 104.74, 104.23, 104.20, 70.42, 70.38, 70.36, 70.35, 70.25, 69.63, 69.62, 69.59, 67.78, 67.43, 67.39, 67.38, 55.49, 55.38, 49.64, 49.46, 46.25, 45.84, 45.77, 42.20, 41.98, 41.96, 40.99, 40.94, 39.08, 33.60, 33.48, 31.62, 30.41, 30.19, 30.14, 25.71, 24.63, 22.87, 11.63, 11.59, 11.56. *Note: complex rotameric mixture observed up to 4 signals per carbon, all signals reported.* HR-MS (ESI+) calc'd for $C_{36}H_{41}N_7O_{11}$: 747.2864, found $[M+H]^+$ 748.2924.

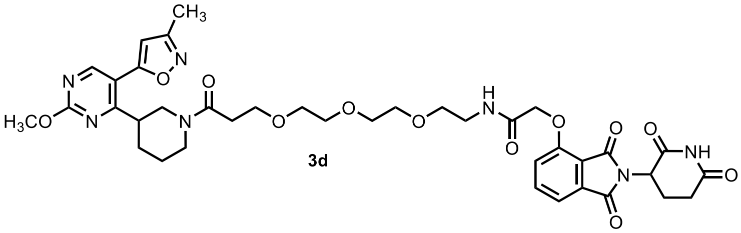

3d

2-((2-(2,6-dioxopiperidin-3-yl)-1,3-dioxoisoindolin-4-yl)oxy)-N-(2-(2-(2-(3-(3-(2-methoxy-5-(3-methylisoxazol-5-yl)pyrimidin-4-yl)piperidin-1-yl)-3-oxopropoxy)ethoxy)ethoxy)ethyl)acetamide (3d)

General Procedure 1A. Reaction scale: Thalidomide-O-acetamido-PEG3-C2-acid 32 mg (60.2 µmol, 1 eq) and 5-(2-methoxy-4-(piperidin-3-yl)pyrimidin-5-yl)-3-methylisoxazole 20 mg (60.2 µmol, 1 eq) Purified by pTLC (6% MeOH/DCM) to afford **3d** as a sticky solid (15.0 mg, 45%). ^{1}H NMR (600 MHz, $CDCl_3$) δ 9.07 (br m, 1H), 8.84 – 8.54 (m, 1H), 7.80 (d, *J* = 20.9 Hz, 1H), 7.73 (t, *J* = 7.8 Hz, 1H), 7.54 (dd, *J* = 7.3, 0.9 Hz, 1H), 7.23 – 7.15 (m, 1H), 6.55 – 6.17 (m, 1H), 5.02 – 4.90 (m, 1H), 4.82 – 4.59 (m, 3H), 4.10 – 4.00 (m, 3H), 4.03 – 3.91 (m, 1H), 3.78 (br s, 2H), 3.71 – 3.40 (m, 13H), 3.15 – 2.97 (m, 2H, partially under water peak), 2.92 – 2.55 (m, 5H), 2.43 – 2.33 (m, 3H), 2.19 – 2.08 (m, 1H), 2.06 – 1.88 (m, 2H), 1.87 – 1.77 (m, 1H), 1.66 – 1.47 (m, 1H). *Note: Presence of rotamers splitting signals in two or more peaks (integrated together).* ^{13}C NMR (151 MHz, $CDCl_3$) δ 171.35, 171.11, 170.91, 169.59, 169.52, 168.39, 166.99, 166.78, 165.92, 165.70, 165.54, 165.32, 164.85, 160.66, 160.60, 159.61, 159.51, 154.56, 137.06, 133.78, 119.45, 118.14, 117.36, 117.32, 115.57, 115.36, 104.76, 104.24, 77.79, 70.49, 70.40, 70.36, 69.60, 68.00, 67.35, 67.29, 55.47, 55.35, 49.60, 49.43, 46.23, 45.78, 42.10, 41.96, 41.01, 39.20, 39.18, 33.57, 33.43, 31.58, 30.15, 29.80, 25.73, 24.64, 22.78, 11.62, 11.56. *Note: Complex rotameric mixture up to 4 signals per carbon, all signals reported.* HR-MS (ESI+) calc'd for $C_{38}H_{45}N_7O_{12}$: 791.3126, found $[M+H]^+$ 792.3108.

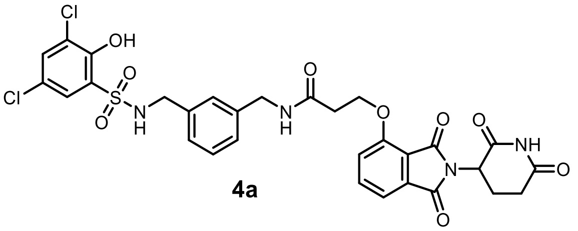

4a

N-(3-(((3,5-dichloro-2-hydroxyphenyl)sulfonamido)methyl)benzyl)-3-((2-(2,6-dioxopiperidin-3-yl)-1,3-dioxoisoindolin-4-yl)oxy)propanamide (4a)

General Procedure 1A. Reaction scale: thalidomide-O-C2-acid 20 mg (57.75 µmol, 1 eq) and N-(3-(aminomethyl)benzyl)-3-chloro-2-hydroxybenzenesulfonamide (**F4**) 20.85 mg (57.75 µmol, 1 eq) Purified by pTLC (6% MeOH/DCM) to afford **4a** as an off-white powder (7.4 mg, 19%). ^{1}H NMR (400 MHz, $CDCl_3$) δ 8.76 (s, 1H), 7.70 (dd, *J* = 8.4, 7.3 Hz, 1H), 7.51 (d, *J* = 2.5 Hz, 1H), 7.49 – 7.40 (m, 2H), 7.37 (d, *J* = 2.5 Hz, 1H), 7.24 (d, *J* = 8.5 Hz, 1H), 7.12 (s, 1H), 7.10 – 6.98 (m, 2H), 6.85 (d, *J* = 7.3 Hz, 1H), 6.38 (t, *J* = 6.3 Hz, 1H), 4.96 (dd, *J* = 12.2, 5.4 Hz, 1H), 4.43 (t, *J* = 5.5 Hz, 2H), 4.41 – 4.29 (m, 2H), 4.05 (d, *J* = 5.6 Hz, 2H), 2.91 – 2.62 (m, 5H), 2.17 – 2.03 (m, 2H, partially under water peak). *Note: Co-isolated with <2% MeOH.* ^{13}C NMR (151 MHz, $CDCl_3$) δ 171.51, 171.02, 168.87, 166.77, 166.62, 155.48, 148.81, 138.94, 137.12, 135.56, 133.92, 133.43, 128.37, 127.30, 127.24, 127.01, 126.88, 126.39, 124.80, 123.56, 118.91, 117.17, 116.72, 65.52, 49.15, 47.19, 43.41, 36.63, 31.26, 22.58. HR-MS (ESI+) calc'd for $C_{30}H_{26}Cl_2N_4O_9S$: 688.0798 found $[M+H]^+$ 689.0868.

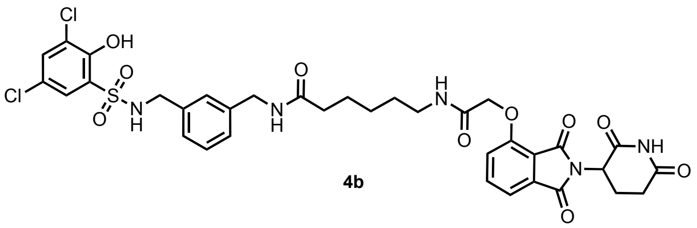

4b

N-(3-(((3,5-dichloro-2-hydroxyphenyl)sulfonamido)methyl)benzyl)-6-(2-((2-(2,6-dioxopiperidin-3-yl)-1,3-dioxoisoindolin-4-yl)oxy)acetamido)hexanamide (4b)

General Procedure 1A. Reaction scale: Thalidomide-O-acetamido-C5-acid 14.6 mg (32.7 µmol, 1 eq) and N-(3-(aminomethyl)benzyl)-3-chloro-2-hydroxybenzenesulfonamide (**F4**) 15.0 mg (32.7 µmol, 1 eq). Purified by pTLC (8% MeOH/DCM) to afford **4b** as an off-white powder (9.0 mg, 35%). ^{1}H NMR (400 MHz, MeOD) δ 8.10 – 8.02 (m, 1H), 7.80 (dd, *J* = 8.4, 7.3 Hz, 1H), 7.57 – 7.49 (m, 3H), 7.41 (d, *J* = 8.4 Hz, 1H), 7.21 – 7.01 (m, 4H), 5.12 (dd, *J* = 12.6, 5.4 Hz, 1H), 4.74 (s, 2H), 4.28 (s, 2H), 4.14 (s, 2H), 3.01 – 2.60 (m, 3H), 2.24 (t, *J* = 7.4 Hz, 2H), 2.13 (dtd, *J* = 10.9, 5.2, 2.8 Hz, 1H), 1.67 (p, *J* = 7.4 Hz, 2H), 1.60 (p, *J* = 6.8 Hz, 2H), 1.46 – 1.34 (m, 2H). *Note: 1 CH2 signal α to amide missing (visible in a CDCl3 spectrum, see below).*

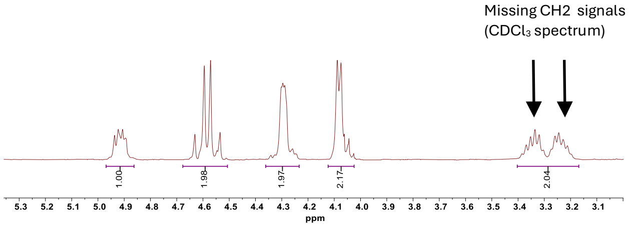

Spectrum of 4b in $CDCl_3$ from a less pure sample (high field aliphatic impurities).

^{13}C NMR (151 MHz, MeOD) δ 175.98, 174.60, 171.42, 169.90, 168.29, 167.79, 156.25, 150.75, 140.32, 138.23, 134.89, 134.48, 130.83, 129.44, 128.63, 128.14, 127.82, 127.75, 125.19, 124.56, 121.73, 119.31, 117.97, 69.40, 50.54, 47.97, 43.88, 39.99, 36.94, 32.15, 29.92, 27.43, 26.64, 23.64. HR-MS (ESI+) calc'd for $C_{35}H_{35}Cl_2N_5O_{10}S$: 787.1482 found $[M+H]^+$ 788.1549.

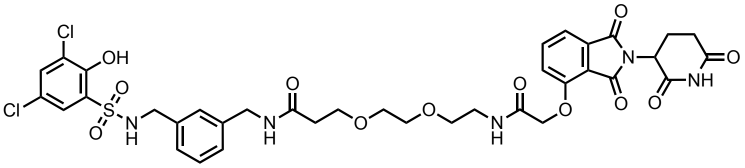

4c

N-(3-(((3,5-dichloro-2-hydroxyphenyl)sulfonamido)methyl)benzyl)-3-(2-(2-(2-((2-(2,6-dioxopiperidin-3-yl)-1,3-dioxoisoindolin-4-yl)oxy)acetamido)ethoxy)ethoxy)propenamide (4c)

General Procedure 1A. Reaction scale: Thalidomide-O-acetamido-PEG2-C2-acid 14 mg (28.5 µmol, 1 eq) and N-(3-(aminomethyl)benzyl)-3-chloro-2-hydroxybenzenesulfonamide (**F4**) 10.3 mg (28.5 µmol, 1 eq) Purified by pTLC (6% MeOH/DCM) to afford **4c** as a sticky solid (8.0 mg, 34%). ^{1}H NMR (600 MHz, $CDCl_3$) δ 8.93 (s, 1H), 7.74 (d, *J* = 7.9 Hz, 1H), 7.64 – 7.61 (m, 1H), 7.56 – 7.54 (m, 2H), 7.38 – 7.36 (m, 1H), 7.21 (s, 1H), 7.18 (d, *J* = 8.4 Hz, 1H), 7.11 – 7.00 (m, 3H), 6.89 (d, *J* = 7.1 Hz, 1H), 5.02 – 4.92 (m, 1H), 4.60 (s, 2H), 4.37 (d, *J* = 6.1 Hz, 2H), 4.10 (s, 2H), 3.83 – 3.77 (m, 2H), 3.73 – 3.65 (m, 2H), 3.63 – 3.58 (m, 2H), 3.54 – 3.50 (m, 2H), 3.39 – 3.35 (m, 2H), 2.88 – 2.84 (m, 2H), 2.77 – 2.74 (m, 2H), 2.59 – 2.48 (m, 2H), 2.16 – 2.14 (m, 1H), 1.46 – 1.44 (m, 2H). ^{13}C NMR (151 MHz, DMSO) δ 173.27, 170.58, 170.36, 167.38, 167.21, 165.91, 155.45, 139.85, 137.96, 137.40, 133.50, 133.30, 130.52, 128.50, 127.48, 126.88, 126.47, 126.42, 124.43, 121.48, 120.76, 117.19, 116.49, 70.01, 69.93, 69.28, 67.91, 67.30, 54.01, 49.26, 46.73, 42.38, 38.86, 36.57, 31.41, 22.47. HR-MS (ESI+) calc'd for $C_{36}H_{37}Cl_2N_5O_{12}S$: 833.1536, found $[M+H]^+$ 834.1595.

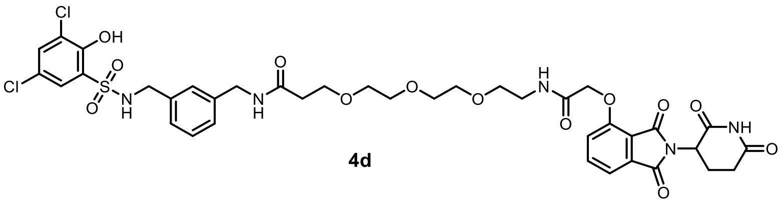

4d

N-(3-(((3,5-dichloro-2-hydroxyphenyl)sulfonamido)methyl)benzyl)-3-(2-(2-(2-(2-((2-(2,6-dioxopiperidin-3-yl)-1,3-dioxoisoindolin-4-yl)oxy)acetamido)ethoxy)ethoxy)ethoxy)propenamide (4d)

General Procedure 1A. Reaction scale: Thalidomide-O-acetamido-PEG3-C2-acid 9 mg (16.8 µmol, 1 eq) and N-(3-(aminomethyl)benzyl)-3-chloro-2-hydroxybenzenesulfonamide (**F4**) 8 mg (16.8 µmol, 1 eq) Purified by pTLC (6% MeOH/DCM) to afford **4d** as a brown sticky solid (4.3 mg, 30%). ^{1}H NMR (600 MHz, $CDCl_3$) δ 8.95 (s, 1H), 7.74 (d, *J* = 7.9 Hz, 1H), 7.66 (s, 1H), 7.61 – 7.48 (m, 2H), 7.37 (d, *J* = 2.5 Hz, 1H), 7.24 – 7.15 (m, 2H), 7.05 (d, *J* = 7.2 Hz, 2H), 6.88 (d, *J* = 6.8 Hz, 1H), 4.92 – 4.89 (m, 2H), 4.62 – 4.59 (m, 2H), 4.40 – 4.36 (m, 2H), 4.10 (s, 2H), 3.83 – 3.81 (m, 2H), 3.75 – 3.38 (m, 8H), 3.16 – 3.12 (m, 1H), 2.89 – 2.82 (m, 1H), 2.79 – 2.63 (m, 2H), 2.55 (t, *J* = 5.6 Hz, 2H), 2.16 – 2.09 (m, 1H), 1.48 – 1.43 (m, 2H). ^{13}C NMR (176 MHz, $CDCl_3$) δ 172.61, 171.57, 168.67, 167.41, 166.76, 166.16, 154.58, 139.29, 137.25, 136.12, 133.93, 133.70, 128.43, 127.48, 126.91, 126.88, 126.43, 119.73, 118.14, 117.60, 70.38, 70.24, 70.22, 70.19, 69.56, 68.03, 67.49, 49.37, 47.39, 43.19, 39.13, 37.07, 31.48, 29.85, 22.80. HR-MS (ESI+) calc'd for $C_{38}H_{41}Cl_2N_5O_{13}S$: 877.1799, found $[M+H]^+$ 878.1851.

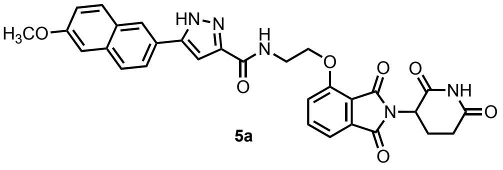

5a

N-(2-((2-(2,6-dioxopiperidin-3-yl)-1,3-dioxoisoindolin-4-yl)oxy)ethyl)-5-(6-methoxynaphthalen-2-yl)-1H-pyrazole-3-carboxamide (5a)

General Procedure 1A. Reaction scale: Thalidomide-O-C2-amine 30 mg (93.1 µmol, 1 eq) and 5-(6-methoxynaphthalen-2-yl)-1H-pyrazole-3-carboxylic acid 25 mg (93.1 µmol, 1.1 eq) Purified by pTLC (5% MeOH/DCM) to afford **5a** as a sticky solid (15.0 mg, 35%). ^{1}H NMR (600 MHz, DMSO) δ 11.12 (s, 1H), 8.36 – 8.28 (m, 2H), 7.98 – 7.77 (m, 4H), 7.63 (d, *J* = 8.5 Hz, 2H), 7.48 (d, *J* = 7.2 Hz, 2H), 7.36 (s, 1H), 7.27 – 7.07 (m, 1H), 5.08 – 5.14 (m, 1H), 4.42 – 4.38 (m, 2H), 3.90 (s, 3H), 3.73 – 3.70 (m, 2H), 2.92 – 2.88 (m, 2H), 2.65 – 2.52 (m, 2H). ^{13}C NMR (151 MHz, DMSO) δ 173.29, 170.44, 167.28, 165.74, 162.49, 158.22, 156.13, 148.02, 144.24, 137.54, 134.48, 133.76, 130.07, 128.85, 127.99, 124.40, 120.50, 119.91, 116.96, 116.02, 106.47, 103.08, 67.62, 55.73, 49.21, 38.08, 31.42, 22.47. HR-MS (ESI+) calc'd for $C_{30}H_{25}N_5O_7$: 567.1836, found $[M+H]^+$ 568.1828.

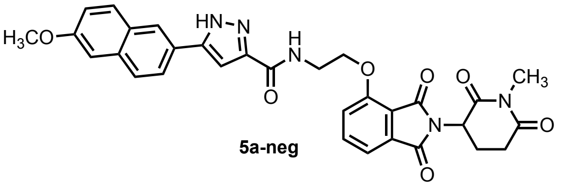

5a-neg

5-(6-methoxynaphthalen-2-yl)-N-(2-((2-(1-methyl-2,6-dioxopiperidin-3-yl)-1,3-dioxoisoindolin-4-yl)oxy)ethyl)-1H-pyrazole-3-carboxamide (5a-neg)

General Procedure 4. Reaction scale: Bifunctional molecule **5a** 15 mg (41.66 µmol, 1 eq) and methyl iodide 3 mg (83.3 µmol, 2 eq). Purified by pTLC (7% MeOH/DCM) to afford **5a-neg** as a sticky solid (6.0

mg, 41%). ^{1}H NMR (600 MHz, $CDCl_3$) δ 8.34 (d, *J* = 7.2 Hz, 1H), 7.99 (s, 1H), 7.69 (d, *J* = 8.6 Hz, 1H), 7.66 – 7.62 (m, 2H), 7.58 (d, *J* = 7.9 Hz, 1H), 7.38 (d, *J* = 7.3 Hz, 1H), 7.15 (d, *J* = 7.8 Hz, 2H), 7.12 – 7.10 (m, 1H), 7.07 – 7.05 (m, 1H), 4.90 – 4.86 (m, 1H), 4.34 – 4.30 (m, 2H), 3.89 (s, 3H), 3.74 – 3.68 (m, 2H), 3.09 (s, 3H), 2.86 – 2.75 (m, 1H), 2.64 – 2.58 (m, 2H), 2.00 – 1.83 (m, 1H). ^{13}C NMR (151 MHz, $CDCl_3$) δ 171.14, 168.97, 166.97, 166.89, 162.48, 158.27, 156.38, 136.88, 134.45, 133.44, 129.72, 128.67, 127.62, 124.46, 124.04, 119.68, 119.51, 117.14, 116.43, 105.68,103.36, 68.79, 55.37, 49.92, 38.59, 31.72, 27.23, 21.83. HR-MS (ESI+) calc'd for $C_{31}H_{27}N_5O_7$: 581.1910, found $[M+H]^+$ 582.1984.

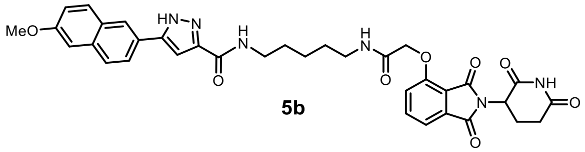

5b

N-(5-(2-((2-(2,6-dioxopiperidin-3-yl)-1,3-dioxoisoindolin-4-yl)oxy)acetamido)pentyl)-5-(6-methoxynaphthalen-2-yl)-1H-pyrazole-3-carboxamide (5b)

General Procedure 1A. Reaction scale: Thalidomide-O-acetamido-C5-acid 15 mg (36.02 µmol, 1 eq) and 5-(6-methoxynaphthalen-2-yl)-1H-pyrazole-3-carboxylic acid (**F5**) 10.6 mg (39.62 µmol, 1.1 eq) Purified by pTLC (8% MeOH/DCM) to afford **5b** as a white powder (8.2 mg, 34%). ^{1}H NMR (400 MHz, DMSO-d6) δ 11.19 (s, 1H), 8.37 (s, 1H), 8.31 (s, 1H), 8.05 (t, *J* = 5.6 Hz, 1H), 7.98 – 7.83 (m, 4H), 7.55 (d, *J* = 7.2 Hz, 1H), 7.49 – 7.39 (m, 2H), 7.34 – 7.19 (m, 2H), 5.20 (dd, *J* = 13.0, 5.4 Hz, 1H), 4.84 (s, 2H), 3.96 (s, 3H), 3.31 (q, *J* = 6.7 Hz, 2H), 3.23 (q, *J* = 7.2 Hz, 2H), 2.97 (ddd, *J* = 17.3, 14.0, 5.4 Hz, 1H), 2.70 – 2.60 (m, 2H), 2.19 – 2.05 (m, 1H), 1.66 – 1.49 (m, 4H), 1.48 – 1.33 (m, 2H). ^{13}C NMR (151 MHz, DMSO-d6) δ 172.84, 169.94, 166.77, 166.69, 165.54, 157.96 & 157.75 (amide rot.), 157.62, 155.09, 136.97, 133.96, 133.04, 129.61, 128.43, 127.42, 123.98, 123.73 (br), 123.69 (br), 120.38, 119.31, 116.81, 116.04, 106.02, 102.29 (br), 67.63, 55.29, 48.81, 38.43, 38.34, 30.97, 28.94, 28.75, 23.78, 22.01. *Note: Quaternary pyrazole carbons missing likely due to tautomerism/amide rotamers. Visible in $CDCl_3$ (147.59 & 145.09 ppm, see below).* HR-MS (ESI+) calc'd for $C_{35}H_{34}N_6O_8$: 666.2438, found $[M+H]^+$ 667.2512.

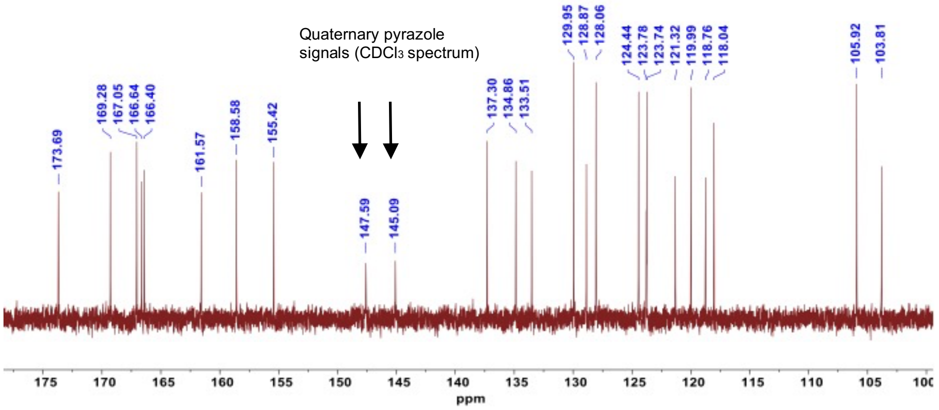

Spectrum of 5b (aromatic and carbonyl region) in $CDCl_3$ from a less pure sample (aliphatic impurities).

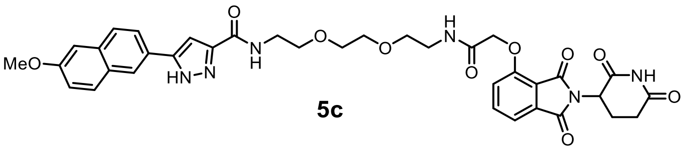

5c

N-(2-(2-(2-(2-((2-(2,6-dioxopiperidin-3-yl)-1,3-dioxoisoindolin-4-yl)oxy)acetamido)ethoxy)ethoxy)ethyl)-5-(6-methoxynaphthalen-2-yl)-1H-pyrazole-3-carboxamide (5c)

General Procedure 1A. Reaction scale: Thalidomide-O-acetamido-PEG2-C2-amine 15 mg (32.44 µmol, 1 eq) and 5-(6-methoxynaphthalen-2-yl)-1H-pyrazole-3-carboxylic acid (**F5**) 9.57 mg (35.68 µmol, 1.1 eq) Purified by pTLC (8% MeOH/DCM) to afford **5c** as a white powder (5.5 mg, 24%). ^{1}H NMR (400 MHz, DMSO-d6) δ 11.13 (s, 1H), 8.27 – 8.22 (m, 2H), 8.04 (t, *J* = 5.6 Hz, 1H), 7.96 – 7.83 (m, 3H), 7.79 (dd, *J* = 8.5, 7.3 Hz, 1H), 7.48 (d, *J* = 7.2 Hz, 1H), 7.41 – 7.32 (m, 2H), 7.28 – 7.08 (m, 2H), 5.11 (dd, *J* = 12.9, 5.3 Hz, 1H), 4.79 (s, 2H), 3.89 (s, 3H), 3.60 – 3.45 (m, 10H, partially under water peak), 3.32 (q, *J* = 5.7 Hz, 2H), 2.89 (ddd, *J* = 17.0, 14.0, 5.4 Hz, 1H), 2.64 – 2.53 (m, 2H), 2.10 – 1.97 (m, 1H). ^{13}C NMR (151 MHz, DMSO-d6) δ 173.28, 170.37, 167.38, 167.21, 165.90, 158.51 & 158.29 (amide rot.), 158.10, 155.45, 137.38, 134.43, 133.49, 130.06, 128.86, 127.89, 124.41, 124.19 (br, likely 2xC), 120.75, 119.78, 117.18, 116.47, 106.46, 102.82, 70.08, 70.00, 69.41, 69.32, 67.90, 55.73, 49.25, 38.88, 38.88, 31.41, 22.46. HR-MS (ESI+) calc'd for $C_{36}H_{36}N_{6}O_{10}$: 712.2493, found $[M+H]^{+}$ 713.2557.

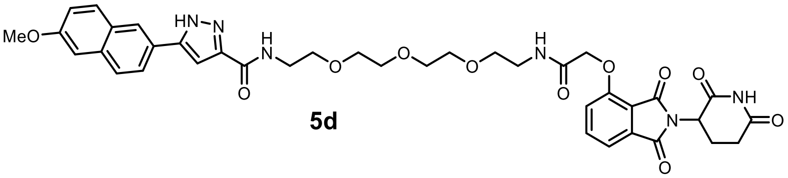

5d

N-(1-((2-(2,6-dioxopiperidin-3-yl)-1,3-dioxoisoindolin-4-yl)oxy)-2-oxo-6,9,12-trioxa-3-azatetradecan-14-yl)-5-(6-methoxynaphthalen-2-yl)-1H-pyrazole-3-carboxamide (5d)

General Procedure 1A. Reaction scale: Thalidomide-O-acetamido-PEG3-C2-amine 15 mg (29.6 µmol, 1 eq) and 5-(6-methoxynaphthalen-2-yl)-1H-pyrazole-3-carboxylic acid (**F5**) 8.7 mg (32.6 µmol, 1.1 eq) Purified by pTLC (8% MeOH/DCM) to afford **5d** as a white powder (13.58 mg, 62%). ^{1}H NMR (500 MHz, $CDCl_3$) δ 10.41 (s, 1H), 8.03 (d, *J* = 1.7 Hz, 1H), 7.75 (t, *J* = 8.8 Hz, 2H), 7.71 – 7.68 (m, 3H), 7.61 – 7.59 (m, 1H), 7.52 (d, *J* = 7.3 Hz, 1H), 7.19 – 7.13 (m, 2H), 7.11 (d, *J* = 8.4 Hz, 2H), 5.05 – 5.01 (m, 1H), 4.65 – 4.51 (m, 2H), 3.93 (s, 3H), 3.71 – 3.56 (m, 16H), 2.97 – 2.71 (m, 4H), 2.17 – 2.14 (m, 1H). ^{13}C NMR (151 MHz, CD_3OD_SPE) δ 174.63, 173.03, 171.48, 168.48, 167.90, 159.79, 157.60, 138.11, 136.05, 135.03, 130.70, 130.28, 128.78, 125.20, 124.94, 120.90, 120.61, 118.37, 116.95, 106.77, 103.42, 69.44, 61.55, 55.82, 54.81, 50.46, 39.60, 32.14, 23.62, 20.86, 14.45. HR-MS (ESI+) calc'd for $C_{38}H_{40}N_{6}O_{11}$: 756.2755, found $[M+H]^{+}$ 757.2830.

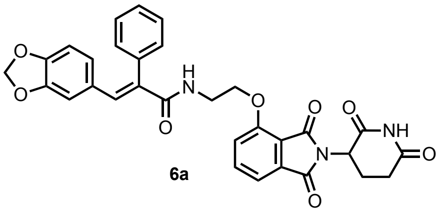

6a

(E)-3-(benzo[d][1,3]dioxol-5-yl)-N-(2-((2-(2,6-dioxopiperidin-3-yl)-1,3-dioxoisoindolin-4-yl)oxy)ethyl)-2-phenylacrylamide (6a)

General Procedure 1A. Reaction scale: Thalidomide-O-C2-amine 18 mg (55.9 μmol, 1 eq) and (E)-3-(benzo[d][1,3]dioxol-5-yl)-2-phenylacrylic acid 15 mg (55.9 μmol, 1 eq) Purified by pTLC (5% MeOH/DCM) to afford **6a** as an off-white solid (15.0 mg, 55%). ^{1}H NMR (600 MHz, DMSO) ^{1}H NMR (600 MHz, $CDCl_3$) δ 8.33 (s, 1H), 7.73 (s, 1H), 7.69 – 7.67 (m, 1H), 7.47 (d, *J* = 7.3 Hz, 1H), 7.42 – 7.37 (m, 3H), 7.25 (d, *J* = 8.5 Hz, 1H), 7.21 – 7.17 (m, 2H), 6.67 (dd, *J* = 8.1, 1.7 Hz, 1H), 6.62 (d, *J* = 8.2 Hz, 1H), 6.24 (d, *J* = 1.7 Hz, 1H), 6.17 (d, *J* = 5.9 Hz, 1H), 5.85 (s, 2H), 4.94 – 4.92 (m, 1H), 4.29 (t, *J* = 5.3 Hz, 2H), 3.82 – 3.63 (m, 2H), 2.95 – 2.69 (m, 4H), 2.12 – 2.10 (m, 1H).^{13}C NMR (151 MHz, DMSO) δ 171.04, 168.09, 166.92, 165.52, 156.18, 148.07, 147.39, 137.06, 136.70, 135.85, 133.75, 132.24, 129.85, 129.78, 128.63, 126.49, 119.50, 117.31, 116.41, 109.36, 108.15, 101.18, 67.92, 49.13, 39.44, 31.41, 22.62. HR-MS (ESI+) calc'd for $C_{31}H_{25}N_3O_8$: 567.1642, found $[M+H]^+$ 568.1713

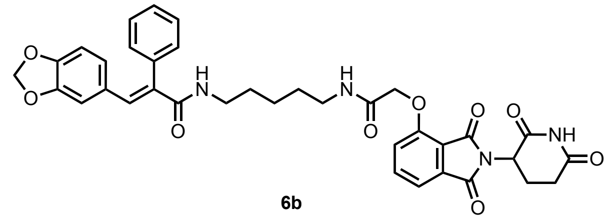

6b

(E)-3-(benzo[d][1,3]dioxol-5-yl)-N-(5-(2-((2-(2,6-dioxopiperidin-3-yl)-1,3-dioxoisoindolin-4-yl)oxy)acetamido)pentyl)-2-phenylacrylamide (6b)

General Procedure 1A. Reaction scale: Thalidomide-O-acetamido-C5-amine 14 mg (26.1 μmol, 1 eq) and (E)-3-(benzo[d][1,3]dioxol-5-yl)-2-phenylacrylic acid 7 mg (26.1 μmol, 1 eq). Purified by pTLC (5% MeOH/DCM) to afford **6b** as an off-white solid (14.0 mg, 64 %). ^{1}H NMR (600 MHz, CDCl3) δ 9.45 (s, 1H), 7.82 – 7.71 (m, 2H), 7.66 – 7.60 (m, 1H), 7.58 (d, J = 7.3 Hz, 1H), 7.51 – 7.44 (m, 3H), 7.30 – 7.19 (m, 3H), 6.73 (dd, J = 8.1, 1.7 Hz, 1H), 6.67 (d, J = 8.2 Hz, 1H), 6.48 – 6.45 (m, 1H), 6.26 (d, J = 1.6 Hz, 1H), 5.90 (s, 2H), 5.56 – 5.48 (m, 1H), 5.12 – 5.07 (m, 1H), 4.70 – 4.66 (m, 2H), 3.78 – 3.72 (m, 2H), 3.52 – 3.46 (m, 1H), 3.42 – 3.19 (m, 5H), 3.02 (s, 1H), 3.01 – 2.75 (m, 3H), 2.27 – 2.16 (m, 1H), 1.64 – 1.58 (m, 2H), 1.55 – 1.39 (m, 8H). ^{13}C NMR (151 MHz, $CDCl_3$) δ 171.49, 168.39, 167.53, 166.86, 166.67, 166.21, 154.57, 148.00, 137.10, 137.06, 136.20, 133.56, 132.15, 129.90, 129.80, 129.10, 128.69, 126.53, 119.91, 118.30, 117.49, 109.28, 108.22, 108.16, 101.15, 68.23, 55.64, 55.61, 49.35, 43.60, 40.21, 38.98, 31.45, 29.17, 28.72, 24.13, 22.81, 18.58, 17.17, 12.59. HR-MS (ESI$^+$) calc'd for $C_{36}H_{34}N_4O_9$: 666.6870, found $[M+H]^+$ 667.2391

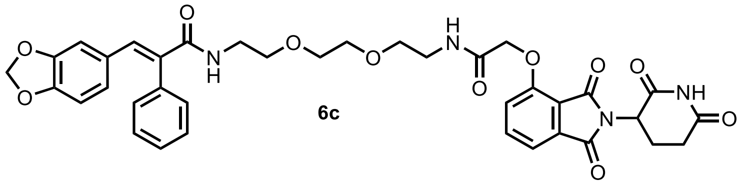

6c

(E)-3-(benzo[d][1,3]dioxol-5-yl)-N-(2-(2-(2-(2-((2-(2,6-dioxopiperidin-3-yl)-1,3-dioxoisoindolin-4-yl)oxy)acetamido)ethoxy)ethoxy)ethyl)-2-phenylacrylamide (6c)

General Procedure 1A. Reaction scale: Thalidomide-O-acetamido-PEG2-C2-amine 12.5 mg (25.0 μmol, 1 eq) and (E)-3-(benzo[d][1,3]dioxol-5-yl)-2-phenylacrylic acid 7 mg (25.0 μmol, 1 eq). Purified by pTLC (7% MeOH/DCM) to afford **6c** as an off-white solid (12.0 mg, 60 %).^{1}H NMR (600 MHz, $CDCl_3$) δ 8.97 (s, 1H), 7.83 – 7.75 (m, 1H), 7.70 – 7.68 (m, 1H), 7.60 (d, *J* = 7.3 Hz, 1H), 7.55 – 7.43 (m, 2H), 7.35 – 7.29 (m, 2H), 7.25 (d, *J* = 8.4 Hz, 1H), 6.73 (dd, *J* = 8.2, 1.7 Hz, 1H), 6.69 (d, *J* = 8.2 Hz, 1H), 6.28 (d, *J* = 1.6 Hz, 1H), 6.01 (d, *J* = 5.7 Hz, 1H), 5.92 (s, 1H), 5.08 – 4.95 (m, 1H), 4.71 (s, 1H), 3.78 (t, *J* = 7.4 Hz, 1H), 3.68 – 3.56 (m, 6H), 3.56 – 3.54 (m, 1H), 3.26 – 3.23 (m, 1H), 2.97 – 2.72 (m, 2H), 1.56 – 1.46 (m, 5H).^{13}C NMR

(151 MHz, $CDCl_3$) δ 171.20, 168.22, 167.64, 166.97, 166.67, 165.89, 154.40, 148.01, 147.36, 137.01, 136.16, 133.67, 132.30, 129.97, 129.68, 129.09, 128.58, 126.52, 119.35, 118.01, 117.33, 109.27, 108.15, 101.15, 70.29, 70.00, 69.77, 69.53, 67.87, 55.62, 49.31, 43.60, 39.91, 39.08, 31.41, 22.69, 18.62, 17.24, 12.56. HR-MS (ESI$^+$) calc'd for $C_{37}H_{36}N_4O_{11}$: 712.2381, found $[M+H]^+$ 713.2449.

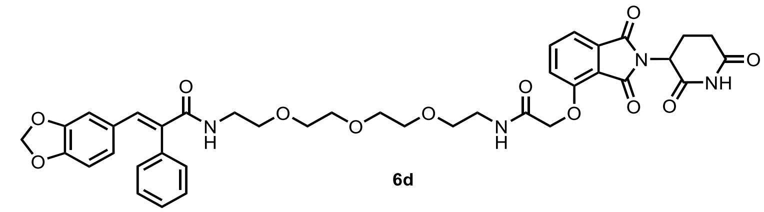

6d

(E)-3-(benzo[d][1,3]dioxol-5-yl)-N-(1-((2-(2,6-dioxopiperidin-3-yl)-1,3-dioxoisoindolin-4-yl)oxy)-2-oxo-6,9,12-trioxa-3-azatetradecan-14-yl)-2-phenylacrylamide (6d)

General Procedure 1A. Reaction scale: Thalidomide-O-acetamido-PEG3-C2-amine 11.5 mg (20.5 µmol, 1 eq) and (E)-3-(benzo[d][1,3]dioxol-5-yl)-2-phenylacrylic acid 6 mg (20.5 µmol, 1 eq). Purified by pTLC (7% MeOH/DCM) to afford **6d** as an off-white solid (8.0 mg, 52%).^{1}H NMR (600 MHz, CDCl3) δ 8.94 (s, 1H), 7.80 – 7.71 (m, 2H), 7.69 – 7.67 (m, 1H), 7.56 (d, J = 7.3 Hz, 1H), 7.49 – 7.38 (m, 3H), 7.33 – 7.24 (m, 3H), 7.22 (d, J = 8.4 Hz, 1H), 6.69 (dd, J = 8.2, 1.7 Hz, 1H), 6.64 (d, J = 8.1 Hz, 1H), 6.25 (d, J = 1.6 Hz, 1H), 6.01 (t, J = 5.2 Hz, 1H), 5.88 (s, 2H), 5.01 – 4.89 (m, 1H), 4.67 (s, 2H), 3.74 (s, 1H), 3.69 – 3.52 (m, 16H), 3.50 (q, J = 5.6 Hz, 2H), 3.20 (s, 1H), 2.92 – 2.65 (m, 3H), 2.21 – 2.09 (m, 1H), 1.54 – 1.40 (m, 7H). ^{13}C NMR (151 MHz, CDCl3) δ 171.16, 168.21, 166.64, 165.87, 147.96, 147.36, 137.04, 136.19, 132.49, 129.96, 129.64, 129.14, 128.51, 126.44, 119.44, 118.03, 117.34, 109.29, 108.14, 70.27, 70.23, 70.21, 70.19, 69.77, 69.51, 67.94, 55.57, 49.27, 43.60, 39.87, 39.12, 31.38, 22.69, 18.72, 17.34, 12.63. HR-MS (ESI$^+$) calc'd for $C_{39}H_{40}N_4O_{12}$: 756.2643, found $[M+H]^+$ 757.2720.

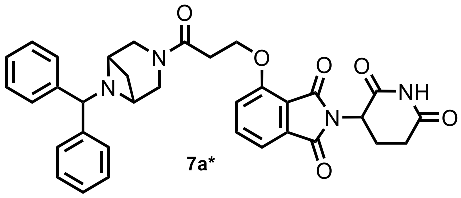

7a*

4-(3-(6-benzhydryl-3,6-diazabicyclo[3.1.1]heptan-3-yl)-3-oxopropoxy)-2-(2,6-dioxopiperidin-3-yl)isoindoline-1,3-dione (7a*)

General Procedure 1A. Reaction scale: Thalidomide-O-C2-acid 15 mg (43.6 µmol, 1 eq) of and 6-benzhydryl-3,6-diazabicyclo[3.1.1]heptane 20.0 mg (43.6 µmol, 1. eq) Purified by pTLC (7% MeOH/DCM) to afford **7a*** as an off-white solid (16 mg, 48%).^{1}H NMR (500 MHz, $CDCl_3$) δ 8.10 (s, 1H), 7.78 (d, *J* = 7.3 Hz, 1H), 7.68 – 7.50 (m, 5H), 7.33 (s, 10H), 4.98 – 4.94 (m, 1H), 4.80 (s, 1H), 4.67 – 4.63 (m, 3H), 3.84 (s, 1H), 3.64 – 3.60 (m, 5H), 3.07 (s, 1H), 3.04 – 2.89 (m, 3H), 2.89 – 2.70 (m, 4H), 2.27 – 2.12 (m, 1H), 1.70 (s, 5H). ^{13}C NMR (151 MHz, $CDCl_3$) δ 173.99, 171.10, 171.05, 168.22, 167.07, 165.74, 156.65, 136.56, 133.79, 128.72, 127.45, 127.30, 118.99, 117.11, 115.78, 69.14, 64.89, 53.47, 49.09, 44.46, 42.68, 38.63, 33.19, 31.39, 28.83, 25.71, 22.62. (*Note: rotameric mixture observed*), HR-MS (ESI$^+$) calc'd for $C_{34}H_{32}N_4O_6$: 592.2322, found $[M+H]^+$ 593.2398.

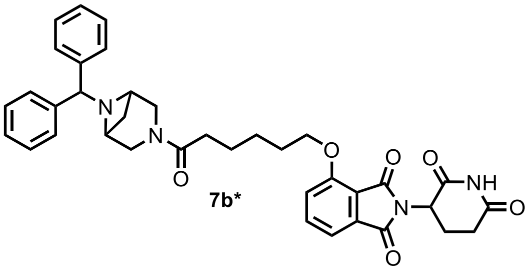

7b*

4-((6-(6-benzhydryl-3,6-diazabicyclo[3.1.1]heptan-3-yl)-6-oxohexyl)oxy)-2-(2,6-dioxopiperidin-3-yl)isoindoline-1,3-dione (7b*)

General Procedure 1A. Reaction scale: Thalidomide-O-C5-acid 12.7 mg (32.7 µmol, 1 eq) of and 6-benzhydryl-3,6-diazabicyclo[3.1.1]heptane 15.0 mg (32.7 µmol, 1. eq). Purified by pTLC (7% MeOH/DCM) to afford **7b*** as an off-white solid (15.0 mg, 52%). ^{1}H NMR (500 MHz, $CDCl_3$) δ 8.14 (s, 1H), 7.72 – 7.68 (m, 1H), 7.58 – 7.54 (m, 3H), 7.47 (d, J = 7.1 Hz, 1H), 7.31 – 7.19 (m, 7H), 4.97 – 4.94 (m, 1H), 4.75 (s, 1H), 4.26 – 4.23 (m, 2H), 3.79 – 3.60 (m, 4H), 3.61 – 3.42 (m, 2H), 3.20 – 3.14 (m, 1H), 2.95 – 2.65 (m, 4H), 2.44 – 2.40 (m, 2H), 2.12 – 2.08 (m, 1H), 1.98 – 1.96 (m, 2H), 1.86 – 1.84 (m, 3H), 1.66 – 1.62 (m, 3H), 1.55 – 1.49 (m, 3H), 1.49 – 1.38 (m, 4H). ^{13}C NMR (151 MHz, $CDCl_3$) δ 174.11, 171.13, 168.26, 167.08, 165.75, 156.67, 136.62, 133.75, 128.89, 128.77, 127.46, 127.33, 119.05, 117.06, 115.76, 69.15, 66.52, 64.84, 55.24, 53.47, 49.07, 44.41, 43.26, 42.63, 33.17, 31.37, 29.70, 28.79, 28.77, 28.49, 25.68, 24.35, 24.33, 22.61, 18.57, 17.19, 12.50. (*Note: rotameric mixture observed*) HR-MS (ESI$^+$) calc'd for $C_{37}H_{38}N_4O_6$: 634.2791, found [M+H]$^+$ 635.2862.

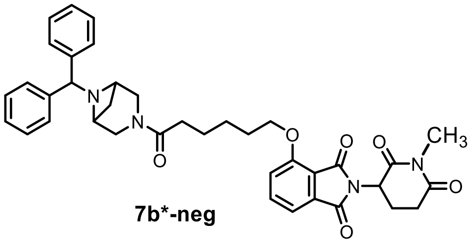

7b*-neg

4-((6-(6-benzhydryl-3,6-diazabicyclo[3.1.1]heptan-3-yl)-6-oxohexyl)oxy)-2-(2,6-dioxopiperidin-3-yl)isoindoline-1,3-dione (7b*-neg)

General Procedure 4. Reaction scale: bifunctional molecule **7b** 10 mg (15.8 µmol, 1.0 eq) of and 4.9 mg (31.6 µmol, 2.0 eq) Purified by pTLC (7% MeOH/DCM) to afford **7b*-neg** as an off-white solid (5 mg, 45%). ^{1}H NMR (600 MHz, $CDCl_3$) δ 7.85 (s, 2H), 7.69 – 7.67 (m, 1H), 7.47 – 7.45 (m, 3H), 7.32 – 7.03 (m, 6H), 5.02 – 4.87 (m, 1H), 4.67 – 6.63 (m, 1H), 4.28 – 4.13 (m, 2H), 3.73 – 3.60 (m, 1H), 3.56 – 3.54 (m, 1H), 3.48 – 3.46 (m, 1H), 3.36 – 3.34 (m, 1H), 3.21 – 3.18 (m, 2H), 3.00 – 2.90 (m, 1H), 2.83 – 2.61 (m, 2H), 2.39 – 2.36 (m, 1H), 2.11 – 1.99 (m, 1H), 1.96 – 1.92 (m, 1H), 1.88 – 1.75 (m, 2H), 1.63 – 1.61 (m 3H). ^{13}C NMR (151 MHz, DMSO) δ 173.00, 171.82, 169.73, 166.87, 165.36, 156.09, 142.25, 137.14, 133.25, 132.75, 129.65, 128.61, 127.21, 127.11, 119.86, 116.20, 115.22, 68.82, 49.33, 32.26, 31.14, 28.43, 26.64, 25.20, 24.08, 21.24. (*Note: rotameric mixture observed*) HR-MS (ESI$^+$) calc'd for $C_{38}H_{40}N_4O_6$: 648.2948, found [M+H]$^+$ 649.3021.

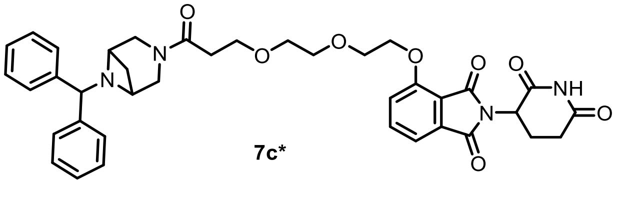

7c*

4-(2-(2-(3-(6-benzhydryl-3,6-diazabicyclo[3.1.1]heptan-3-yl)-3-oxopropoxy)ethoxy)ethoxy)-2-(2,6-dioxopiperidin-3-yl)isoindoline-1,3-dione (7c*)

General Procedure 1A. Reaction scale: Thalidomide-O-PEG2-acid 13.2 mg (32.7 μmol, 1 eq) of and 6-benzhydryl-3,6-diazabicyclo[3.1.1]heptane 15.0 mg (32.7 μmol, 1. eq). Purified by pTLC (7% MeOH/DCM) to afford **7c*** as an off-white solid (15.0 mg, 52%). ^{1}H NMR (400 MHz, pyridine-d5, 90°C) δ 12.36 (s, 1H), 7.73 (d, *J* = 7.6 Hz, 2H), 7.66 (d, *J* = 7.6 Hz, 2H), 7.66 – 7.57 (m, 1H), 7.52 (dd, *J* = 7.2, 0.8 Hz, 1H), 7.43 – 7.30 (m, 5H), 7.29 – 7.18 (m, 2H), 5.42 (dd, *J* = 12.4, 5.4 Hz, 1H), 4.99 (s, 1H), 4.50 – 4.41 (m, 2H), 4.10 (td, *J* = 6.5, 2.5 Hz, 2H, partially under water peak), 4.02 – 3.98 (m, 2H, partially under water peak), 3.93 – 3.77 (m, 6H), 3.67 – 3.59 (m, 1H), 3.57 (d, *J* = 6.0 Hz, 2H), 3.44 (d, *J* = 11.5 Hz, 1H), 3.04 (tdd, *J* = 12.3, 10.9, 7.0 Hz, 1H), 2.95 – 2.86 (m, 2H), 2.77 (td, *J* = 6.5, 2.6 Hz, 2H), 2.71 – 2.61 (m, 1H), 2.29 – 2.17 (m, 1H), 1.43 (d, *J* = 8.5 Hz, 1H). ^{13}C NMR (151 MHz, DMSO) δ 172.85, 171.35, 170.00, 166.86, 165.33, 155.86, 142.26, 137.04, 133.27, 128.58, 127.24, 127.07, 120.05, 116.34, 115.43, 70.21, 69.86, 68.94, 68.74, 66.72, 53.52, 48.79, 41.78, 32.94, 30.99, 22.03, 18.07, 16.74, 12.43. *Note: rotameric mixture observed.* HR-MS (ESI$^+$) calc'd for $C_{38}H_{40}N_4O_8$: 680.2846, found [M+H]$^+$ 681.2843.

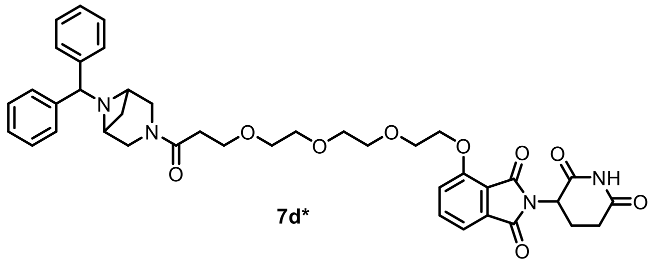

7d*

4-(2-(2-(2-(3-(6-benzhydryl-3,6-diazabicyclo[3.1.1]heptan-3-yl)-3-oxopropoxy)ethoxy)ethoxy)ethoxy)-2-(2,6-dioxopiperidin-3-yl)isoindoline-1,3-dione (7d*)

General Procedure 1A. Reaction scale: Thalidomide-O-PEG3-C2-acid 10.0 mg (21.8 μmol, 1.0 eq) of and 6-benzhydryl-3,6-diazabicyclo[3.1.1]heptane 10.0 mg (21.8 μmol, 1.0 0eq) Purified by pTLC (7% MeOH/DCM) to afford **7d*** as an off-white solid (10 mg, 50%). ^{1}H NMR (600 MHz, $CDCl_3$) δ 8.62 (s, 1H), 7.65 – 7.63 (m, 1H), 7.52 – 7.50 (m, 2H), 7.43 – 7.41 (m, 1H), 7.31 – 7.24 (m, 3H), 7.24 – 7.15 (m, 2H), 4.94 – 4.92 (m, 1H), 4.69 (s, 1H), 4.35 – 4.33 (s, 1H), 3.94 – 3.92 (m, 1H), 3.87 – 3.85 (m, 2H), 3.78 – 3.74 (m, 2H), 3.73 – 3.63 (m, 5H), 3.59 – 3.57 (m, 2H), 3.45 – 3.43 (m, 1H), 3.16 – 3.14 (m, 1H), 2.86 – 2.84 (m, 1H), 2.80 – 2.68 (m, 2H), 2.65 – 2.62 (m, 1H), 2.11 – 2.07 (m, 1H), 1.52 – 1.38 (m, 5H). ^{13}C NMR (151 MHz, $CDCl_3$) δ 172.52, 171.21, 171.19, 168.27, 167.00, 165.83, 156.34, 156.32, 136.54, 133.71, 133.69, 128.78, 128.69, 127.40, 127.39, 127.35, 119.39, 119.35, 117.40, 117.37, 116.19, 116.18, 71.10, 70.47, 70.39, 70.37, 70.33, 69.24, 69.23, 69.15, 69.10, 67.13, 67.09, 64.66, 55.38, 49.17, 49.15, 44.47, 43.38, 42.55, 33.67, 31.41, 29.71, 28.52, 22.63, 18.58, 17.21, 12.47. (*Note: rotameric mixture observed*) HR-MS (ESI$^+$) calc'd for $C_{40}H_{44}N_4O_9$: 724.3108, found [M+H]$^+$ 725.3179.

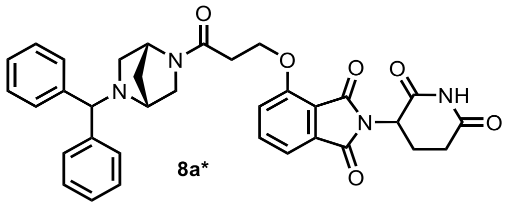

8a*

4-(3-((1R,4R)-5-benzhydryl-2,5-diazabicyclo[2.2.1]heptan-2-yl)-3-oxopropoxy)-2-(2,6-dioxopiperidin-3-yl)isoindoline-1,3-dione (8a)

General Procedure 1A. Reaction scale: Thalidomide-O-C2-acid 15 mg (43.6 μmol, 1 eq) and (1R,4R)-2-benzhydryl-2,5-diazabicyclo[2.2.1]heptane 20 mg (43.6 μmol, 1.0 eq). Purified by pTLC (7% MeOH/DCM) to afford **8a** as an off-white solid (8.0 mg, 40%). ^{1}H NMR (600 MHz, $CDCl_3$) δ 8.89 (s, 1H), 7.67 – 7.65 (m,

2H), 7.59 – 7.38 (m, 6H), 7.36 – 7.21 (m, 6H), 7.22 – 7.05 (m, 3H), 4.94 – 4.90 (m, 1H), 4.77 (s, 1H), 4.71 – 4.38 (m, 4H), 3.80 – 3.78 (s, 1H), 3.61 – 3.40 (m, 1H), 3.24 – 3.04 (m, 1H), 2.97 – 2.94 (s, 1H), 2.91 – 2.63 (m, 6H), 2.56 – 2.54 (m, 1H), 2.04 (s, 2H), 1.94 (s, 1H), 1.74 (s, 1H), 1.66 (s, 1H), 1.44 – 1.31 (m, 2H). ^{13}C NMR (151 MHz, $CDCl_3$) δ 171.57, 171.51, 168.51, 167.76, 167.52, 167.06, 167.02, 165.78, 165.68, 156.26, 156.13, 136.74, 136.71, 133.67, 133.64, 128.63, 127.74, 127.34, 127.30, 127.20, 119.24, 119.21, 117.06, 116.09, 116.06, 72.69, 71.99, 65.91, 65.83, 59.65, 59.37, 59.13, 57.99, 56.51, 55.4 (*Note: rotameric mixture observed*). HR-MS (ESI$^+$) calc'd for $C_{34}H_{32}N_4O_6$: 592.2322, found $[M+H]^+$ 593.2400.

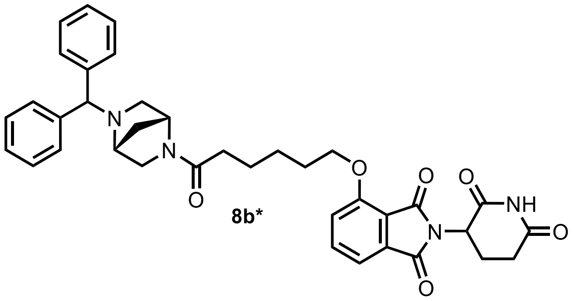

8b*

4-((6-((1R,4R)-5-benzhydryl-2,5-diazabicyclo[2.2.1]heptan-2-yl)-6-oxohexyl)oxy)-2-(2,6-dioxopiperidin-3-yl)isoindoline-1,3-dione (8b*)

General Procedure 1A. Reaction scale: Thalidomide-O-C5-acid 12.7 mg (32.7 µmol, 1.0 eq) and (1R,4R)-2-benzhydryl-2,5-diazabicyclo[2.2.1]heptane15 mg (32.7 µmol, 1.0 eq). Purified by pTLC (7% MeOH/DCM) to afford **8b** as an off-white solid (6 mg, 42%). ^{1}H NMR (600 MHz, $CDCl_3$) δ 8.18 (s, 1H), 7.72 – 7.63 (m, 1H), 7.51 – 7.40 (m, 4H), 7.31 – 7.24 (m, 4H), 7.24 – 7.14 (m, 2H), 4.98 – 4.94 (m, 1H), 4.76 (s, 1H), 4.63 (s, 1H), 4.59 – 4..54 (s, 1H), 4.27 (s, 1H), 4.23 – 4.20 (s, 2H), 3.81 – 3.79 (s, 1H), 3.66 – 3.64 (s, 1H), 3.52 – 3.48 (s, 1H), 3.25 – 3.22 (m, 1H), 3.19 – 3.15 (m, 1H), 3.00 – 2.92 (m, 1H), 2.92 – 2.67 (m, 3H), 2.62 – 2.57 (m, 1H), 2.47 – 2.39 (m, 1H), 2.40 – 2.33 (m, 1H), 2.33 – 2.24 (m, 1H), 2.15 – 2.05 (m, 1H), 2.06 – 1.99 (m, 1H), 1.99 – 1.88 (m, 2H), 1.79 – 1.69 (m, 3H), 1.69 – 1.45 (m, 3H). (*Note: rotameric mixture observed*) HR-MS (ESI$^+$) calc'd for $C_{37}H_{38}N_4O_6$: 634.7330, found $[M+H]^+$ 635.2867.

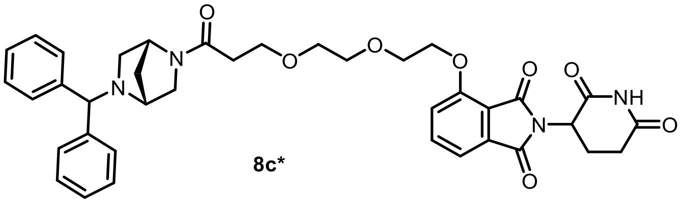

8c*

4-(2-(2-(3-((1R,4R)-5-benzhydryl-2,5-diazabicyclo[2.2.1]heptan-2-yl)-3-oxopropoxy)ethoxy)ethoxy)-2-(2,6-dioxopiperidin-3-yl)isoindoline-1,3-dione (8c*)

General Procedure 1A. Reaction scale: Thalidomide-O-PEG2-acid 14.2 mg (32.7 µmol, 1.0 eq) and (1R,4R)-2-benzhydryl-2,5-diazabicyclo[2.2.1]heptane 15 mg (32.7 µmol, 1.0 eq). Purified by pTLC (7% MeOH/DCM) to afford **8c** as an off-white solid (11.0 mg, 38%). ^{1}H NMR (600 MHz, $CDCl_3$) δ 8.80 (s, 1H), 7.66 – 7.62 (m, 2H), 7.53 – 7.34 (m, 9H), 7.31 – 7.19 (m, 9H), 7.15 – 7.12 (m, 3H), 7.06 (s, 1H), 4.92 – 4.88 (s, 2H), 4.70 (s, 1H), 4.30 – 4.26 (s, 4H), 3.92 – 3.88 (m, 2H), 3.84 – 3.74 (m, 6H), 3.69 – 3.63 (m, 6H), 3.60 – 3.56 (s, 2H), 3.24 – 3.20 (s, 1H), 3.15 – 3.12 (m, 3H), 2.85 – 2.64 (m, 6H), 2.64 – 2.40 (m, 6H), 2.07 – 2.05 (s, 2H), 1.46 – 1.31 (m, 6H). ^{13}C NMR (151 MHz, $CDCl_3$) δ 171.55, 168.63, 167.01, 165.78, 156.34, 136.66, 133.65, 128.70, 127.75, 127.33, 119.53, 119.51, 119.48, 117.19, 117.15, 116.14, 116.12, 70.95, 70.87, 70.42, 70.37, 69.25, 69.16, 67.34, 67.17, 59.16, 55.31, 49.10, 43.32, 35.15, 34.46, 31.33, 31.31, 29.69, 22.59, 22.57, 22.55, 18.49, 17.04, 12.62. (*Note: rotameric mixture observed*) HR-MS (ESI$^+$) calc'd for $C_{38}H_{40}N_4O_8$: 680.2846, found $[M+H]^+$ 681.2841.

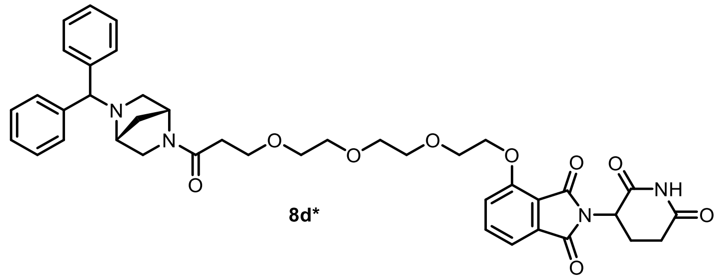

4-(2-(2-(2-(3-((1R,4R)-5-benzhydryl-2,5-diazabicyclo[2.2.1]heptan-2-yl)-3-oxopropoxy)ethoxy) ethoxy)ethoxy)-2-(2,6-dioxopiperidin-3-yl)isoindoline-1,3-dione (8d*)

General Procedure 1A. Reaction scale: Thalidomide-O-PEG3-acid 15.2 mg (32.7 µmol, 1.0 eq) and (1R,4R)-2-benzhydryl-2,5-diazabicyclo[2.2.1]heptane 15 mg (32.7 µmol, 1.0 eq). Purified by pTLC (7% MeOH/DCM) to afford **8d*** as an off-white solid (9.0 mg, 34%). ^{1}H NMR (600 MHz, CDCl3) δ 8.98 (s, 1H), 7.72 – 7.60 (m, 1H), 7.48 – 7.45 (m, 4H), 7.30 – 7.25 (m, 5H), 7.24 – 7.05 (m, 4H), 4.96 – 4.92 (m, 1H), 4.58 – 4.54 (m, 1H), 4.35 – 4.30 (m, 3H), 3.93 – 3.88 (m, 3H), 3.82 – 3.58 (m, 13H), 3.46 (s, 1H), 3.08 – 3.04 (m, 1H), 2.89 – 2.70 (m, 4H), 2.66 – 2.40 (m, 3H), 2.12 – 2.09 (m, 1H), 1.99 (s, 1H), 1.64 –1.62 (m, 1H). ^{13}C NMR (151 MHz, $CDCl_3$) δ ^{13}C NMR (151 MHz, $CDCl_3$) δ 171.66, 168.65, 167.02, 156.25, 136.74, 133.60, 128.69, 127.76, 127.37, 119.50, 117.36, 116.27, 70.86, 70.14, 69.15, 69.05, 67.12, 54.86, 49.18, 34.87, 31.39, 29.74, 22.61, 22.54, 18.59, 17.21. (*Note: rotameric mixture observed*) HR-MS (ESI^{+}) calc'd for $C_{40}H_{44}N_4O_9$: 724.3108, found [M+H]$^{+}$ 724.3108.

.

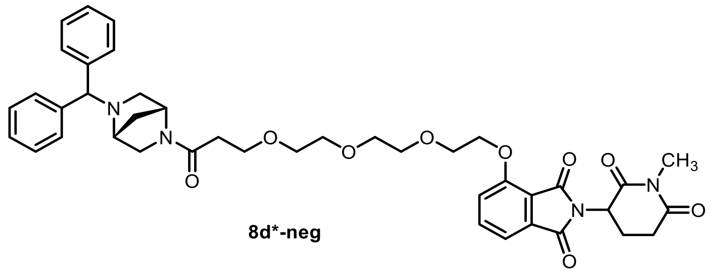

4-(2-(2-(2-(3-((1R,4R)-5-benzhydryl-2,5-diazabicyclo[2.2.1]heptan-2-yl)-3-oxopropoxy)ethoxy)ethoxy)ethoxy)-2-(1-methyl-2,6-dioxopiperidin-3-yl)isoindoline-1,3-dione (8d*-neg)

General Procedure 4. Reaction scale: bifunctional molecule **8d*** 14.5 mg (20.0 µmol,1 eq) and methyl iodide 7 mg (50.0 µmol, 2.5 eq). Purified by pTLC (6% MeOH/DCM) to afford **8d*-neg** as a sticky solid (8.0 mg, 54%). ^{1}H NMR (600 MHz, $CDCl_3$) δ 7.68 – 7.95 (m, 1H), 7.58 – 7.37 (m, 5H), 7.37 – 7.20 (m, 5H), 7.18 – 7.15 (m, 2H), 5.02 – 4.92 (m, 1H), 4.72 (s, 1H), 4.62 (s, 1H), 4.57 (s, 1H), 4.40 – 4.29 (m, 2H), 3.96 – 3.92 (m, 2H), 3.88 – 3.81 (m, 1H), 3.81 – 3.76 (m, 2H), 3.76 – 3.74 (m, 1H), 3.71 – 3.72 (m, 3H), 3.65 – 3.56 (m, 3H), 3.50 – 3.46 (m, 1H), 3.27 – 3.09 (m, 4H), 3.01 – 2.89 (m, 2H), 2.83 – 2.69 (m, 2H), 2.67 – 2.43 (m, 3H), 2.16 – 2.02 (m, 3H), 1.73 – 1.61 (m, 2H). ^{13}C NMR (151 MHz, $CDCl_3$) δ 171.24, 168.79, 168.73, 168.52, 167.16, 165.79, 156.40, 156.38, 143.98, 143.90, 143.13, 143.11, 136.46, 136.44, 133.79, 128.66, 128.55, 127.72, 127.65, 127.29, 127.22, 119.51, 119.41, 117.36, 116.15, 116.08, 72.74, 71.91, 71.87, 71.10, 70.56, 70.43, 69.35, 69.21, 67.36, 67.27, 59.20, 59.12, 59.06, 58.03, 56.30, 56.20, 51.14, 49.93, 49.78, 49.05, 36.37, 35.17, 34.51, 31.89, 27.30, 27.21, 21.92. (*Note: rotameric mixture observed*) HR-MS (ESI+) calc'd for $C_{41}H_{46}N_4O_9$: 738.3265, found [M+H]$^{+}$ 739.3343.

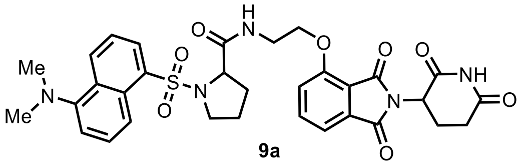

9a

1-((5-(dimethylamino)naphthalen-1-yl)sulfonyl)-N-(2-((2-(2,6-dioxopiperidin-3-yl)-1,3-dioxoisoindolin-4-yl)oxy)ethyl)pyrrolidine-2-carboxamide (9a)

General Procedure 1B. Reaction scale: ((5-(dimethylamino)naphthalen-1-yl)sulfonyl)proline (6.6 mg, 20 µmol, 1.0 eq.) was reacted with thalidomide-O-C2-amine (TFA salt, 9.1 mg, 21 µmol, 1.05 eq.). Purified by preparative HPLC (MeCN/H_2O gradient, 0.1% FA) to afford **9a** as a white solid (9.5 mg, 14.7 µmol, 73%). ^{1}H NMR (600 MHz, CD_3OD) (rotamers present) δ 8.58 – 8.52 (m, 1H), 8.48 – 8.43 (m, 1H), 8.24 – 8.17 (m, 1H), 7.76 (ddd, J = 8.4, 7.2, 1.0 Hz, 1H), 7.63 – 7.50 (m, 2H), 7.47 (dd, J = 7.2, 2.4 Hz, 1H), 7.40 – 7.33 (m, 1H), 7.23 – 7.16 (m, 1H), 5.19 – 5.05 (m, 1H), 4.34 – 4.24 (m, 1H), 4.16 – 3.97 (m, 2H), 3.65 – 3.57 (m, 1H), 3.57 – 3.42 (m, 2H), 3.37 – 3.32 (m, 1H), 2.92 – 2.82 (m, 1H), 2.85 (s, 6H), 2.79 – 2.63 (m, 2H), 2.17 – 2.07 (m, 1H), 2.01 – 1.82 (m, 3H), 1.77 – 1.68 (m, 1H). ^{13}C NMR (151 MHz, CD_3OD) δ 174.69, 174.66, 171.40, 168.49 & 168.46 (rot.), 167.72, 157.40, 153.18, 138.08, 135.01, 134.76 & 134.68 (rot.), 132.06 & 132.04 (rot.), 131.76 & 131.74 (rot.), 131.60 & 131.48 (rot.), 131.25, 129.57, 124.47 & 124.44 (rot.), 121.13 & 121.01 (rot.), 120.56 & 120.54 (rot.), 118.63 & 118.58 (rot.), 117.08 & 117.01 (rot.), 116.52, 68.84 & 68.74 (rot.), 63.27 & 63.25 (rot.), 50.45, 50.40 & 50.39 (rot.), 45.75, 39.51, 32.57 & 32.54 (rot.), 32.15, 25.67 & 25.65 (rot.), 23.68 & 23.66 (rot.). HR-MS (ESI+) calc'd for $[M+H]^+$ $C_{32}H_{34}N_5O_8S$: 648.2123, found 648.2121 (0.4 ppm).
.

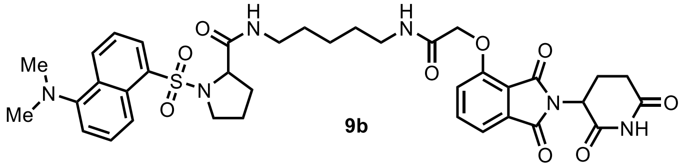

9b

1-((5-(dimethylamino)naphthalen-1-yl)sulfonyl)-N-(5-(2-((2-(2,6-dioxopiperidin-3-yl)-1,3-dioxoisoindolin-4-yl)oxy)acetamido)pentyl)pyrrolidine-2-carboxamide (9b)

General Procedure 1B. Reaction scale: ((5-(dimethylamino)naphthalen-1-yl)sulfonyl)proline (6.6 mg, 20 µmol, 1.0 eq.) was reacted with thalidomide-O-acetamido-C5-amine (TFA salt, 11.1 mg, 21 µmol, 1.05 eq.). Purified by preparative HPLC (MeCN/H_2O gradient, 0.1% FA) to afford **9b** as a white solid (11.2 mg, 15.0 µmol, 75%). ^{1}H NMR (600 MHz, CD_3OD) δ 8.59 – 8.54 (m, 1H), 8.42 (dd, *J* = 8.7, 2.6 Hz, 1H), 8.19 – 8.08 (m, 1H), 7.64 – 7.48 (m, 3H), 7.35 – 7.20 (m, 3H), 5.12 (dd, *J* = 12.8, 5.5 Hz, 1H), 4.72 (dd, *J* = 14.4, 2.5 Hz, 1H), 4.68 (dd, *J* = 14.3, 2.5 Hz, 1H), 4.20 (dd, *J* = 8.2, 4.0 Hz, 1H), 3.58 – 3.45 (m, 1H), 3.43 – 3.26 (m, 3H), 3.18 – 3.01 (m, 2H), 2.88 (s, 6H), 2.93 – 2.83 (m, 1H), 2.81 – 2.65 (m, 2H), 2.22 – 2.10 (m, 1H), 2.00 – 1.76 (m, 3H), 1.73 – 1.51 (m, 3H), 1.49 – 1.29 (m, 4H).^{13}C NMR (151 MHz, CD_3OD) δ 174.64, 174.27, 171.41, 169.94, 168.26, 167.71, 155.98, 153.23, 137.82, 134.64, 134.30 & 134.26 (rot.), 132.03, 131.75 & 131.63 (rot.), 131.56, 131.32, 129.57, 124.50, 121.38, 120.50, 119.21, 117.57, 116.62, 69.19, 63.31, 50.50, 50.28, 45.79, 40.20, 39.97, 32.30, 32.16, 29.94, 29.84, 25.61, 24.95, 23.68. HR-MS (ESI+) calc'd for $[M+H]^+$ $C_{37}H_{43}N_6O_9S$: 747.2807, found 747.2801 (0.9 ppm).

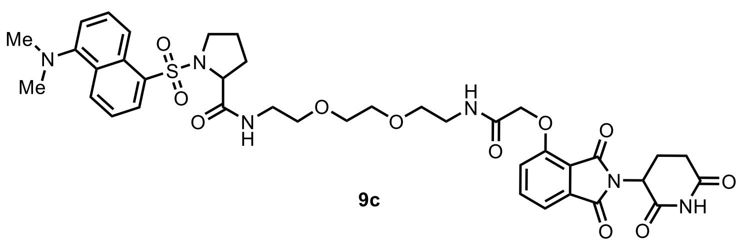

9c

1-((5-(dimethylamino)naphthalen-1-yl)sulfonyl)-N-(2-(2-(2-(2-((2-(2,6-dioxopiperidin-3-yl)-1,3-dioxoisoindolin-4-yl)oxy)acetamido)ethoxy)ethoxy)ethyl)pyrrolidine-2-carboxamide (9c)

General Procedure 1B. Reaction scale: ((5-(dimethylamino)naphthalen-1-yl)sulfonyl)proline (6.6 mg, 20 μmol, 1.0 eq.) was reacted with thalidomide-O-acetamido-PEG2-amine (HCl salt, 10.5 mg, 21 μmol, 1.05 eq.). The product was purified by preparative HPLC (MeCN/H_2O gradient, 0.1% FA) followed by preparative TLC (7% MeOH/DCM) to afford **9c** as a white solid (8.2 mg, 10.3 μmol, 52%). ^{1}H NMR (400 MHz, $CDCl_3$) δ 9.13 – 8.86 (m, 1H), 8.56 (dd, *J* = 8.5, 3.2 Hz, 1H), 8.52 (dd, *J* = 8.7, 2.6 Hz, 1H), 8.31 – 8.11 (m, 1H), 7.70 (t, *J* = 7.9 Hz, 1H), 7.65 (br s, 1H), 7.62 – 7.49 (m, 3H), 7.25 – 7.21 (m, 1H), 7.21 – 7.08 (m, 2H), 4.97 (dt, *J* = 12.1, 4.9 Hz, 1H), 4.64 (s, 2H), 4.35 – 4.27 (m, 1H), 3.79 – 3.24 (m, 14H), 2.88 (d, *J* = 1.2 Hz, 6H), 2.95 – 2.70 (m, 3H), 2.20 – 2.07 (m, 2H), 1.85 – 1.71 (m, 1H), 1.68 – 1.58 (m, 2H). *Note: shifted H_2O peak at 5.30 ppm integrating to 2H.* ^{13}C NMR (151 MHz, $CDCl_3$) δ 171.93 & 171.86 (rot.), 171.56 & 171.47 (rot.), 168.47 & 168.37 (rot.), 166.99 & 166.97 (rot.), 166.83, 165.95 & 165.92 (rot.), 154.55 & 154.54 (rot.), 151.98, 137.01, 133.81, 132.65 & 132.55 (rot.), 131.23 & 131.20 (rot.), 130.98 & 130.89 (rot.), 130.75 & 130.73 (rot.), 130.17, 128.78, 123.47 & 123.46 (rot.), 119.39 & 119.34 (rot.), 119.29, 118.17, 117.35 & 117.34 (rot.), 115.51, 70.34 & 70.30 (rot.), 70.21 & 70.16 (rot.), 69.78 & 69.67 (rot.), 69.63, 68.02 & 67.99 (rot.), 62.40 & 62.34 (rot.), 53.58, 49.41, 45.54, 39.41 & 39.37 (rot.), 39.26 & 39.20 (rot.), 31.56 & 31.53 (rot.), 30.79 & 30.72 (rot.), 24.62 & 24.58 (rot.), 22.84. HR-MS (ESI+) calc'd for $[M+H]^+$ $C_{38}H_{45}N_6O_{11}S$: 793.2862, found 793.2846 (2.1 ppm).

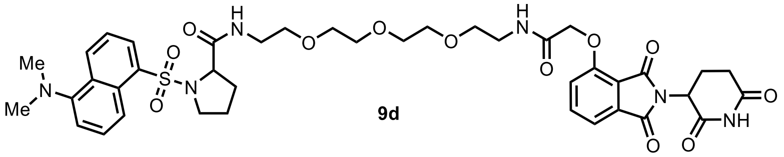

9d

1-((5-(dimethylamino)naphthalen-1-yl)sulfonyl)-N-(1-((2-(2,6-dioxopiperidin-3-yl)-1,3-dioxoisoindolin-4-yl)oxy)-2-oxo-6,9,12-trioxa-3-azatetradecan-14-yl)pyrrolidine-2-carboxamide (9d)

General Procedure 1B. Reaction scale: ((5-(dimethylamino)naphthalen-1-yl)sulfonyl)proline (6.6 mg, 20 μmol, 1.0 eq.) was reacted with thalidomide-O-acetamido-PEG3-amine (HCl salt, 11.4 mg, 21 μmol, 1.05 eq.). Purified by preparative HPLC (MeCN/H_2O gradient, 0.1% FA) to afford **9d** as a white solid (11.0 mg, 13.1 μmol, 66%). ^{1}H NMR (600 MHz, CD_3OD) δ 8.59 (d, *J* = 8.5 Hz, 1H), 8.47 (d, *J* = 8.7 Hz, 1H), 8.20 (dd, *J* = 7.3, 1.3 Hz, 1H), 7.74 (t, *J* = 7.9 Hz, 1H), 7.59 (t, *J* = 8.0 Hz, 2H), 7.48 (d, *J* = 7.3 Hz, 1H), 7.36 (d, *J* = 8.5 Hz, 1H), 7.27 (d, *J* = 7.5 Hz, 1H), 5.12 (dd, *J* = 12.9, 5.5 Hz, 1H), 4.73 (s, 2H), 4.32 – 4.23 (m, 1H), 3.70 – 3.59 (m, 8H), 3.58 – 3.47 (m, 5H), 3.46 – 3.35 (m, 3H), 3.29 – 3.22 (m, 1H), 3.21 – 3.12 (m, 1H), 2.87 (s, 6H), 2.91 – 2.82 (m, 1H), 2.80 – 2.67 (m, 2H), 2.19 – 2.09 (m, 1H), 1.97 – 1.81 (m, 3H), 1.76 – 1.63 (m, 1H). ^{13}C NMR (151 MHz, CD_3OD) δ 174.61, 174.38, 171.35, 169.97, 168.30, 167.55, 156.09, 153.23, 138.13, 134.90, 134.63, 132.03, 131.78, 131.50, 131.33, 129.60, 124.50, 121.44, 120.61, 119.15, 117.83, 116.62, 71.60, 71.57, 71.43, 71.34, 70.30, 70.24, 69.14, 63.15, 50.53, 50.32, 45.79, 40.28, 40.22, 32.37, 32.18, 25.65, 23.67. HR-MS (ESI+) calc'd for $[M+H]^+$ $C_{40}H_{49}N_6O_{12}S$: 837.3124, found 837.3110 (1.7 ppm).

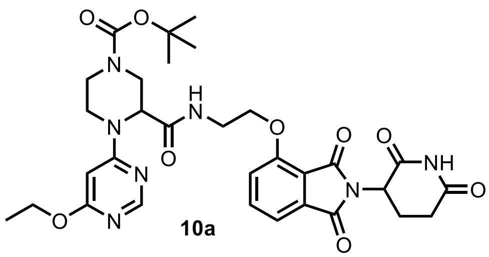

10a

***tert*-butyl 3-((2-((2-(2,6-dioxopiperidin-3-yl)-1,3-dioxoisoindolin-4-yl)oxy)ethyl)carbamoyl)-4-(6-ethoxypyrimidin-4-yl)piperazine-1-carboxylate (10a)**

General Procedure 1B. Reaction scale: 4-(*tert*-butoxycarbonyl)-1-(6-ethoxypyrimidin-4-yl)piperazine-2-carboxylic acid (7.0 mg, 20 μmol, 1.0 eq.) was reacted with thalidomide-O-C2-amine (TFA salt, 9.1 mg, 21 μmol, 1.05 eq.). Purified by preparative HPLC (MeCN/H_2O gradient, 0.1% FA) to afford **10a** as a white solid (10.7 mg, 16.4 μmol, 82%). ^1H NMR (600 MHz, CD_3OD) *(rotamers present)* δ 8.15 – 8.03 (br m, 1H), 7.86 – 7.70 (m, 1H), 7.47 (t, *J* = 7.6 Hz, 1H), 7.44 – 7.31 (m, 1H), 6.09 – 5.85 (m, 1H), 5.18 – 5.05 (m, 1H), 4.42 (br s, 1H), 4.21 (m, 4H), 3.88 – 3.75 (m, 2H), 3.76 – 3.57 (m, 3H), 3.56 – 3.31 (m, 3H), 2.89 (ddd, *J* = 17.6, 13.9, 5.4 Hz, 1H), 2.85 – 2.66 (m, 2H), 2.26 – 2.10 (m, 1H), 1.50 – 1.25 (m, 12H). ^{13}C NMR (151 MHz, CD_3OD) *(mostly rotameric multiplets, major intensity is reported)* δ 174.63, 173.44, 171.40, 168.44, 168.16, 168.00, 165.54, 158.11, 157.45, 156.39, 138.20, 134.97, 120.73, 118.27, 117.03, 87.18, 81.58, 69.25, 63.58, 58.43, 50.60, 44.83, 43.89, 42.12, 39.77, 32.19, 28.55, 23.72, 14.80. HR-MS (ESI+) calc'd for $[M+H]^+$ $C_{31}H_{38}N_7O_9$: 652.2726, found 652.2713 (2.0 ppm).

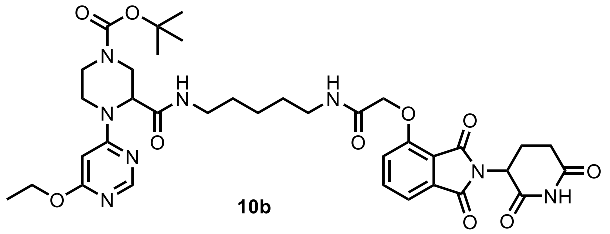

10b

***tert*-butyl 3-((5-(2-((2-(2,6-dioxopiperidin-3-yl)-1,3-dioxoisoindolin-4-yl)oxy)acetamido)pentyl)carbamoyl)-4-(6-ethoxypyrimidin-4-yl)piperazine-1-carboxylate (10b)**

General Procedure 1B. Reaction scale: 4-(*tert*-butoxycarbonyl)-1-(6-ethoxypyrimidin-4-yl)piperazine-2-carboxylic acid (7.0 mg, 20 μmol, 1.0 eq.) was reacted with thalidomide-O-acetamido-C5-amine (TFA salt, 11.1 mg, 21 μmol, 1.05 eq.). Purified by preparative HPLC (MeCN/H_2O gradient, 0.1% FA) to afford **10b** as a white solid (10.8 mg, 14.4 μmol, 72%). ^1H NMR (400 MHz, CD_3OD) δ 8.19 (br s, 1H), 7.81 (t, *J* = 7.9 Hz, 1H), 7.54 (d, *J* = 7.3 Hz, 1H), 7.43 (d, *J* = 8.4 Hz, 1H), 5.96 (br s, 1H), 5.23 – 5.09 (m, 1H), 4.77 (s, 2H), 4.40 (br s, 1H), 4.28 (q, *J* = 7.0 Hz, 2H), 3.93 – 3.76 (m, 2H), 3.71 – 3.58 (m, 1H), 3.52 – 3.31 (m, 2H), 3.31 – 3.09 (m, 4H), 2.98 – 2.83 (m, 1H), 2.82 – 2.61 (m, 2H), 2.25 – 2.07 (m, 1H), 1.67 – 1.22 (m, 18H). ^{13}C NMR (151 MHz, CD_3OD) δ 174.58, 172.83, 171.54, 171.40, 169.91, 168.30, 167.79, 165.78, 158.15, 156.31, 156.27, 138.23, 134.92, 121.77, 119.33, 117.96, 87.05, 81.57, 69.45, 63.58, 57.63 & 57.56 (rot.), 50.55, 45.43 (br), 44.17 (br), 42.25 (br), 40.47 (br), 40.02, 32.17, 30.09 (br multiplet), 29.87, 28.64, 25.10, 23.65, 14.86. HR-MS (ESI+) calc'd for $[M+H]^+$ $C_{36}H_{46}N_8O_{10}$: 751.3410, found 751.3395 (2.0 ppm).

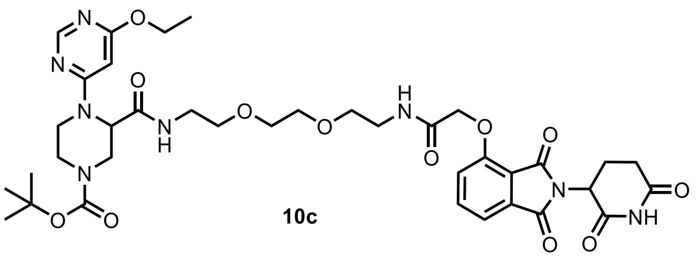

10c

***tert*-butyl 3-((2-(2-(2-(2-((2-(2,6-dioxopiperidin-3-yl)-1,3-dioxoisoindolin-4-yl)oxy)acetamido)ethoxy)ethoxy)ethyl)carbamoyl)-4-(6-ethoxypyrimidin-4-yl)piperazine-1-carboxylate (10c)**

General Procedure 1B. Reaction scale: 4-(*tert*-butoxycarbonyl)-1-(6-ethoxypyrimidin-4-yl)piperazine-2-carboxylic acid (7.0 mg, 20 μmol, 1.0 eq.) was reacted with thalidomide-O-acetamido-PEG2-amine (HCl salt, 10.5 mg, 21 μmol, 1.05 eq.). The product was purified by preparative HPLC (MeCN/H_2O gradient, 0.1% FA) followed by preparative TLC (7% MeOH/DCM) to afford **10c** as a white solid (8.0 mg, 10.0 μmol, 50%). ^{1}H NMR (400 MHz, $CDCl_3$) δ 10.02 – 8.93 (m, 1H), 8.30 (s, 1H), 7.81 – 7.71 (m, 1H), 7.65 (br s, 1H), 7.55 (d, *J* = 7.3 Hz, 1H), 7.19 (dd, *J* = 8.4, 2.6 Hz, 1H), 7.08 – 6.80 (m, 1H), 5.81 (s, 1H), 4.95 (s, 1H), 4.64 (s, 2H), 4.60 – 4.45 (m, 1H), 4.40 – 4.28 (m, 2H), 3.99 – 3.72 (m, 2H), 3.70 – 3.15 (m, 16H), 2.88 – 2.74 (m, 3H), 2.28 – 2.11 (m, 1H), 1.45 (s, 9H), 1.39 – 1.30 (m, 3H). ^{13}C NMR (151 MHz, $CDCl_3$) δ *(3 signals at coalescence or overlapping in 173-160 ppm region)* 170.33, 166.85, 168.84, 166.65, 165.89, 157.59 (br),154.75, 154.44, 137.03, 133.68, 119.32, 118.08, 117.35, 86.44, 80.52 (br), 69.98, 69.92, 69.74, 69.50, 67.88, 62.30 (br), 56.03 (br), 49.28, 49.23, 43.47, 41.55 (br), 39.49, 39.08, 31.41, 28.35, 22.72, 14.62. HR-MS (ESI+) calc'd for $[M+H]^+$ $C_{37}H_{49}N_8O_{12}$: 797.3465, found 797.3480 (1.9 ppm).

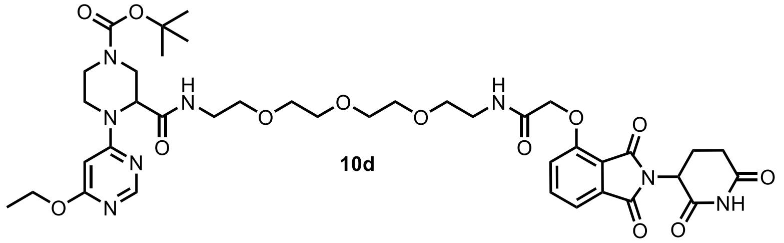

10d

***tert*-butyl 3-((1-((2-(2,6-dioxopiperidin-3-yl)-1,3-dioxoisoindolin-4-yl)oxy)-2-oxo-6,9,12-trioxa-3-azatetradecan-14-yl)carbamoyl)-4-(6-ethoxypyrimidin-4-yl)piperazine-1-carboxylate (10d)**

General Procedure 1B. Reaction scale: 4-(*tert*-butoxycarbonyl)-1-(6-ethoxypyrimidin-4-yl)piperazine-2-carboxylic acid (7.0 mg, 20 μmol, 1.0 eq.) was reacted with thalidomide-O-acetamido-PEG3-amine (HCl salt, 11.4 mg, 21 μmol, 1.05 eq.). The product was purified by preparative HPLC (MeCN/H_2O gradient, 0.1% FA) followed by preparative TLC (7% MeOH/DCM) to afford **10d** as a white solid (7.6 mg, 9.0 μmol, 45%). ^{1}H NMR (400 MHz, $CDCl_3$) δ 9.96 – 9.04 (m, 1H), 8.40 – 8.14 (m, 1H), 7.74 (t, *J* = 7.7 Hz, 1H), 7.70 (s, 1H), 7.55 (d, *J* = 7.3 Hz, 1H), 7.19 (d, *J* = 8.4 Hz, 1H), 7.13 – 6.95 (m, 1H), 5.80 (s, 1H), 4.95 (br s, 1H), 4.64 (s, 2H), 4.55 – 4.40 (m, 1H), 4.39 – 4.28 (m, 2H), 3.91 – 3.73 (m, 2H), 3.74 – 3.47 (m, 16H), 3.47 – 3.22 (m, 4H), 2.96 – 2.66 (m, 3H), 2.17 – 2.09 (m, 1H), 1.45 (s, 9H), 1.35 (t, *J* = 7.1 Hz, 3H). ^{13}C NMR (151 MHz, $CDCl_3$) δ 171.74 (br), 170.42 (br), 170.12 (br), 168.46 (br), 166.85, 166.77, 165.93, 164.02 (br), 157.61 (br), 154.89 (br), 154.50, 137.12, 133.83, 119.29, 118.15, 117.43, 86.57 (br), 80.47, 70.49, 70.47, 70.38, 70.30, 70.24, 69.58, 67.87, 62.41 (br), 56.38 (br), 49.40, 43.72 (br), 43.14 (br), 41.61 (br), 39.43, 39.22, 31.55, 28.50, 22.86, 14.76. HR-MS (ESI+) calc'd for $[M+H]^+$ $C_{39}H_{53}N_8O_{13}$: 841.3727, found 841.3711 (2.0 ppm).

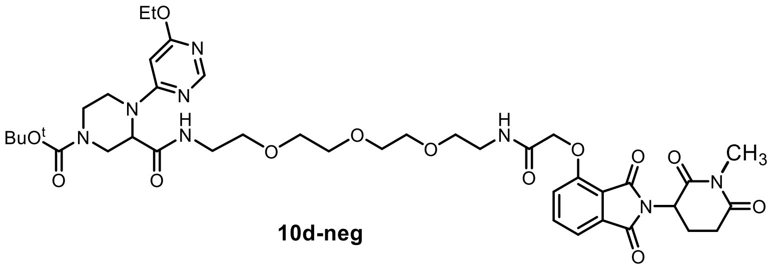

10d-neg

tert-butyl 4-(6-ethoxypyrimidin-4-yl)-3-((1-((2-(1-methyl-2,6-dioxopiperidin-3-yl)-1,3-dioxoisoindolin-4-yl)oxy)-2-oxo-6,9,12-trioxa-3-azatetradecan-14-yl)carbamoyl)piperazine-1-carboxylate (10d-neg)

General Procedure 4. Reaction scale: bifunctional molecule **10a** 8 mg (0.09 μmol, 1 eq) and methyl iodide 3.0 mg (0.18 μmol, 2.0 eq). Purified by pTLC (6% MeOH/DCM) to afford **10d-neg** as a sticky solid (3.0 mg, 42%). ^{1}H NMR (600 MHz, $CDCl_3$) δ 8.29 (s, 1H), 7.73 (d, *J* = 7.9 Hz, 1H), 7.60 (s, 1H), 7.55 (d, *J* = 7.3 Hz, 1H), 7.20 (d, *J* = 8.4 Hz, 1H), 7.03 (s, 1H), 6.81 (s, 1H), 5.82 (s, 1H), 4.98 – 4.95 (m, 2H), 4.66 (s, 1H), 4.48 (s, 1H), 4.33 (d, *J* = 7.1 Hz, 2H), 3.85 – 3.75 (m, 2H), 3.69 – 3.45 (m, 6H), 3.35 (s, 1H), 3.20 (s, 1H), 3.04 – 2.98 (m, 2H), 2.83 – 2.78 (m, 2H), 2.10 (d, *J* = 7.7 Hz, 1H), 1.65 (s, 3H), 1.50 – 1.52 (m, 4H), 1.45(s, 9H), 1.25 (s, 3H). ^{13}C NMR (151 MHz, $CDCl_3$) δ 171.13, 170.35, 170.00, 168.68, 167.11, 166.80, 166.01, 157.47, 154.75, 154.51, 136.99, 133.68, 119.51, 118.08, 117.35, 86.45, 80.31, 70.43, 70.26, 69.89, 69.56, 68.08, 62.26, 55.93, 50.03, 41.32, 39.40, 39.02, 31.95, 31.89, 29.72, 28.37, 27.30, 22.72, 21.94, 14.63, 14.15. HR-MS (ESI+) calc'd for $C_{40}H_{54}N_8O_{13}$: 854.9150, found $[M+H]^+$ 855.3881.

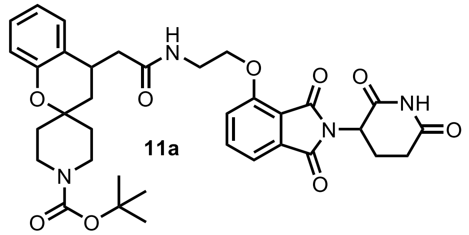

11a

***tert*-butyl 4-(2-((2-((2-(2,6-dioxopiperidin-3-yl)-1,3-dioxoisoindolin-4-yl)oxy)ethyl)amino)-2-oxoethyl)spiro[chromane-2,4'-piperidine]-1'-carboxylate (11a)**

General Procedure 1B. Reaction scale: 2-(1'-(*tert*-butoxycarbonyl)spiro[chromane-2,4'-piperidin]-4-yl)acetic acid (7.2 mg, 20 μmol, 1.0 eq.) was reacted with thalidomide-O-C2-amine (TFA salt, 9.1 mg, 21 μmol, 1.05 eq.). Purified by preparative HPLC (MeCN/H_2O gradient, 0.1% FA) to afford **11a** as a white solid (10.2 mg, 15.4 μmol, 77%). ^{1}H NMR (600 MHz, CD_3OD) (2 H's expected in water peak region) δ 7.80 (t, *J* = 7.8 Hz, 1H), 7.51 – 7.46 (m, 2H), 7.25 – 7.20 (m, 1H), 7.08 – 7.02 (m, 1H), 6.83 – 6.75 (m, 2H), 5.11 (dd, *J* = 12.9, 5.4 Hz, 1H), 4.41 – 4.24 (m, 2H), 3.84 – 3.60 (m, 4H), 3.40 – 3.32 (m, 2H), 3.02 (br s, 1H), 2.96 (ddd, *J* = 13.8, 5.0, 2.4 Hz, 1H), 2.92 – 2.82 (m, 1H), 2.78 – 2.62 (m, 2H), 2.21 (ddd, *J* = 13.3, 9.8, 2.9 Hz, 1H), 2.15 – 2.06 (m, 1H), 1.87 (ddd, *J* = 13.6, 6.3, 2.0 Hz, 1H), 1.74 (d, *J* = 13.8 Hz, 1H), 1.68 – 1.61 (m, 1H), 1.54 (ddd, *J* = 13.5, 11.7, 6.6 Hz, 1H), 1.45 (s, 9H), 1.35 (td, *J* = 12.8, 4.7 Hz, 1H). ^{13}C NMR (151 MHz, CD_3OD) δ 174.87 & 174.85 (rot.), 174.60, 171.30 & 171.26 (rot.), 168.39, 167.88, 157.65, 156.50, 154.06, 138.23, 135.12, 128.82, 128.12, 125.65, 121.65, 120.83, 118.57, 118.39, 117.03, 81.03, 73.88, 69.14, 50.47, 49.57, 42.88, 41.22 (br), 39.85, 39.64, 38.33 (br.), 32.68 (br.), 32.15, 29.59, 28.70, 23.66. HR-MS (ESI+) calc'd for $[M+H]^+$ $C_{35}H_{41}N_4O_9$: 661.2868, found 661.2863 (0.8 ppm).

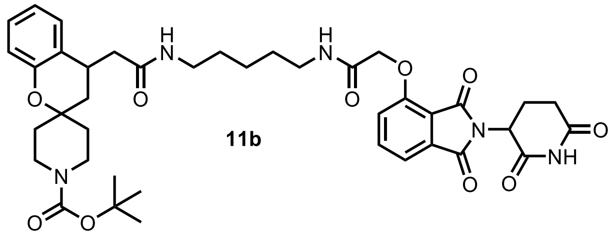

11b

***tert*-butyl 4-(2-((5-(2-((2-(2,6-dioxopiperidin-3-yl)-1,3-dioxoisoindolin-4-yl)oxy)acetamido)pentyl)amino)-2-oxoethyl)spiro[chromane-2,4'-piperidine]-1'-carboxylate (11b)**

General Procedure 1B. Reaction scale: 2-(1'-(*tert*-butoxycarbonyl)spiro[chromane-2,4'-piperidin]-4-yl)acetic acid (7.2 mg, 20 µmol, 1.0 eq.) was reacted with thalidomide-O-acetamido-C5-amine (TFA salt, 11.1 mg, 21 µmol, 1.05 eq.). Purified by preparative HPLC (MeCN/H_2O gradient, 0.1% FA) to afford **11b** as a white solid (9.8 mg, 12.9 µmol, 64%). ^{1}H NMR (600 MHz, CD_3OD) δ 7.81 (t, *J* = 7.9 Hz, 1H), 7.54 (d, *J* = 7.3 Hz, 1H), 7.43 (d, *J* = 8.5 Hz, 1H), 7.22 (dd, *J* = 7.8, 1.6 Hz, 1H), 7.08 (t, *J* = 7.6 Hz, 1H), 6.85 (t, *J* = 7.5 Hz, 1H), 6.80 (dd, *J* = 8.1, 1.3 Hz, 1H), 5.19 – 5.12 (m, 1H), 4.76 (s, 2H), 3.86 (d, *J* = 13.4 Hz, 1H), 3.76 (d, *J* = 13.4 Hz, 1H), 3.44 – 3.27 (m, 4H), 3.27 – 3.18 (m, 2H), 3.06 (br s, 1H), 2.97 – 2.84 (m, 2H), 2.80 – 2.68 (m, 2H), 2.19 (dd, *J* = 14.1, 9.2 Hz, 1H), 2.16 – 2.11 (m, 1H), 1.93 (ddd, *J* = 13.5, 6.3, 1.7 Hz, 1H), 1.79 (d, *J* = 13.9 Hz, 1H), 1.76 – 1.70 (m, 1H), 1.69 – 1.51 (m, 6H), 1.45 (s, 9H), 1.51 – 1.39 (m, 3H). ^{13}C NMR (151 MHz, CD_3OD) δ 174.59, 174.40, 171.43, 169.89, 168.28, 167.85, 156.58, 156.24, 154.08, 138.27, 134.90, 128.82, 128.22, 125.83, 121.75, 121.67, 119.33, 118.59, 118.01, 81.09, 73.93, 69.41, 50.55, 49.57, 42.90, 41.13 (br), 40.33, 40.00, 39.79, 38.36 (br), 32.74 (br), 32.17, 29.96 & 29.94 (rot.), 29.89, 29.38 & 29.37 (rot.), 28.69, 25.20 & 25.19 (rot.), 23.66. HR-MS (ESI+) calc'd for $[M+H]^+$ $C_{40}H_{50}N_5O_{10}$: 760.3552, found 760.3566 (1.9 ppm).

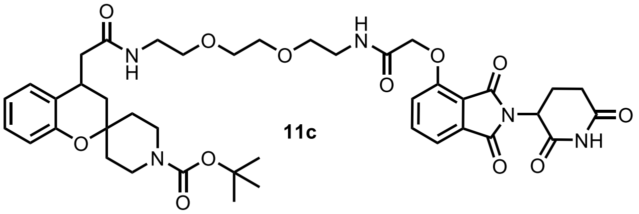

11c

***tert*-butyl 4-(14-((2-(2,6-dioxopiperidin-3-yl)-1,3-dioxoisoindolin-4-yl)oxy)-2,13-dioxo-6,9-dioxa-3,12-diazatetradecyl)spiro[chromane-2,4'-piperidine]-1'-carboxylate (11c)**

General Procedure 1B. Reaction scale: 2-(1'-(*tert*-butoxycarbonyl)spiro[chromane-2,4'-piperidin]-4-yl)acetic acid (7.2 mg, 20 µmol, 1.0 eq.) was reacted with thalidomide-O-acetamido-PEG2-amine (HCl salt, 10.5 mg, 21 µmol, 1.05 eq.). Purified by preparative HPLC (MeCN/H_2O gradient, 0.1% FA) to afford **11c** as a white solid (10.8 mg, 13.4 µmol, 67%). ^{1}H NMR (600 MHz, CD_3OD) δ 7.79 (t, *J* = 8.1 Hz, 1H), 7.53 (d, *J* = 7.3 Hz, 1H), 7.40 (d, *J* = 8.5 Hz, 1H), 7.21 (d, *J* = 7.8 Hz, 1H), 7.07 (t, *J* = 7.6 Hz, 1H), 6.86 – 6.82 (m, 1H), 6.79 (d, *J* = 8.5 Hz, 1H), 5.16 – 5.10 (m, 1H), 4.73 (s, 2H), 3.86 (d, *J* = 13.4 Hz, 1H), 3.76 (d, *J* = 13.3 Hz, 1H), 3.68 – 3.61 (m, 6H), 3.55 (t, *J* = 5.4 Hz, 2H), 3.51 (t, *J* = 5.4 Hz, 2H), 3.38 (t, *J* = 5.4 Hz, 2H), 3.38 – 3.31 (m, 2H), 3.06 (s, 1H), 2.92 – 2.83 (m, 2H), 2.79 – 2.67 (m, 2H), 2.20 (dd, *J* = 14.1, 9.4 Hz, 1H), 2.17 – 2.11 (m, 1H), 1.94 (dd, *J* = 13.5, 6.2 Hz, 1H), 1.78 (d, *J* = 13.9 Hz, 1H), 1.71 (d, *J* = 13.7 Hz, 1H), 1.64 (td, *J* = 12.6, 4.7 Hz, 1H), 1.53 (dd, *J* = 13.6, 11.7 Hz, 1H), 1.49 – 1.42 (m, 1H), 1.45 (s, 9H). ^{13}C NMR (151 MHz, CD_3OD) δ 174.58 (2 C's), 171.35, 169.98, 168.28, 167.68, 156.56, 156.14, 154.07, 138.23, 134.93, 128.81, 128.20, 125.81, 121.69, 121.55, 119.19, 118.59, 117.92, 81.07, 73.93, 71.48, 71.32, 70.59, 70.39, 69.18, 50.54, 49.57, 42.79, 41.13 (br), 40.44, 40.16, 39.71, 38.37 (br), 32.78 (br), 32.17, 29.35, 28.69, 23.66. HR-MS (ESI+) calc'd for $[M+H]^+$ $C_{41}H_{52}N_5O_{12}$: 806.3607, found 806.3629 (2.8 ppm).

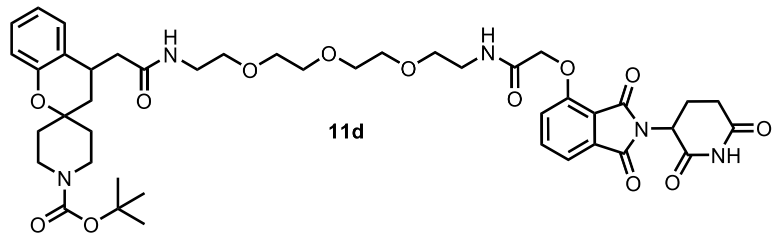

11d

***tert*-butyl 4-(17-((2-(2,6-dioxopiperidin-3-yl)-1,3-dioxoisoindolin-4-yl)oxy)-2,16-dioxo-6,9,12-trioxa-3,15-diazaheptadecyl)spiro[chromane-2,4'-piperidine]-1'-carboxylate (11d)**

General Procedure 1B. Reaction scale: 2-(1'-(*tert*-butoxycarbonyl)spiro[chromane-2,4'-piperidin]-4-yl)acetic acid (7.2 mg, 20 µmol, 1.0 eq.) was reacted with thalidomide-O-acetamido-PEG3-amine (HCl salt, 11.4 mg, 21 µmol, 1.05 eq.). Purified by preparative HPLC (MeCN/H_2O gradient, 0.1% FA) to afford **11d** as a white solid (11.1 mg, 13.1 µmol, 65%). ^{1}H NMR (600 MHz, CD_3OD) δ 7.80 (t, *J* = 7.9 Hz, 1H), 7.53 (d, *J* = 7.3 Hz, 1H), 7.41 (d, *J* = 8.5 Hz, 1H), 7.22 (d, *J* = 7.8 Hz, 1H), 7.06 (t, *J* = 7.8 Hz, 1H), 6.84 (t, *J* = 7.5 Hz, 1H), 6.81 – 6.76 (m, 1H), 5.12 (dd, *J* = 12.9, 5.2 Hz, 1H), 4.74 (s, 2H), 3.86 (d, *J* = 13.4 Hz, 1H), 3.76 (d, *J* = 13.5 Hz, 1H), 3.68 – 3.57 (m, 10H), 3.54 (t, *J* = 5.4 Hz, 2H), 3.48 (t, *J* = 5.7 Hz, 2H), 3.39 (t, *J* = 5.4 Hz, 2H), 3.37 – 3.32 (m, 2H), 3.06 (s, 1H), 2.95 – 2.83 (m, 2H), 2.81 – 2.67 (m, 2H), 2.22 (dd, *J* = 14.1, 9.3 Hz, 1H), 2.18 – 2.10 (m, 1H), 1.94 (dd, *J* = 13.5, 6.3 Hz, 1H), 1.79 (d, *J* = 13.9 Hz, 1H), 1.75 – 1.69 (m, 1H), 1.65 (td, *J* = 12.7, 4.8 Hz, 1H), 1.55 (t, *J* = 12.6 Hz, 1H), 1.50 – 1.41 (m, 1H), 1.45 (s, 9H). ^{13}C NMR (151 MHz, CD_3OD) δ 174.56 (2 Cs), 171.32, 169.92, 168.28, 167.60, 156.55, 156.14, 154.07, 138.22, 134.95, 128.81, 128.21, 125.82, 121.69, 121.53, 119.18, 118.59, 117.90, 81.06, 73.94, 71.65, 71.57, 71.38, 71.25, 70.54, 70.30, 69.15, 50.54, 49.57, 42.78, 41.03 (br), 40.40, 40.15, 39.72, 38.38 (br), 32.79 (br), 32.18, 29.37, 28.69, 23.66. HR-MS (ESI+) calc'd for $[M+H]^+$ $C_{43}H_{56}N_5O_{13}$: 850.3869, found 850.3862 (0.9 ppm).

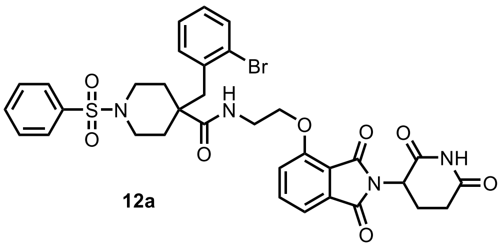

12a

4-(2-bromobenzyl)-N-(2-((2-(2,6-dioxopiperidin-3-yl)-1,3-dioxoisoindolin-4-yl)oxy)ethyl)-1-(phenylsulfonyl)piperidine-4-carboxamide (12a)

General Procedure 1B. Reaction scale: 4-(2-bromobenzyl)-1-(phenylsulfonyl)piperidine-4-carboxylic acid (8.8 mg, 20 µmol, 1.0 eq.) was reacted with thalidomide-O-C2-amine (TFA salt, 9.1 mg, 21 µmol, 1.05 eq.). Purified by preparative HPLC (MeCN/H_2O gradient, 0.1% FA) to afford **12a** as a white solid (10.0 mg, 13.6 µmol, 68%). ^{1}H NMR (600 MHz, CD_3OD) δ 7.82 (dd, *J* = 8.5, 7.3 Hz, 1H), 7.66 – 7.61 (m, 2H), 7.54 (d, *J* = 7.2 Hz, 1H), 7.44 – 7.30 (m, 5H), 7.13 (dd, *J* = 7.7, 1.9 Hz, 1H), 7.09 (td, *J* = 7.4, 1.3 Hz, 1H), 6.96 (ddd, *J* = 7.9, 7.1, 1.9 Hz, 1H), 5.13 (dd, *J* = 12.7, 5.5 Hz, 1H), 4.03 (t, *J* = 5.1 Hz, 2H), 3.68 – 3.56 (m, 2H), 3.53 – 3.41 (m, 2H), 2.98 (s, 2H), 2.89 (ddd, *J* = 17.1, 13.7, 5.3 Hz, 1H), 2.82 – 2.66 (m, 2H), 2.35 – 2.22 (m, 4H), 2.19 – 2.12 (m, 1H), 1.77 – 1.67 (m, 2H). ^{13}C NMR (151 MHz, CD_3OD) δ 175.44, 174.63, 171.27, 168.41, 168.06, 157.47, 138.34, 137.35, 137.19, 135.03, 134.02, 133.84, 133.36, 130.04, 129.60, 128.60, 128.24, 126.61, 120.73, 118.26, 117.10, 68.97, 50.54, 47.79, 45.97, 45.06 (2 Cs), 39.95, 33.97, 33.90, 32.16, 23.72. HR-MS (ESI+) calc'd for $[M+H]^+$ $C_{34}H_{34}BrN_4O_8S$: 661.2868, found 661.2863 (0.4 ppm).

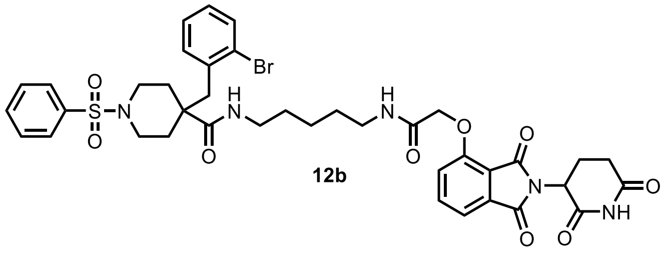

12b

4-(2-bromobenzyl)-N-(5-(2-((2-(2,6-dioxopiperidin-3-yl)-1,3-dioxoisoindolin-4-yl)oxy)acetamido)pentyl)-1-(phenylsulfonyl)piperidine-4-carboxamide (12b)

General Procedure 1B. Reaction scale: 4-(2-bromobenzyl)-1-(phenylsulfonyl)piperidine-4-carboxylic acid (8.8 mg, 20 µmol, 1.0 eq.) was reacted with thalidomide-O-acetamido-C5-amine (TFA salt, 11.1 mg, 21 µmol, 1.05 eq.). Purified by preparative HPLC (MeCN/H_2O gradient, 0.1% FA) to afford **12b** as a white solid (7.8 mg, 9.3 µmol, 47%). ^{1}H NMR (600 MHz, CD_3OD) δ 7.81 (dd, *J* = 8.5, 7.3 Hz, 1H), 7.75 – 7.67 (m, 2H), 7.66 – 7.61 (m, 1H), 7.60 – 7.53 (m, 3H), 7.51 (dd, *J* = 8.4, 1.3 Hz, 1H), 7.43 (d, *J* = 8.4 Hz, 1H), 7.21 (td, *J* = 7.4, 1.3 Hz, 1H), 7.13 – 7.04 (m, 2H), 5.17 (dd, *J* = 12.8, 5.5 Hz, 1H), 4.76 (s, 2H), 3.63 – 3.53 (m, 2H), 3.29 – 3.22 (m, 2H), 3.11 – 2.98 (m, 2H), 2.93 (s, 2H), 2.87 (ddd, *J* = 18.1, 14.4, 5.4 Hz, 1H), 2.80 – 2.67 (m, 2H), 2.29 (t, *J* = 12.1 Hz, 2H), 2.22 – 2.11 (m, 3H), 1.64 (ddd, *J* = 14.9, 12.7, 4.1 Hz, 2H), 1.50 (p, *J* = 7.0 Hz, 2H), 1.31 – 1.24 (m, 2H), 1.23 – 1.15 (m, 2H). ^{13}C NMR (151 MHz, CD_3OD) δ 175.18, 174.58, 171.48, 169.88, 168.28, 167.88, 156.18, 138.31, 137.44, 136.98, 134.89, 134.23, 134.09, 133.38, 130.33, 129.67, 128.80, 128.28, 126.66, 121.77, 119.33, 118.03, 69.40, 50.55, 47.76, 45.78, 45.08, 45.06, 40.50, 39.99, 33.57 (3 Cs), 32.16, 29.88, 29.85, 25.21, 23.66. HR-MS (ESI+) calc'd for $[M+H]^+$ $C_{39}H_{43}BrN_5O_9S$: 836.1959, found 836.1974 (1.8 ppm).

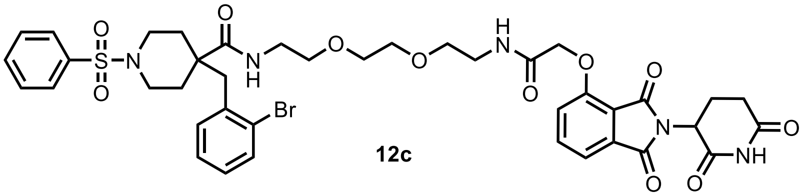

12c

4-(2-bromobenzyl)-N-(2-(2-(2-(2-((2-(2,6-dioxopiperidin-3-yl)-1,3-dioxoisoindolin-4-yl)oxy)acetamido)ethoxy)ethoxy)ethyl)-1-(phenylsulfonyl)piperidine-4-carboxamide (12c)

General Procedure 1B. Reaction scale: 4-(2-bromobenzyl)-1-(phenylsulfonyl)piperidine-4-carboxylic acid (8.8 mg, 20 µmol, 1.0 eq.) was reacted with thalidomide-O-acetamido-PEG2-amine (HCl salt, 10.5 mg, 21 µmol, 1.05 eq.). Purified by preparative HPLC (MeCN/H_2O gradient, 0.1% FA) to afford **12c** as a white solid (14.1 mg, 16.0 µmol, 61%). ^{1}H NMR (400 MHz, CD_3OD) δ 7.79 (dd, *J* = 8.4, 7.3 Hz, 1H), 7.73 – 7.64 (m, 2H), 7.65 – 7.59 (m, 1H), 7.59 – 7.46 (m, 4H), 7.40 (d, *J* = 8.4 Hz, 1H), 7.21 (td, *J* = 7.4, 1.3 Hz, 1H), 7.11 (dd, *J* = 7.8, 1.8 Hz, 1H), 7.07 (td, *J* = 7.6, 1.8 Hz, 1H), 5.14 (dd, *J* = 12.6, 5.5 Hz, 1H), 4.74 (s, 2H), 3.77 – 3.52 (m, 6H), 3.53 – 3.42 (m, 4H), 3.30 – 3.28 (m, 2H), 3.21 (q, *J* = 5.3 Hz, 2H), 2.93 (s, 2H), 2.92 – 2.82 (m, 1H), 2.80 – 2.64 (m, 2H), 2.35 (td, *J* = 12.4, 2.2 Hz, 2H), 2.18 – 2.08 (m, 3H), 1.71 – 1.56 (m, 2H). ^{13}C NMR (151 MHz, CD_3OD) δ 174.05, 173.19, 169.99, 168.62, 166.89, 166.27, 154.69, 136.83, 136.12, 135.99, 133.51, 132.72, 132.65, 132.04, 128.96, 128.27, 127.32, 126.95, 125.25, 120.16, 117.79, 116.52, 70.04, 69.77, 68.91 (2 Cs), 67.77, 49.14, 46.38, 44.28, 43.56 (2 Cs), 39.11, 38.81, 32.24 (2 Cs), 30.78, 22.28. HR-MS (ESI+) calc'd for $[M+H]^+$ $C_{40}H_{45}BrN_5O_{11}S$: 882.2014, found 882.2009 (0.6 ppm).

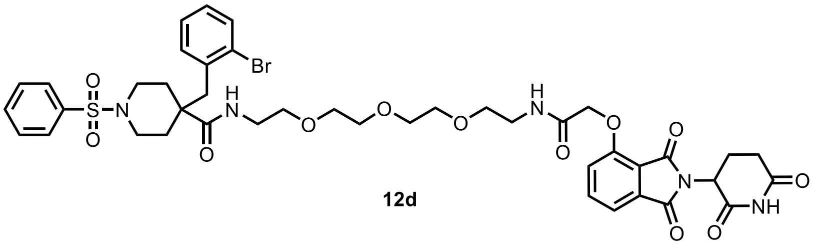

12d

4-(2-bromobenzyl)-N-(1-((2-(2,6-dioxopiperidin-3-yl)-1,3-dioxoisoindolin-4-yl)oxy)-2-oxo-6,9,12-trioxa-3-azatetradecan-14-yl)-1-(phenylsulfonyl)piperidine-4-carboxamide (12d)

General Procedure 1B. Reaction scale: 4-(2-bromobenzyl)-1-(phenylsulfonyl)piperidine-4-carboxylic acid (8.8 mg, 20 µmol, 1.0 eq.) was reacted with thalidomide-O-acetamido-PEG3 amine (HCl salt, 11.4 mg, 21 µmol, 1.05 eq.). Purified by preparative HPLC (MeCN/H_2O gradient, 0.1% FA) to afford **12d** as a white solid (11.2 mg, 12.1 µmol, 61%). ^1H NMR (400 MHz, CD_3OD) δ 7.79 (dd, *J* = 8.4, 7.3 Hz, 1H), 7.73 – 7.64 (m, 2H), 7.65 – 7.59 (m, 1H), 7.59 – 7.46 (m, 4H), 7.40 (d, *J* = 8.4 Hz, 1H), 7.21 (td, *J* = 7.4, 1.3 Hz, 1H), 7.11 (dd, *J* = 7.8, 1.8 Hz, 1H), 7.07 (td, *J* = 7.6, 1.8 Hz, 1H), 5.14 (dd, *J* = 12.6, 5.5 Hz, 1H), 4.74 (s, 2H), 3.77 – 3.52 (m, 6H), 3.53 – 3.42 (m, 4H), 3.30 – 3.28 (m, 2H), 3.21 (q, *J* = 5.3 Hz, 2H), 2.93 (s, 2H), 2.92 – 2.82 (m, 1H), 2.80 – 2.64 (m, 2H), 2.35 (td, *J* = 12.4, 2.2 Hz, 2H), 2.18 – 2.08 (m, 3H), 1.71 – 1.56 (m, 2H). ^{13}C NMR (151 MHz, CD_3OD) δ 174.03, 173.18, 169.96, 168.57, 166.91, 166.20, 154.72, 136.81, 136.01 (2 Cs), 133.54, 132.72, 132.67, 132.03, 128.96, 128.28, 127.36, 126.94, 125.25, 120.11, 117.80, 116.50, 70.20, 70.16, 70.04, 69.73, 68.91, 68.85, 67.75, 49.14, 46.39, 44.33, 43.60 (2 Cs), 39.09, 38.81, 32.30, 32.28, 30.78, 22.27. HR-MS (ESI+) calc'd for $[M+H]^+$ $C_{42}H_{49}BrN_5O_{12}S$: 926.2276, found 926.2282 (0.7 ppm).

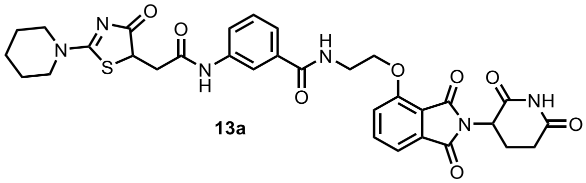

13a

N-(2-((2-(2,6-dioxopiperidin-3-yl)-1,3-dioxoisoindolin-4-yl)oxy)ethyl)-3-(2-(4-oxo-2-(piperidin-1-yl)-4,5-dihydrothiazol-5-yl)acetamido)benzamide (13a)

General Procedure 1B. Reaction scale: 3-(2-(4-oxo-2-(piperidin-1-yl)-4,5-dihydrothiazol-5-yl)acetamido)benzoic acid (7.2 mg, 20 µmol, 1.0 eq.) was reacted with thalidomide-O-C2-amine (TFA salt, 9.1 mg, 21 µmol, 1.05 eq.). Purified by preparative HPLC (MeCN/H_2O gradient, 0.1% FA) to afford **13a** as a white solid (11.8 mg, 17.9 µmol, 89%). ^1H NMR (600 MHz, CD_3OD) δ 8.00 (dt, *J* = 3.8, 1.9 Hz, 1H), 7.76 (dd, *J* = 8.5, 7.3 Hz, 1H), 7.71 – 7.65 (m, 1H), 7.53 (dt, *J* = 7.8, 1.3 Hz, 1H), 7.48 (d, *J* = 8.5 Hz, 1H), 7.45 (d, *J* = 7.3 Hz, 1H), 7.39 (t, *J* = 7.9 Hz, 1H), 5.11 (ddd, *J* = 12.7, 5.5, 1.5 Hz, 1H), 4.56 (dd, *J* = 10.8, 3.6 Hz, 1H), 4.41 (t, *J* = 5.3 Hz, 2H), 3.94 – 3.80 (m, 2H), 3.83 (t, *J* = 5.3 Hz, 2H), 3.59 – 3.47 (m, 2H), 3.38 (dd, *J* = 16.6, 3.6 Hz, 1H), 2.91 – 2.78 (m, 2H), 2.77 – 2.64 (m, 2H), 2.18 – 2.08 (m, 1H), 1.85 – 1.58 (m, 6H). ^{13}C NMR (151 MHz, CD_3OD) δ 191.39, 181.77, 174.60, 171.48, 170.70, 170.17, 168.45, 167.99, 157.65, 140.00, 138.16, 136.36, 135.05, 130.12, 124.02, 123.83, 121.05, 120.03, 118.41, 117.02, 69.34, 53.15, 51.42, 50.75, 50.47, 41.07, 40.40, 32.16, 26.98, 26.58, 24.79, 23.64. HR-MS (ESI+) calc'd for $[M+H]^+$ $C_{32}H_{33}N_6O_8S$: 661.2075, found 661.2078 (0.5 ppm).

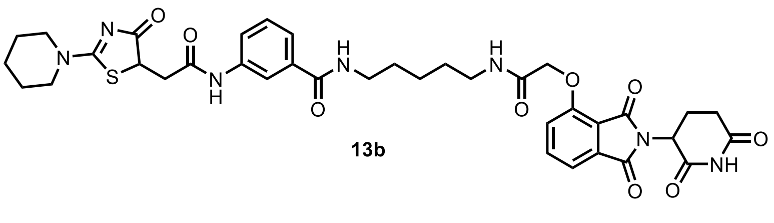

13b

N-(5-(2-((2-(2,6-dioxopiperidin-3-yl)-1,3-dioxoisoindolin-4-yl)oxy)acetamido)pentyl)-3-(2-(4-oxo-2-(piperidin-1-yl)-4,5-dihydrothiazol-5-yl)acetamido)benzamide (13b)

General Procedure 1B. Reaction scale: 3-(2-(4-oxo-2-(piperidin-1-yl)-4,5-dihydrothiazol-5-yl)acetamido)benzoic acid (7.2 mg, 20 µmol, 1.0 eq.) was reacted with thalidomide-O-acetamido-C5-amine (TFA salt, 11.1 mg, 21 µmol, 1.05 eq.). Purified by preparative HPLC (MeCN/H_2O gradient, 0.1% FA) to afford **13b** as a white solid (12.7 mg, 16.7 µmol, 84%). ^1H NMR (600 MHz, CD_3OD) δ 7.97 (t, *J* = 1.9 Hz, 1H), 7.77 (t, *J* = 7.9 Hz, 1H), 7.60 – 7.53 (m, 1H), 7.53 – 7.43 (m, 2H), 7.38 (d, *J* = 8.4 Hz, 1H), 7.32 (td, *J* = 7.9, 3.1 Hz, 1H), 5.13 (dd, *J* = 12.9, 5.5 Hz, 1H), 4.73 (s, 2H), 4.57 (dd, *J* = 10.8, 3.7 Hz, 1H), 4.01 – 3.81 (m, 2H), 3.61 – 3.51 (m, 2H), 3.48 – 3.33 (m, 5H), 3.00 – 2.63 (m, 4H), 2.14 (td, *J* = 7.1, 3.4 Hz, 1H), 1.87 – 1.60 (m, 10H), 1.48 (p, *J* = 7.8 Hz, 2H). ^{13}C NMR (151 MHz, DMSO-d_6) δ 187.65, 179.26, 172.82, 169.93, 168.84, 166.76, 166.67 & 166.59 (rot.), 166.04 & 165.96 (rot.), 165.52, 155.07, 138.85, 136.95, 135.51, 133.04, 128.64, 121.70, 121.51, 120.37, 118.38, 116.80, 116.05, 67.60, 54.95, 51.65, 49.27, 48.80, 48.77, 38.31, 38.18, 30.96, 30.73, 28.77 & 28.74 & 28.73 (rot.), 25.58, 25.10, 23.78, 23.35, 22.01. HR-MS (ESI+) calc'd for $[M+H]^+$ $C_{37}H_{42}N_7O_{11}S$: 760.2759, found 760.2756 (0.4 ppm).

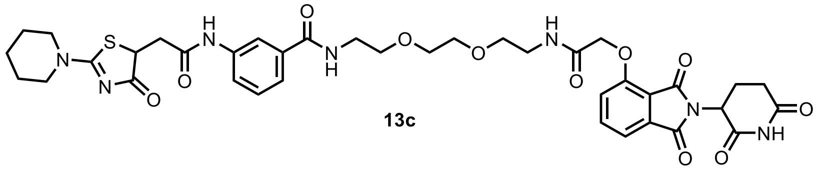

13c

N-(2-(2-(2-(2-((2-(2,6-dioxopiperidin-3-yl)-1,3-dioxoisoindolin-4-yl)oxy)acetamido)ethoxy)ethoxy)ethyl)-3-(2-(4-oxo-2-(piperidin-1-yl)-4,5-dihydrothiazol-5-yl)acetamido)benzamide (13c)

General Procedure 1B. Reaction scale: 3-(2-(4-oxo-2-(piperidin-1-yl)-4,5-dihydrothiazol-5-yl)acetamido)benzoic acid (7.2 mg, 20 µmol, 1.0 eq.) was reacted with thalidomide-O-acetamido-PEG2-amine (HCl salt, 10.5 mg, 21 µmol, 1.05 eq.). Purified by preparative HPLC (MeCN/H_2O gradient, 0.1% FA) to afford **13c** as a white solid (10.6 mg, 13.2 µmol, 66%). ^1H NMR (600 MHz, DMSO-d_6) δ 11.12 (s, 1H), 10.31 (s, 1H), 8.54 – 8.42 (m, 1H), 8.02 (t, *J* = 1.9 Hz, 1H), 8.01 (s, 1H), 7.80 (dd, *J* = 8.5, 7.3 Hz, 1H), 7.70 (d, *J* = 7.7 Hz, 1H), 7.49 (t, *J* = 7.5 Hz, 2H), 7.41 – 7.34 (m, 2H), 5.11 (dd, *J* = 12.9, 5.4 Hz, 1H), 4.78 (s, 2H), 4.44 (dd, *J* = 11.2, 3.5 Hz, 1H), 3.84 (dt, *J* = 11.5, 5.5 Hz, 1H), 3.77 (dt, *J* = 12.5, 5.6 Hz, 1H), 3.58 – 3.49 (m, 6H), 3.46 (t, *J* = 5.9 Hz, 4H), 3.39 (q, *J* = 5.9 Hz, 2H), 3.36 – 3.25 (m, 9H), 2.89 (ddd, *J* = 16.9, 13.9, 5.5 Hz, 1H), 2.72 (dd, *J* = 16.6, 11.2 Hz, 1H), 2.66 – 2.51 (m, 2H), 2.04 (ddd, *J* = 14.2, 6.3, 3.9 Hz, 1H), 1.76 – 1.49 (m, 4H). ^{13}C NMR (151 MHz, DMSO-d_6) (*1 C expected under solvent*) δ 187.65, 179.27, 172.82, 169.92, 168.86, 166.92, 166.76, 166.20, 165.45, 155.00, 138.90, 136.94, 135.21, 133.04, 128.68, 121.72, 121.64, 120.31, 118.40, 116.74, 116.04, 69.61, 69.57, 68.87, 68.85, 67.46, 54.95, 51.65, 49.27, 48.80, 48.77, 38.42, 30.96, 25.58, 25.10, 23.35, 22.01. HR-MS (ESI+) calc'd for $[M+H]^+$ $C_{38}H_{44}N_7O_{11}S$: 806.2814, found 806.2832 (2.3 ppm).

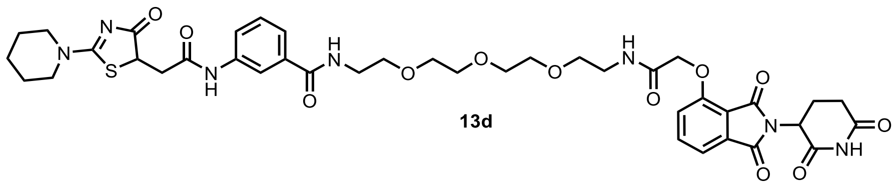

13d

N-(1-((2-(2,6-dioxopiperidin-3-yl)-1,3-dioxoisoindolin-4-yl)oxy)-2-oxo-6,9,12-trioxa-3-azatetradecan-14-yl)-3-(2-(4-oxo-2-(piperidin-1-yl)-4,5-dihydrothiazol-5-yl)acetamido)benzamide (13d)

General Procedure 1B. Reaction scale: 3-(2-(4-oxo-2-(piperidin-1-yl)-4,5-dihydrothiazol-5-yl)acetamido)benzoic acid (7.2 mg, 20 µmol, 1.0 eq.) was reacted with thalidomide-O-acetamido-PEG3-

amine (HCl salt, 11.4 mg, 21 μmol, 1.05 eq.). Purified by preparative HPLC (MeCN/H_2O gradient, 0.1% FA) to afford **13d** as a white solid (11.7 mg, 13.8 μmol, 69%). ^{1}H NMR (600 MHz, CD_3OD) δ 8.00 (t, *J* = 1.9 Hz, 1H), 7.78 (t, *J* = 7.9 Hz, 1H), 7.65 – 7.61 (m, 1H), 7.53 – 7.48 (m, 2H), 7.42 – 7.34 (m, 2H), 5.11 (ddd, *J* = 12.8, 5.6, 1.7 Hz, 1H), 4.73 (s, 2H), 4.56 (ddd, *J* = 10.9, 3.6, 1.3 Hz, 1H), 3.95 – 3.82 (m, 2H), 3.68 – 3.51 (m, 16H), 3.46 (t, *J* = 5.4 Hz, 2H), 3.38 (dd, *J* = 16.6, 3.2 Hz, 1H), 2.94 – 2.66 (m, 5H), 2.20 – 2.09 (m, 1H), 1.79 – 1.60 (m, 6H). ^{13}C NMR (151 MHz, CD_3OD) δ 191.35, 181.77, 174.61, 171.41, 170.70, 169.93, 169.91, 168.29, 167.56, 156.11, 139.98, 138.21, 136.54, 134.90, 130.10, 123.82 (3 Cs)z, 121.46, 119.87, 119.10, 117.86, 71.59, 71.57, 71.36, 71.33, 70.51, 70.27, 69.10, 53.14, 51.42, 50.75, 50.55, 41.09, 41.01, 40.19, 32.19, 26.99, 26.59, 24.80, 23.67. HR-MS (ESI+) calc'd for $[M+H]^+$ $C_{40}H_{48}N_7O_{12}S$: 850.3076, found 850.3073 (0.4 ppm).

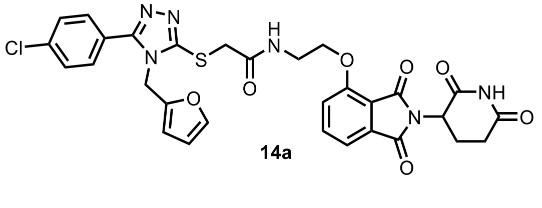

14a

2-((5-(4-chlorophenyl)-4-(furan-2-ylmethyl)-4H-1,2,4-triazol-3-yl)thio)-N-(2-((2-(2,6-dioxopiperidin-3-yl)-1,3-dioxoisoindolin-4-yl)oxy)ethyl)acetamide (14a)

General Procedure 1B. Reaction scale: 2-((5-(4-chlorophenyl)-4-(furan-2-ylmethyl)-4H-1,2,4-triazol-3-yl)thio)acetic acid (7.0 mg, 20 μmol, 1.0 eq.) was reacted with thalidomide-O-C2-amine (TFA salt, 9.1 mg, 21 μmol, 1.05 eq.). Purified by preparative HPLC (MeCN/H_2O gradient, 0.1% FA) to afford **14a** as a white solid (10.3 mg, 15.9 μmol, 79%). ^{1}H NMR (600 MHz, CD_3OD) δ 7.73 (dd, *J* = 8.5, 7.3 Hz, 1H), 7.62 – 7.56 (m, 2H), 7.56 – 7.49 (m, 2H), 7.47 – 7.37 (m, 3H), 6.34 (dd, *J* = 3.3, 1.8 Hz, 1H), 6.30 (dd, *J* = 3.3, 0.8 Hz, 1H), 5.28 (s, 2H), 5.07 (dd, *J* = 12.7, 5.5 Hz, 1H), 4.25 (t, *J* = 5.2 Hz, 2H), 3.94 (s, 2H), 3.76 – 3.59 (m, 2H), 2.85 (ddd, *J* = 17.3, 13.8, 5.4 Hz, 1H), 2.73 (ddd, *J* = 17.3, 4.5, 2.5 Hz, 1H), 2.67 (qd, *J* = 12.8, 4.5 Hz, 1H), 2.08 (dtd, *J* = 13.1, 5.3, 2.4 Hz, 1H). ^{13}C NMR (151 MHz, CD_3OD) δ 174.60, 171.37, 170.21, 168.40, 167.84, 157.52, 156.55, 153.09, 149.00, 144.84, 138.14, 138.03, 135.03, 131.56, 130.39, 126.19, 120.99, 118.35, 117.00, 111.72, 111.05, 69.32, 50.45, 42.96, 40.19, 37.86, 32.15, 23.63. HR-MS (ESI+) calc'd for $[M+H]^+$ $C_{30}H_{26}ClN_6O_7S$: 649.1267, found 649.1264 (0.5 ppm).

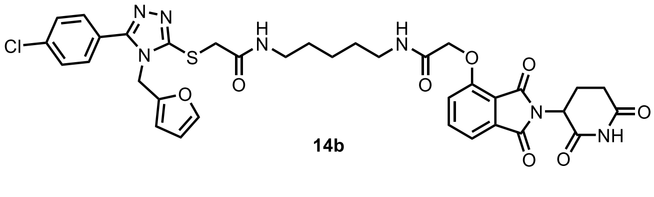

14b

2-((5-(4-chlorophenyl)-4-(furan-2-ylmethyl)-4H-1,2,4-triazol-3-yl)thio)-N-(5-(2-((2-(2,6-dioxopiperidin-3-yl)-1,3-dioxoisoindolin-4-yl)oxy)acetamido)pentyl)acetamide (14b)

General Procedure 1B. Reaction scale: Reaction scale: 2-((5-(4-chlorophenyl)-4-(furan-2-ylmethyl)-4H-1,2,4-triazol-3-yl)thio)acetic acid (7.0 mg, 20 μmol, 1.0 eq.) was reacted with thalidomide-O-acetamido-C5-amine (TFA salt, 11.1 mg, 21 μmol, 1.05 eq.). Purified by preparative HPLC (MeCN/H_2O gradient, 0.1% FA) to afford **14b** as a white solid (11.1 mg, 14.8 μmol, 74%). ^{1}H NMR (400 MHz, DMSO-d_6) δ 11.13 (s, 1H), 8.24 (t, *J* = 5.6 Hz, 1H), 7.97 (t, *J* = 5.7 Hz, 1H), 7.81 (dd, *J* = 8.5, 7.3 Hz, 1H), 7.70 (d, *J* = 8.5 Hz, 2H), 7.62 (d, *J* = 8.5 Hz, 3H), 7.49 (d, *J* = 7.2 Hz, 1H), 7.38 (d, *J* = 8.5 Hz, 1H), 6.41 (dd, *J* = 3.3, 1.9 Hz, 1H), 6.32 (d, *J* = 3.3 Hz, 1H), 5.26 (s, 2H), 5.12 (dd, *J* = 12.9, 5.4 Hz, 1H), 4.77 (s, 2H), 3.93 (s, 2H), 3.12 (q, *J* = 6.6 Hz, 2H), 3.05 (q, *J* = 6.6 Hz, 2H), 2.90 (ddd, *J* = 17.4, 14.0, 5.4 Hz, 1H), 2.69 – 2.49 (m, 2H), 2.10 – 1.98 (m, 1H), 1.48 – 1.33 (m, 4H), 1.30 – 1.20 (m, 2H). ^{13}C NMR (151 MHz, DMSO-d_6) δ 172.83, 169.93, 166.77, 166.67, 166.47, 165.53, 155.08, 154.19, 151.22, 147.83, 143.72, 136.96, 135.07, 133.04, 130.31, 129.12, 125.72, 120.38, 116.80, 116.05, 110.79, 109.59, 67.61, 48.81, 41.36, 38.92, 38.25, 36.88, 30.96, 28.66, 28.57, 23.60, 22.01. HR-MS (ESI+) calc'd for $[M+H]^+$ $C_{35}H_{35}ClN_7O_8S$: 748.1951, found 748.1947 (0.6 ppm).

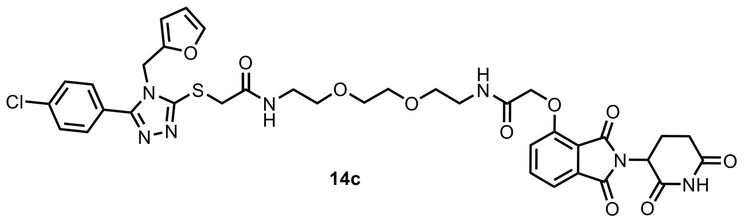

14c

2-((5-(4-chlorophenyl)-4-(furan-2-ylmethyl)-4H-1,2,4-triazol-3-yl)thio)-N-(2-(2-(2-(2-((2-(2,6-dioxopiperidin-3-yl)-1,3-dioxoisoindolin-4-yl)oxy)acetamido)ethoxy)ethoxy)ethyl)acetamide (14c)

General Procedure 1B. Reaction scale: 2-((5-(4-chlorophenyl)-4-(furan-2-ylmethyl)-4H-1,2,4-triazol-3-yl)thio)acetic acid (7.0 mg, 20 µmol, 1.0 eq.) was reacted with thalidomide-O-acetamido-PEG2-amine (HCl salt, 10.5 mg, 21 µmol, 1.05 eq.). Purified by preparative HPLC (MeCN/H_2O gradient, 0.1% FA) to afford **14c** as a white solid (8.2 mg, 10.3 µmol, 52%). ^{1}H NMR (400 MHz, CD_3OD) δ 7.79 (dd, *J* = 8.5, 7.3 Hz, 1H), 7.69 – 7.64 (m, 2H), 7.60 – 7.54 (m, 2H), 7.52 (d, *J* = 7.3 Hz, 1H), 7.46 (dd, *J* = 1.9, 0.8 Hz, 1H), 7.42 (d, *J* = 8.5 Hz, 1H), 6.36 (dd, *J* = 3.3, 1.9 Hz, 1H), 6.31 (dd, *J* = 3.3, 0.8 Hz, 1H), 5.28 (s, 2H), 5.12 (dd, *J* = 12.5, 5.5 Hz, 1H), 4.76 (s, 2H), 3.91 (s, 2H), 3.64 – 3.58 (m, 6H), 3.50 (m, 4H), 3.35 (t, *J* = 5.4 Hz, 2H), 2.88 (ddd, *J* = 17.8, 14.2, 5.2 Hz, 1H), 2.79 – 2.64 (m, 2H), 2.22 – 2.07 (m, 1H). ^{13}C NMR (151 MHz, DMSO-d_6) (*1 C expected under solvent*) δ 173.28, 170.37, 167.38, 167.27, 167.21, 165.92, 155.45, 154.64, 151.63, 148.28, 144.17, 137.41, 135.52, 133.50, 130.75, 129.57, 126.18, 120.78, 117.20, 116.51, 111.24, 110.02, 70.02 (2 Cs), 69.34, 69.29, 67.92, 49.26, 41.81, 38.86 & 38.75 (rot), 37.27, 31.41, 22.46. HR-MS (ESI+) calc'd for $[M+H]^+$ $C_{36}H_{37}ClN_7O_{10}S$: 794.2006, found 794.2027 (2.7 ppm).

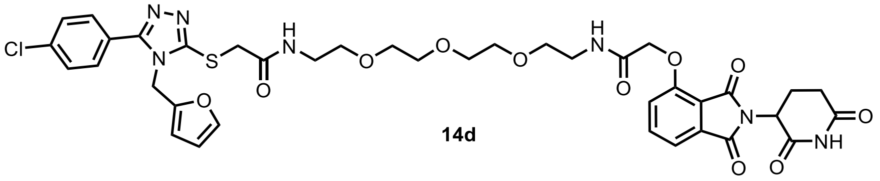

14d

2-((5-(4-chlorophenyl)-4-(furan-2-ylmethyl)-4H-1,2,4-triazol-3-yl)thio)-N-(1-((2-(2,6-dioxopiperidin-3-yl)-1,3-dioxoisoindolin-4-yl)oxy)-2-oxo-6,9,12-trioxa-3-azatetradecan-14-yl)acetamide (14d)

General Procedure 1B. Reaction scale: 2-((5-(4-chlorophenyl)-4-(furan-2-ylmethyl)-4H-1,2,4-triazol-3-yl)thio)acetic acid (7.0 mg, 20 µmol, 1.0 eq.) was reacted with thalidomide-O-acetamido-PEG3-amine (HCl salt, 11.4 mg, 21 µmol, 1.05 eq.). Purified by preparative HPLC (MeCN/H_2O gradient, 0.1% FA) to afford **14d** as a white solid (11.3 mg, 13.5 µmol, 67%). ^{1}H NMR (400 MHz, CD_3OD) δ 7.79 (dd, *J* = 8.5, 7.3 Hz, 1H), 7.72 – 7.61 (m, 2H), 7.61 – 7.55 (m, 2H), 7.52 (d, *J* = 7.3 Hz, 1H), 7.46 (dd, *J* = 1.9, 0.8 Hz, 1H), 7.42 (d, *J* = 8.4 Hz, 1H), 6.36 (dd, *J* = 3.3, 1.9 Hz, 1H), 6.31 (dd, *J* = 3.3, 0.8 Hz, 1H), 5.28 (s, 2H), 5.13 (dd, *J* = 12.6, 5.5 Hz, 1H), 4.76 (s, 2H), 3.93 (s, 2H), 3.62 (s, 4H), 3.61 – 3.53 (m, 6H), 3.54 – 3.46 (m, 4H), 3.36 (t, *J* = 5.4 Hz, 2H), 2.88 (ddd, *J* = 17.7, 14.3, 5.1 Hz, 1H), 2.81 – 2.65 (m, 2H), 2.14 (dtd, *J* = 12.9, 4.9, 2.1 Hz, 1H). ^{13}C NMR (151 MHz, CD_3OD) δ 174.61, 171.36, 169.96, 169.90, 168.30, 167.59, 156.63, 156.16, 153.27, 149.04, 144.86, 138.20, 138.08, 134.93, 131.73, 130.42, 126.33, 121.52, 119.17, 117.89, 111.74, 111.04, 71.58, 71.54, 71.36, 71.27, 70.32, 70.29, 69.18, 50.55, 49.43, 42.93, 40.87, 40.19, 38.05, 32.18, 23.66. HR-MS (ESI+) calc'd for $[M+H]^+$ $C_{38}H_{41}ClN_7O_{11}S$: 838.2268, found 838.2261 (0.9 ppm).

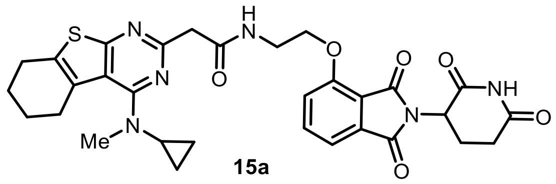

15a

2-(4-(cyclopropyl(methyl)amino)-5,6,7,8-tetrahydrobenzo[4,5]thieno[2,3-d]pyrimidin-2-yl)-N-(2-((2-(2,6-dioxopiperidin-3-yl)-1,3-dioxoisoindolin-4-yl)oxy)ethyl)acetamide (15a)

General Procedure 1A. Reaction scale: thalidomide-O-C2-amine 15 mg (47.2 μmol, 1.0 eq) and 2-(4-(cyclopropyl(methyl)amino)-5,6,7,8-tetrahydrobenzo[4,5]thieno[2,3-d]pyrimidin-2-yl)acetic acid 15 mg (47.2 μmol,1.0 eq). Purified by pTLC (6% MeOH/DCM) to afford **15a** as a yellow sticky solid (8 mg, 28%). ^{1}H NMR (600 MHz, CDCl3) δ 8.49 (s, 2H), 8.15 (s, 1H), 7.67 – 7.64 (m, 1H), 7.43 (d, J = 7.3 Hz, 1H), 7.30 (d, J = 8.5 Hz, 1H), 4.98 – 4.92 (m, 1H), 4.32 (t, J = 5.5 Hz, 2H), 3.97 (s, 2H), 3.80 – 3.72 (m, J = 5.5 Hz, 2H), 3.70 – 3.62 (m, 1H), 3.21 (s, 2H), 3.16 – 3.03 (m, 2H), 2.86 – 2.78 (m, 4H), 2.19 – 2.08 (m, 1H), 1.94 – 1.87 (m, 2H), 1.80 – 1.73 (m, 2H), 1.56 – 1.50 (m, 2H), 1.44 – 1.42 (m, 1H), 1.27 – 1.23 (m, 2H), 0.90 – 0.87 (m, 2H). ^{13}C NMR (151 MHz, $CDCl_3$) δ 171.13, 170.35, 170.00, 168.68, 167.11, 166.80, 166.01, 157.47, 154.75, 154.51, 136.99, 133.68, 119.51, 118.08, 117.35, 86.45, 80.31, 70.43, 70.26, 69.89, 69.56, 68.08, 62.26, 55.93, 50.03, 41.32, 39.40, 39.02, 31.95, 31.89, 29.72, 28.37, 27.30, 22.72, 21.94, 14.63, 14.15. HR-MS (ESI+) calc'd for $C_{31}H_{32}N_6O_6S$: 616.2103, found $[M+H]^+$ 617.2184.

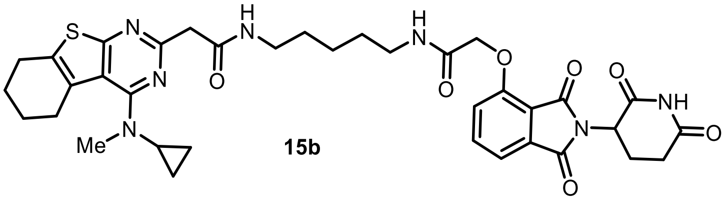

15b

2-(4-(cyclopropyl(methyl)amino)-5,6,7,8-tetrahydrobenzo[4,5]thieno[2,3-d]pyrimidin-2-yl)-N-(5-(2-((2-(2,6-dioxopiperidin-3-yl)-1,3-dioxoisoindolin-4-yl)oxy)acetamido) pentyl) acetamide (15b)

General Procedure 1A. Reaction scale: thalidomide-O-acetamido-C5-amine 19 mg (37.8 μmol, 1.0 eq) and 2-(4-(cyclopropyl(methyl)amino)-5,6,7,8-tetrahydrobenzo[4,5]thieno[2,3-d]pyrimidin-2-yl)acetic acid 12 mg (37.8 μmol,1.0 eq). Purified by pTLC (7% MeOH/DCM) **15b** as a yellow sticky solid (6.8 mg, 23 %). ^{1}H NMR (600 MHz, $CDCl_3$) δ 9.27 (s, 1H), 8.32 (s, 1H), 7.80 (s, 1H), 7.76 – 7.69 (m, 1H), 7.61 – 7.58 (m, 1H), 7.53 (d, *J* = 7.4 Hz, 1H), 7.22 – 7.18 (m, 1H), 5.02 – 4.98 (m, 1H), 4.70 – 4.59 (m, 2H), 3.91 (s, 2H), 3.72 – 3.67 (m, 2H), 3.48 – 3.44 (m, 1H), 3.32 – 3.28 (m, 3H), 3.24 – 3.19 (m, 2H), 3.17 – 3.14 (m, 2H), 3.04 – 2.98 (m, 1H), 2.92 – 2.74 (m, 4H), 2.19 – 2.17 (m, 2H), 1.97 – 1.88 (m, 2H), 1.78 – 1.74 (m, 2H), 1.61 – 1.57 (m, 4H), 1.49 – 1.46 (m, 3H), 1.43 – 1.39 (m, 3H), 0.92 (s, 1H). ^{13}C NMR (151 MHz, DMSO) δ 172.82, 169.92, 167.49, 167.43, 166.77, 166.66, 165.53, 160.25, 156.29, 155.09, 136.96, 133.04, 132.84, 128.43, 120.38, 118.01, 117.96, 116.79, 116.05, 67.61, 53.50, 48.81, 44.67, 41.76, 40.73, 38.65, 38.26, 35.17, 30.96, 28.73, 26.78, 25.20, 23.66, 22.55, 22.28, 22.02, 18.06, 16.74, 12.43, 8.62. HR-MS (ESI+) calc'd for $C_{36}H_{41}N_7O_7S$: 715.8260, found $[M+H]^+$ 716.2861.

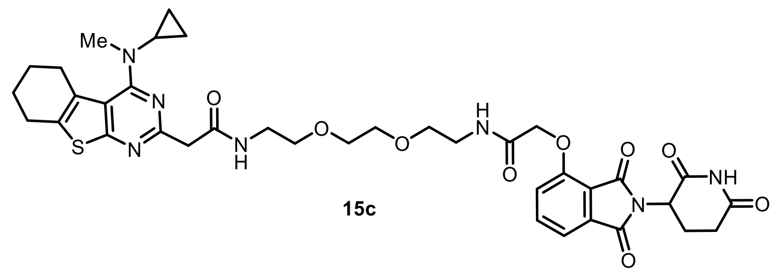

15c

2-(4-(cyclopropyl(methyl)amino)-5,6,7,8-tetrahydrobenzo[4,5]thieno[2,3-d]pyrimidin-2-yl)-N-(2-(2-(2-(2-((2-(2,6-dioxopiperidin-3-yl)-1,3-dioxoisoindolin-4-yl)oxy)acetamido)ethoxy)ethoxy)ethyl)acetamide (15c)

General Procedure 1A. Reaction scale: Thalidomide-O-acetamido-PEG2-C2-amine 19 mg (37.8 µmol, 1.0 eq) and 2-(4-(cyclopropyl(methyl)amino)-5,6,7,8-tetrahydrobenzo[4,5]thieno[2,3-d]pyrimidin-2-yl)acetic acid 12 mg (37.8 µmol,1.0 eq). Purified by pTLC (7% MeOH/DCM) **15c** as a yellow sticky solid (4 mg, 22 %). ^{1}H NMR (600 MHz, CDCl3) δ 9.13 (s, 1H), 8.32 (s, 1H), 7.72 (d, *J* = 7.3 Hz, 1H), 7.64 –7.61 (m, 1H), 7.53 (d, *J* = 7.3 Hz, 1H), 7.18 (d, *J* = 8.4 Hz, 1H), 5.01 – 4.90 (m, 1H), 4.64 (s, 2H), 3.86 (s, 2H), 3.64 – 3.53 (m, 9H), 3.47 (d, *J* = 5.7 Hz, 2H), 3.08 (s, 3H), 2.87 – 2.84 (m 6H), 1.94 – 1.90 (m, 2H), 1.76 –1.72 (m, 2H), 0.82 – 0.80 (m, 1H). ^{13}C NMR (151 MHz, $CDCl_3$) δ 171.39, 169.23, 168.38, 167.01, 166.81, 165.95, 154.52, 137.07, 133.83, 127.84, 119.37, 119.27, 118.16, 117.39, 70.51, 70.18, 70.06, 69.66, 67.98, 53.57, 49.44, 42.09, 39.46, 39.20, 34.80, 31.58, 27.14, 25.92, 23.10, 22.96, 22.81, 8.52. HR-MS (ESI+) calc'd for $C_{37}H_{43}N_7O_9S$: 761.2843, found $[M+H]^+$ 762.2902.

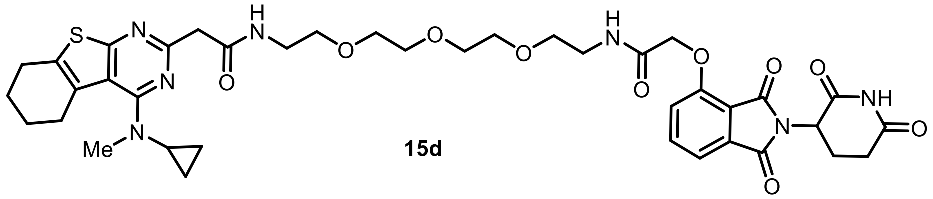

15d

2-(4-(cyclopropyl(methyl)amino)-5,6,7,8-tetrahydrobenzo[4,5]thieno[2,3-d]pyrimidin-2-yl)-N-(1-((2-(2,6-dioxopiperidin-3-yl)-1,3-dioxoisoindolin-4-yl)oxy)-2-oxo-6,9,12-trioxa-3-azatetradecan-14-yl)acetamide (15d)

General Procedure 1A. Reaction scale: Thalidomide-O-acetamido-PEG3-C2-amine 20 mg (37.8 µmol, 1.0 eq) and 2-(4-(cyclopropyl(methyl)amino)-5,6,7,8-tetrahydrobenzo[4,5]thieno[2,3-d]pyrimidin-2-yl)acetic acid 12 mg (37.8 µmol,1.0 eq). Purified by pTLC (7% MeOH/DCM) **15d** as a yellow sticky solid (10 mg, 34 %). ^{1}H NMR (600 MHz, $CDCl_3$) δ 9.01 (s, 1H), 8.20 (t, *J* = 5.8 Hz, 1H), 7.73 (d, *J* = 7.3 Hz, 1H), 7.66 (t, *J* = 5.6 Hz, 1H), 7.54 (d, *J* = 7.3 Hz, 1H), 7.19 (d, *J* = 8.4 Hz, 1H), 4.98 – 4.94 (m, 1H), 4.65 (s, 2H), 3.86 (s, 2H), 3.67 – 3.56 (m, 10 H), 3.48 (d, *J* = 5.4 Hz, 2H), 3.11 (s, 3H), 2.90 – 2.81 (m, 4H), 2.81 – 2.71 (m, 2H), 1.95 – 1.87 (m, 2H), 1.78 – 1.74 (m, 2H), 0.82 (d, *J* = 6.6 Hz, 1H). ^{13}C NMR (151 MHz, DMSO) δ 172.81, 169.90, 168.14, 166.92, 166.75, 165.46, 160.73, 157.46, 155.00, 136.96, 133.05, 132.43, 128.05, 120.33, 118.31, 116.75, 116.05, 69.76, 69.63, 69.13, 68.84, 67.49, 48.81, 45.44, 40.90, 40.43, 38.78, 38.42, 34.86, 30.97, 26.56, 25.23, 22.58, 22.34, 22.02, 8.28. HR-MS (ESI+) calc'd for $C_{39}H_{47}N_7O_{10}S$: 805.3105, found $[M+H]^+$ 806.3179.

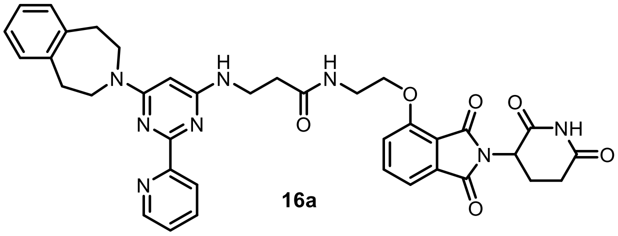

16a

N-(2-((2-(2,6-dioxopiperidin-3-yl)-1,3-dioxoisoindolin-4-yl)oxy)ethyl)-3-((2-(pyridin-2-yl)-6-(1,2,4,5-tetrahydro-3H-benzo[d]azepin-3-yl)pyrimidin-4-yl)amino)propenamide (16a)

General Procedure 1A. Reaction scale: Thalidomide-O-C2-amine 9 mg (25.6 µmol, 1.0 eq) and 3-((2-(pyridin-2-yl)-6-(1,2,4,5-tetrahydro-3H-benzo[d]azepin-3-yl)pyrimidin-4-yl)amino)propanoic acid 10 mg (25.6 µmol,1.0 eq). Purified by pTLC (7% MeOH/DCM) to afford **16a** as a yellow sticky solid (4 mg, 22%). ^{1}H NMR (600 MHz, $CDCl_3$) δ 8.7 (d, *J* = 7.2 Hz, 1H), 8.33 (d, *J* = 7.8 Hz, 1H), 7.86 – 7.78 (m, 1H), 7.61 – 7.57 (m, 2H), 7.42 – 7.39 (m, 2H), 7.21 – 7.11 (m, 5H), 5.62 (s, 1H), 5.00 – 4.94 (m, 1H), 4.28 – 4.24 (m, 1H), 4.15 – 4.10 (m, 1H), 3.97– 3.91 (m, 4H), 3.77 – 3.74 (m, 1H), 3.68 – 3.63 (m, 2H), 3.57 (s, 1H), 3.06 – 2.99 (m, 4H), 2.88 – 2.81 (m, 1H), 2.78 – 2.75 (m, 2H), 2.65 – 2.61 (m, 2H). ^{13}C NMR (151 MHz, DMSO) δ 172.82, 171.01, 169.96, 166.78, 165.27, 160.96, 155.68, 149.04, 140.38, 137.03, 133.25, 129.88, 126.33, 123.16, 120.06, 116.49, 115.57, 67.46, 54.96, 48.76, 48.62, 47.13, 40.43, 38.04, 35.74, 30.97, 22.04. HR-MS (ESI+) calc'd for $C_{37}H_{36}N_8O_6$: 688.7450, found $[M+H]^+$ 689.2818.

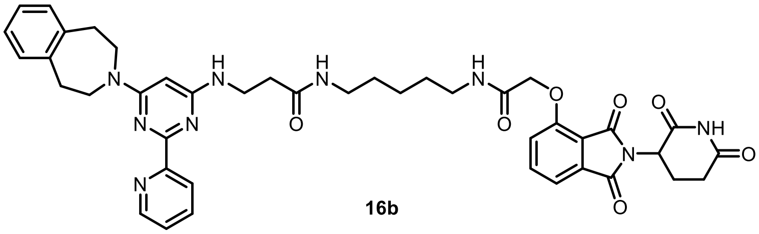

16b

N-(5-(2-((2-(2,6-dioxopiperidin-3-yl)-1,3-dioxoisoindolin-4-yl)oxy)acetamido)pentyl)-3-((2-(pyridin-2-yl)-6-(1,2,4,5-tetrahydro-3H-benzo[d]azepin-3-yl)pyrimidin-4-yl)amino) propenamide (16b)

General Procedure 2. Reaction scale: Thalidomide-O-acetamido-C5-amine 13 mg (25.6 µmol, 1.0 eq) and 3-((2-(pyridin-2-yl)-6-(1,2,4,5-tetrahydro-3H-benzo[d]azepin-3-yl)pyrimidin-4-yl)amino)propanoic acid 10 mg (25.6 µmol,1.0 eq). Purified by pTLC (7% MeOH/DCM) **16b** as a yellow sticky solid (4 mg, 22 %). ^{1}H NMR (400 MHz, $CDCl_3$) δ 8.76 – 8.74 (m, 1H), 8.36 (d, *J* = 7.8 Hz, 1H), 7.85 (d, *J* = 7.0 Hz, 1H), 7.77 – 7.67 (m, 1H), 7.58 (s, 1H), 7.54 – 7.48 (m, 1H), 7.47 – 7.44 (m, 1H), 7.19 – 7.11 (m, 5H), 5.65 (s, 1H), 5.05 – 5.01 (m, 1H), 4.63 – 4.61 (m, 2H), 3.96 – 3.92 (m, 4H), 3.70 – 3.55 (m, 2H), 3.26 – 3.24 (m, 3H), 3.07 – 2.95 (m, 4H), 2.91 – 2.73 (m, 3H), 2.72 – 2.53 (m, 3H), 2.15 – 2.11 (m, 1H), 1.56 – 1.52 (m, 2H), 1.44 – 1.39 (m, 2H), ^{13}C NMR (151 MHz, DMSO) δ 172.84, 170.50, 169.93, 166.77, 166.66, 165.54, 155.06, 148.99, 140.87, 136.96, 133.04, 129.81, 126.22, 123.18, 120.39, 116.82, 116.08, 67.63, 48.82, 46.69, 38.48, 38.29, 35.97, 30.98, 28.81, 28.73, 23.75, 22.03. HR-MS (ESI+) calc'd for $C_{42}H_{45}N_9O_7$: 787.8780, found $[M+H]^+$ 788.3516.

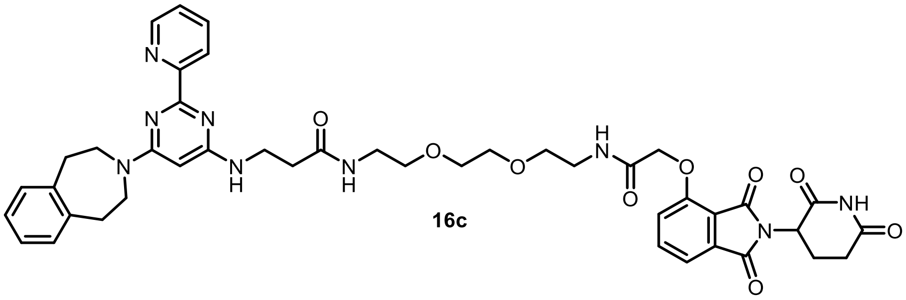

16c

N-(2-(2-(2-(2-((2-(2,6-dioxopiperidin-3-yl)-1,3-dioxoisoindolin-4-yl)oxy)acetamido) ethoxy) ethoxy)ethyl)-3-((2-(pyridin-2-yl)-6-(1,2,4,5-tetrahydro-3H-benzo[d]azepin-3-yl)pyrimidin-4-yl)amino)propenamide (16c)

General Procedure 2. Reaction scale: Thalidomide-O-acetamido-PEG2-C2-amine 17 mg (33.9 µmol, 1.0 eq), and 3-((2-(pyridin-2-yl)-6-(1,2,4,5-tetrahydro-3H-benzo[d]azepin-3-yl)pyrimidin-4-yl)amino)propanoic acid 12 mg (30.8 µmol,1.0 eq). Purified by pTLC (7% MeOH/DCM) **16c** as a yellow sticky solid (4 mg, 23%). ^{1}H NMR (600 MHz, CD_3OD_SPE) δ 8.74 – 8.67 (m, 1H), 8.47 – 8..45 (m, 2H), 8.27 (s, 1H), 8.02 (dd, *J* = 7.8, 1.7 Hz, 1H), 7.72 (d, *J* = 7.3 Hz, 1H), 7.63 – 7.57 (m, 1H), 7.45 (d, *J* = 7.3 Hz, 1H), 7.33 (d, *J* = 8.4 Hz, 1H), 7.19 – 7.03 (m, 3H), 5.12 – 5.09 (m, 1H), 4.67 (s, 2H), 4.58 (s, 4H), 3.97 (s, 4H), 3.67 (t, *J* = 6.4 Hz, 2H), 3.61 – 3.54 (m, 4H), 3.50 – 3.46 (m, 2H), 3.34 (t, *J* = 5.4 Hz, 1H), 3.06 – 3.00 (m, 2H), 2.91 – 2.80 (m, 1H), 2.78 – 2.63 (m, 2H), 2.56 (t, *J* = 6.4 Hz, 1H), 2.13 – 2.09 (m, 2H). ^{13}C NMR (151 MHz, DMSO) δ 172.84, 170.64, 169.92, 166.94, 166.76, 165.47, 161.19, 154.98, 149.04, 140.55, 136.97, 133.04, 129.86, 126.29, 123.18, 120.33, 116.76, 116.08, 69.57, 69.13, 68.83, 67.48, 48.82, 46.92, 38.60, 38.40, 35.82, 30.97, 22.02. HR-MS (ESI+) calc'd for $C_{43}H_{47}N_9O_9$: 833.9030, found $[M+H]^+$ 834.3573.

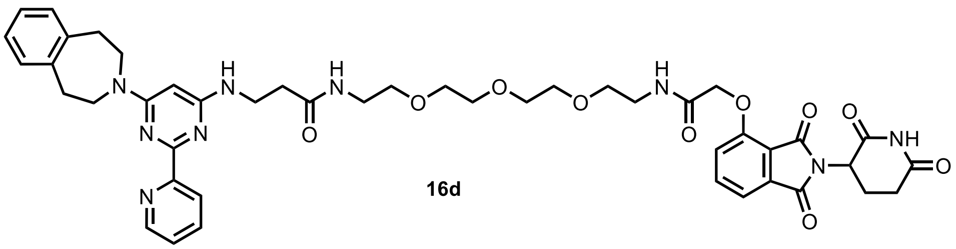

16d

N-(1-((2-(2,6-dioxopiperidin-3-yl)-1,3-dioxoisoindolin-4-yl)oxy)-2-oxo-6,9,12-trioxa-3-azatetradecan-14-yl)-3-((2-(pyridin-2-yl)-6-(1,2,4,5-tetrahydro-3H-benzo[d]azepin-3-yl)pyrimidin-4-yl)amino)propenamide (16d)

General Procedure 2. Reaction scale: thalidomide-O-acetamido-PEG3-C2-amine 18.4 mg (33.9 µmol, 1.0 eq), and 3-((2-(pyridin-2-yl)-6-(1,2,4,5-tetrahydro-3H-benzo[d]azepin-3-yl)pyrimidin-4-yl)amino)propanoic acid 12 mg (30.8 µmol,1.0 eq). Purified by pTLC (7% MeOH/DCM) to get **16d** as a yellow sticky solid (3.5 mg, 21%). ^{1}H NMR (600 MHz, $CDCl_3$) δ 8.68 (d, *J* = 7.2 Hz, 1H), 8.45 – 8.42 (m, 2H), 8.05 – 7.93 (m, 1H), 7.75 – 7.73 (m, 1H), 7.56 – 7.53 (m, 1H), 7.51 – 7.47 (m, 1H), 7.35 – 7.29 (m, 1H), 7.22 – 6.98 (m, 3H), 5.81 (d, *J* = 6.3 Hz, 1H), 5.54 – 5.39 (m, 1H), 5.17 – 5.02 (m, 2H), 4.68 (t, *J* = 7.7 Hz, 2H), 3.95 (s, 2H), 3.68 (t, *J* = 7.2 Hz, 2H), 3.64 – 3.51 (m, 4H), 3.49 – 3.45 (m, 2H), 3.39 – 3.26 (m, 8H), 3.04 – 3.01 (m, 2H), 2.92 – 2.61 (m, 4H), 2.58 – 2.54 (m, 2H), 2.15 – 2.09 (m, 2H). ^{13}C NMR (151 MHz, DMSO) δ 173.28, 171.30, 170.38, 167.39, 167.22, 165.93, 161.99, 155.45, 149.47, 141.36, 137.43, 137.09, 133.51, 130.26, 126.66, 124.76, 123.63, 120.80, 117.22, 116.53, 69.58, 69.28, 67.95, 49.28, 47.11, 40.89, 39.03, 38.87, 37.81, 36.44, 31.43, 22.48. HR-MS (ESI+) calc'd for $C_{45}H_{51}N_9O_{10}$: 877.3759, found $[M+H]^+$ 878.3839.

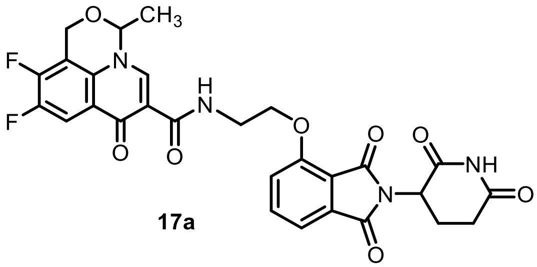

17a

N-(2-((2-(2,6-dioxopiperidin-3-yl)-1,3-dioxoisoindolin-4-yl)oxy)ethyl)-9,10-difluoro-3-methyl-7-oxo-1H,3H,7H-[1,3]oxazino[5,4,3-ij]quinoline-6-carboxamide (17a)

General Procedure 2. Reaction scale: Thalidomide-O-C2-amine 22 mg (71.1 μmol, 1.0 eq) and 9,10-difluoro-3-methyl-7-oxo-1H,3H,7H-[1,3]oxazino[5,4,3-ij]quinoline-6-carboxylic acid 20 mg (71.1 μmol,1.0 eq). Purified by pTLC (7% MeOH/DCM) to afford **17a** as an off-white solid (15.0 mg, 54%). ^{1}H NMR (600 MHz, DMSO) δ 11.13 (s, 1H), 10.04 (s, 1H), 8.90 (s, 1H), 7.81 (d, J = 7.9 Hz, 1H), 7.72 (d, J = 7.6 Hz, 1H), 7.64 – 7.61 (m, 2H), 7.46 (d, J = 7.0 Hz, 1H), 5.13 – 5.07 (m, 1H), 4.97 – 4.90 (m, 1H), 4.66 – 4.63 (m, 1H), 4.48 – 4.44 (m, 1H), 4.38 (t, J = 5.8 Hz, 2H), 3.83 – 3.76 (m, 2H), 2.90 – 2.88 (m, 2H), 2.63 – 2.47 (m, 4H), 2.08 – 2.02 (m, 1H), 1.46 – 1.42 (m, 3H).^{13}C NMR (151 MHz, DMSO) δ 174.37, 173.29, 170.44, 167.27, 165.66, 164.69, 156.17, 149.62, 149.55, 147.99, 147.92, 146.20, 142.30, 142.18, 140.63, 140.52, 137.51, 135.84, 135.76, 133.75, 125.21, 123.27, 123.23, 120.57, 116.95, 116.04, 111.21, 104.08, 103.95, 69.34, 68.29, 54.63, 49.21, 31.42, 22.48, 18.21. HR-MS (ESI+) calc'd for $C_{28}H_{22}F_2N_4O_8$: 580.1406, found [M+H]$^+$ 581.1485.

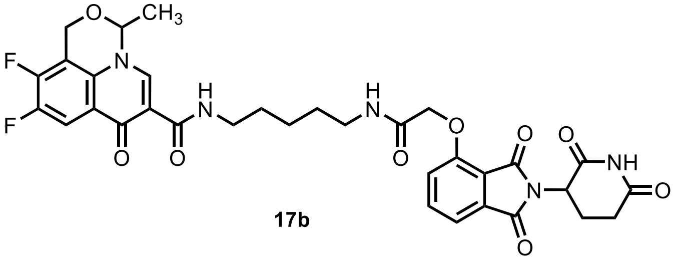

17b

N-(5-(2-((2-(2,6-dioxopiperidin-3-yl)-1,3-dioxoisoindolin-4-yl)oxy)acetamido)pentyl)-9,10-difluoro-3-methyl-7-oxo-1H,3H,7H-[1,3]oxazino[5,4,3-ij]quinoline-6-carboxamide (17b)

General Procedure 1A. Reaction scale: Thalidomide-O-acetamido-C5-amine 28 mg (53.3 μmol, 1.0 eq) and 9,10-difluoro-3-methyl-7-oxo-1H,3H,7H-[1,3]oxazino[5,4,3-ij]quinoline-6-carboxylic acid 15 mg (53.3 μmol,1.0 eq). Purified by pTLC (7% MeOH/DCM) to afford **17b** as an off-white solid (15.0 mg, 45%). ^{1}H NMR (600 MHz, CDCl3) δ 10.10 (s, 1H), 9.89 (s, 1H), 8.80 (s, 1H), 7.85 – 7.82 (m, 1H), 7.73 (s, 1H), 7.71 – 7.63 (m, 1H), 7.53 (dd, J = 7.3, 2.2 Hz, 1H), 7.22 – 7.18 (m, 1H), 5.09 – 4.93 (m, 1H), 4.74 – 4.53 (m, 3H), 4.50 – 4.48 (m, 1H), 4.42 – 4.38 (m, 1H), 3.56 – 3.54 (m, 2H), 3.42 – 3.38 (m, 1H), 3.26 – 3.24 (m, 1H), 2.92 – 2.71 (m, 3H), 2.17 – 2.14 (m, 1H), 1.71 – 1.55 (m, 6H), 1.54 – 1.38 (m, 4H). ^{13}C NMR (151 MHz, DMSO) δ 174.48, 173.29, 170.37, 167.20, 167.14, 165.99, 164.05, 155.45, 149.58, 149.50, 147.94, 147.87, 145.97, 142.24, 142.13, 140.58, 140.47, 137.39, 135.78, 135.70, 133.44, 125.20, 125.18, 123.21, 123.17, 120.84, 117.27, 116.48, 111.53, 104.03, 103.90, 69.34, 68.11, 54.60, 54.03, 49.27, 42.29, 38.75, 38.65, 31.42, 29.31, 29.06, 24.21, 22.45, 18.53, 18.19, 17.18, 12.95. HR-MS (ESI+) calc'd for $C_{33}H_{31}F_2N_5O_9$: 679.2090, found [M+H]$^+$ 680.2144.

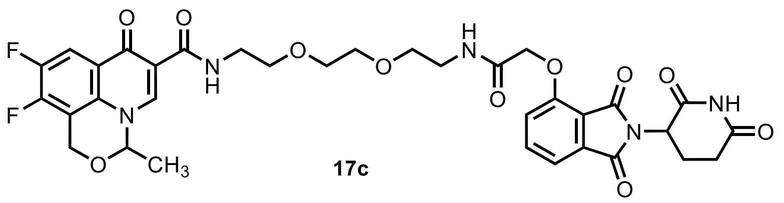

17c

N-(2-(2-(2-(2-((2-(2,6-dioxopiperidin-3-yl)-1,3-dioxoisoindolin-4-yl)oxy)acetamido)ethoxy)ethoxy) ethyl)-9,10-difluoro-3-methyl-7-oxo-1H,3H,7H-[1,3]oxazino[5,4,3-ij]quinoline-6-carboxamide (17c)

General Procedure 1A. Reaction scale: Thalidomide-O-acetamido-PEG2-C2-amine 18 mg (36.0 µmol, 1.0 eq) and 9,10-difluoro-3-methyl-7-oxo-1H,3H,7H-[1,3]oxazino[5,4,3-ij]quinoline-6-carboxylic acid 10.6 mg (37.9 µmol,1.0 eq). Purified by pTLC (7% MeOH/DCM) to afford **17c** as an off-white solid (7.0 mg, 38%). ^{1}H NMR (600 MHz, $CDCl_3$) ^{1}H NMR (600 MHz, $CDCl_3$) δ 9.97 (s, 1H), 8.73 (s, 1H), 7.77 – 7.74 (m, 1H), 7.71 – 7.55 (m, 2H), 7.46 – 7.44 (m, 1H), 7.13 – 7.11 (m, 1H), 4.94 – 4.92 (m, 1H), 4.65 – 4.51 (m, 2H), 4.50 – 4.46 (m, 1H), 4.42 – 4.38 (m, 1H), 4.32 – 4.28 (m, 1H), 3.68 – 3.47 (m, 10H), 2.87 – 2.68 (m, 2H), 2.07 – 2.05 (m, 1H), 1.52 – 1.48 (m, 4H), 1.48 – 1.36 (m, 1H). ^{13}C NMR (151 MHz, DMSO) δ 174.41, 173.27, 170.35, 167.33, 167.20, 165.88, 164.21, 155.38, 149.59, 149.51, 147.95, 147.88, 146.16, 146.06, 142.26, 142.14, 140.60, 140.48, 137.37, 135.79, 135.72, 133.45, 125.19, 123.27, 123.22, 120.73, 117.15, 116.44, 111.42, 104.07, 103.94, 70.17, 70.12, 69.74, 69.36, 67.91, 54.59, 49.26, 38.99, 38.93, 31.41, 22.45, 18.20, HR-MS (ESI+) calc'd for $C_{34}H_{33}F_2N_5O_{11}$: 725.2145, found $[M+H]^+$ 726.2235.

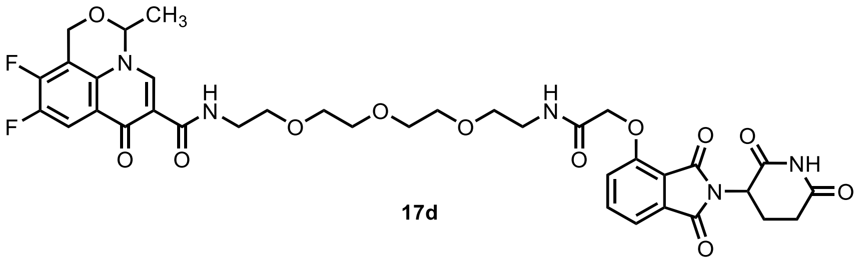

17d

N-(1-((2-(2,6-dioxopiperidin-3-yl)-1,3-dioxoisoindolin-4-yl)oxy)-2-oxo-6,9,12-trioxa-3-azatetradecan-14-yl)-9,10-difluoro-3-methyl-7-oxo-1H,3H,7H-[1,3]oxazino[5,4,3-ij]quinoline-6-carboxamide (17d)

General Procedure 1A. Reaction scale: Thalidomide-O-acetamido-PEG3-C2-amine 20 mg (36.9 µmol, 1.0 eq) and 9,10-difluoro-3-methyl-7-oxo-1H,3H,7H-[1,3]oxazino[5,4,3-ij]quinoline-6-carboxylic acid 11 mg (38.7 µmol,1.0 eq). Purified by pTLC (7% MeOH/DCM) to afford **17d** as an off-white solid (12.0 mg, 45%). ^{1}H NMR (600 MHz, CD_3OD) δ 10.24 (s, 1H), 8.77 (s, 1H), 8.54 (s, 1H), 7.83 – 7.64 (m, 2H), 7.52 – 7.44 (m, 1H), 7.44 – 7.33 (m, 1H), 5.19 – 5.05 (m, 2H), 4.75 – 4.73 (m, 4H), 4.58 – 4.54 (m, 2H), 4.45 (m, 1H), 3.71 – 3.54 (m, 10H), 3.49 – 3.38 (m, 4H), 2.89 – 2.86 (m, 1H), 2.79 – 2.61 (m, 2H), 2.18 – 2.07 (m, 1H), 1.54 (d, J = 6.9 Hz, 3H). ^{13}C NMR (151 MHz, DMSO) δ 174.40, 173.28, 170.36, 167.35, 167.20, 165.91, 164.23, 155.40, 149.59, 149.52, 147.96, 147.89, 146.06, 142.26, 142.15, 140.60, 140.49, 137.40, 135.80, 135.73, 133.46, 125.19, 120.74, 117.17, 116.48, 111.41, 104.05, 103.92, 70.25, 70.17, 70.10, 69.72, 69.33, 69.26, 67.90, 54.60, 49.26, 38.99, 38.88, 31.40, 22.46, 18.19. HR-MS (ESI+) calc'd for $C_{36}H_{37}F_2N_5O_{12}$: 769.2407, found $[M+H]^+$ 770.2470.

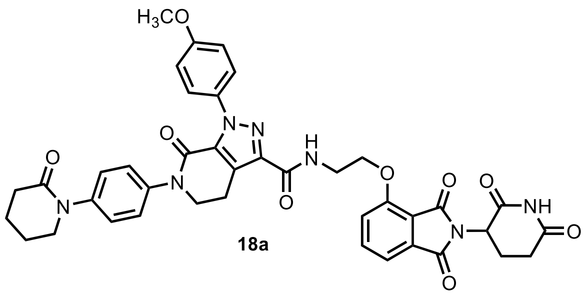

18a

N-(2-((2-(2,6-dioxopiperidin-3-yl)-1,3-dioxoisoindolin-4-yl)oxy)ethyl)-1-(4-methoxyphenyl)-7-oxo-6-(4-(2-oxopiperidin-1-yl)phenyl)-4,5,6,7-tetrahydro-1H-pyrazolo[3,4-c]pyridine-3-carboxamide (18a)

General Procedure 1A. Reaction scale: Thalidomide-O-C2-amine 10.3 mg (32.6 μmol, 1.0 eq) and 1-(4-methoxyphenyl)-7-oxo-6-(4-(2-oxopiperidin-1-yl)phenyl)-4,5,6,7-tetrahydro-1H-pyrazolo[3,4-c]pyridine-3-carboxylic acid 15 mg (32.6 μmol,1.0 eq). Purified by pTLC (7% MeOH/DCM) to afford **18a** as an off-white solid (7.0 mg, 38%). ^{1}H NMR (600 MHz, $CDCl_3$) δ 8.28 (s, 1H), 7.73 – 7.61 (m, 2H), 7.49 – 7.46 (m, 3H), 7.35 – 7.30 (m, 2H), 7.28 (d, *J* = 8.5 Hz, 1H), 7.25 – 7.21 (m, 2H), 6.95 – 6.83 (m, 2H), 4.90 – 4.87 (m, 1H), 4.37 (t, *J* = 5.4 Hz, 2H), 4.14 – 4.02 (m, 2H), 3.93 –3.89 (m, 2H), 3.80 (s, 3H), 3.67 – 3.55 (m, 2H), 3.35 (t, *J* = 6.7 Hz, 2H), 3.12 – 3.08 (m, 2H), 2.85 – 2.79 (m, 1H), 2.78 – 2.62 (m, 2H), 2.54 (t, *J* = 6.1 Hz, 2H), 2.07 – 1.99 (m, 1H), 1.94 – 1.92 (m, 3H), 1.83 (s, 2H), 1.43 – 1.31 (m, 4H). HR-MS (ESI+) calc'd for $C_{40}H_{37}N_7O_9$; 759.7760, ^{13}C NMR (151 MHz, DMSO) δ 173.29, 170.42, 169.35, 167.28, 165.73, 162.01, 159.64, 157.04, 156.09, 141.85, 141.52, 140.23, 137.52, 133.74, 133.50, 132.93, 127.34, 126.81, 126.47, 125.58, 120.49, 116.97, 116.01, 113.86, 67.42, 55.94, 54.02, 51.40, 51.31, 49.21, 42.28, 37.99, 33.05, 31.41, 23.46, 22.47, 21.42, 21.37, 18.53, 17.18, 12.94. HR-MS (ESI+) calc'd for $C_{40}H_{37}N_7F_2N_5O_{12}$: 759.2653, found $[M+H]^+$ 760.2727.

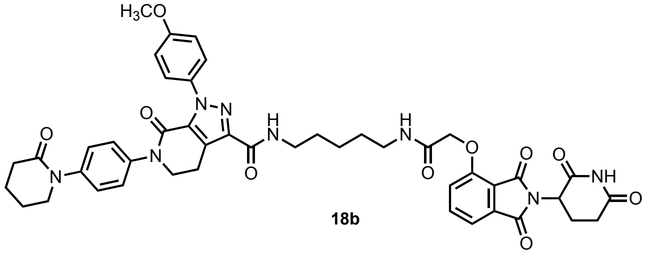

18b

N-(5-(2-((2-(2,6-dioxopiperidin-3-yl)-1,3-dioxoisoindolin-4-yl)oxy)acetamido)pentyl)-1-(4-methoxyphenyl)-7-oxo-6-(4-(2-oxopiperidin-1-yl)phenyl)-4,5,6,7-tetrahydro-1H-pyrazolo[3,4-c]pyridine-3-carboxamide (18b)

General Procedure 1A. Reaction scale: Thalidomide-O-acetamido-C5-amine 17 mg (32.5 μmol, 1.0 eq) and 1-(4-methoxyphenyl)-7-oxo-6-(4-(2-oxopiperidin-1-yl)phenyl)-4,5,6,7-tetrahydro-1H-pyrazolo[3,4-c]pyridine-3-carboxylic acid 15 mg (32.5 μmol,1.0 eq). Purified by pTLC (7% MeOH/DCM) to afford **18b** as an off-white solid (14 mg, 42%). 1H NMR (600 MHz, CDCl3) δ 9.29 (s, 1H), 7.69 – 7.64 (m, 1H), 7.52 – 7.48 (m, 1H), 7.48 (d, J = 7.3 Hz, 1H), 7.40 – 7.35 (m, 2H), 7.29 – 7.25 (m, 2H), 7.20 (s, 1H), 7.20 – 7.15 (m, 2H), 7.12 (d, J = 8.4 Hz, 1H), 7.03 – 7.00 (m, 1H), 6.88 – 6.82 (m, 1H), 4.98 – 4.88 (m, 1H), 4.59 – 4.57 (m, 2H), 4.09 – 4.00 (m, 2H), 3.74 (s, 2H), 3.60 – 3.48 (m, 2H), 3.44 – 3.42 (m, 2H), 3.39 – 3.18 (m, 3H), 3.10 – 3.03 (m, 1H), 2.84 – 2.65 (m, 2H), 2.48 (t, J = 6.1 Hz, 2H), 2.12 – 2.00 (m, 1H), 1.98 (s, 1H), 1.92 – 1.73 (m, 4H), 1.65 – 1.53 (m, 2H), 1.48 – 1.42 (m, 2H), 1.39 – 1.27 (m, 2H), 1.19 (t, J = 7.2 Hz, 1H). ^{13}C NMR (151 MHz, DMSO) δ 173.28, 170.38, 169.34, 167.22, 167.12, 166.00, 161.58, 159.59, 157.08, 155.52, 141.96, 141.83, 140.26, 137.42, 133.49, 133.40, 132.99, 127.36, 126.80, 126.46, 125.41, 120.84, 117.26, 116.51, 113.83, 68.09, 55.94, 54.02, 51.40, 51.31, 49.27, 42.28, 38.77, 33.05, 31.41, 29.37, 29.18, 24.22, 23.46, 22.46, 21.43, 21.37, 18.53, 17.18, 12.94. HR-MS (ESI+) calc'd for $C_{45}H_{46}N_8O_{10}$: 858.9090, found [M+H]+ 859.3403.

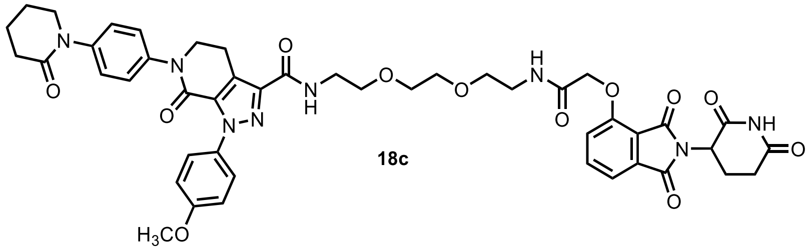

18c

N-(2-(2-(2-(2-((2-(2,6-dioxopiperidin-3-yl)-1,3-dioxoisoindolin-4-yl)oxy)acetamido)ethoxy)ethoxy)ethyl)-1-(4-methoxyphenyl)-7-oxo-6-(4-(2-oxopiperidin-1-yl)phenyl)-4,5,6,7-tetrahydro-1H-pyrazolo[3,4-c]pyridine-3-carboxamide (18c)

General Procedure 1A. Reaction scale: Thalidomide-O-acetamido-PEG2-C2-amine 13 mg (26.0 µmol, 1.0 eq) and 1-(4-methoxyphenyl)-7-oxo-6-(4-(2-oxopiperidin-1-yl)phenyl)-4,5,6,7-tetrahydro-1H-pyrazolo[3,4-c]pyridine-3-carboxylic acid 13 mg (26.0 µmol,1.0 eq). Purified by pTLC (7% MeOH/DCM) to afford **18c** as an off-white solid (11 mg, 48%). ^{1}H NMR (600 MHz, $CDCl_3$) δ 8.91 (s, 1H), 7.69 – 7.62 (m, 1H), 7.56 – 7.51 (m, 1H), 7.47 (d, *J* = 7.3 Hz, 1H), 7.38 (d, *J* = 7.2, Hz, 2H), 7.28 – 7.23 (m, 2H), 7.19 – 7.15 (m, 1H), 7.10 (d, *J* = 8.4 Hz, 1H), 6.87 – 6.83 (m, 1H), 4.86 – 4.81 (m, 2H), 4.56 (s, 1H), 4.04 – 4.01 (m, 2H), 3.74 (s, 2H), 3.63 – 3.51 (m, 10H), 3.50 – 3.42 (m, 2H), 3.31 – 3.23 (m, 2H), 2.78 – 2.74 (m, 2H), 2.73 – 2.65 (m, 2H), 2.65 – 2.54 (m, 1H), 2.52 – 2.43 (m, 1H), 2.06 – 1.98 (m, 1H), 1.89 – 1.82 (m, 3H), 1.42 –1.34 (m, 2H), 1.32 –1.26 (m, 2H). ^{13}C NMR (151 MHz, DMSO) δ 173.28, 170.37, 169.34, 167.36, 167.22, 165.92, 161.70, 159.61, 157.04, 155.42, 141.83, 141.66, 140.24, 137.41, 133.47, 132.94, 127.32, 126.80, 126.46, 125.46, 120.75, 117.18, 116.49, 113.85, 70.08, 69.94, 69.30, 69.25, 67.91, 55.93, 53.98, 51.38, 51.30, 49.27, 38.87, 38.62, 33.05, 31.41, 23.46, 22.46, 21.41, 21.37, 18.51, 17.18. HR-MS (ESI+) calc'd for $C_{46}H_{48}N_8O_{12}$: 904.9340, found $[M+H]^+$ 905.3461.

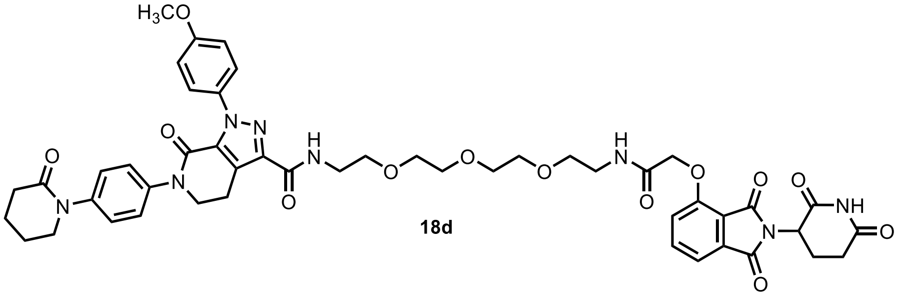

18d

N-(1-((2-(2,6-dioxopiperidin-3-yl)-1,3-dioxoisoindolin-4-yl)oxy)-2-oxo-6,9,12-trioxa-3-azatetradecan-14-yl)-1-(4-methoxyphenyl)-7-oxo-6-(4-(2-oxopiperidin-1-yl)phenyl)-4,5,6,7-tetrahydro-1H-pyrazolo[3,4-c]pyridine-3-carboxamide (18d)

General Procedure 1A. Reaction scale: Thalidomide-O-acetamido-PEG3-C2-amine 16 mg (29.5 µmol, 1.0 eq) and 1-(4-methoxyphenyl)-7-oxo-6-(4-(2-oxopiperidin-1-yl)phenyl)-4,5,6,7-tetrahydro-1H-pyrazolo[3,4-c]pyridine-3-carboxylic acid 13 mg (30.1 µmol,1.0 eq). Purified by pTLC (7% MeOH/DCM) to afford **18d** as an off-white solid (16 mg, 52%). ^{1}H NMR (600 MHz, $CDCl_3$) δ 9.09 (s, 1H), 7.74 – 7.72 (m, 1H), 7.63 – 7.61 (m, 1H), 7.52 (d, *J* = 7.3 Hz, 1H), 7.50 – 7.46 (m, 2H), 7.46 – 7.42 (m, 1H), 7.32 (d, *J* = 8.7 Hz, 2H), 7.24 (d, *J* = 8.7 Hz, 2H), 7.17 (d, *J* = 8.4 Hz, 1H), 6.96 – 6.91 (m 2H), 4.86 – 4.80 (m, 1H), 4.62 (s, 2H), 4.14 – 4.01 (m, 2H), 3.80 (s, 3H), 3.73 – 3.62 (m, 9H), 3.62 – 3.54 (m, 7H), 3.53 – 3.44 (m, 1H), 3.36 – 3.32 (m, 2H), 2.78 – 2.63 (m, 2H), 2.58 – 2.53(m, 2H), 2.05 – 1.97 (m, 1H), 1.96 – 1.91 (m, 4H), 1.87 (s, 1H), 1.44 – 1.35 (m, 1H). ^{13}C NMR (151 MHz, DMSO) δ 173.28, 170.37, 169.36, 167.37, 167.22, 165.92, 161.70, 159.61, 157.05, 155.42, 141.83, 141.67, 140.24, 137.43, 133.47, 132.95, 127.32, 126.80, 126.47, 125.47, 120.77, 117.19, 116.52, 113.85, 70.21, 70.08, 69.97, 69.27, 69.23, 67.92, 55.93, 51.38, 51.31, 49.27, 38.87, 38.62, 33.05, 31.41, 23.45, 22.45, 21.41, 21.36. HR-MS (ESI^+) calc'd for $C_{48}H_{52}N_8O_{13}$: 948.9870, found $[M+H]^+$ 949.3722.

NMR Spectra

Headgroup intermediates

^{1}H NMR **F2**

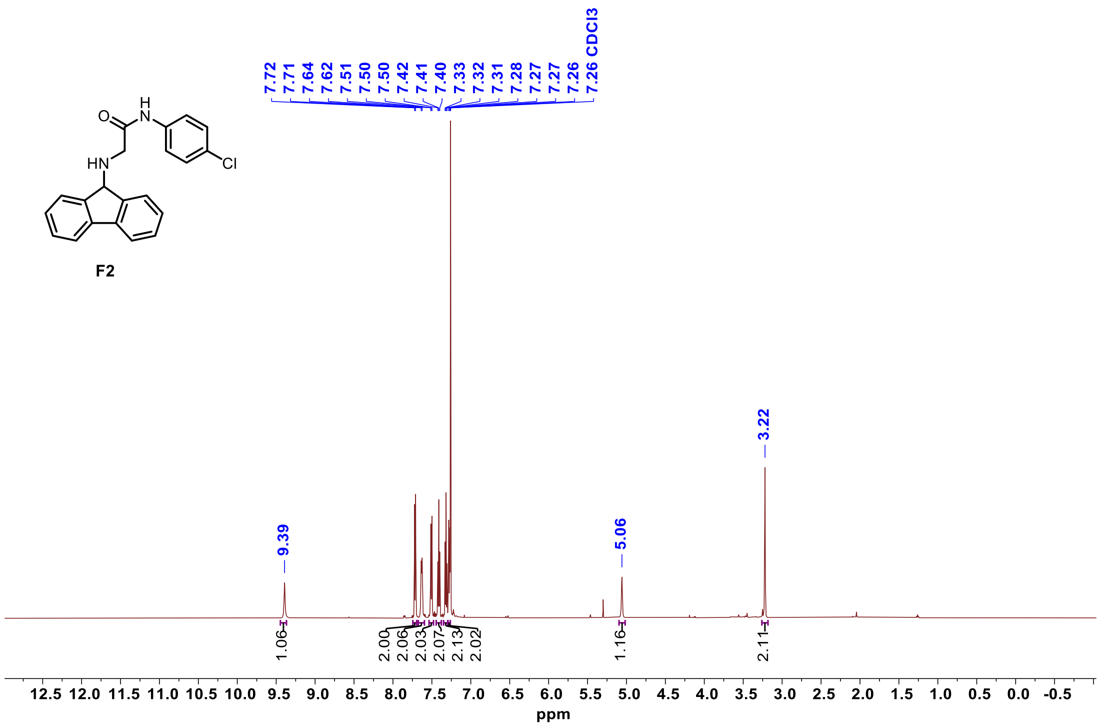

^{13}C NMR **F2**

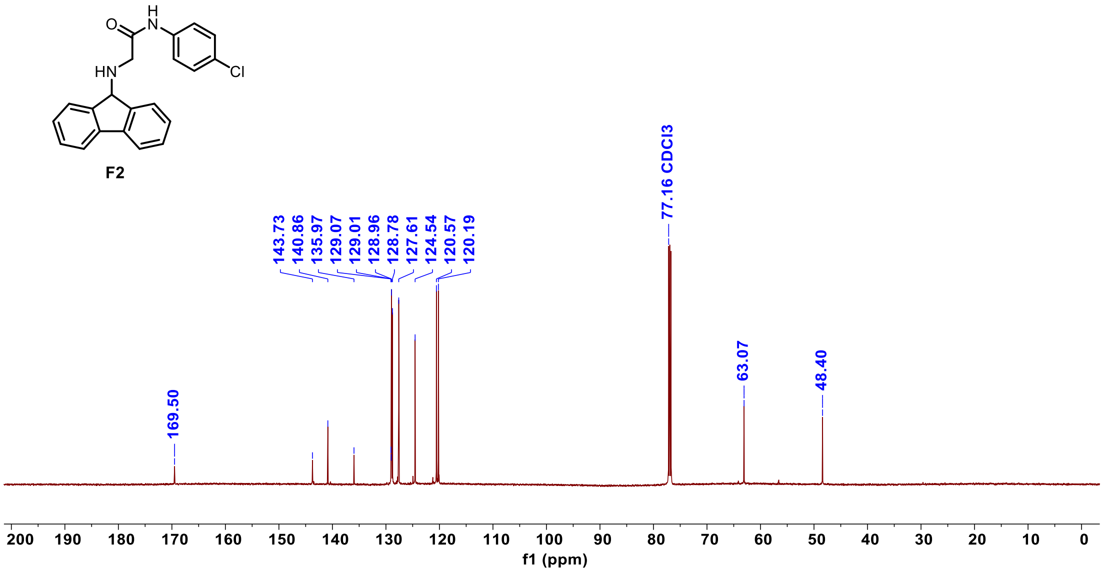

^{1}H NMR **F4**

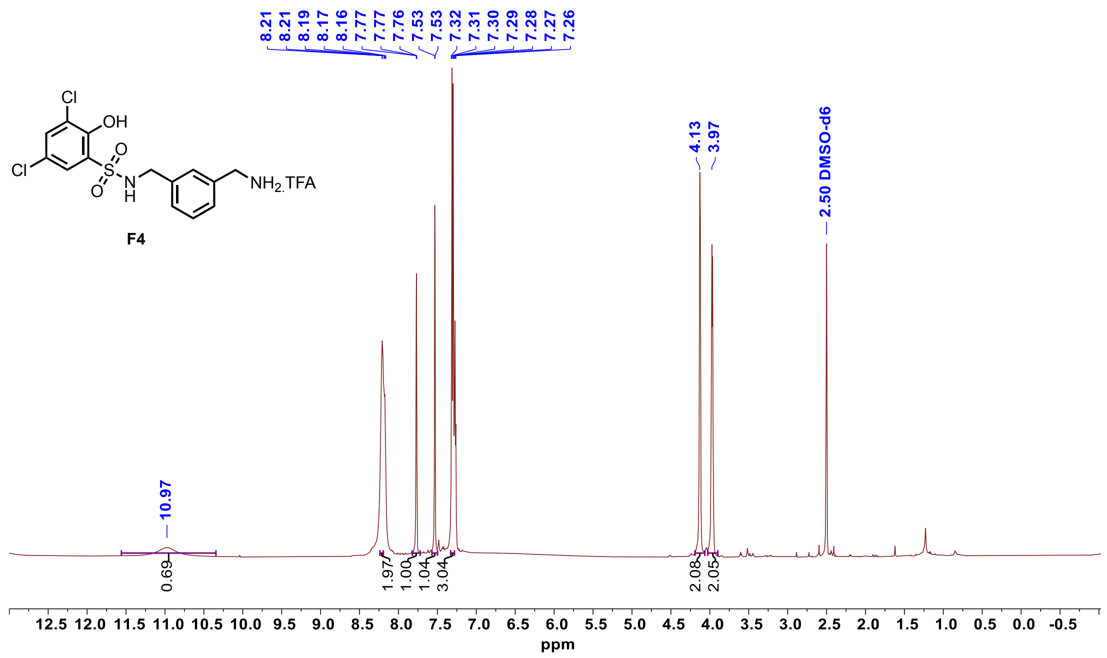

^{1}H NMR **F7**

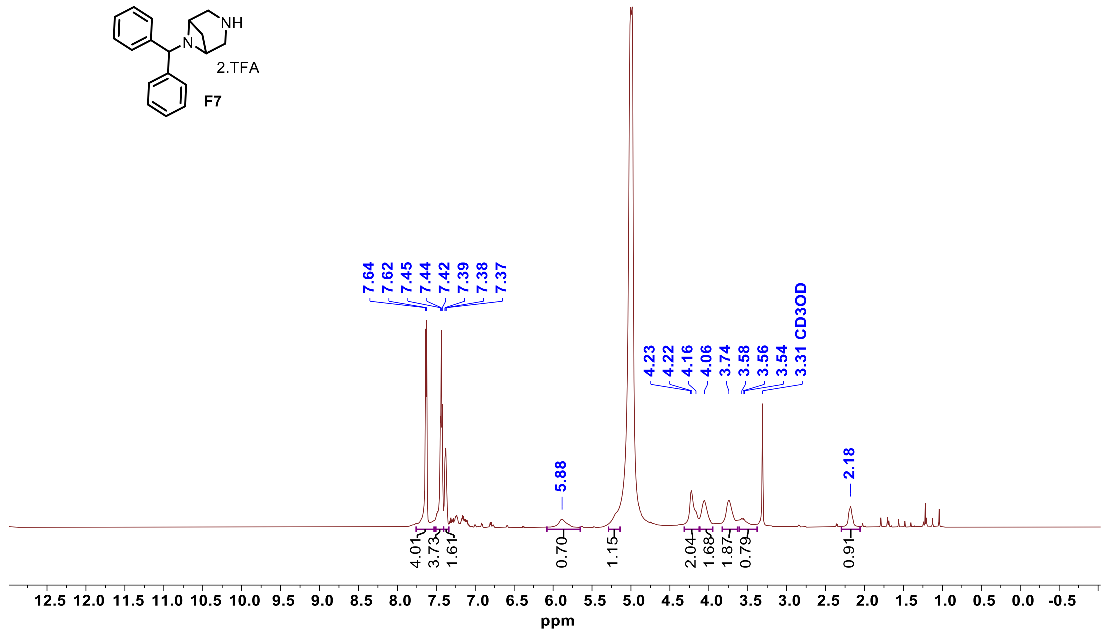

^{1}H NMR **F8**

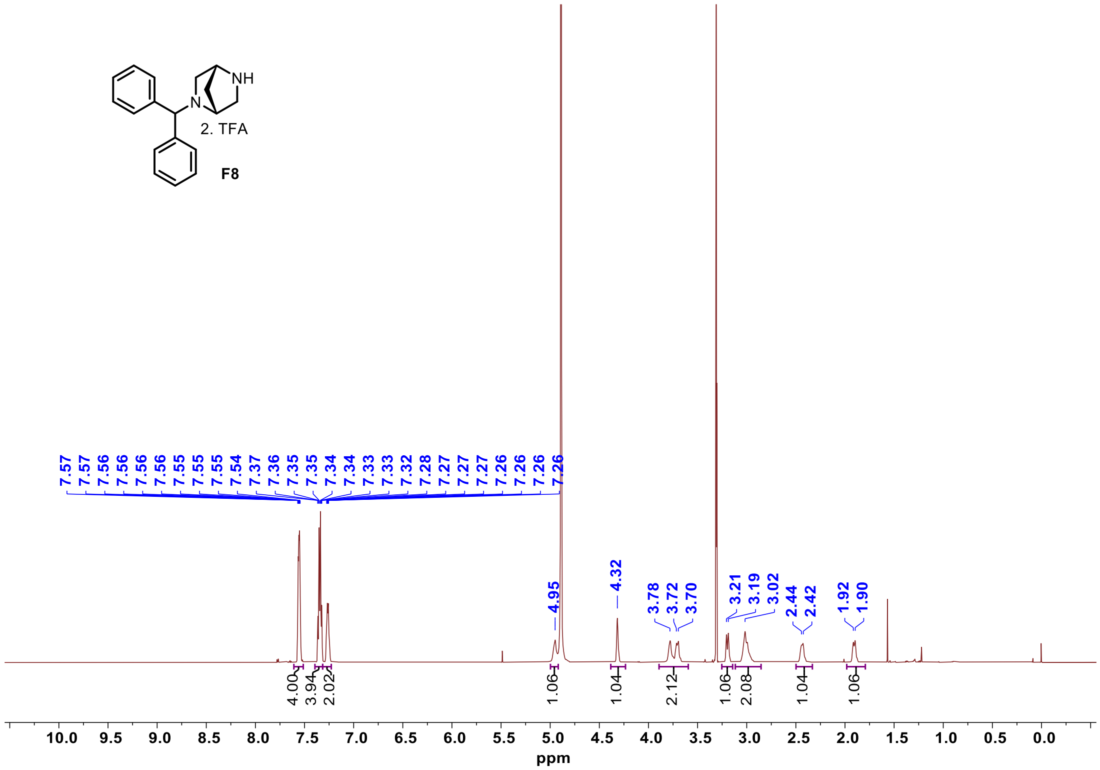

Propylated Headgroups

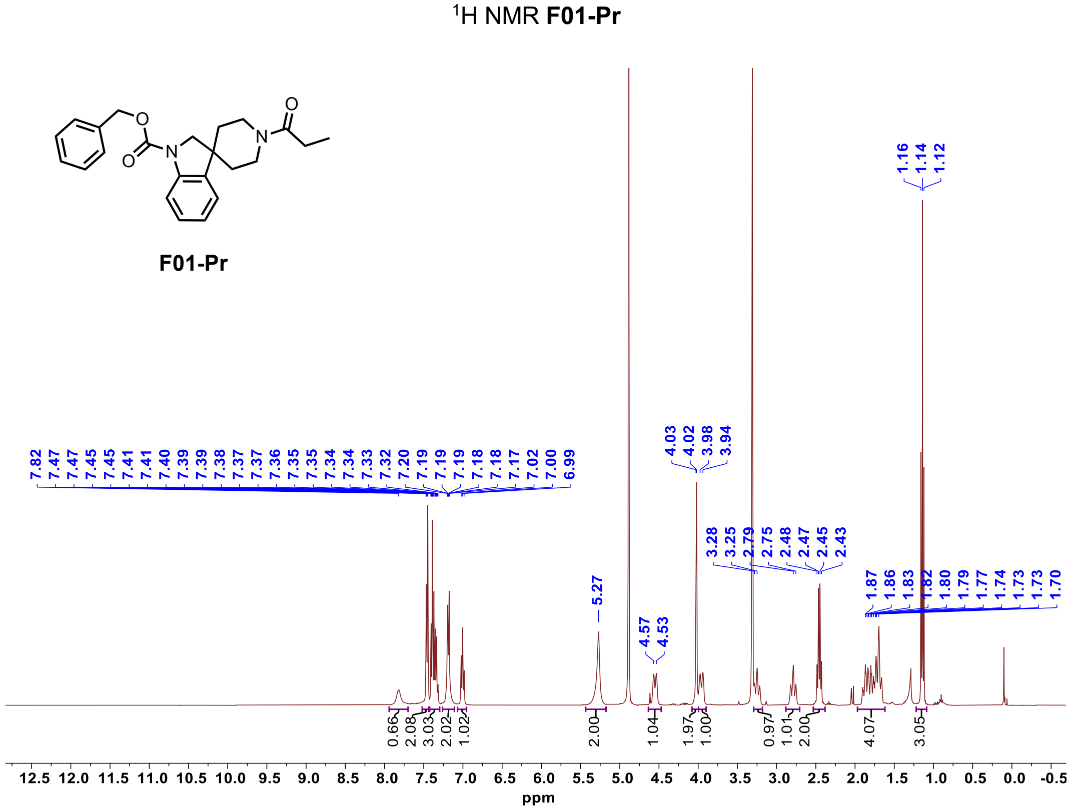

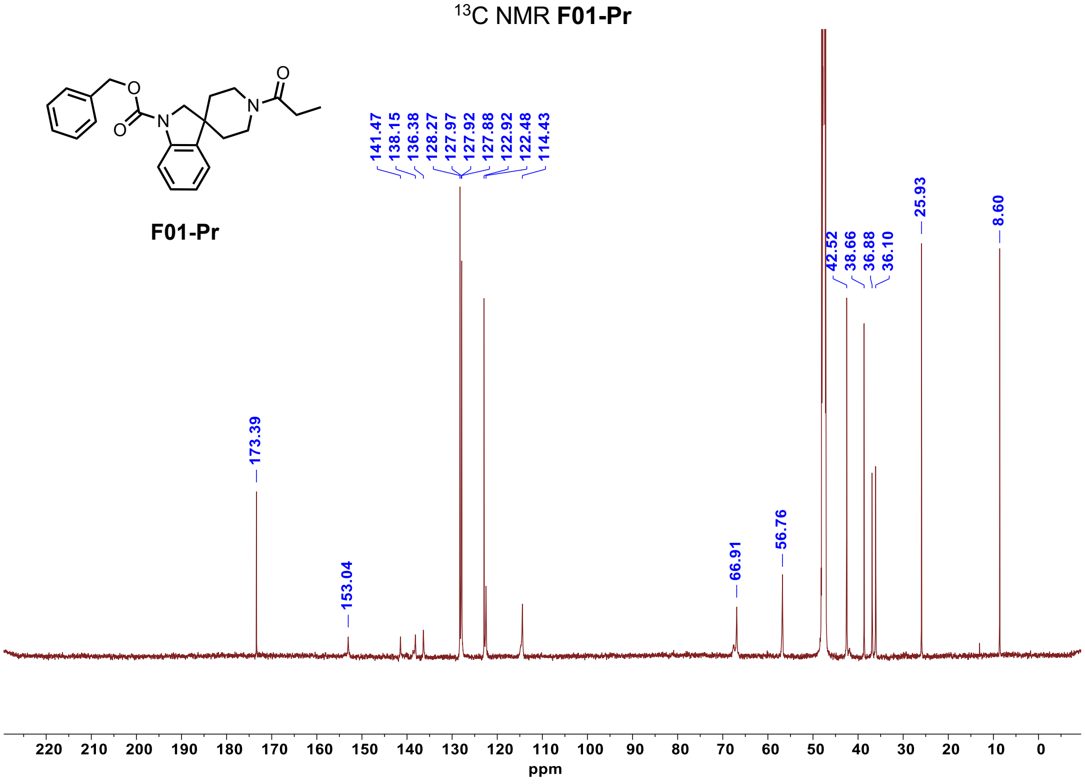

^{1}H NMR **F02-Pr**

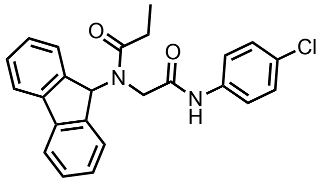

F02-Pr

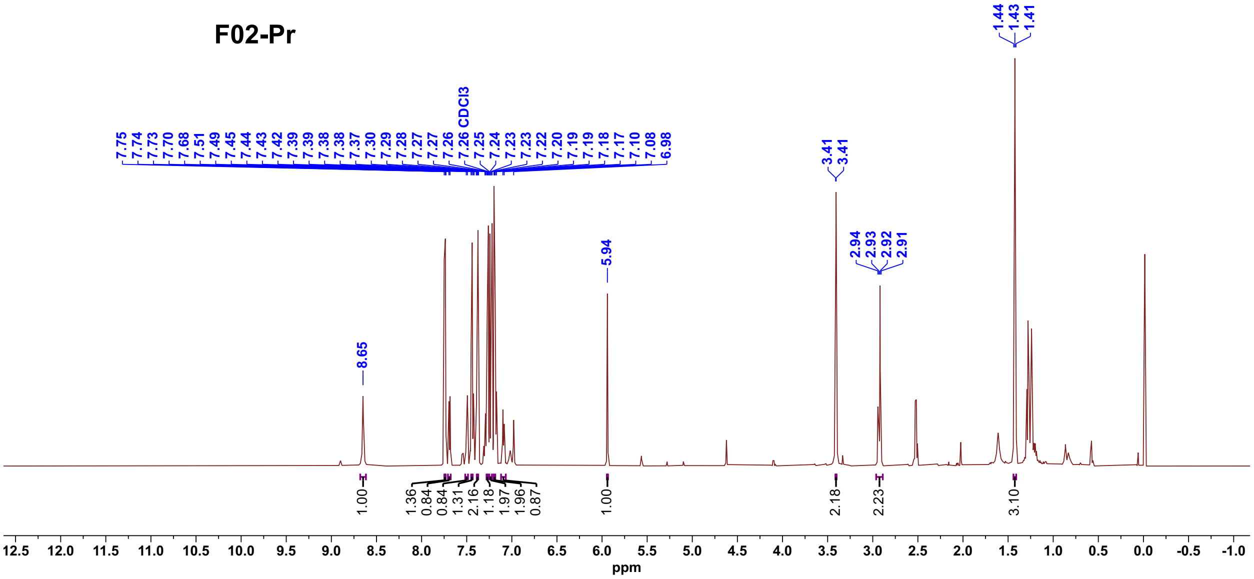

^{13}C NMR **F02-Pr**

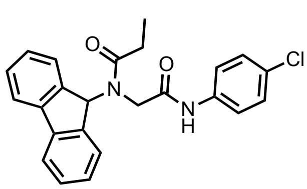

F02-Pr

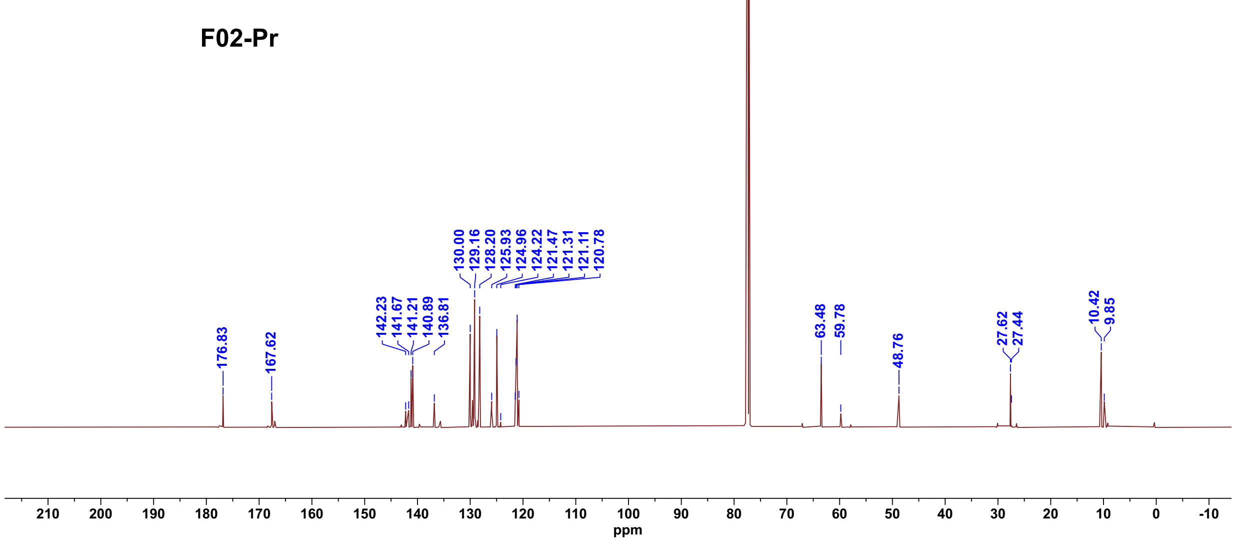

^{1}H NMR **F07-Pr**

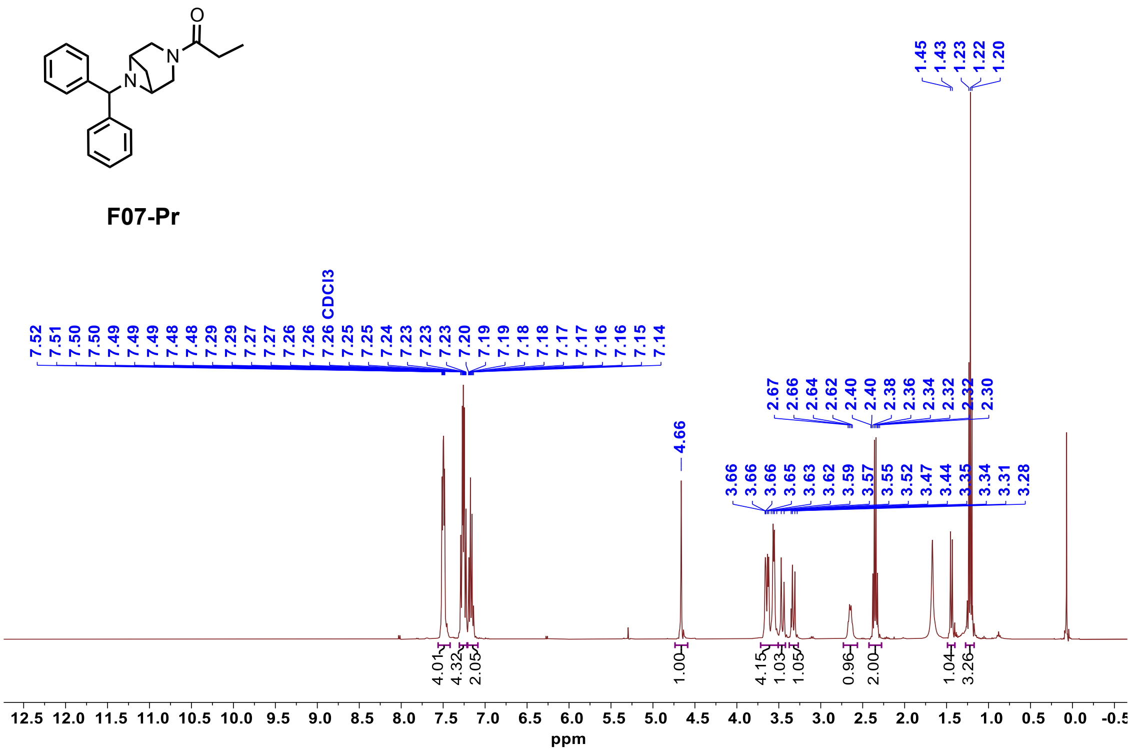

^{13}C NMR **F07-Pr**

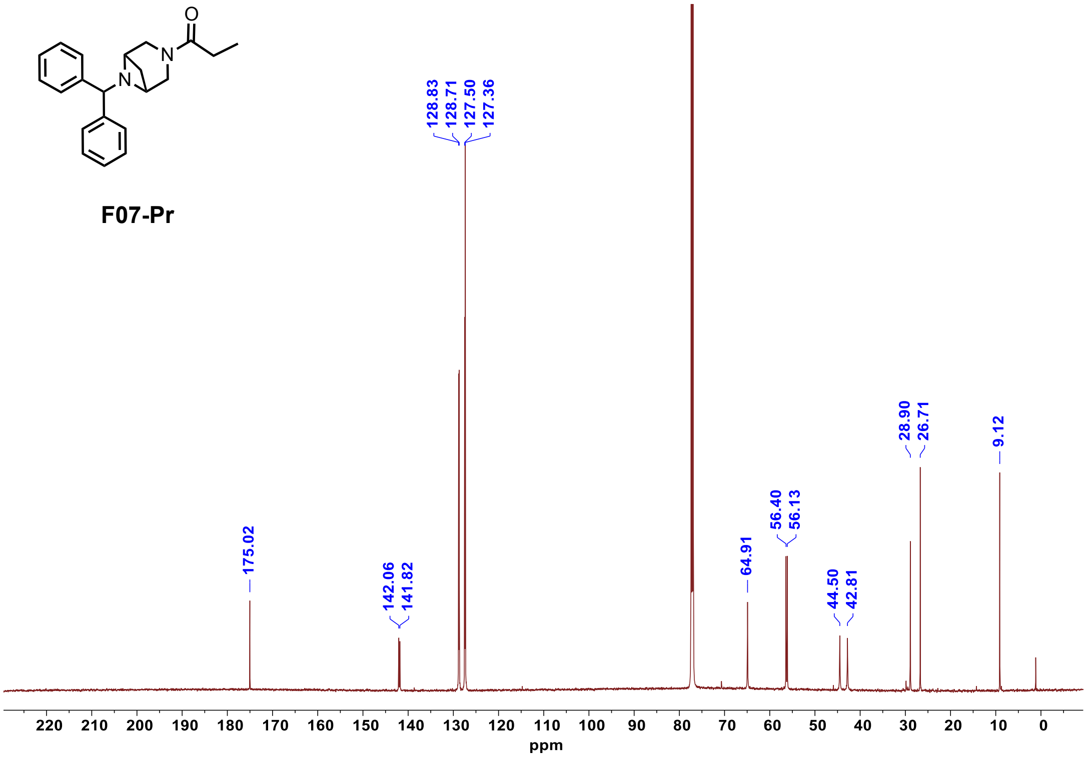

^{1}H NMR **F08-Pr**

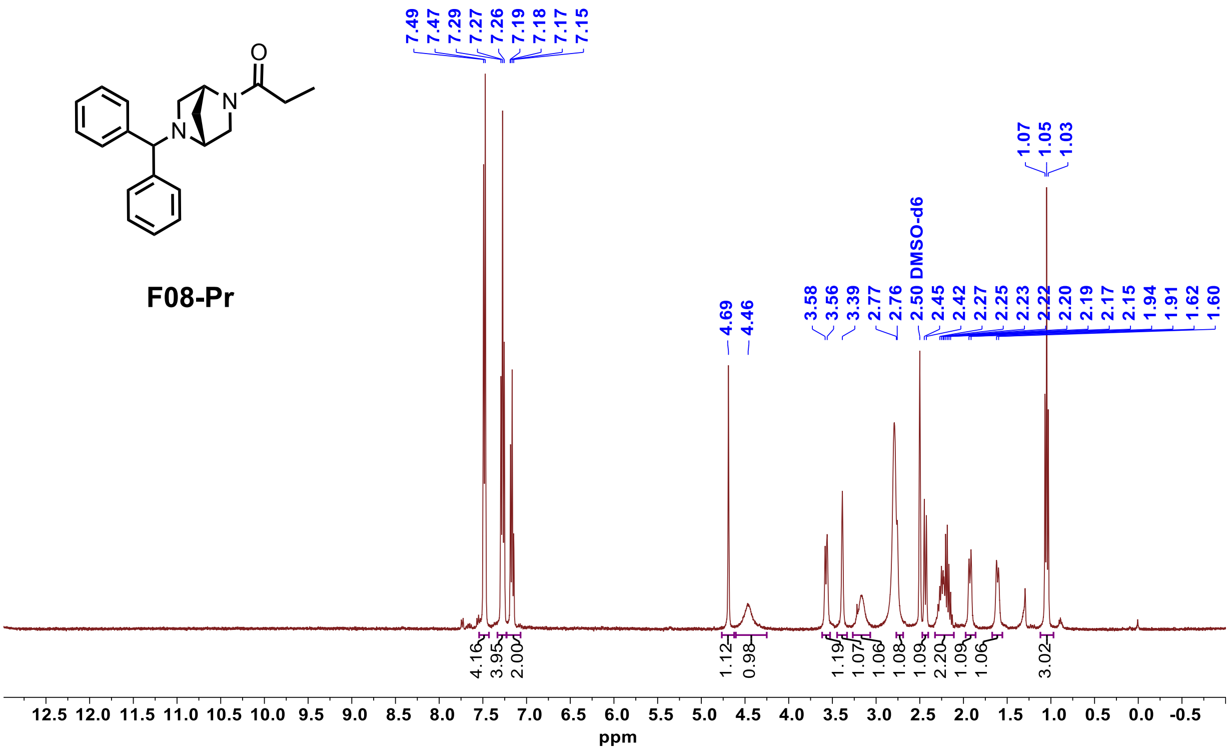

^{13}C NMR **F08-Pr**

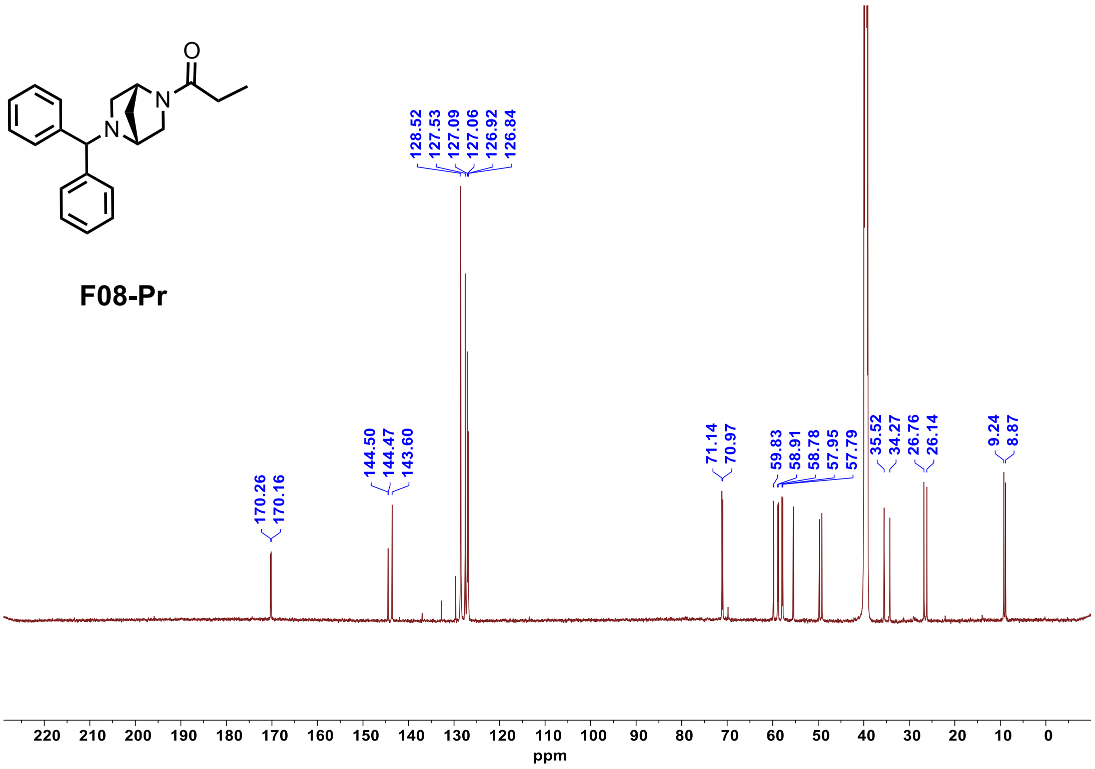

^{1}H NMR **F10-Pr**

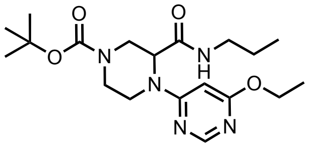

F10-Pr

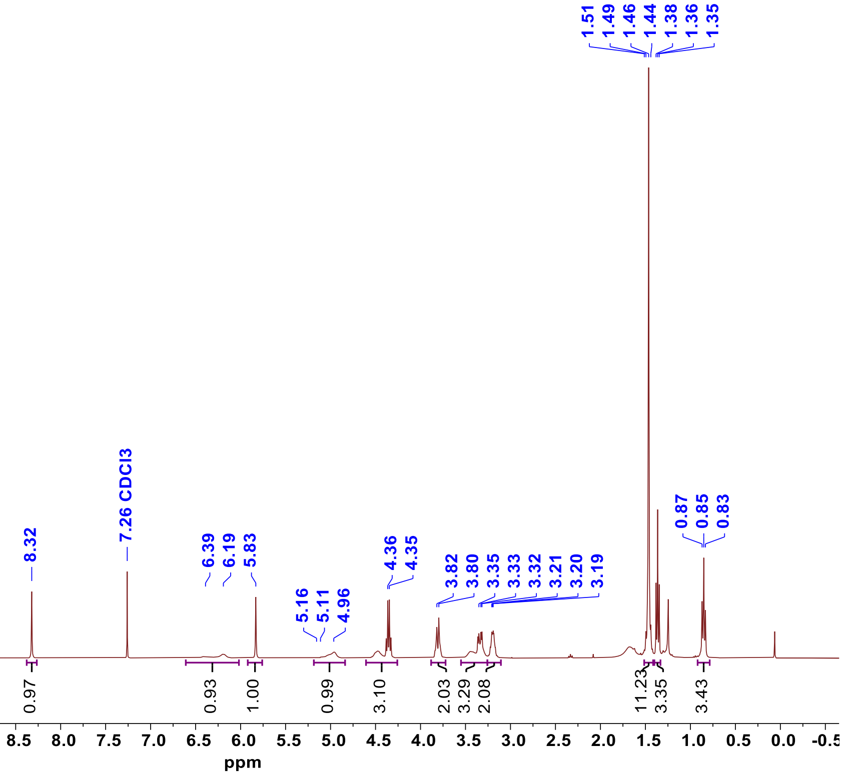

^{13}C NMR **F10-Pr**

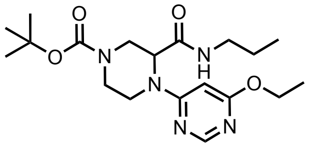

F10-Pr

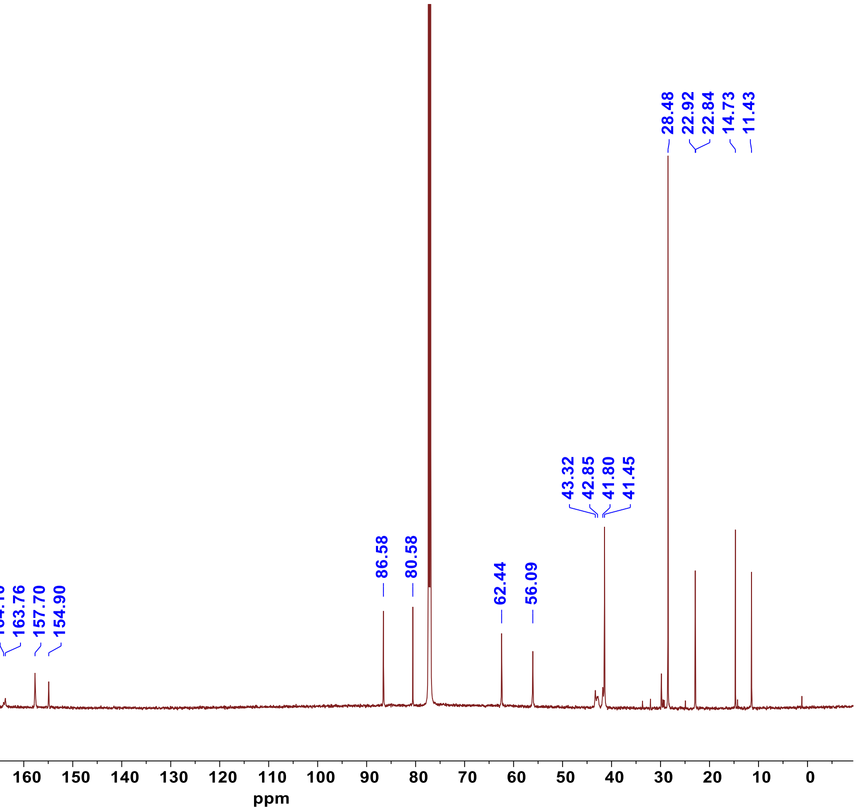

Fully-Functionalized Photoaffinity Probes

^{1}H NMR **FFF-2**

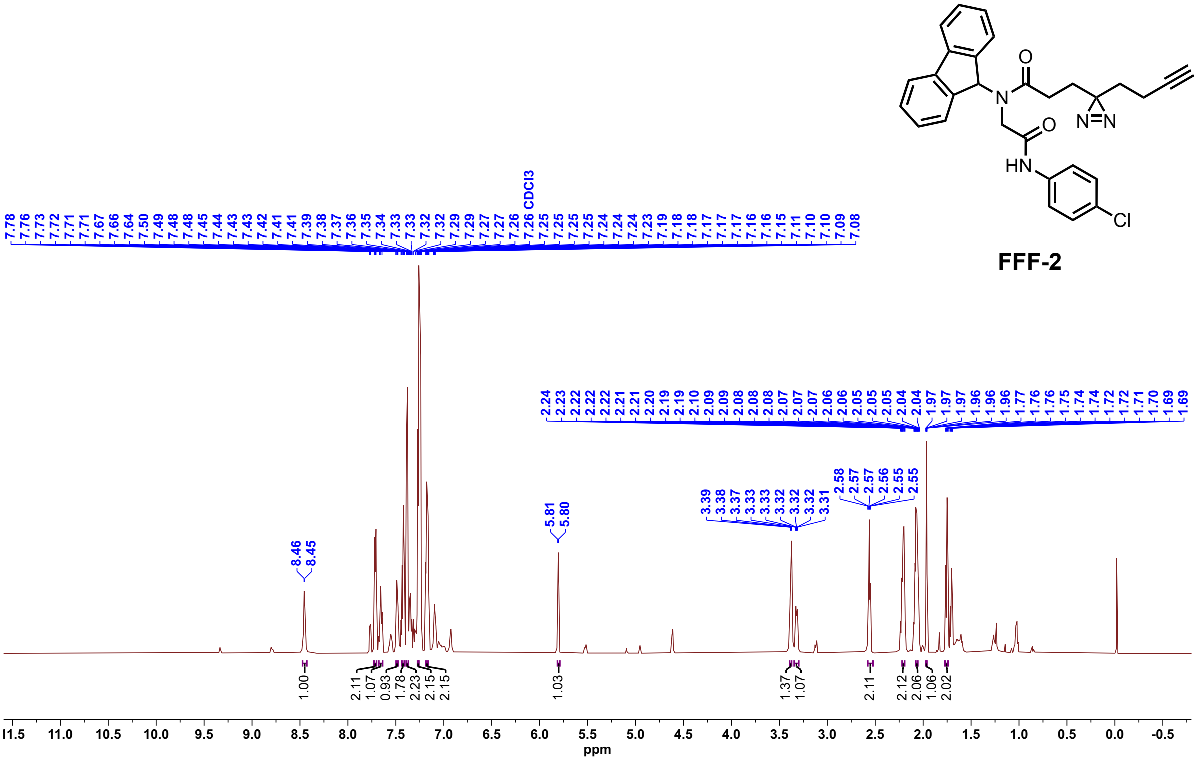

^{13}C NMR **FFF-2**

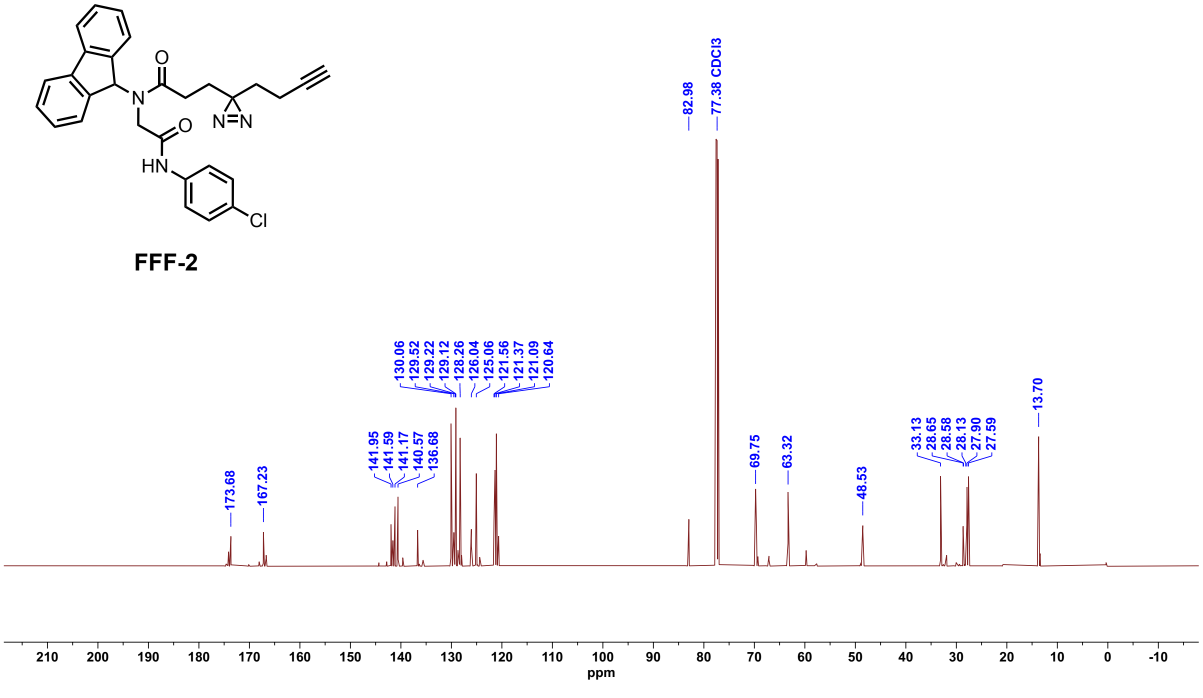

^{1}H NMR **FFF-8**

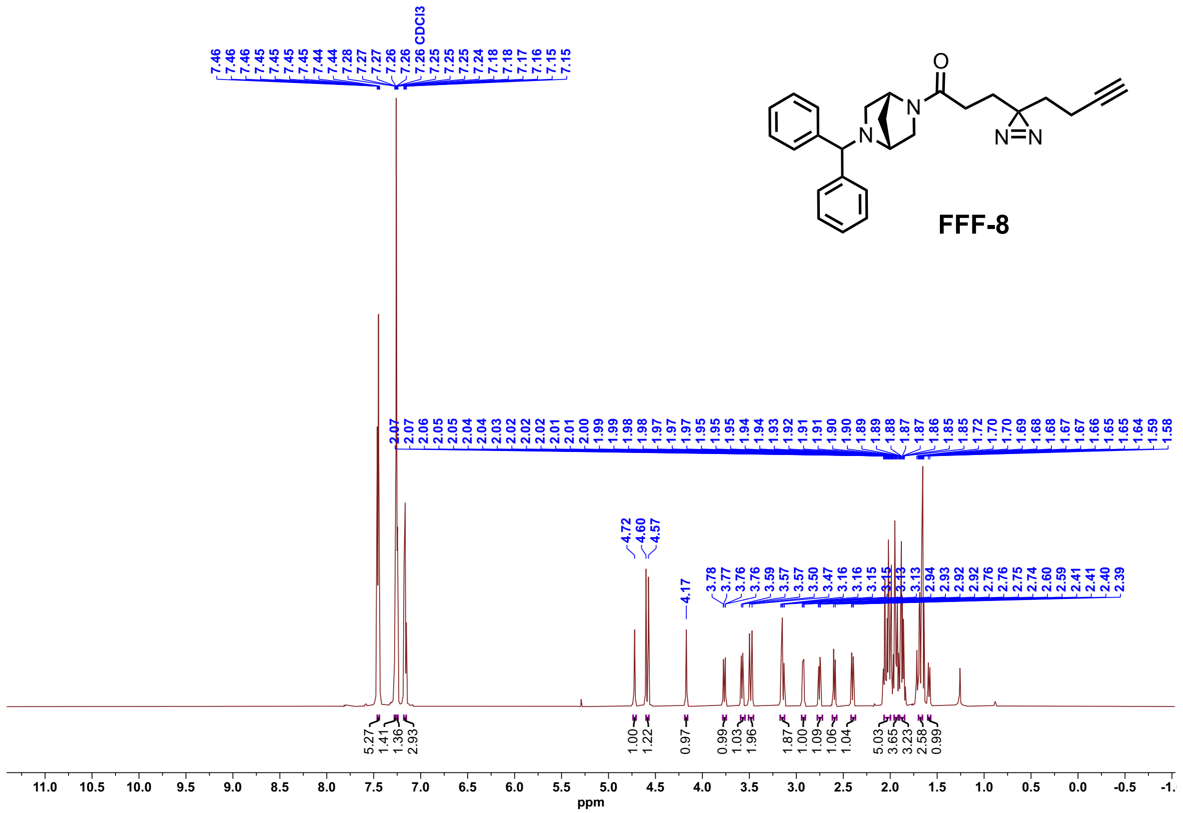

^{13}C NMR **FFF-8**

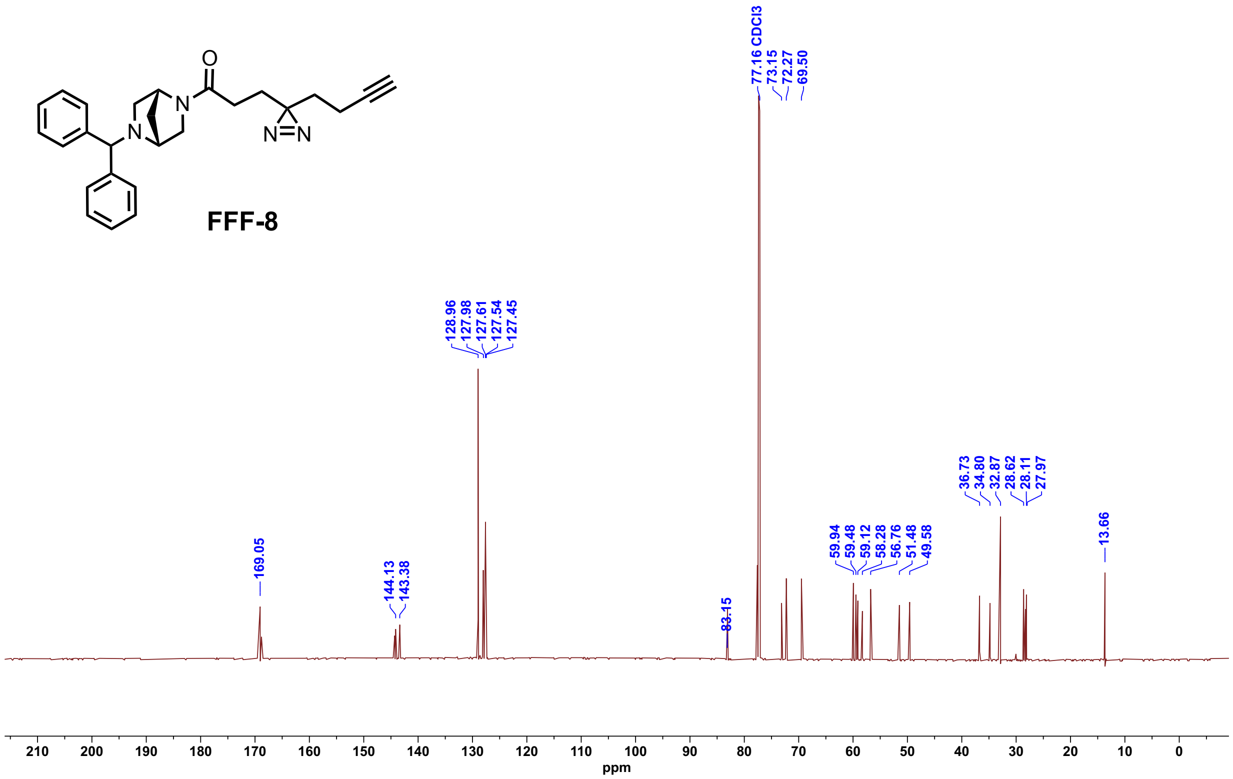

Final Degrader Compounds

^{1}H NMR **1a**

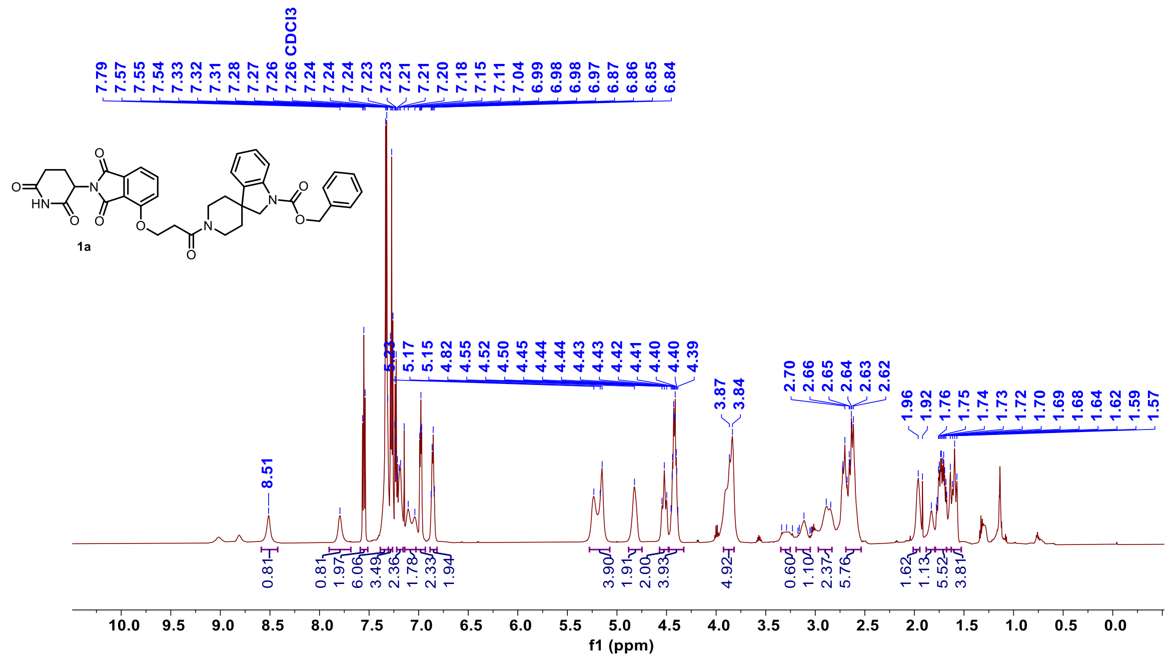

^{13}C NMR **1a**

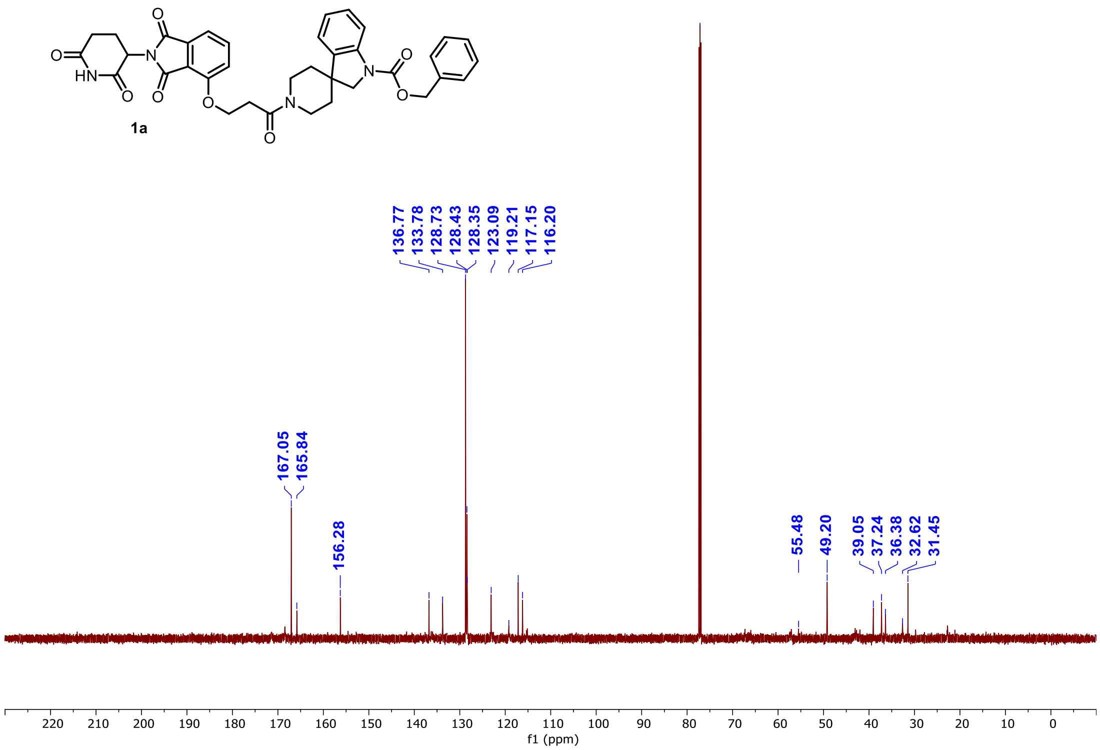

^{1}H NMR **1b**

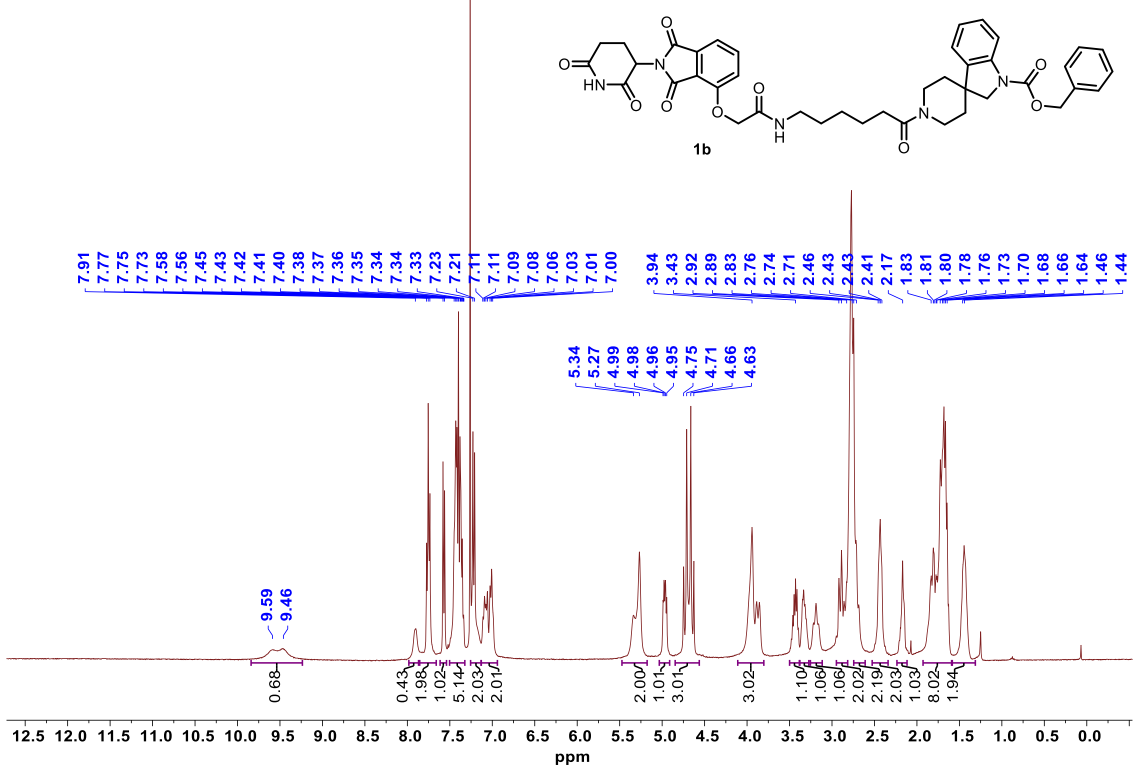

^{13}C NMR **1b**

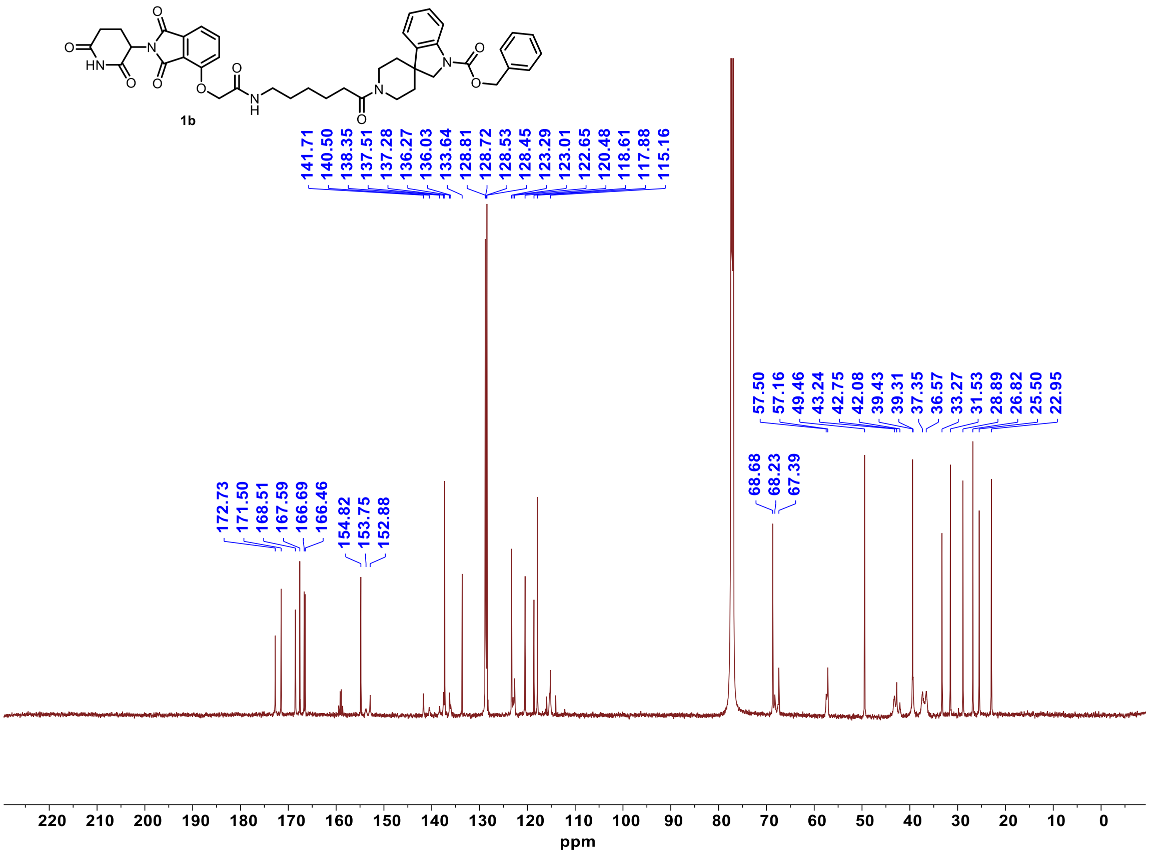

^{1}H NMR **1c**

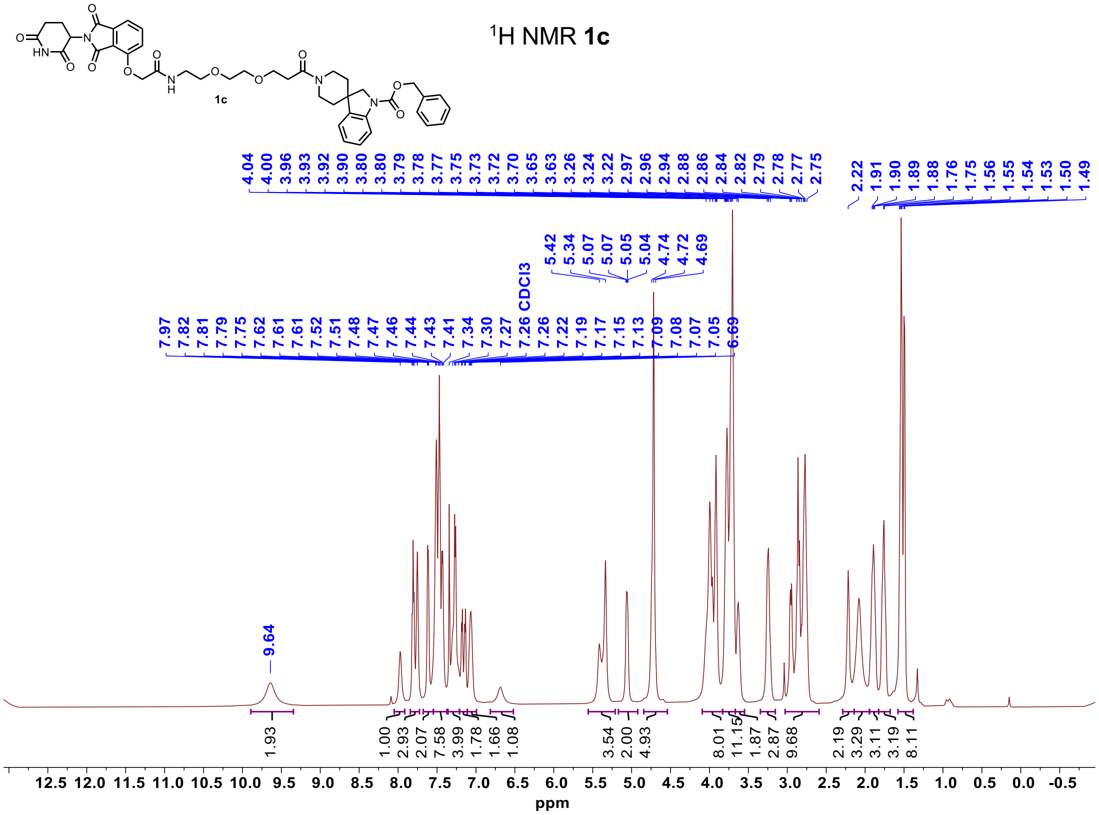

^{13}C NMR **1c**

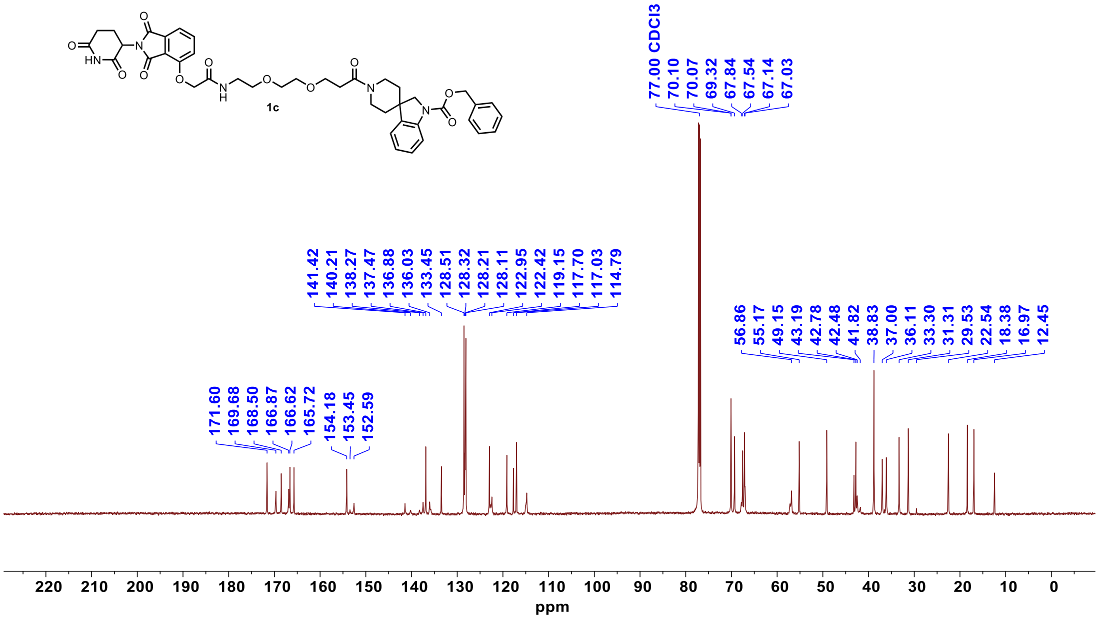

^{1}H NMR **1c-neg**

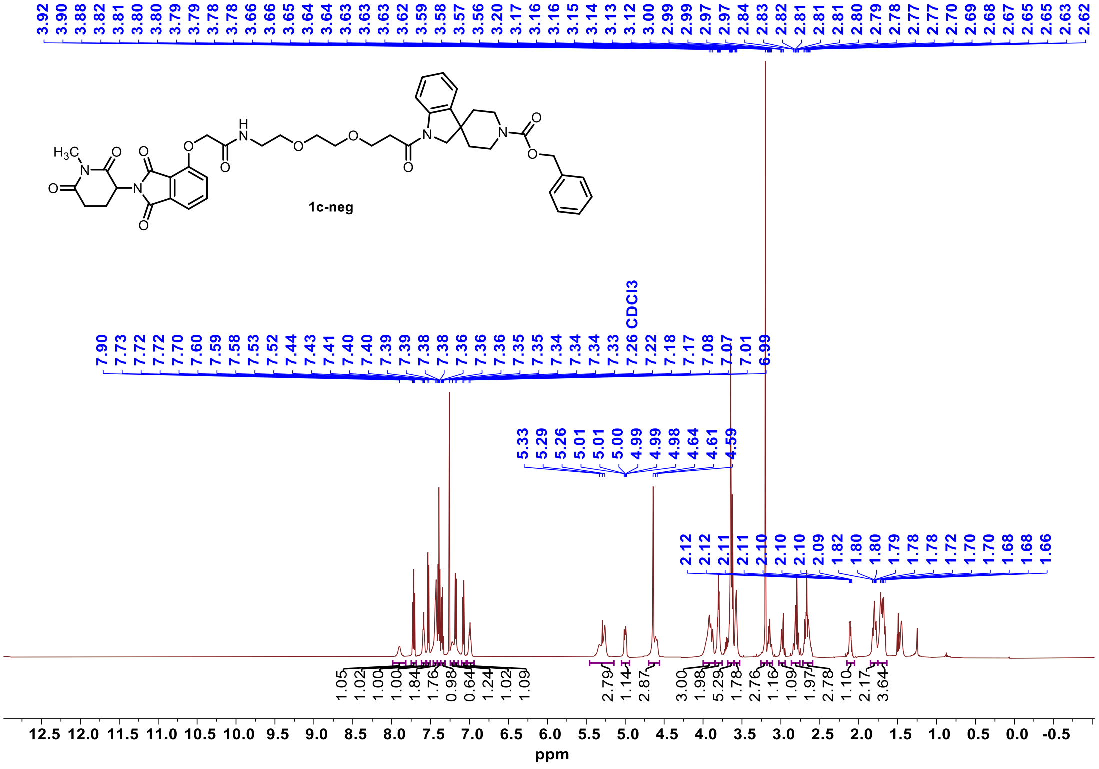

^{13}C NMR **1c-neg**

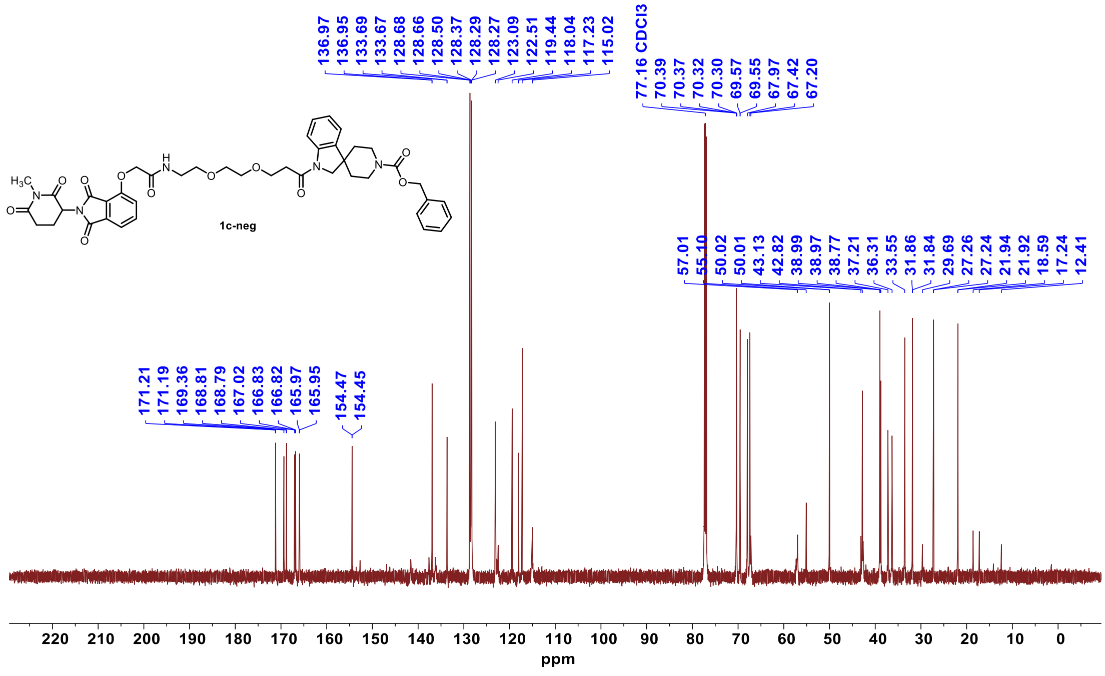

^{1}H NMR **1d**

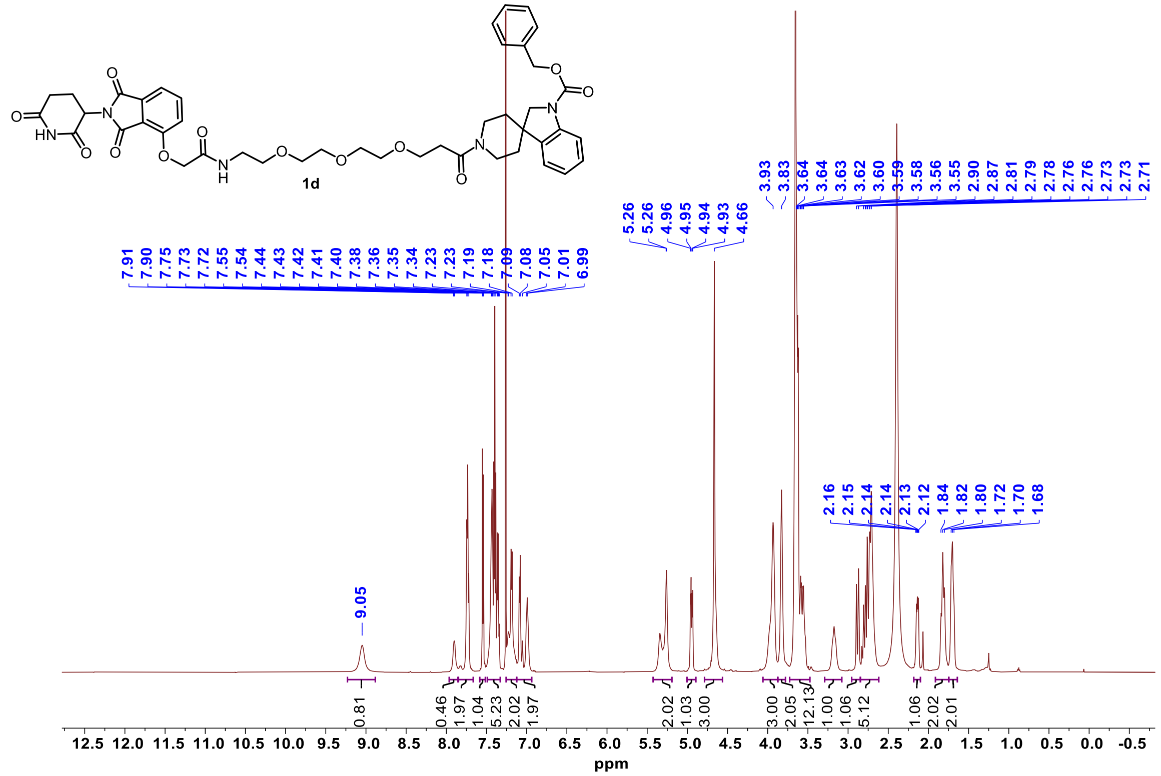

^{13}C NMR **1d**

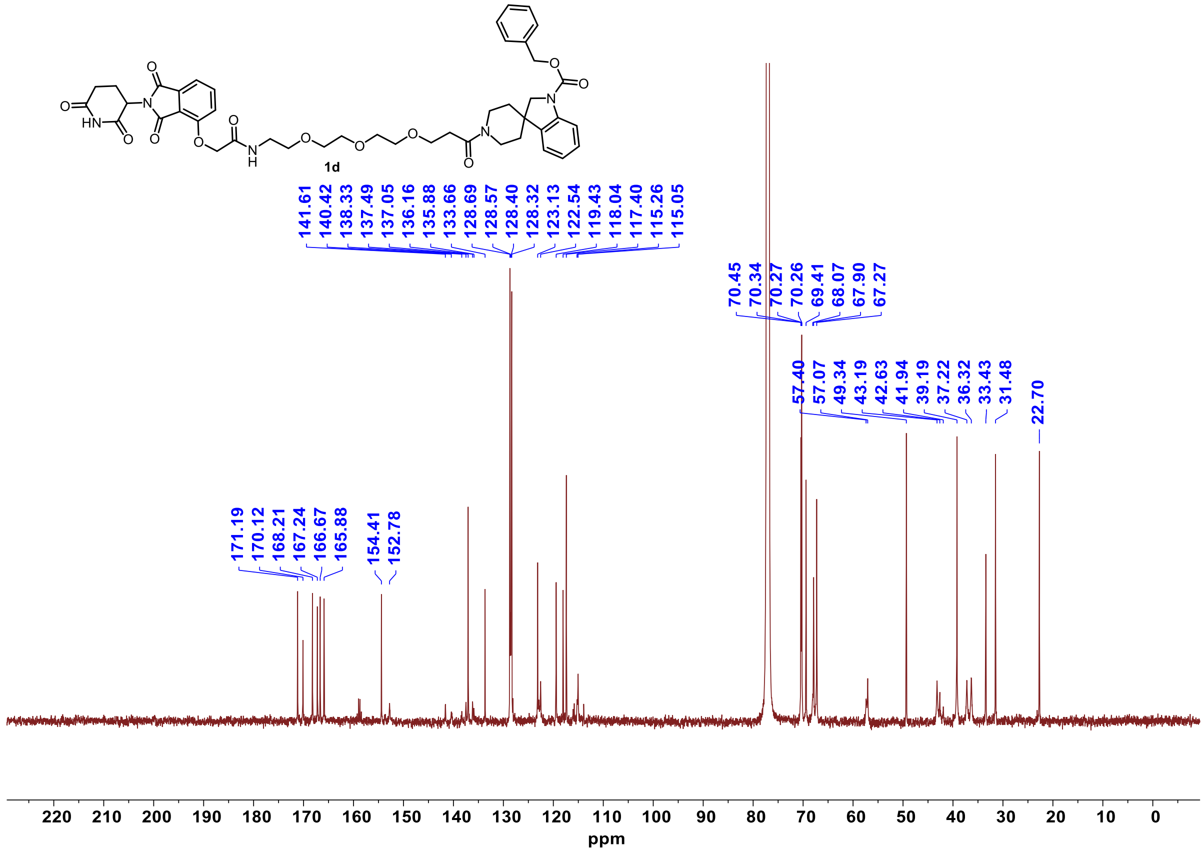

^{1}H NMR **2a**

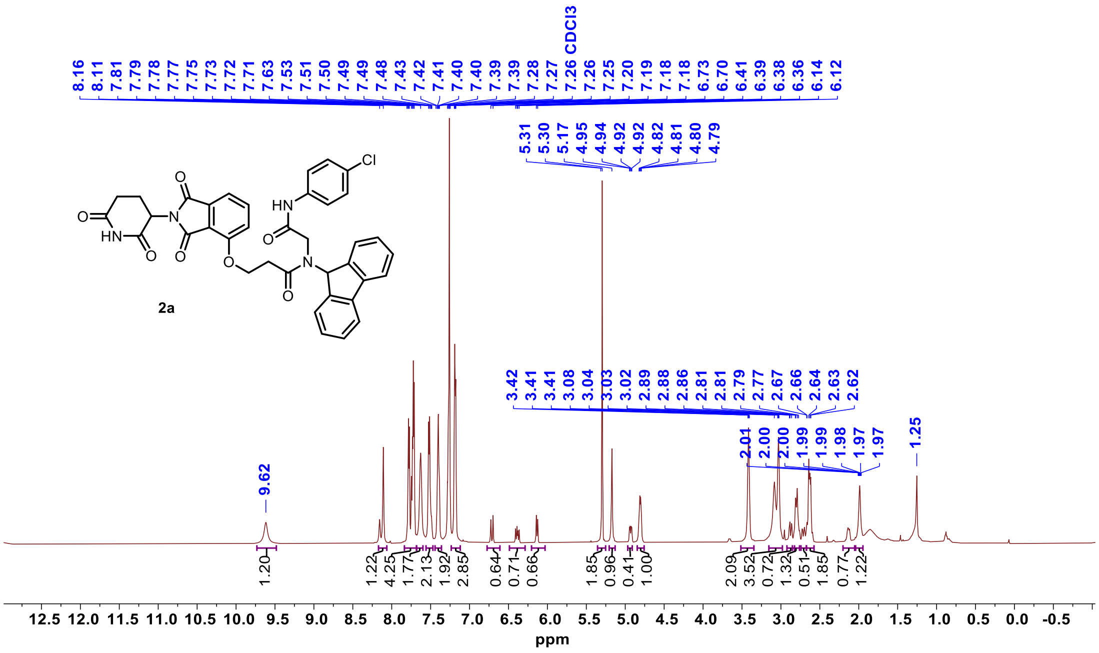

^{13}C NMR **2a**

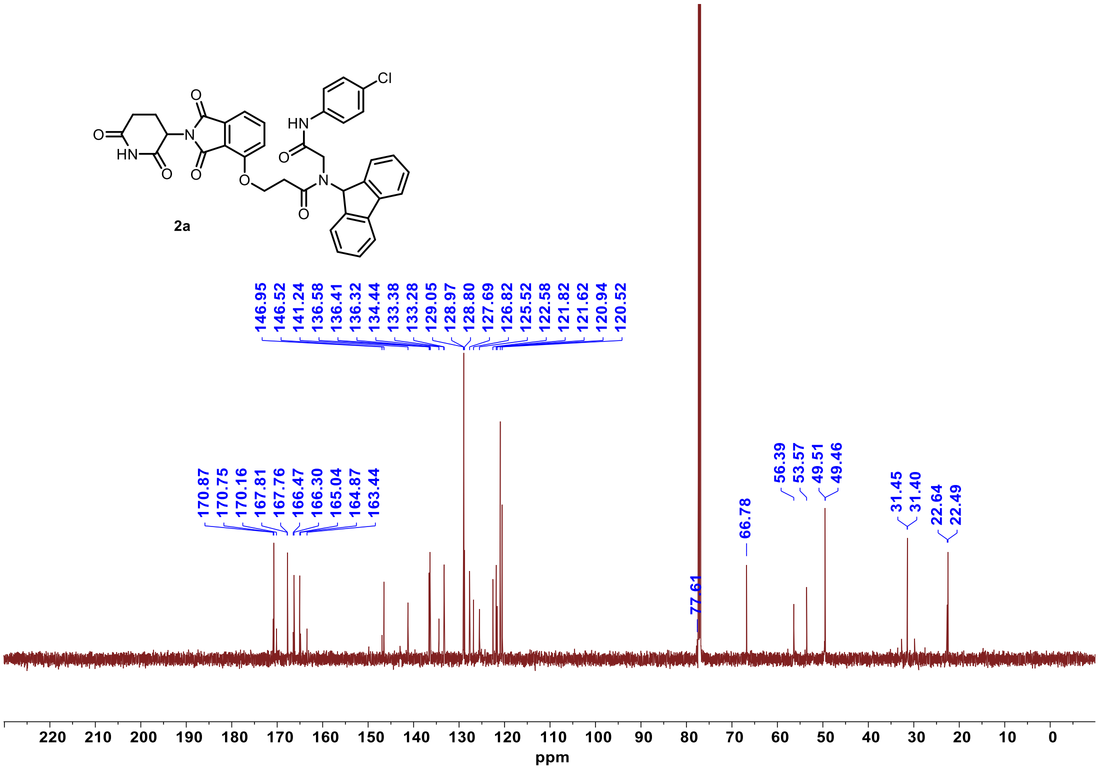

^{1}H NMR **2a-neg**

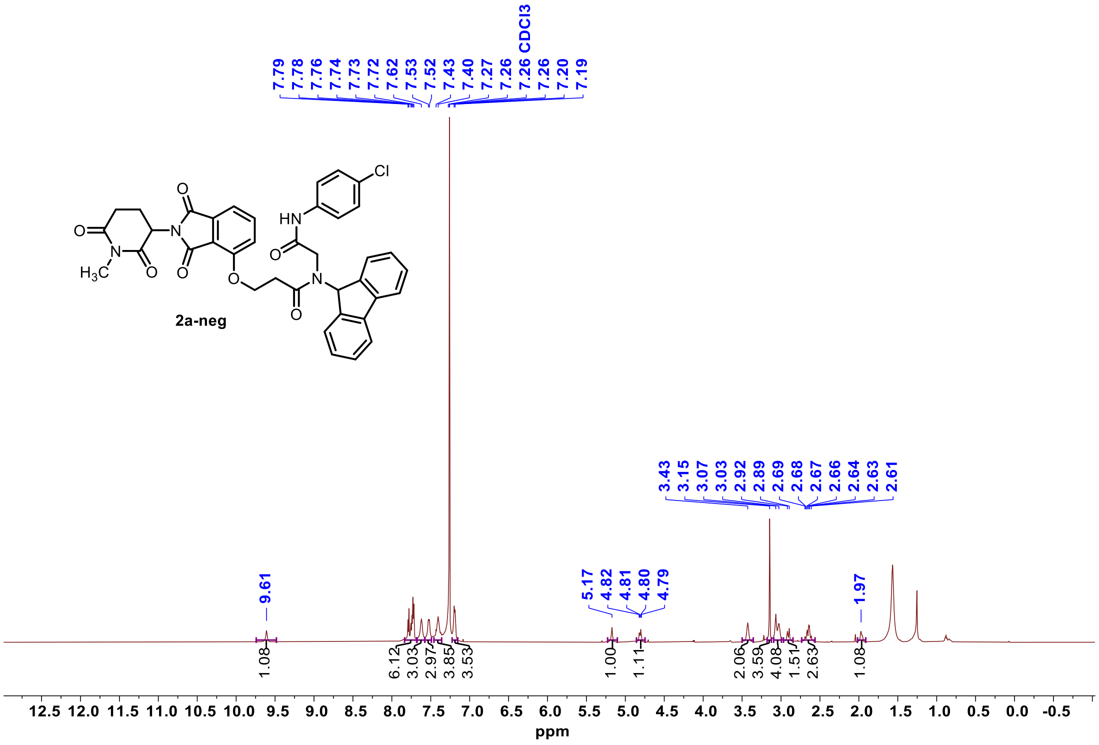

^{13}C NMR **2a-neg**

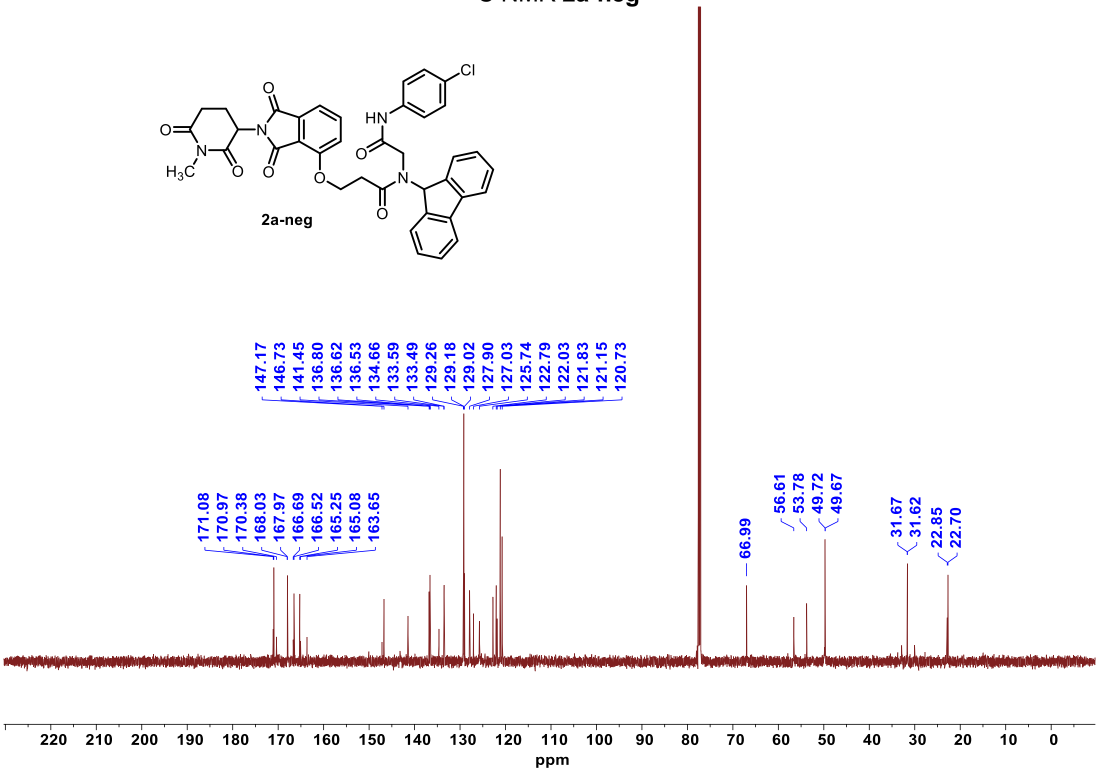

^{1}H NMR **2b**

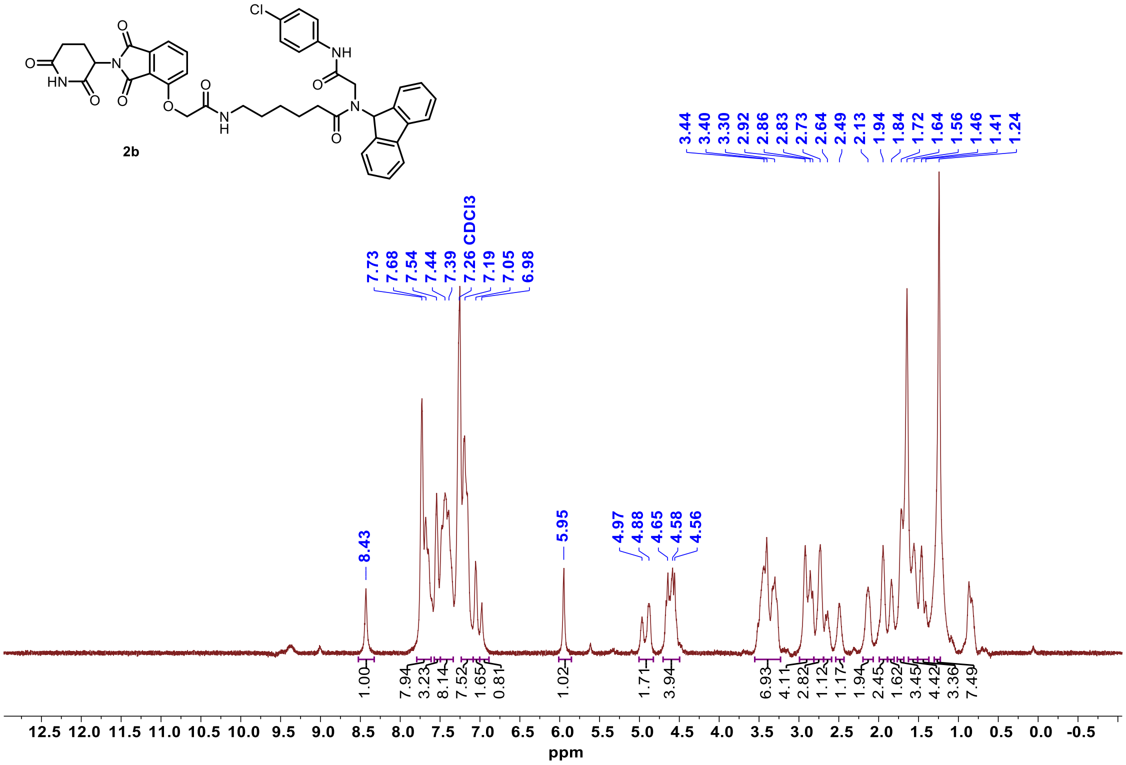

2b

^{13}C NMR **2b**

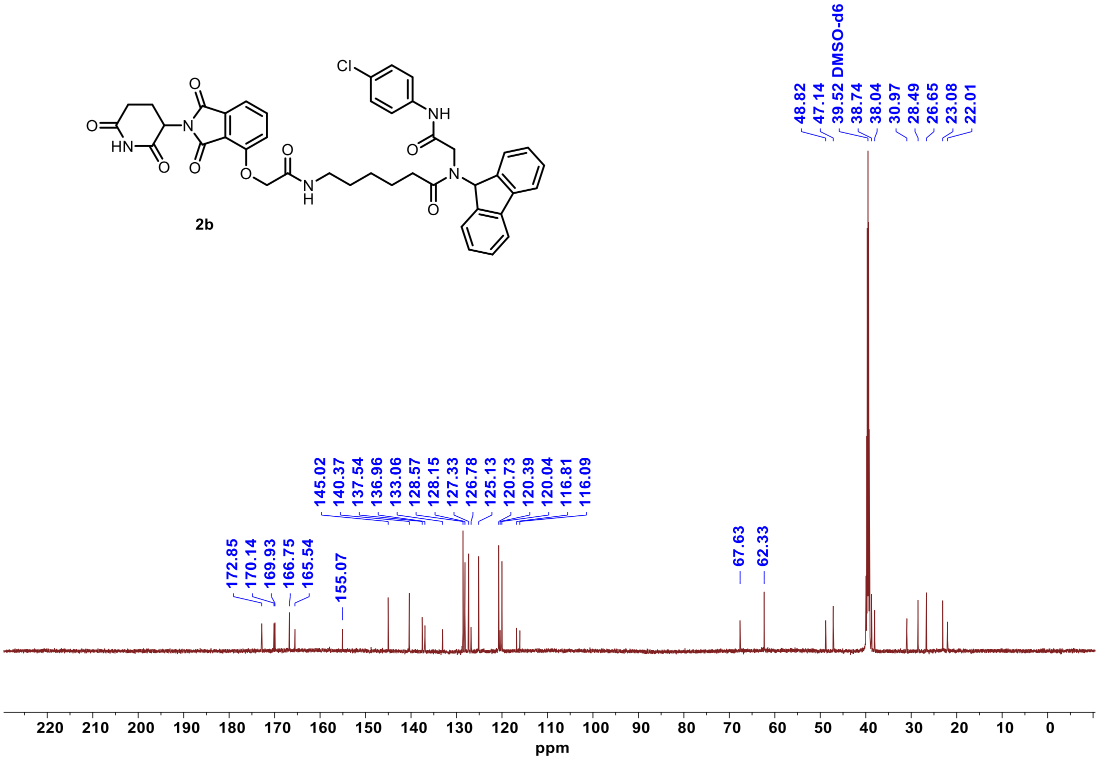

2b

^{1}H NMR **2c**

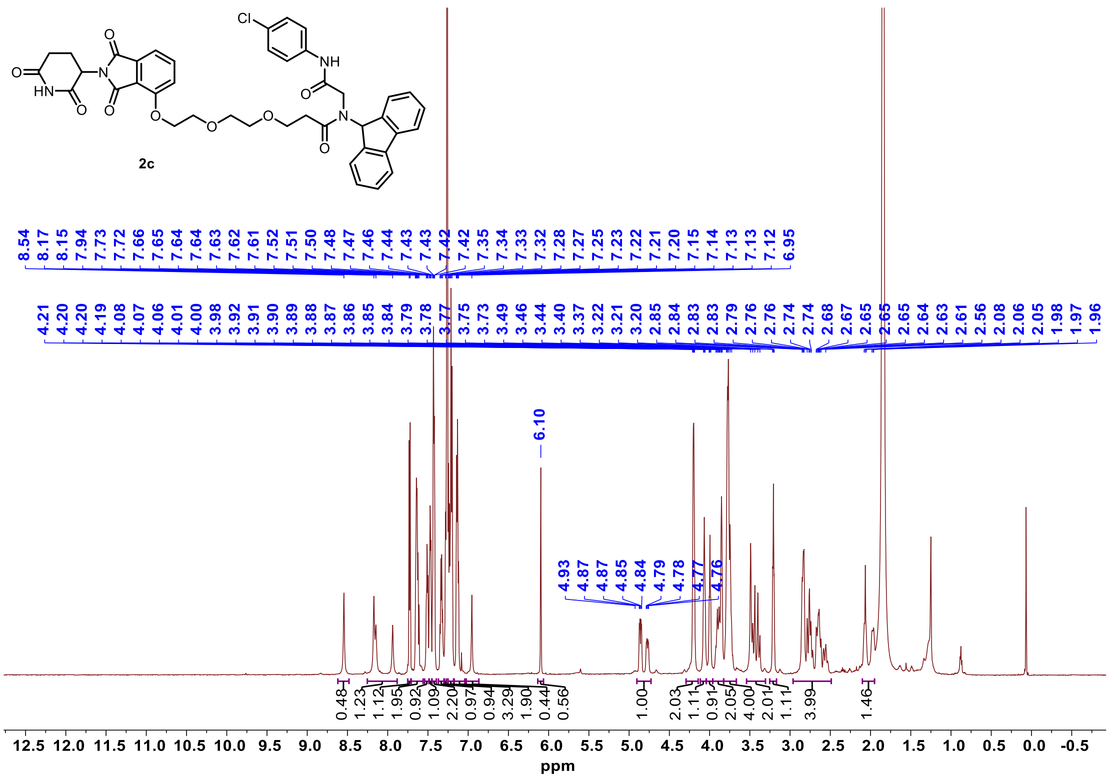

^{13}C NMR **2c**

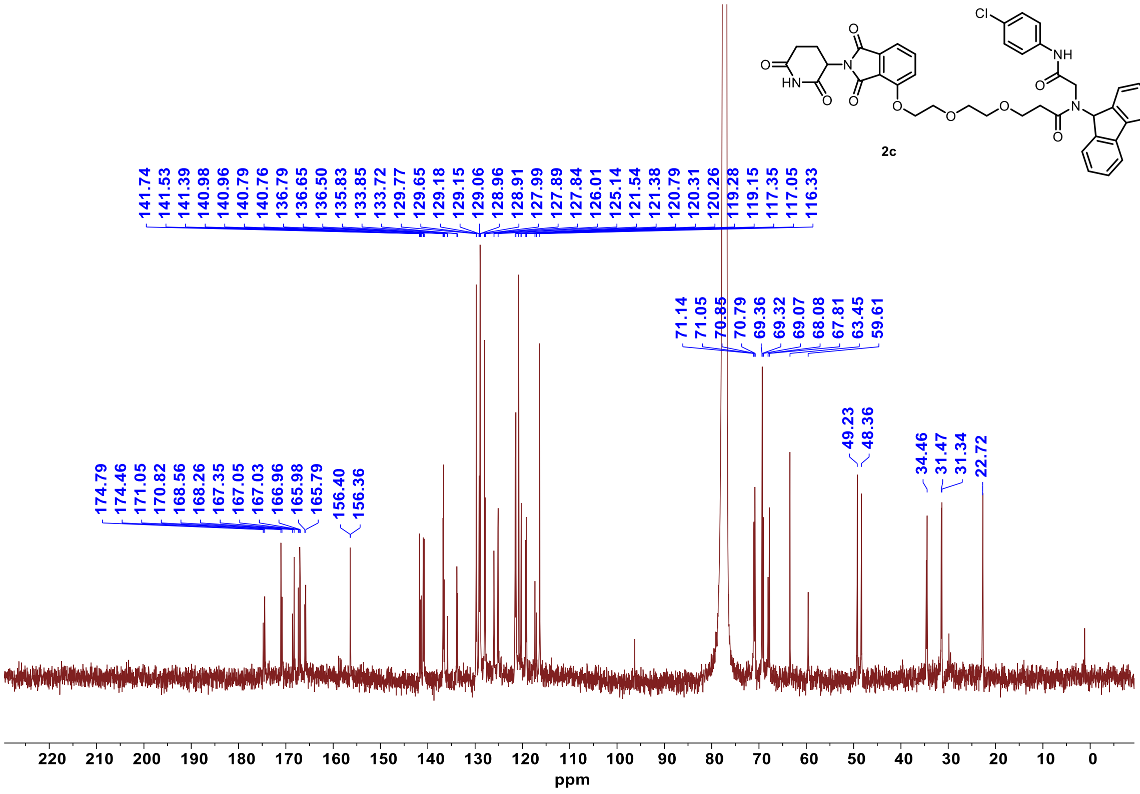

^{1}H NMR **2d**

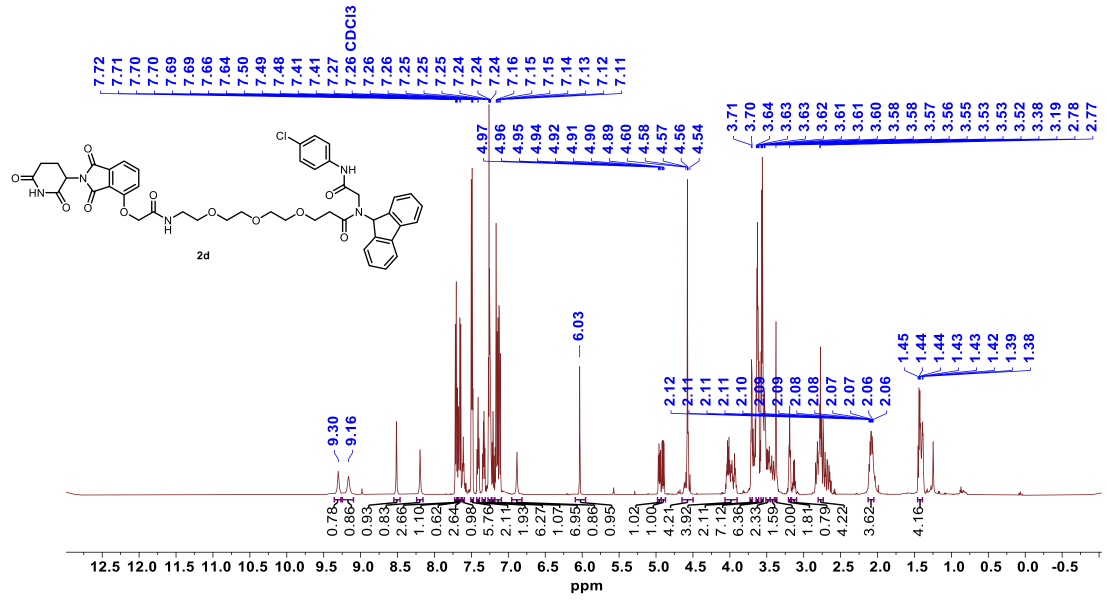

^{13}C NMR **2d**

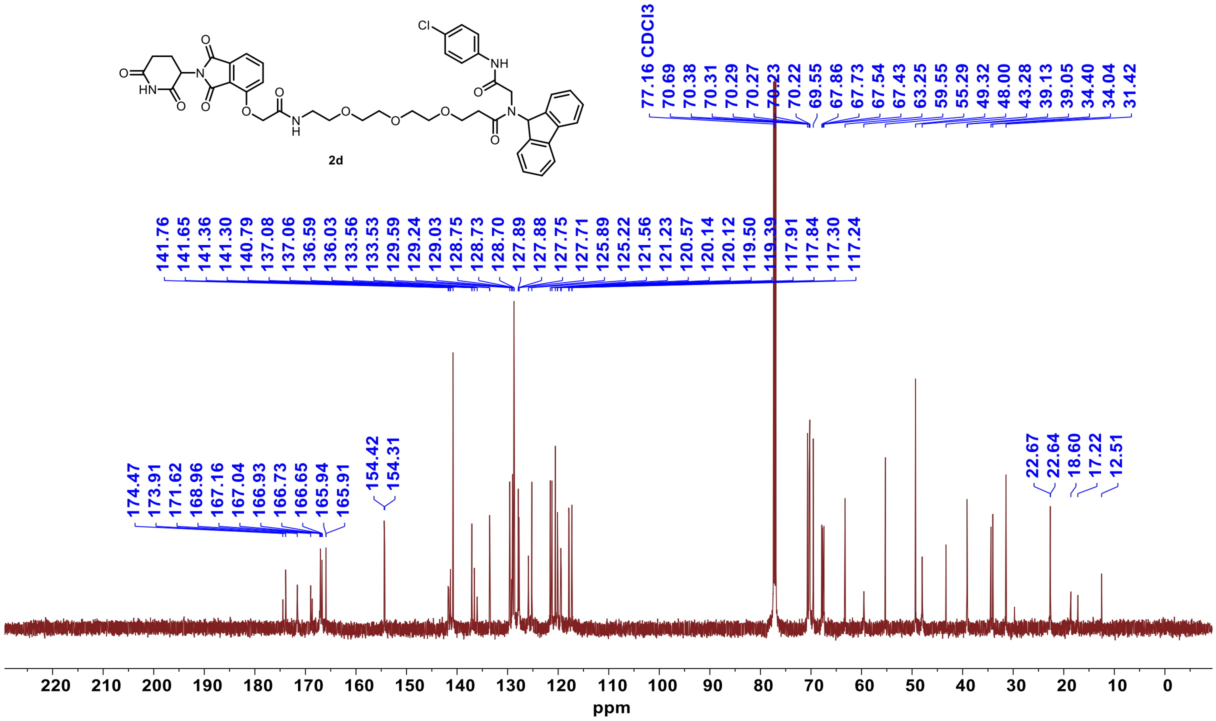

^{1}H NMR **2d-neg**

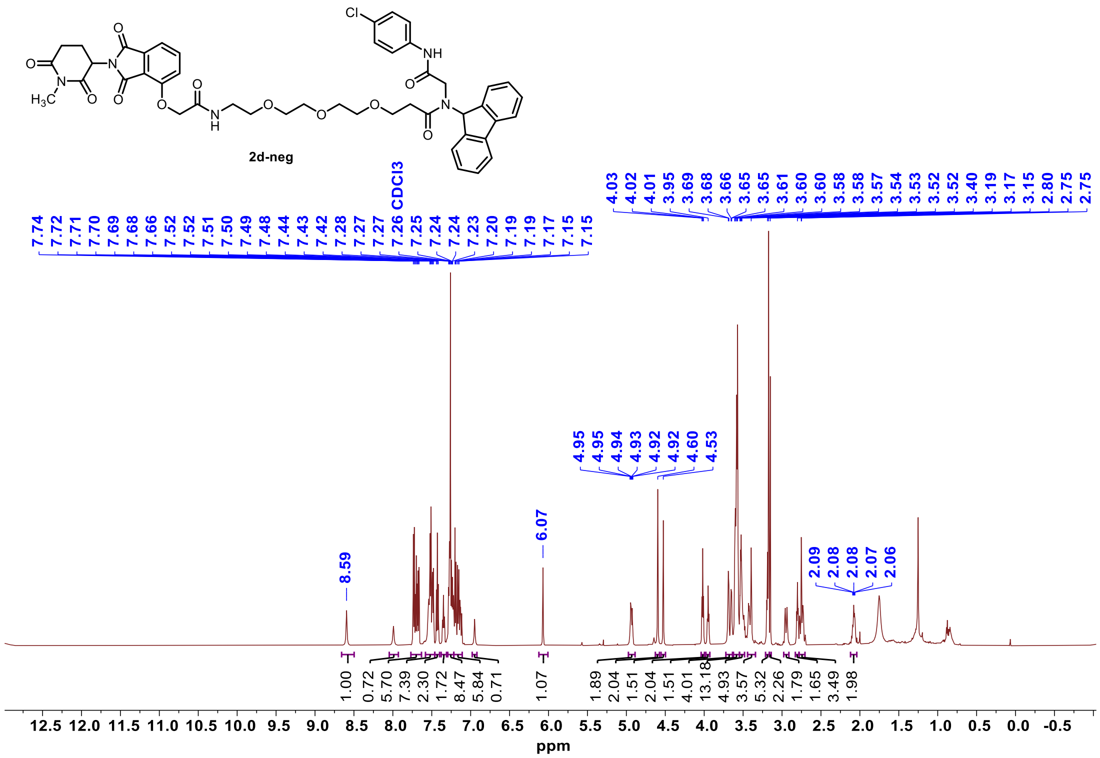

^{13}C NMR **2d-neg**

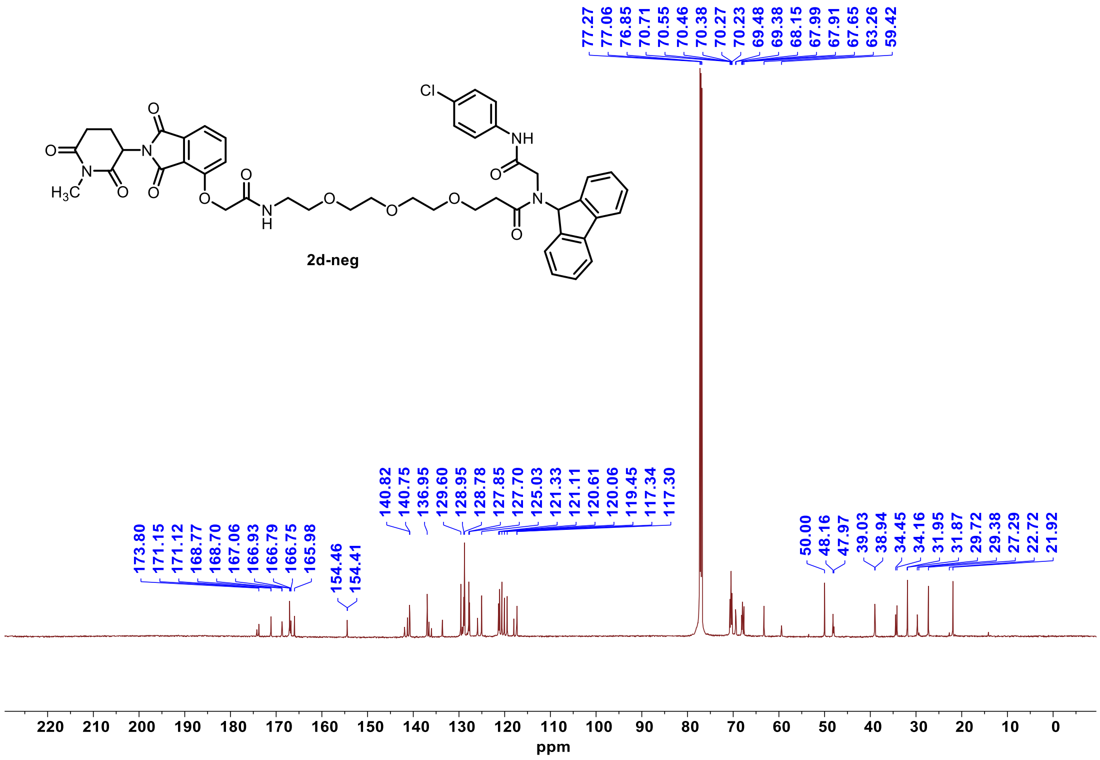

^{1}H NMR **3a**

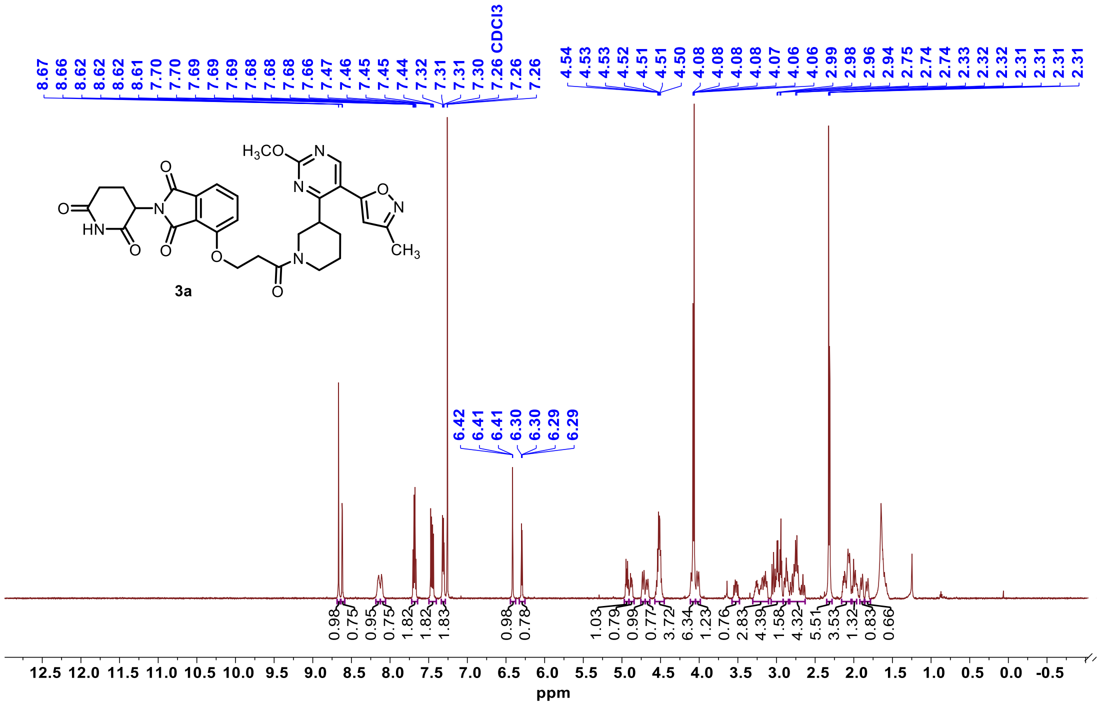

^{13}C NMR **3a**

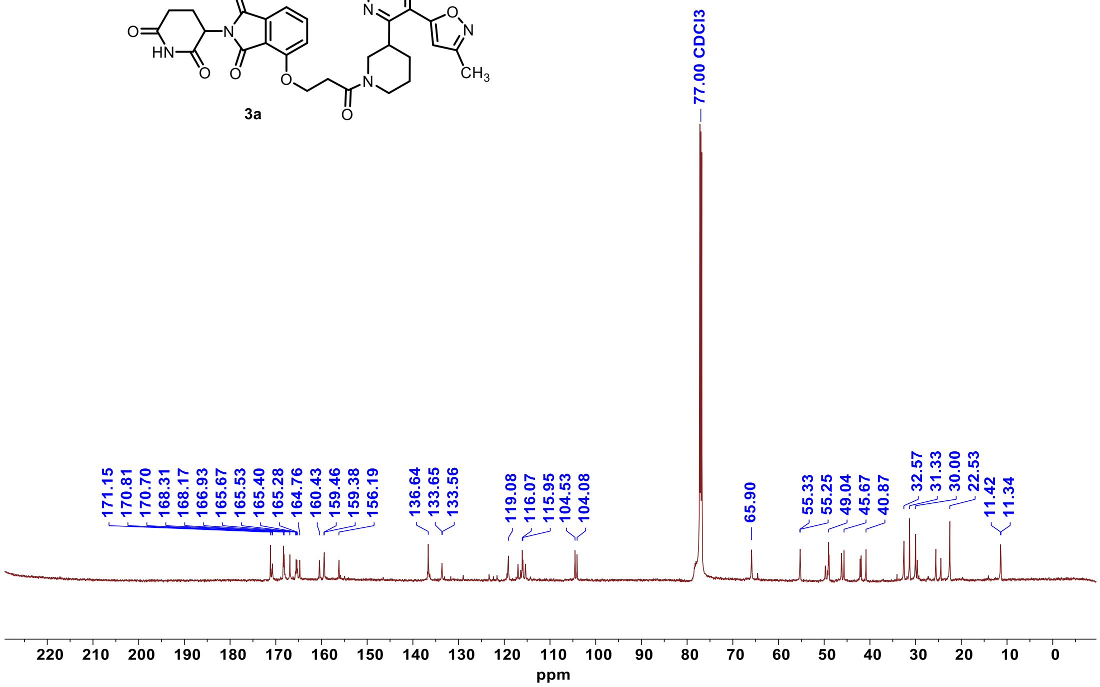

^{1}H NMR **3b**

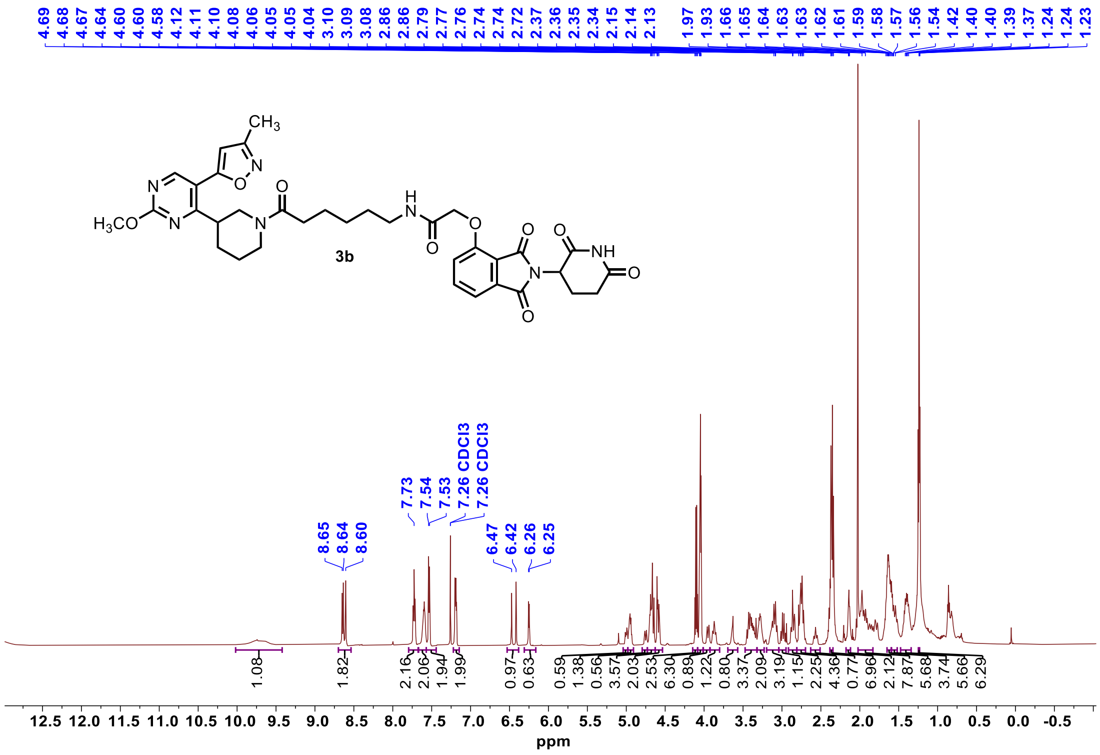

^{13}C NMR **3b**

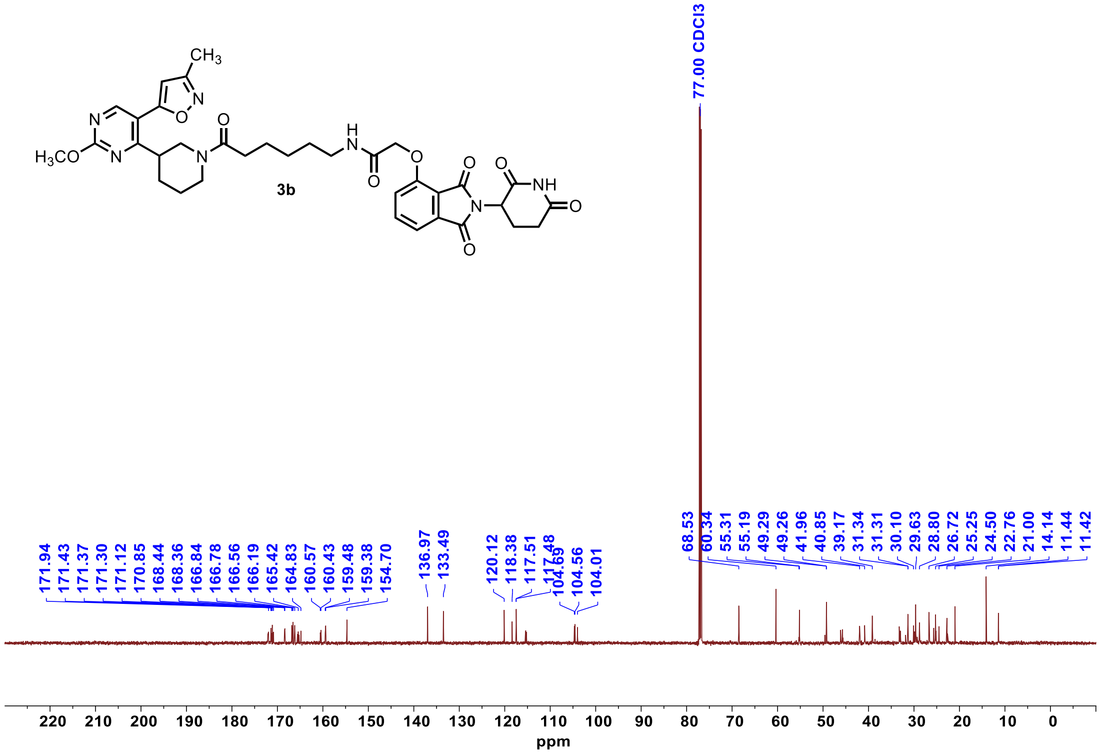

^{1}H NMR **3c**

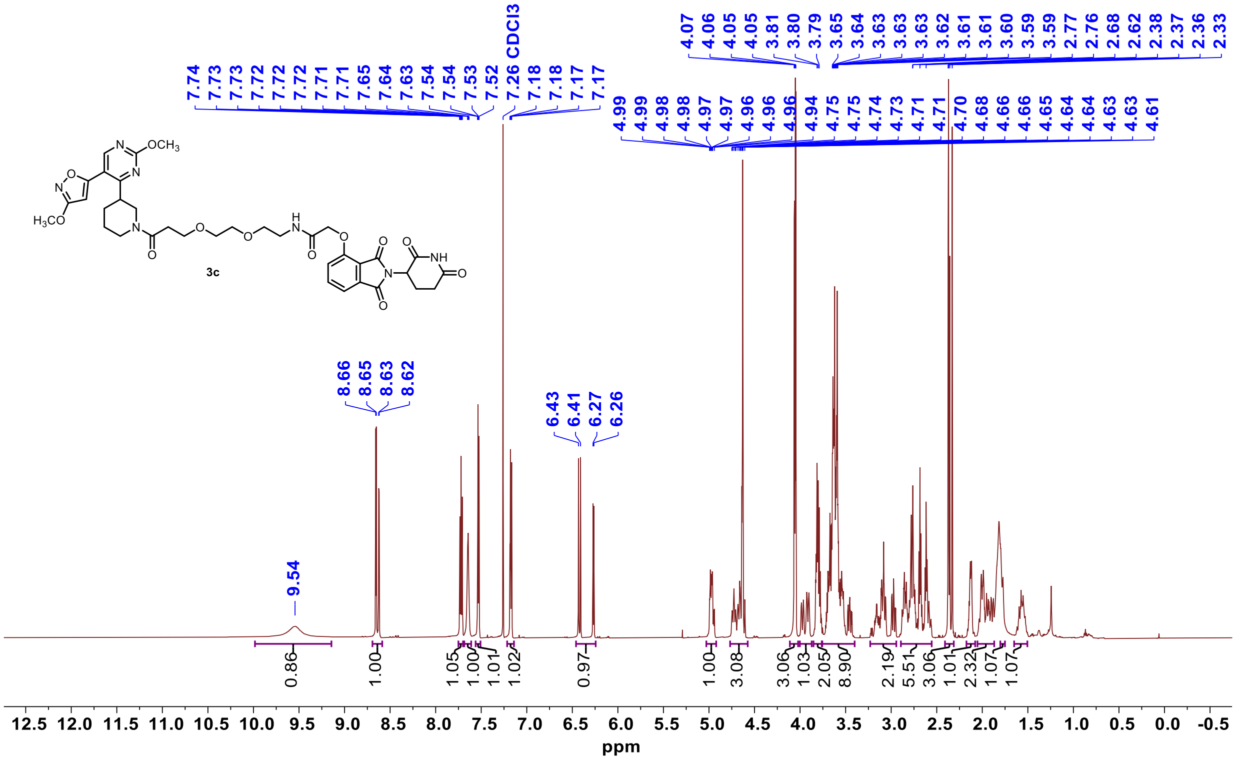

^{13}C NMR **3c**

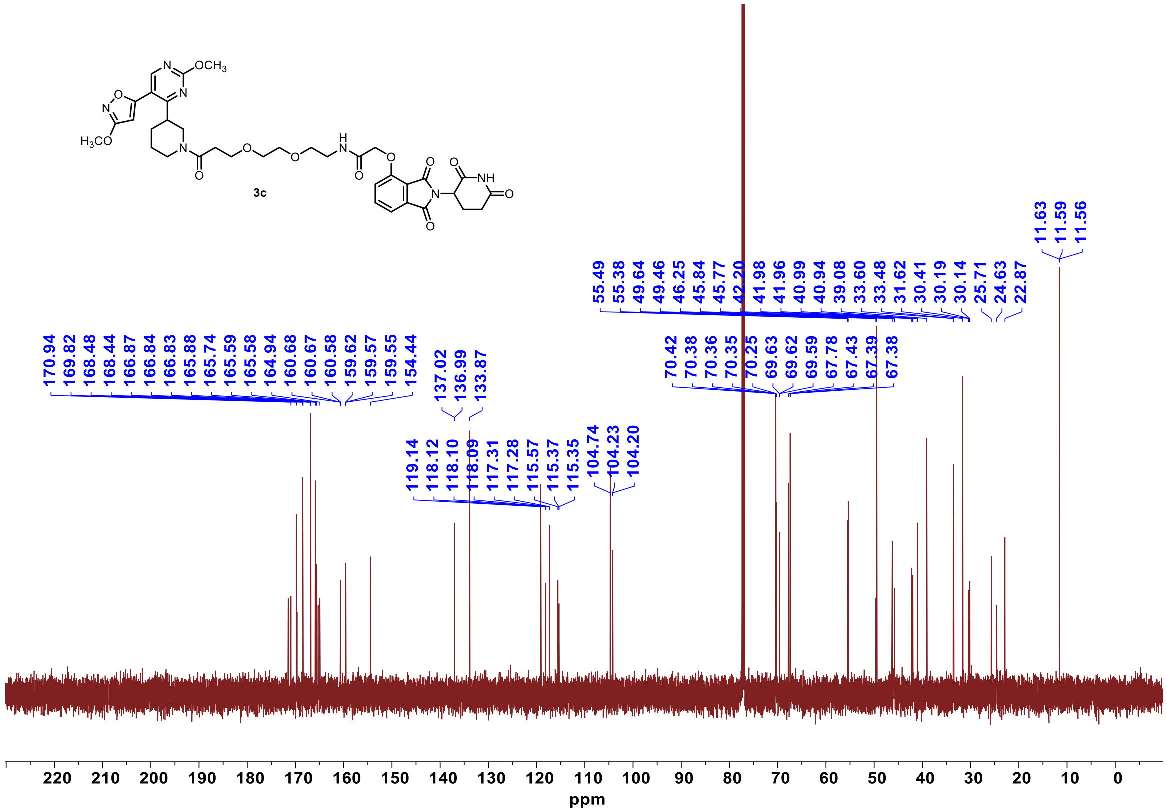

^{1}H NMR **3d**

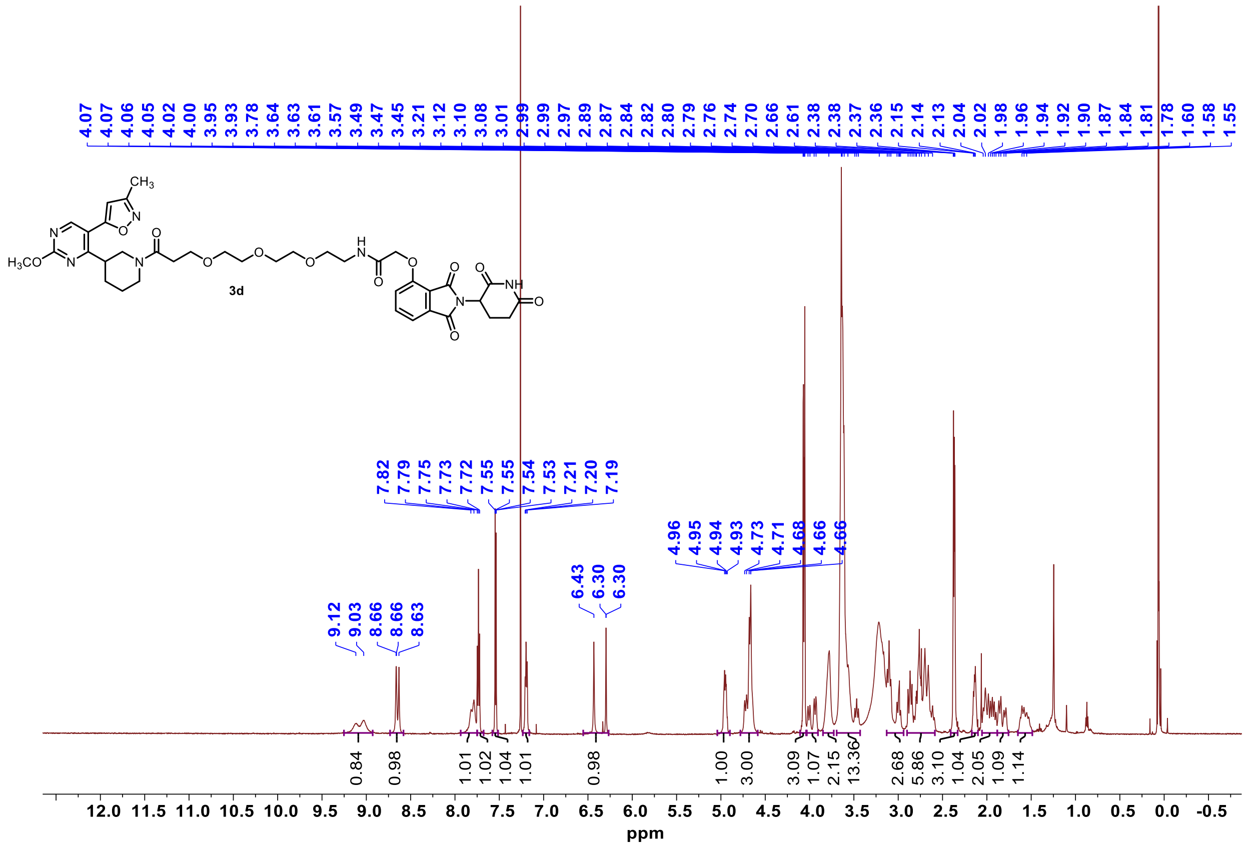

^{13}C NMR **3d**

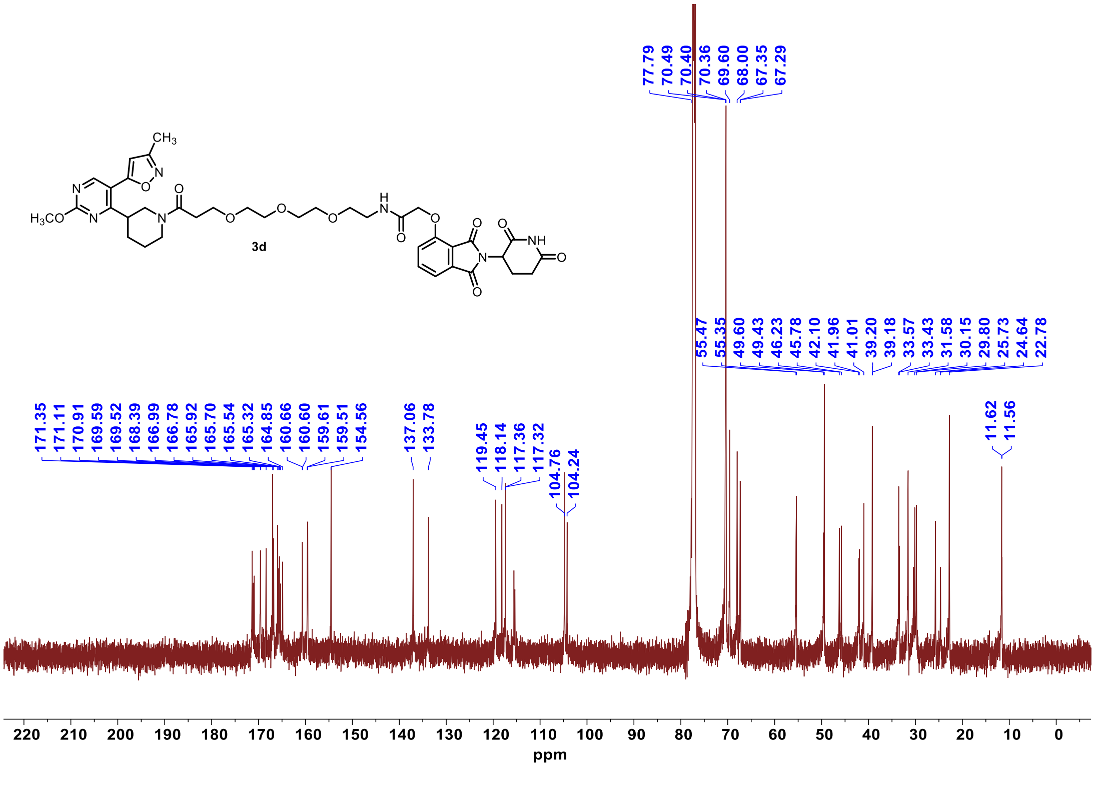

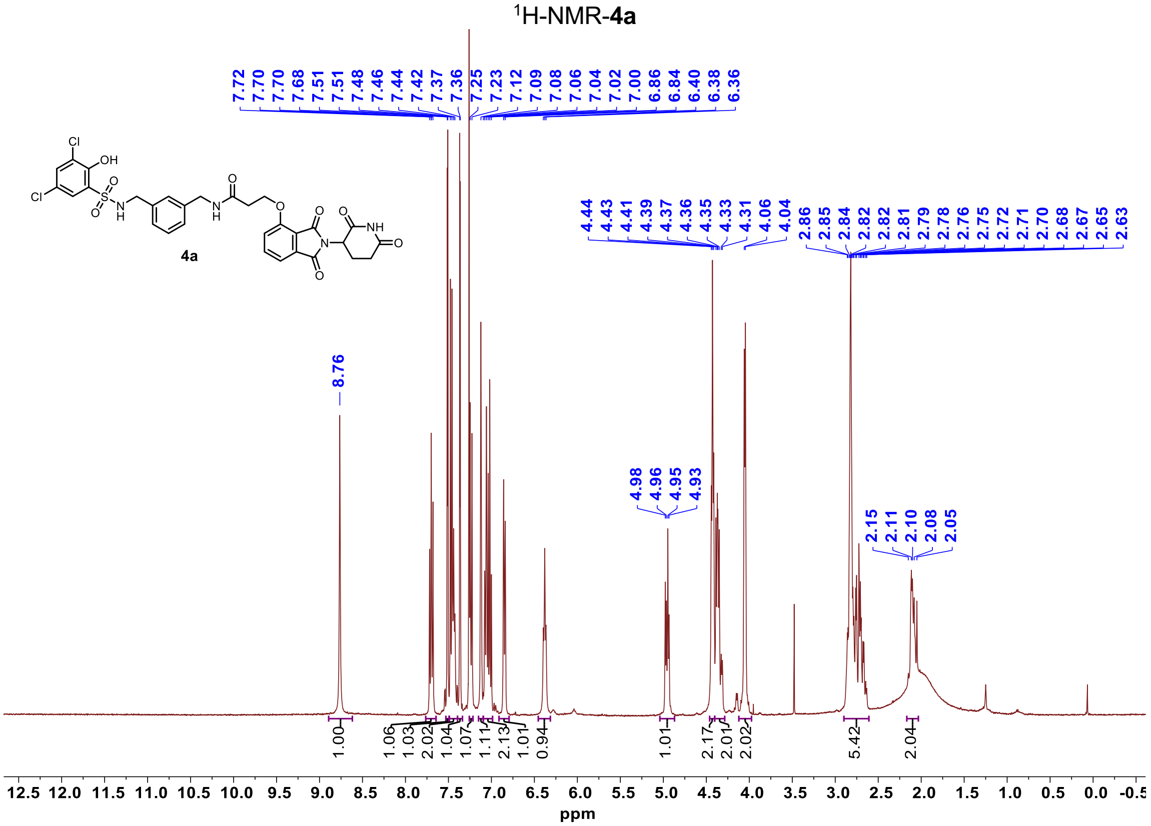

1H-NMR-4a
4a
7.72 7.70 7.70 7.68 7.51 7.51 7.48 7.46 7.44 7.42 7.37 7.36 7.25 7.23 7.12 7.09 7.08 7.06 7.04 7.02 7.00 6.86 6.84 6.40 6.38 6.36
8.76
4.44 4.43 4.41 4.39 4.37 4.36 4.35 4.33 4.31 4.06 4.04
4.98 4.96 4.95 4.93
2.86 2.85 2.84 2.82 2.82 2.81 2.79 2.78 2.76 2.75 2.72 2.71 2.70 2.68 2.67 2.65 2.63
2.15 2.11 2.10 2.08 2.05
1.00 1.06 1.03 2.02 1.04 1.07 1.11 2.13 1.01 0.94 1.01 2.17 2.01 2.02 5.42 2.04
ppm

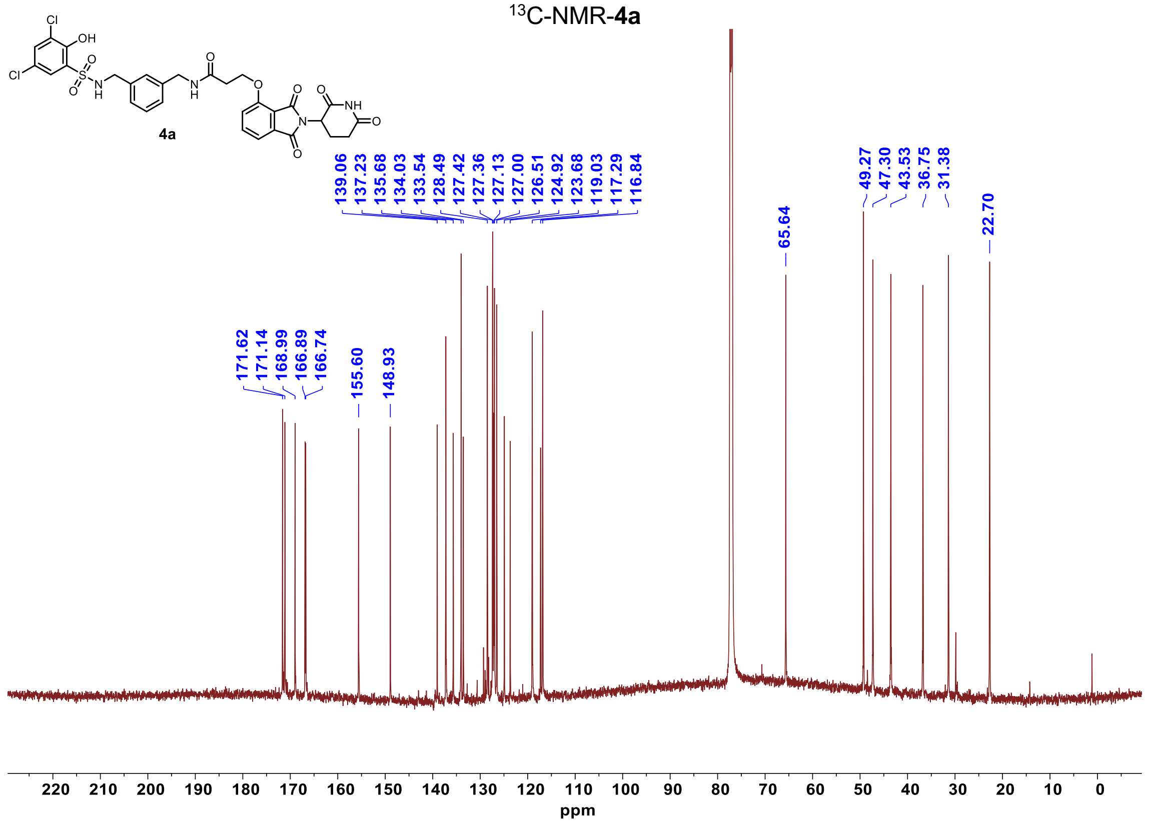

13C-NMR-4a
4a
171.62 171.14 168.99 166.89 166.74 155.60 148.93
139.06 137.23 135.68 134.03 133.54 128.49 127.42 127.36 127.13 127.00 126.51 124.92 123.68 119.03 117.29 116.84
65.64
49.27 47.30 43.53 36.75 31.38 22.70
ppm

^{1}H-NMR-**4b**

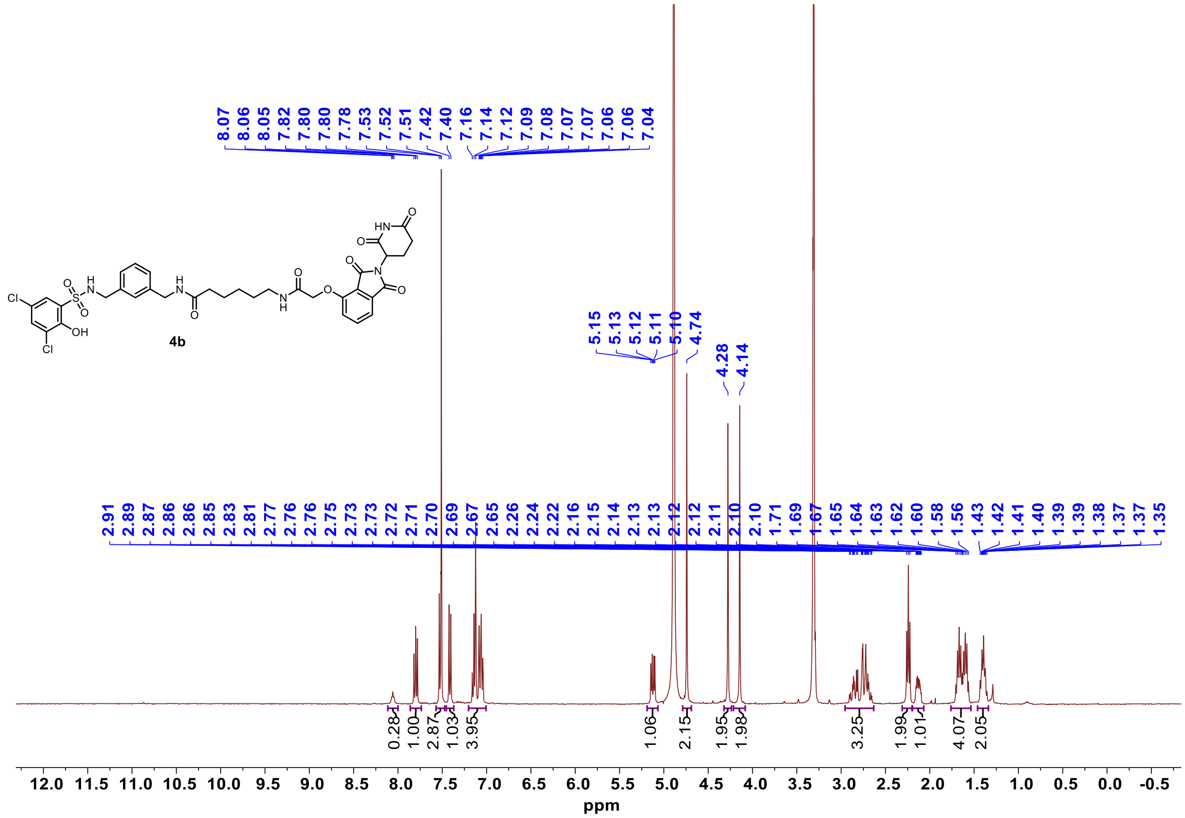

^{13}C-NMR-**4b**

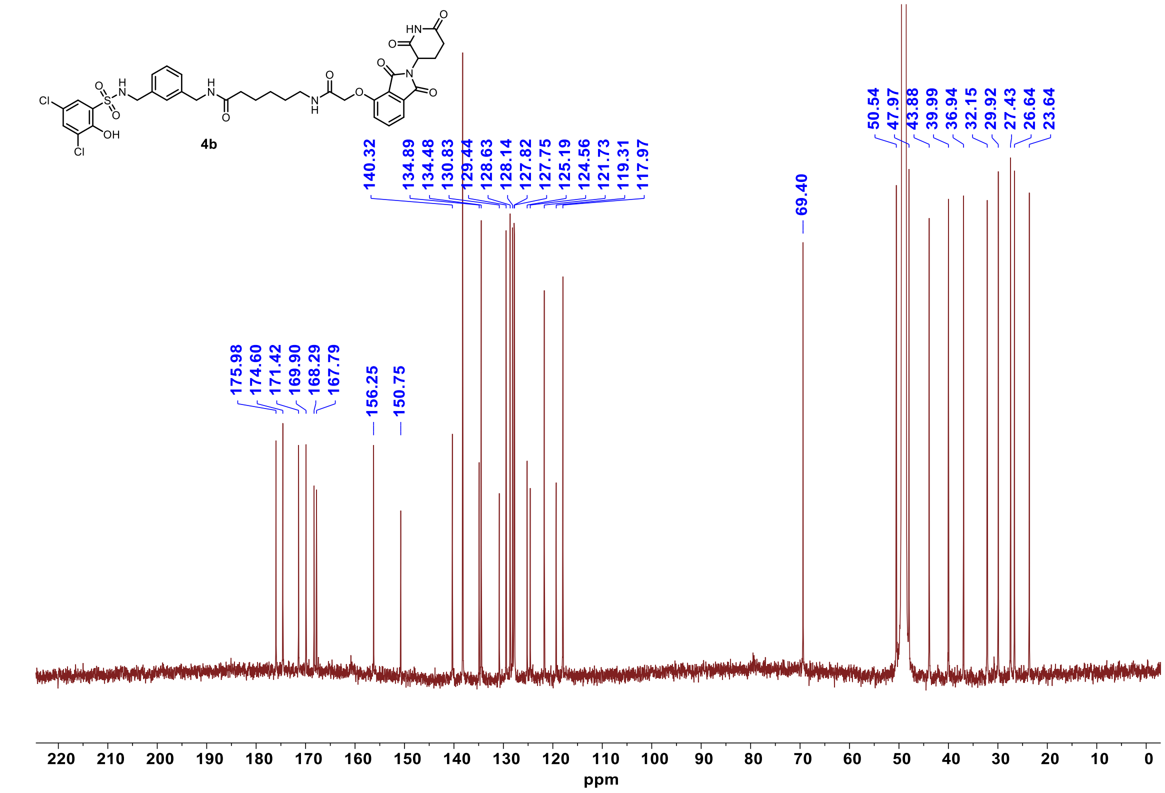

^{1}H NMR **4c**

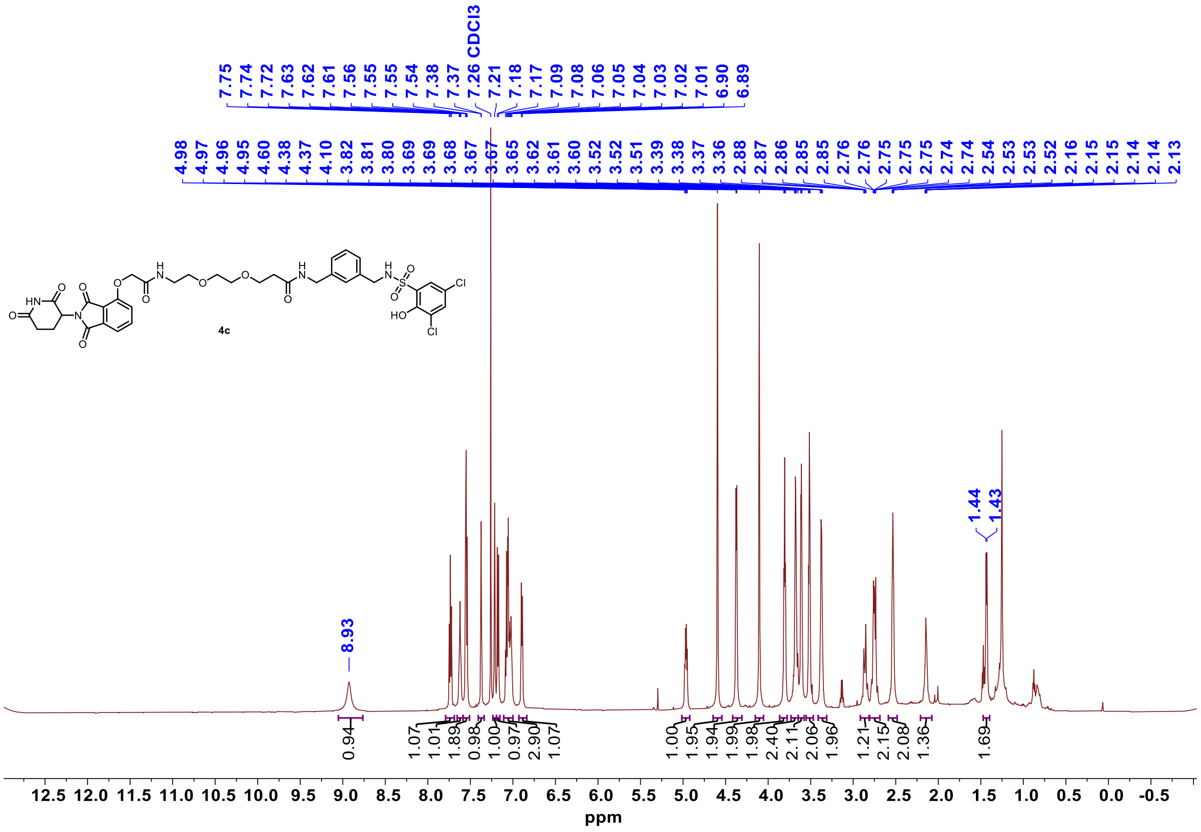

^{13}C NMR **4c**

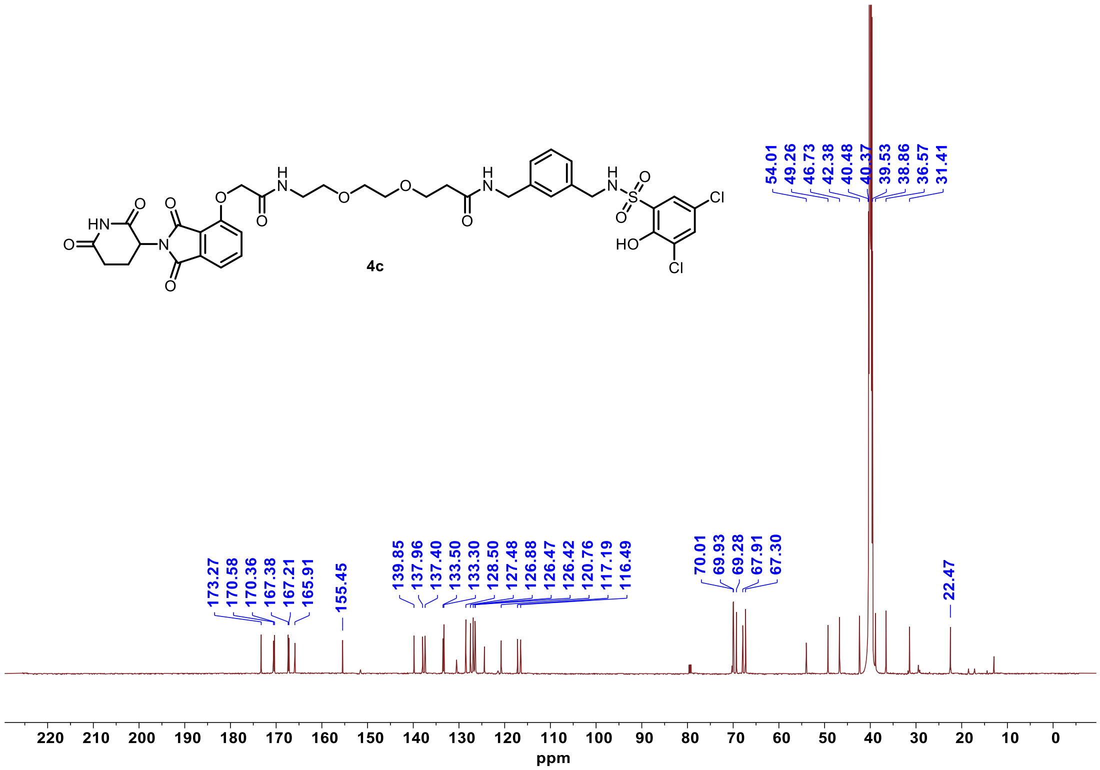

^{1}H-NMR-**4d**

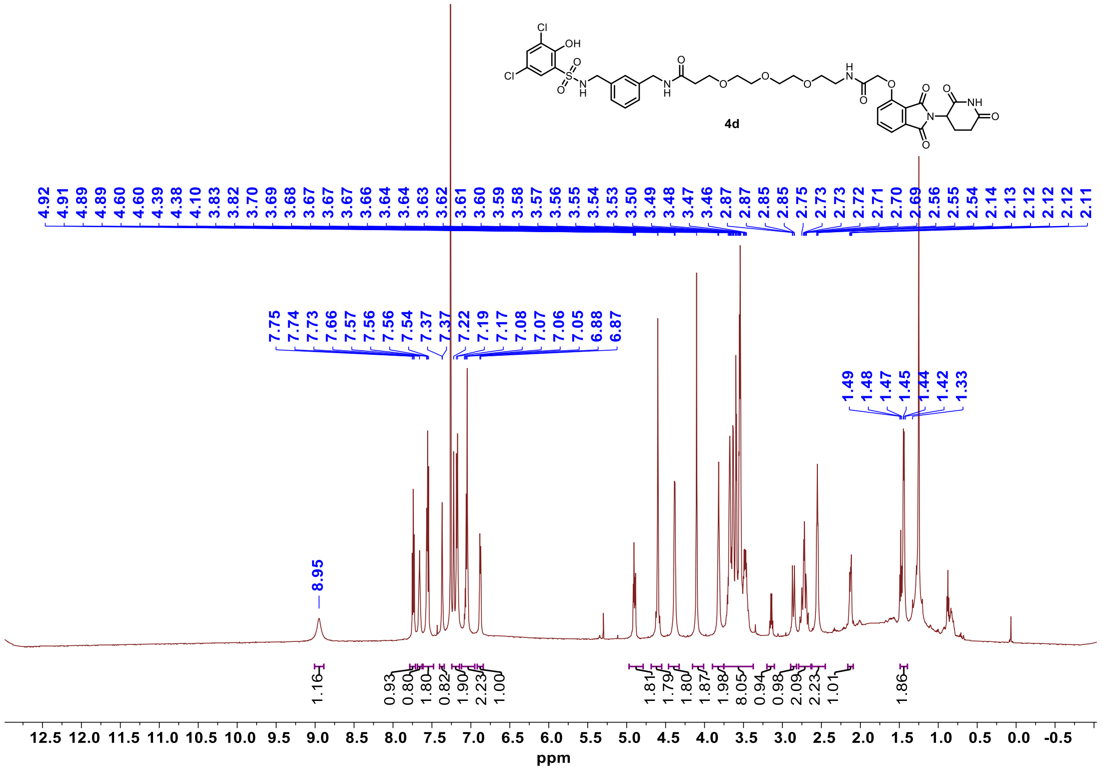

^{13}C-NMR-**4d**

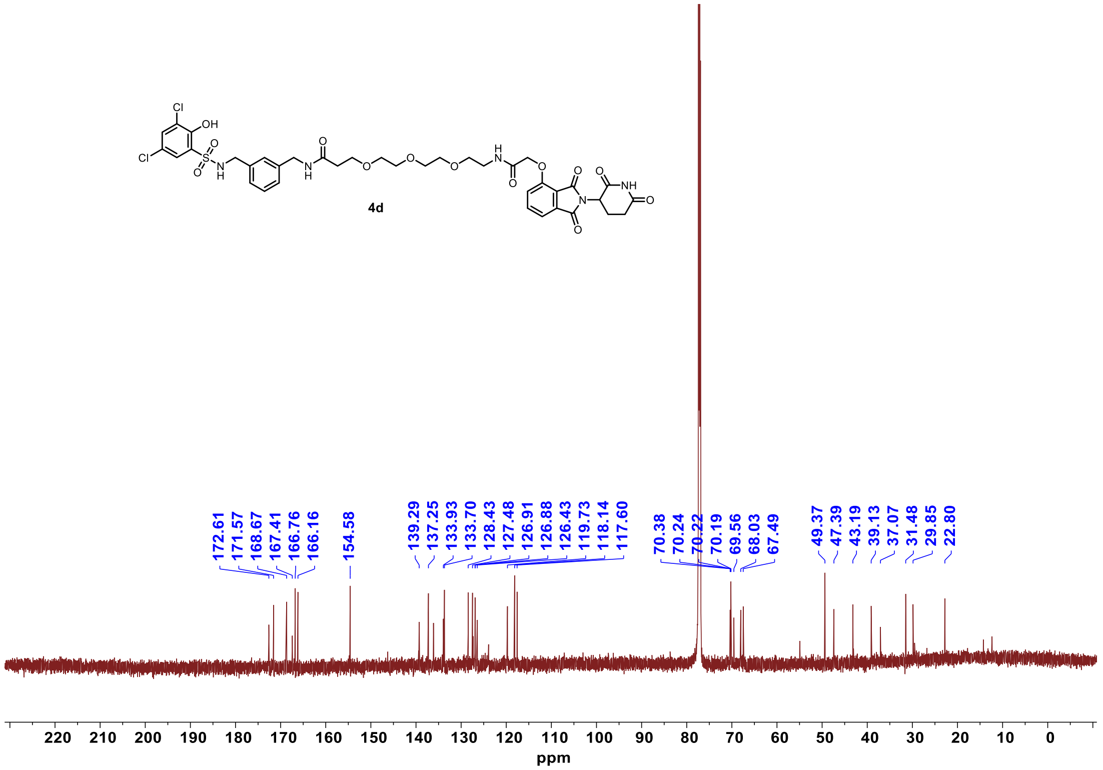

^{1}H NMR **5a**

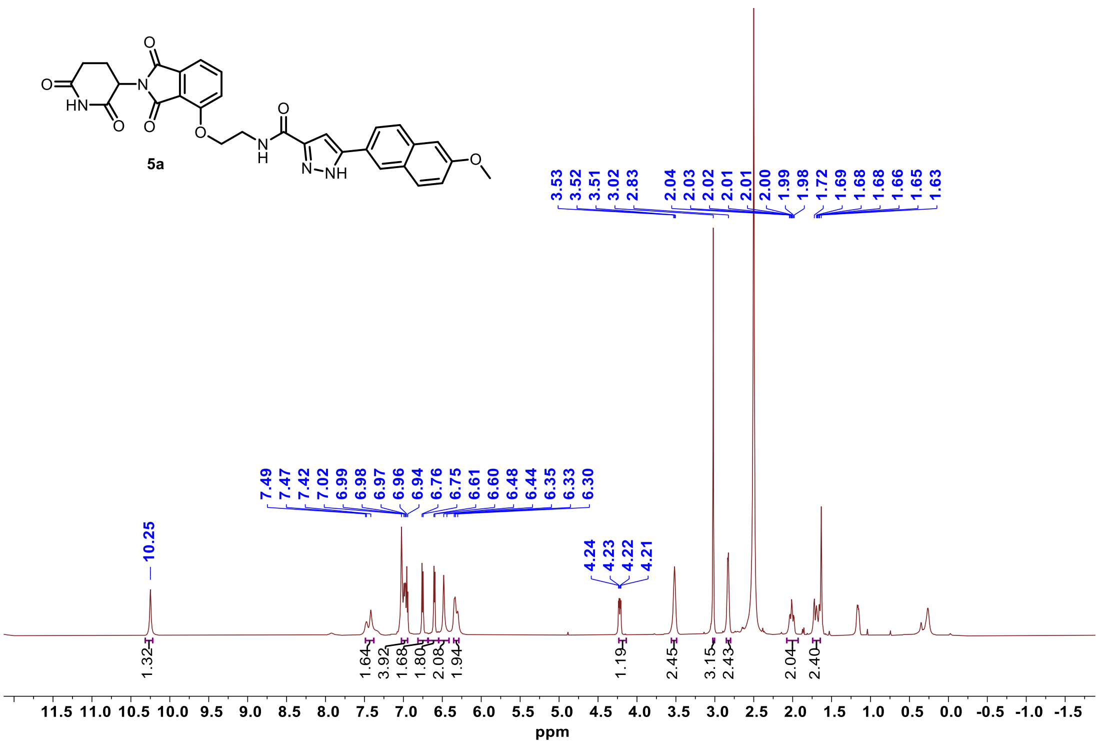

^{13}C NMR **5a**

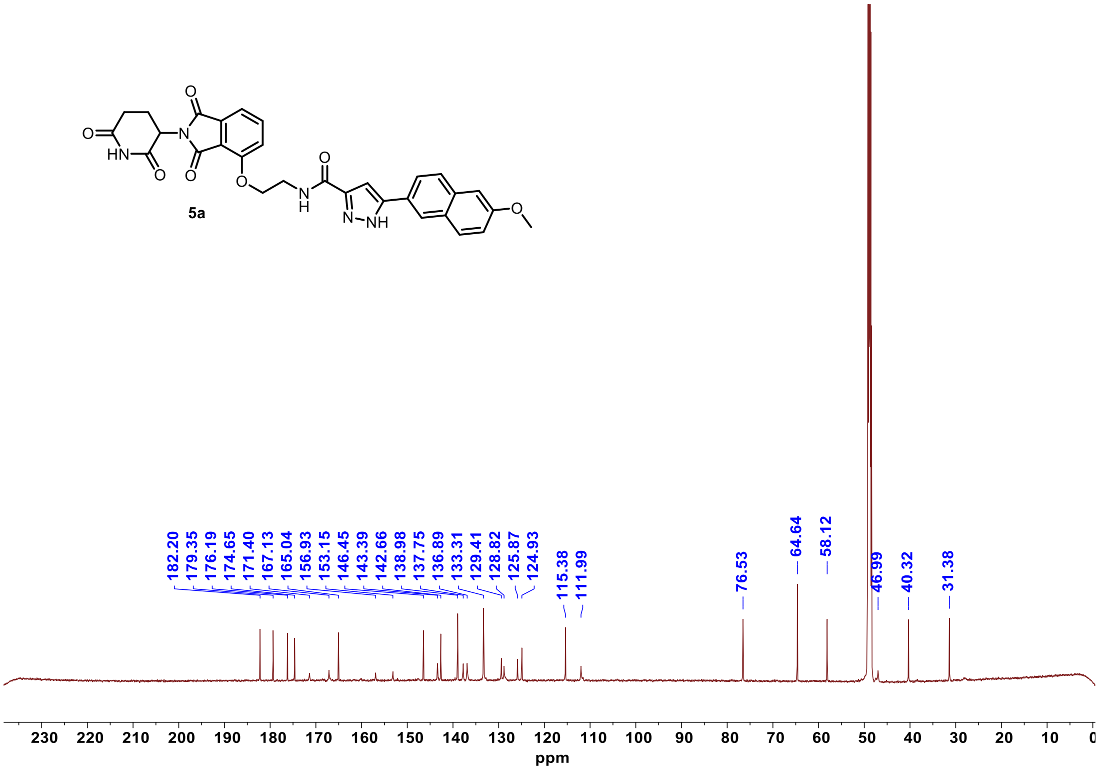

^{1}H NMR **5a-neg**

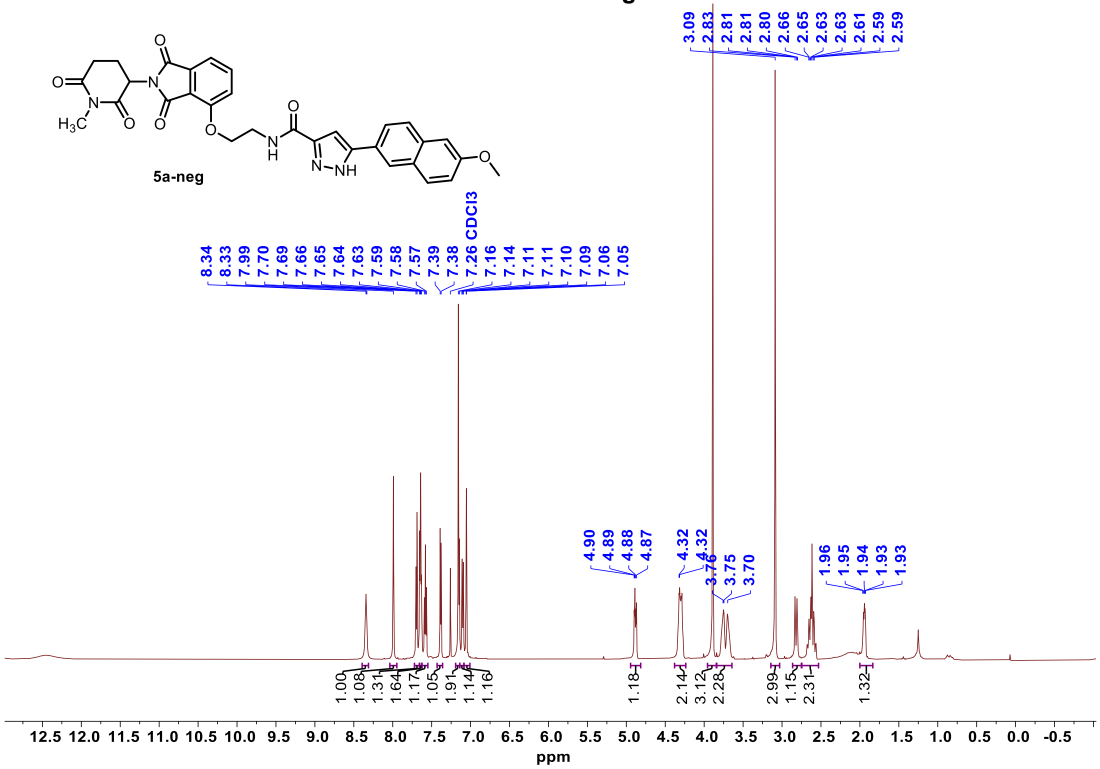

^{13}C NMR **5a-neg**

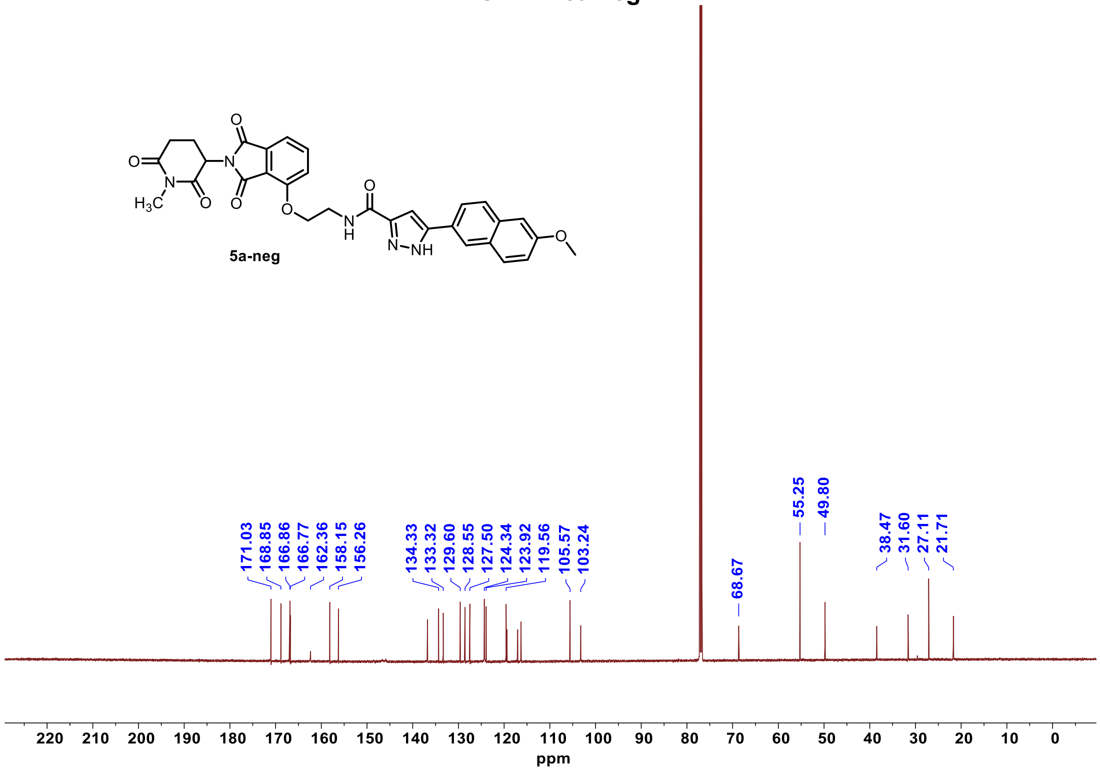

^{1}H-NMR-**5b**

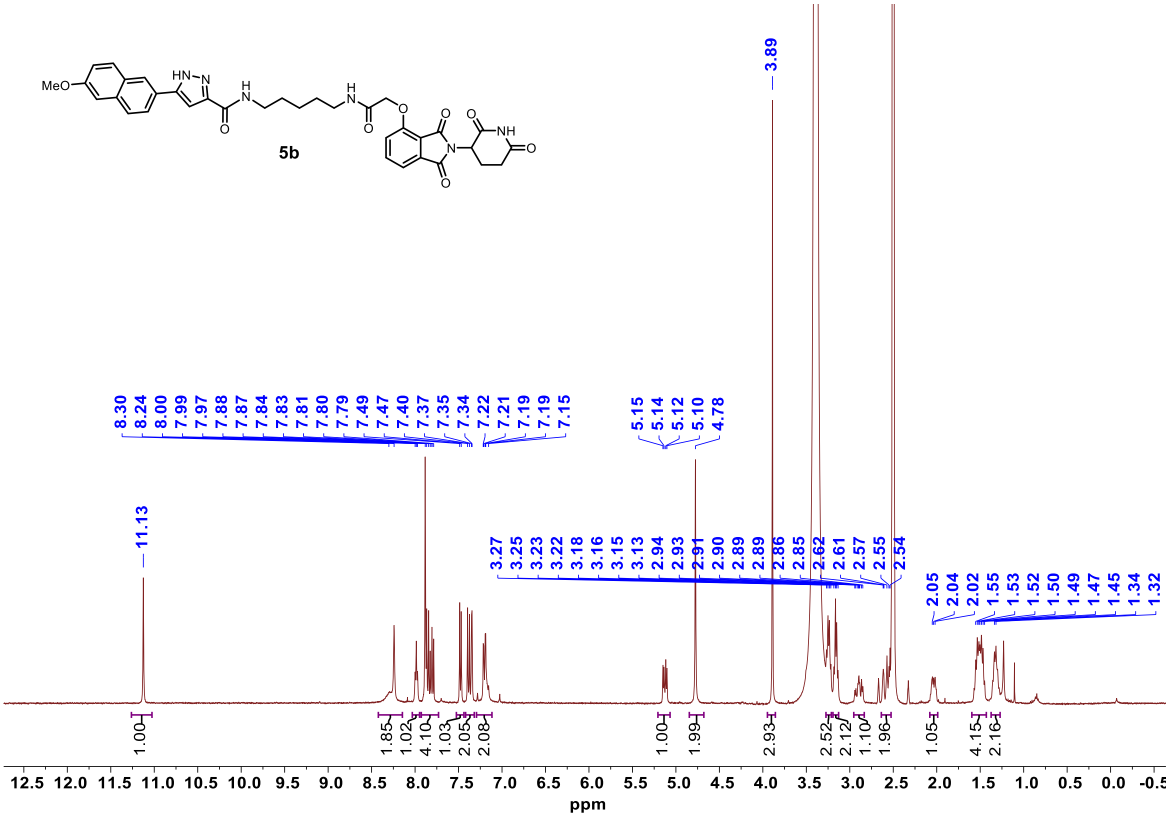

^{13}C-NMR-**5b**

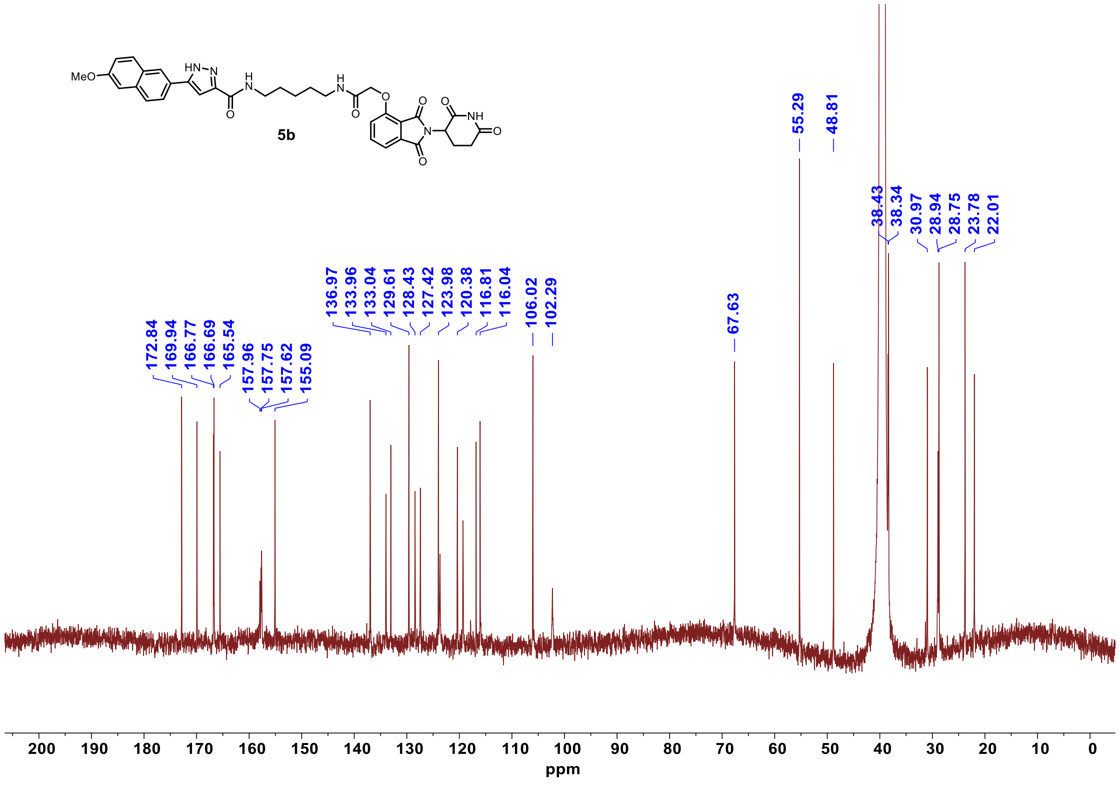

^{1}H-NMR-**5c**

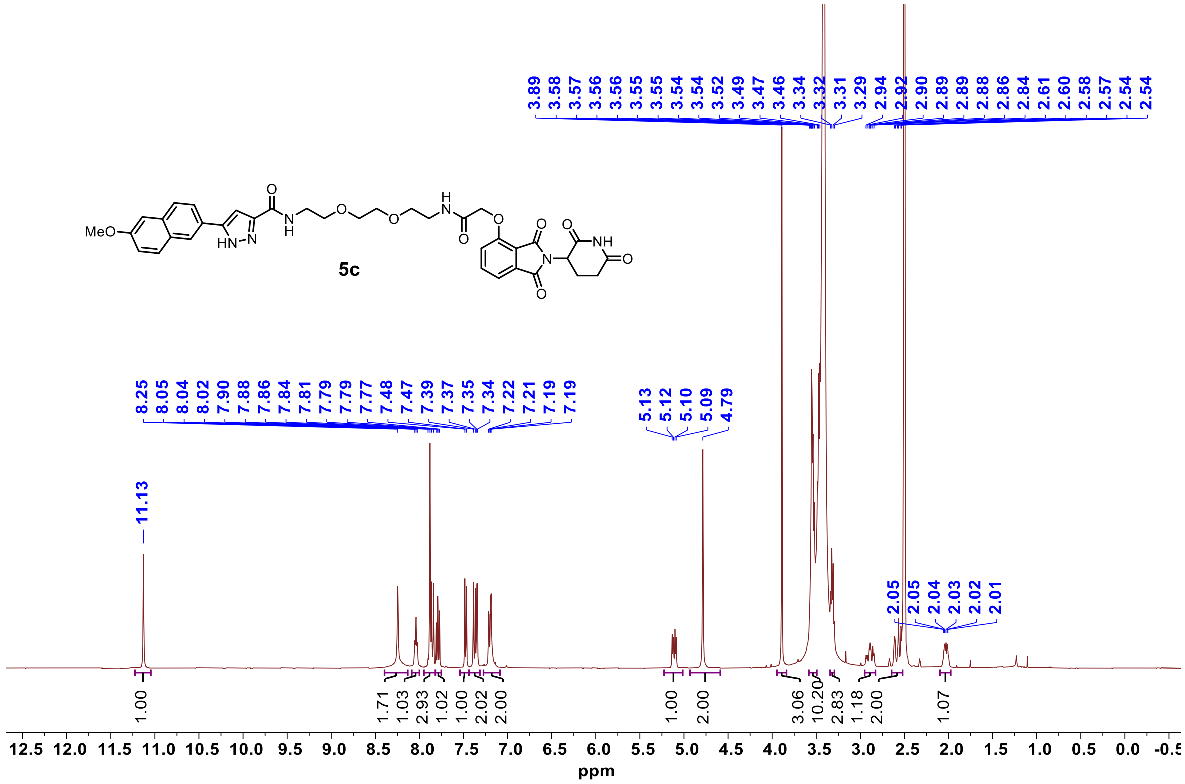

^{13}C-NMR-**5c**

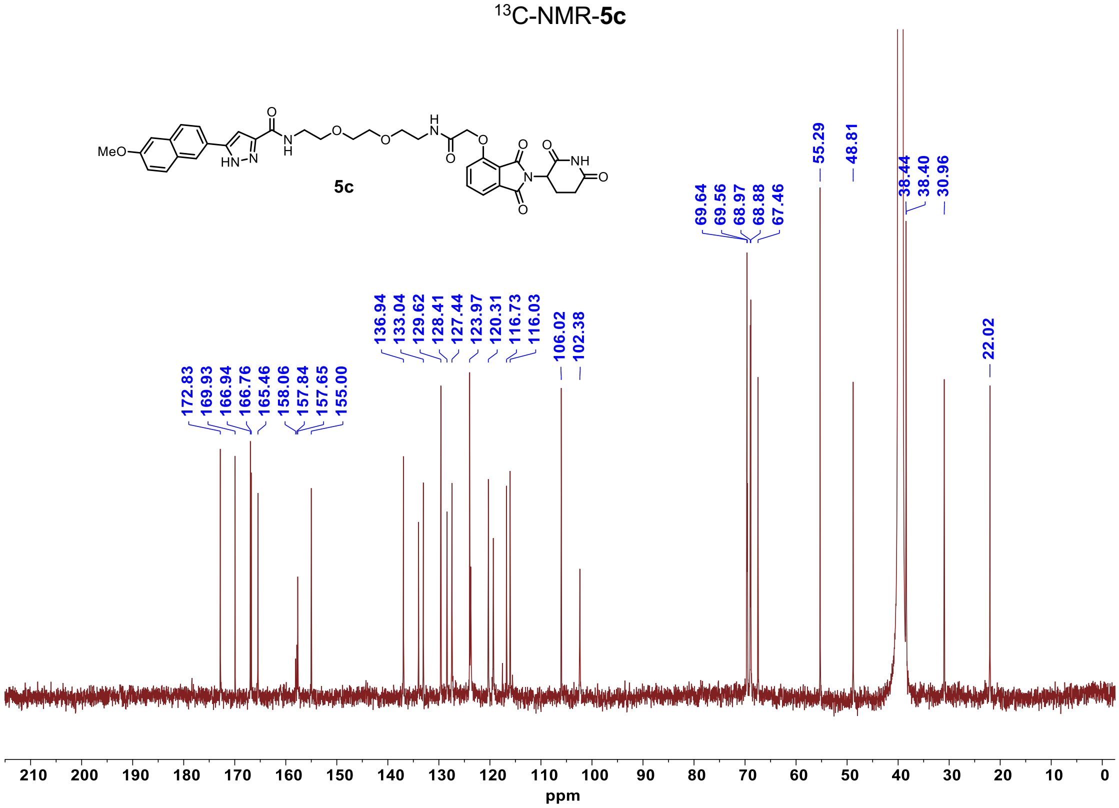

^{1}H-NMR-**5d**

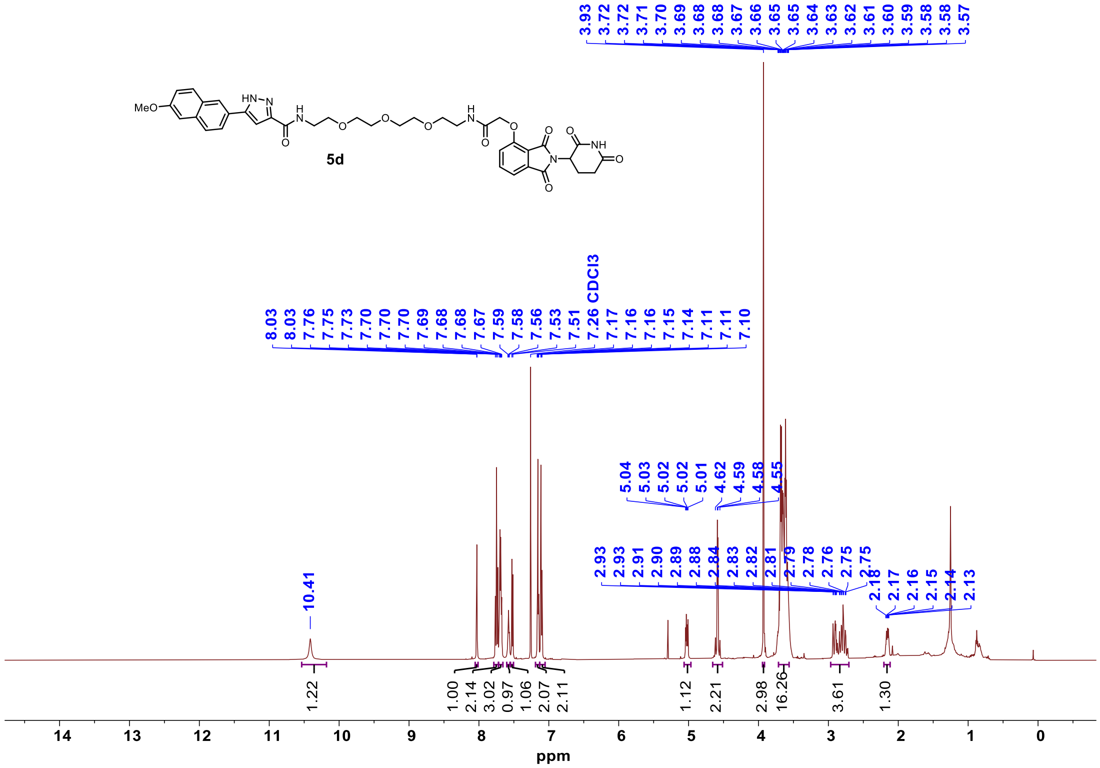

^{13}C-NMR-**5d**

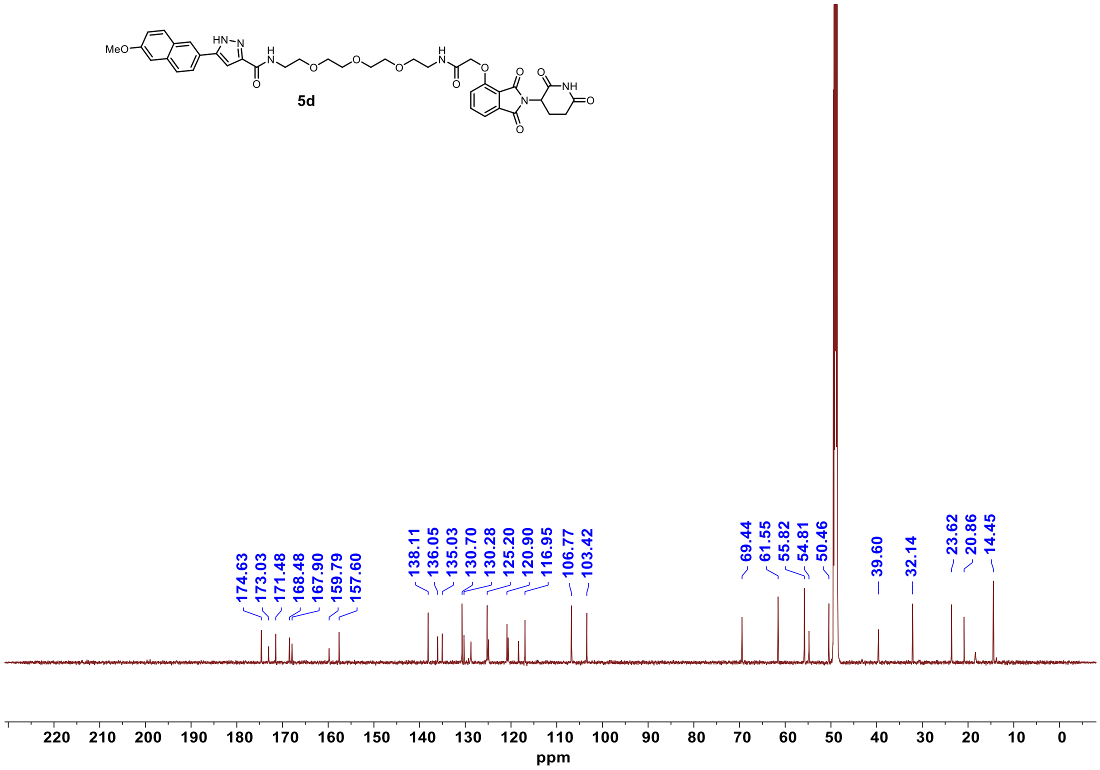

^{1}H NMR **6a**

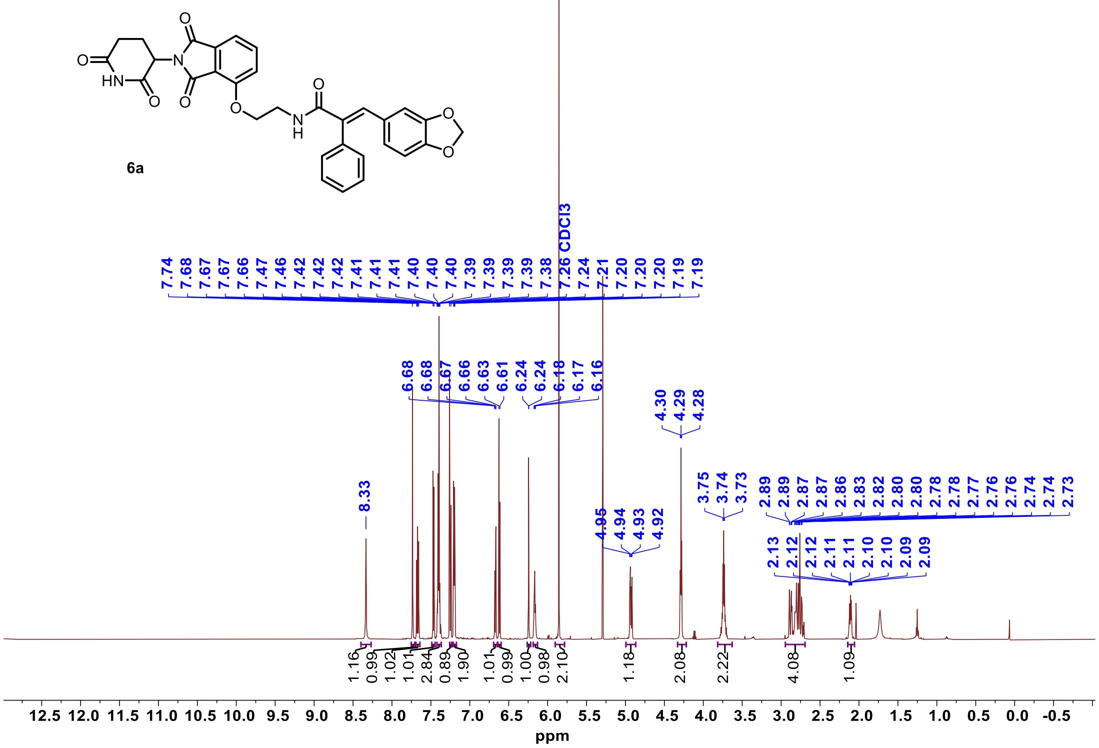

^{13}C NMR **6a**

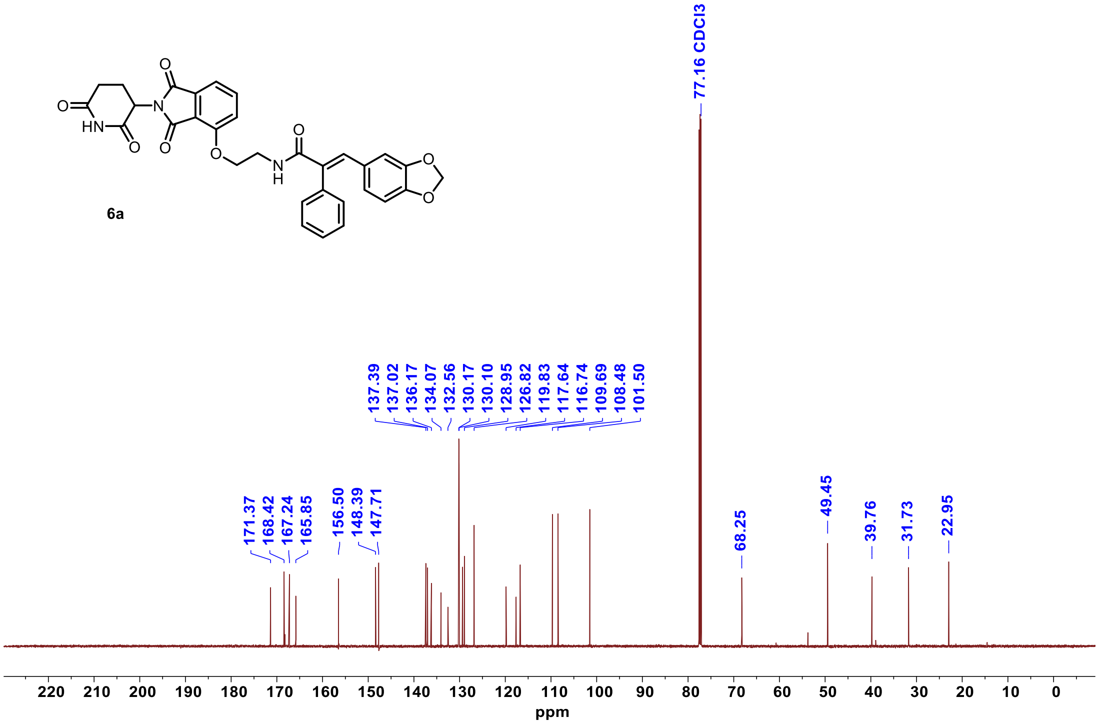

^{1}H NMR **6b**

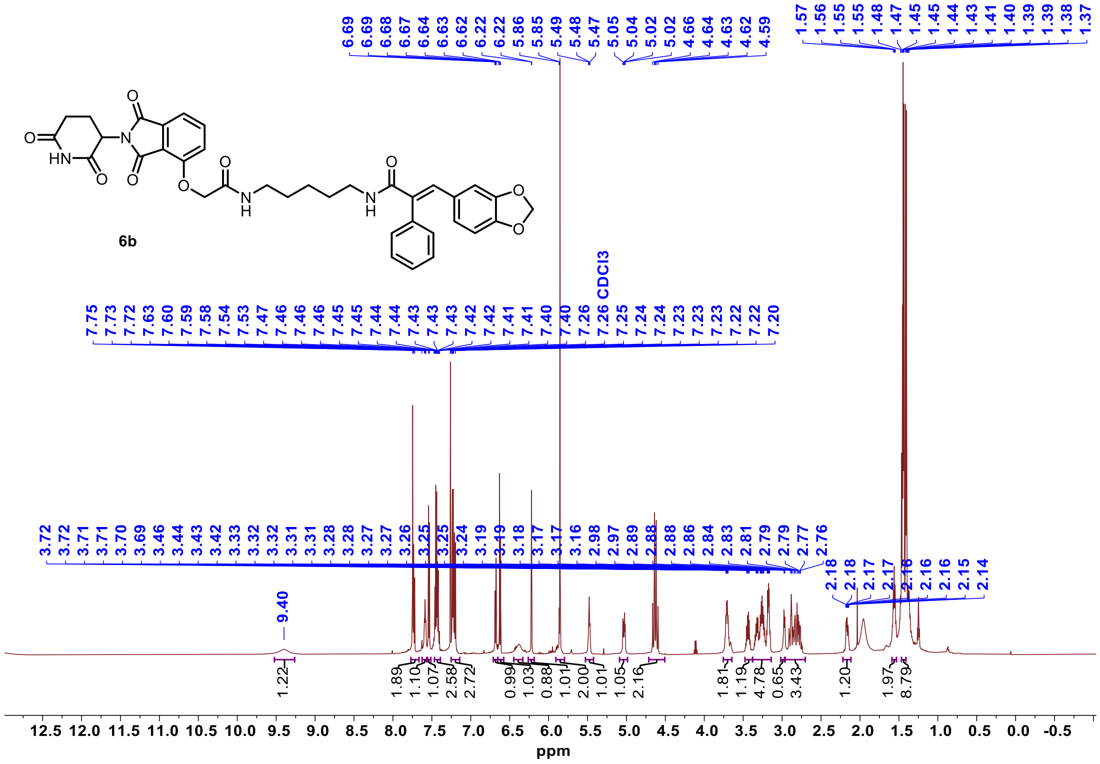

^{13}C NMR **6b**

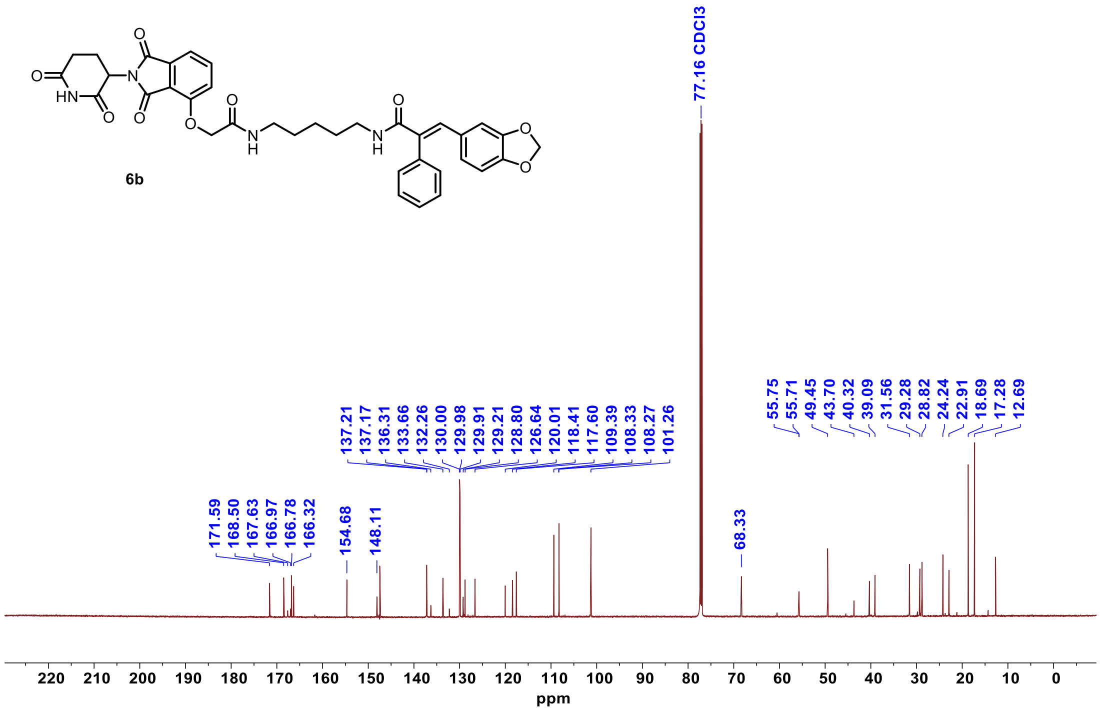

^{1}H NMR **6c**

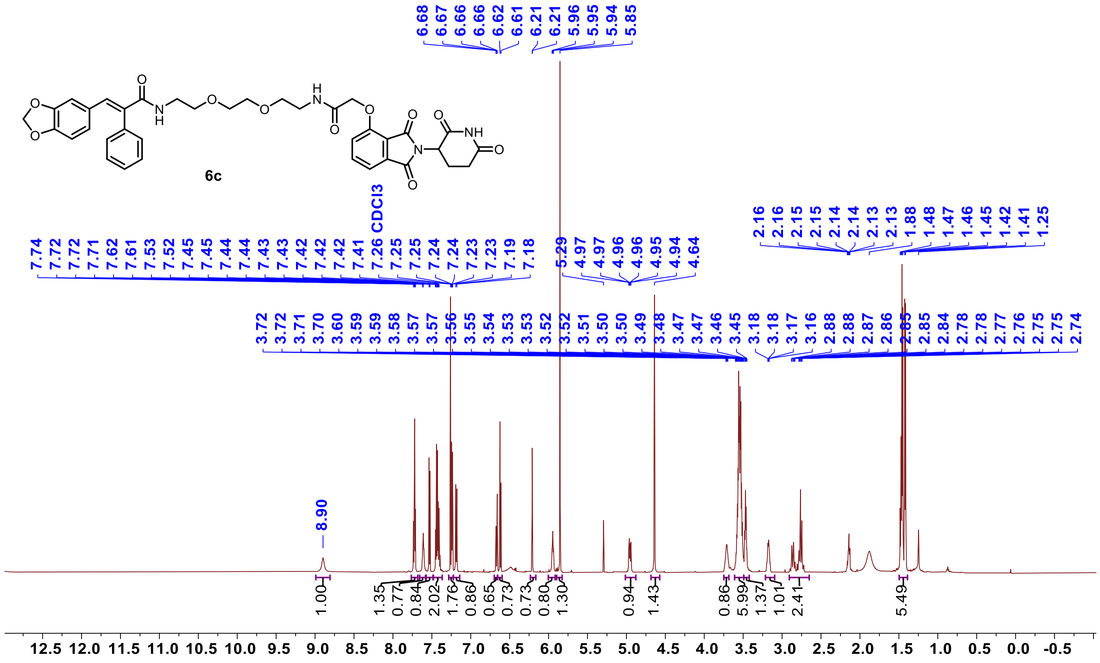

^{13}C NMR **6c**

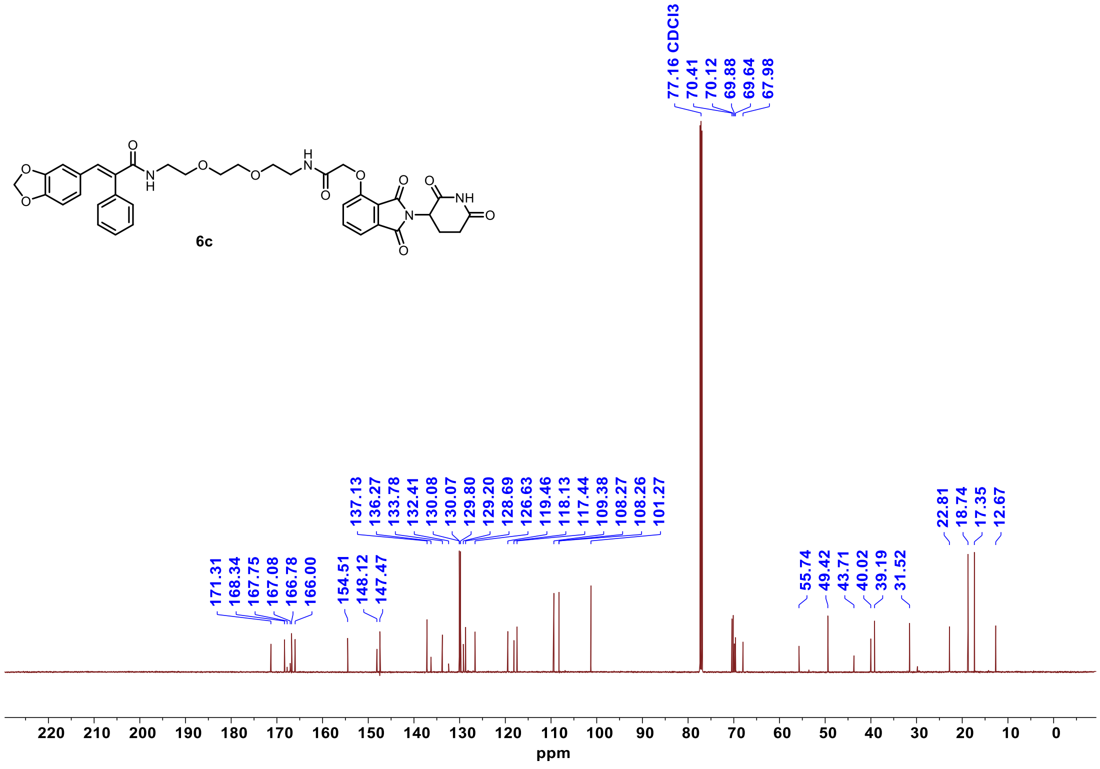

^{1}H NMR **6d**

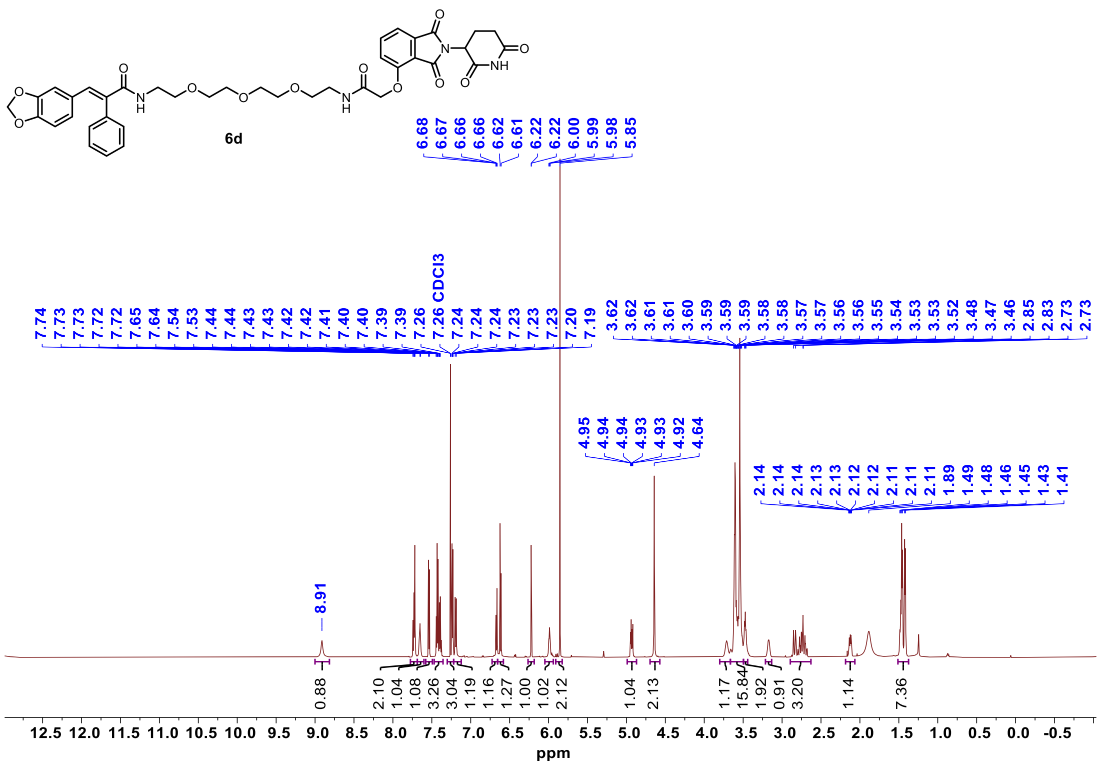

^{13}C NMR **6d**

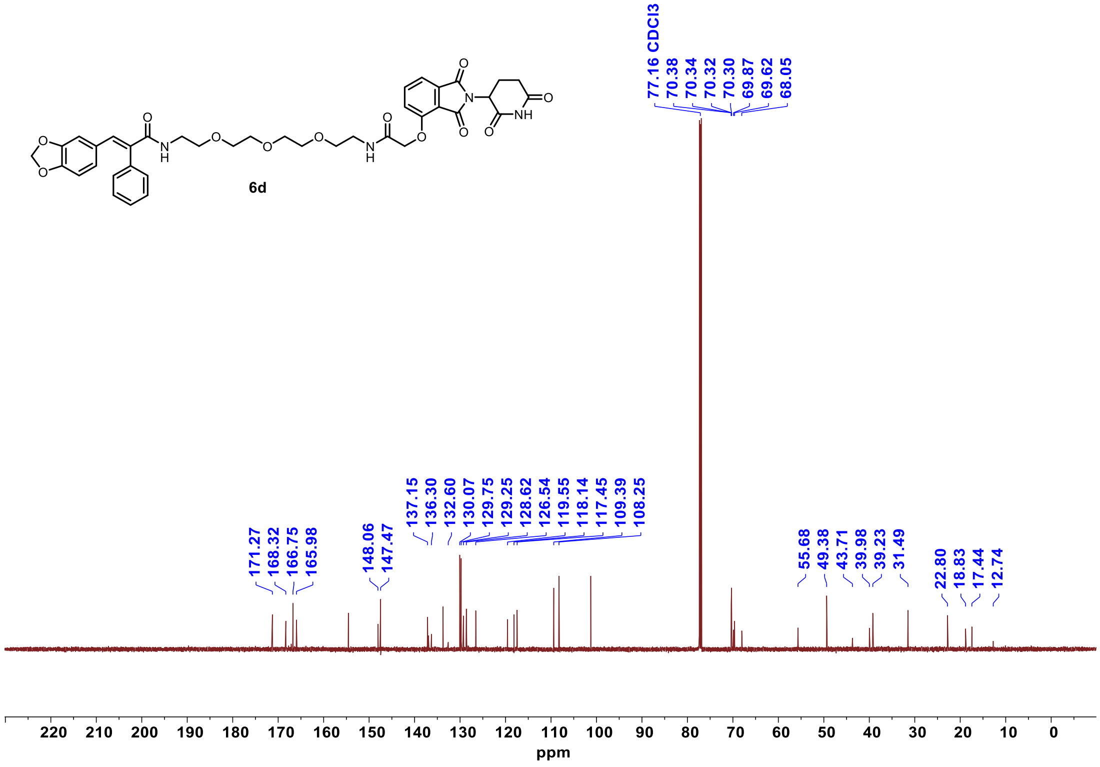

^{1}H NMR **7a**

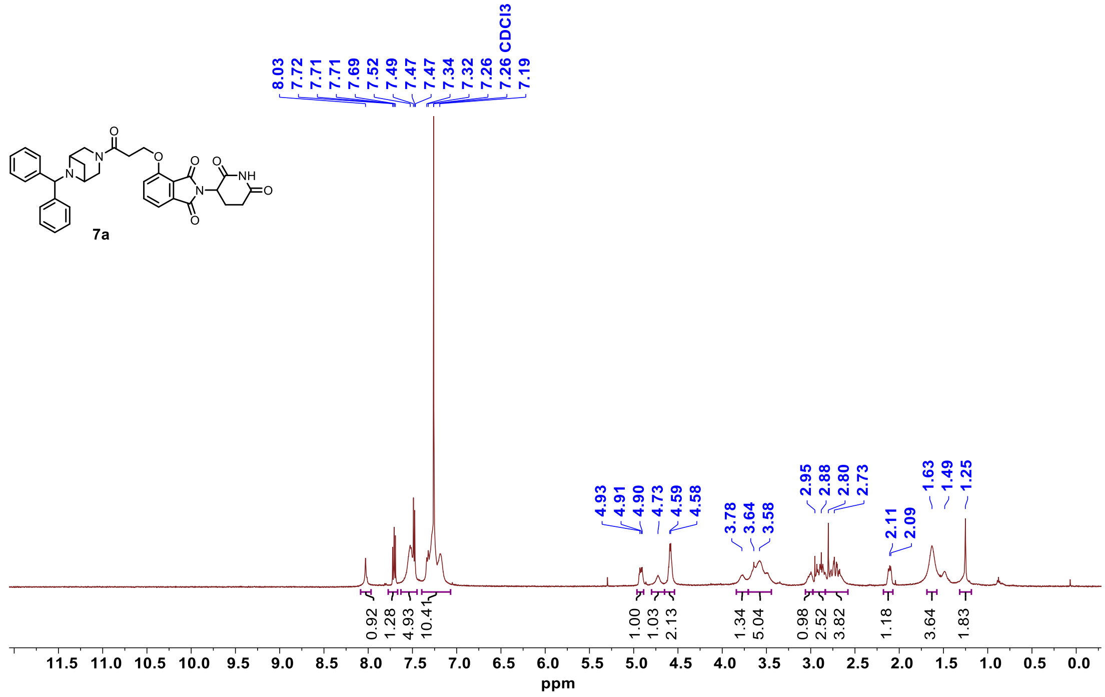

^{13}C NMR **7a**

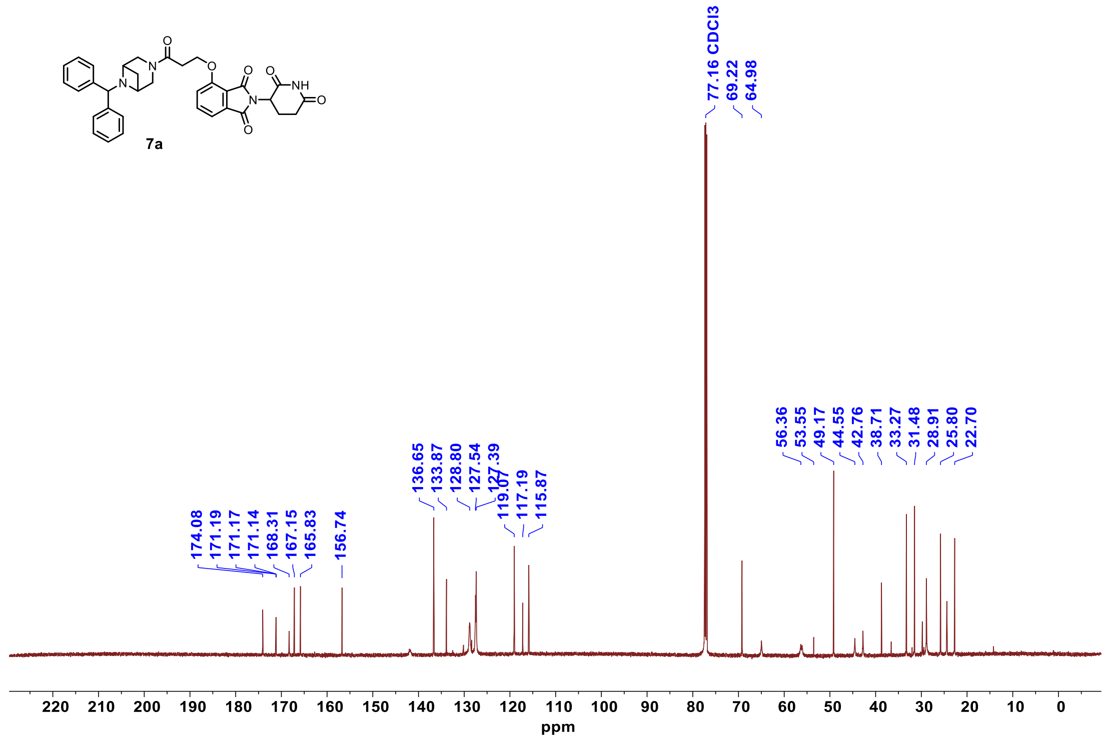

^{1}H NMR **7b**

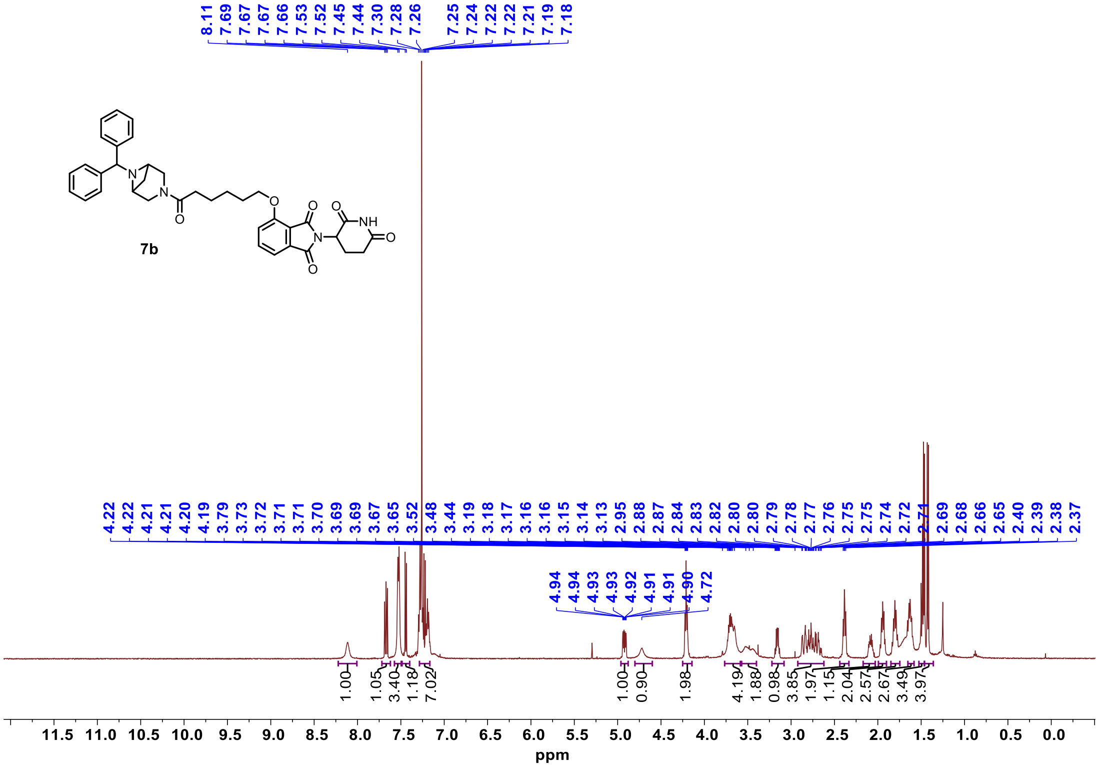

^{13}C NMR **7b**

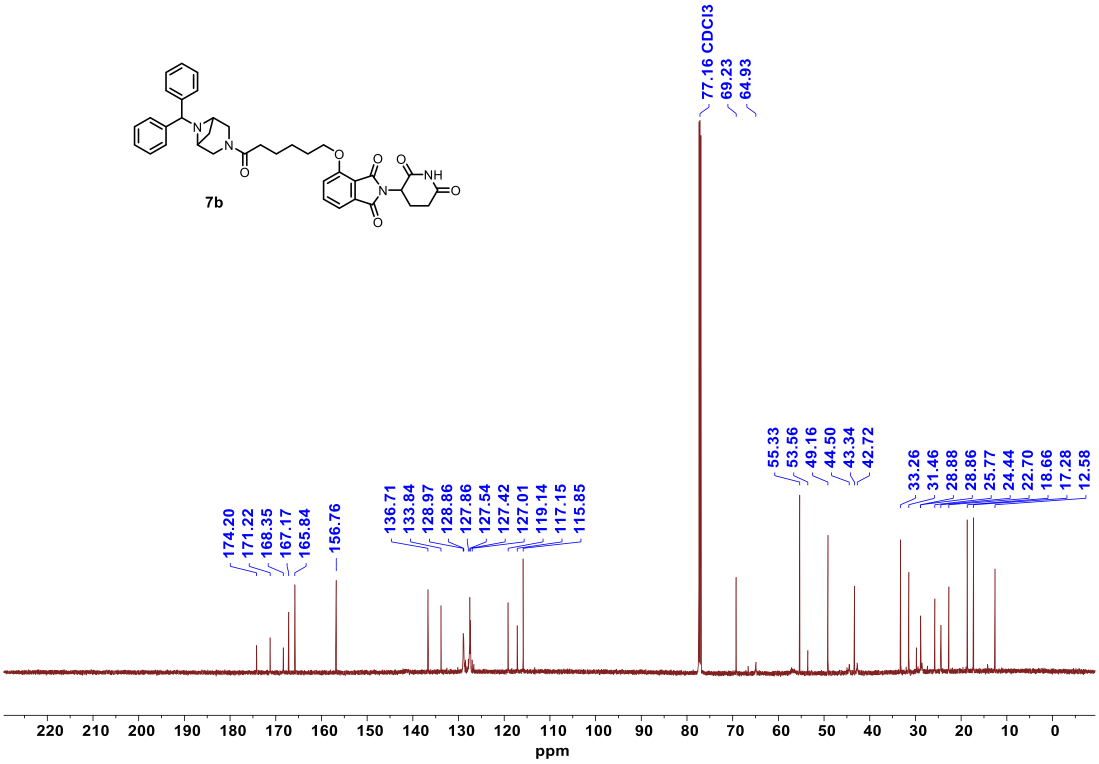

^{1}H NMR **7b-neg**

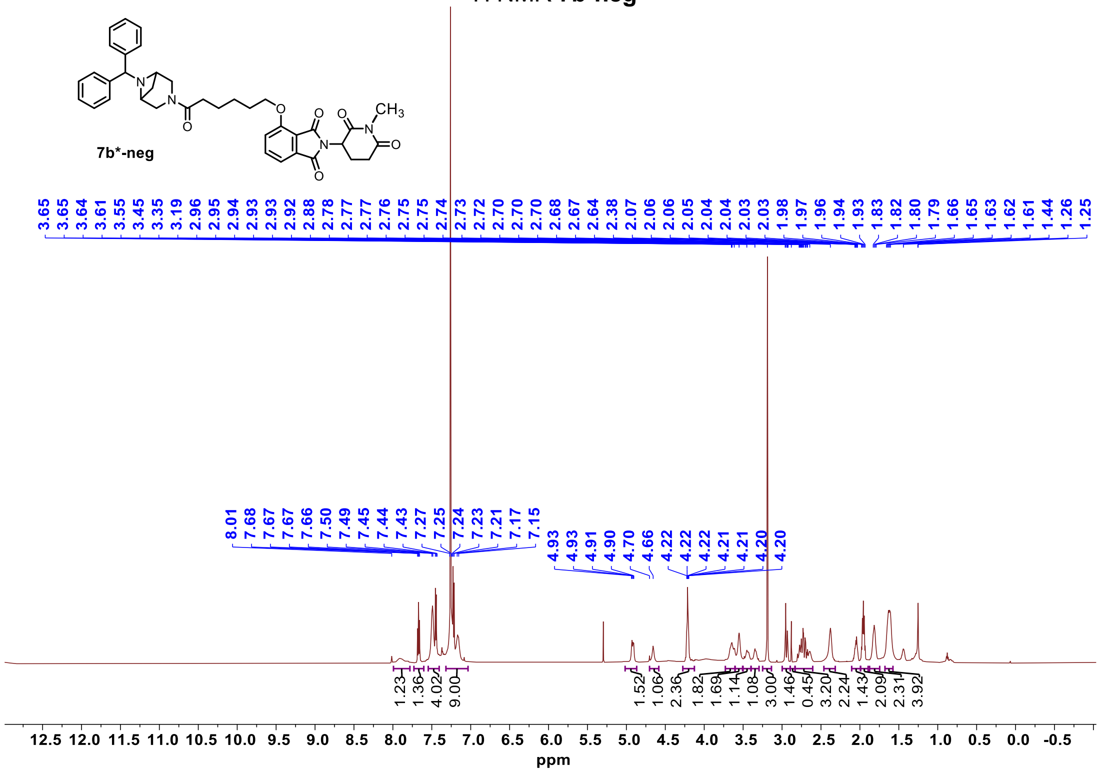

^{13}C NMR **7b-neg**

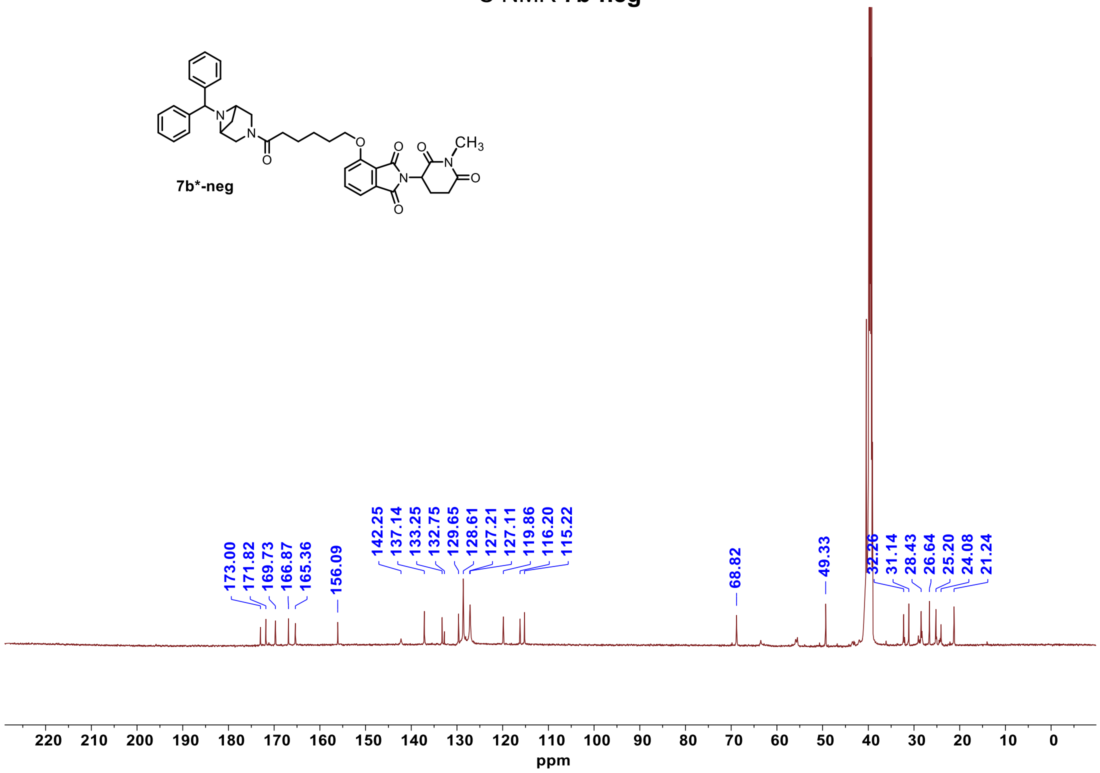

^{1}H NMR **7c**

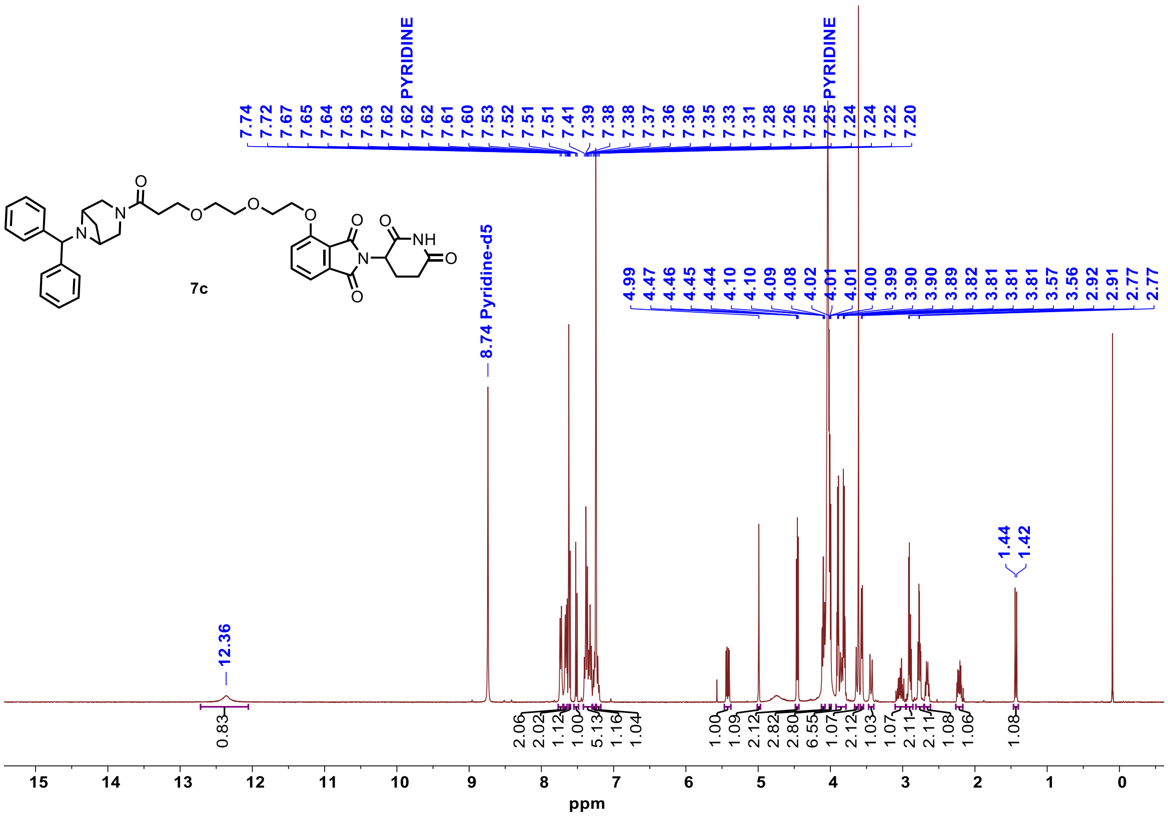

^{13}C NMR **7c**

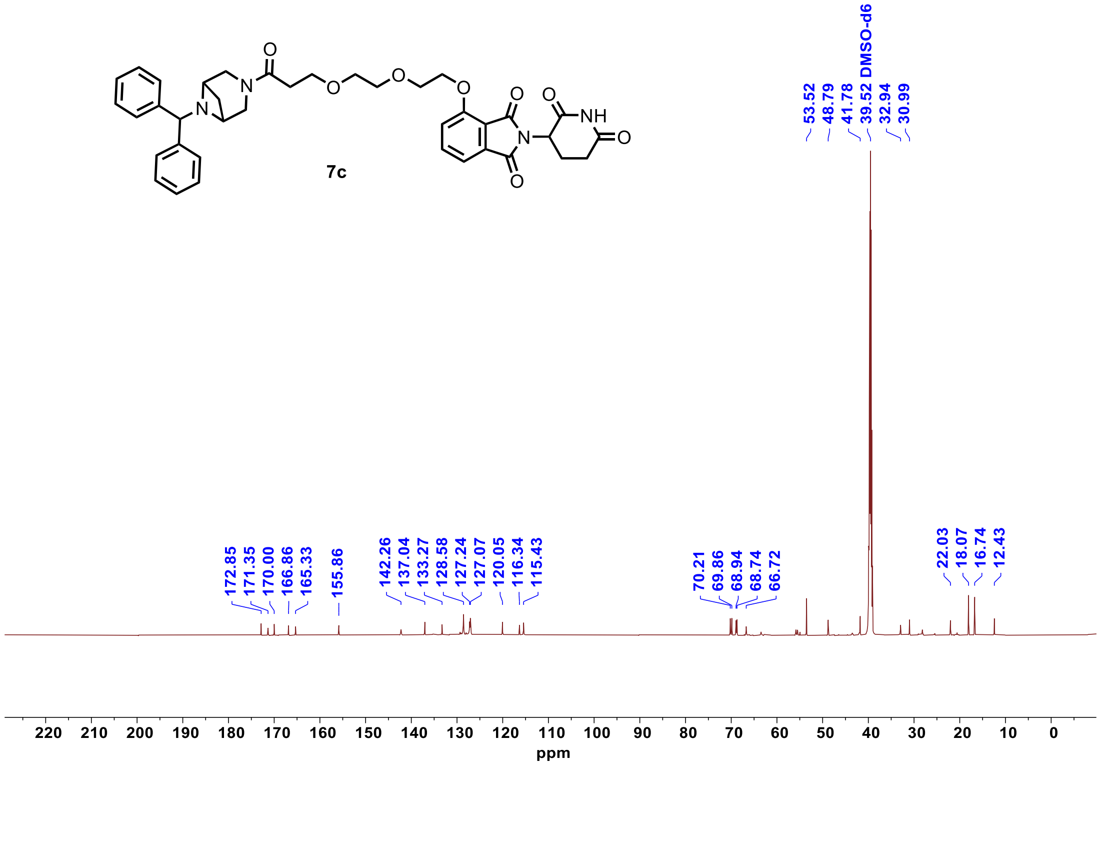

^{1}H NMR **7d**

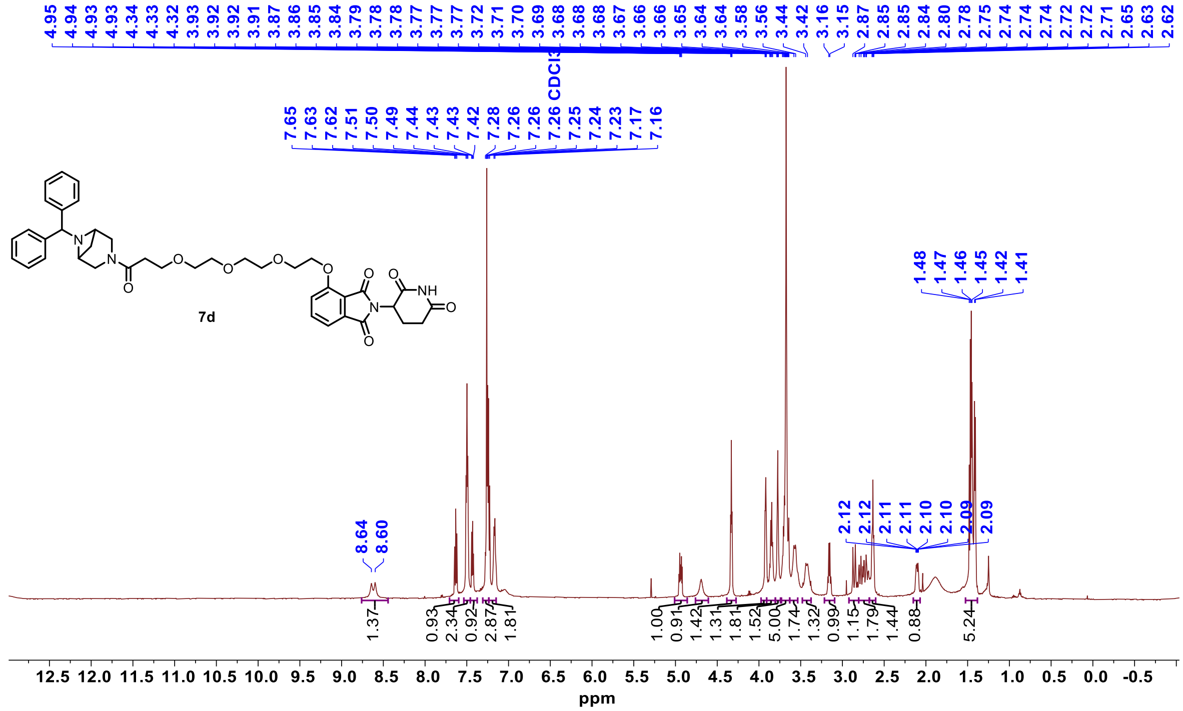

^{13}C NMR **7d**

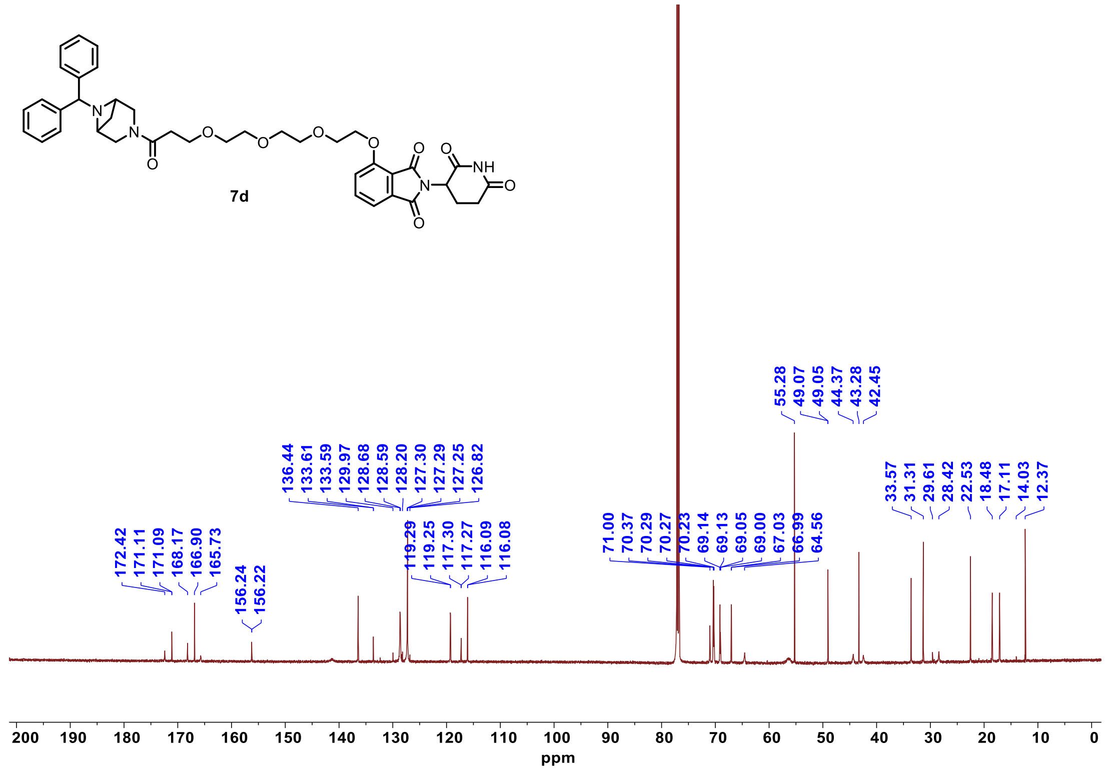

^{1}H NMR **8a**

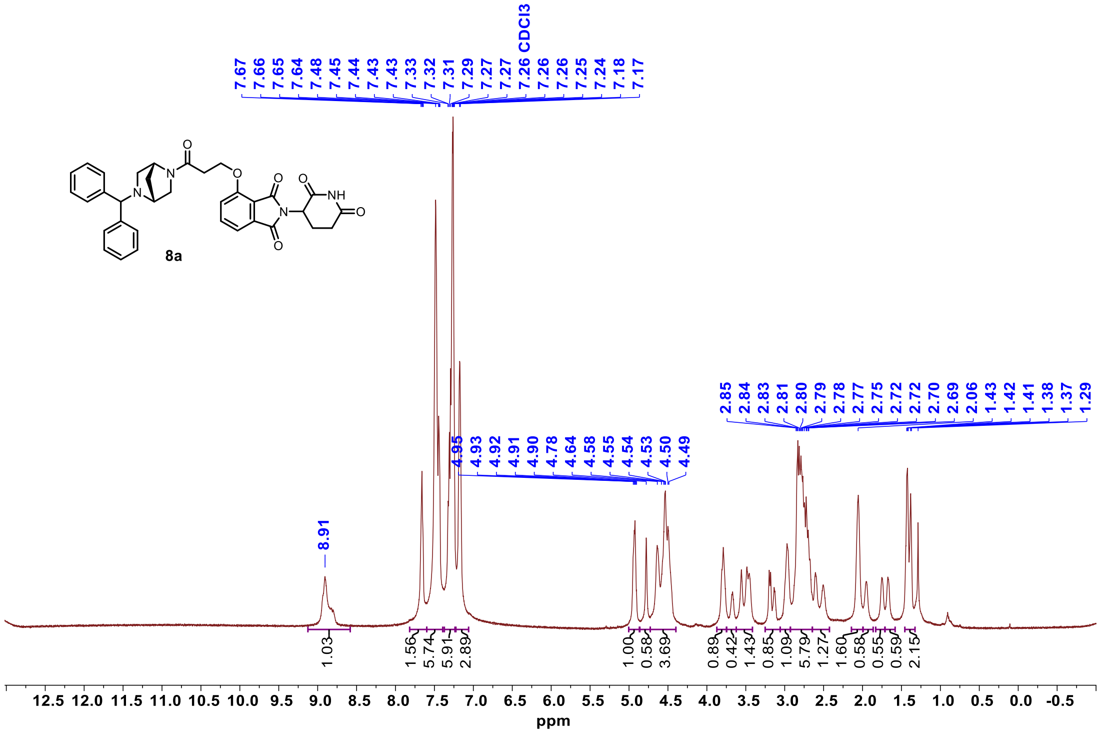

^{13}C NMR **8a**

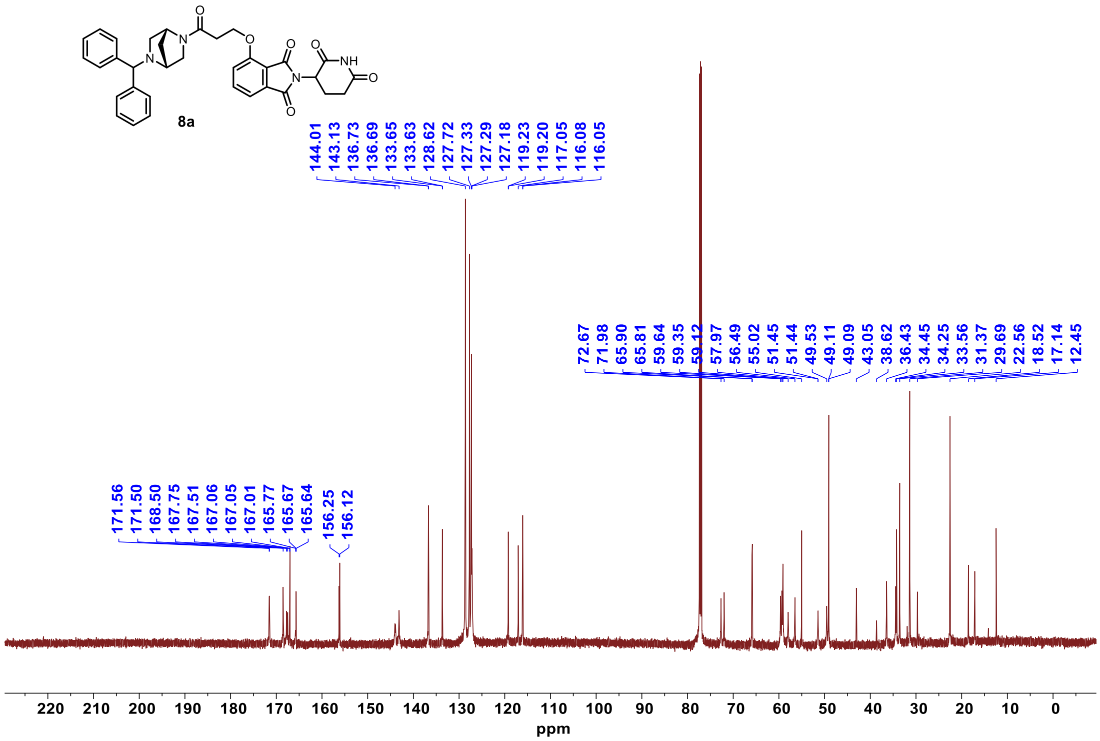

^{1}H NMR **8b**

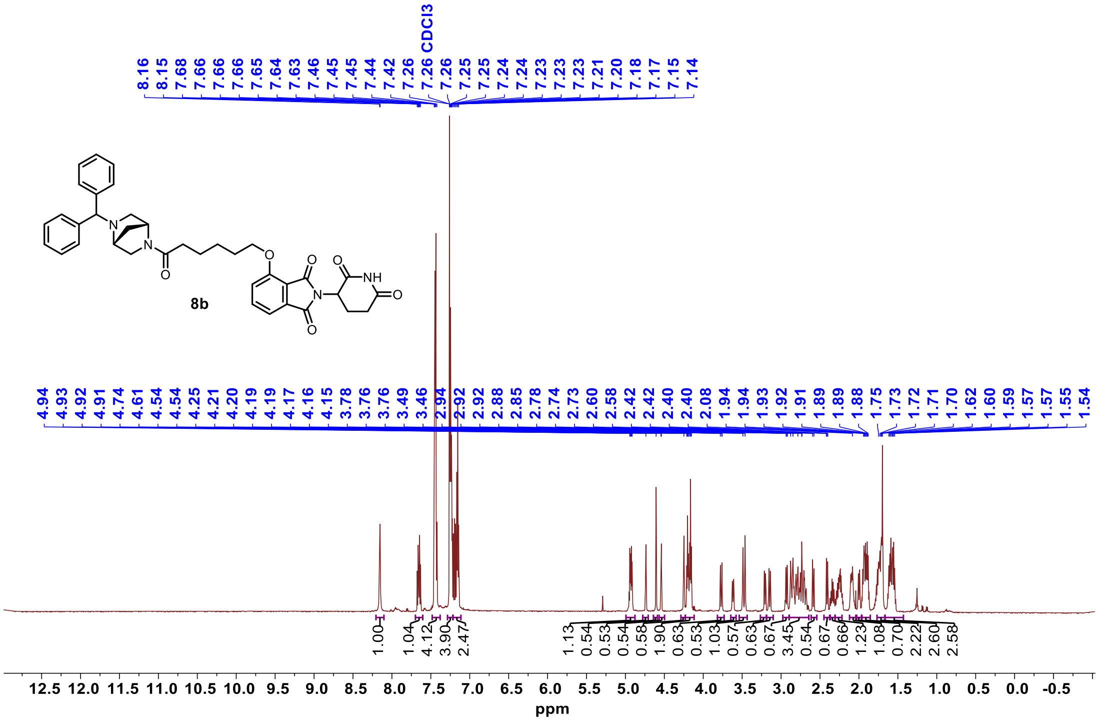

^{13}C NMR **8b**

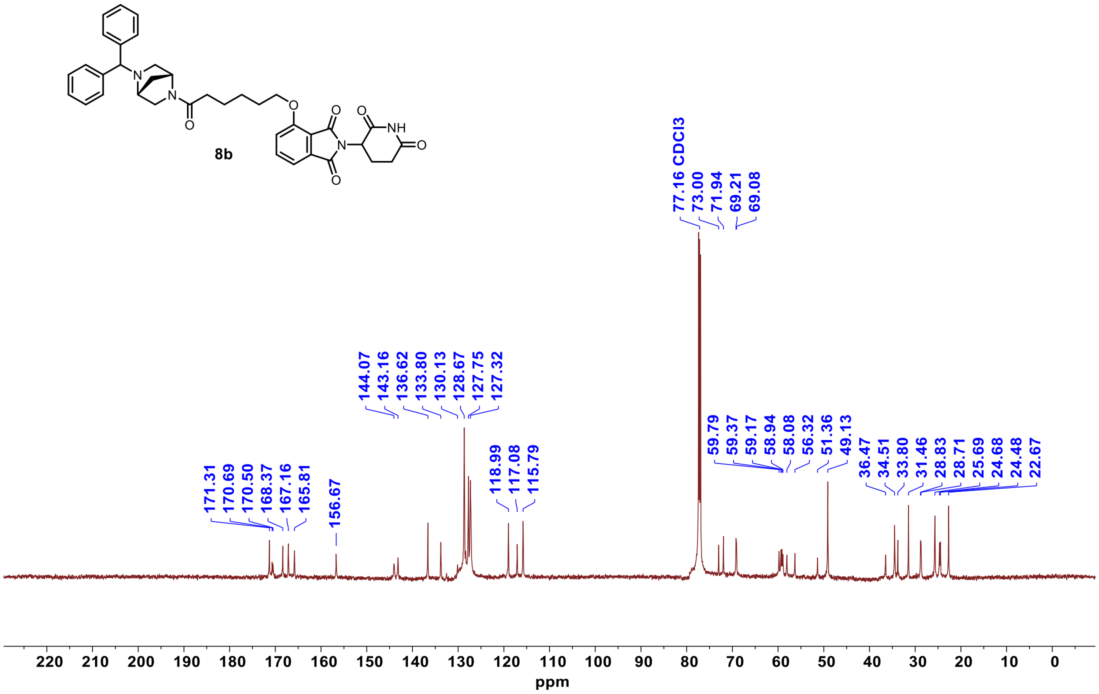

^{1}H NMR **8c**

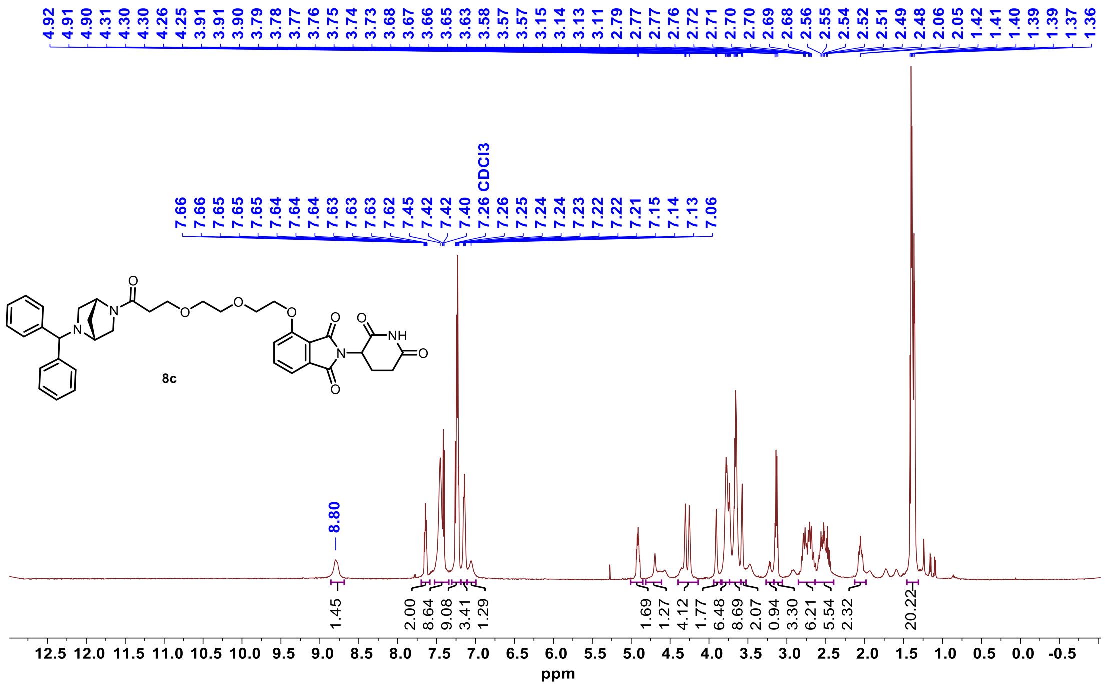

^{13}C NMR **8c**

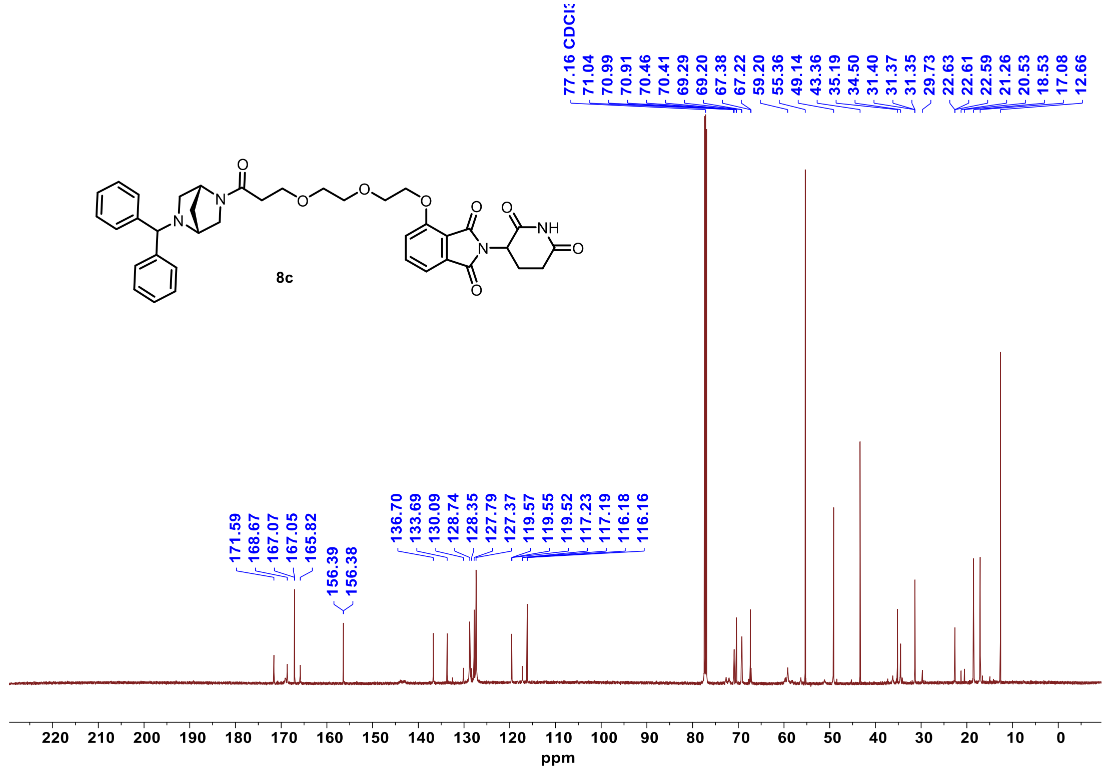

^{1}H NMR **8d**

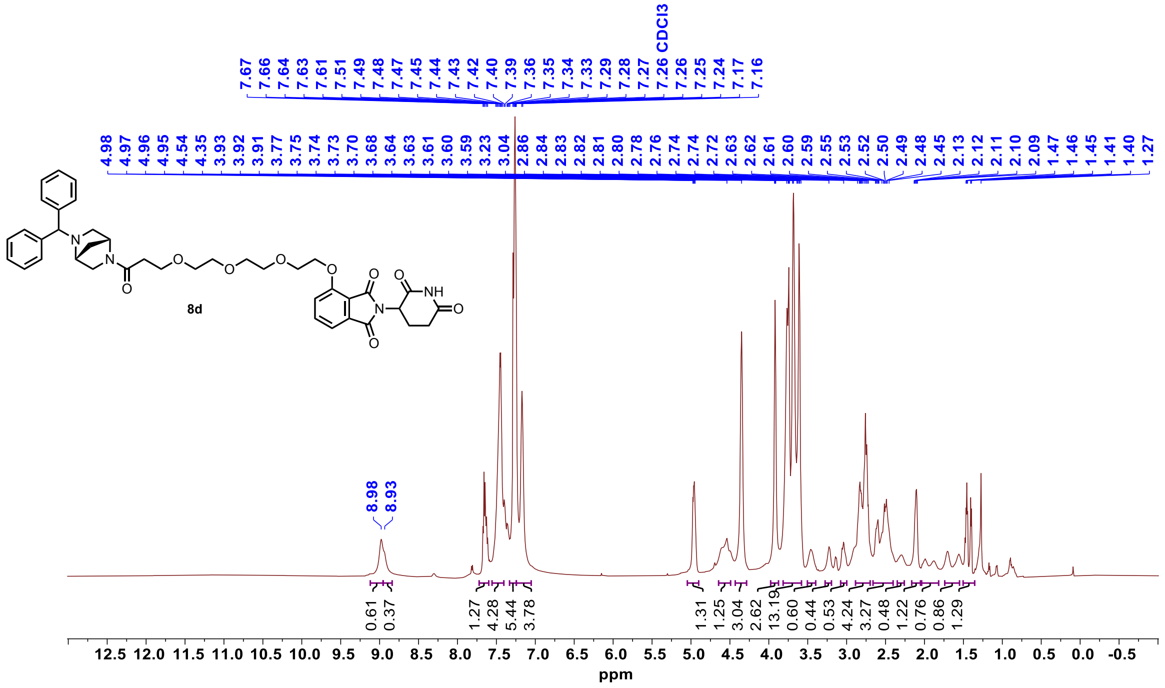

^{13}C NMR **8d**

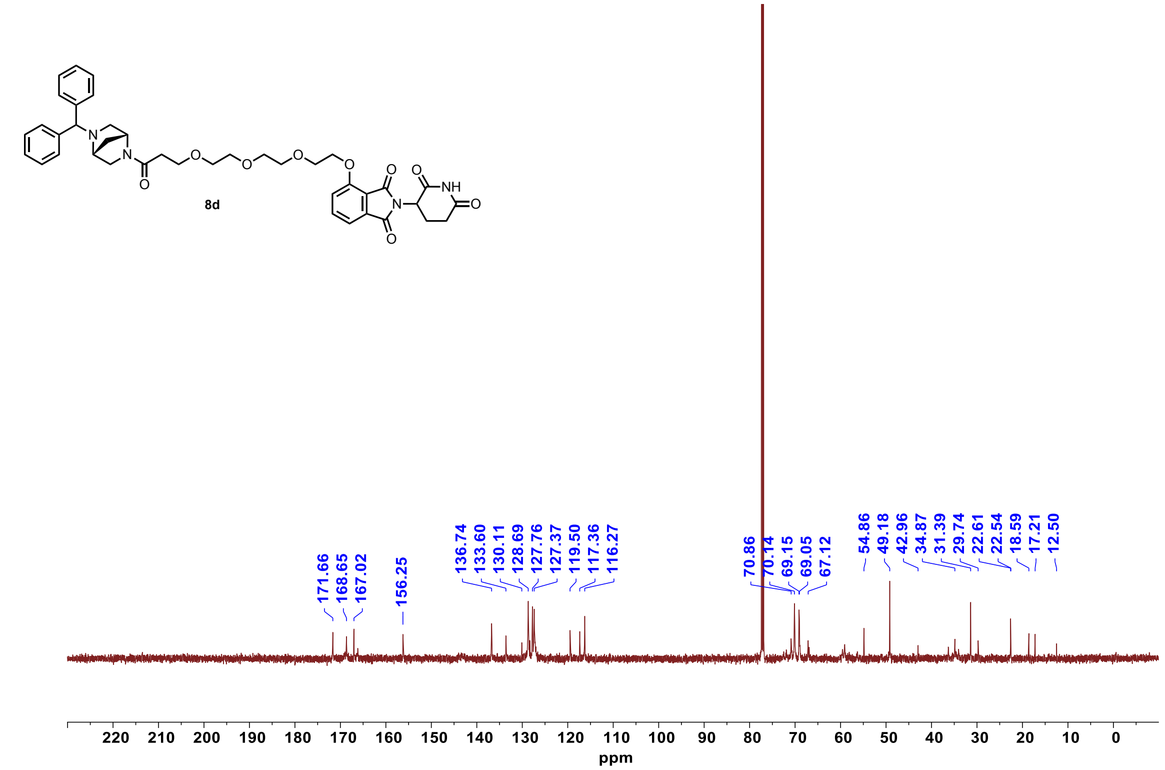

^{1}H NMR **8d*-neg**

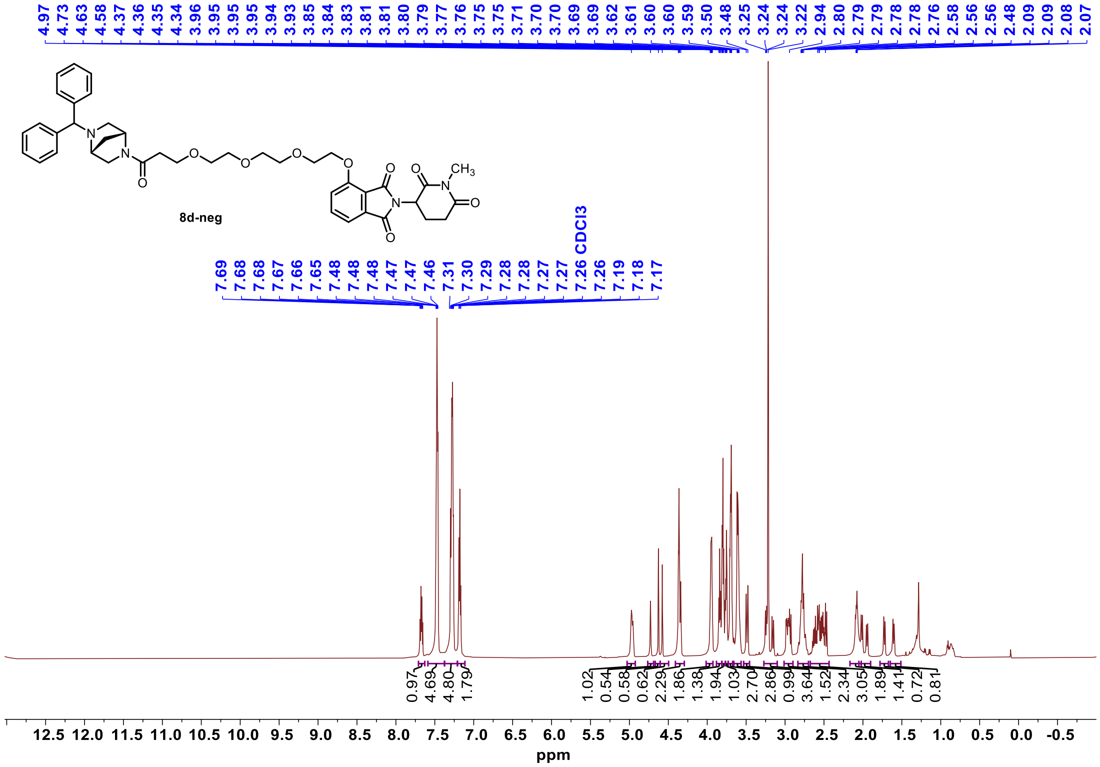

^{13}C NMR **8d*-neg**

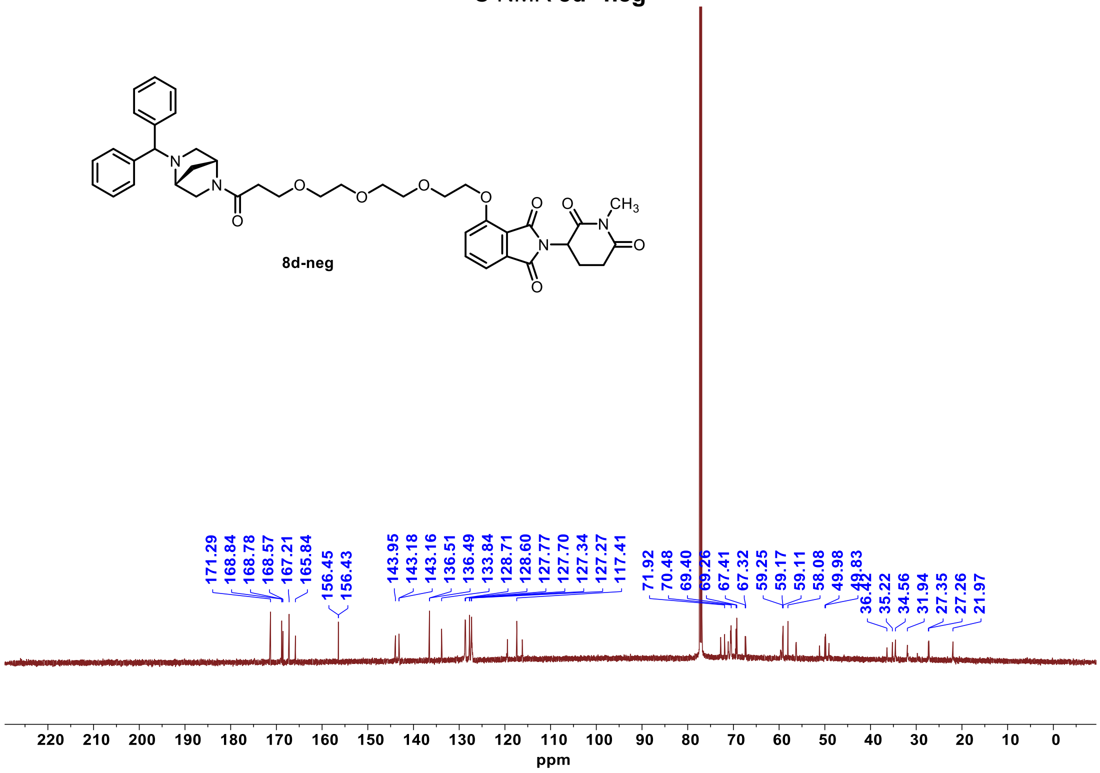

^{1}H NMR **9a**

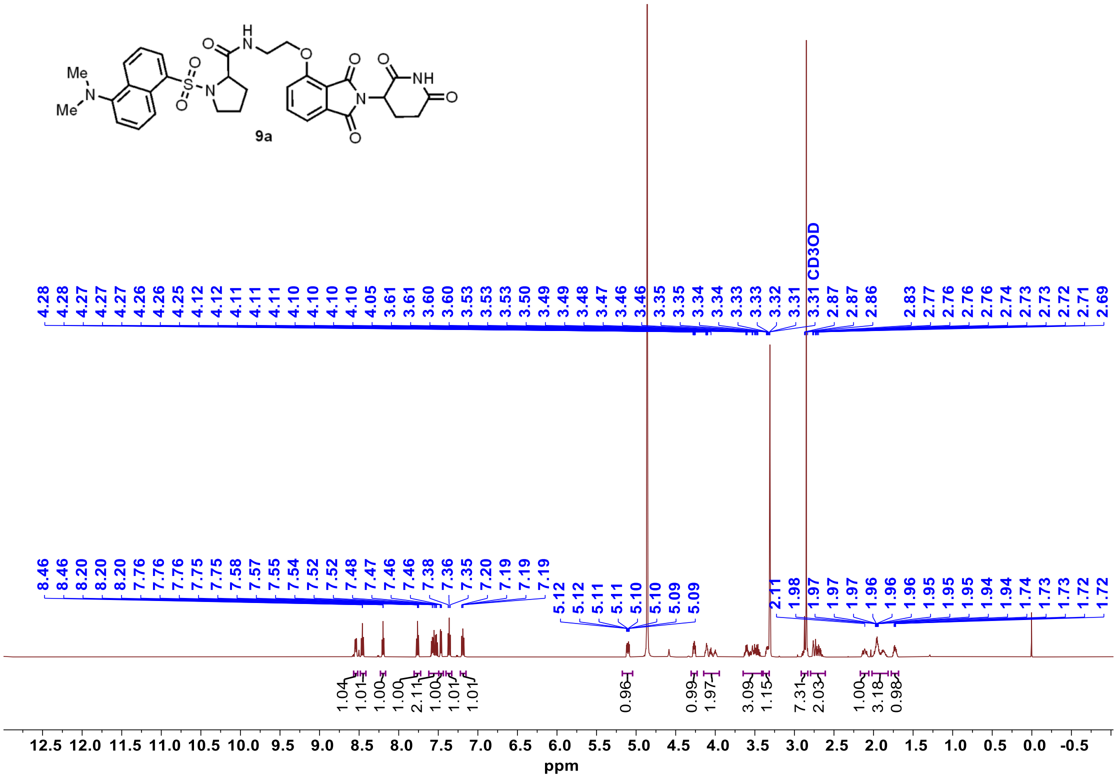

^{13}C NMR **9a**

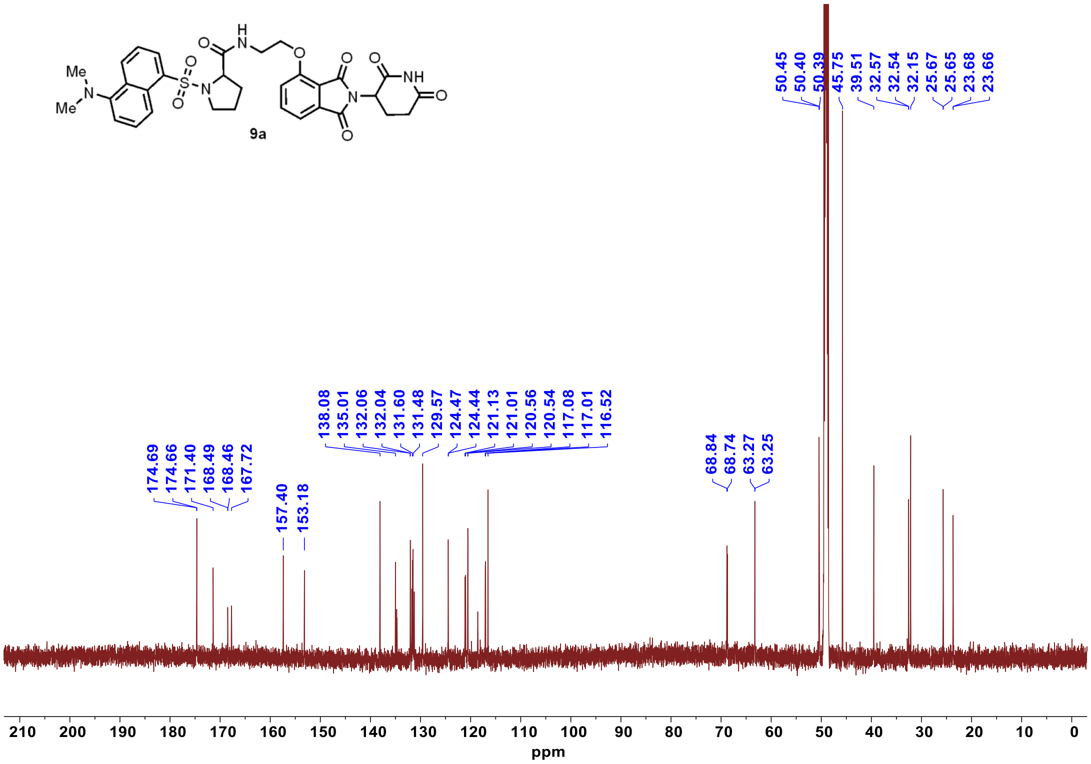

^{1}H NMR **9b**

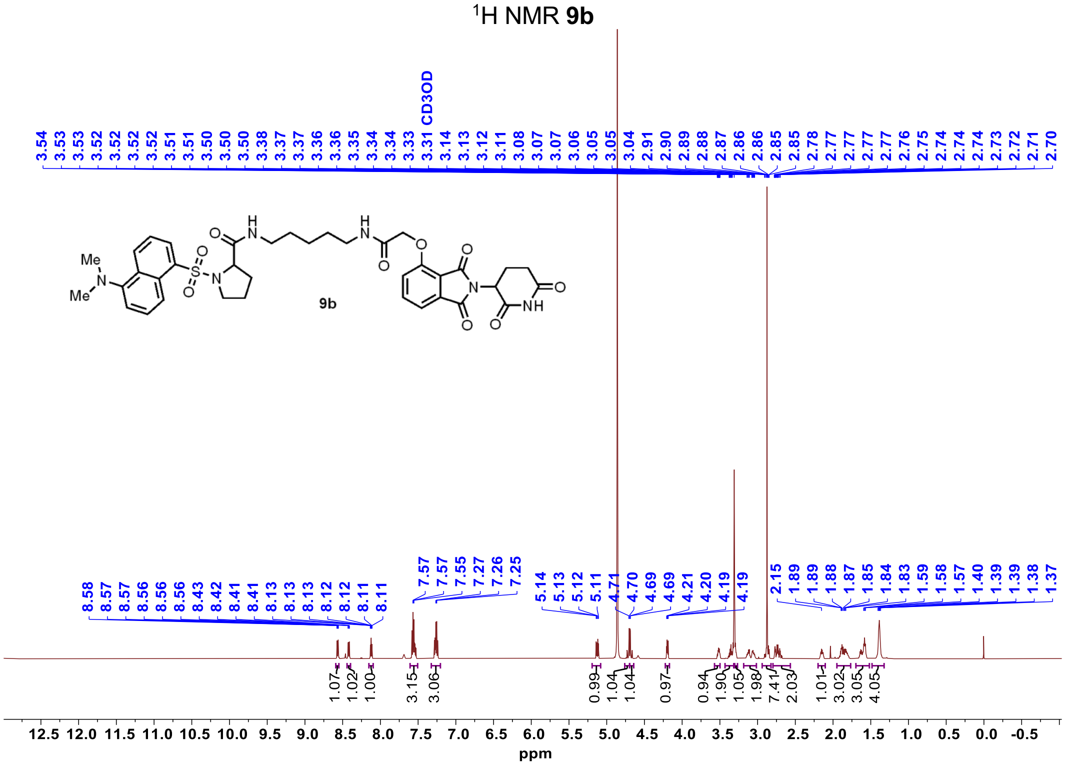

^{13}C NMR **9b**

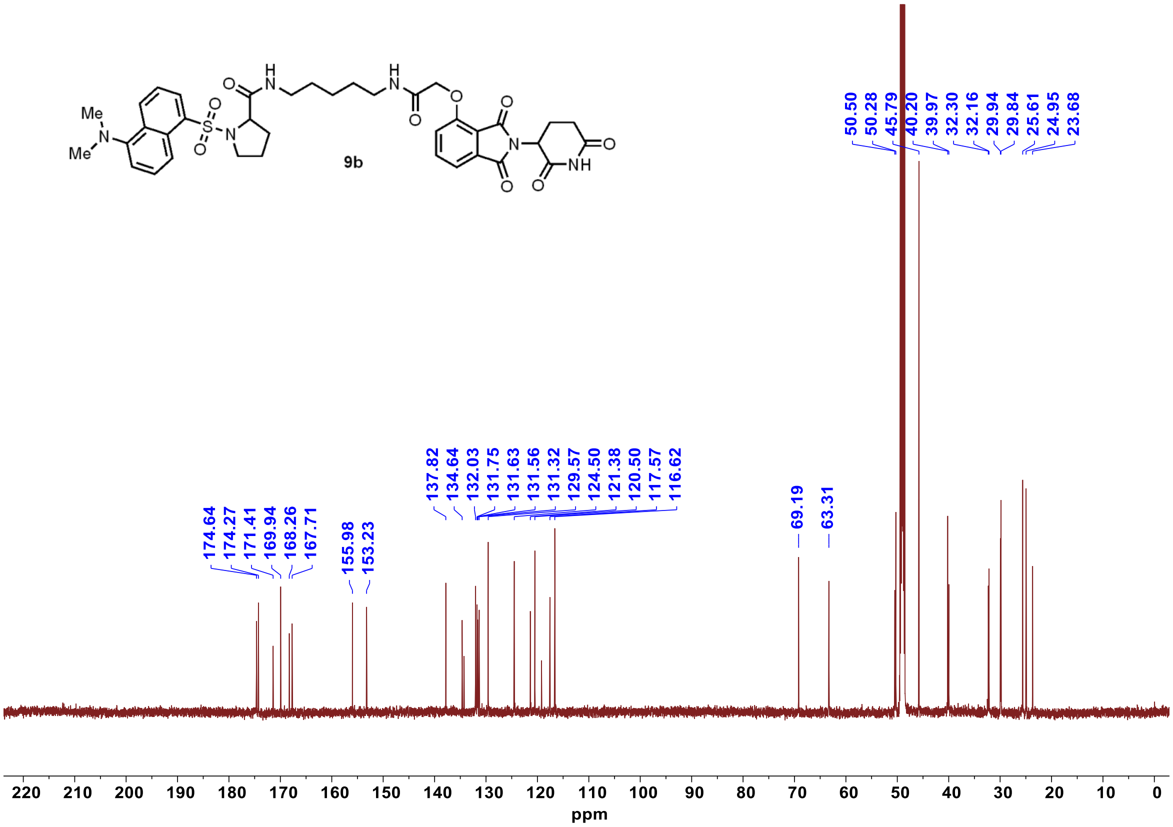

^{1}H NMR **9c**

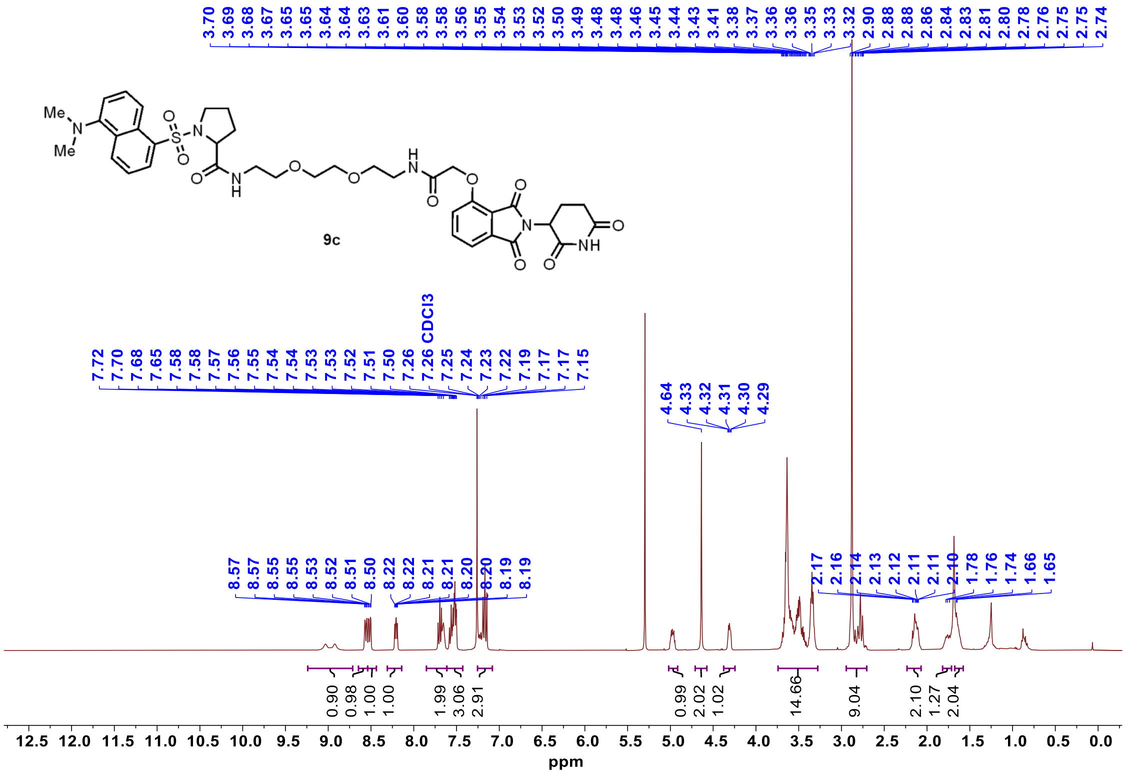

^{13}C NMR **9c**

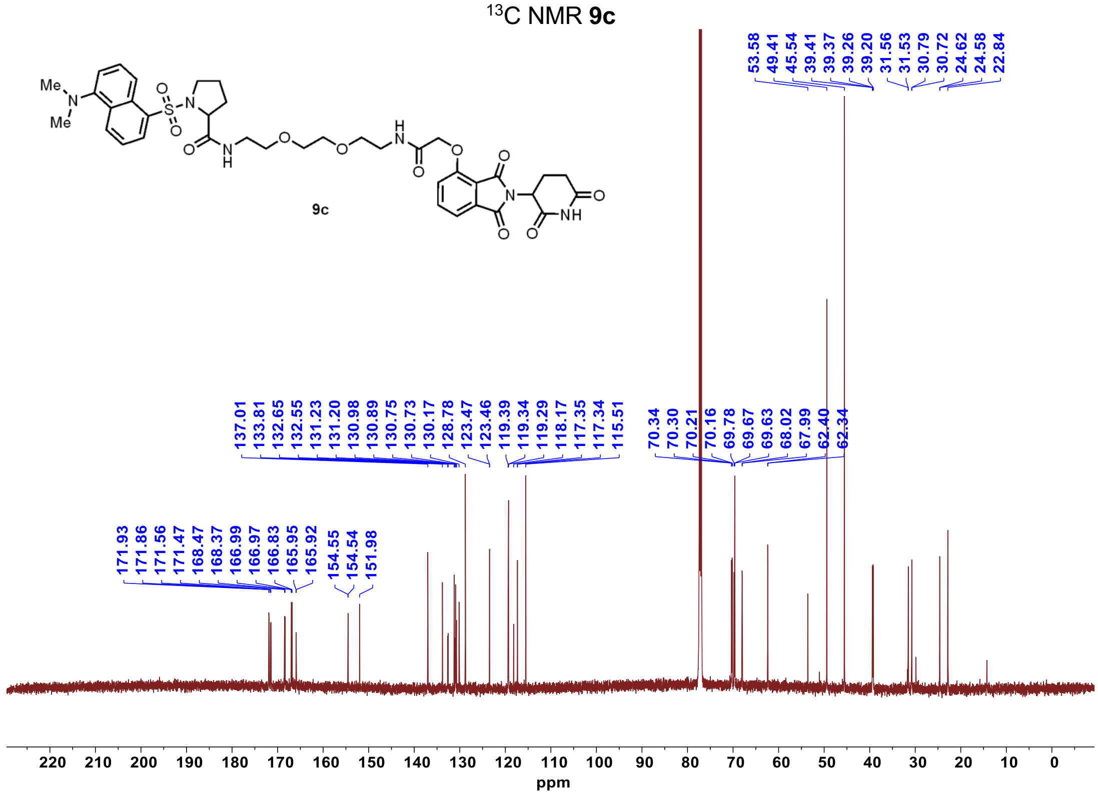

^{1}H NMR **9d**

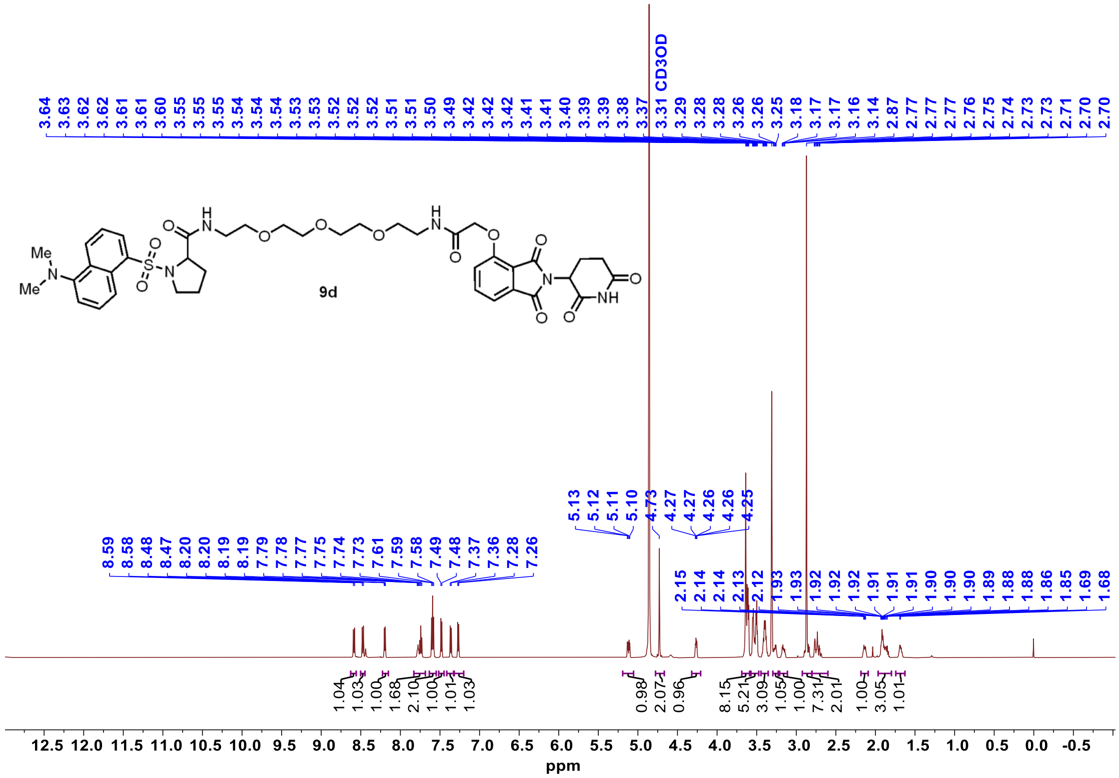

^{13}C NMR **9d**

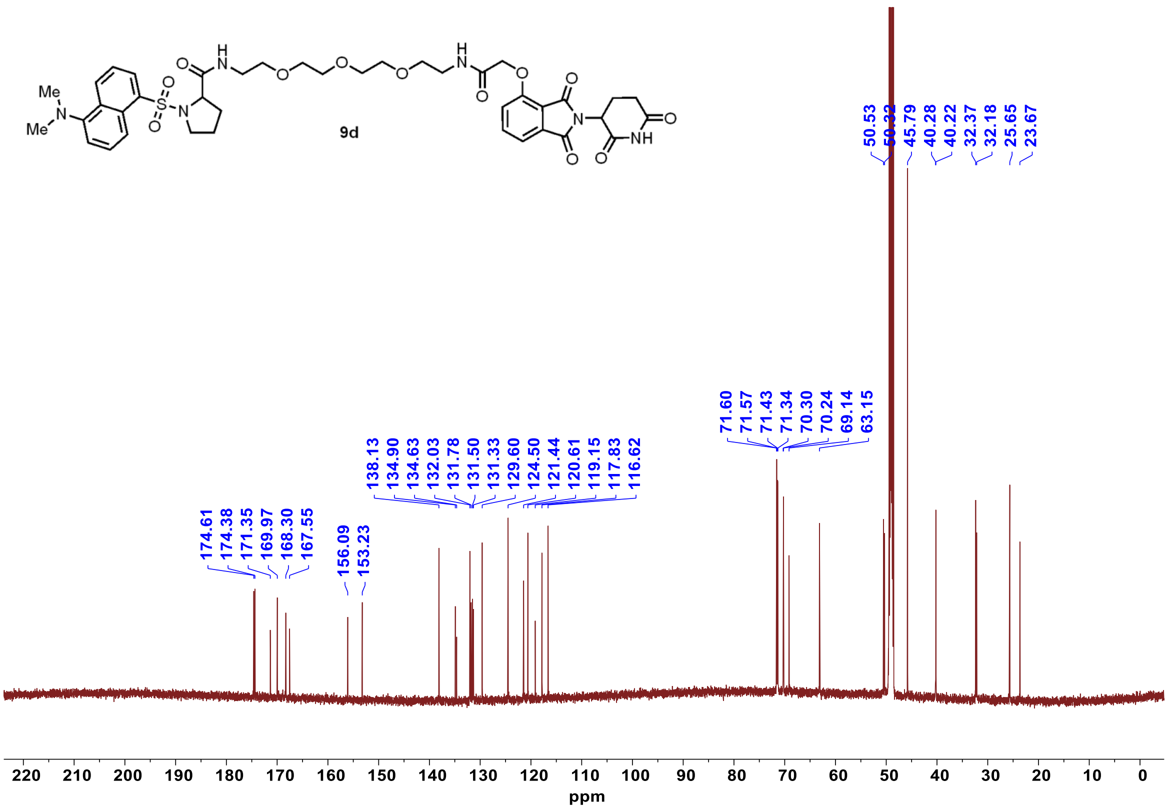

^{1}H NMR **10a**

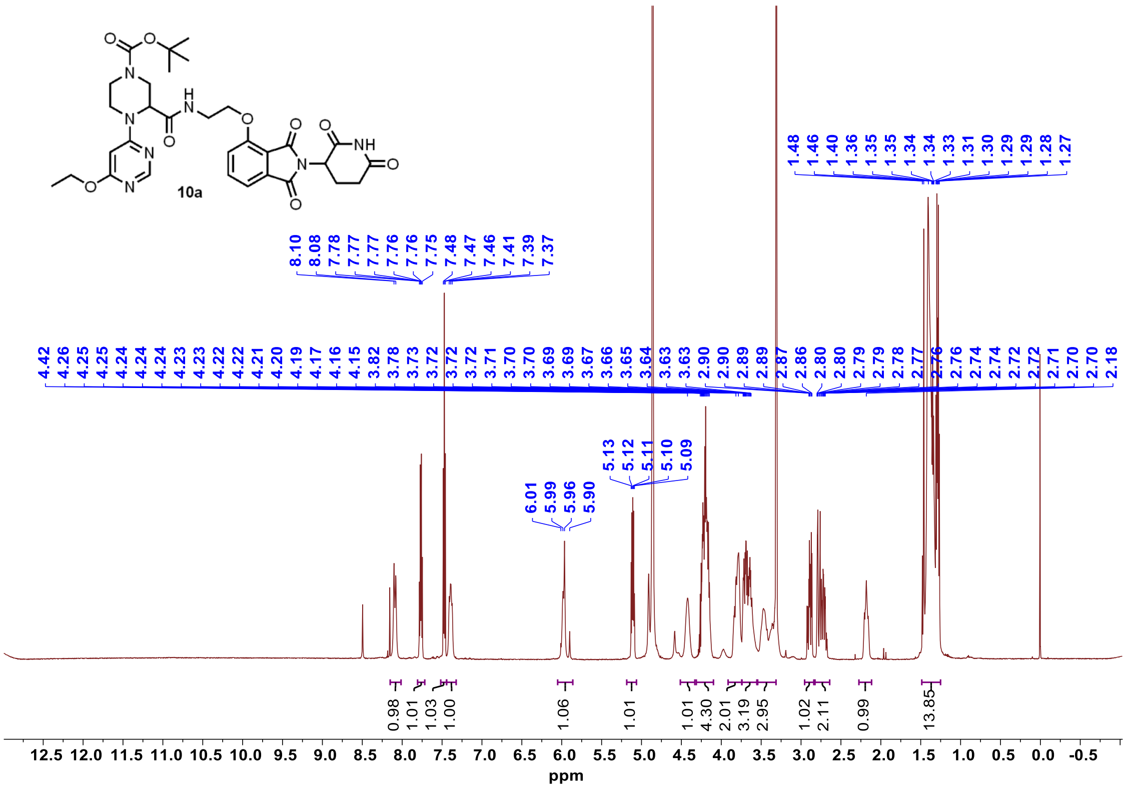

^{13}C NMR **10a**

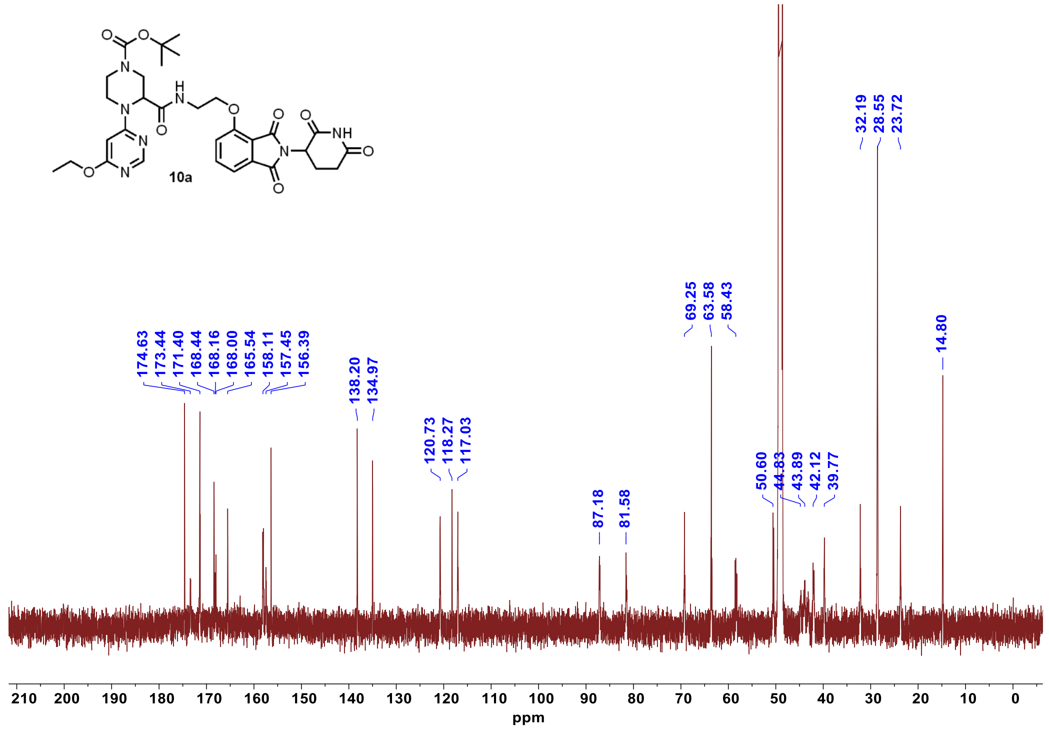

^{1}H NMR **10b**

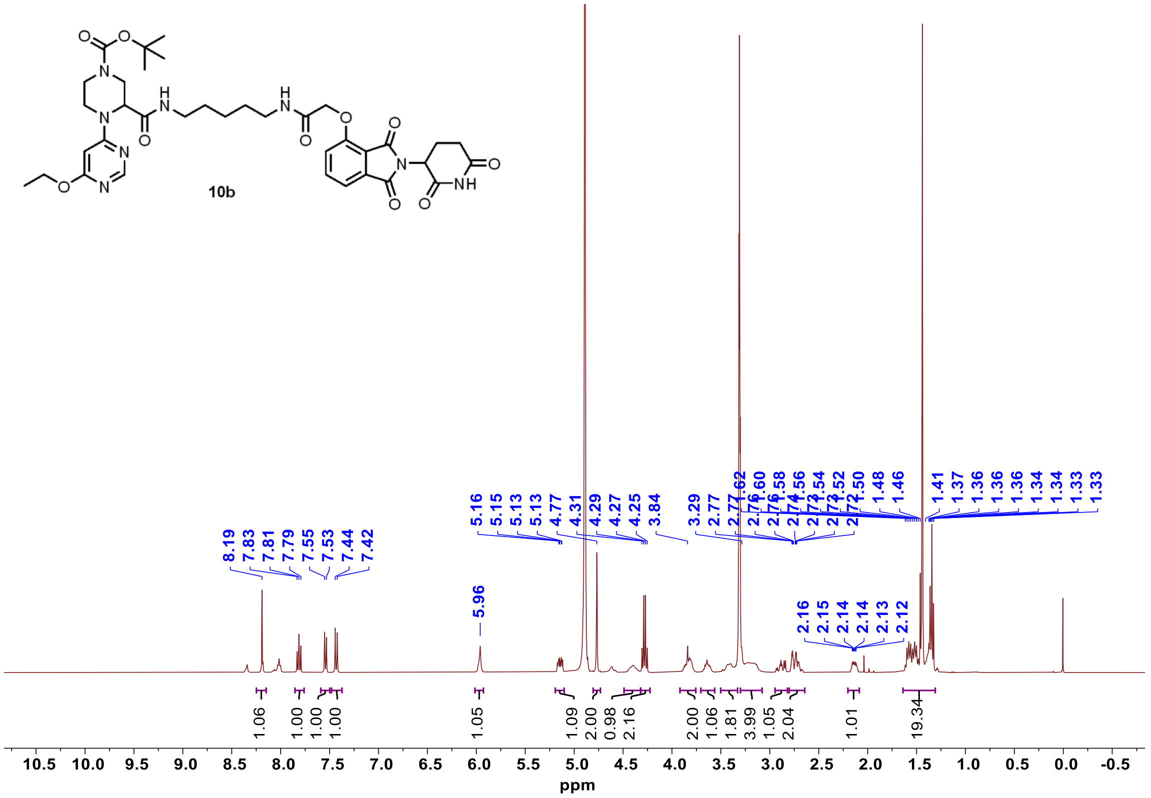

^{13}C NMR **10b**

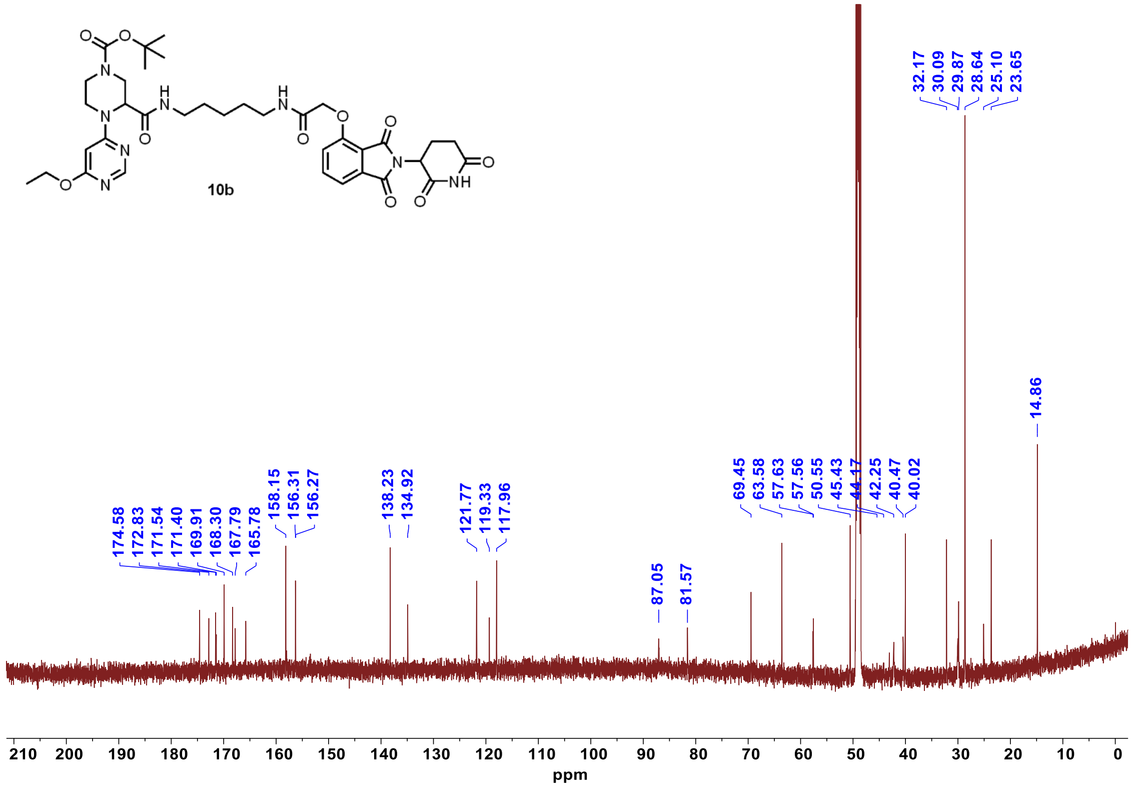

^{1}H NMR **10c**

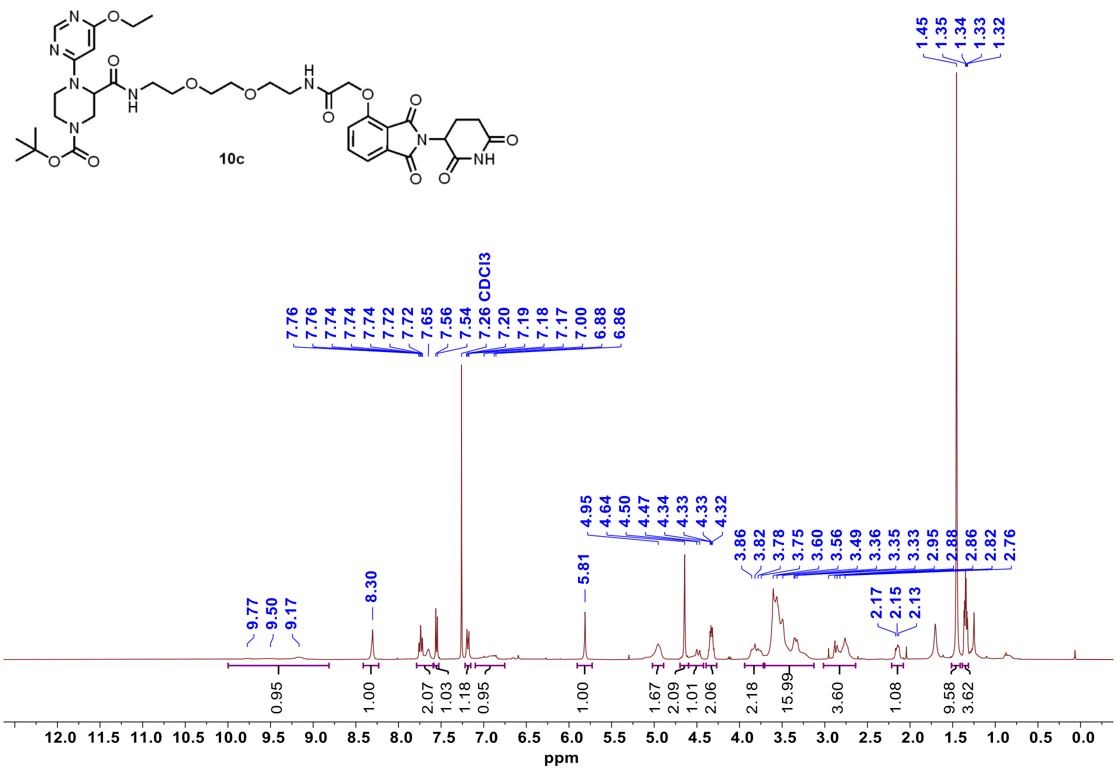

^{13}C NMR **10c**

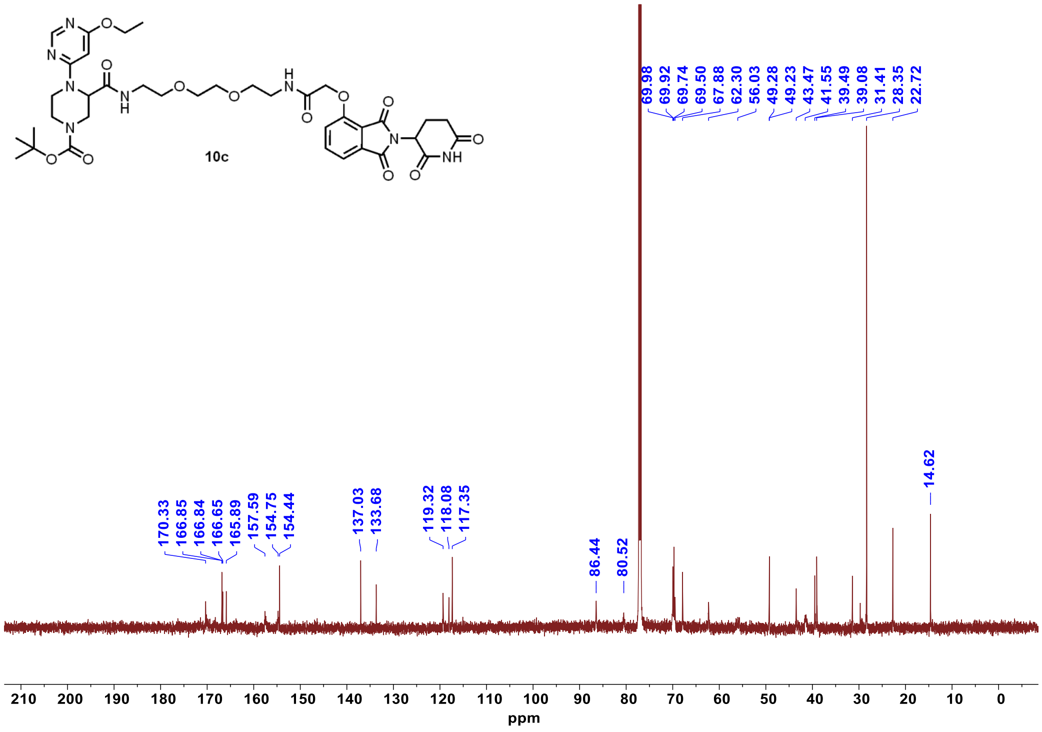

^{1}H NMR **10d**

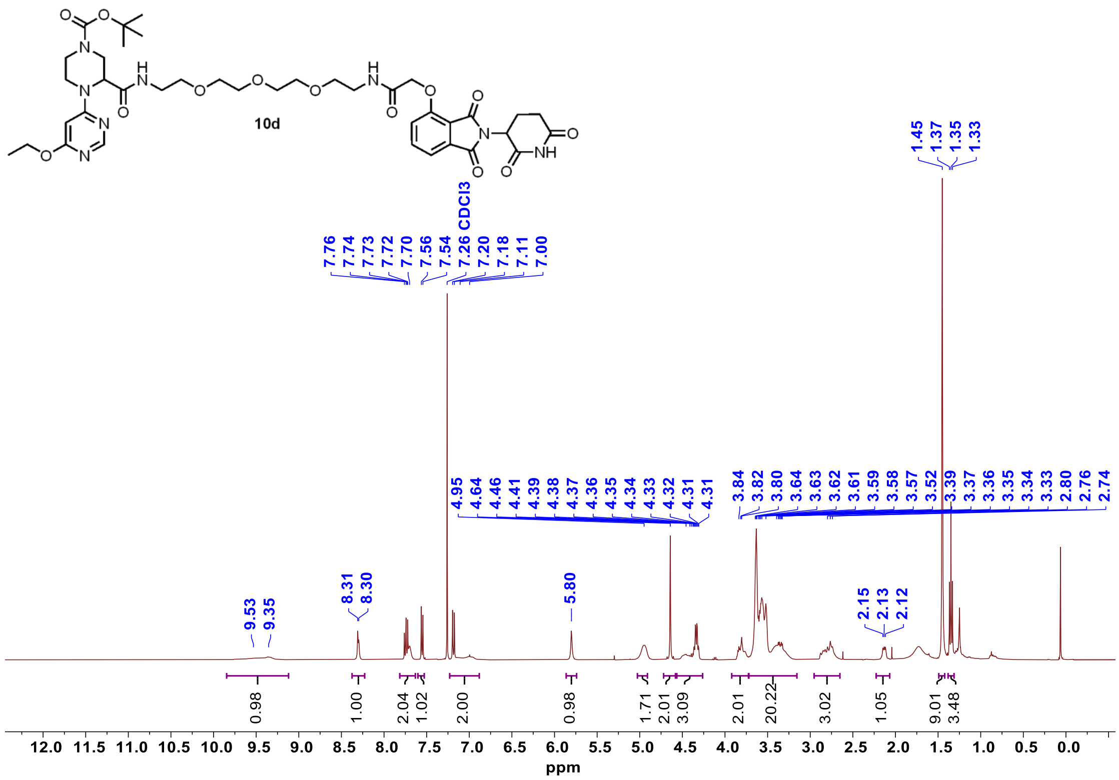

^{13}C NMR **10d**

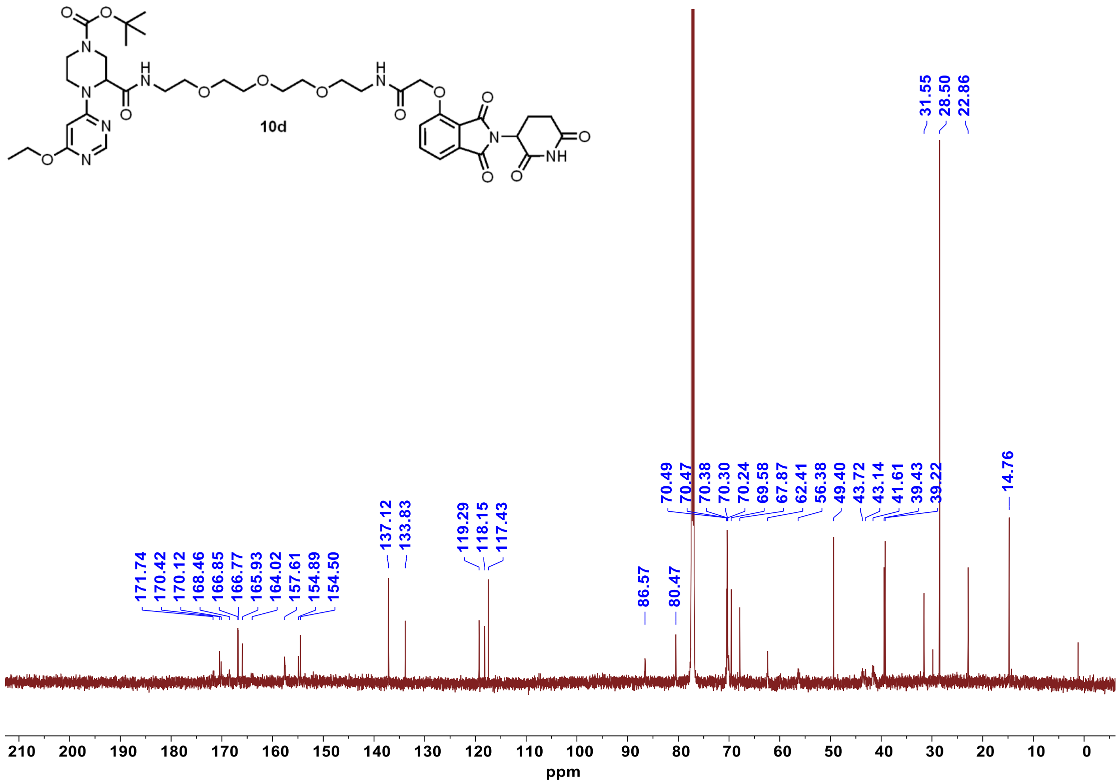

^{1}H NMR **10d-neg**

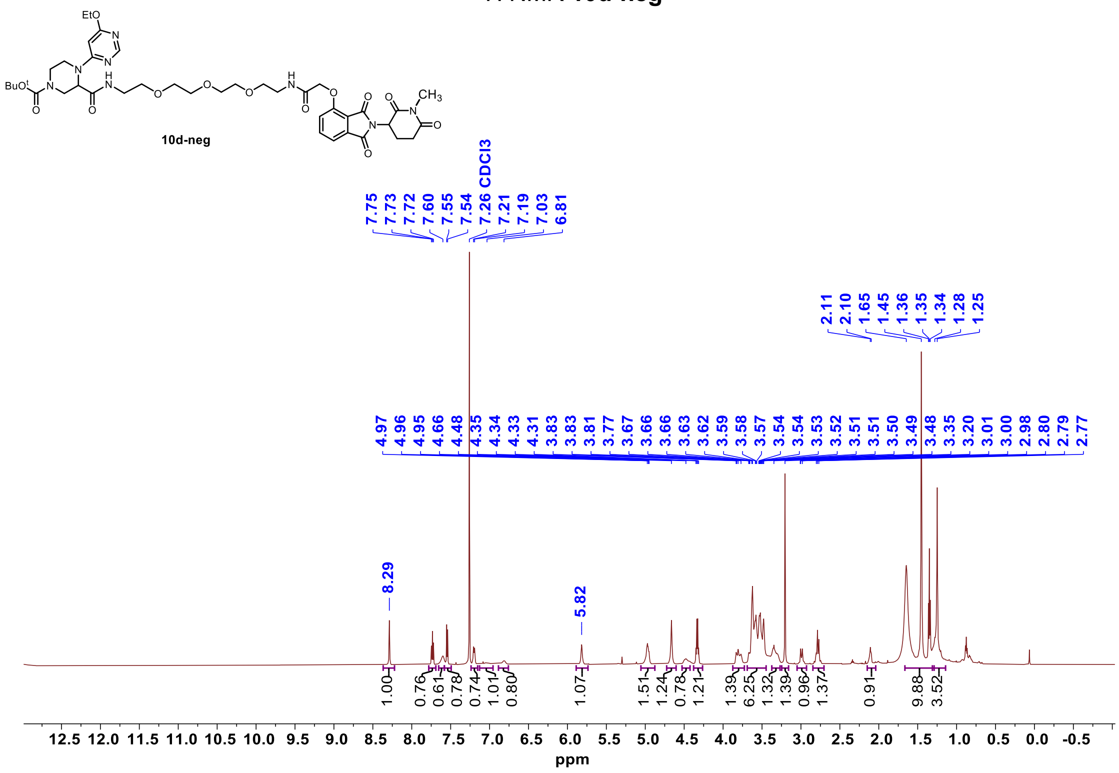

^{13}C NMR **10d-neg**

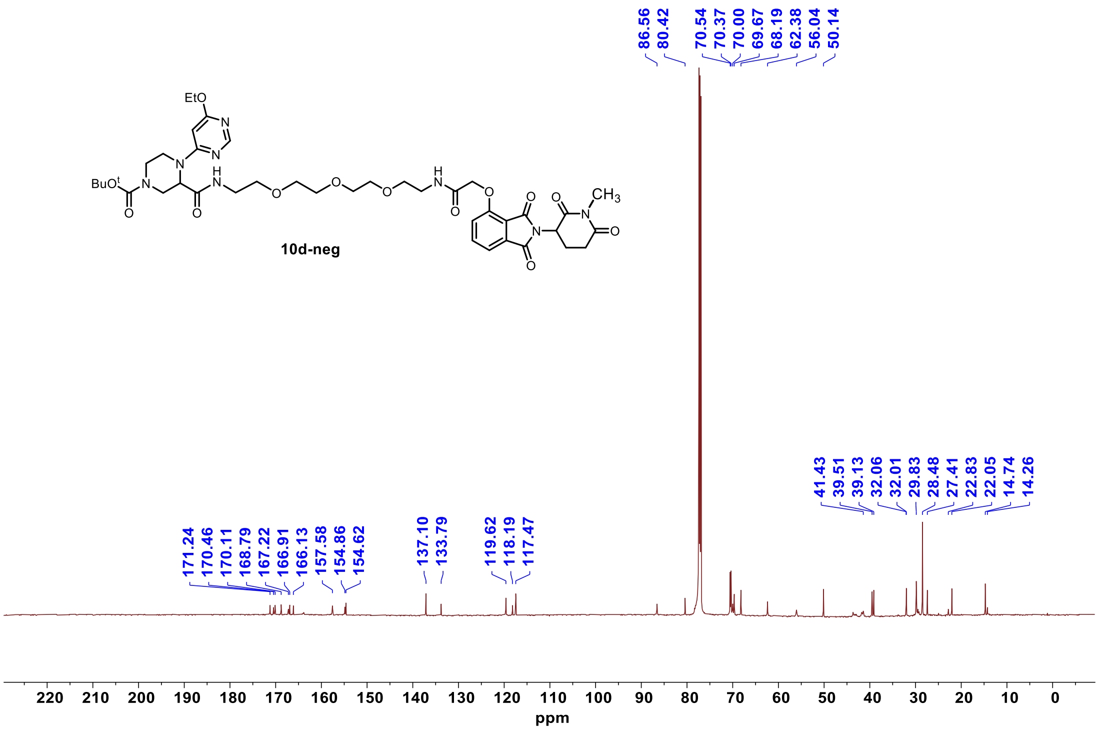

^{1}H NMR **11a**

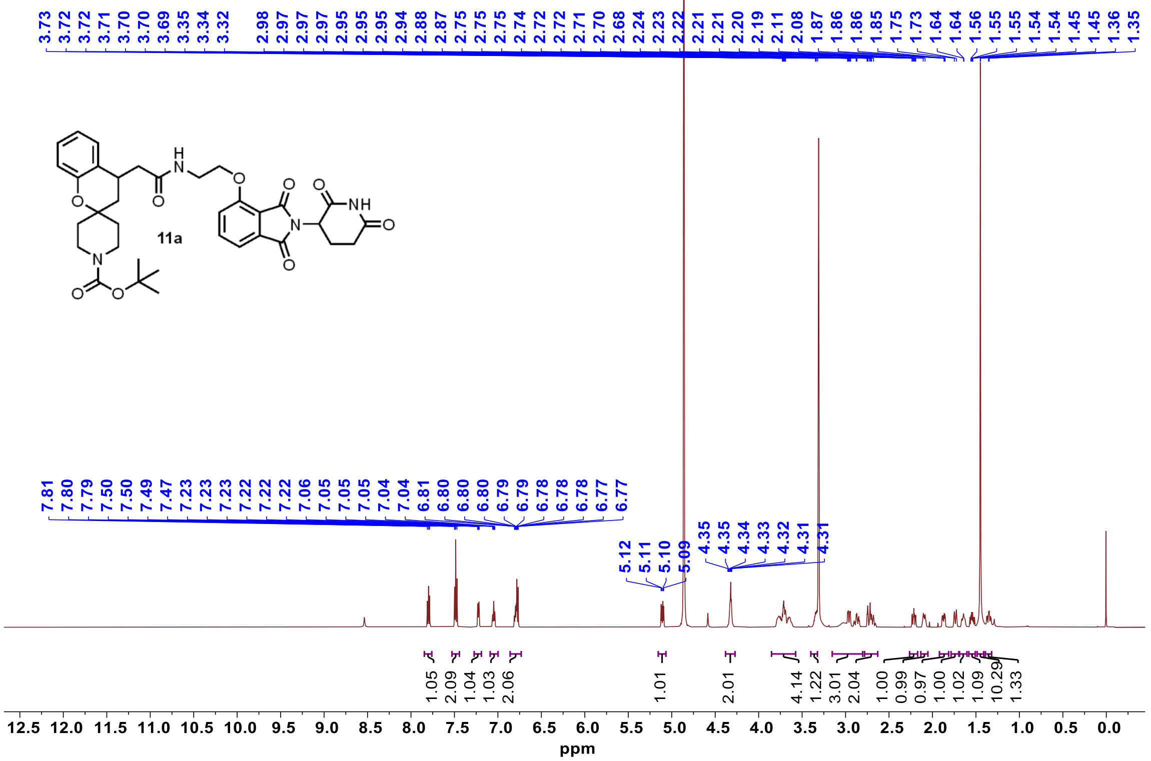

^{13}C NMR **11a**

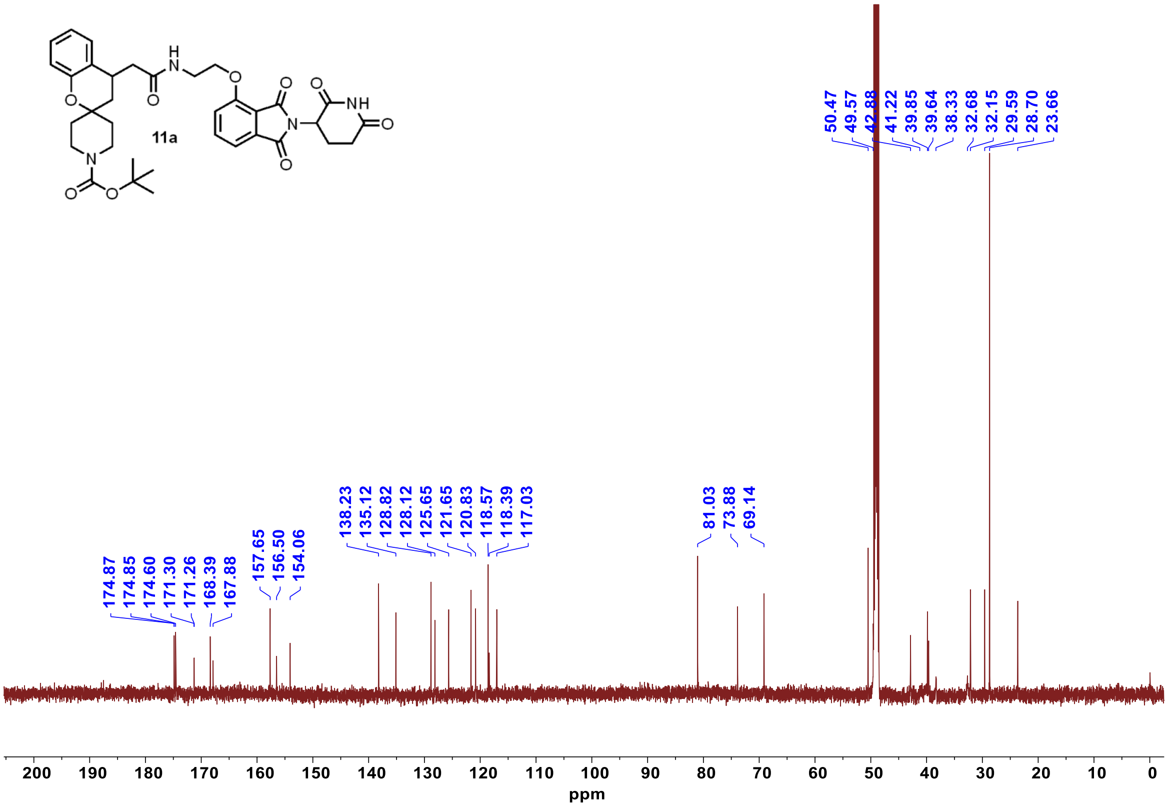

^{1}H NMR **11b**

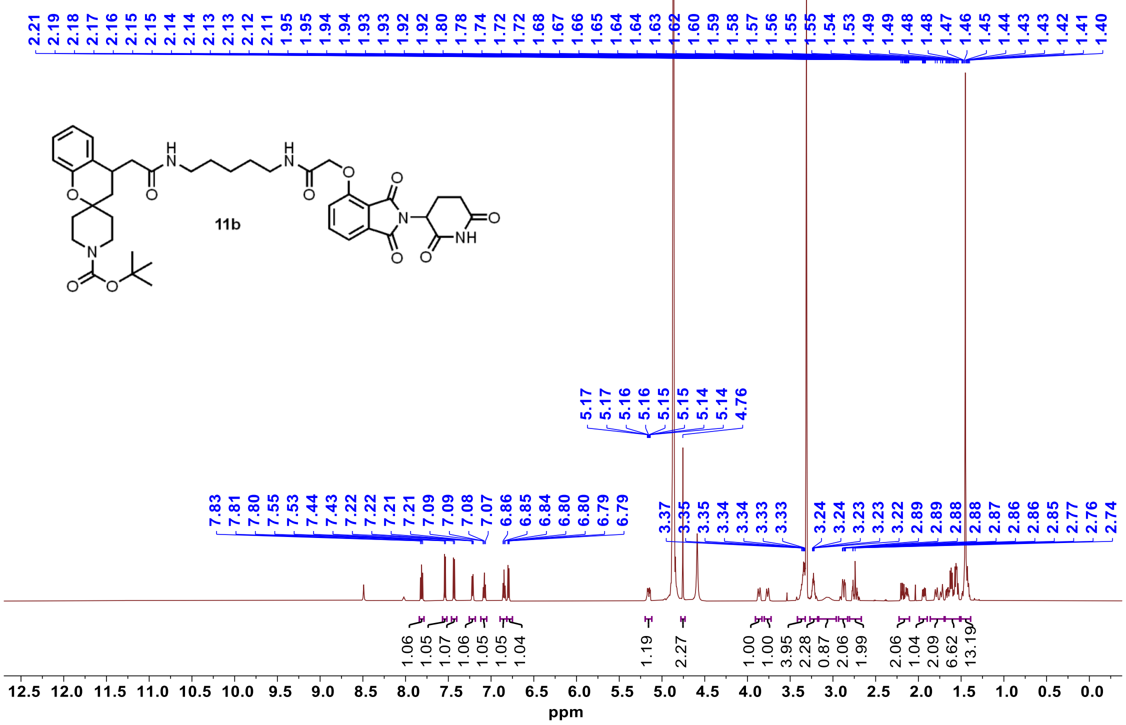

^{13}C NMR **11b**

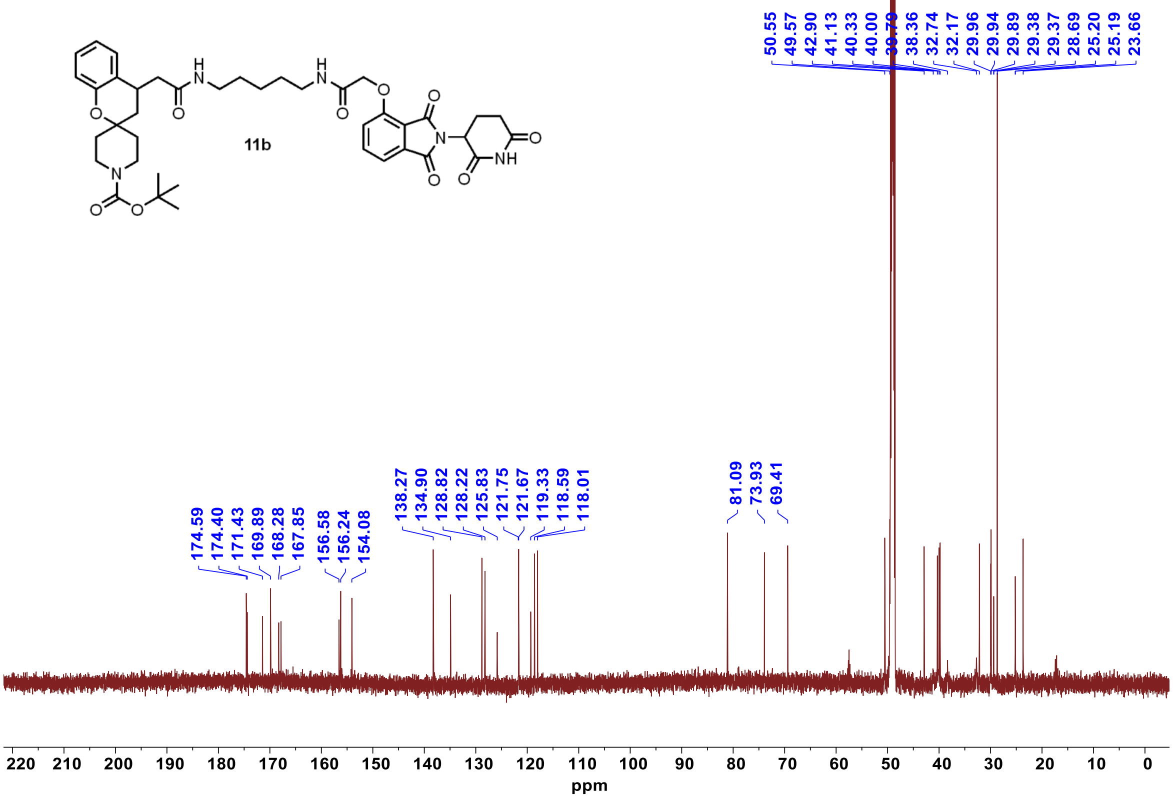

^{1}H NMR **11c**

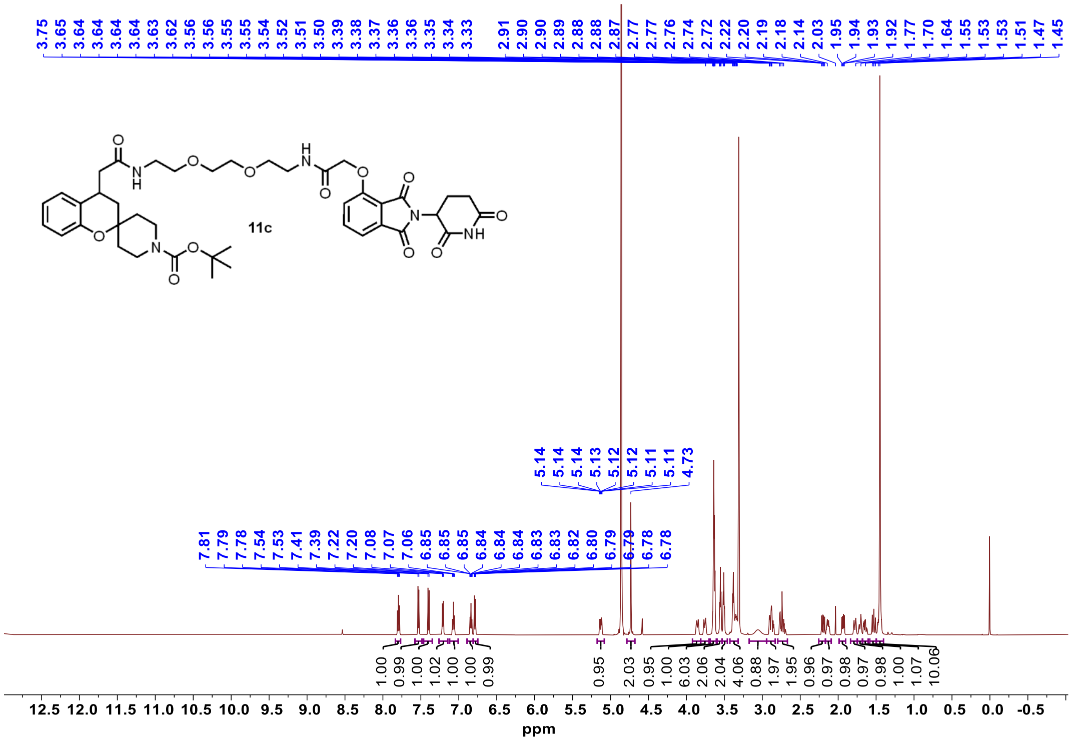

^{13}C NMR **11c**

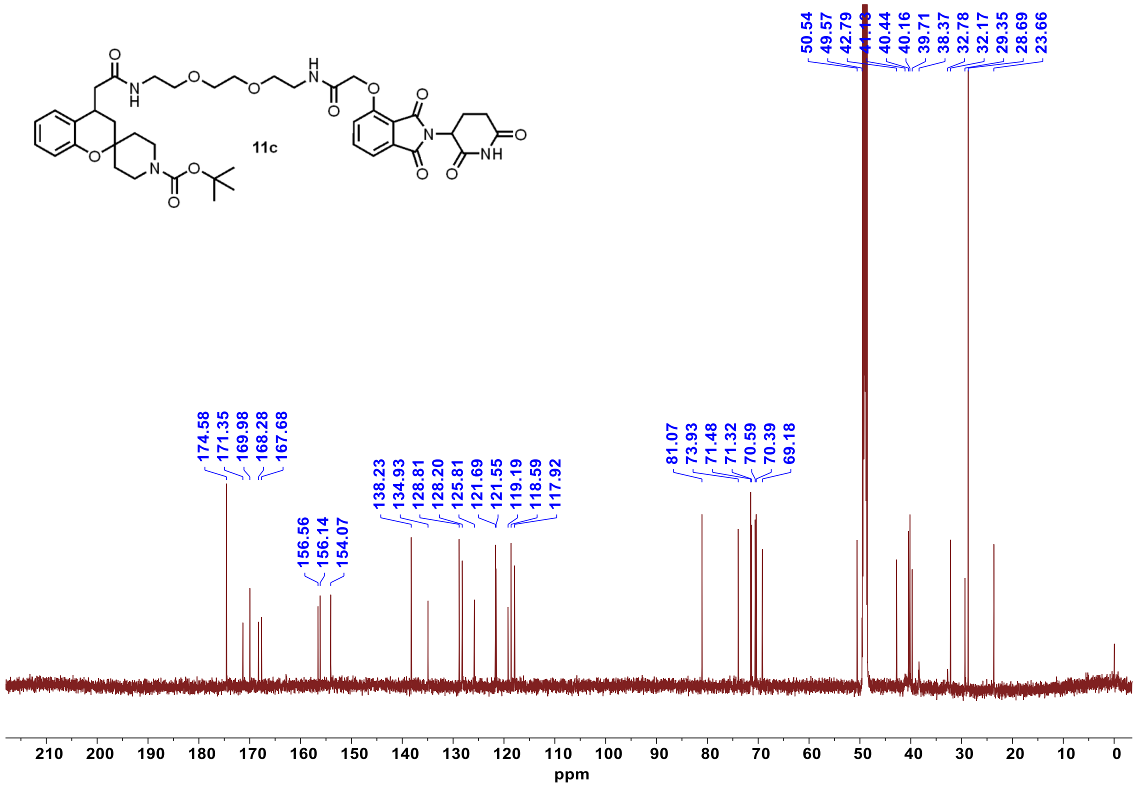

^{1}H NMR **11d**

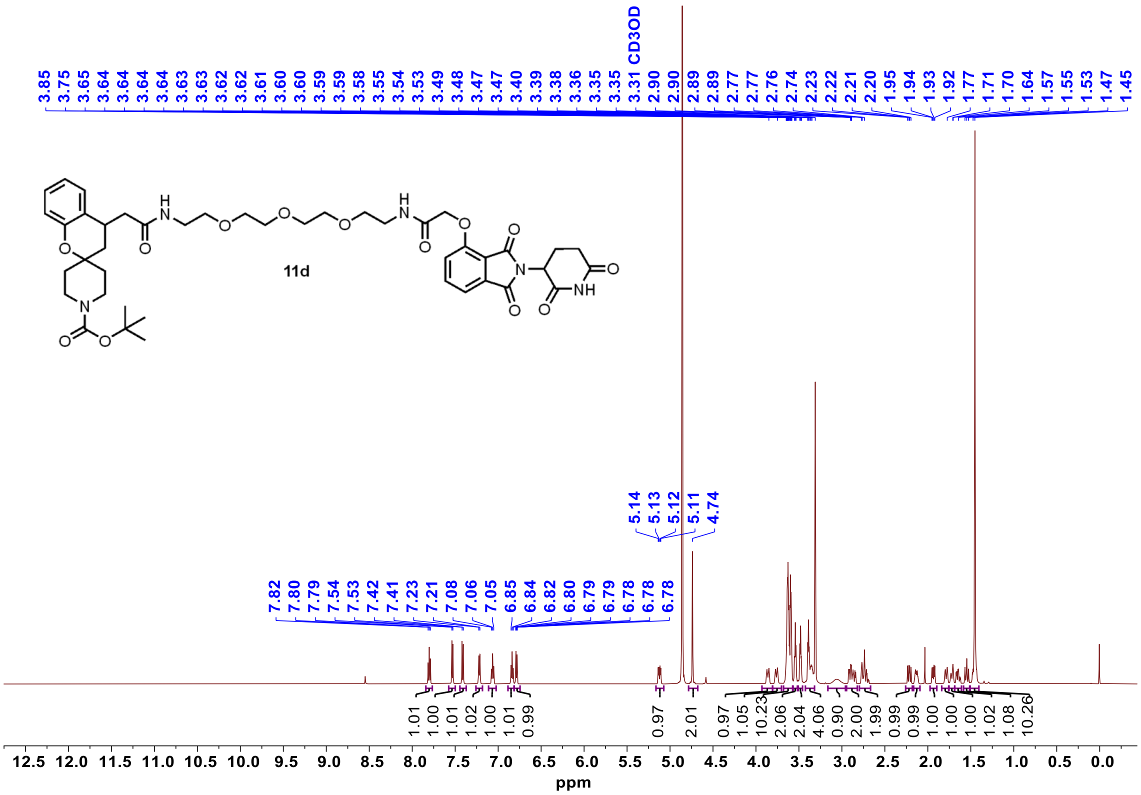

^{13}C NMR **11d**

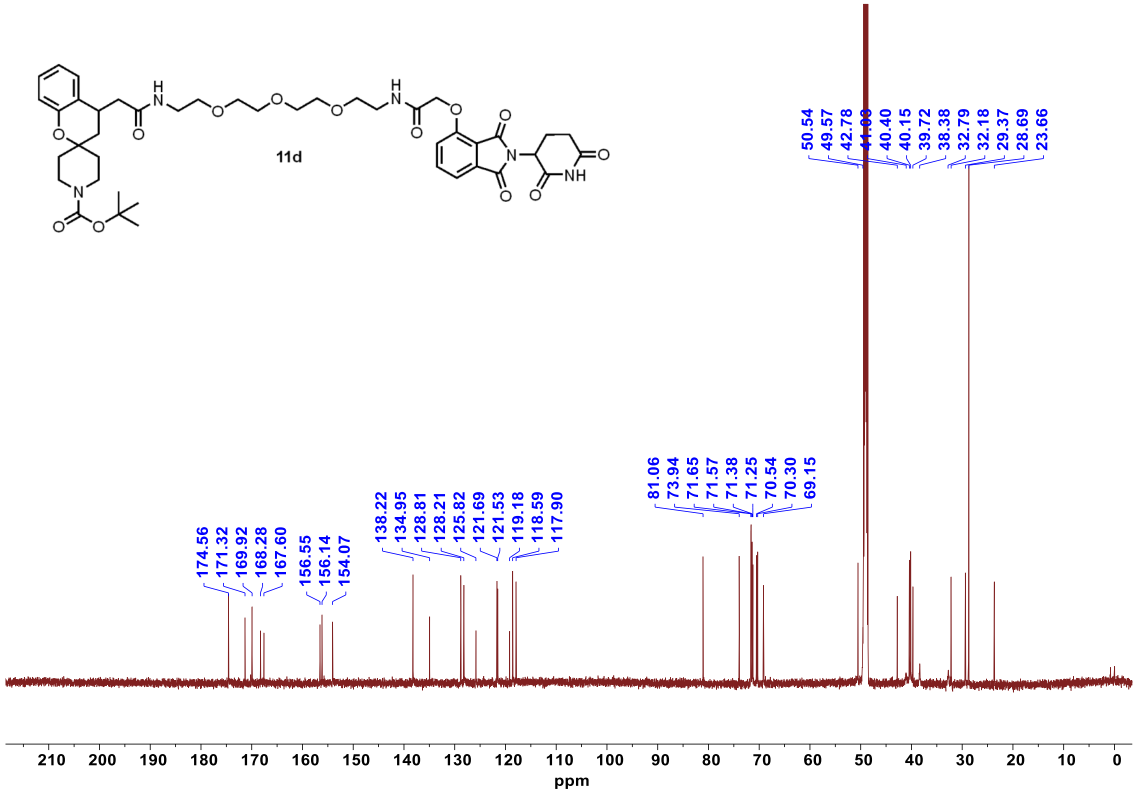

^{1}H NMR **12a**

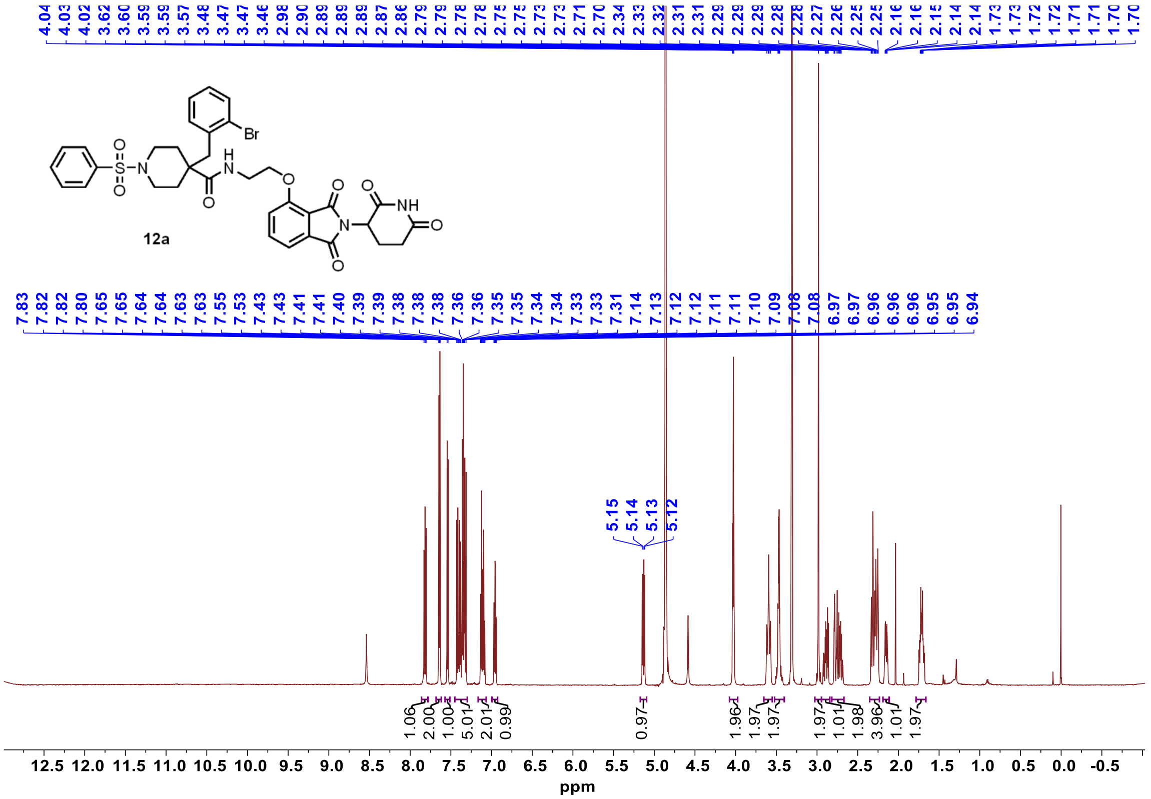

^{13}C NMR **12a**

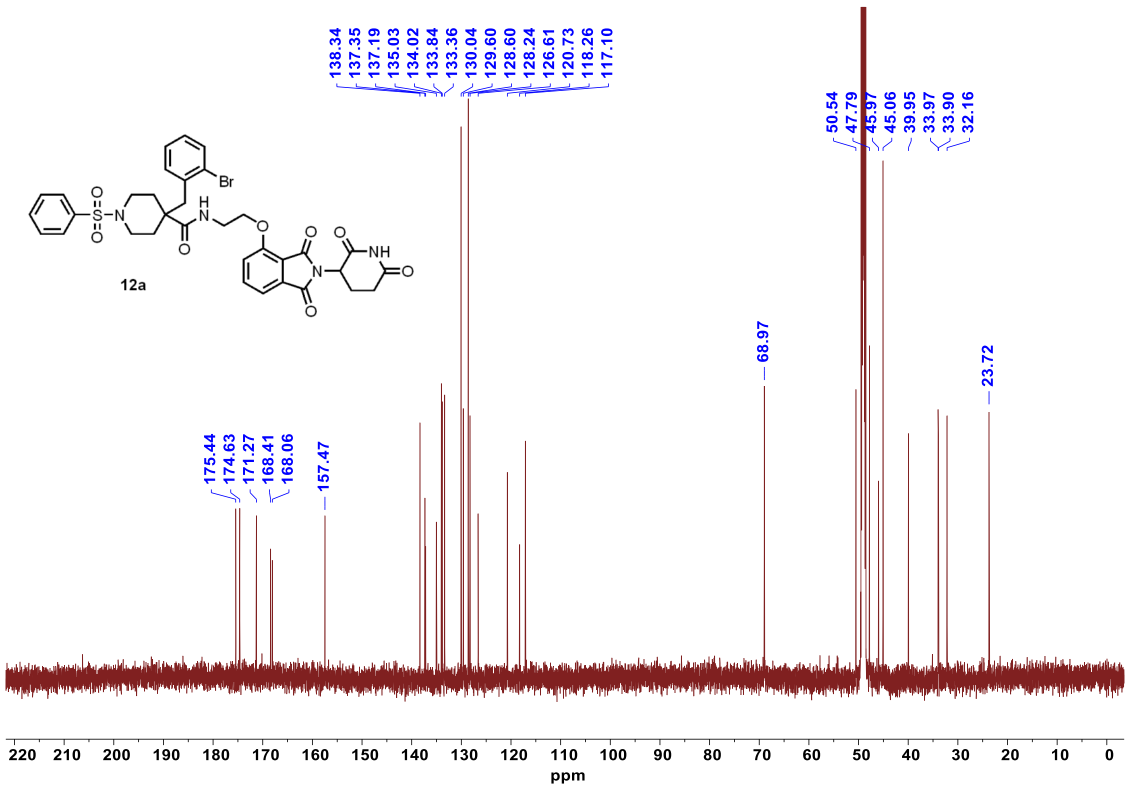

^{1}H NMR **12b**

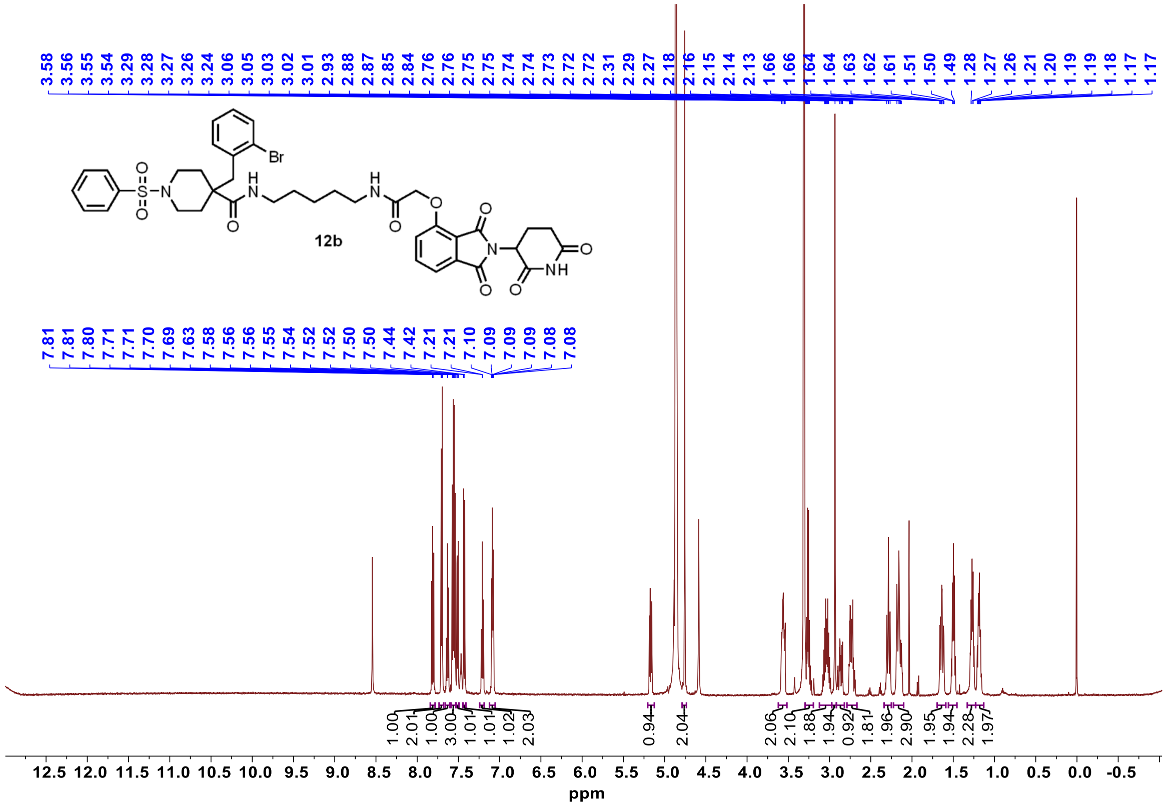

^{13}C NMR **12b**

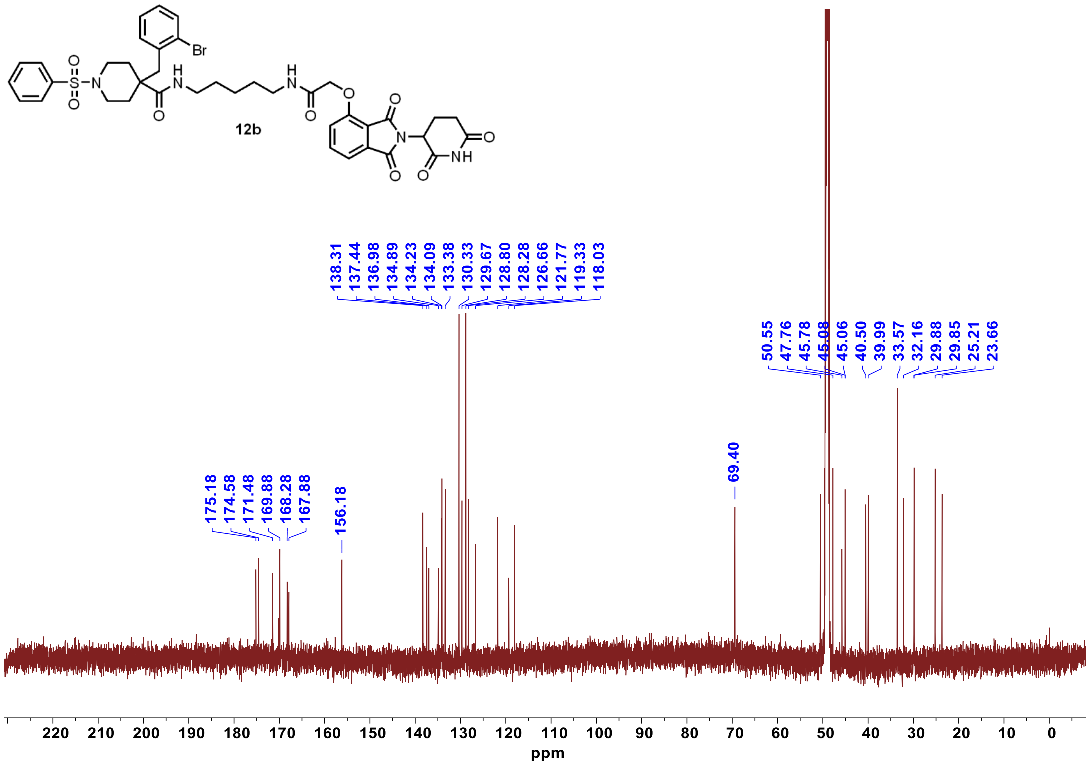

^{1}H NMR **12c**

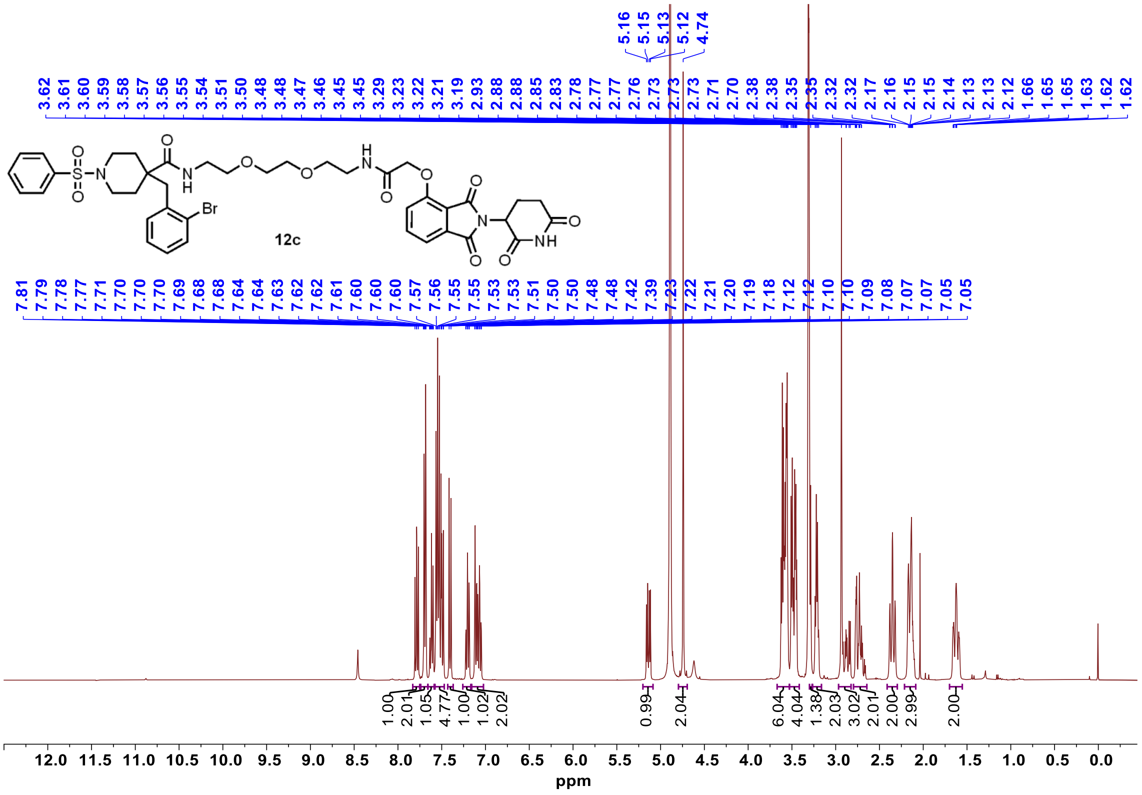

^{13}C NMR **12c**

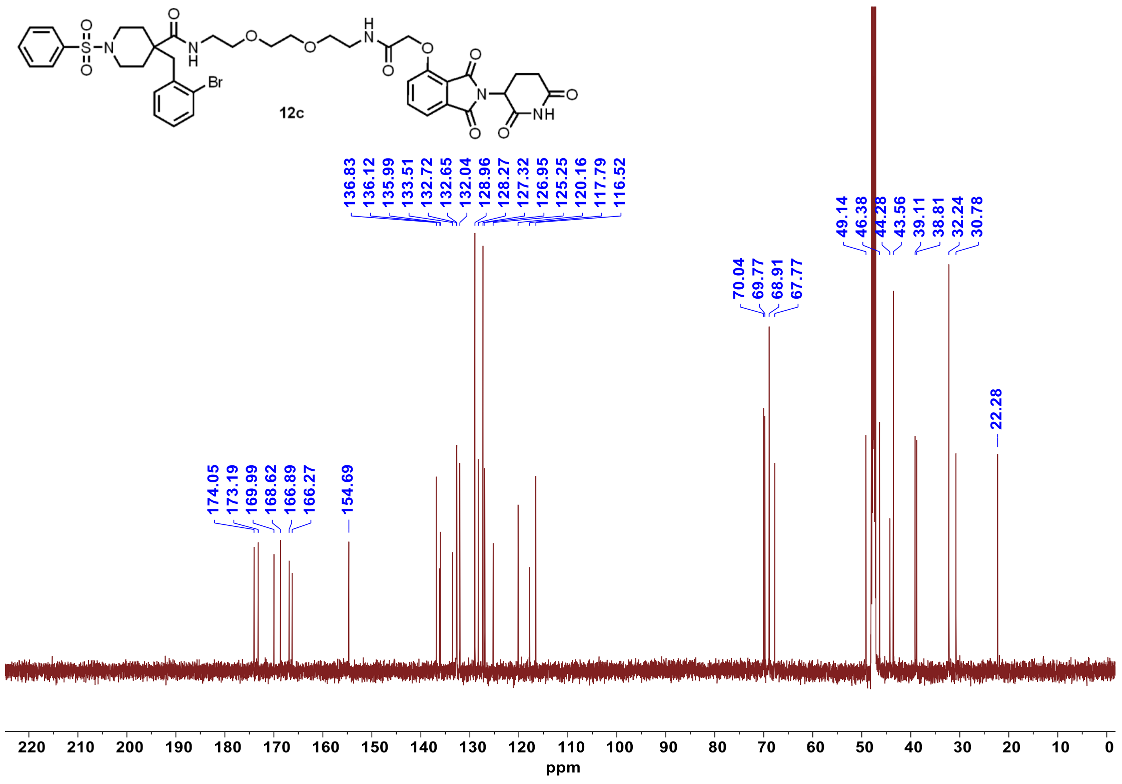

^{1}H NMR **12d**

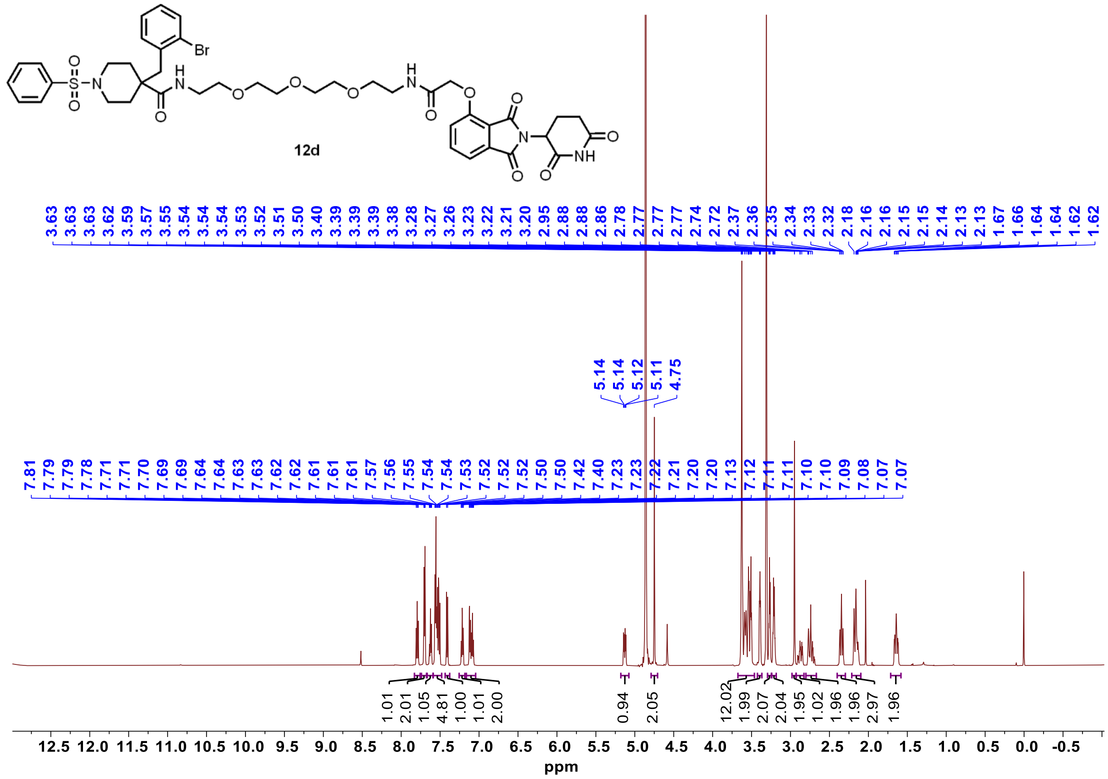

^{13}C NMR **12d**

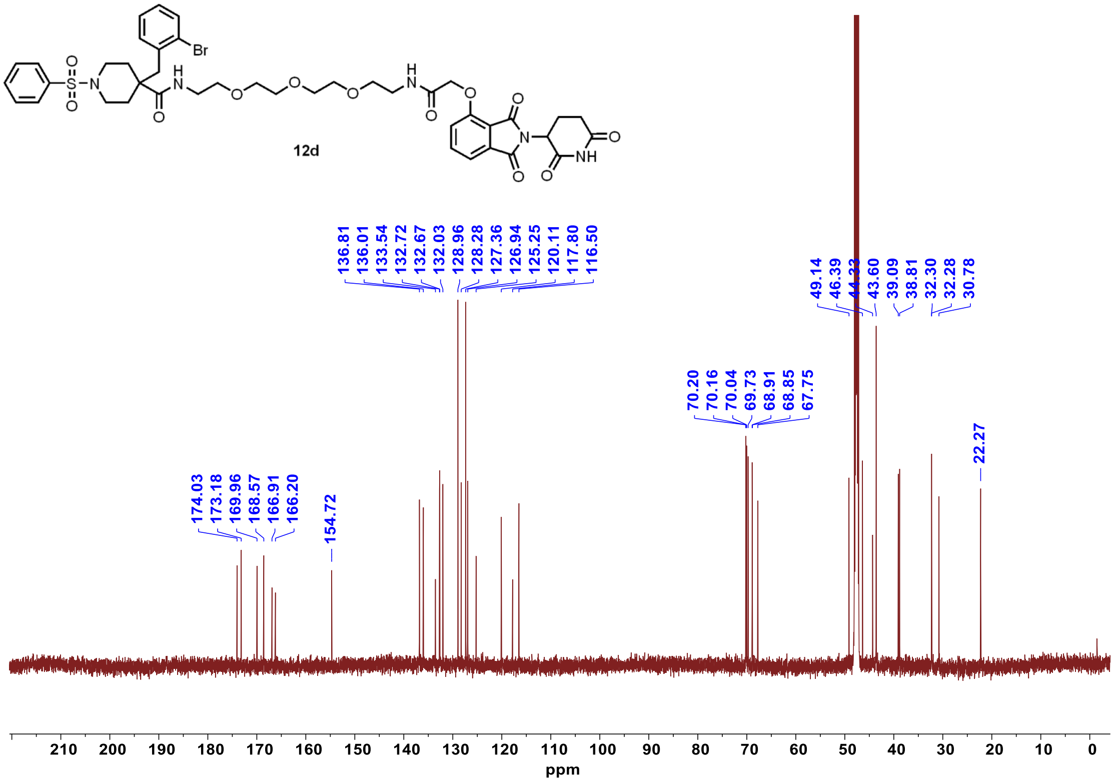

^{1}H NMR **13a**

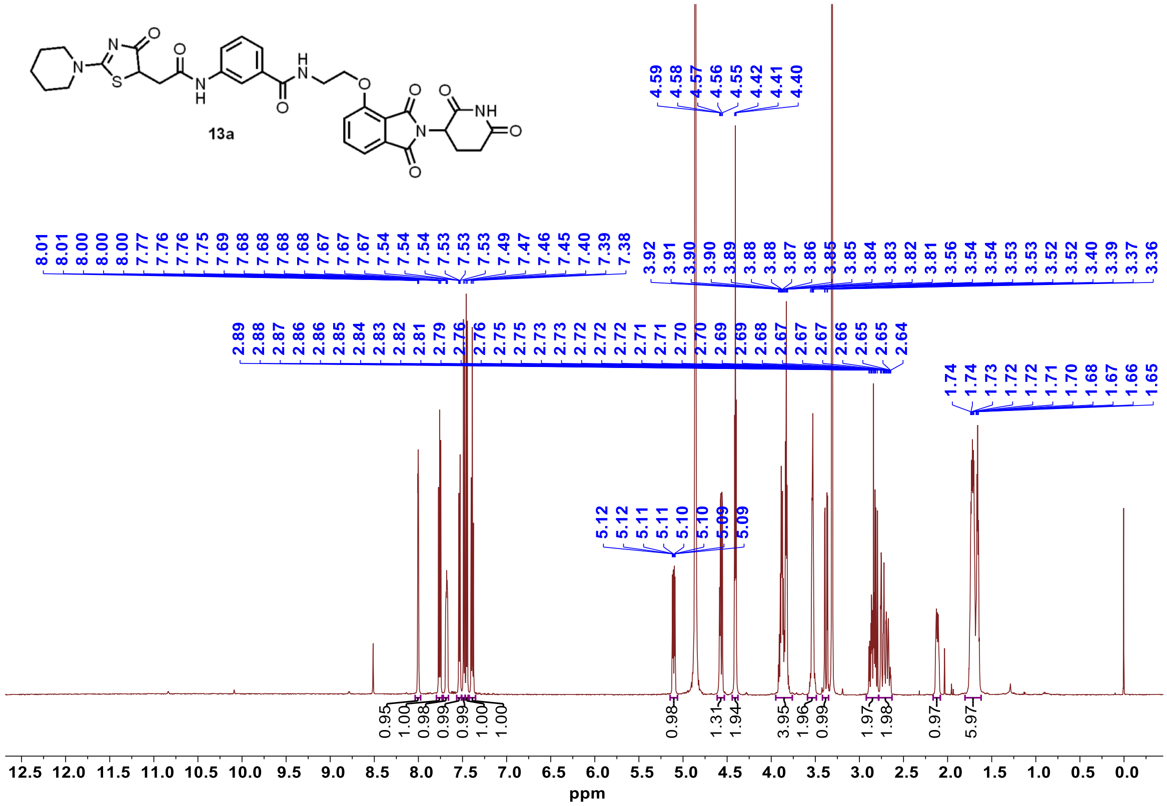

^{13}C NMR **13a**

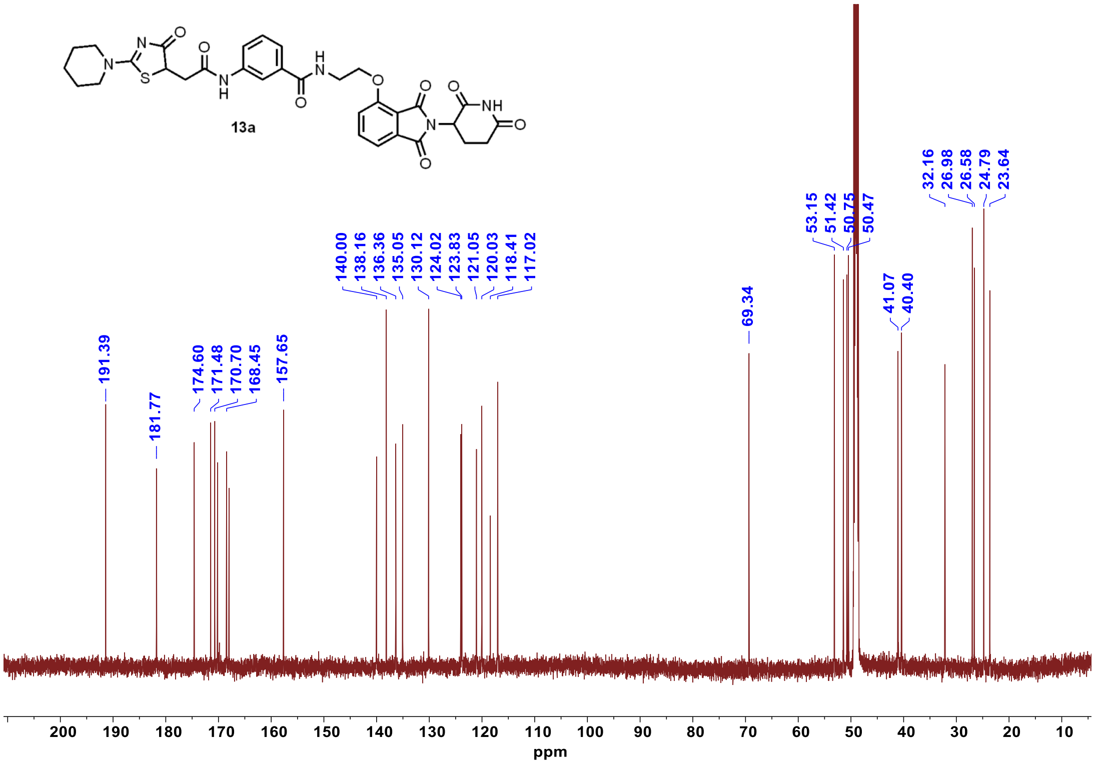

^{1}H NMR **13b**

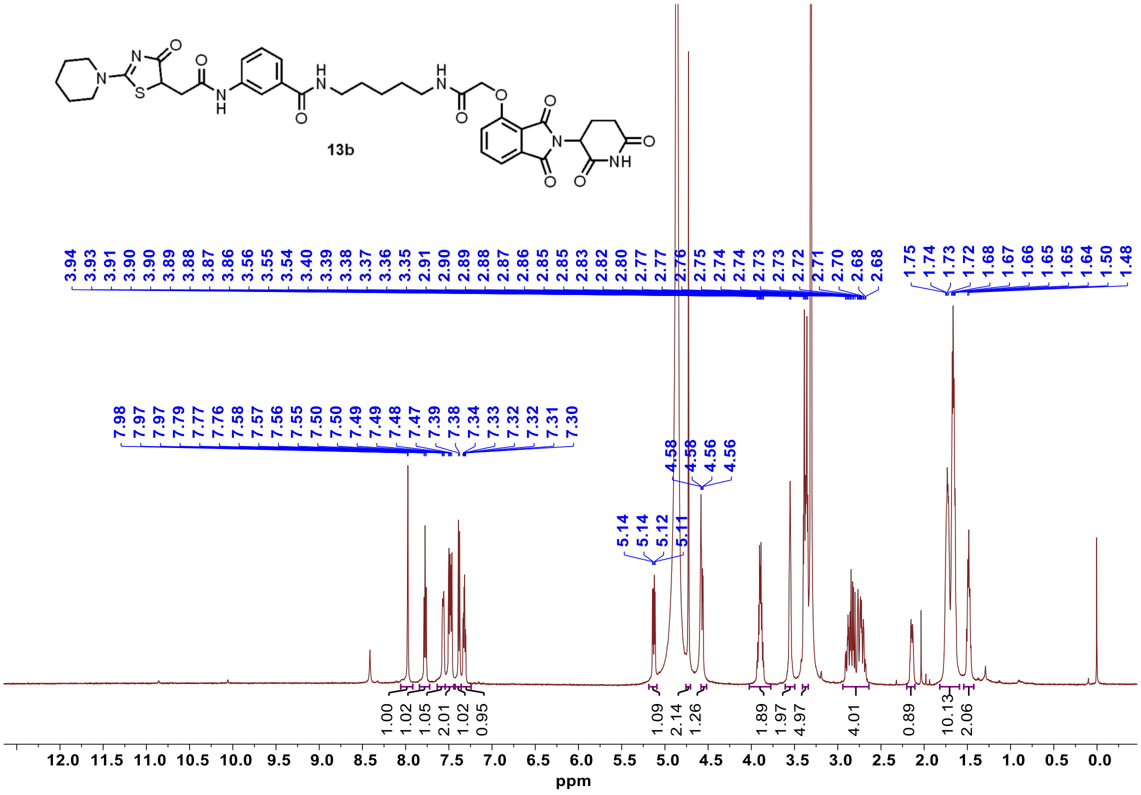

^{13}C NMR **13b**

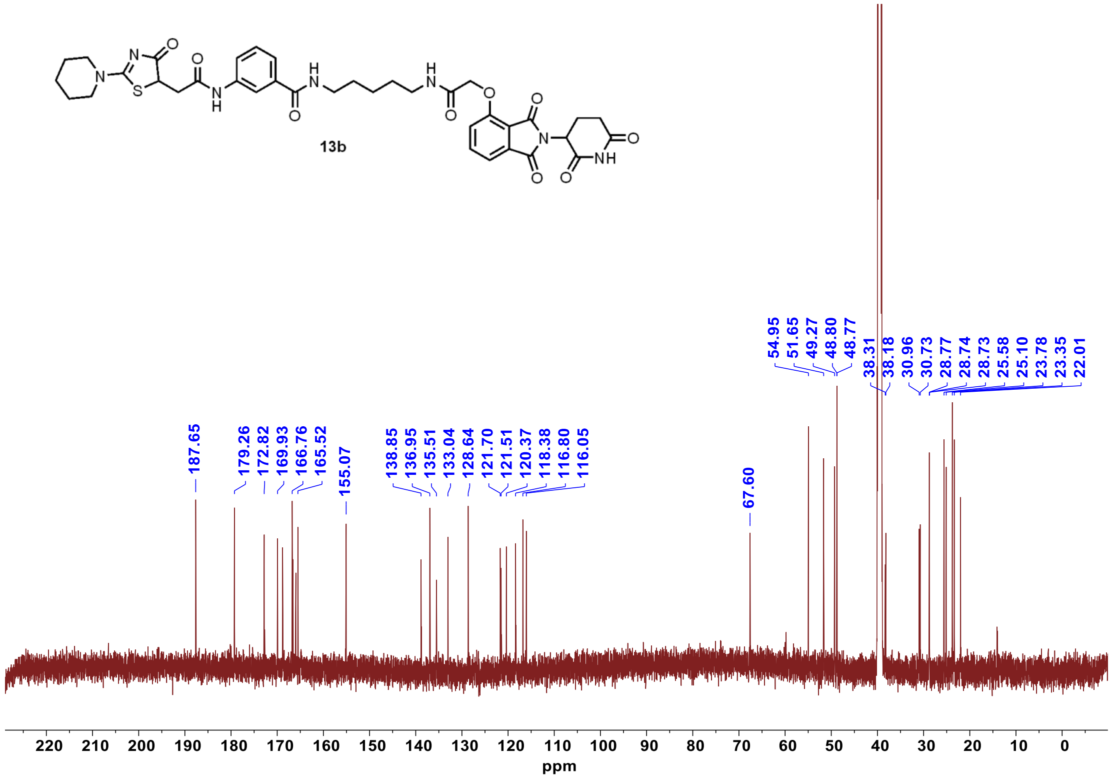

^{1}H NMR **13c**

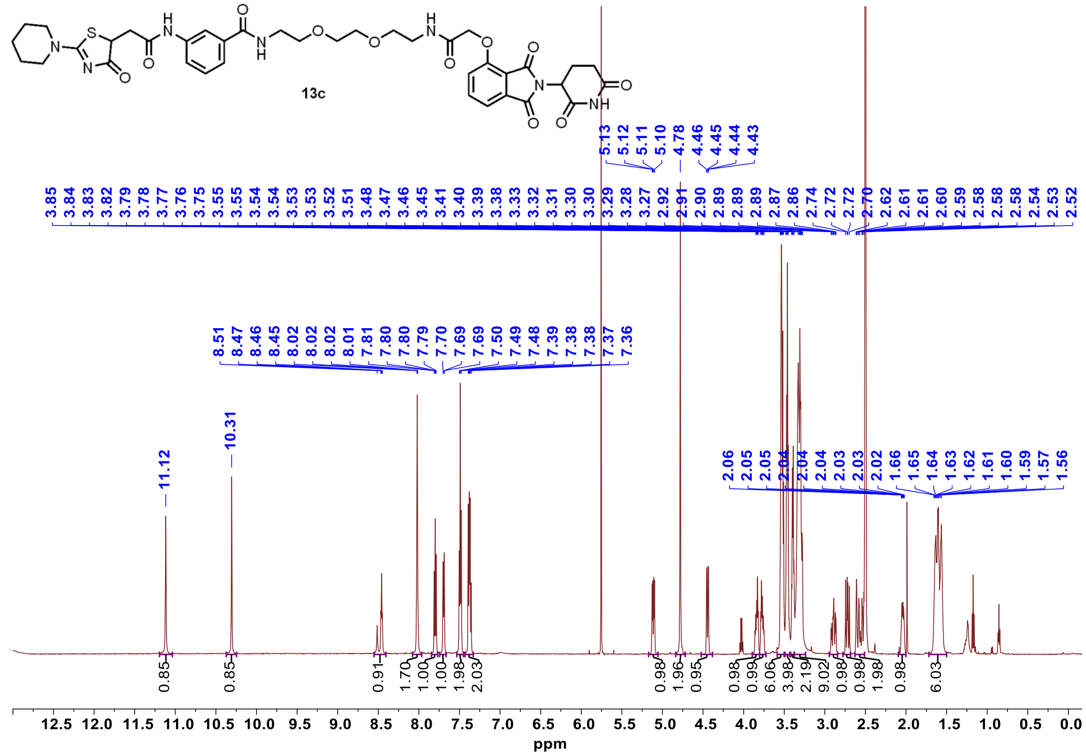

^{13}C NMR **13c**

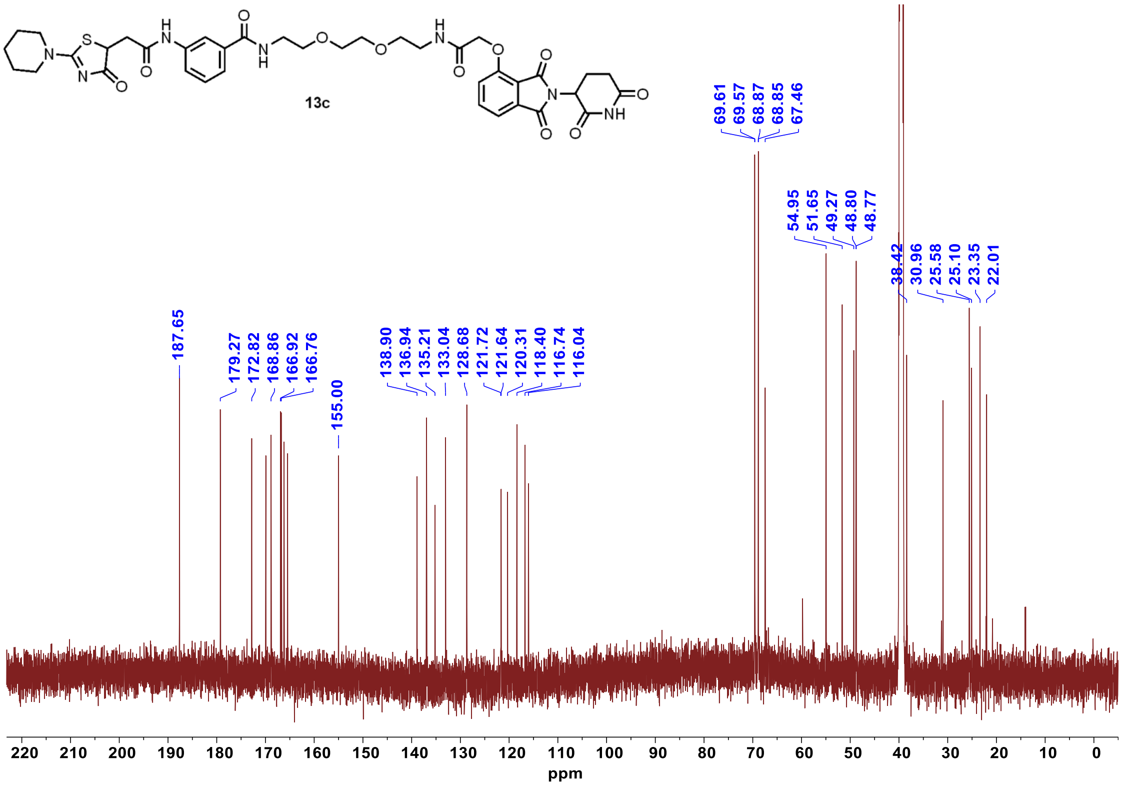

^{1}H NMR **13d**

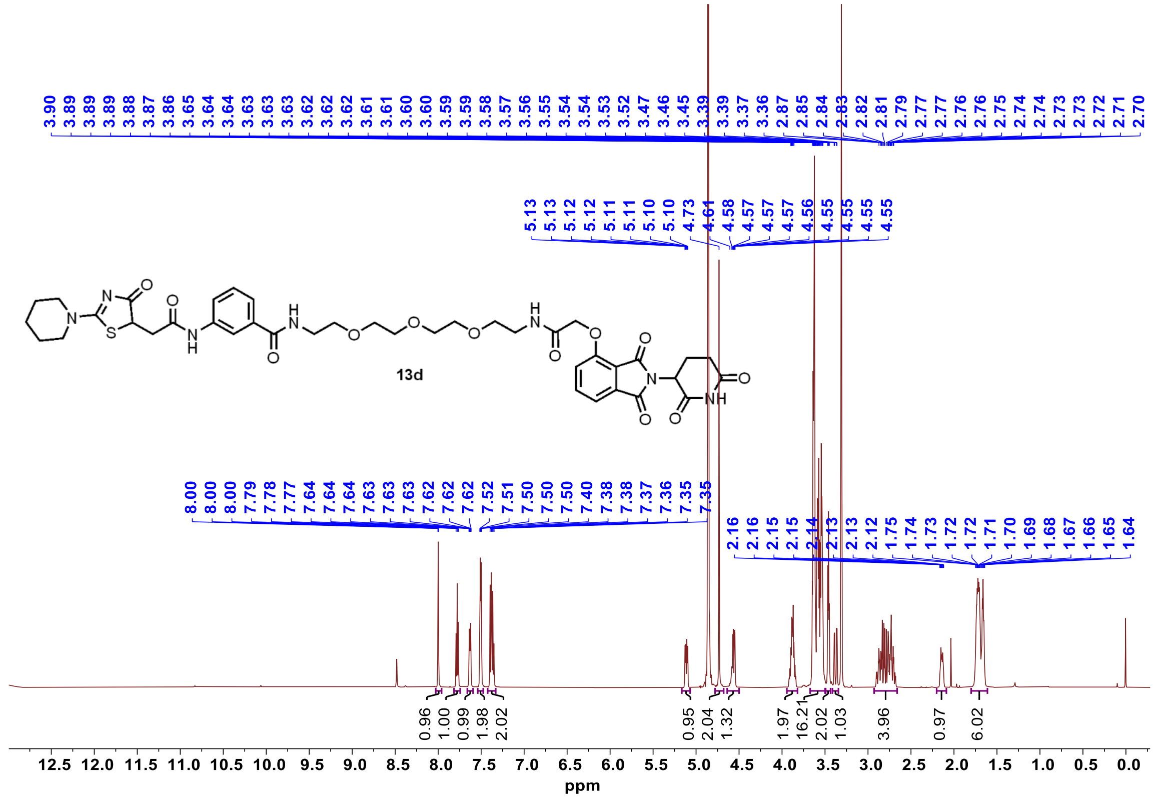

^{13}C NMR **13d**

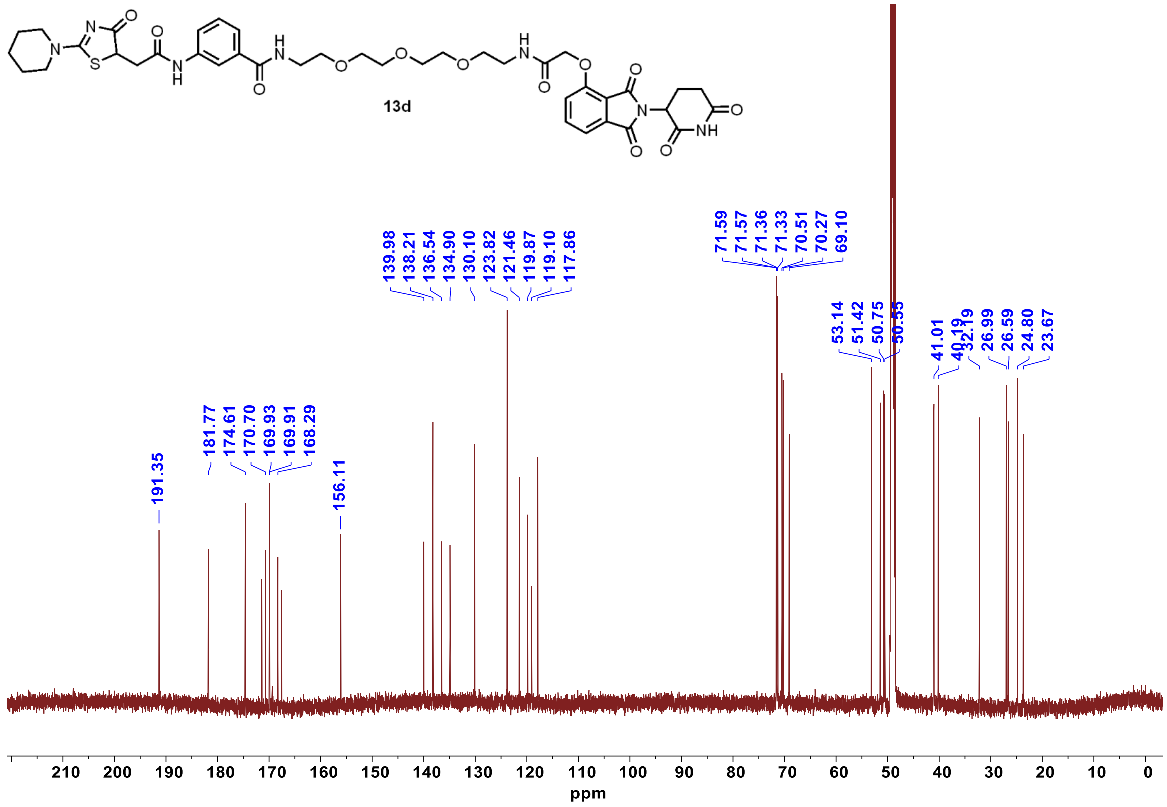

^{1}H NMR **14a**

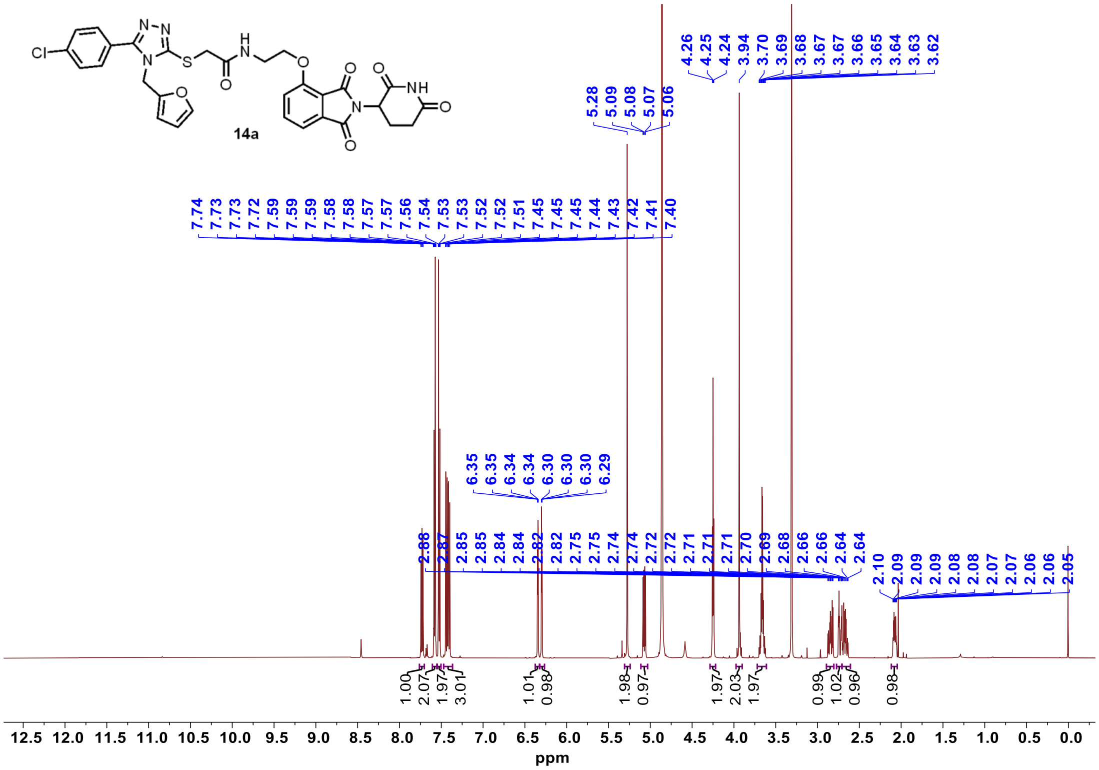

^{13}C NMR **14a**

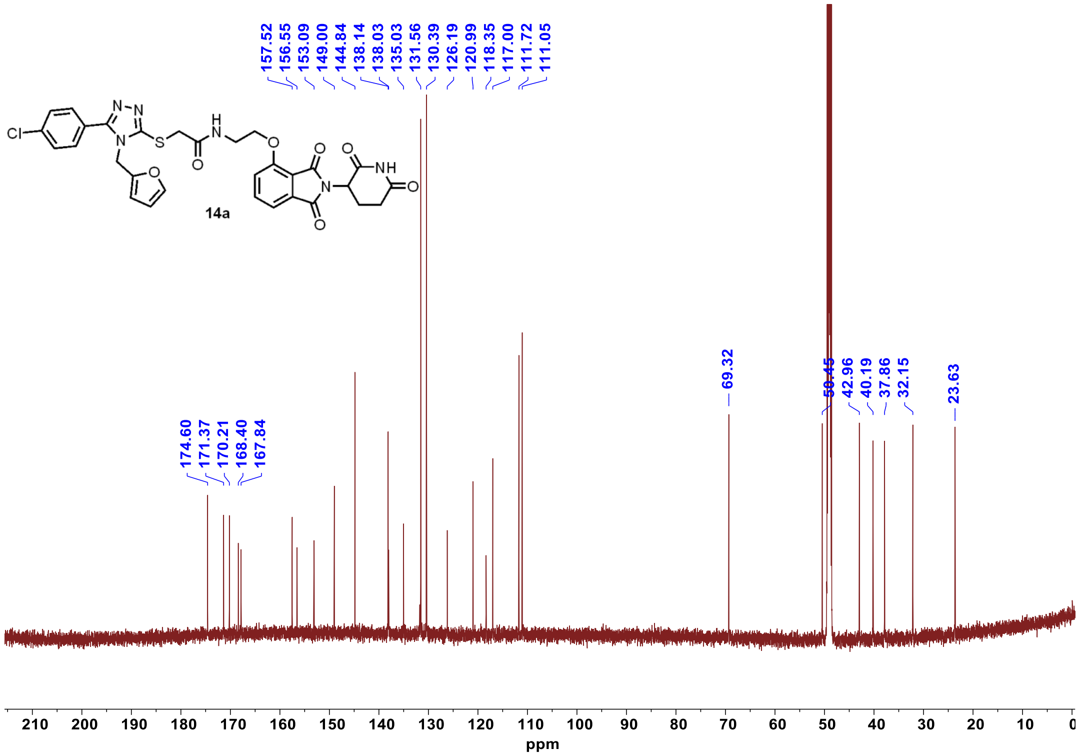

^{1}H NMR **14b**

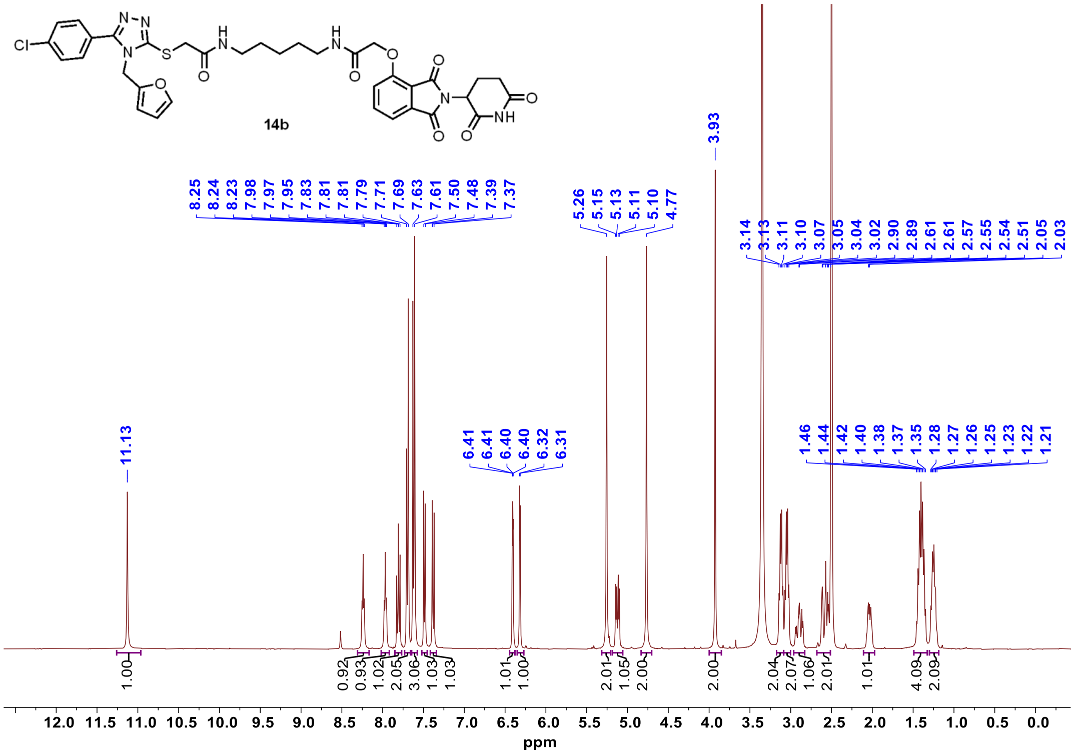

^{13}C NMR **14b**

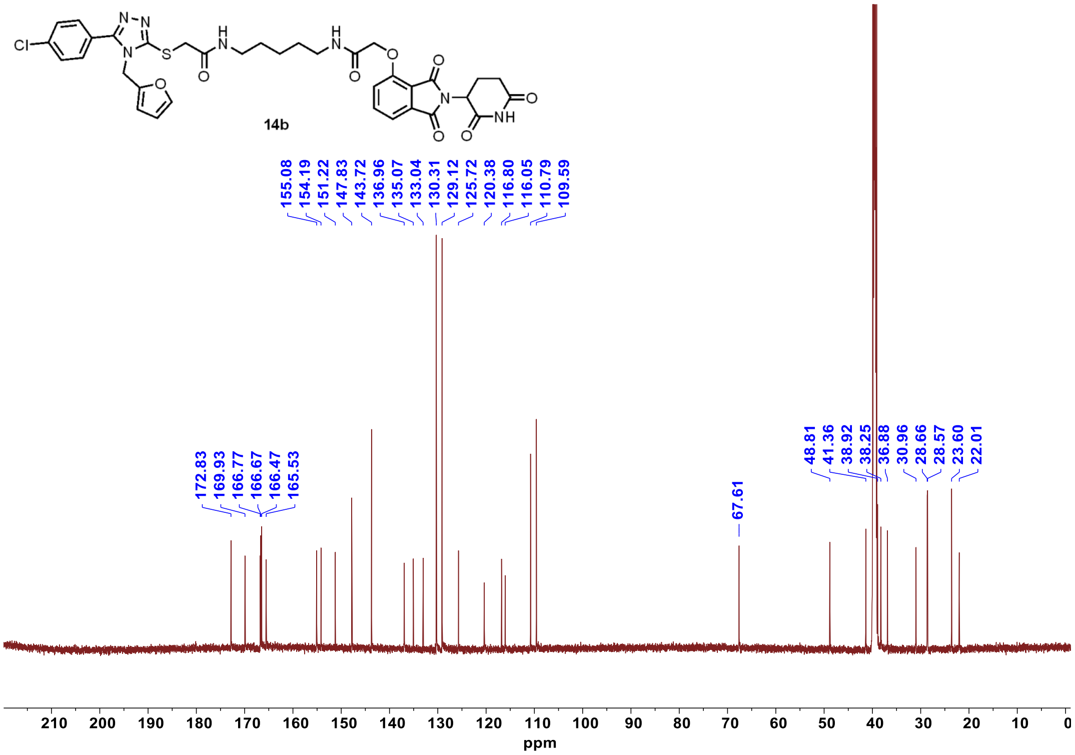

^{1}H NMR **14c**

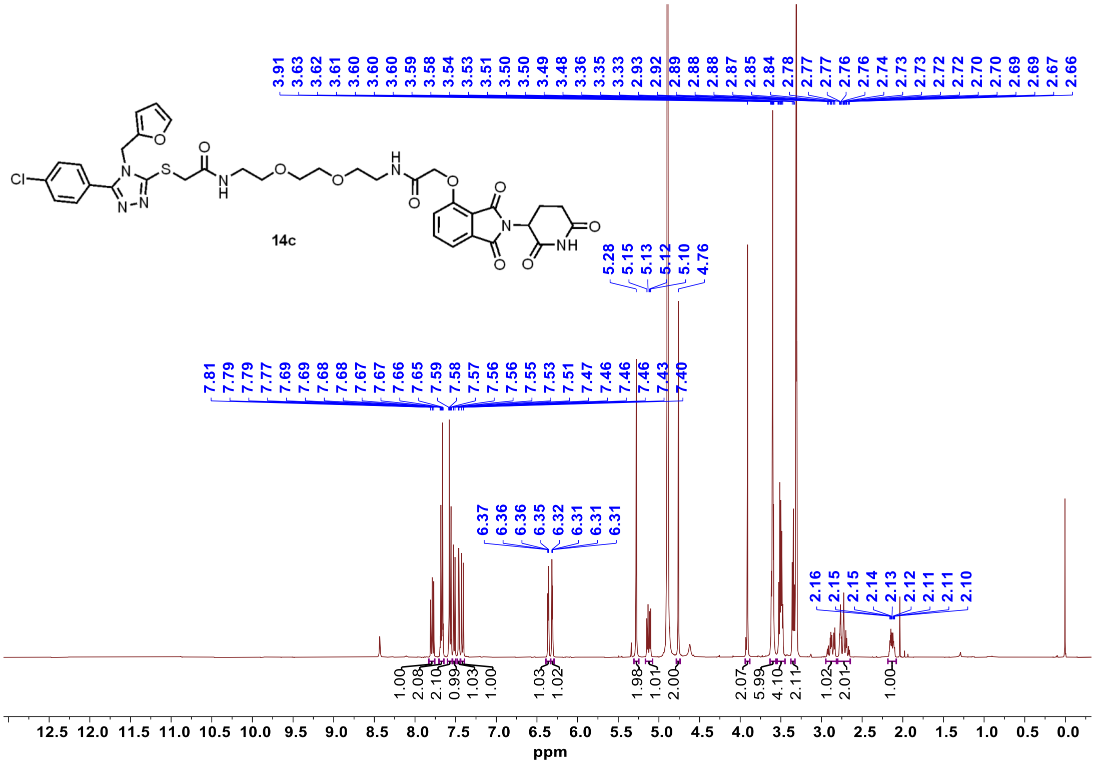

^{13}C NMR **14c**

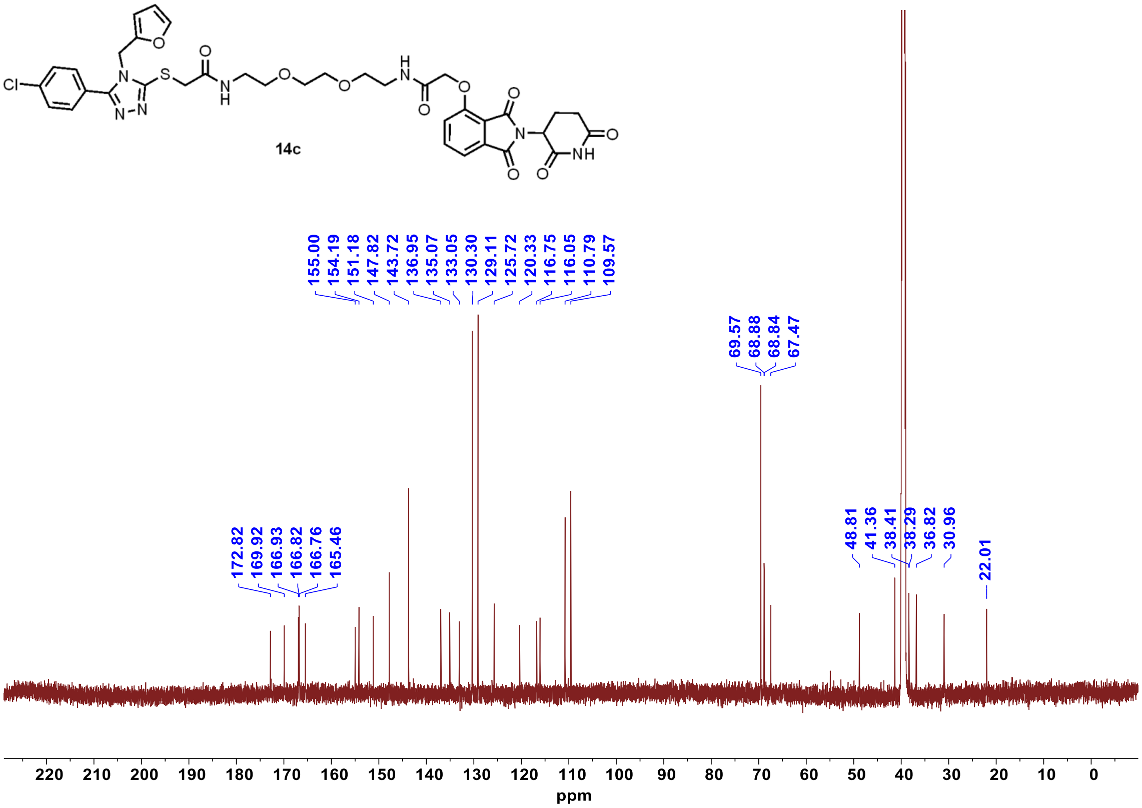

^{1}H NMR **14d**

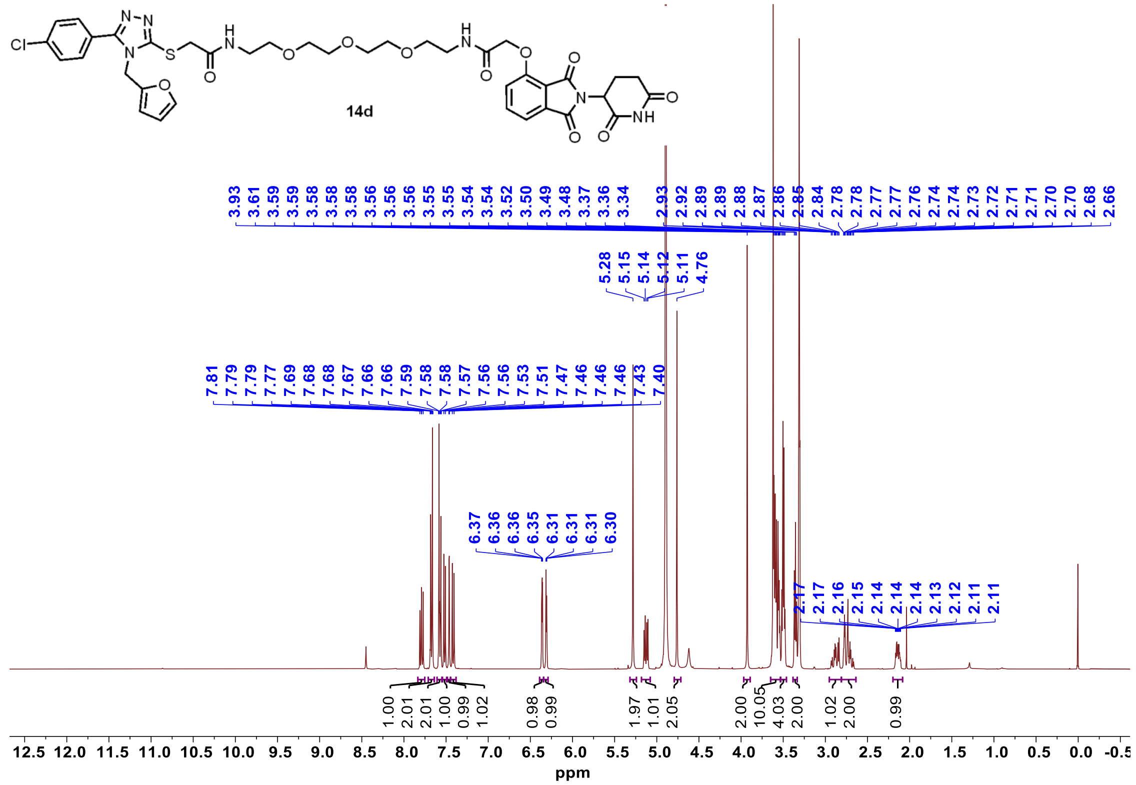

^{13}C NMR **14d**

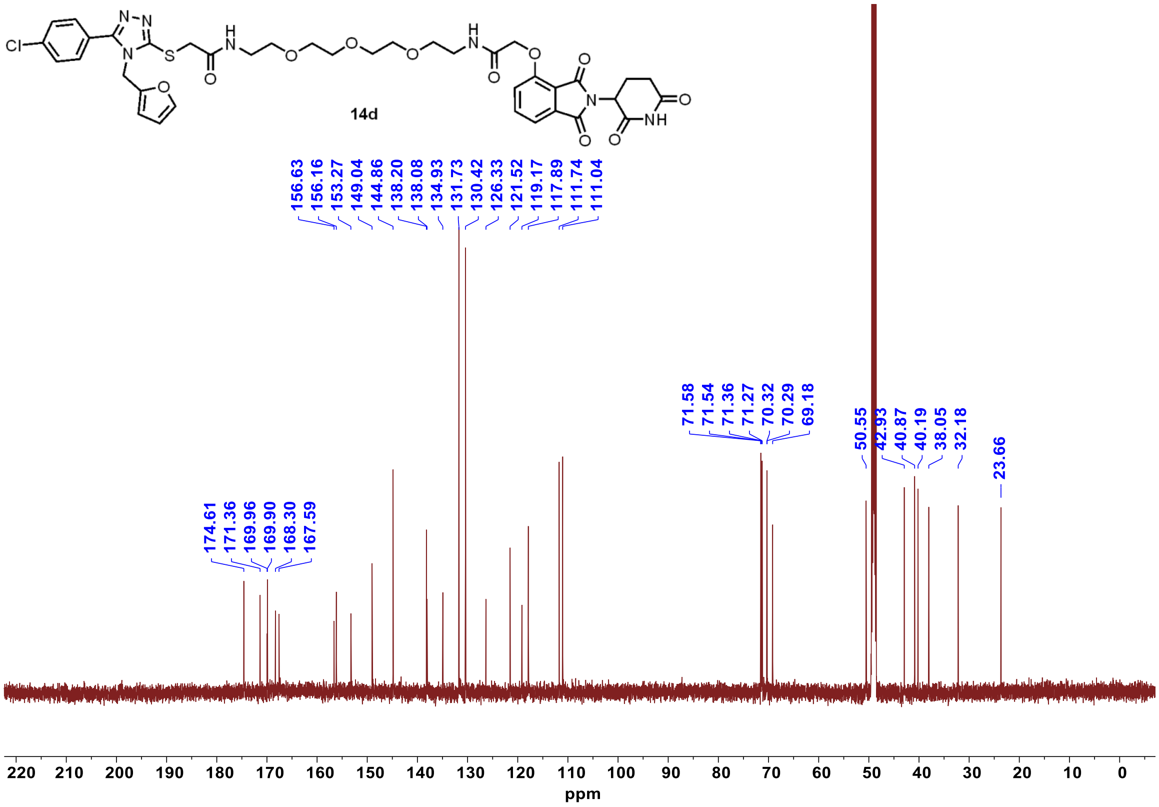

^{1}H NMR **15a**

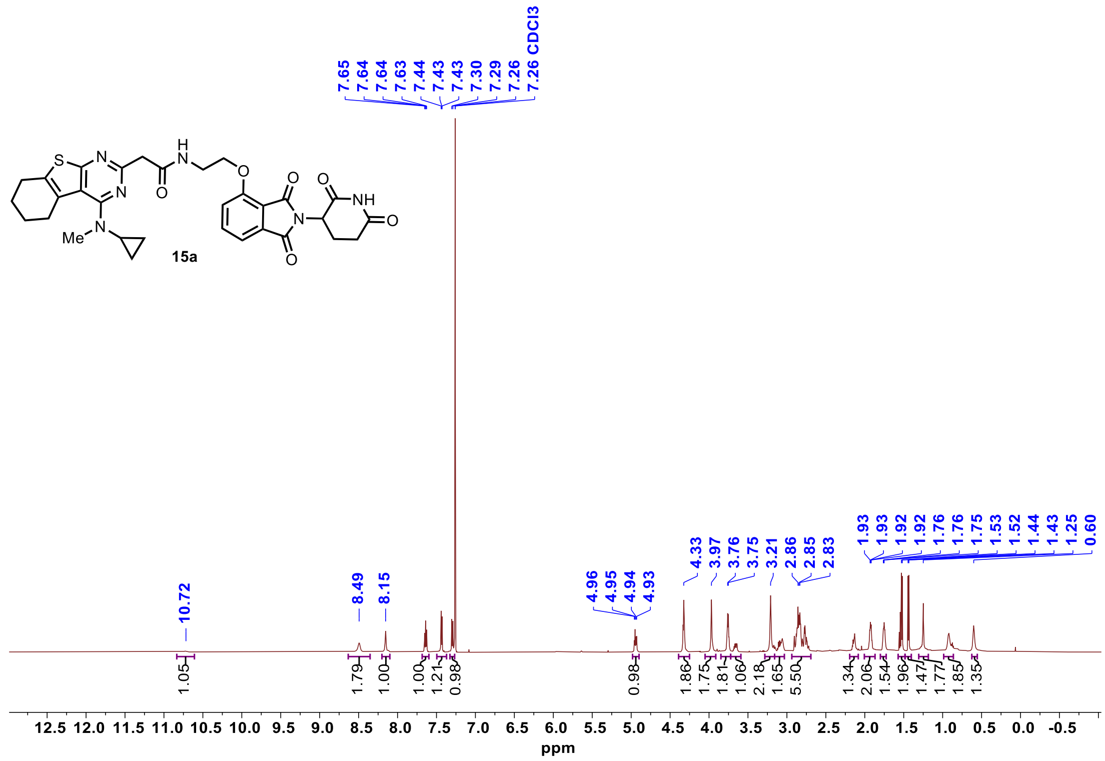

^{13}C NMR **15a**

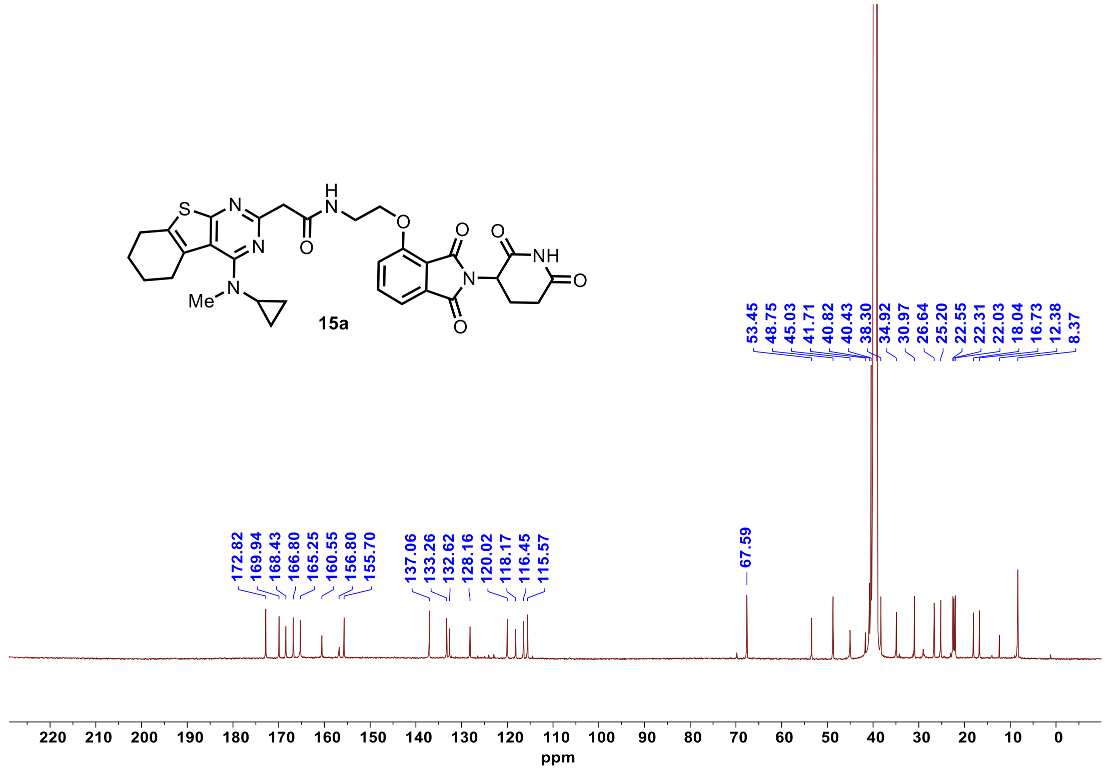

^{1}H NMR **15b**

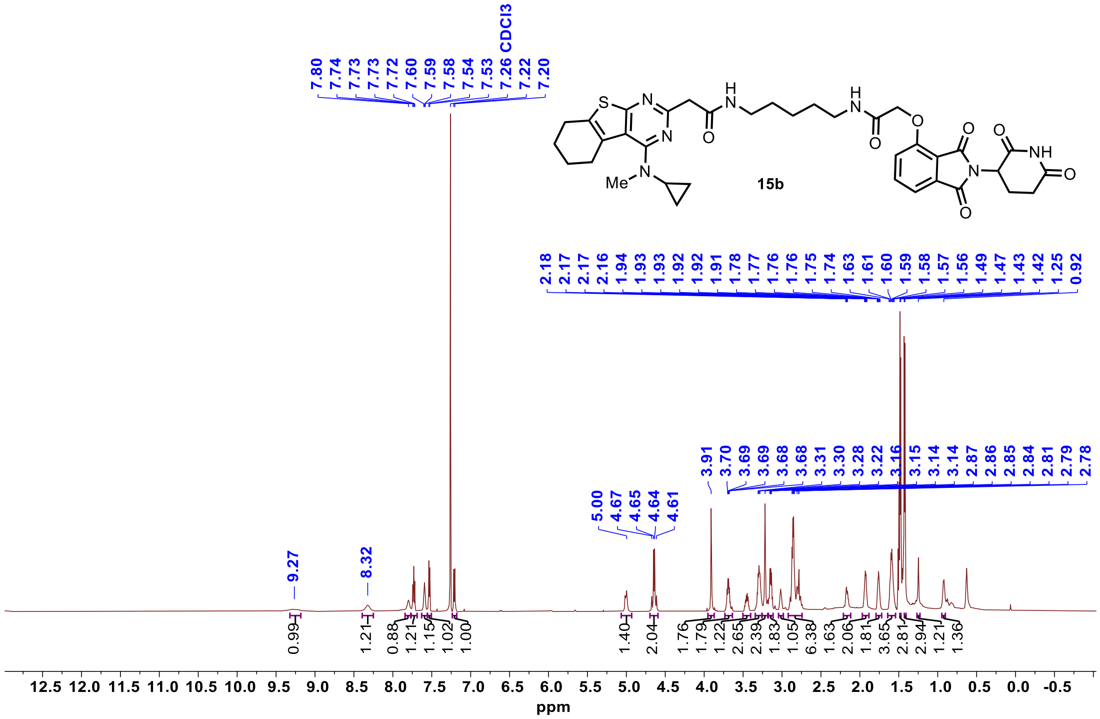

^{13}C NMR **15b**

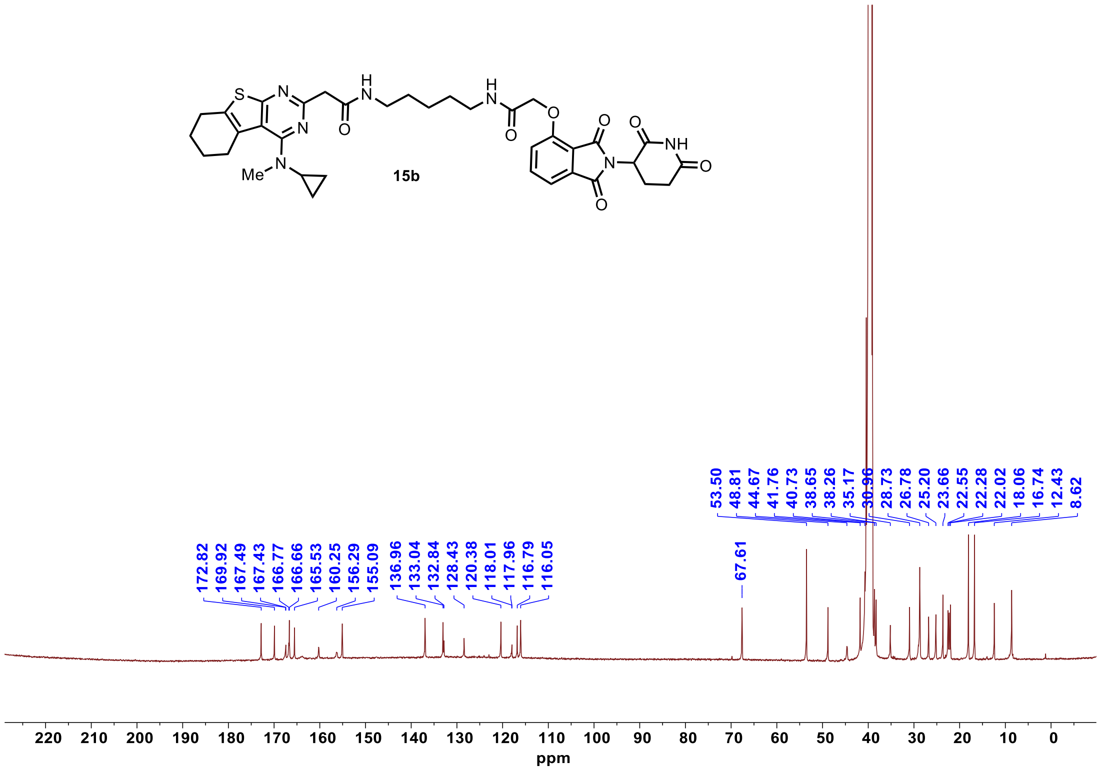

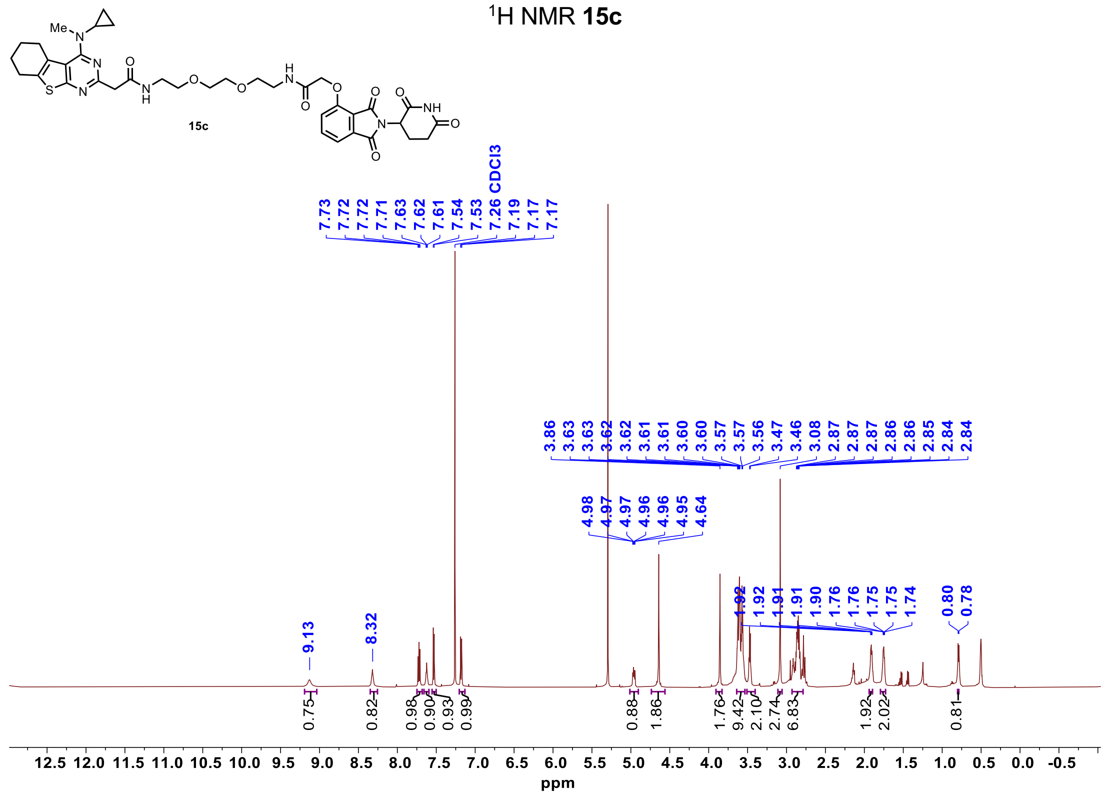

^{1}H NMR **15c**
Me
N
S
N
N
O
H
N
O
O
H
N
O
O
O
N
O
O
NH
O
15c
7.73
7.72
7.72
7.71
7.63
7.62
7.61
7.54
7.53
7.26 CDCl3
7.19
7.17
7.17
9.13
8.32
3.86
3.63
3.62
3.62
3.61
3.61
3.60
3.60
3.57
3.57
3.56
3.47
3.46
3.08
2.87
2.87
2.87
2.86
2.86
2.85
2.84
2.84
4.98
4.97
4.97
4.96
4.96
4.95
4.64
1.92
1.92
1.91
1.91
1.90
1.76
1.76
1.75
1.75
1.74
0.80
0.78
0.75
0.82
0.98
0.90
0.93
0.99
0.88
1.86
1.76
9.42
2.10
2.74
6.83
1.92
2.02
0.81
12.5 12.0 11.5 11.0 10.5 10.0 9.5 9.0 8.5 8.0 7.5 7.0 6.5 6.0 5.5 5.0 4.5 4.0 3.5 3.0 2.5 2.0 1.5 1.0 0.5 0.0 -0.5
ppm

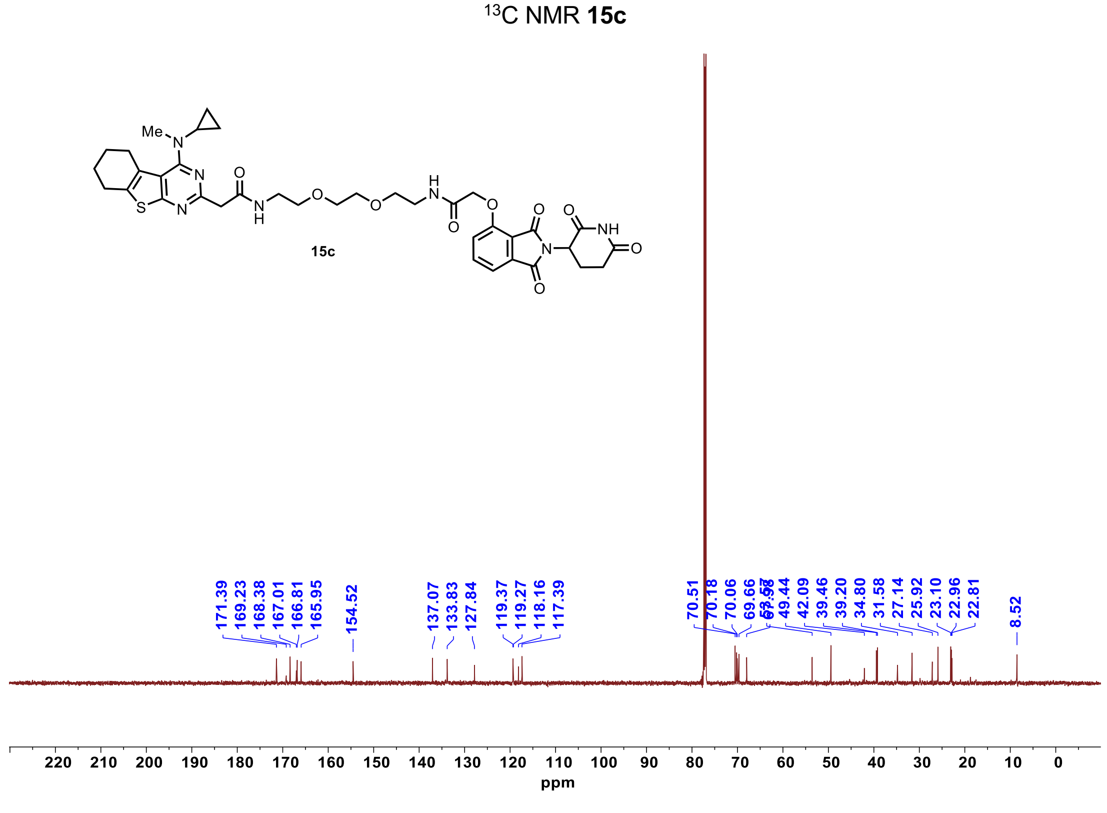

^{13}C NMR **15c**
Me
N
S
N
N
O
H
N
O
O
H
N
O
O
O
N
O
O
NH
O
15c
171.39
169.23
168.38
167.01
166.81
165.95
154.52
137.07
133.83
127.84
119.37
119.27
118.16
117.39
70.51
70.18
70.06
69.66
8.52
49.44
42.09
39.46
39.20
34.80
31.58
27.14
25.92
23.10
22.96
22.81
220 210 200 190 180 170 160 150 140 130 120 110 100 90 80 70 60 50 40 30 20 10 0
ppm

^{1}H NMR **15d**

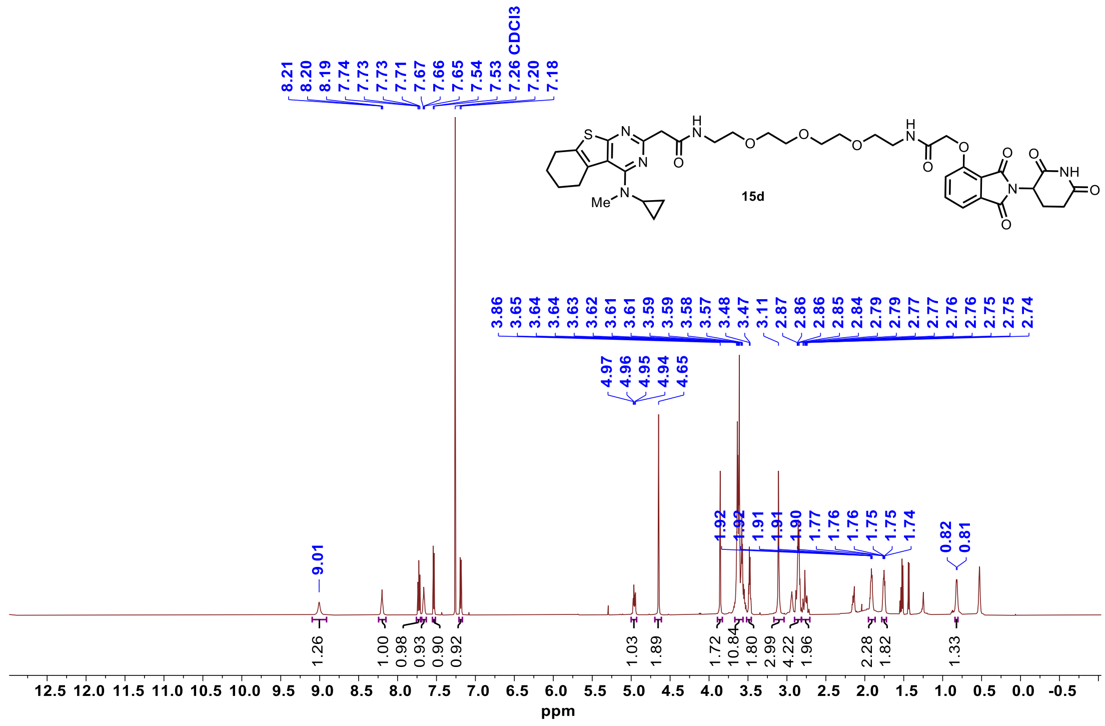

^{13}C NMR **15d**

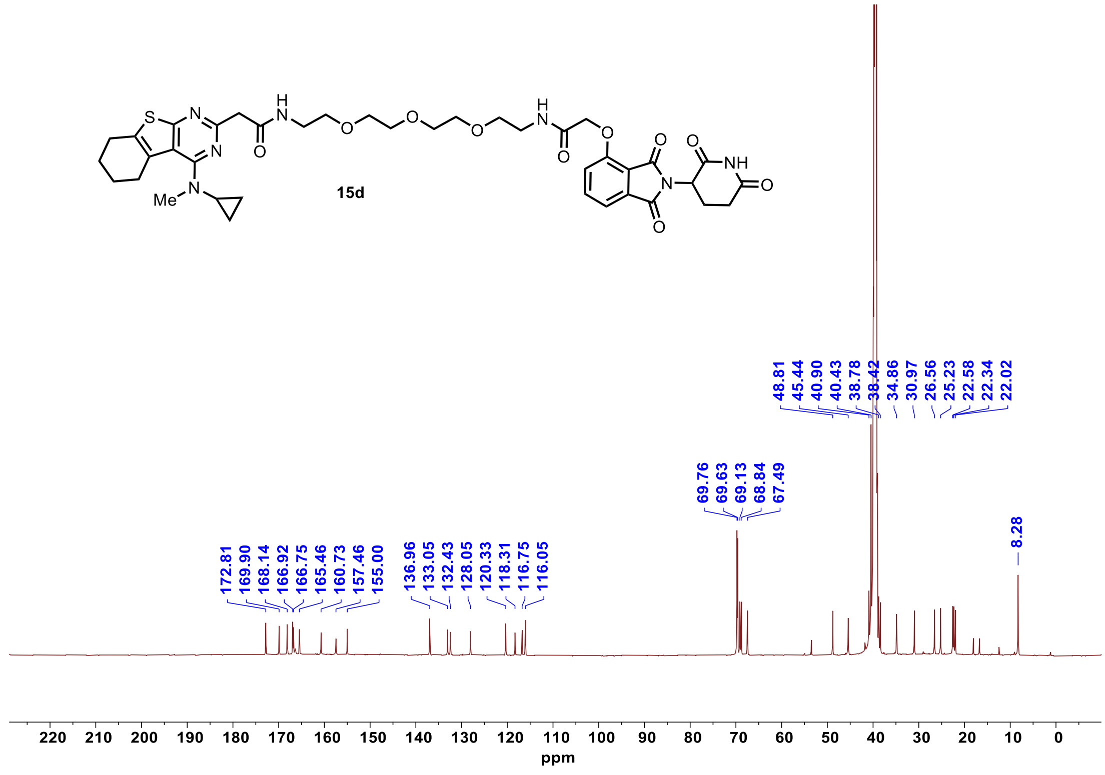

^{1}H NMR **16a**

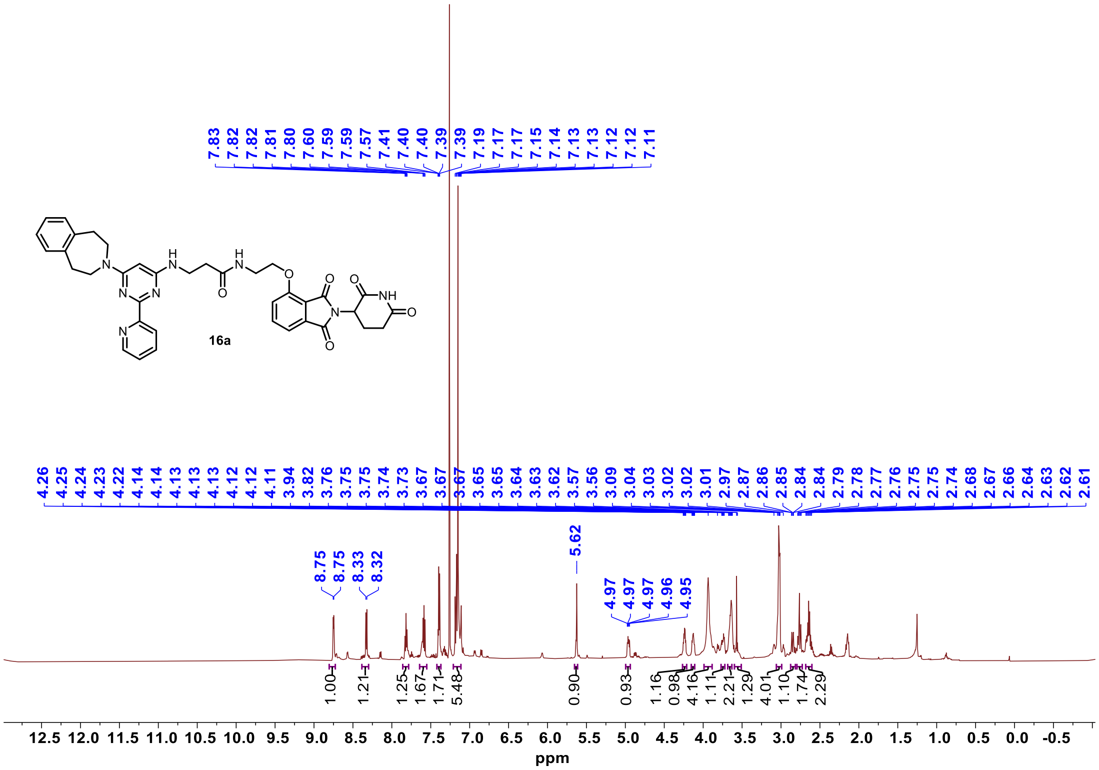

^{13}C NMR **16a**

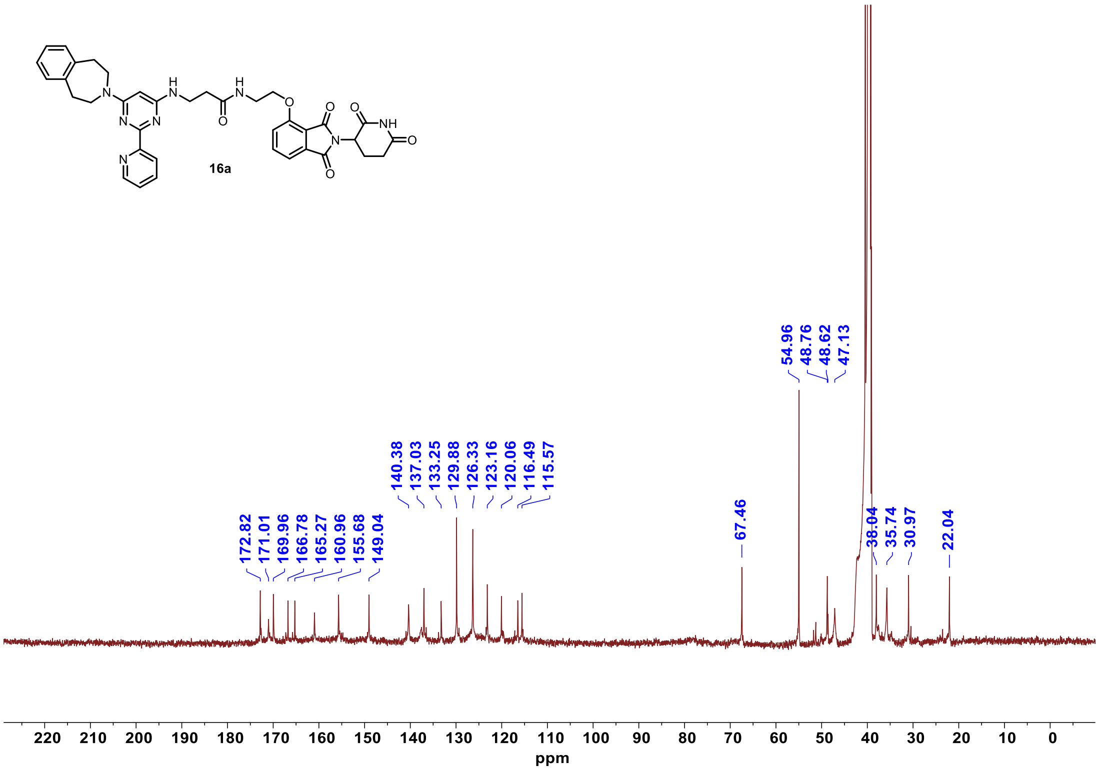

^{1}H NMR **16b**

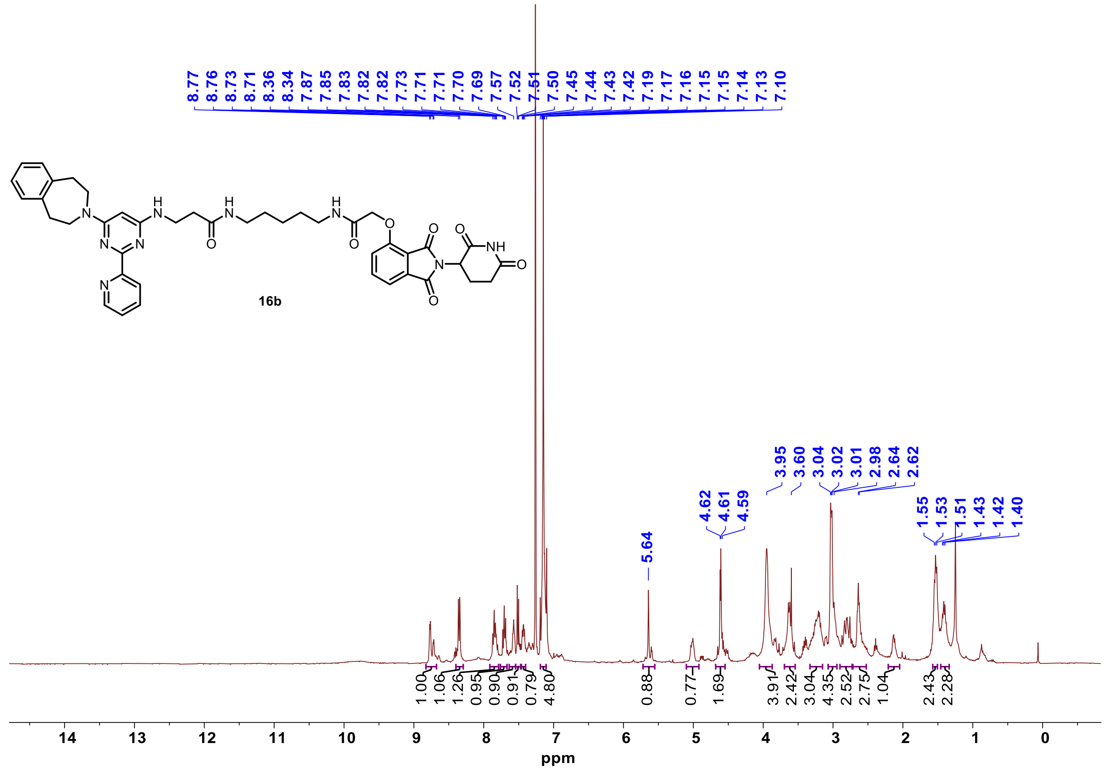

^{13}C NMR **16b**

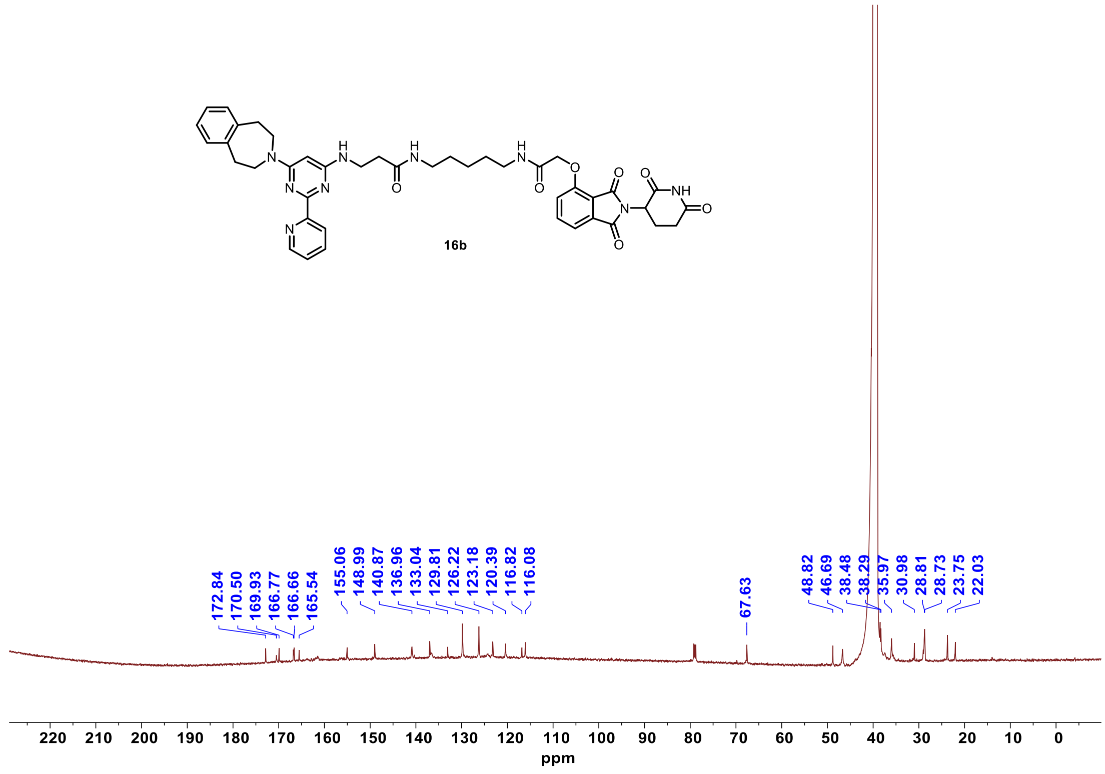

^{1}H NMR **16c**

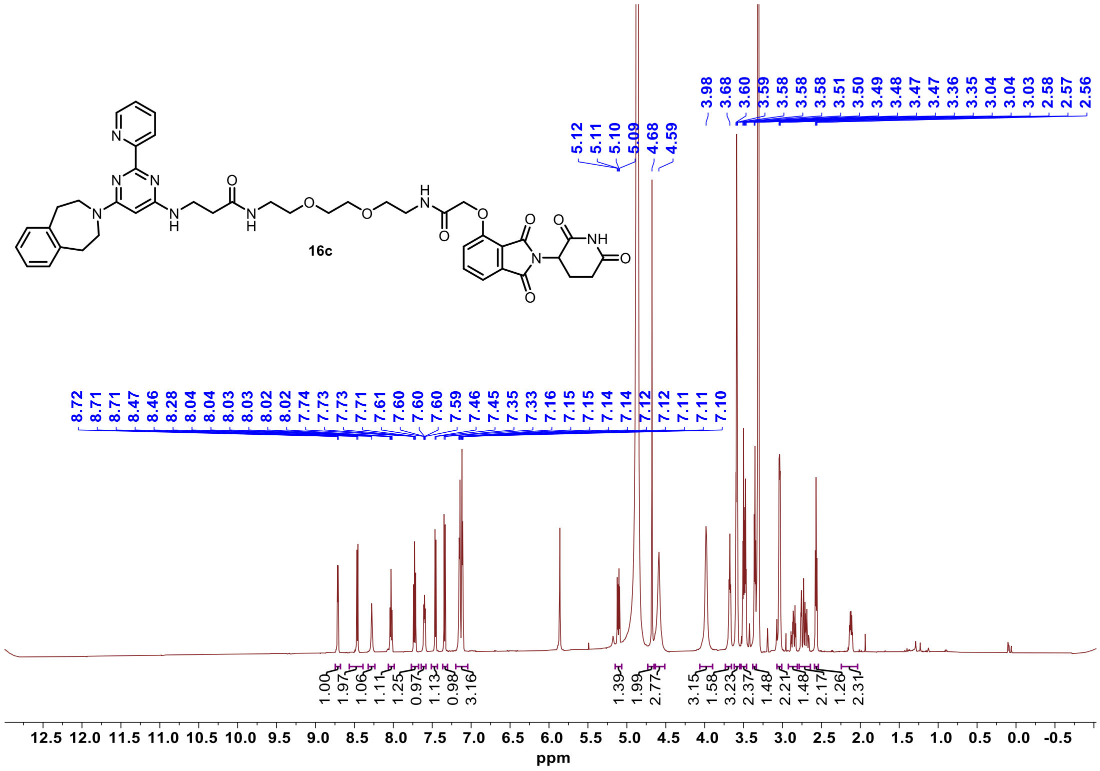

^{13}C NMR **16c**

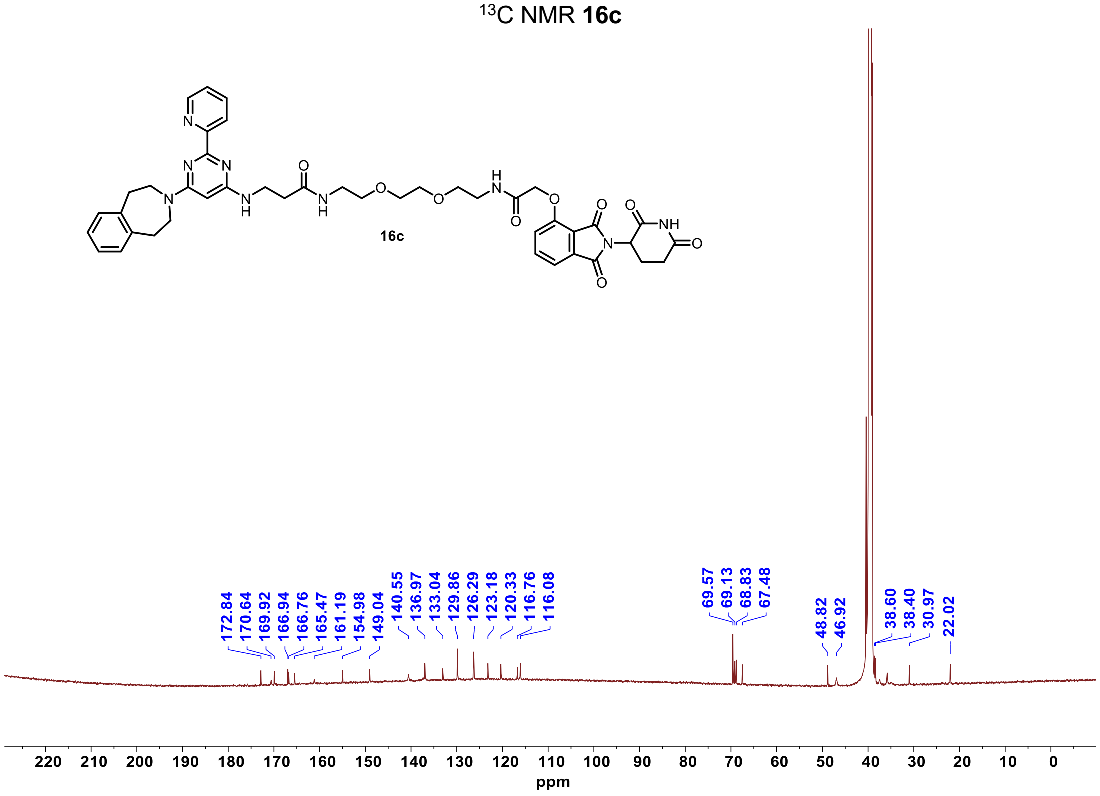

^{1}H NMR **16d**

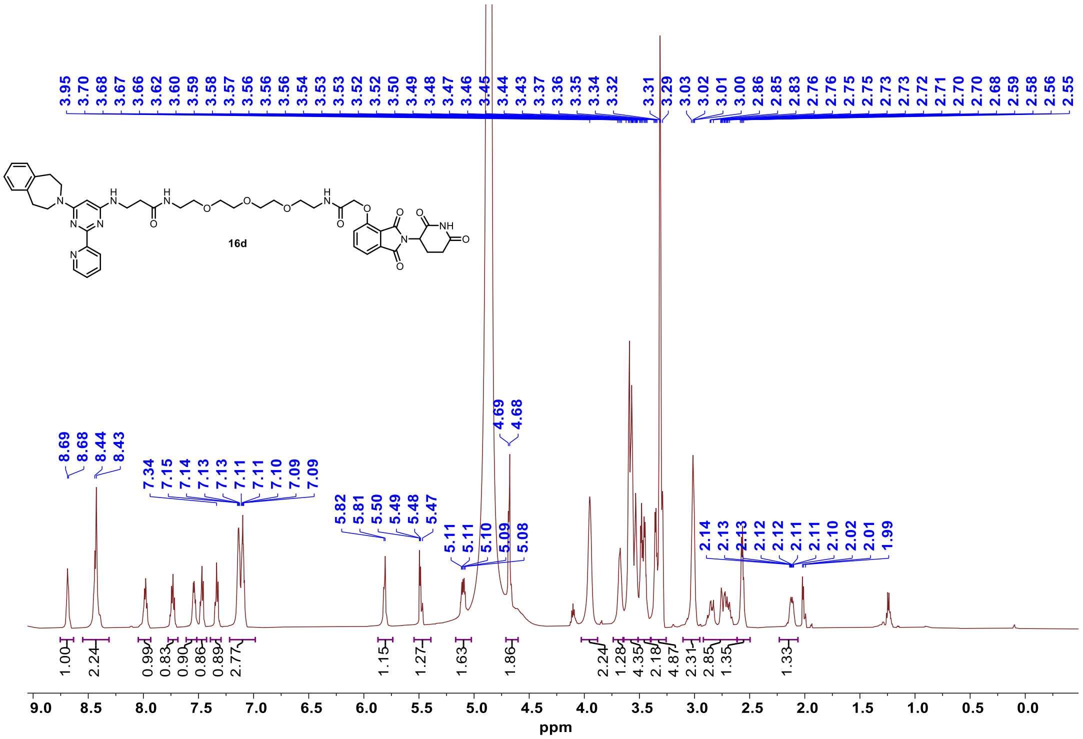

^{13}C NMR **16d**

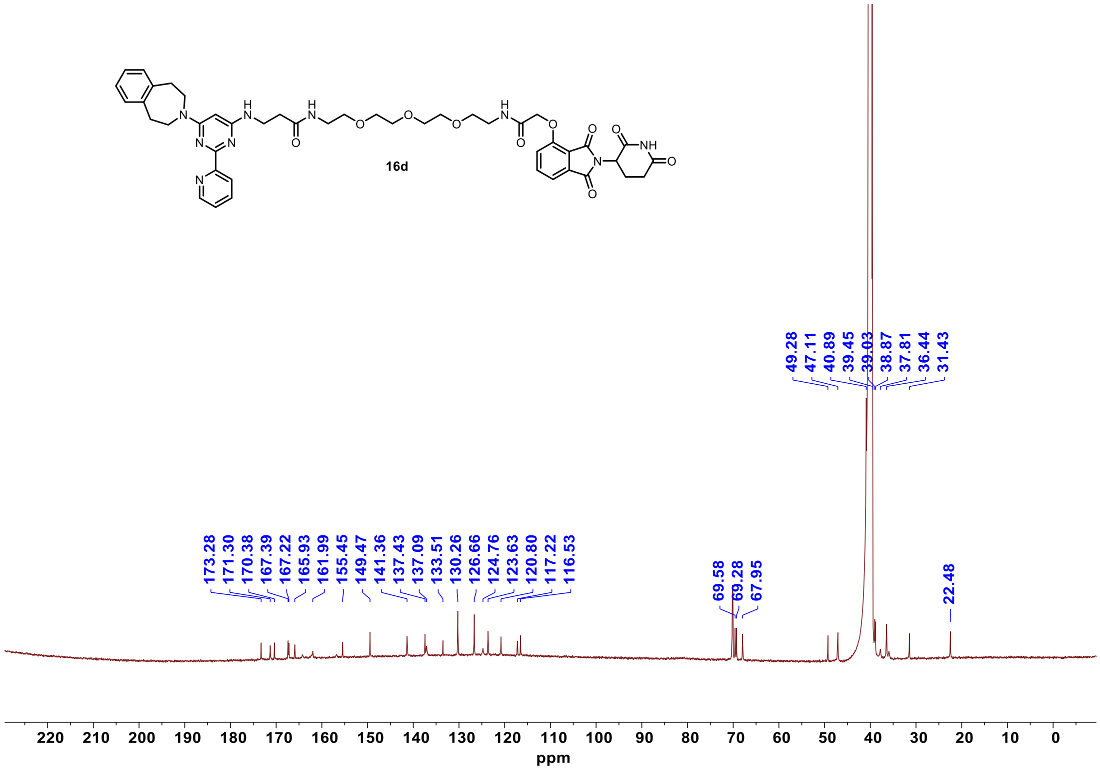

^{1}H NMR **17a**

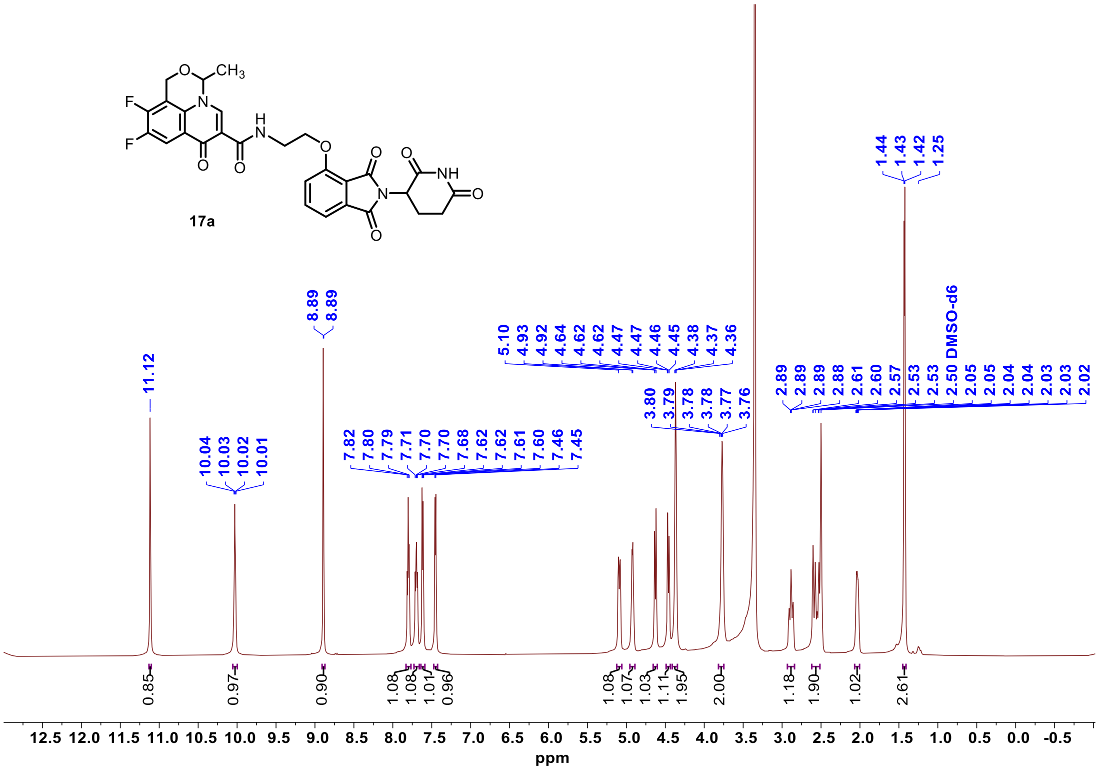

^{13}C NMR **17a**

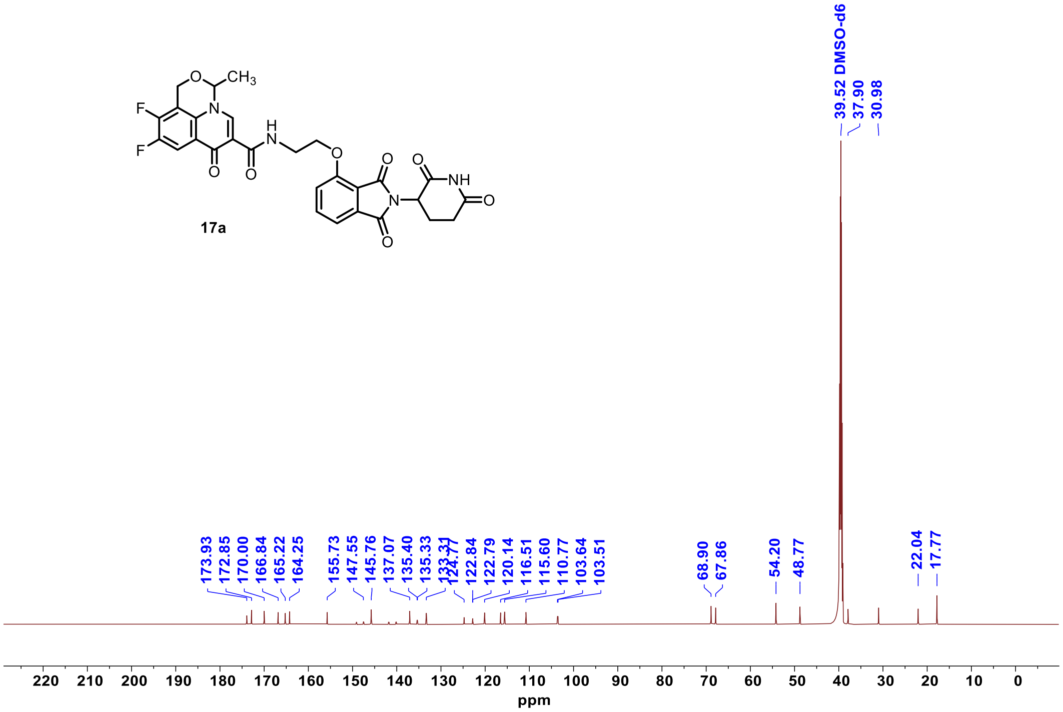

^{1}H NMR **17b**

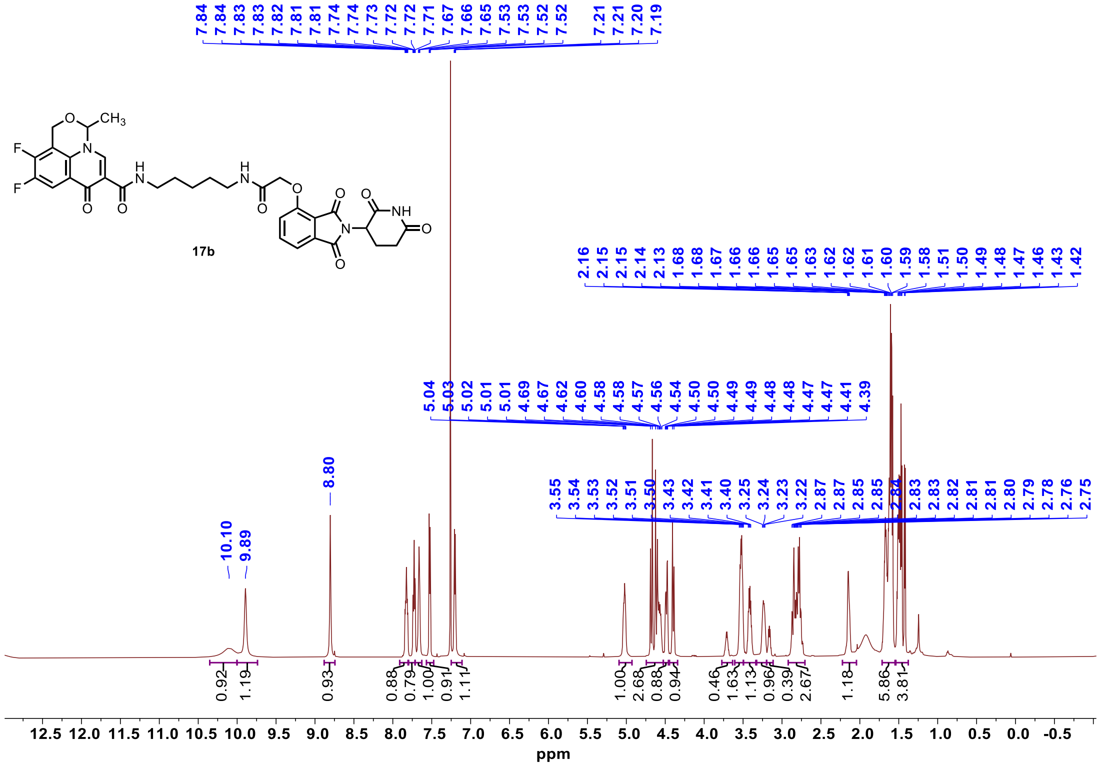

^{13}C NMR **17b**

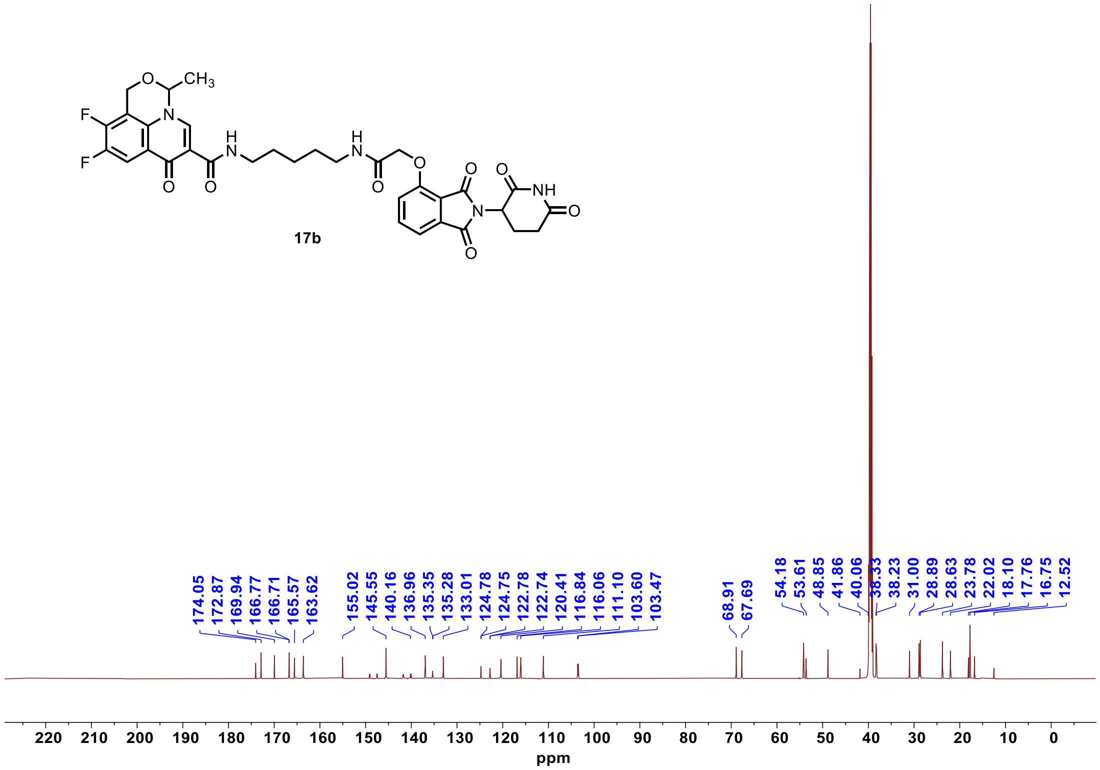

^{1}H NMR **17c**

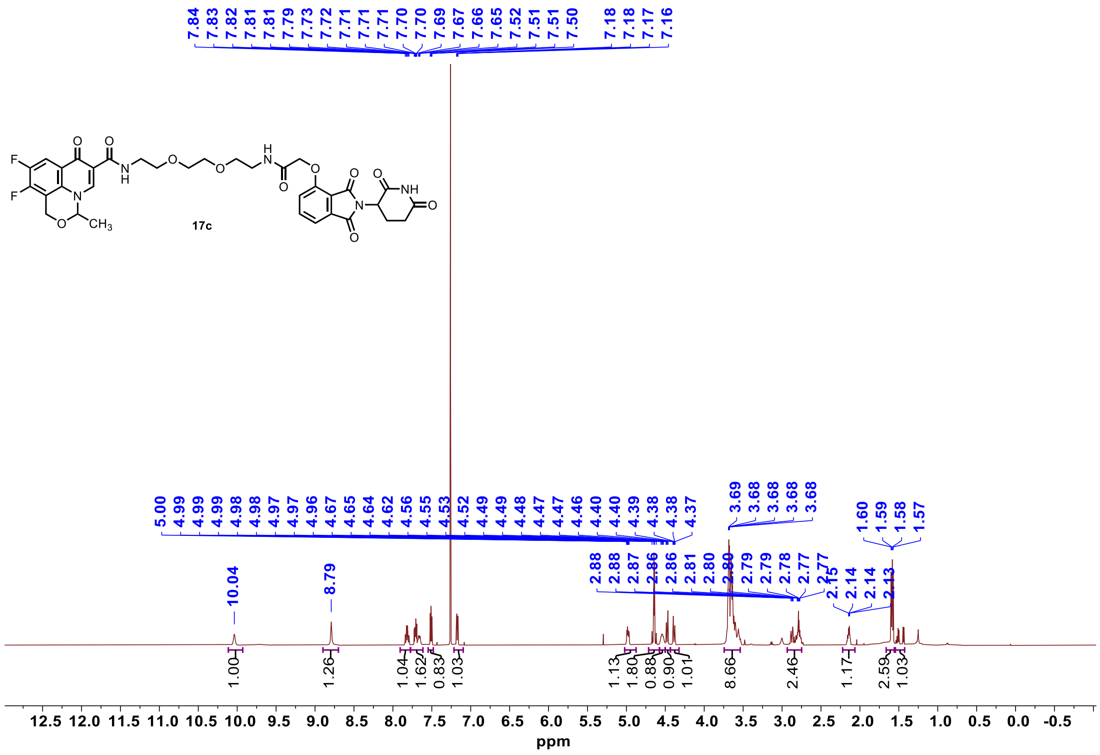

^{13}C NMR **17c**

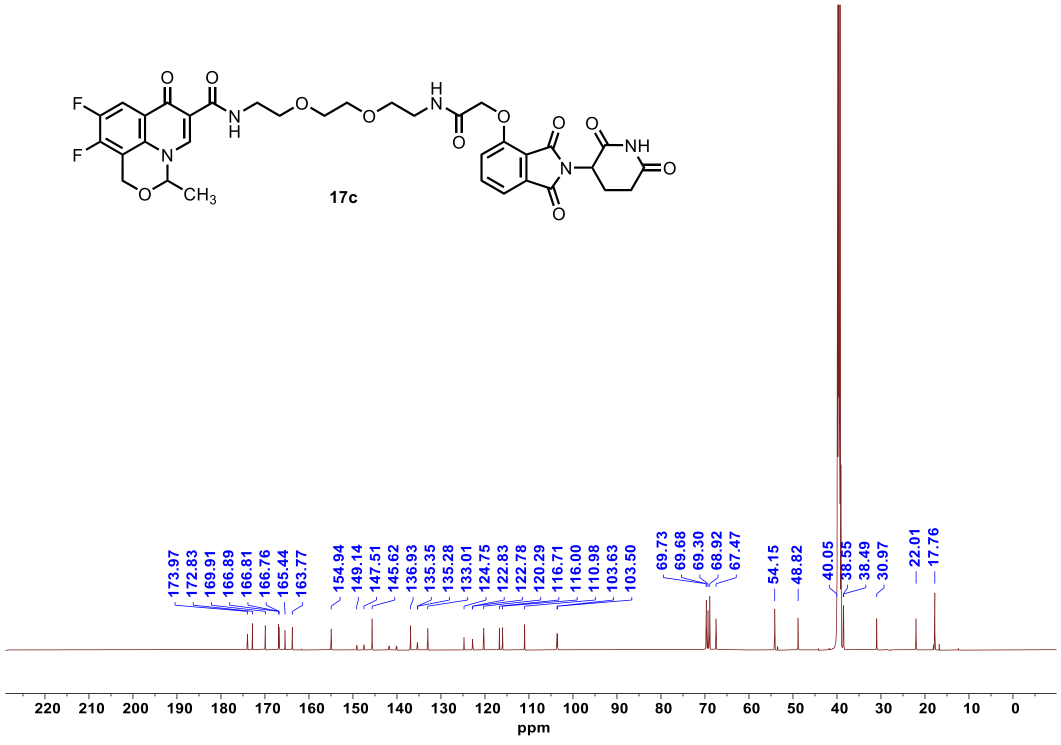

^{1}H NMR **17d**

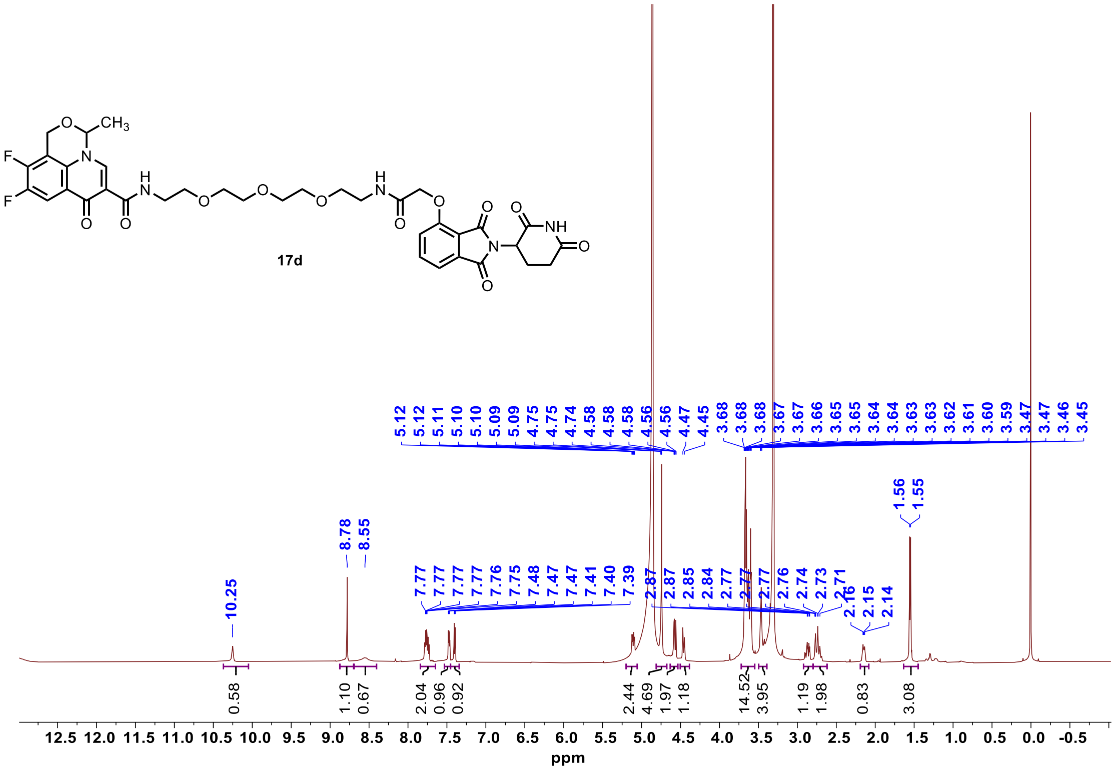

^{13}C NMR **17d**

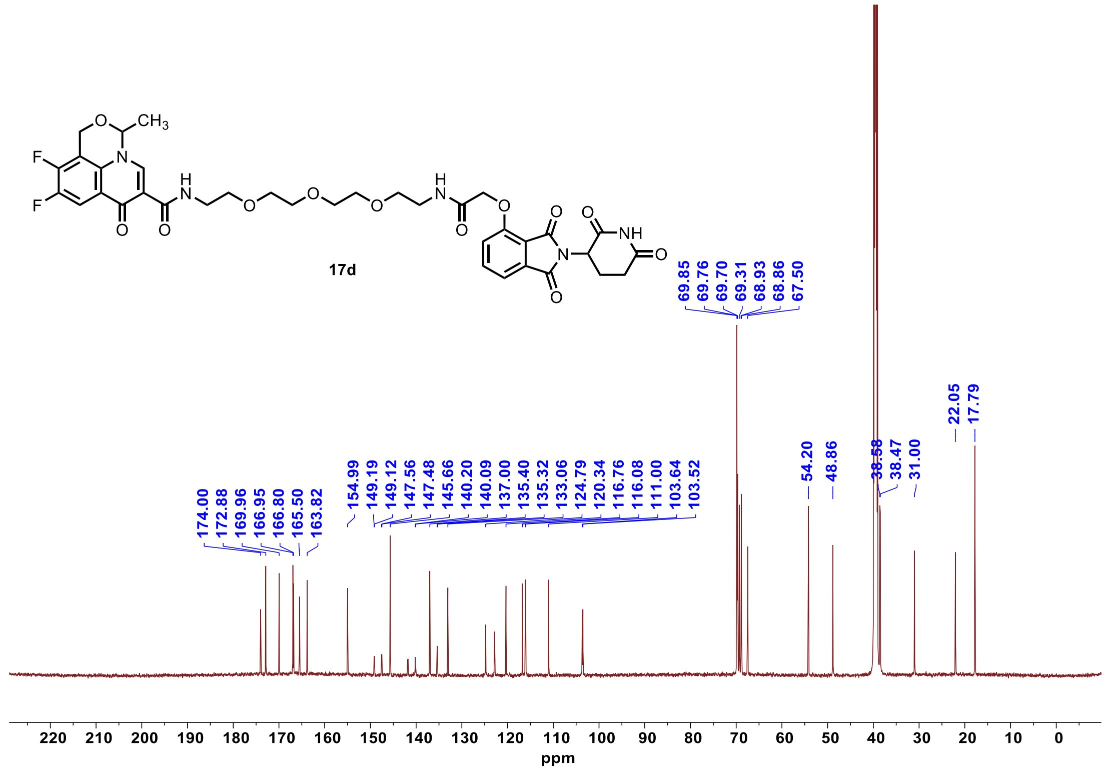

^{1}H NMR **18a**

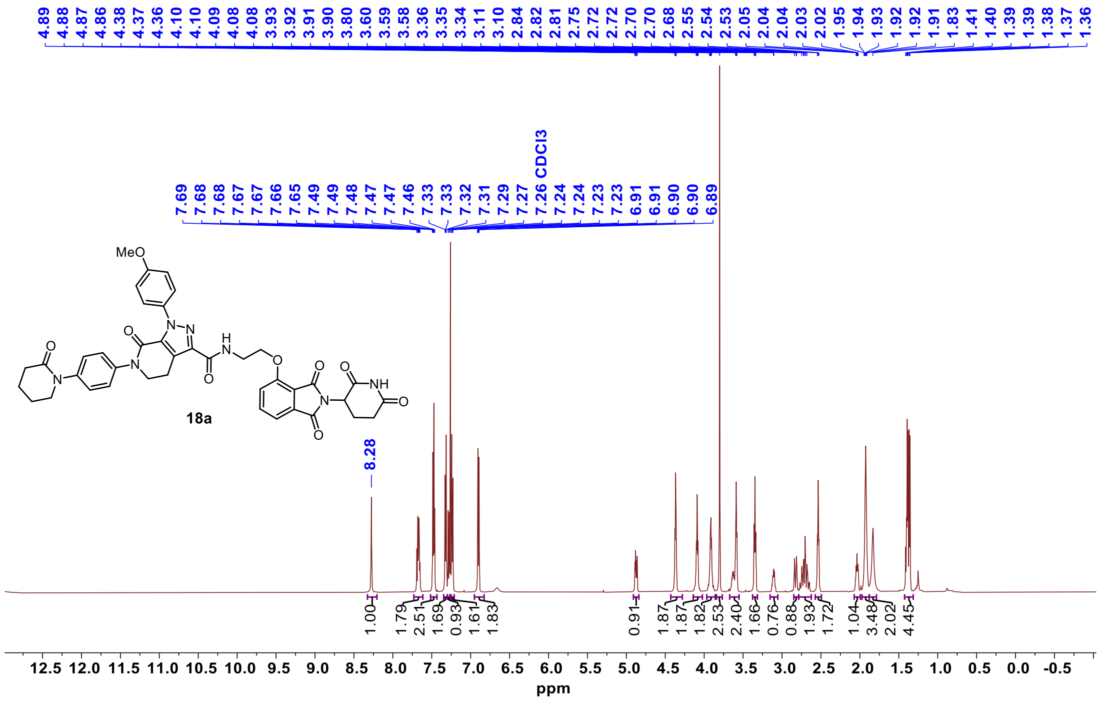

^{13}C NMR **18a**

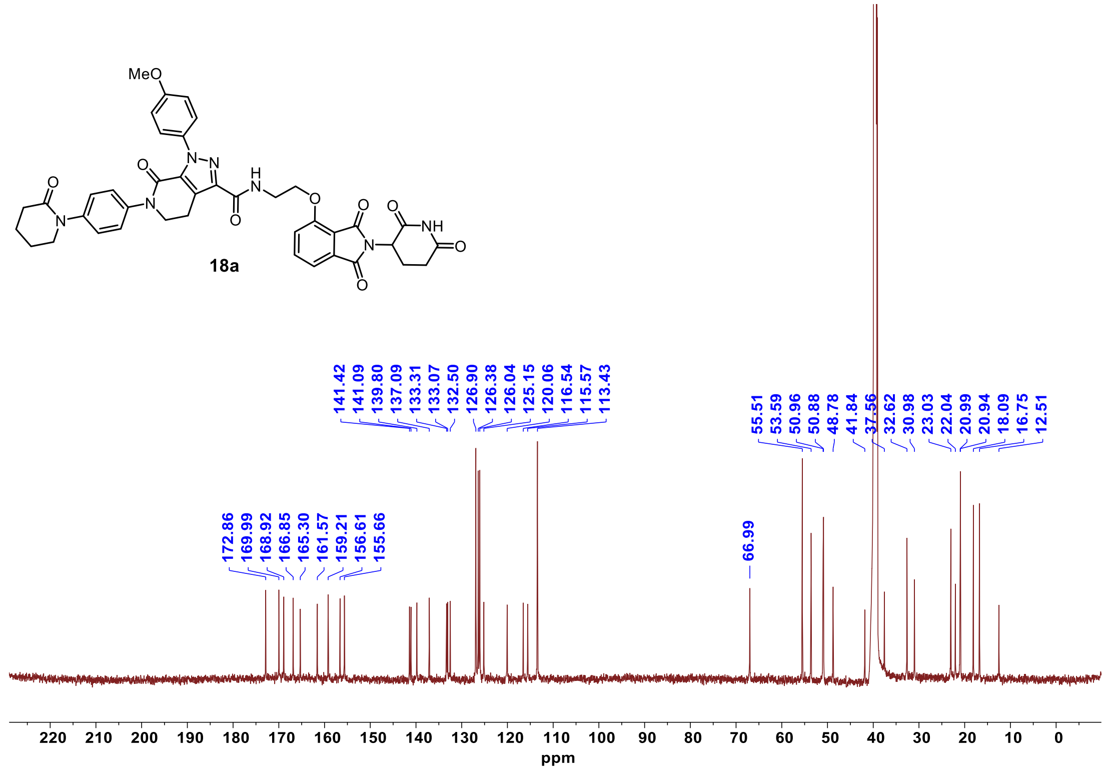

^{1}H NMR **18b**

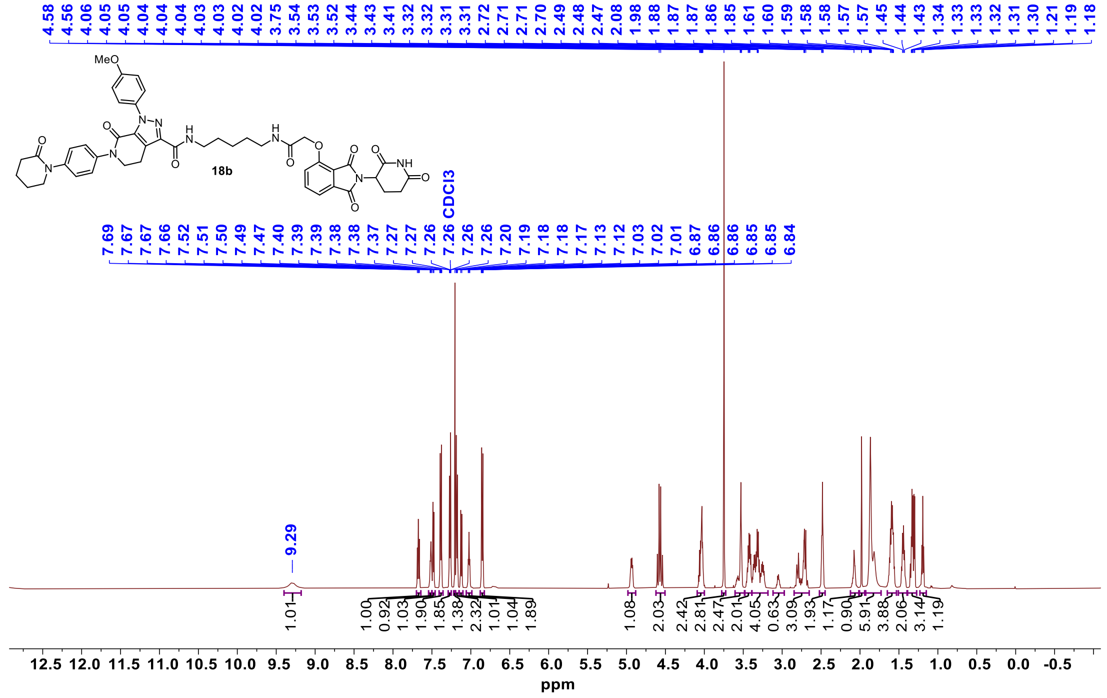

^{13}C NMR **18b**

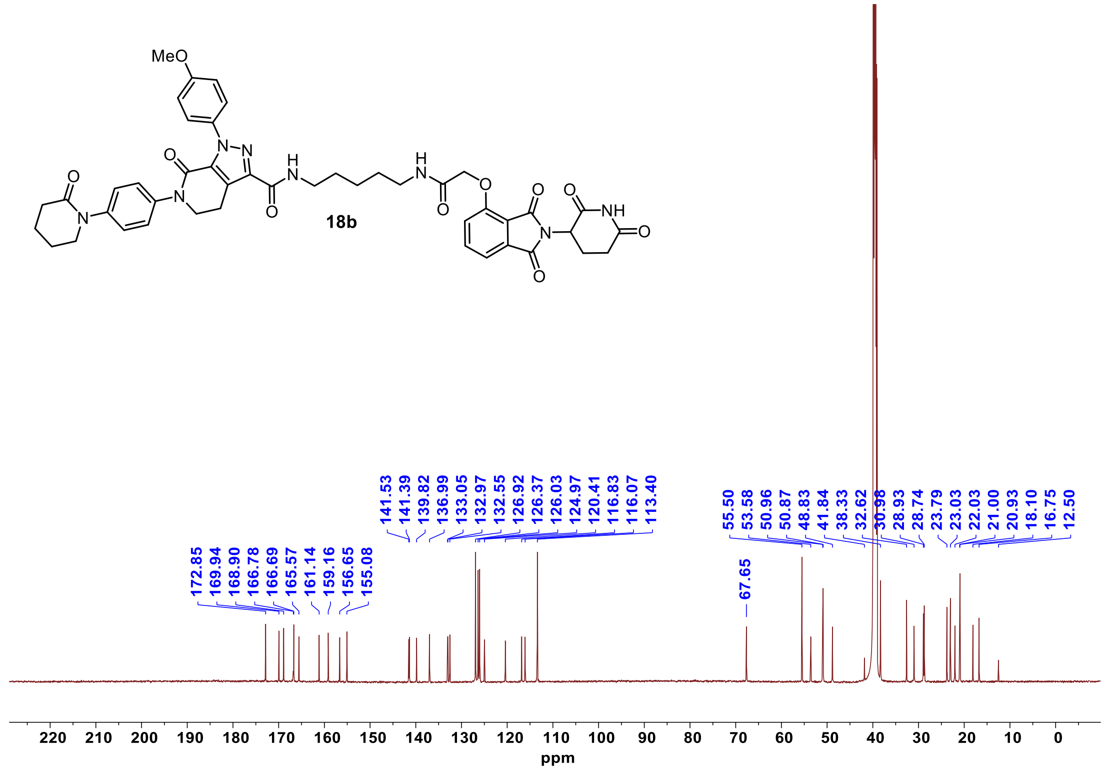

^{1}H NMR **18c**

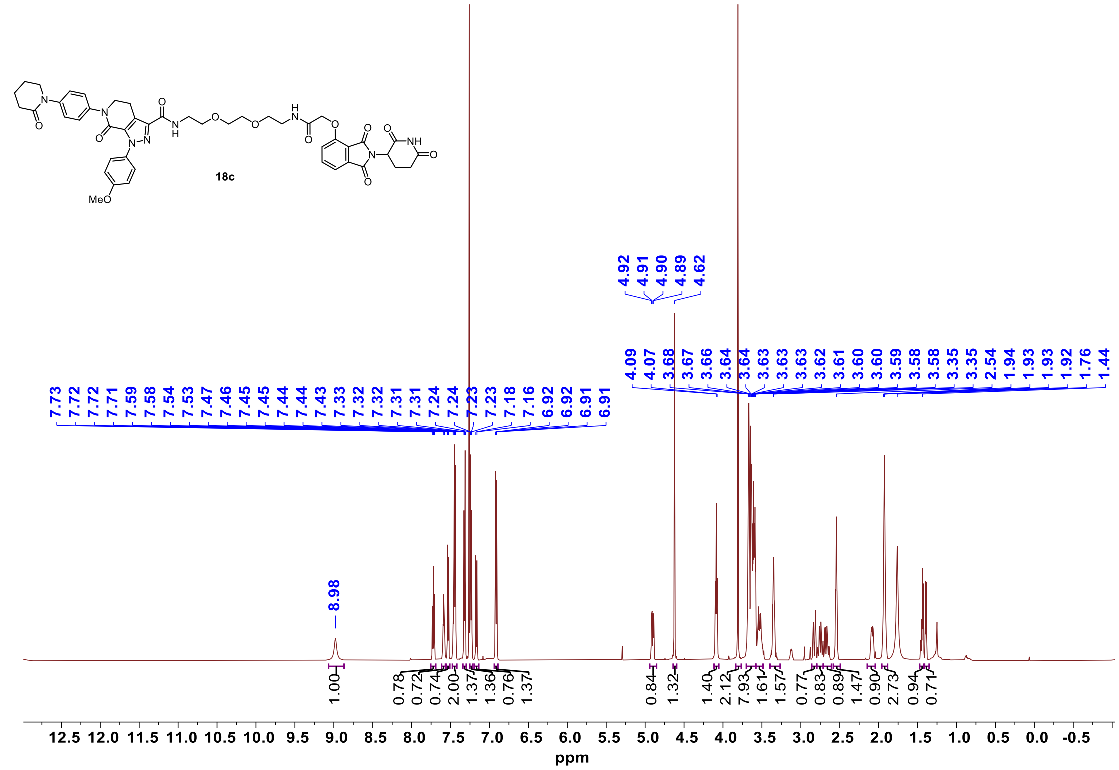

^{13}C NMR **18c**

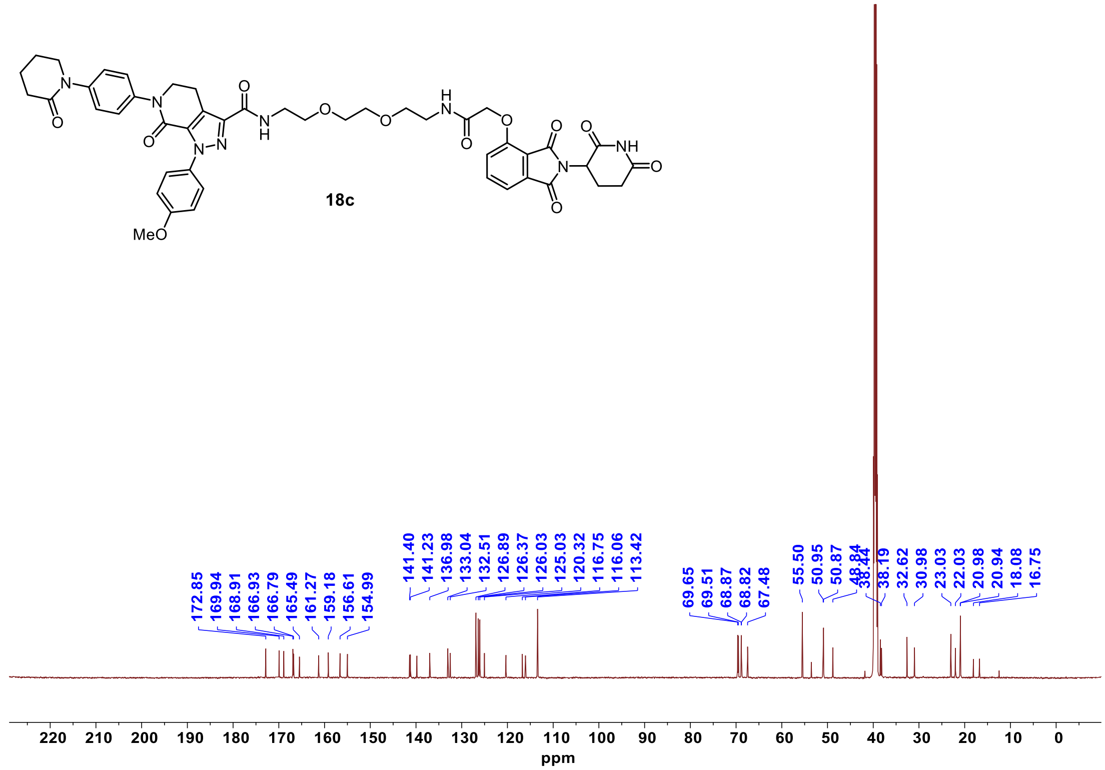

^{1}H NMR **18d**

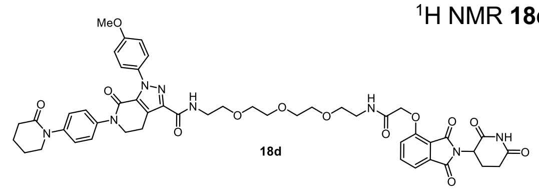

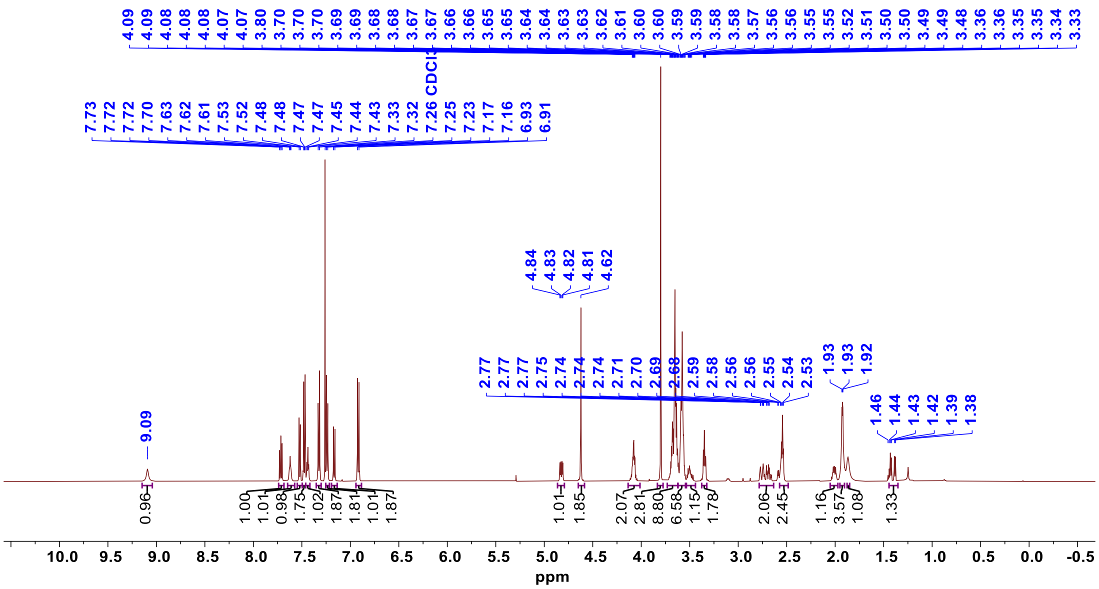

^{13}C NMR **18d**

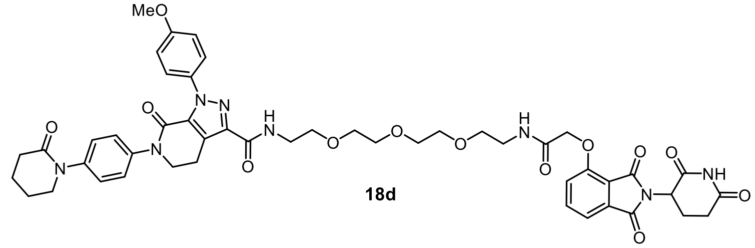

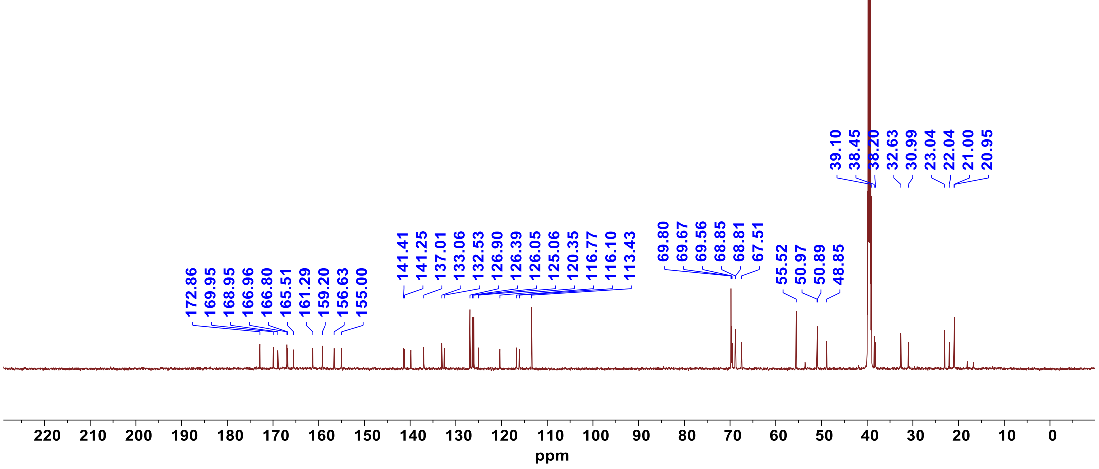

Supplement: Supplementary file 4 [file oc5c01594_si_004.pdf]
